# Chemoenzymatic Synthesis with Plant Oxidases and Metabolic Engineering Enable Rapid Access to Rare Gibberellins

**DOI:** 10.1021/jacs.6c06067

**Published:** 2026-04-29

**Authors:** Ahmed Arafa, Jennifer Gerke, Russell Cox, Jakob Franke

**Affiliations:** † Centre of Biomolecular Drug Research, 26555 Leibniz University Hannover, Schneiderberg 38, Hannover 30167, Germany; ‡ Institute of Botany, 26555 Leibniz University Hannover, Herrenhäuser Street 2, Hannover 30419, Germany; § Institute of Organic Chemistry, 26555 Leibniz University Hannover, Schneiderberg 1B, Hannover 30167, Germany; ∥ Pharmacognosy Department, Faculty of Pharmacy, Tanta University,Tanta 31527, Egypt

## Abstract

Gibberellins are structurally complex diterpenoid plant hormones with widespread agricultural applications. However, of the 136 known congeners found in nature, very few are easily accessible, preventing further research regarding their biological function. In biosynthesis, oxidases from plants generate the diverse oxidation patterns of natural gibberellins. Plant oxidases, in contrast to microbial oxidases, are very challenging to use for chemoenzymatic synthesis. Here, we develop a chemoenzymatic and metabolic engineering platform using plant oxidases that allows the production and full characterization of eight rare gibberellins as well as 16 *ent*-kaurene derivatives with various oxidation patterns. The filamentous fungus *Aspergillus oryzae* was engineered to produce common gibberellins and pathway intermediates in high titers, resulting in a panel of 20 diterpenoid substrates. These were screened using leaf disks from the model plant *Nicotiana benthamiana* producing ten different plant oxidases from diterpenoid metabolism. From the 200 substrate–enzyme pairs a total of 65 compounds were identified. We scaled up five reactions to produce milligram quantities of oxidized diterpenoids. Finally, by adding one of the plant oxidase genes to *A. oryzae*, we also accessed 15β-hydroxylated gibberellins in a single step by metabolic engineering. In summary, our work enables the flexible and sustainable synthesis of rare gibberellins and other highly oxidized diterpenoids. More importantly, our work demonstrates how plant oxidases can be used in chemoenzymatic synthesis and total biosynthesis campaigns, which will help to better utilize the catalytic potential of these previously neglected enzymes in the future.

## Graphical abstract

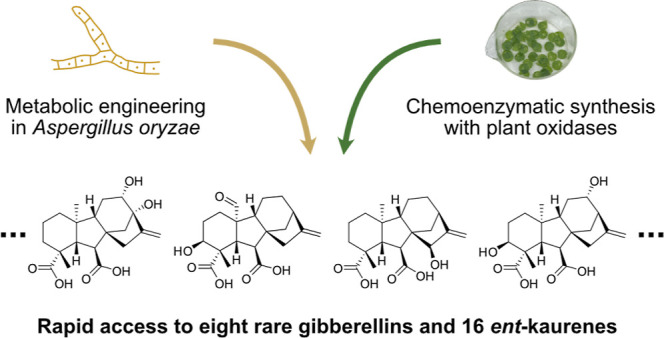

## Introduction

Gibberellins are potent plant hormones that regulate and control the growth and development of all vascular plants. In particular, their role in the development and maturation of flowers and seeds and their germination leads to important uses in agriculture and in plant science.^1,2^ However, most work has been focused on the main representatives of this plant hormone class, particularly GA_3_ (**1**, commonly known as gibberellic acid), GA_4_ (**2**), GA_7_ (**3**), and GA_1_ (**4**) (Figure 1), which have been investigated in detail and are widely applied.^3^ In contrast, a large proportion of the 136 currently known gibberellins^1,4^ are produced in low abundance in nature. For several minor gibberellin congeners there is not even fully reported NMR data available for this reason.^5–7^ This scarcity has prevented detailed biological evaluation. Rare gibberellins might play specialized roles in signaling. For example, it was discovered only in 2015 that the minor gibberellin GA_12_ (**5**), rather than one of the major congeners, acts as a long-distance transport form.^8,9^

**Figure 1:**
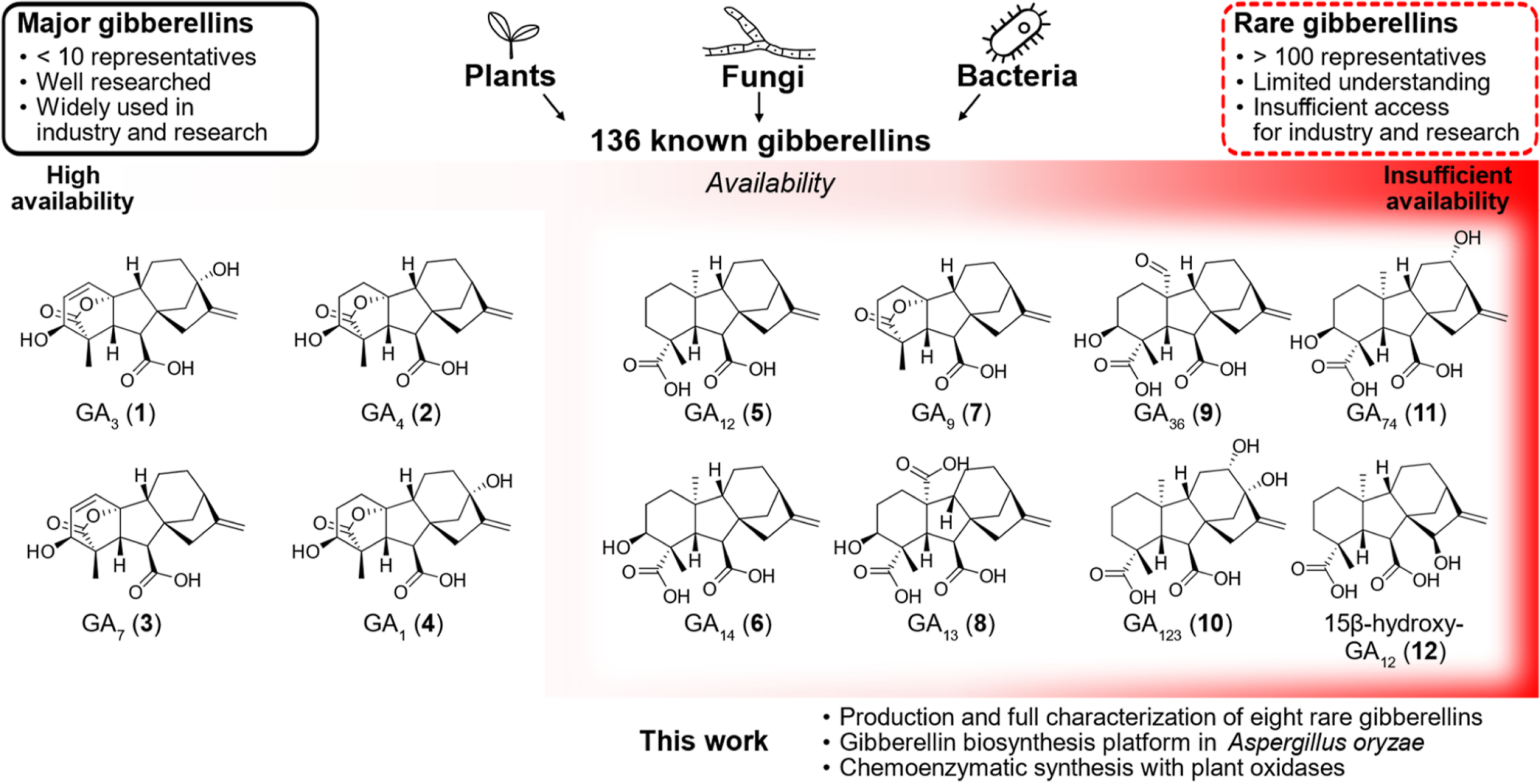
Gibberellins are a class of 136 diterpenoids produced by plants, fungi, and bacteria that function as plant hormones. While a few major gibberellins are well studied, the majority of rare gibberellins have not been investigated due to their very limited accessibility.

To enable a more comprehensive investigation of rare gibberellins, it is therefore crucial to develop new ways to access these structurally complex diterpenoids. Total chemical synthesis has been deployed for the production of gibberellins but involves many steps and is extremely resource intensive.^10^ For example, Mander’s chemical synthesis of GA_4_ (**2**) requires 15 steps from an advanced precursor.^11^ A more attractive option is to employ biotechnological production. Gibberellins are produced not only by plants but also by fungi^12^ and bacteria.^13^ The biosynthetic pathways of gibberellins in plants, fungi and bacteria are well understood.^1,14^ The fungus *Fusarium fujikuroi* (*Gibberella fujikuroi*) is known as a prolific producer of the major gibberellins, particularly GA_3_ (**1**),^2^ but it is not a good source of the minor gibberellins. More recently, *Escherichia coli* and yeast have been engineered to produce the main gibberellins.^15,16^ In the case of *E. coli* as a host, the engineered pathway starts from the valuable diterpenoid steviol rather than a simple carbon source such as glucose.^15^ In the case of yeast as a host, significant engineering was required to improve the formation of precursors and products and rare compounds were not produced.^16^ Gibberellins have also been produced *in vitro*, using purified biosynthetic enzymes, cofactors and substrates,^17^ but once again this is not an efficient process, requiring significant effort to provide the purified proteins and cofactors. All of these examples were used to produce a few main gibberellins, but the targeted (bio)­synthesis of rare congeners has not been demonstrated so far.

Here, we describe a chemoenzymatic and metabolic engineering system to access rare gibberellins, building on plant oxidases. First, we create an effective gibberellin total biosynthesis platform in the filamentous fungal host *Aspergillus oryzae* NSAR1 ^18^ to obtain a set of 18 diterpenoids that includes common gibberellins, rare pathway intermediates, and side or shunt products. Then, we set up a screening system using leaf disks of the model plant *Nicotiana benthamiana* to test these 18 substrates plus two commercially available compounds against a panel of ten plant oxidases. We demonstrate that this system can be scaled up for chemoenzymatic synthesis to produce milligram quantities of seven oxidized diterpenoids. In total, our work enabled the production and full characterization of eight rare gibberellins **5**–**12** in addition to 16 *ent*-kaurene derivatives (Table S1); more importantly, it describes an effective strategy for the use of plant oxidases in efficient chemoenzymatic synthesis and total biosynthesis campaigns.

## Results

### Establishment of a Gibberellin Production Platform in *A. oryzae*

Our first aim was to set up an *A. oryzae* system capable of producing major gibberellins, such as GA_3_ (**1**), GA_4_ (**2**), and GA_1_ (**4**) and related intermediates, which could be used as a starting point for chemoenzymatic modification to gain access to rare gibberellins (Figure 2). The diterpene *ent*-kaurene (**13**), itself derived from geranylgeranyl diphosphate (GGPP) (**14**), is the key precursor of gibberellins, and its biosynthesis is known in plants (e.g., the pathway to the potent anti-inflammatory oridonin),^19,20^ bacteria (e.g., the pathway to the antibacterial platensimycin)^21^ and fungi.^22^ To ensure good production of **13,** we expressed three genes in *A. oryzae* NSAR1 using the pTYGS·*argB* vector described by Lazarus and co-workers (Table S2).^23^ First, a gene encoding a truncated version of hydroxymethylglutaryl CoA reductase (tHMGR) from the plant *Avena strigosa* ^24^ was cloned downstream of the constitutive enolase promoter (P_*eno*_) to ensure high-level supply of the terpene precursors isopentenyl diphosphate (IPP) and dimethylallyl diphosphate (DMAPP).^24^ *A. oryzae* can already synthesize farnesyl diphosphate (FPP) (**15**) in good titers, but production of GGPP (**14**) is not constitutive.^25^ We therefore cloned the GGPP synthase gene *ggs2* from *F. fujikuroi* downstream of the strong constitutive alcohol dehydrogenase promoter (P_*adh*_). A similar strategy was successful during the biosynthesis of the diterpenoid pleuromutilin.^26^ Finally, *cps*/*ks* that encodes the bifunctional *ent*-copalyl diphosphate synthase/*ent*-kaurene synthase from *F. fujikuroi* was cloned downstream of the strong constitutive glyceraldehyde 3-phosphate dehydrogenase promoter (P_*gpdA*_) to create the vector pTYGS·*argB·AstHMGR·ggs2·cps*/*ks*. Integration of this vector into the genome of *A. oryzae* NSAR1 led to production of *ent*-kaurene (**13**) as expected; *ent*-copalyl diphosphate (**16**) was also shunted to compounds **17** and **18** (Figure 2). Comparison of corresponding fungal and bacterial terpene cyclase genes showed that all were functional, but the *cps*/*ks* from *F. fujikuroi* was the most active as judged by production of downstream metabolites (Figure S1).

**Figure 2:**
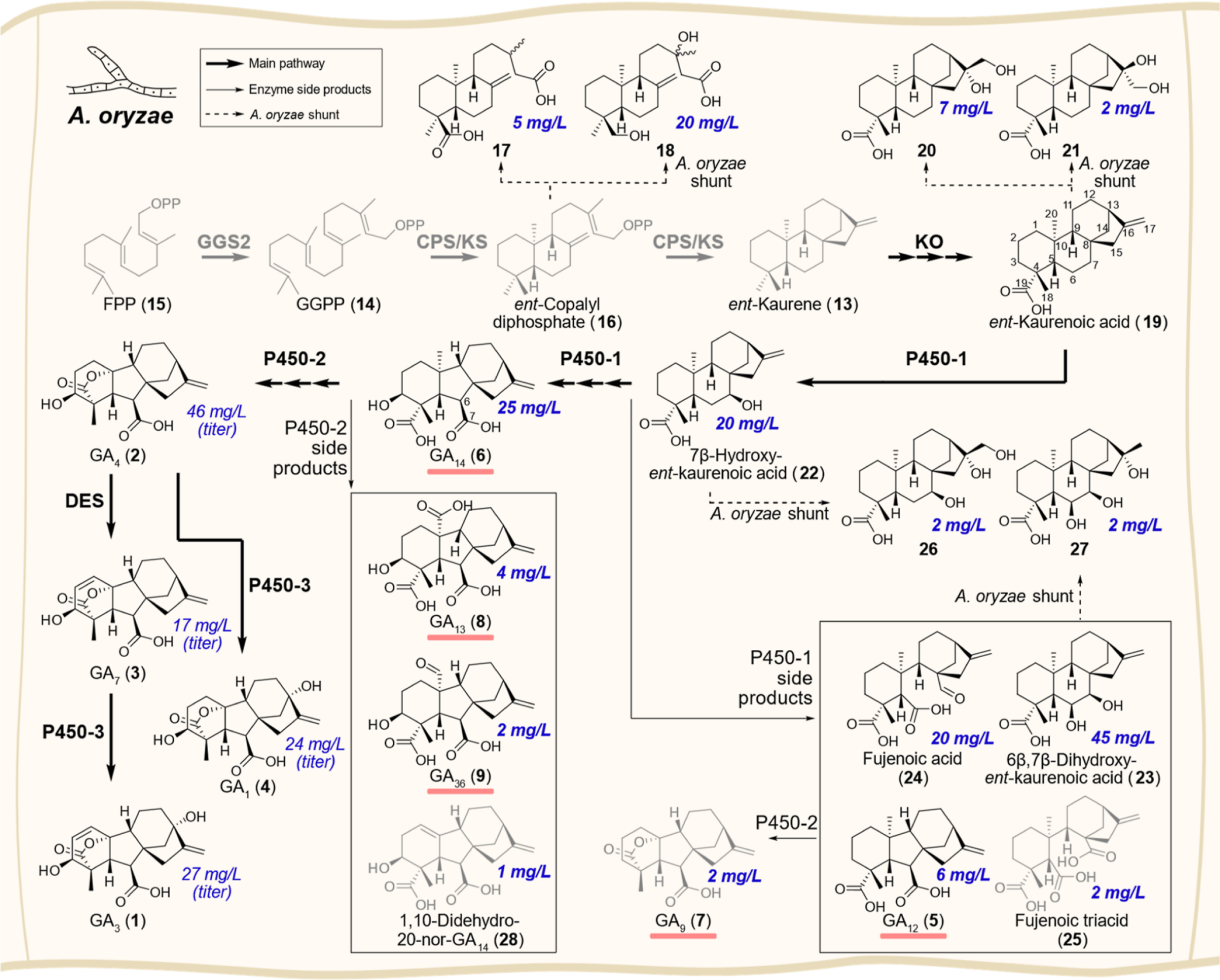
Development of a gibberellin total biosynthesis platform in *A. oryzae* NSAR1. Yields shown are isolated yields unless stated otherwise and refer to the maximum product levels across all created transformants containing different gene combinations. All compounds shown (except FPP (**15**) to *ent*-kaurenoic acid (**19**) and GA_7_ (**3**)) were isolated and fully characterized by NMR and HRMS (Table S1). GA_7_ (**3**) and *ent*-kaurenoic acid (**19**) were identified in comparison to reference compounds. Only compounds shown in black were subsequently screened with oxidases. Rare gibberellins are underlined in red. GGS2, Geranylgeranyl diphosphate synthase 2; CPS/KS, bifunctional *ent*-copalyl diphosphate synthase/*ent*-kaurene synthase; KO, *ent*-kaurene oxidase; DES, desaturase; FPP, farnesyl diphosphate; GGPP, geranylgeranyl diphosphate.

Next, *ent*-kaurene oxidase^27^ (KO) was added to the synthetic pathway. The *F. fujikuroi* *ko* gene was cloned downstream of P_*adh*_ in pTYGS·*adeA* to create pTYGS·*adeA*·*ko* (Table S2). Cotransformation of *A. oryzae* with this and the previous vector led to production of *ent*-kaurenoic acid (**19**) as expected, and a significant diminution of the previously observed shunt metabolites. However, two new shunts, the diols **20** and **21** were observed (Figure 2). Both were isolated and fully characterized by NMR spectroscopy. Addition of P450-1 to the system was achieved by cloning *P450-1* from *F. fujikuroi* downstream of P_*gpdA*_ to create pTYGS·*adeA*·*ko*·*P450-1*. Cotransformation of *A. oryzae* NSAR1 with pTYGS·*argB·AstHMGR·ggs2·cps/ks* and pTYGS·*adeA*·*ko*·*P450-1* then led to the synthesis of the first gibberellin intermediate GA_14_ (**6**) (25 mg/L isolated yield). P450-1 catalyzes a complex series of oxygenation and rearrangement reactions.^28^ The pathway begins by hydroxylation of C-7 to create 7β-hydroxy-*ent*-kaurenoic acid (**22**), that subsequently undergoes oxidative ring-contraction and 3β-hydroxylation to GA_14_ (**6**). However, these subsequent reactions are not well-controlled by P450-1 and the known side products 6β,7β-dihydroxy*-ent*-kaurenoic acid (**23**) (45 mg/L), GA_12_ (**5**) (6 mg/L), fujenoic acid (**24**) (20 mg/L), fujenoic triacid (**25**) (2 mg/L), and the two shunt products **26** and **27** were also observed and isolated from this transformant. Addition of *P450-2* from *F. fujikuroi*, by cloning it downstream of P_eno_ to create pTYGS·*adeA*·*ko*·*P450-1*·*P450-2* then led to the formation of GA_4_ (**2**) (46 mg/L titer) with minor amounts of GA_9_ (**7**) (2 mg/L), GA_13_ (**8**) (4 mg/L), and GA_36_ (**9**) (2 mg/L). Furthermore, we isolated very small amounts of an unusual 1,10-didehydro-20-nor derivative **28** of GA_14_ (1 mg/L); this compound has not been found in nature yet but was previously prepared as an intermediate for gibberellin synthesis.^29,30^

A third vector was used to clone the final two genes required for the synthesis of GA_3_ (**1**) (Table S2). The *F. fujikuroi* *des* gene encoding a nonheme iron-dependent 1,2-desaturase was cloned downstream of P_*adh*_ in the vector pTYGS·*sC* to give pTYGS·*sC*·*des*. Combination of this vector with the previous two then gave GA_7_ (**3**) (17 mg/L titer). Finally, inclusion of *P450-3* from *F. fujikuroi*, downstream of P_*gpdA*_ to give pTYGS·*sC*·*des*·*P450-3*, then afforded the final product of the pathway GA_3_ (**1**) (27 mg/L titer) (Figure S2). Alternatively, GA_1_ (**4**) (24 mg/L titer) is synthesized if *des* is not expressed. In total, 18 *ent*-kaurenes and gibberellins were isolated from the *A. oryzae* strains in sufficient quantities for full characterization and use as substrates for chemoenzymatic synthesis. Two further compounds, steviol (**29**) and isosteviol (**30**) (Figure 3) obtained by semisynthesis from stevioside, were also included, resulting in a panel of 20 diterpenoids as potential substrates for chemoenzymatic oxidations.

**Figure 3:**
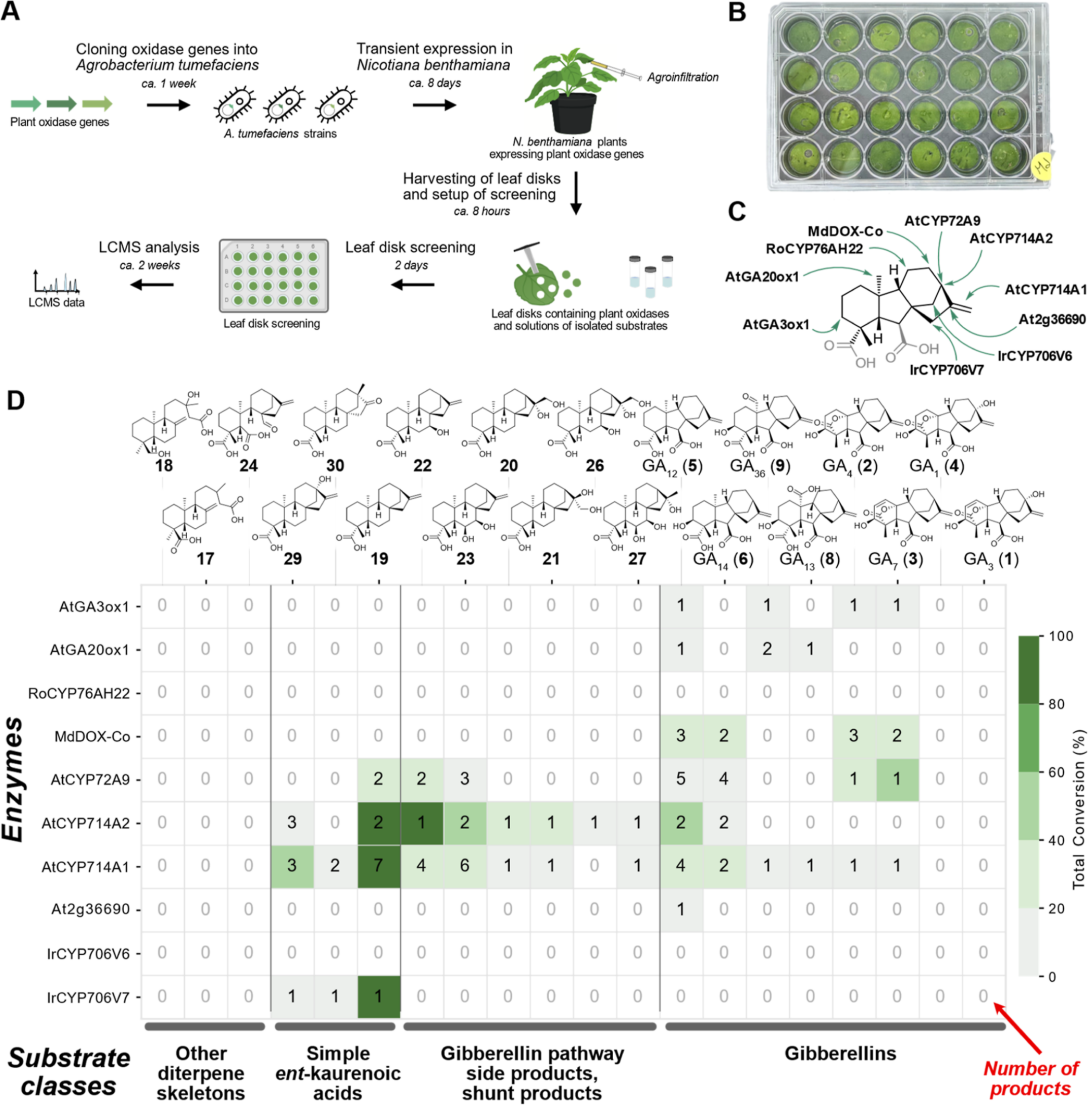
Screening the substrate scope of plant diterpenoid oxidases in leaf disks of the model plant *N. benthamiana*. (A) Schematic overview of workflow. (B) Photograph of a 24-well plate containing *N. benthamiana* leaf disks. (C) Selection of ten previously reported plant oxidases known to act on gibberellins and related diterpenoids (see also Table S3). The arrows indicate reported oxidation positions. (D) Results from leaf disk assay screening of ten plant oxidases against a panel of 20 diterpenoid substrates. Numbers in the cells indicate number of products observed by LC–MS. Color indicates total conversion of the substrate (based on sum of all product peaks). Detailed results of the leaf disk screening are presented in Figures S4–S10, Tables S4,S5, and Supporting Files 1 and 2.

### Screening Plant Diterpenoid Oxidases with a Leaf Disk Platform

With a set of 20 gibberellins and related diterpenes in hand, we next wanted to screen plant oxidases to find enzyme–substrate pairs that might lead to rare gibberellins. A key challenge of this strategy is that most oxidases known to act on diterpenoid backbones originate from higher organisms, particularly plants.^31,32^ The most important class of diterpenoid oxidases in plants are cytochrome P450 monooxygenases (CYPs),^31^ which possess a membrane anchor that severely hampers common *in vitro* or *E. coli*-based screening systems.^33–35^ Testing gene candidates directly in *A. oryzae* would be possible, but transformation of fungi is a relatively slow and low-throughput process. We therefore envisioned that a plant-based expression system building on the capacity of the model plant *N. benthamiana* for rapid transient expression^36^ combined with LCMS-based analysis would offer a fast and reliable way to screen plant oxidases against a substrate library (Figure 3A,B). The use of leaf disks instead of whole leaves or plants additionally would help to minimize substrate consumption.

As a proof of concept, we selected a panel of ten diterpenoid oxidases which are known or implicated to oxidize different carbon atoms of their substrates (Figure 3C).^20,37–45^ This set of enzymes comprised six CYPs and four nonheme iron oxoglutarate-dependent dioxygenases (ODDs). Six of these enzymes natively act on gibberellins, three on other *ent*-kaurenes, and one on other diterpenes (Figure 3C, Table S3).

In combination with the 20 diterpenoid substrates, this comprised a panel of 200 enzyme–substrate pairs. We anticipated that our screening approach would lead to the discovery of enzymatic reaction pairs that had not been previously described and would provide access to rare gibberellins. Technically, target oxidase genes were transiently expressed in *N. benthamiana* leaves.^36^ After 6 days, individual leaf disks (14 mm diameter) were cut from transformed leaves and placed singly in wells of a 24-well plate with HEPES buffer (500 μL) containing 60 μg of each test substrate (Figure S3). For substrates with low solubility in HEPES buffer at pH 7.5, the buffer pH was increased to 9.2 to improve solubility and 2–3% methanol or dimethyl sulfoxide were used as cosolvents. Plates were incubated in a phytochamber for 40 h and then analyzed by UPLC-MS.

In total, of the 200 tested enzyme–substrate pairs, 46 combinations resulted in the formation of enzymatic products (23%) that were not observed in empty vector transformed control leaf disks (Figure 3D, Tables S4 and S5, Figures S4–S10, Supporting Files 1 and 2). Twenty-four of the 46 productive combinations (52%) led to a single product, whereas in other cases up to seven different product peaks were observed (Figures 3D, S9, Table S4, Supporting Files 1 and 2). Only two of the ten tested enzymes, RoCYP76AH22 and IrCYP706V6, did not show activity with any of the tested substrates. Overall, this suggested that our leaf disk system provided a reliable means to produce active plant oxidases. *Vice versa*, five of the 20 tested compounds were not accepted by any enzyme; these were the three compounds with broken ring systems **17**, **18**, and **24** as well as the fully processed gibberellins GA_3_ (**1**) and GA_1_ (**4**).

A total of 91 peaks were identified from the 46 productive enzyme–substrate pairs, which were linked to 65 unique metabolites (Table S4, Supporting Files 1 and 2). Notably, 55 of the 91 product peaks (60%) exhibited mass shifts that corresponded to the addition of one or two oxygen atoms (+16 or +32 Da) (Table S4, Supporting Files 1 and 2). For 30 product peaks (33%), we observed mass differences of +18 and +34 Da (Table S4, Supporting Files 1 and 2), suggesting that nonredox processes or further reactions with background enzymes can also occur in the leaf disk system.

### Chemoenzymatic Synthesis with Plant Oxidases on Preparative Scale

The analytical scale assay showed that 7β-hydroxy-*ent*-kaurenoic acid (**22**) is oxidized by AtCYP714A1. To perform a preparative biotransformation, around 140 leaf disks from three different *N. benthamiana* plants expressing *AtCYP714A1* were pooled and incubated with 7β-hydroxy-*ent*-kaurenoic acid (**22**) (28 mg) dissolved in buffer (Figure 4A). After 6–7 days of incubation under the same conditions as before but with additional stirring, leaf disks were removed and reaction products were isolated by preparative HPLC. Three products were obtained: **31** (1.1 mg), **32** (1.6 mg), and **26** (0.7 mg), in addition to unreacted substrate (10.3 mg) (Figures 4B, S11). All products were fully characterized by NMR spectroscopy and HRMS (Table S1). Motivated by these results, we performed chemoenzymatic reactions with 6β,7β-dihydroxy-*ent*-kaurenoic acid (**23**) and either AtCYP714A1 or AtCYP714A2, resulting in 1.9 mg of **33** and 2.2 mg of **34**, respectively.

**Figure 4:**
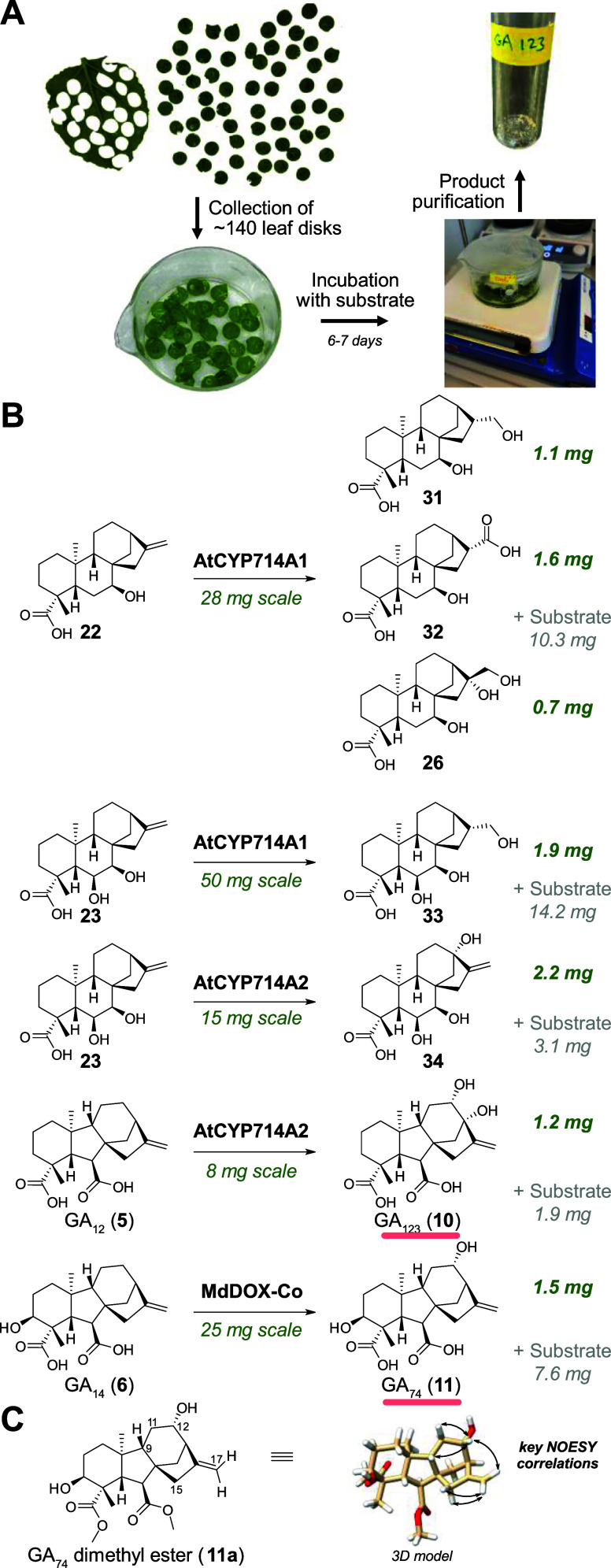
Chemoenzymatic synthesis of diterpenoids and rare gibberellins with plant oxidases produced in leaf disks of the model plant *N. benthamiana*. (A) Workflow for preparative leaf disk assays. (B) Preparative reactions. Rare gibberellins are underlined in red. (C) Key NOESY correlations for the dimethyl ester of GA_74_ (**11a**).

We then treated GA_12_ (**5**) (8 mg) with AtCYP714A2 leaf disks. This resulted in the formation of 1.2 mg of a dihydroxylated product **10**, which was fully characterized by NMR spectroscopy. This rare gibberellin GA_123_ (**10**) has been described from strawberries based on GC–MS fragmentation data,^5^ but was never isolated in sufficient amounts for full spectroscopic characterization or biological evaluation. Using the same approach, we also performed preparative leaf disk assays with the dioxygenase MdDOX-Co and GA_14_ (**6**) as a substrate. From 25 mg GA_14_ (**6**), 7.6 mg of unconsumed substrate was reisolated, and 1.5 mg of the 12α-hydroxylated product **11** was obtained. Its structure was confirmed as GA_74_ (**11**) by 2D NMR spectroscopy. GA_74_ has been previously identified from plants, but only via MS fragmentation data^46–48^ and has not been isolated in preparative amounts before. NOE spectroscopy of the corresponding dimethyl ester of GA_74_ (**11a**) confirmed the original tentative assignment^46,48^ of the 12-hydroxy group as α, particularly based on the NOESY correlation H12–H17a (Figure 4C). Lastly, we also tested the conversion of GA_14_ (**6**) with AtCYP714A2; low quantities (<0.5 mg) of a product were isolated, which was tentatively assigned as GA_18_ based on ^1^H NMR data (Figure S12, Table S6). These results clearly demonstrate that the leaf disk system enables chemoenzymatic reactions with membrane-bound enzymes from plants at preparative scale.

### Production of Rare Gibberellins in *A. oryzae* by Metabolic Engineering

Finally, we investigated if we could leverage information from our enzyme screening campaign to expand the *A. oryzae* gibberellin production platform toward the rational production of rare gibberellins in a single process. For this purpose, we selected IrCYP706V7 based on the very good conversion yields that we observed in the leaf disk system. First, we confirmed that IrCYP706V7 was active in *A. oryzae* by introducing the corresponding gene into a strain producing *ent*-kaurenoic acid but not containing P450-1 and P450-2 required for the further conversion of this intermediate into gibberellins. A gene encoding cytochrome P450 reductase AtCPR1 ^49^ was also included as a redox partner. Indeed, this led to formation of 15β-hydroxy-*ent*-kaurenoic acid (**35**) as the single major product at a titer of 63 mg/L (Figure S13).

Next, in the hope of accessing rare 15β-hydroxylated gibberellins, we generated an *A. oryzae* strain containing *IrCYP706V7*, *P450-1*, and *P450-2* (Figure 5A). Compared to a strain with *P450-1* and *P450-2* but without *IrCYP706V7*, several new peaks were present, but at relatively minor levels (Figure 5B). The same major products such as GA_4_ (**2**) as in the absence of *IrCYP706V7* were obtained. Only one clear new peak was observed, which was identified as 7β,15β-dihydroxy-*ent*-kaurenoic acid (**36**). P450-1 catalyzes a multistep reaction cascade that first involves 7β-hydroxylation followed by contraction of the B-ring, 3β-hydroxylation and C-7 oxidation.^28^ The accumulation of 7β,15β-dihydroxy-*ent*-kaurenoic acid (**36**) in our system suggested that the presence of a 15β-hydroxy group hinders further progress of the P450-1 reaction cascade after the initial 7β-hydroxylation (Figure 5A). Particularly due to the competition with the native favored intermediate 7β-hydroxy-*ent*-kaurenoic acid (**22**), the generation of 15β-hydroxylated gibberellins was therefore limited in efficiency. Nonetheless, the presence of multiple new peaks upon addition of *IrCYP706V7* suggested that P450-1 and P450-2 were in principle capable of accepting 15β-hydroxylated intermediates. Indeed, we managed to isolate and fully characterize one of the products, 15β-hydroxy-GA_12_ (**12**) (Table S1). Five further 15β-hydroxylated gibberellins occurred in trace amounts: 15β-hydroxy-GA_14_ (**37**), GA_45_ (**38**), GA_63_ (**39**), GA_64_ (**40**), and GA_65_ (**41**) were identified based on their GC–MS fragmentation patterns in comparison to literature data (Figure S14, Table S5). In the case of GA_65_ (**41**), we isolated enough material to obtain a ^1^H spectrum that showed an aldehyde proton (Figure S15) in support of this assignment. Besides 7β,15β-dihydroxy-*ent*-kaurenoic acid (**36**), the four additional 15β-hydroxylated *ent*-kaurene derivatives **42**, **43**, **44**, and **45** were also isolated and characterized by NMR spectroscopy (Table S1).

**Figure 5:**
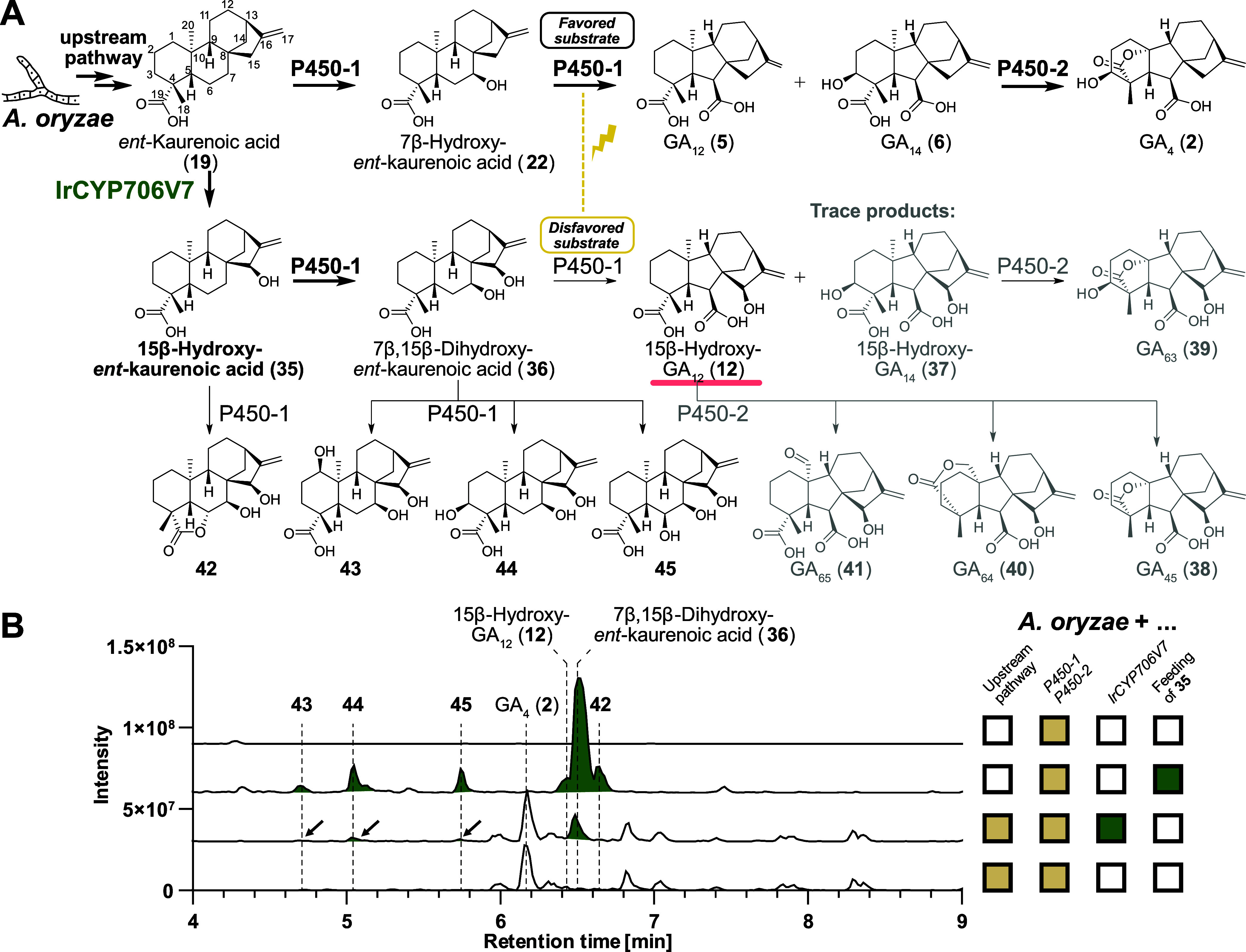
Formation of rare 15β-hydroxylated gibberellins in *A. oryzae* with IrCYP706V7. (A) Pathway to 15β-hydroxylated *ent*-kaurenes and gibberellins based on IrCYP706V7 oxidation of *ent*-kaurenoic acid (**19**). Trace products determined by GC–MS fragmentation patterns are shown in gray. All other compounds shown were isolated and verified by NMR spectroscopy. (B) Base peak chromatograms (negative mode) showing the formation of new peaks dependent on IrCYP706V7 (green-filled peaks and/or black arrows) in *A. oryzae*. The 15β hydroxy group hinders further progress of the P450-1 reaction cascade after the initial 7β-hydroxylation. The competition against the favored native intermediate 7β-hydroxy-*ent*-kaurenoic acid (**22**) can be circumvented by biotransformation of the unnatural intermediate 15β-hydroxy-*ent*-kaurenoic acid (**35**) (highlighted in bold) in an *A. oryzae* strain only containing *P450-1* and *P450-2* genes but not genes for *ent*-kaurenoic acid biosynthesis. The brown colored checkboxes refer to fungal genes, whereas the green checkboxes refer to the plant gene *IrCYP706V7* and feeding of the desired IrCYP706V7 product 15β-hydroxy-*ent*-kaurenoic acid (**35**). Rare gibberellins are underlined in red.

As a potential solution to circumvent this competition dilemma between 15β-hydroxylated and nonhydroxylated intermediates, we also tested a biotransformation strategy. For this purpose, we added isolated 15β-hydroxy-*ent*-kaurenoic acid (**35**) to *A. oryzae* strains harboring *P450-1* and *P450-2* genes but not the upstream pathway required to make *ent*-kaurenoic acid (**19**) (Figure 5A). Gratifyingly, the new peaks from the strain containing *IrCYP706V7* together with the full gibberellin pathway were also present in the strain with the partial gibberellin pathway upon feeding of 15β-hydroxy-*ent*-kaurenoic acid (**35**) but much more intense (Figure 5B). Under these conditions compound **35** was fully consumed. These results indicate that this biotransformation approach can be a viable and clean alternative to access 15β-hydroxylated gibberellins with the help of metabolic engineering in *A. oryzae*.

## Discussion

Individual plant species contain typically several hundred different CYPs.^50^ These oxidases have evolved to become pivotal factors for the generation of structurally diverse complex natural products, including diterpenoids.^31^ However, their accessibility for enzyme screening campaigns and chemoenzymatic synthesis has been severely limited so far. The most challenging aspect of plant CYPs is the fact that they are membrane-bound,^51^ hindering their production in simple prokaryotic hosts such as *E. coli* or in cell-free systems.^34^ Accordingly, previous work where oxidases were successfully screened and applied for chemoenzymatic synthesis of natural products^52–55^ had to be restricted to microbial CYPs and other classes of oxidases,^56^ thereby neglecting the large biocatalytic potential of plant oxidases. To address this unresolved dilemma of potential usefulness of plant CYPs but lack of a suitable screening system, we presented here a system based on *N. benthamiana* leaf disks in which the target oxidases are produced directly *in vivo*. Notably, of the tested ten diterpenoid oxidases, eight showed activity on one or more of the 20 tested substrates. The two oxidases that were not active with the tested substrates were RoCYP76AH22 and IrCYP706V6. While RoCYP76AH22 was previously shown to act broadly on diterpene scaffolds belonging to the abietane, labdane, and pimarane classes, no activity on *ent-*kaurenes is known for this enzyme.^43,57^ As such, our data suggests that the previously noted broad substrate spectrum of RoCYP76AH22 does not extend to *ent*-kaurene diterpenes. IrCYP706V6, on the other hand, was reported to hydroxylate *ent*-kaurene,^20^ and complete lack of activity for this enzyme with the tested substrate panel was therefore surprising to us. To verify that this is not caused by a failure of our expression system, we performed a control experiment to produce *ent*-kaurene in situ; indeed, this experiment confirmed that IrCYP706V6 produced in *N. benthamiana* was in principle active (Figure S16) and simply did not accept *ent*-kaurene derivatives of higher oxidation states. Overall, the data demonstrates that our leaf disk screening platform is a very reliable way to generate active plant oxidases with little experimental workload.

Of the 20 tested substrates, 15 were transformed by at least one of the tested enzymes, showing that uptake and conversion of diverse substrates into the leaf disks is overall efficient. Three of the substrates which were not accepted, compounds **17**, **18**, and **24,** exhibit disrupted ring systems, which likely explains why they were not converted. The other two compounds not turned over were the gibberellins GA_3_ (**1**) and GA_1_ (**4**), which were already the most highly functionalized substrates of our panel. Thanks to the leaf disk format performed in well plates, relatively low quantities of substrates are needed for these assays: Only 0.2 mg substrate per enzyme to be tested were sufficient to carry out measurements in triplicate. We also demonstrated that this system can be easily scaled up for preparative applications in the 8–50 mg substrate scale (Figure 4B).

Compared with traditional single-host approaches in microbial hosts, our leaf disk screening platform offers three major advantages (Figure 6): First, as a plant host, *N. benthamiana* is much more reliable to assess the activity of plant oxidases than a microbial host. Indeed, even though IrCYP706V7 showed very good activity in *A. oryzae* (Figures 5, S13), this was not consistent for other oxidases; for AtCYP714A2, we observed much lower activity in *A. oryzae* than in *N. benthamiana* (Figure S17) and for MdDOX-Co no activity in the fungal host at all (Figure S18). Second, screening in *N. benthamiana* is substantially faster than in *A. oryzae*. Even if episomal vectors were used, fungal transformation is an inherently slow process compared to transient expression in *N. benthamiana* ^36^ (Figure S19). Third, screening the enzymes with isolated substrates offers a very clean background, which helps to accurately identify enzymatic activity. This is in stark contrast to direct screening in the original host strain, where co-occurring pathway intermediates and shunt products lead to substantial peak overlap and competing routes which overshadow intrinsic enzymatic activity (Figure S20). Nonetheless, as our results for IrCYP706V7 demonstrate, the leaf disk screening results are an excellent starting point to make informed decisions which enzymes are worthwhile for reintegration into a microbial host. Therefore, our leaf disk screening approach is not a replacement for total biosynthesis in a single host but rather a complementary strategy to select the most suitable oxidases from a larger pool (Figure 6).

**Figure 6:**
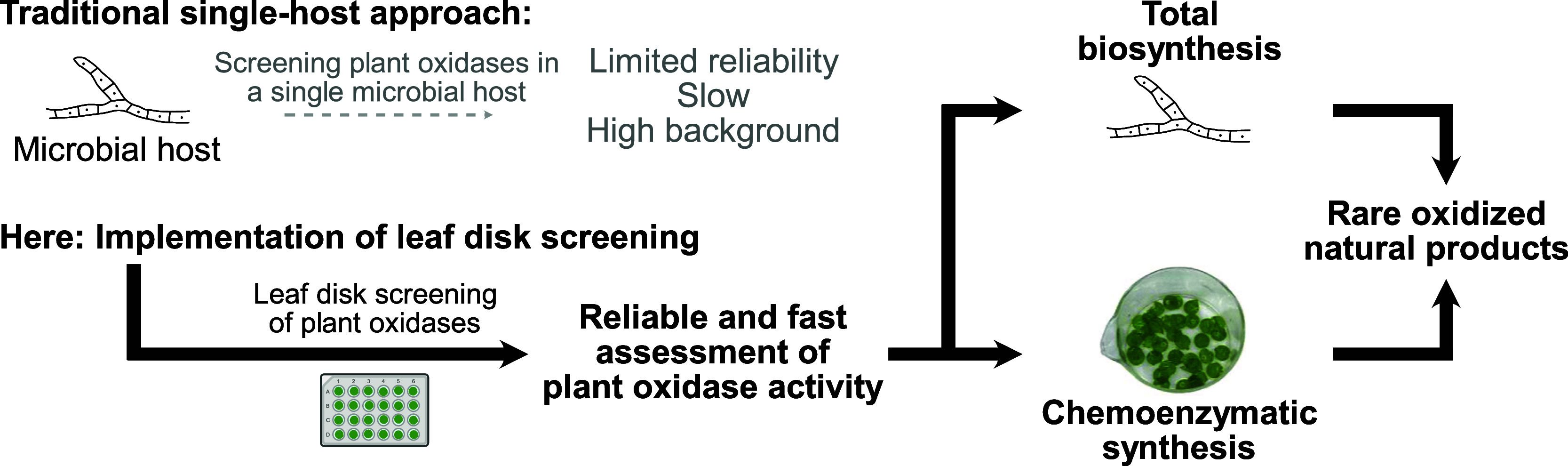
Strategic advantages of integrating the leaf disk screening platform presented here compared to traditional single-host strategies.

We successfully applied our leaf disk system for chemoenzymatic synthesis of seven diterpenoids including two rare gibberellins in quantities between 0.7 and 2.2 mg (Figure 4B). Taking into consideration that most gibberellins – except for a few major representatives – are exceedingly rare natural products, the yields obtained in our system are on par, and in many aspects superior, to established methods (Figure S21, Table S7). For example, microbial fermentation only has higher space-time-yields for common gibberellins but has not yet provided access to rare gibberellins (Figure S21A). Compared to isolation from plants, the specific yields from our leaf disk chemoenzymatic reactions are ca. 100–1,000,000 times higher (Figure S21B). Overall yields from total synthesis and semisynthesis can be comparable but are based on multiple labor- and resource-intensive steps rather than a single clean biotransformation (Figure S21C). The relevance of our system for accessing very rare gibberellins and diterpenoids is also underlined by the fact that five of the seven diterpenoids that we produced by chemoenzymatic synthesis have never been obtained in sufficient amounts for full NMR characterization before. Nonetheless, further improvements will be necessary to increase the efficiency and yield of this system in the future to make it more universal and also applicable to less rare natural products. One issue is the overall low turnover of substrates; for all tested reactions, at least 21–37% of the substrate were not consumed and could be reisolated. A biochemical reason might be that we used non-native substrates, which might be converted more slowly. An alternative explanation could be that excised leaf disks lose their viability over time, which could lead to issues in maintaining NADPH supply for CYP activity or to losses of active protein due to protein degradation. The low amounts of isolated products can likely also be attributed at least partially to losses during the final chromatographic workup. As our leaf disks contain plant background enzymes, unwanted shunt reactions from this *in vivo* system could also diminish product yields however, in our experiments, we did not observe substantial peaks that could be caused by undesired background reactions such as glycosylations. Another limitation that we noted during our screening campaign was the requirement for sufficient solubility of the substrates in the aqueous system. Small quantities of 2–3% of methanol or dimethyl sulfoxide as cosolvents were tolerated, but not higher concentrations. Nonetheless, despite these limitations, we propose that our leaf disk screening platform will help to make oxidases from plants and other eukaryotes more accessible for biocatalytic applications and thereby expands the enzyme space that can be employed for chemoenzymatic synthesis. This approach will become even more useful with growing knowledge regarding tailoring enzymes in diterpenoid biosynthesis in the future.^58^

So far, one of the most popular host organisms for the heterologous production of diterpenoids has been *Saccharomyces cerevisiae*.^16,42,43,59,60^ However, the titers of oxidized diterpenoids achieved in *S. cerevisiae* are often below 10 mg/L, typically caused by low activity of many plant CYPs in yeast. While plants should provide a better environment for CYP activity, high-level production of diterpene scaffolds in plants such as *N. benthamiana* is also still challenging,^61,62^ even though major improvements were achieved by transferring diterpenoid biosynthetic enzymes from plastids to the cytosol to benefit from the higher carbon flux via the mevalonate pathway.^63^ Although still relatively rare, several examples indicate that filamentous fungi such as *A. oryzae* can be superior alternative hosts for the production of diterpenoids.^26,64–68^ This is also supported by our data. In our work, usage of a truncated HMGR from plants^24^ helped to achieve high carbon flux through the mevalonate pathway. In addition, very good activity for the fungal CYPs P450-1 and P450-2 as well as the plant CYP IrCYP706V7 was observed, whereas many other host organisms struggle with efficient electron transfer for fueling CYPs or the correct processing of membrane-bound enzymes.^69^ Never-the-less, we observed several shunt products that suggest that there is still large untapped potential for further host improvement; we anticipate that identifying and inactivating the enzymes involved in shunt product formation, for example by targeted gene deletions, could provide further drastic improvements to diterpenoid yields in *A. oryzae*. Nonetheless, our *A. oryzae* strains surpass the titers of GA_3_, GA_4_, and GA_7_ previously achieved in yeast without the requirement for extensive strain engineering.^16^ More importantly for the purpose of this work, it enabled access to multiple rare gibberellins which are not commercially available. We believe that this improved access to rare gibberellins will fuel further studies to gain a better understanding of their biological relevance and assess their potential for agricultural applications.^70^

## Conclusion

In this work, we produced eight rare gibberellins as well as 16 oxidized *ent*-kaurene derivatives using a combination of chemoenzymatic synthesis with plant oxidases and metabolic engineering in the filamentous fungus *A. oryzae*. These one- or two-step processes dramatically out-compete total chemical synthesis for the production of this class of metabolites.^71^ By improving the accessibility of rare gibberellins, it is now possible to further investigate the biological roles of these complex diterpenoids in plant hormone signaling and search for new applications in agriculture. More generally, our work demonstrates how oxidases from plants and not just from microbes can be used for enzyme screening campaigns and chemoenzymatic synthesis. This will therefore help to harness the diversity of plant oxidases for biocatalysis in the future.

## Supplementary Material







## Notes

Table of leaf disk screening results for each tested enzyme–substrate pair (XLSX)
Table of all 65 metabolites identified during leaf disk screening (XLSX)
Experimental details, compound characterization details, chromatograms, spectra, and sequences. NMR characterization and spectra of isolated compounds (PDF)

## Abbreviations

CYP: cytochrome P450 monooxygenase
GA: gibberellic acid
NMR: nuclear magnetic resonance
NOESY: nuclear overhauser effect spectroscopy
ODD: oxoglutarate-dependent dioxygenase

## References

[1] Hedden P. (2020). The Current
Status of Research on Gibberellin Biosynthesis. Plant Cell Physiol..

[2] Camara M. C., Vandenberghe L. P. S., Rodrigues C., de Oliveira J., Faulds C., Bertrand E., Soccol C. R. (2018). Current Advances
in Gibberellic Acid (GA_3_) Production, Patented Technologies
and Potential Applications. Planta.

[3] Rademacher, W. Chemical Regulators of Gibberellin Status and Their Application in Plant Production. In Annual Plant Reviews; John Wiley & Sons, Ltd, 2016; Vol. 49, pp 359–404.

[4] Ishida T., Zhang Y., Zhu H., Fudano S., Peng Y., Seto Y., Mashiguchi K., Liu J., He Z., Zhang S., Yamaguchi S. (2025). Stepwise Deactivation
of Gibberellins
during Rice Internode Elongation. Proc. Natl.
Acad. Sci. U. S. A..

[5] Blake P. S., Taylor D. R., Crisp C. M., Mander L. N., Owen D. J. (2000). Identification
of Endogenous Gibberellins in Strawberry, Including the Novel Gibberellins
GA_123_, GA_124_ and GA_125_. Phytochemistry.

[6] Yuda E., Nakagawa S., Murofushi N., Yokota T., Takahashi N., Koshioka M., Murakami Y., Pearce D., Pharis R. P., Patrick G. L., Mander L. N., Kraft-Klaunzer P. (1992). Endogenous
Gibberellins in the Immature Seed and Pericarp of Loquat (*Eriobotrya Japonica*). Biosci., Biotechnol.,
Biochem..

[7] Yokota T., Takahashi N. (1981). Gibberellin
A_59_: A New Gibberellin from *Canavalia gladiata*. Agric. Biol. Chem..

[8] Binenbaum J., Weinstain R., Shani E. (2018). Gibberellin Localization and Transport
in Plants. Trends Plant Sci..

[9] Regnault T., Davière J.-M., Wild M., Sakvarelidze-Achard L., Heintz D., Carrera
Bergua E., Lopez Diaz I., Gong F., Hedden P., Achard P. (2015). The Gibberellin Precursor
GA_12_ Acts as a Long-Distance Growth Signal in Arabidopsis. Nat. Plants.

[10] Li L., Liang W., Rivera M. E., Wang Y.-C., Dai M. (2023). Concise Synthesis
of (−)-GA_18_ Methyl Ester. J. Am. Chem. Soc..

[11] Cossey A. L., Lombardo L., Mander L. N. (1980). Total Synthesis of Gibberellin A_4_. Tetrahedron Lett..

[12] Tudzynski B. (2005). Gibberellin
Biosynthesis in Fungi: Genes, Enzymes, Evolution, and Impact on Biotechnology. Appl. Microbiol. Biotechnol..

[13] Morrone D., Chambers J., Lowry L., Kim G., Anterola A., Bender K., Peters R. J. (2009). Gibberellin Biosynthesis
in Bacteria:
Separate *ent*-Copalyl Diphosphate and *ent*-Kaurene Synthases in *Bradyrhizobium japonicum*. FEBS Lett..

[14] Hedden P., Sponsel V. (2015). A Century of Gibberellin Research. J. Plant Growth Regul..

[15] Lin Y., Liang M., Pang H., Wang Z., Bi H., Wei Y., Du L. (2024). Production of Gibberellins via a Non-Natural Pathway
Using Steviol as a Substrate. J. Agric. Food
Chem..

[16] Kildegaard K. R., Arnesen J. A., Adiego-Pérez B., Rago D., Kristensen M., Klitgaard A. K., Hansen E. H., Hansen J., Borodina I. (2021). Tailored Biosynthesis
of Gibberellin Plant Hormones in Yeast. Metab.
Eng..

[17] Sugai Y., Miyazaki S., Mukai S., Yumoto I., Natsume M., Kawaide H. (2011). Enzymatic Total Synthesis
of Gibberellin A_4_ from Acetate. Biosci.,
Biotechnol., Biochem..

[18] Jin F. J., Maruyama J., Juvvadi P. R., Arioka M., Kitamoto K. (2004). Development
of a Novel Quadruple Auxotrophic Host Transformation System by *argB* Gene Disruption Using *adeA* Gene and
Exploiting Adenine Auxotrophy in *Aspergillus oryzae*. FEMS Microbiol. Lett..

[19] Fujita T., Takao S., Fujita E. (1973). Biosynthesis
of the Diterpenes Enmein
and Oridonin from *ent*-16-Kaurene. J. Chem. Soc., Chem. Commun..

[20] Sun Y., Shao J., Liu H., Wang H., Wang G., Li J., Mao Y., Chen Z., Ma K., Xu L., Wang Y. (2023). A Chromosome-Level Genome Assembly Reveals That Tandem-Duplicated
CYP706V Oxidase Genes Control Oridonin Biosynthesis in the Shoot Apex
of *Isodon rubescens*. Mol. Plant.

[21] Smanski M. J., Yu Z., Casper J., Lin S., Peterson R. M., Chen Y., Wendt-Pienkowski E., Rajski S. R., Shen B. (2011). Dedicated *ent*-Kaurene
and *ent*-Atiserene Synthases for Platensimycin
and Platencin Biosynthesis. Proc. Natl. Acad.
Sci. U. S. A..

[22] Toyomasu T., Kawaide H., Ishizaki A., Shinoda S., Otsuka M., Mitsuhashi W., Sassa T. (2000). Cloning of a Full-Length cDNA Encoding *ent*-Kaurene Synthase from *Gibberella fujikuroi*: Functional Analysis of a Bifunctional Diterpene Cyclase. Biosci., Biotechnol., Biochem..

[23] Pahirulzaman, K. A. K. ; Williams, K. ; Lazarus, C. M. Chapter Twelve - A Toolkit for Heterologous Expression of Metabolic Pathways in *Aspergillus oryzae* . In Methods in Enzymology; Natural Product Biosynthesis by Microorganisms and Plants, Part C; Hopwood, D. A. , Ed.; Academic Press, 2012; Vol. 517, pp 241–260. 10.1016/B978-0-12-404634-4.00012-7 23084942

[24] Reed J., Stephenson M. J., Miettinen K., Brouwer B., Leveau A., Brett P., Goss R. J. M., Goossens A., O’Connell M. A., Osbourn A. (2017). A Translational Synthetic Biology Platform for Rapid
Access to Gram-Scale Quantities of Novel Drug-like Molecules. Metab. Eng..

[25] Tagami K., Liu C., Minami A., Noike M., Isaka T., Fueki S., Shichijo Y., Toshima H., Gomi K., Dairi T., Oikawa H. (2013). Reconstitution of Biosynthetic
Machinery for Indole-Diterpene
Paxilline in *Aspergillus oryzae*. J. Am. Chem. Soc..

[26] Bailey A. M., Alberti F., Kilaru S., Collins C. M., de Mattos-Shipley K., Hartley A. J., Hayes P., Griffin A., Lazarus C. M., Cox R. J., Willis C. L., O’Dwyer K., Spence D. W., Foster G. D. (2016). Identification and Manipulation of
the Pleuromutilin Gene Cluster from *Clitopilus passeckerianus* for Increased Rapid Antibiotic Production. Sci. Rep..

[27] Helliwell C. A., Chandler P. M., Poole A., Dennis E. S., Peacock W. J. (2001). The CYP88A
Cytochrome P450, *ent*-Kaurenoic Acid Oxidase, Catalyzes
Three Steps of the Gibberellin Biosynthesis Pathway. Proc. Natl. Acad. Sci. U. S. A..

[28] Rojas M. C., Hedden P., Gaskin P., Tudzynski B. (2001). The P450–1
Gene of *Gibberella fujikuroi* Encodes a Multifunctional
Enzyme in Gibberellin Biosynthesis. Proc. Natl.
Acad. Sci. U. S. A..

[29] Ward J. L., Gaskin P., Brown R. G. S., Jackson G. S., Hedden P., Phillips A. L., Willis C. L., Beale M. H. (2002). Probing the Mechanism
of Loss of Carbon-20 in Gibberellin Biosynthesis. Synthesis of Gibberellin
3α,20-Hemiacetal and 19,20-Lactol Analogues and Their Metabolism
by a Recombinant GA 20-Oxidase. J. Chem. Soc.,
Perkin Trans. 1.

[30] Murofushi N., Sugimoto M., Itoh K., Takahashi N. (1979). Three Novel
Gibberellins Produced by *Gibberella fujikuroi*. Agric. Biol. Chem..

[31] Bathe U., Tissier A. (2019). Cytochrome P450 Enzymes:
A Driving Force of Plant Diterpene
Diversity. Phytochemistry.

[32] Helfrich E. J. N., Lin G.-M., Voigt C. A., Clardy J. (2019). Bacterial Terpene Biosynthesis:
Challenges and Opportunities for Pathway Engineering. Beilstein J. Org. Chem..

[33] Poborsky M., Crocoll C., Motawie M. S., Halkier B. A. (2023). Systematic Engineering
Pinpoints a Versatile Strategy for the Expression of Functional Cytochrome
P450 Enzymes in *Escherichia coli* Cell Factories. Microb. Cell Factories.

[34] Hausjell J., Halbwirth H., Spadiut O. (2018). Recombinant Production of Eukaryotic
Cytochrome P450s in Microbial Cell Factories. Biosci. Rep..

[35] Schoch G. A., Attias R., Belghazi M., Dansette P. M., Werck-Reichhart D. (2003). Engineering
of a Water-Soluble Plant Cytochrome P450, CYP73A1, and NMR-Based Orientation
of Natural and Alternate Substrates in the Active Site. Plant Physiol..

[36] Chuang, L. ; Franke, J. Rapid Combinatorial Coexpression of Biosynthetic Genes by Transient Expression in the Plant Host *Nicotiana benthamiana* . In Engineering Natural Product Biosynthesis: Methods and Protocols; Skellam, E. , Ed.; Methods of Molecular Biology; Springer US: New York, NY, 2022; pp 395–420. 10.1007/978-1-0716-2273-5_20 35524061

[37] Watanabe D., Takahashi I., Jaroensanti-Tanaka N., Miyazaki S., Jiang K., Nakayasu M., Wada M., Asami T., Mizutani M., Okada K., Nakajima M. (2021). The Apple Gene Responsible for Columnar
Tree Shape Reduces the Abundance of Biologically Active Gibberellin. Plant J..

[38] Phillips A. L., Ward D. A., Uknes S., Appleford N. E. J., Lange T., Huttly A. K., Gaskin P., Graebe J. E., Hedden P. (1995). Isolation and Expression of Three Gibberellin 20-Oxidase
cDNA Clones from Arabidopsis. Plant Physiol..

[39] Xiong W., Ye T., Yao X., Liu X., Ma S., Chen X., Chen M.-L., Feng Y.-Q., Wu Y. (2018). The Dioxygenase GIM2
Functions in Seed Germination by Altering Gibberellin Production in *Arabidopsis*. J. Integr. Plant Biol..

[40] Liu H., Guo S., Lu M., Zhang Y., Li J., Wang W., Wang P., Zhang J., Hu Z., Li L., Si L., Zhang J., Qi Q., Jiang X., Botella J. R., Wang H., Song C.-P. (2019). Biosynthesis of DHGA12 and Its Roles
in *Arabidopsis* Seedling Establishment. Nat. Commun..

[41] Mitchum M. G., Yamaguchi S., Hanada A., Kuwahara A., Yoshioka Y., Kato T., Tabata S., Kamiya Y., Sun T. (2006). Distinct and
Overlapping Roles of Two Gibberellin 3-Oxidases in Arabidopsis Development. Plant J..

[42] Scheler U., Brandt W., Porzel A., Rothe K., Manzano D., Božić D., Papaefthimiou D., Balcke G. U., Henning A., Lohse S., Marillonnet S., Kanellis A. K., Ferrer A., Tissier A. (2016). Elucidation
of the Biosynthesis of Carnosic Acid and
Its Reconstitution in Yeast. Nat. Commun..

[43] Frey M., Bathe U., Meink L., Balcke G. U., Schmidt J., Frolov A., Soboleva A., Hassanin A., Davari M. D., Frank O., Schlagbauer V., Dawid C., Tissier A. (2024). Combinatorial
Biosynthesis in Yeast Leads to over 200 Diterpenoids. Metab. Eng..

[44] He J., Chen Q., Xin P., Yuan J., Ma Y., Wang X., Xu M., Chu J., Peters R. J., Wang G. (2019). CYP72A Enzymes Catalyse 13-Hydrolyzation
of Gibberellins. Nat. Plants.

[45] Nomura T., Magome H., Hanada A., Takeda-Kamiya N., Mander L. N., Kamiya Y., Yamaguchi S. (2013). Functional
Analysis of *Arabidopsis* CYP714A1 and CYP714A2 Reveals
That They Are Distinct Gibberellin Modification Enzymes. Plant Cell Physiol..

[46] Blechschmidt S., Castel U., Gaskin P., Hedden P., Graebe J. E., MacMillan J. (1984). GC/MS Analysis of the Plant Hormones in Seeds of *Cucurbita maxima*. Phytochemistry.

[47] Lange T., Hedden P., Graebe J. E. (1993). Gibberellin
Biosynthesis in Cell-Free
Extracts from Developing *Cucurbita maxima* Embryos
and the Identification of New Endogenous Gibberellins. Planta.

[48] Murofushi N., Nakayama M., Takahashi N., Gaskin P., MacMillan J. (1988). 12-Hydroxylation
of Gibberellins A_12_ and A_14_ by Prothallia of *Lygodium japonicum* and Identification of a New Gibberellin.
GA_74_. Agric. Biol. Chem..

[49] Urban P., Mignotte C., Kazmaier M., Delorme F., Pompon D. C. (1997). Yeast Expression,
and Characterization of the Coupling of Two Distantly Related *Arabidopsis thaliana* NADPH-Cytochrome P450 Reductases with
P450 CYP73A5. J. Biol. Chem..

[50] Hansen C. C., Nelson D. R., Møller B. L., Werck-Reichhart D. (2021). Plant Cytochrome
P450 Plasticity and Evolution. Mol. Plant.

[51] Werck-Reichhart D., Feyereisen R. (2000). Cytochromes
P450: A Success Story. Genome Biol..

[52] Liu X., Xu Y., Li L., Li J. (2024). Chemoenzymatic Oxidation of Labdane
and Formal Synthesis of Nimbolide. J. Am. Chem.
Soc..

[53] Lin G.-M., Voigt C. A. (2023). Design
of a Redox-Proficient *Escherichia coli* for Screening
Terpenoids and Modifying Cytochrome P450s. Nat.
Catal..

[54] Zetzsche L. E., Yazarians J. A., Chakrabarty S., Hinze M. E., Murray L. A. M., Lukowski A. L., Joyce L. A., Narayan A. R. H. (2022). Biocatalytic
Oxidative Cross-Coupling Reactions for Biaryl Bond Formation. Nature.

[55] Zhang X., King-Smith E., Dong L.-B., Yang L.-C., Rudolf J. D., Shen B., Renata H. (2020). Divergent Synthesis
of Complex Diterpenes
through a Hybrid Oxidative Approach. Science.

[56] Romero E. O., Saucedo A. T., Hernández-Meléndez J. R., Yang D., Chakrabarty S., Narayan A. R. H. (2023). Enabling Broader
Adoption of Biocatalysis in Organic Chemistry. JACS Au.

[57] Božić D., Papaefthimiou D., Brückner K., de Vos R. C. H., Tsoleridis C. A., Katsarou D., Papanikolaou A., Pateraki I., Chatzopoulou F. M., Dimitriadou E., Kostas S., Manzano D., Scheler U., Ferrer A., Tissier A., Makris A. M., Kampranis S. C., Kanellis A. K. (2015). Towards Elucidating Carnosic Acid Biosynthesis in Lamiaceae:
Functional Characterization of the Three First Steps of the Pathway
in *Salvia fruticosa* and *Rosmarinus officinalis*. PLoS One.

[58] Rutz A., Probst D., Aguilar C., Akiyama D. Y., Alberti F., Augustijn H. E., Avalon N. E., Beemelmanns C., Bertoletti Barbieri H., Biermann F., Bridge A. J., Charria
Girón E., Cox R., Crüsemann M., D’Agostino P. M., Feuermann M., Gerke J., Gutiérrez
García K., Holme J., Hwang J.-Y., Iacovelli R., Jeronimo Barbosa J. C., Kaur N., Klapper M., Köhler A. M., Korenskaia A., Kubach N., Lee B. T., Loureiro C., Mantri S., Narula S., Meijer D., Navarro-Muñoz J. C., Nguyen G.-S., Paliyal S., Panghal M., Rao L., Sieber S., Sokolova N., Sowa S. T., Szenei J., Terlouw B., Weddeling H. G., Yu J., Ziemert N., Weber T., Blin K., van der Hooft J. J. J., Medema M. H., Zdouc M. M. (2025). MITE: The Minimum Information about
a Tailoring Enzyme Database for Capturing Specialized Metabolite Biosynthesis. ChemRxiv.

[59] Wong J., de Rond T., d’Espaux L., van der Horst C., Dev I., Rios-Solis L., Kirby J., Scheller H., Keasling J. (2018). High-Titer
Production of Lathyrane Diterpenoids from Sugar by Engineered *Saccharomyces cerevisiae*. Metab. Eng..

[60] Ignea C., Athanasakoglou A., Ioannou E., Georgantea P., Trikka F. A., Loupassaki S., Roussis V., Makris A. M., Kampranis S. C. (2016). Carnosic
Acid Biosynthesis Elucidated by a Synthetic
Biology Platform. Proc. Natl. Acad. Sci. U.
S. A..

[61] Forestier E. C. F., Czechowski T., Cording A. C., Gilday A. D., King A. J., Brown G. D., Graham I. A. (2021). Developing a *Nicotiana benthamiana* Transgenic Platform for High-Value Diterpene Production and Candidate
Gene Evaluation. Plant Biotechnol. J..

[62] Forestier E. C. F., Brown G. D., Harvey D., Larson T. R., Graham I. A. (2021). Engineering
Production of a Novel Diterpene Synthase Precursor in *Nicotiana
benthamiana*. Front. Plant Sci..

[63] De
La Peña R., Sattely E. S. (2021). Rerouting Plant Terpene Biosynthesis
Enables Momilactone Pathway Elucidation. Nat.
Chem. Biol..

[64] Bromann K., Toivari M., Viljanen K., Ruohonen L., Nakari-Setälä T. (2016). Engineering *Aspergillus nidulans* for Heterologous *ent*-Kaurene and Gamma-Terpinene Production. Appl.
Microbiol. Biotechnol..

[65] Fujii R., Minami A., Tsukagoshi T., Sato N., Sahara T., Ohgiya S., Gomi K., Oikawa H. (2011). Total Biosynthesis
of Diterpene Aphidicolin, a Specific Inhibitor of DNA Polymerase α:
Heterologous Expression of Four Biosynthetic Genes in *Aspergillus
oryzae*. Biosci., Biotechnol., Biochem..

[66] Guan Z., Yao N., Yuan W., Li F., Xiao Y., Rehmutulla M., Xie Y., Chen C., Zhu H., Zhou Y., Tong Q., Xiang Z., Ye Y., Zhang Y. (2025). Total Biosynthesis
of Cotylenin Diterpene Glycosides as 14-3-3 Protein–Protein
Interaction Stabilizers. Chem. Sci..

[67] Liu C., Tagami K., Minami A., Matsumoto T., Frisvad J. C., Suzuki H., Ishikawa J., Gomi K., Oikawa H. (2015). Reconstitution of Biosynthetic Machinery
for the Synthesis
of the Highly Elaborated Indole Diterpene Penitrem. Angew. Chem., Int. Ed..

[68] Liu C., Minami A., Ozaki T., Wu J., Kawagishi H., Maruyama J., Oikawa H. (2019). Efficient Reconstitution of Basidiomycota
Diterpene Erinacine Gene Cluster in Ascomycota Host *Aspergillus
oryzae* Based on Genomic DNA Sequences. J. Am. Chem. Soc..

[69] Renault H., Bassard J.-E., Hamberger B., Werck-Reichhart D. (2014). Cytochrome
P450-Mediated Metabolic Engineering: Current Progress and Future Challenges. Curr. Opin. Plant Biol..

[70] Robil J. M., Awale P., McSteen P., Best N. B. (2025). Gibberellins:
Extending
the Green Revolution. J. Exp. Bot..

[71] Tian D.-S., Zhang X., Cox R. J. (2025). Comparing
Total Chemical Synthesis
and Total Biosynthesis Routes to Fungal Specialized Metabolites. Nat. Prod. Rep..

