# Supplementary material for: Chemoenzymatic Synthesis with Plant Oxidases and Metabolic Engineering Enable Rapid Access to Rare Gibberellins
Source: J Am Chem Soc. 2026 Apr 29;148(18):19436–46. doi: 10.1021/jacs.6c06067 (PMC13185120; doi:10.1021/jacs.6c06067)
Supplement: Supplementary file 3 [file ja6c06067_si_003.pdf]

Supporting Information for

Chemoenzymatic Synthesis with Plant Oxidases and Metabolic Engineering Enable Rapid Access to Rare Gibberellins

Ahmed Arafa[a][b][c][d], Jennifer Gerke[a][c], Russell Cox[a][c]* and Jakob Franke[a][b]*

[a] Centre of Biomolecular Drug Research, Leibniz University Hannover, Schneiderberg 38, 30167 Hannover, Germany

[b] Institute of Botany, Leibniz University Hannover, Herrenhäuser Str. 2, 30419 Hannover, Germany

[c] Institute of Organic Chemistry, Leibniz University Hannover, Schneiderberg 1B, 30167 Hannover, Germany

[d] Pharmacognosy Department, Faculty of Pharmacy, Tanta University, 31527 Tanta, Egypt

* To whom correspondence should be addressed: russell.cox@oci.uni-hannover.de or jakob.franke@botanik.uni-hannover.de

Table of Contents

Table of Contents 2
Experimental Methods 4
Molecular biology methods 4
Gene source 4
Aspergillus oryzae transformation 4
Substrate feeding to fungal culture 4
Transient expression in *Nicotiana benthamiana* 4
Leaf Disk Assays 5
Analytical leaf disk assays 5
Preparative leaf disk assays 5
Metabolite Extraction 5
Fungal culture extraction 5
Extraction of analytical *Nicotiana benthamiana* leaf disk assays 6
Preparative-scale leaf disk extraction 6
General chemical methods 6
LC-MS and flash chromatography 6
GC-MS analysis 6
HRMS analysis 6
NMR analysis 6
Reference compounds 7
Sample Preparation 7
LC-MS samples 7
GC-MS sample derivatization 7
NMR sample preparation 7
Chemical hydrolysis of stevioside 7
Supporting Figures 8
Supporting Tables 29
NMR data and spectra 44
Gibberellin A_3 (Gibberellic acid) (1) 44
Gibberellin A_4 (2) 48
Gibberellin A_1 (4) 54
Gibberellin A_{12} (5) 57
Gibberellin A_{14} (6) 61
Gibberellin A_9 methyl ester (7a) 68
Gibberellin A_{13} (8) 72
Gibberellin A_{36} dimethyl ester (9a) 80
Gibberellin A_{123} (10) 84
Gibberellin A_{123} dimethyl ester (10a) 88
Gibberellin A_{74} (11) 92
Gibberellin A_{74} dimethyl ester (11a) 97
15β-Hydroxy-Gibberellin A_{12} dimethyl ester (12a) 101
ent-Labd-8(17)-en-15,18-dioic acid (17) 106
13,18-dihydroxy-*ent*-labd-8(17)-en-15-oic acid (18) 110
16α,17-Dihydroxy-*ent*-kauranoic acid (20) 114
16β,17-Dihydroxy-ent-kauranoic acid (21) 118
7β-Hydroxy-*ent*-kaurenoic acid (22) 122
6β,7β-Dihydroxy-*ent*-kaurenoic acid (23) 129
Fujenoic acid (24) 136
Fujenoic acid dimethyl ester (24a) 144
Fujenoic triacid trimethyl ester (25a) 148
7β,16α,17-Trihydroxy-*ent*-kauranoic acid (26) 152
7β,16α,17-Trihydroxy-*ent*-kauranoic acid methyl ester (26a) 156
6β,7β,16α-Trihydroxy-*ent*-kauranoic acid (27) 160
1,10-Didehydro-20-nor-GA_{14} dimethyl ester (28a) 167
Steviol (29) 171
Isosteviol (30) 174
7β,17-Dihydroxy-16-*epi*-*ent*-kauranoic acid (31) 178
7β,17-Dihydroxy-16-*epi*-*ent*-kauranoic acid methyl ester (31a) 182
7β-Hydroxy-16-*epi*-*ent*-kauran-17,19-dioic acid (32) 186
7β-Hydroxy-16-*epi*-*ent*-kauran-17,19-dioic acid dimethyl ester (32a) 190
6β,7β,17-Trihydroxy-16-*epi*-*ent*-kauranoic acid (33) 194
6β,7β,13-Trihydroxy-*ent*-kaurenoic acid (34) 198
15β-Hydroxy-*ent*-kaurenoic acid (35) 202
7β,15β-Dihydroxy-*ent*-kaurenoic acid (36) 206
7β,15β-Dihydroxy-*ent*-kaurenolide (42) 210
1β,7β,15β-Trihydroxy-*ent*-kaurenoic acid (43) 214
3β,7β,15β-Trihydroxy-*ent*-kaurenoic acid (44) 218
6β,7β,15β-Trihydroxy-*ent*-kaurenoic acid methyl ester (45a) 222
Supporting References 226

Figures

Figure S1. Comparison of bacterial (PtmT2 + *Bj*KS) and fungal terpene cyclases (bifunctional CPS/KS) for the production of GA_4 (**2**) in *A. oryzae*. 8
Figure S2. Comparison of metabolite profiles of *A. oryzae* NSAR1 background strain and our full gibberellin platform strain containing *ggs2*, *cps/ks*, *AstHMGR*, *ko*, *P450-1*, *P450-2*, *des*, and *P450-3* genes. 9
Figure S3. Example set-up for leaf disk screening on analytical scale of six oxidases with 19 of the 20 substrates and a no-substrate control with three biological replicates. 10
Figure S4. Example chromatograms from leaf disk screening. 11
Figure S5. Products from leaf disk screening with *ent*-kaurene/kaurane skeletons identified by GC-MS fragmentation comparison relative to published data. 12
Figure S6. Putative products from leaf disk screening with *ent*-kaurane skeletons proposed based on their GC-MS fragmentation patterns.. 13
Figure S7. Products from leaf disk screening with gibberellin structures based on GC-MS fragmentation comparison relative to published data. 14
Figure S8. Putative products from leaf disk screening with gibberellin structures proposed based on their GC-MS fragmentation patterns.... 15
Figure S9. Leaf disk screening results presented as bar plots of product profiles. 16
Figure S10. Leaf disk screening results presented as a network graph indicating the preferred substrates and conversion of tested enzymes. 17
Figure S11. Chromatograms from preparative leaf disk assays in comparison to analytical scale assays. 18
Figure S12. Tentative production of GA_{18} by chemoenzymatic conversion of GA_{14} (**6**) with AtCYP714A2. 19
Figure S13. Formation of 15β-hydroxy-*ent*-kaurenoic acid (**35**) in *A. oryzae* transformants containing *IrCYP706V7* and the cytochrome P450 reductase gene *AtCPR1* in addition to the upstream pathway to *ent*-kaurenoic acid (**19**) (*tHMGR+ggs2+cps/ks+ko*). 20
Figure S14. GC-MS fragmentation pattern of 15β-hydroxylated gibberellins produced in *Aspergillus oryzae* with IrCYP706V7 (our data in black against published data in red where available). 21
Figure S15. ^{1}H NMR spectrum of GA_{65} dimethyl ester (**41a**) showing an aldehyde proton at 9.64 ppm. 22
Figure S16. Confirmation that IrCYP706V6 is active in *N. benthamiana* despite its lack of activity with the panel of 20 tested substrates. 23
Figure S17. Enzymatic activity of AtCYP714A2 in *A. oryzae* is substantially lower than in *N. benthamiana*. 24
Figure S18. Lack of MdDOX-Co activity in *A. oryzae*. 25
Figure S19. Comparison of the time requirements for leaf disk screening in *N. benthamiana* with a potential analogous process based on episomal vectors in the fungal host *A. oryzae*. 26
Figure S20. Leaf disk biotransformations offer a much cleaner background compared to single host fermentation in *A. oryzae*. 27
Figure S21. Comparison of yields achieved in this work with reported methods for production of gibberellins in the literature. 28

Tables

Table S1. List of compounds fully characterized by NMR spectroscopy and HRMS in this work. 29
Table S2. Summary of constructed vectors and main products in *A. oryzae* NSAR1. 32
Table S3. List of ten previously reported diterpenoid oxidases screened in this work. 33
Table S4. Summary of metabolites produced during leaf disk screening of the ten plant oxidases with the 20 substrates. 34
Table S5. List of GC-MS electron impact fragmentation patterns compared to literature where available. 35
Table S6. Identification of GA_{18} based on ^{1}H NMR comparison of isolated GA_{18} after methylation with literature data. 38
Table S7. Comparison of amounts of gibberellins produced in this work and in previous studies. 39
Table S8. List of primers used in this study. 40
Table S9. List of gene sequences used in this study. 41

Experimental Methods

Molecular biology methods

Gene source

All primers used for cloning are listed in **Table S8**. All genes used in this study, except *AstHMGR, AtCPR1*, *AtGA3ox1*, and *AtGA20ox1*, were obtained as synthetic genes from Twist Bioscience (Berlin, Germany) or GeneWiz (Azenta Life Sciences, Leipzig, Germany) containing the overhangs required for the respective cloning methods described in **Table S9**. The genes *AtCPR1*, *AtGA3ox1*, and *AtGA20ox1* were amplified from *Arabidopsis thaliana* leaf cDNA. *AstHMGR* was obtained as reported previously.[1]

Aspergillus oryzae transformation

For *Aspergillus oryzae* transformation, the vectors pTYGS-*argB*, pTYGS-*adeA*, and pTYGS-*sC* were used.[2] Gene insertion into these vectors was performed by yeast homologous recombination using *Saccharomyces cerevisiae* CEN.PK2 cells (Euroscarf). Constructs were isolated from the yeast cells using Zymoprep Yeast Plasmid Miniprep II kit (Zymo Research). *Escherichia coli* One Shot ccdB Survival 2 $T1^R$ cells (Thermo Fisher Scientific) were used for plasmid amplification and storage. *A. oryzae* NSAR1 (Lazarus group, Bristol) was used as a heterologous host,[3,4] and fungal transformation was achieved using polyethylene glycol mediated protoplast transformation.[4,5] *A. oryzae* NSAR1 was grown on DPY agar plates[6] at 28 °C for 7-12 days. Mycelium from these plates was used to inoculate 50 mL GN medium[6] in a 250 mL shake flask, which was incubated at 28 °C and 110 rpm for approximately 18-20 h.

Biomass was separated from the culture medium by filtration through Miracloth (Millipore) and incubated with 10 mL of VinoTaste® Pro (Novozymes) solution (10 mg/mL; sterilized through a 0.45 µm filter, Roth) while gently shaking at room temperature and 5 rpm for 3–4 h. The suspension was pipetted gently to release protoplasts, which were collected by filtering through Miracloth and centrifugation (3000 × *g* for 5 min).

The resulting pellet was resuspended in 1 mL of transformation solution 1 (0.8 M NaCl, 10 mM $CaCl_2$, 50 mM Tris-HCl, pH 7.5). The DNA constructs (1-2 µg of each construct) were mixed and added to 100 µL of protoplast solution and incubated on ice for 2 min. Transformation solution 2 (60% [w/v] PEG 3350, 0.8 M NaCl, 10 mM $CaCl_2$, 50 mM Tris-HCl, pH 7.5; 1 mL) was then added, followed by incubation at room temperature for 20 min. The mixture was combined with 5 mL of selective soft agar and overlaid onto selective plates[6], which were incubated at 28 °C for 4–5 days. The following selective media were used: For transformations with pTYGS-*argB*, arginine was omitted from the medium; for co-transformations with pTYGS-*argB* and pTYGS-*adeA*, both arginine and adenine were omitted; for transformations involving pTYGS-*argB*, pTYGS-*adeA*, and pTYGS-*sC* (complete pathway reconstruction), arginine, adenine, and methionine were all omitted from the selective medium. Emerging colonies were subjected to two rounds of re-selection on fresh selective agar plates (2 days each) to confirm positive transformants. Verified transformants were maintained on DPY agar plates for 7 days, and spores were used to inoculate 100 mL DPY liquid medium[6] in a 500 mL shake flask for cultivation and metabolite production. The flasks were incubated at 28 °C and 110 rpm for 7 days.

Substrate feeding to fungal culture

A. oryzae strains were cultivated in shake flasks (100 mL culture per 500 mL flask) using DPY medium[6] under conditions as mentioned before, and a total of 1 mg of 15β-hydroxy-*ent*-kaurenoic acid (**35**) (dissolved in HEPES buffer, pH 9.2) was fed to every flask of the growing cultures in two equal portions on days 2 and 4. The feeding experiment was performed in triplicates. Extraction was done after 7 days from the initial inoculation.

Transient expression in *Nicotiana benthamiana*

For transient expression in *Nicotiana benthamiana*, the vectors pHREAC[7] and pEAQ-HT[8] were used. In-Fusion cloning (Takara Bio) was employed to insert *ggs2*, *cps/ks*, and *AtGA20ox1* into pEAQ-HT, whereas Golden Gate cloning was used to insert the remaining genes into pHREAC. *Escherichia coli* Stellar competent cells (Takara Bio) were used for verification, amplification, and storage of the constructs.

Transient expression was carried out in *N. benthamiana* LAB strain plants grown from seed in a phytochamber under a 16.5/7.5 h light/dark photoperiod (100 µmol m^{-2} s^{-1}) at 22 °C (day) and 20 °C (night). DNA constructs were introduced into *Agrobacterium tumefaciens* GV3101 by electroporation. *A. tumefaciens* cultures were grown in LB medium containing 25 µg/mL gentamicin,

50 µg/mL rifampicin, and 50 µg/mL kanamycin at 28 °C with shaking for 2 days. Cells were collected and resuspended in MMA infiltration buffer (10 mM $MgCl_2$, 10 mM MES, 100 µM acetosyringone, pH 5.6) then incubated for 4 h at room temperature before infiltration.

For both analytical-scale assays and preparative-scale experiments, *A. tumefaciens* strains carrying the desired constructs with OD_{600} of 0.1 were syringe-infiltrated into leaves of 4–5-week-old *N. benthamiana* plants. For the IrCYP706V6 activity confirmation experiment, *A. tumefaciens* strains carrying *AstHMGR*, *ggs2*, *cps/ks*, and *IrCYP706V6* were mixed to yield a final OD_{600} of 0.1 per construct.

Leaf Disk Assays

Analytical leaf disk assays

Analytical leaf disk assays were carried out similar to a report by Kamileen *et al.*[9] *N. benthamiana* leaves expressing the desired plant oxidase gene (as described above) were harvested 6 days post-infiltration. Leaf disks from three individual plants were excised as biological triplicates using a cork borer no. 9 to give leaf disks with 14 mm in diameter. The disks were gently scratched on the lower epidermal surface with sterile pipette tips or toothpicks to improve uptake of substrates and placed individually into wells of a 24-well plate with the upper epidermis facing upward.

The tested substrates (2 mg) were dissolved in 16.5 mL 50 mM HEPES buffer. The pH was adjusted according to substrate polarity; polar substrates were readily soluble in HEPES buffer at pH 7.5, whereas relatively nonpolar substrates required buffer at pH 9.2 supplemented with 2–3% methanol or dimethyl sulfoxide to achieve full solubility. From this substrate solution, 500 µL were added to each well containing a leaf disk.

Plates were carefully sealed with Parafilm and incubated in a phytochamber (with conditions as described above) for 40 h. Leaf disks infiltrated with an empty vector (EV) strain served as negative controls. Additionally, enzyme-producing leaf disks incubated with buffer-only solution were used as no-substrate controls to exclude any potential effects of enzyme activity on endogenous plant metabolites.

After incubation, leaf disks were separated from the solutions, transferred to 2 mL Eppendorf tubes, and lyophilized (Alpha 1-4 LDplus, Martin Christ) overnight before extraction.

Preparative leaf disk assays

For preparative scale assays, three individual *Nicotiana benthamiana* plants were infiltrated with *A. tumefaciens* carrying the desired gene construct, and leaves were harvested 6 days post-infiltration. Two leaves from each plant were scratched with sterile toothpicks and cut into disks (approximately 140 disks in total per reaction). The disks were placed in a crystallizing dish (VWR, 115 mm, borosilicate glass 3.3) containing the substrate dissolved in 50 mL of 50 mM HEPES buffer (pH 9.2); for GA_{14} (**6**), the buffer pH was adjusted to 7.5.

Each reaction was performed in a separate crystallizing dish, with its own set of leaf disks and substrate solution. Crystallizing dishes were sealed with Parafilm and incubated in the phytochamber for 3 days (with conditions as described above). Afterwards, crystallizing dishes were transferred to the laboratory, where a magnetic stir bar was added to promote gentle stirring and enhance substrate diffusion into the leaf tissue, ensuring optimal enzyme-substrate contact. Leaf disks were then gently stirred for 3 more days in the laboratory.

After incubation, the leaf disks from each reaction were separated from their respective solutions and lyophilized for 2 days. Both the lyophilized leaf material and the corresponding incubation solution were extracted individually to recover products and any remaining substrate.

Metabolite Extraction

Fungal culture extraction

For fungal extractions, the *A. oryzae* culture broth (medium and biomass) was homogenized using a blender and filtered under vacuum. The filtrate was acidified to pH 3–4 with hydrochloric acid (HCl) and extracted twice with ethyl acetate. The combined organic layers were dried over anhydrous sodium sulfate (Na_2SO_4), filtered, and evaporated to dryness under reduced pressure using a rotary evaporator, and the residue was dissolved in methanol.

Extraction of analytical *Nicotiana benthamiana* leaf disk assays

Lyophilized *N. benthamiana* leaf disks were ground in a ball mill (MM 400, Retsch, Haan, Germany) at 30 Hz for 20 s. Water acidified with 2 M HCl to pH 3-4 (0.7 mL) was added to the leaf powder, followed by vigorous mixing using a vortex mixer. The mixture was extracted twice with ethyl acetate (2 × 1 mL). The combined organic phases were evaporated to dryness using a SpeedVac concentrator (Martin Christ, Germany), and the residue was dissolved in 1 mL of methanol.

Preparative-scale leaf disk extraction

For preparative-scale extractions, lyophilized *N. benthamiana* leaf disks were carefully ground using a mortar and pestle. Acidified water (30 mL, pH 3–4) was added to the powder and transferred into two 50 mL centrifuge tubes (15 mL per tube). Ethyl acetate (20 mL per tube) was added, and the mixtures were vortexed and centrifuged at 3000 × *g* for 3 min. The extraction was repeated three times. After drying the combined organic layers over anhydrous Na_2SO_4 and filtration, the solvent was removed under reduced pressure using a rotary evaporator. The obtained residue was subsequently dissolved in methanol.

General chemical methods

LC-MS and flash chromatography

Analytical LC-MS analyses of fungal and plant extracts were conducted on a Waters system equipped with a 2767 autosampler, a 2545 pump, and a Phenomenex Kinetex C_{18} column (2.6 µm, 100 Å, 4.6 × 100 mm) fitted with a Phenomenex Security Guard precolumn (Luna C_5, 300 Å). The mobile phase was delivered at a flow rate of 1.0 mL/min using a 15 min linear gradient from 10% to 90% acetonitrile (0.045% formic acid) in water (0.05% formic acid). Detection was carried out using a Waters 2998 diode array detector (210-600 nm), a Waters 2424 evaporative light scattering detector (ELSD), and a Waters SQD-2 mass detector operating in both ES+ and ES– modes (*m*/*z* 100–1000).

Preparative LC-MS was performed on a Waters 2767 autosampler coupled to a Waters 2545 pump system (20 mL/min) and a Waters mass-directed auto-purification setup. Separation was achieved using a Phenomenex Kinetex Axia C_{18} column (5 µm, 100 Å, 21.2 × 250 mm) protected by a Phenomenex Security Guard precolumn (Luna C_5, 300 Å). The column effluent was split 100:1, with the major fraction collected and the minor flow (0.8 mL/min) directed to the same set of detectors described above. The solvent gradient was adjusted to achieve the optimal compounds separation. Fractions were collected based on mass detection, combined as appropriate, and concentrated using a rotary evaporator to remove residual acetonitrile. The remaining aqueous phase was frozen and lyophilized.

Flash chromatography was performed on a Biotage Isolera One system using a Sfär C18 D 30 g column. Fractions containing the desired compounds were collected and concentrated using a rotary evaporator and then lyophilized.

GC-MS analysis

Gas chromatography-mass spectrometry (GC-MS) analyses were carried out using Hewlett Packard HP6890N gas chromatograph coupled to a 5973N mass selective detector. Separations were achieved on an OPTIMA 5 MS capillary column (30 m × 0.25 mm i.d., 0.25 µm film thickness; Macherey-Nagel, Düren, Germany). Helium was used as the carrier gas at a flow rate of 1.5 mL/min. Samples (2.5 µL) were injected with a split ratio of 1:5. The oven temperature was programmed to start at 100 °C, followed by a ramp of 12 °C/min to 200 °C, and then increased to 320 °C with a second ramp of 6 °C/min. The total run time was 33 min, allowing optimal signal separation. Data acquisition and processing were performed using Agilent MSD ChemStation F.01.03.2357. Published data used for comparison of mass spectra in mirror plots (**Figure S14**, **Figure S16**) were digitalized using Mass Spec Calculator Professional 5.1.

HRMS analysis

High-resolution mass spectrometry data were obtained using a Waters QTof Premier spectrometer coupled to an Acquity UPLC system (Waters) equipped with a TUV detector. Electrospray ionization was acquired in negative mode.

NMR analysis

NMR spectra of isolated compounds were acquired using Bruker spectrometers operating at 400, 500, or 600 MHz for ^1H NMR and at 100, 126, or 151 MHz for ^{13}C NMR. Experiments were performed at 298 K using deuterated solvents ($CDCl_3$, C_5D_5N,

DMSO-d_6, and MeOD) as specified. Chemical shifts (δ) are reported in parts per million (ppm) relative to residual solvent signals ($CDCl_3$: δ_H = 7.26 ppm, δ_C = 77.16 ppm; C_5D_5N: δ_H = 8.74, 7.58, 7.22 ppm, δ_C = 150.35, 135.91, 123.87 ppm; DMSO-d_6: δ_H = 2.50 ppm, δ_C = 39.52 ppm; MeOD: δ_H = 3.31 ppm, δ_C = 49.00 ppm). Coupling constants (*J*) are reported in hertz (Hz). Data processing and analysis were carried out using TopSpin (version 4.1.3) or MestReNova (version 14.3.1).

Structures for nOe comparison were prepared using Spartan 20 (version 1.1.5 MacOS 26.01, Wavefunction, Inc, USA). Initial models were built, and then minimized using molecular mechanics. A conformer distribution was then calculated in each case using the Merck molecular force field (MMFF). Conformers within 40 kJ/mol were retained. The lowest energy conformer was selected and matched *vs* the observed nOe correlations.

Reference compounds

Reference compounds used in this study were obtained from the following suppliers: *ent*-Kaurenoic acid (**19**) from Toronto Research Chemicals (TRC, Toronto, Canada; distributed by LGC), gibberellin A_7 (GA_7) (**3**) from Fluorochem (Hadfield, UK), and stevioside from Sigma-Aldrich (Steinheim, Germany).

Sample Preparation

LC-MS samples

For analytical and preparative LC-MS, samples dissolved in methanol were used directly without further processing. For GA_4 (**2**), GA_7 (**3**), GA_1 (**4**), and GA_3 (**1**) quantification, steviol (**29**) was used as internal standard, while for quantification of 15β-hydroxy-*ent*-kaurenoic acid (**35**), GA_3 (**1**) was used as internal standard.

GC-MS sample derivatization

For GC-MS analysis, samples were derivatized prior to measurement. The methanolic sample (0.5 mL) was methylated by adding 30 µL of trimethylsilyl diazomethane (2 M solution in hexane) and left in a fume hood for 30 min. Residual reagent and solvent were evaporated to dryness using a SpeedVac concentrator. Derivatization was then carried out at 70 °C for 1 h using 100 µL of a 1:1 (*v/v*) mixture of pyridine and BSTFA + 1% TMCS. The resulting derivatized samples were directly injected into the GC-MS system.

NMR sample preparation

For NMR measurements, isolated compounds were generally dissolved directly in the appropriate deuterated solvent. In some cases, methylation was performed to improve spectral quality. For methylation, the compounds were dissolved in methanol (2 mL), followed by the addition of 100 µL of trimethylsilyl diazomethane. Samples were left in a fume hood for 2 h with gentle shaking and then evaporated to dryness. In certain cases, the residue was measured directly by NMR, whereas in others, the compound was re-purified by preparative LC-MS to obtain clean spectra.

Chemical hydrolysis of stevioside

Acidic and basic hydrolysis of stevioside was performed to produce isosteviol and steviol, respectively, following a procedure by Murillo *et al.*[10]

For steviol, stevioside (200 mg) was dissolved in water (15 mL) and sodium periodate ($NaIO_4$, 1.0 g) was added. The mixture was stirred overnight at room temperature, after which potassium hydroxide (KOH, 0.5 g) was added. The resulting suspension was heated in an oil bath at 110 °C for 1 h. Water (15 mL) was then added, and the reaction mixture was acidified with acetic acid (0.5 mL) before extraction with ethyl acetate (3 × 40 mL). The combined organic layers were dried over anhydrous Na_2SO_4, filtered, and evaporated under reduced pressure using a rotary evaporator. Steviol (4 mg) was purified by preparative LC-MS.

For isosteviol, stevioside (100 mg) was dissolved in 10 mL of 1 M HCl and incubated in a sealed glass vial at 80 °C for 4 h in a drying oven. After cooling to room temperature, the reaction mixture was neutralized with sodium bicarbonate and extracted with ethyl acetate (3 × 20 mL). The combined organic layers were dried over anhydrous Na_2SO_4, filtered, and evaporated under reduced pressure. The residue was purified by preparative LC-MS to yield isosteviol (6 mg).

Supporting Figures

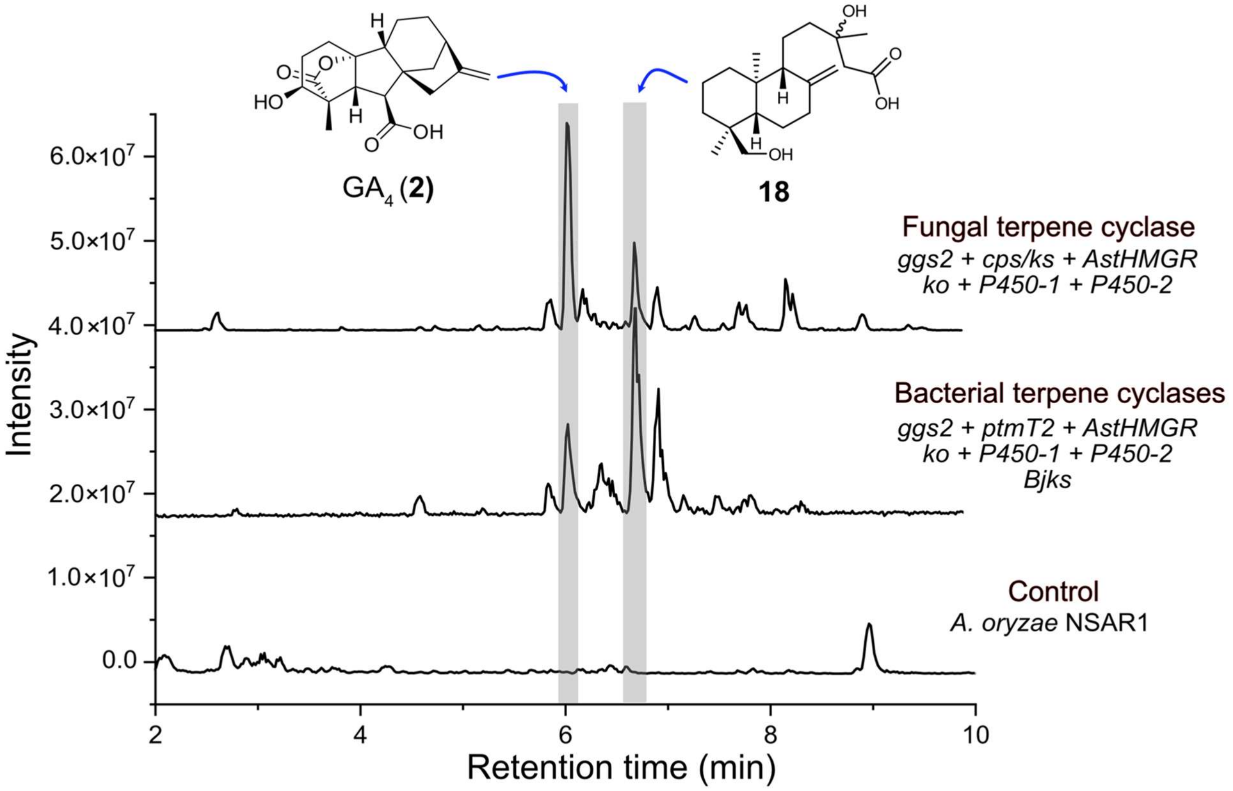

Figure S1. Comparison of bacterial (PtmT2 + *Bj*KS) and fungal terpene cyclases (bifunctional CPS/KS) for the production of GA_4 (**2**) in *A. oryzae*.

Data shown are base peak chromatograms (negative mode). The bacterial terpene cyclases produced more shunt products such as **18** and less of the desired fully cyclized diterpenoids compared to the fungal terpene cyclase. The background strain NSAR1 is shown as a control.

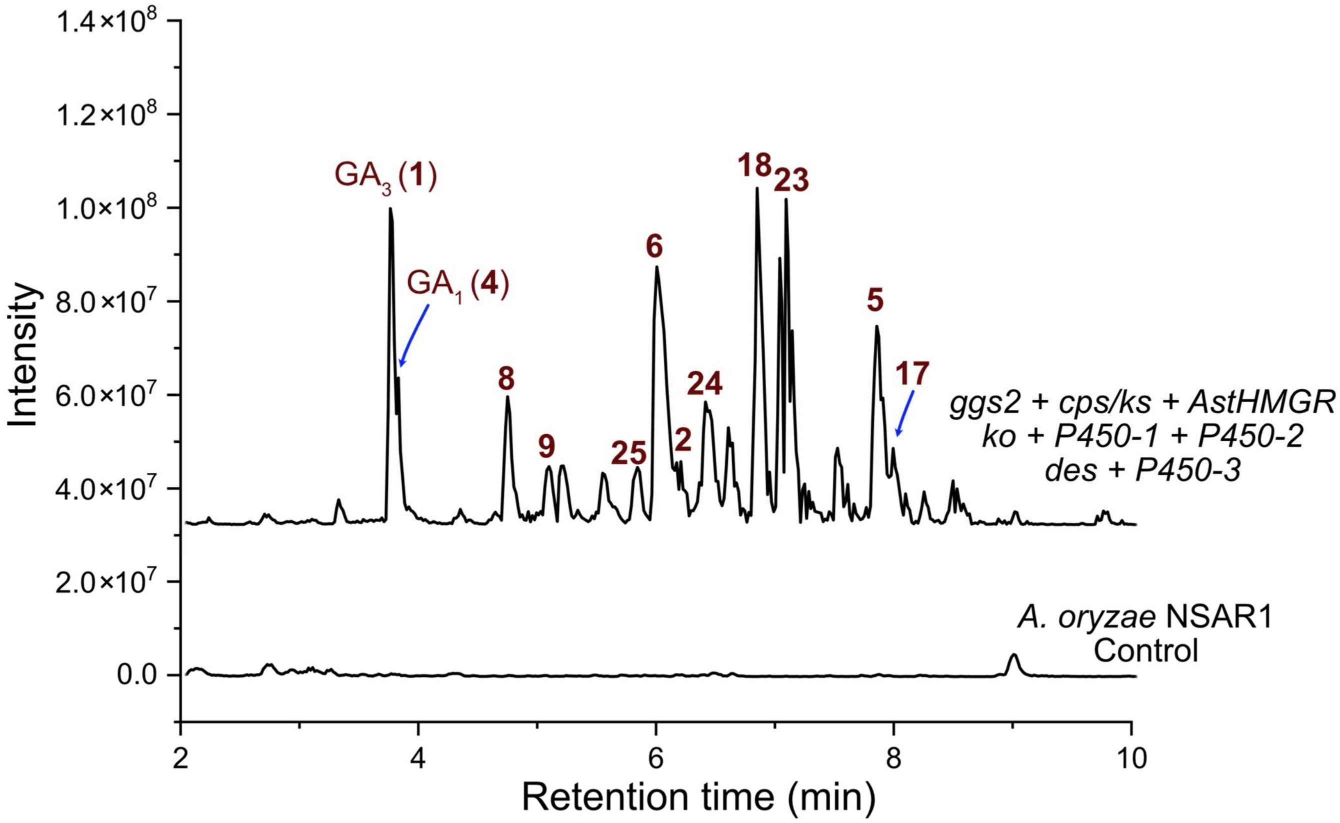

Figure S2. Comparison of metabolite profiles of *A. oryzae* NSAR1 background strain and our full gibberellin platform strain containing *ggs2*, *cps/ks*, *AstHMGR*, *ko*, *P450-1*, *P450-2*, *des*, and *P450-3* genes.

Data shown are base peak chromatograms (negative mode).

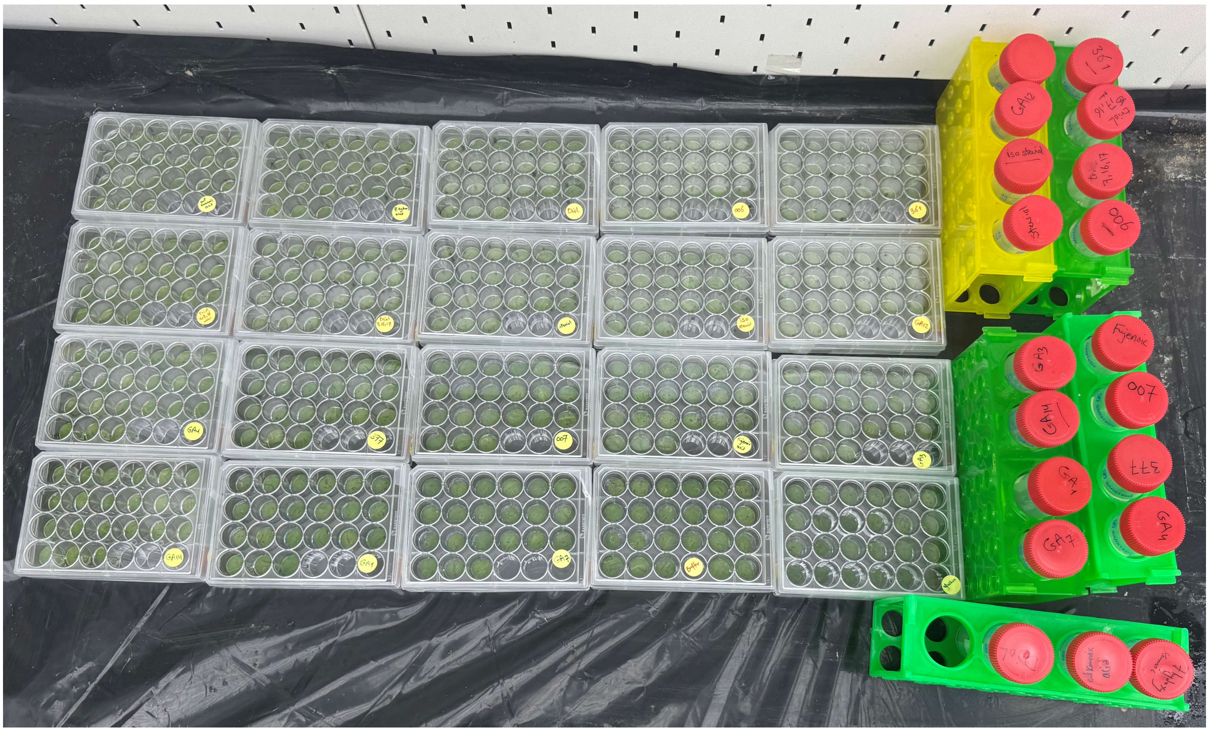

Figure S3. Example set-up for leaf disk screening on analytical scale of six oxidases with 19 of the 20 substrates and a no-substrate control with three biological replicates.

Each 24-well plate was used to test one substrate with six oxidases (columns) in three biological replicates (rows 1-3); additionally, an empty vector (EV) control with three biological replicates was included (row 4).

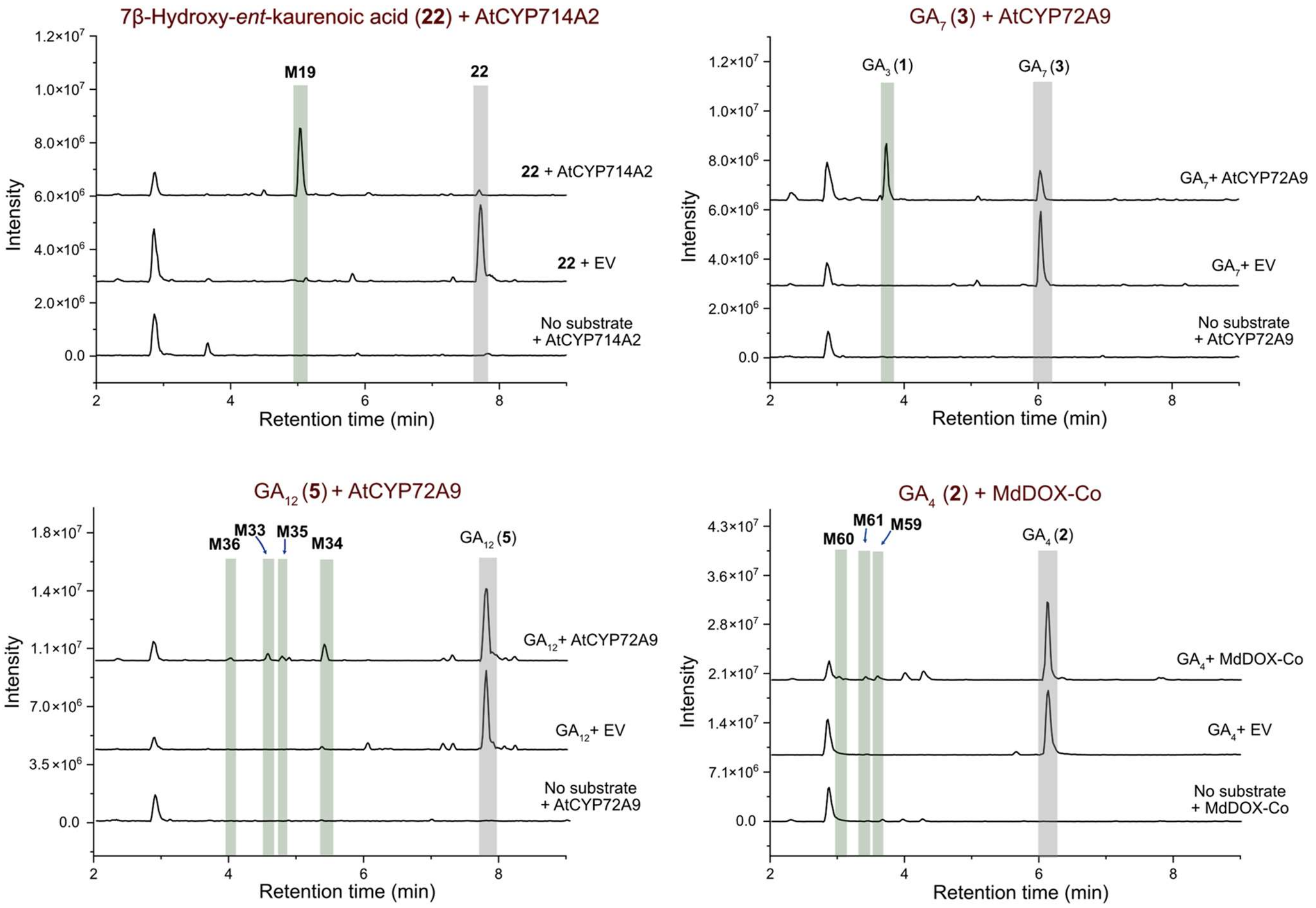

Figure S4. Example chromatograms from leaf disk screening.

Data shown are base peak chromatograms (negative mode). Controls without substrate and without enzyme (EV, empty vector) are included. Substrates are highlighted in gray and products in green.

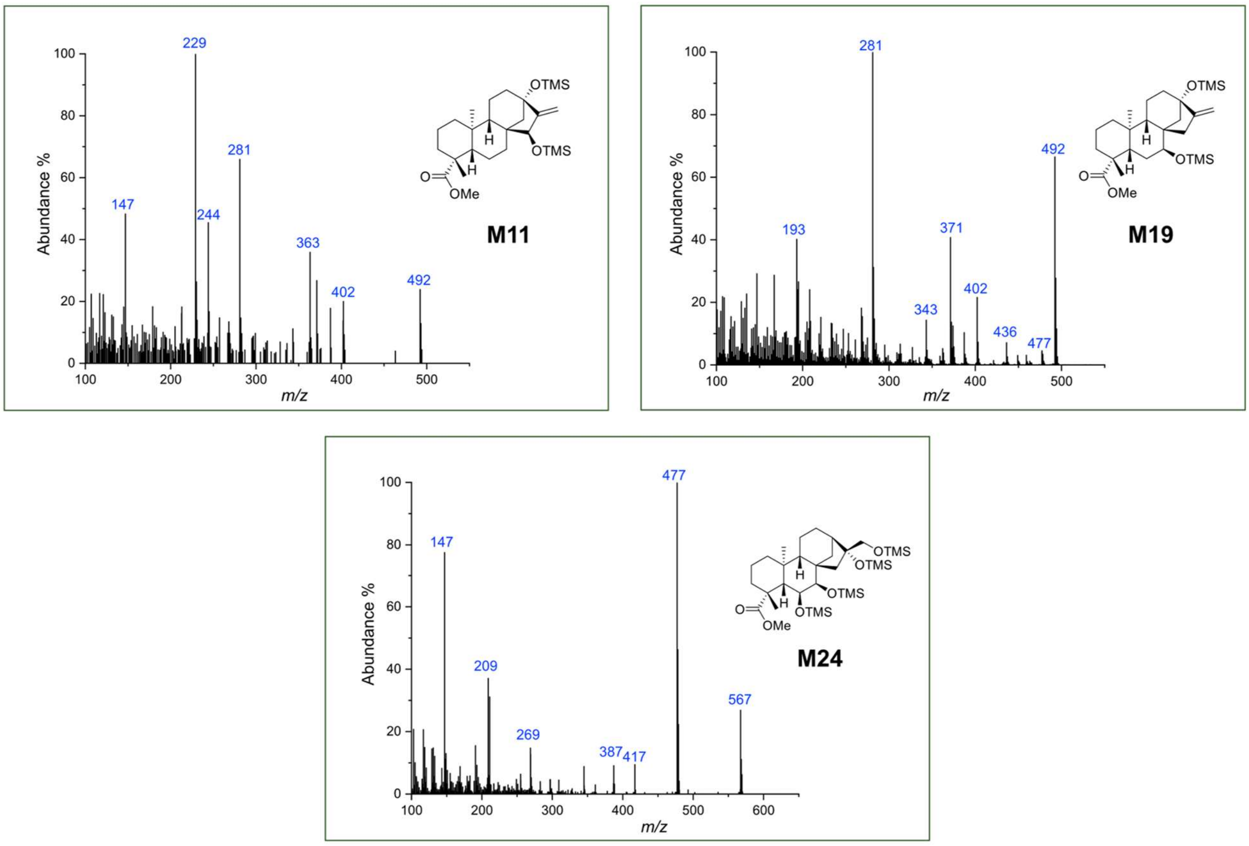

Figure S5. Products from leaf disk screening with *ent*-kaurene/kaurane skeletons identified by GC-MS fragmentation comparison relative to published data.

For a comparison of fragmentation data and literature references see **Table S5**.

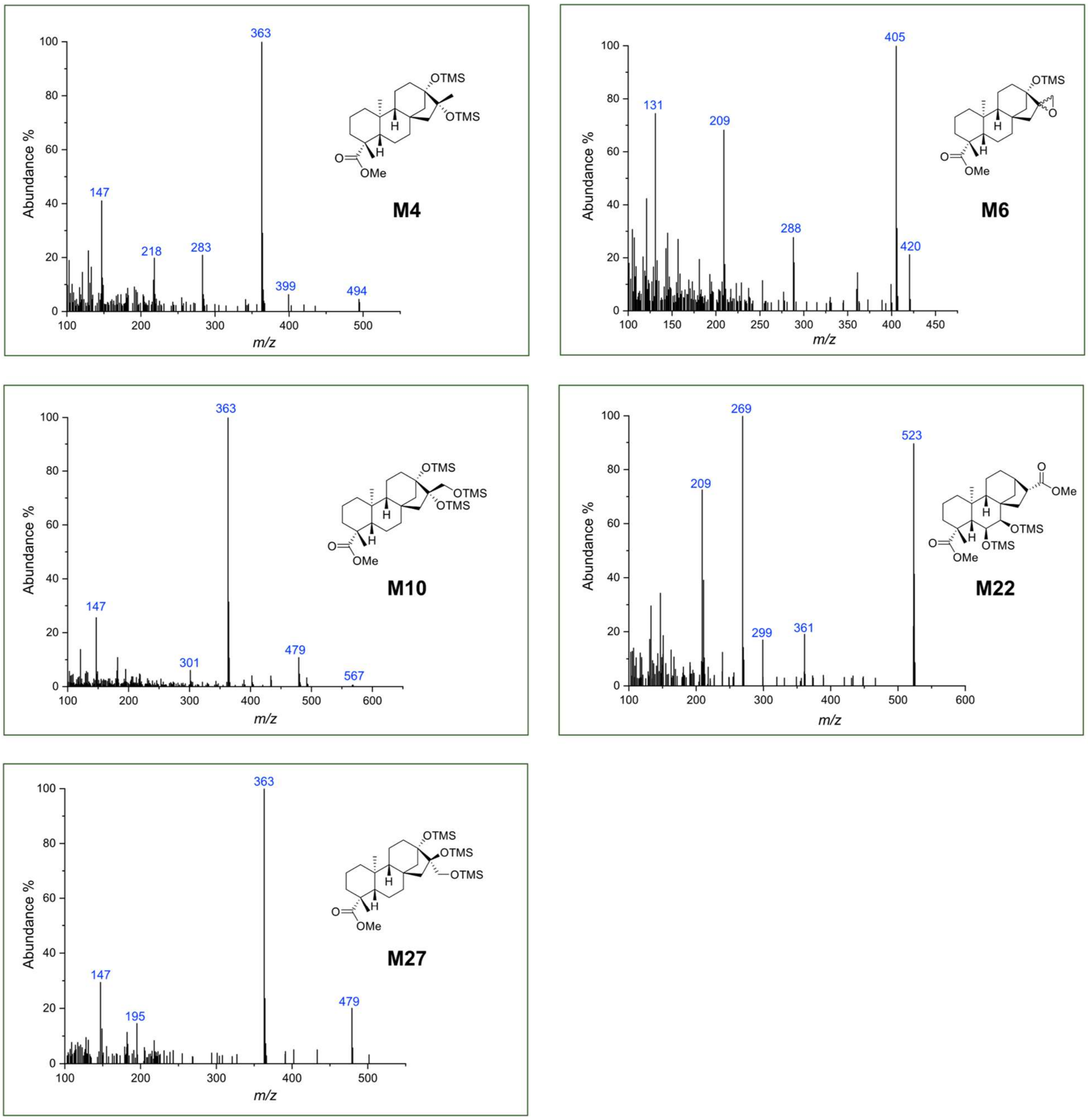

Figure S6. Putative products from leaf disk screening with *ent*-kaurane skeletons proposed based on their GC-MS fragmentation patterns.

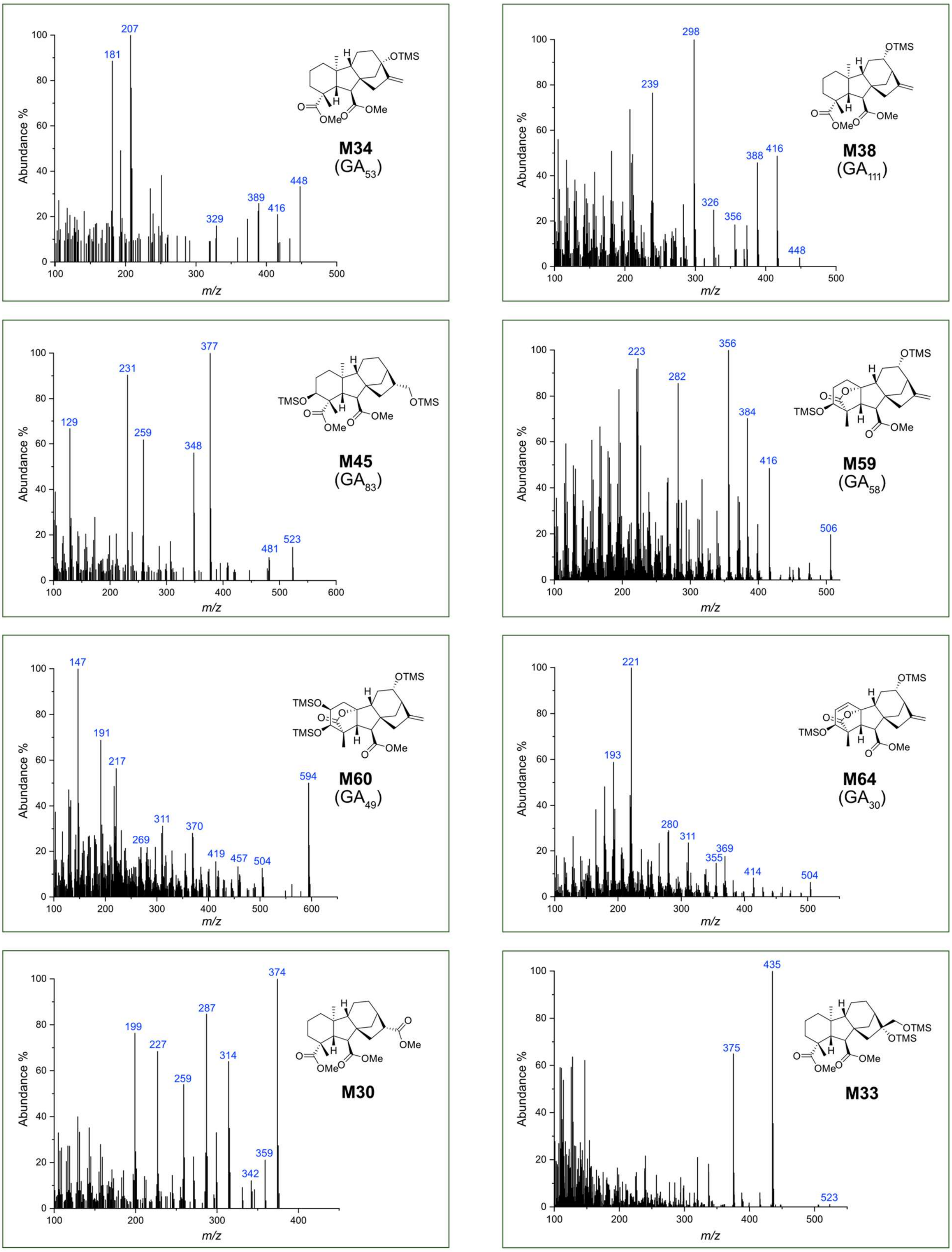

Figure S7. Products from leaf disk screening with gibberellin structures based on GC-MS fragmentation comparison relative to published data. For a comparison of fragmentation data and literature references see **Table S5**.

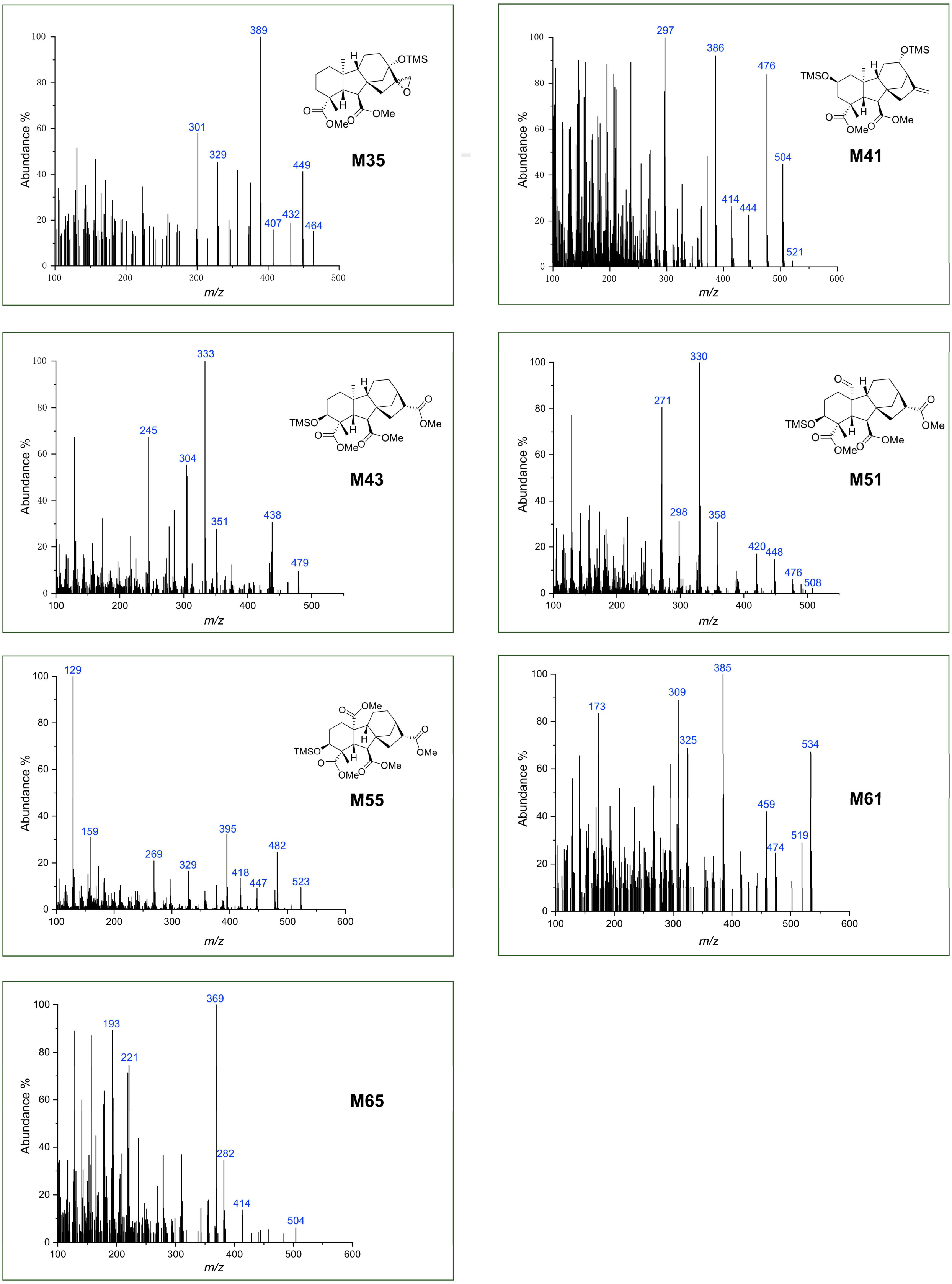

Figure S8. Putative products from leaf disk screening with gibberellin structures proposed based on their GC-MS fragmentation patterns.

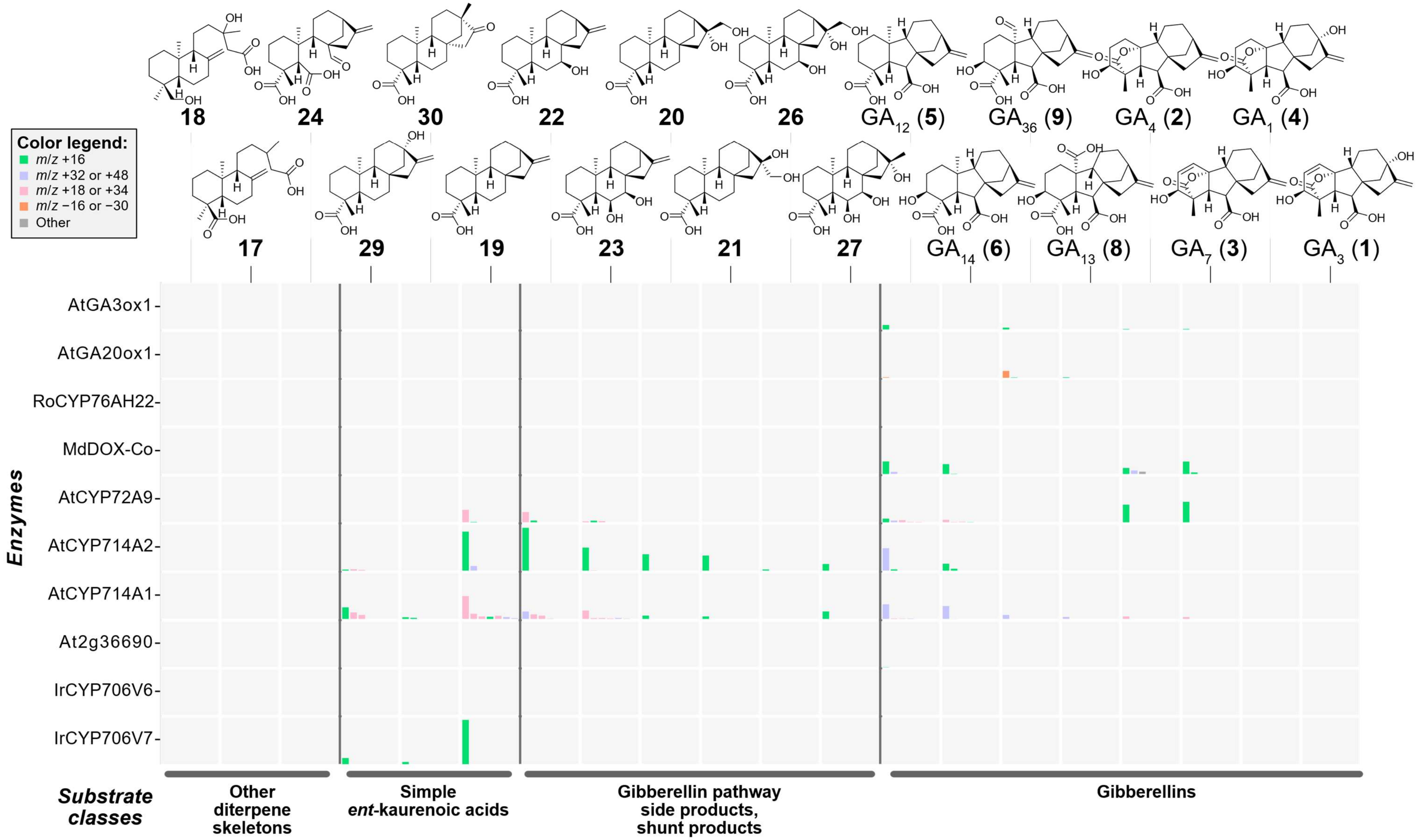

Figure S9. Leaf disk screening results presented as bar plots of product profiles.

Bars indicate product distribution based on conversion of the substrate; the sum of bars corresponds to the total conversion shown in Figure 3. Bar color indicates difference in *m*/*z* values compared to substrate (green: +16; blue: +32/+48; pink: +18/+34; orange: −16/−30; gray: other). Data shown are means of relative LC-MS peak areas (normalized to the summed up peak areas of all products and unconsumed substrate) from three replicate leaf disks. For the metabolite compound numbers, see **Table S4** and Supporting File 2. For the full dataset and product structures, see Supporting Files 1 and 2.

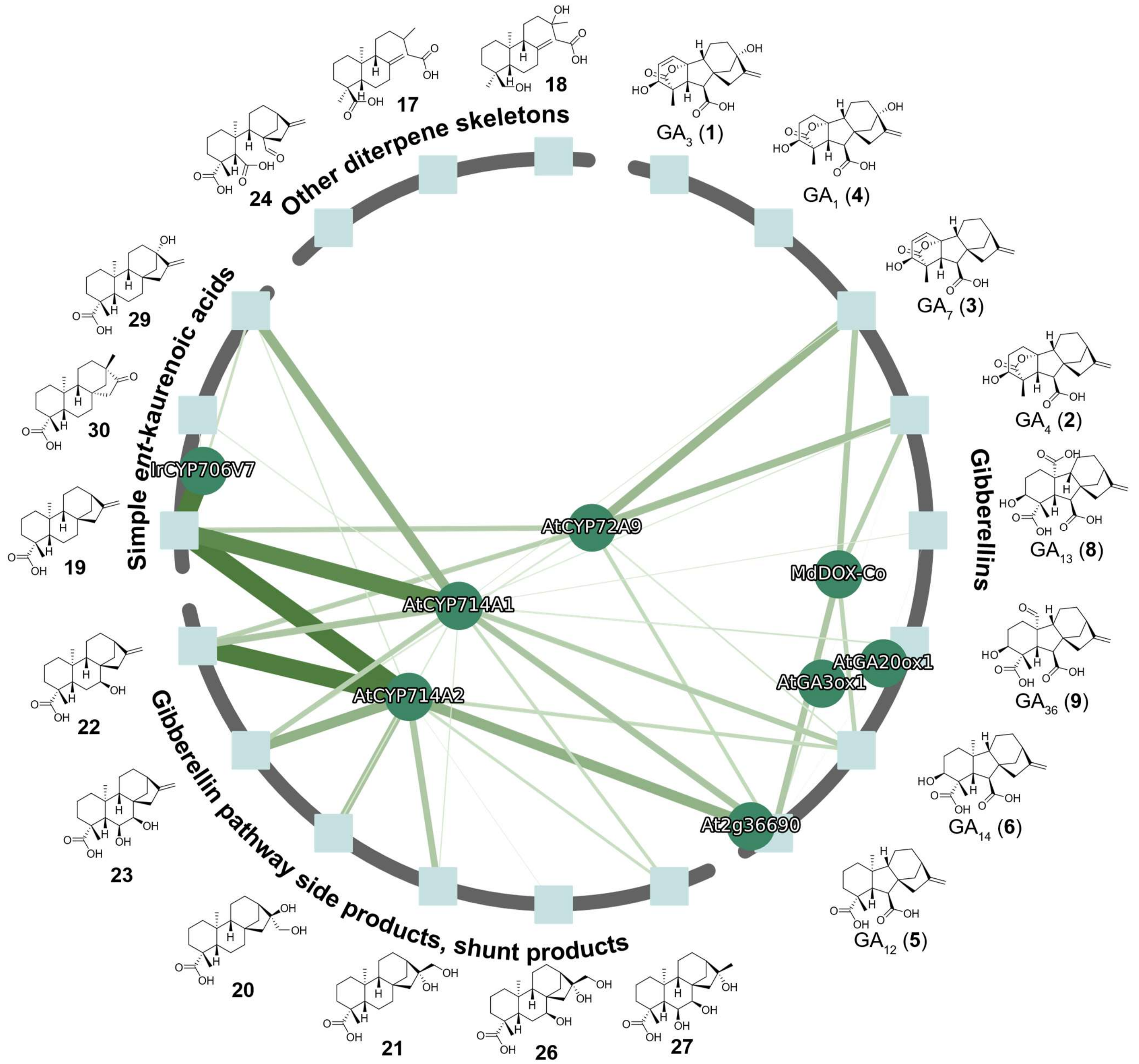

Figure S10. Leaf disk screening results presented as a network graph indicating the preferred substrates and conversion of tested enzymes.

Enzymes (green circles) are positioned relative to their accepted substrates (light blue squares). Edge thickness indicates total conversion as shown in Figure 3. The two enzymes that did not convert any of the tested substrates, IrCYP706V6 and RoCYP76AH22, are omitted for clarity. The graph was generated using the NetworkX package using the spring layout in python.[11]

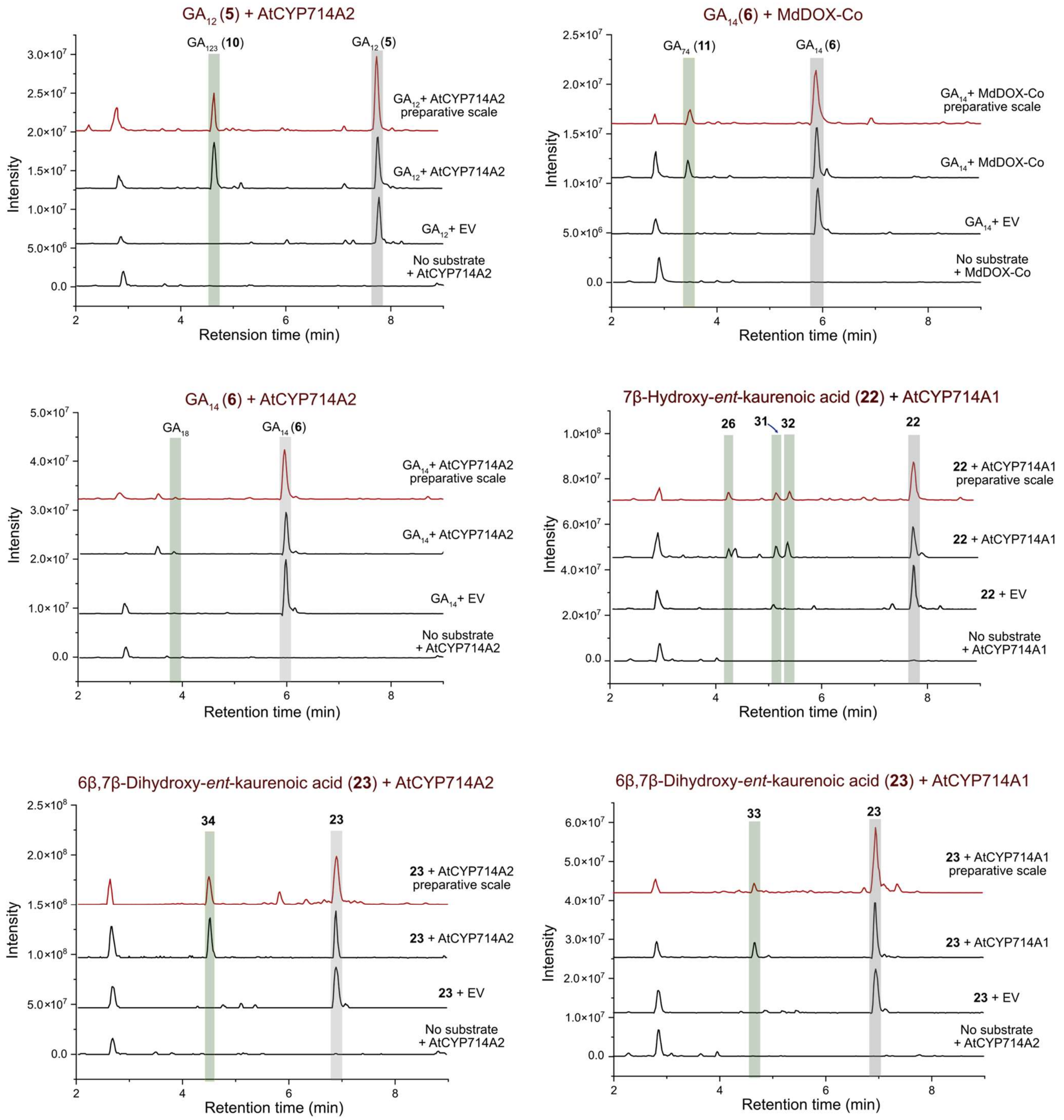

Figure S11. Chromatograms from preparative leaf disk assays in comparison to analytical scale assays.

Controls without substrate and without enzyme (EV, empty vector) are shown. Data shown are base peak chromatograms (negative mode).

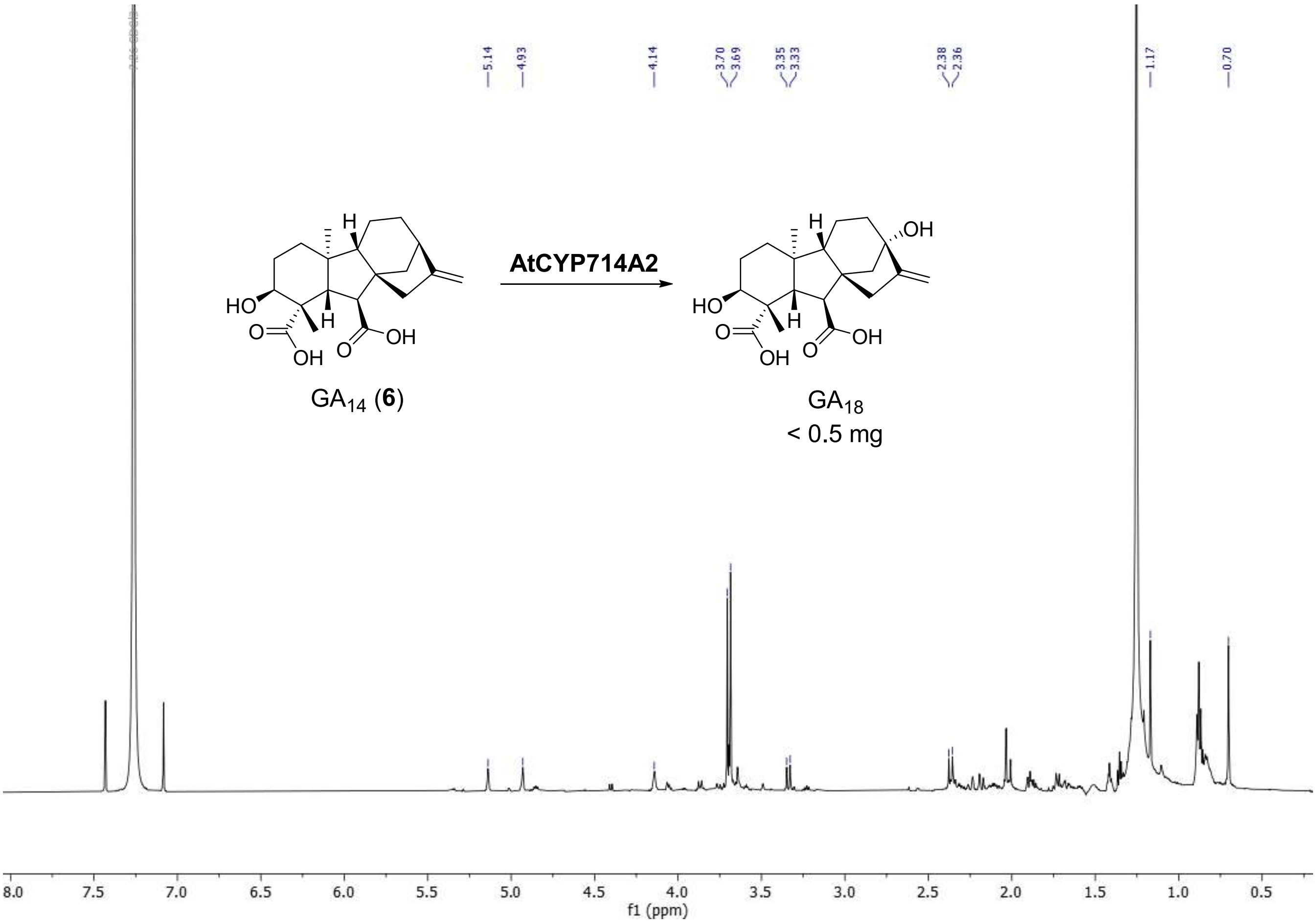

Figure S12. Tentative production of GA_{18} by chemoenzymatic conversion of GA_{14} (**6**) with AtCYP714A2.

Data shown is the ^1H spectrum of the dimethyl ester of the isolated product, which matches very well to reported data for GA_{18} dimethyl ester.[12] Partial ^1H NMR data is reported in **Table S6**. No sufficient amounts for 2D or ^{13}C measurements could be obtained.

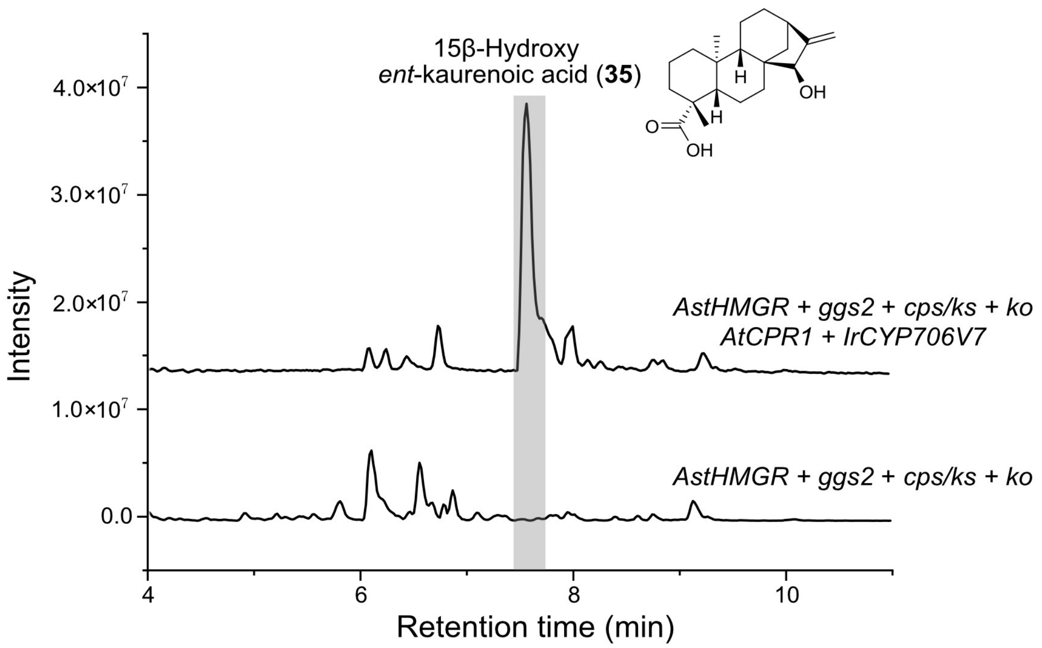

Figure S13. Formation of 15β-hydroxy-*ent*-kaurenoic acid (**35**) in *A. oryzae* transformants containing *IrCYP706V7* and the cytochrome P450 reductase gene *AtCPR1* in addition to the upstream pathway to *ent*-kaurenoic acid (**19**) (*tHMGR+ggs2+cps/ks+ko*).

Data shown are base peak chromatograms (negative mode).

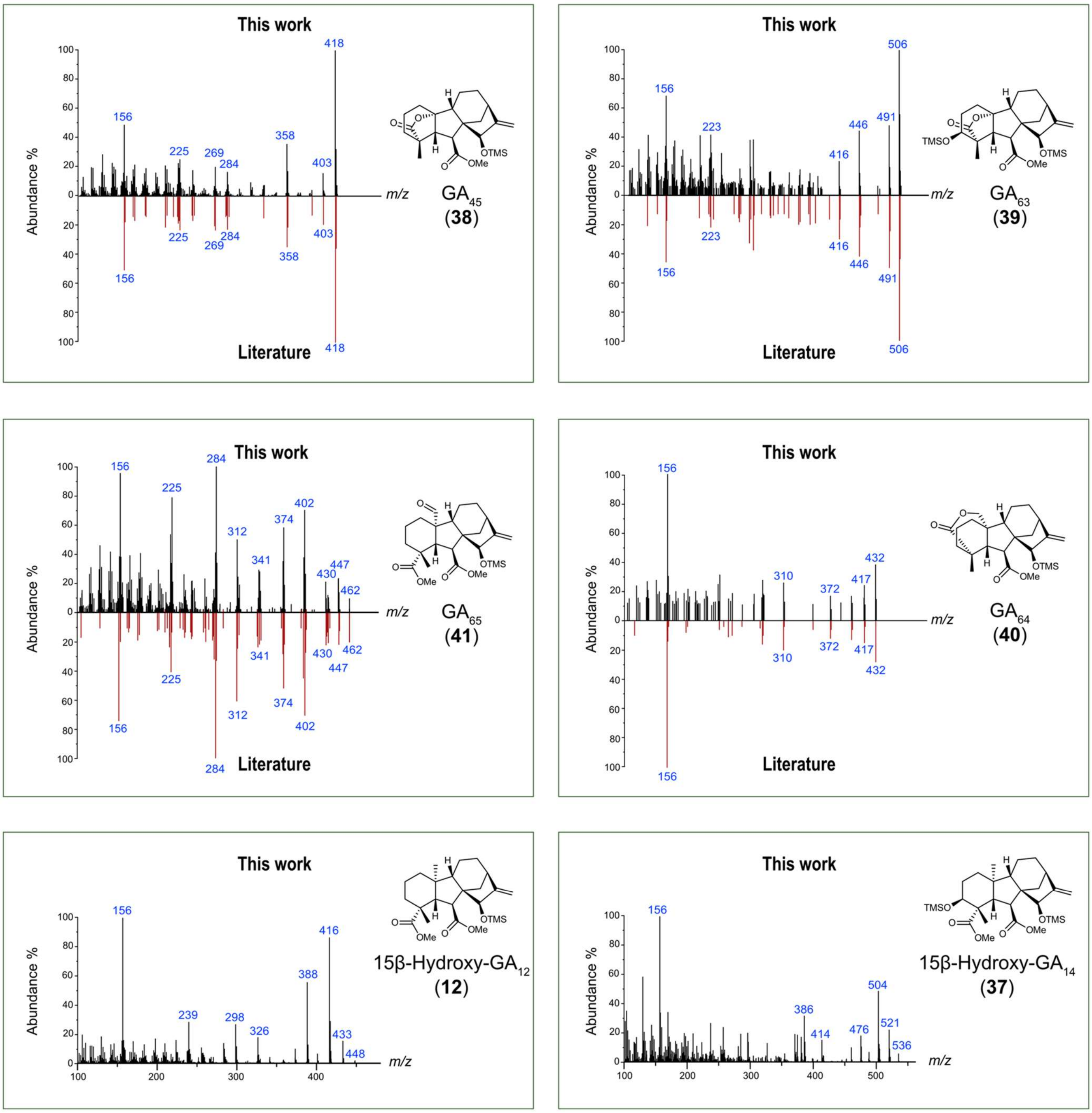

Figure S14. GC-MS fragmentation pattern of 15β-hydroxylated gibberellins produced in *Aspergillus oryzae* with IrCYP706V7 (our data in black against published data in red where available).

For a comparison of fragmentation data and literature references see **Table S5**.

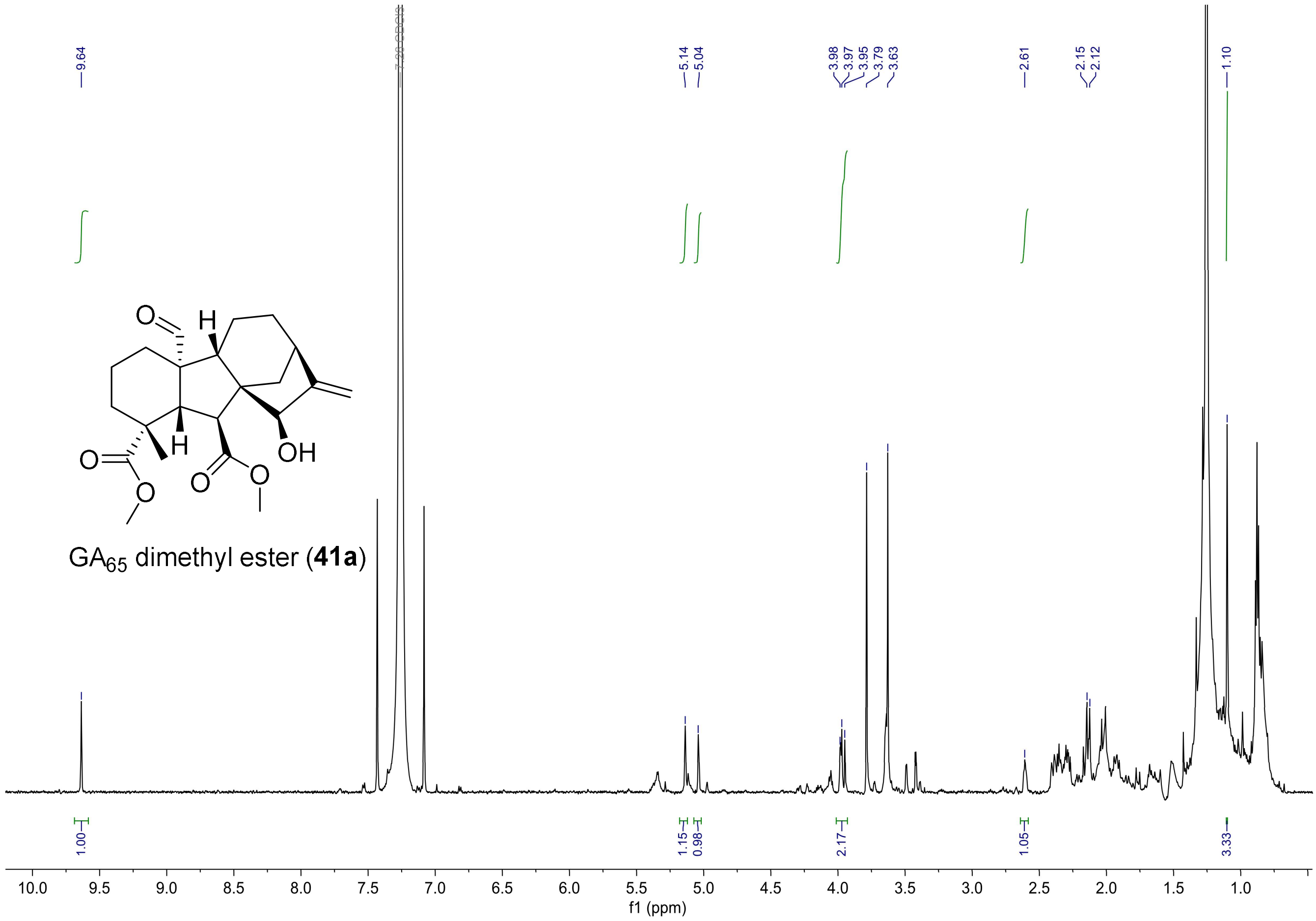

Figure S15. ^{1}H NMR spectrum of GA_{65} dimethyl ester (**41a**) showing an aldehyde proton at 9.64 ppm.

Besides the aldehyde proton, the following signals were assigned: H-5 at 2.13 ppm (d, J = 13.0 Hz, 1H), H-6 at 3.96 ppm (d, J = 13.0 Hz, 1H), H-15 at 3.98 ppm (overlapped, 1H), H-13 at 2.61 ppm (dd, J = 6.0, 6.0 Hz, 1H) H-18 at 1.10 ppm (s, 3H), H-17a at 5.04 ppm (br s ,1H), H-17b at 5.14 ppm (br s, 1H), Me (OMe-1) at 3.63 ppm (s, 3H), and Me (O-Me-2) at 3.79 ppm (s, 3H).

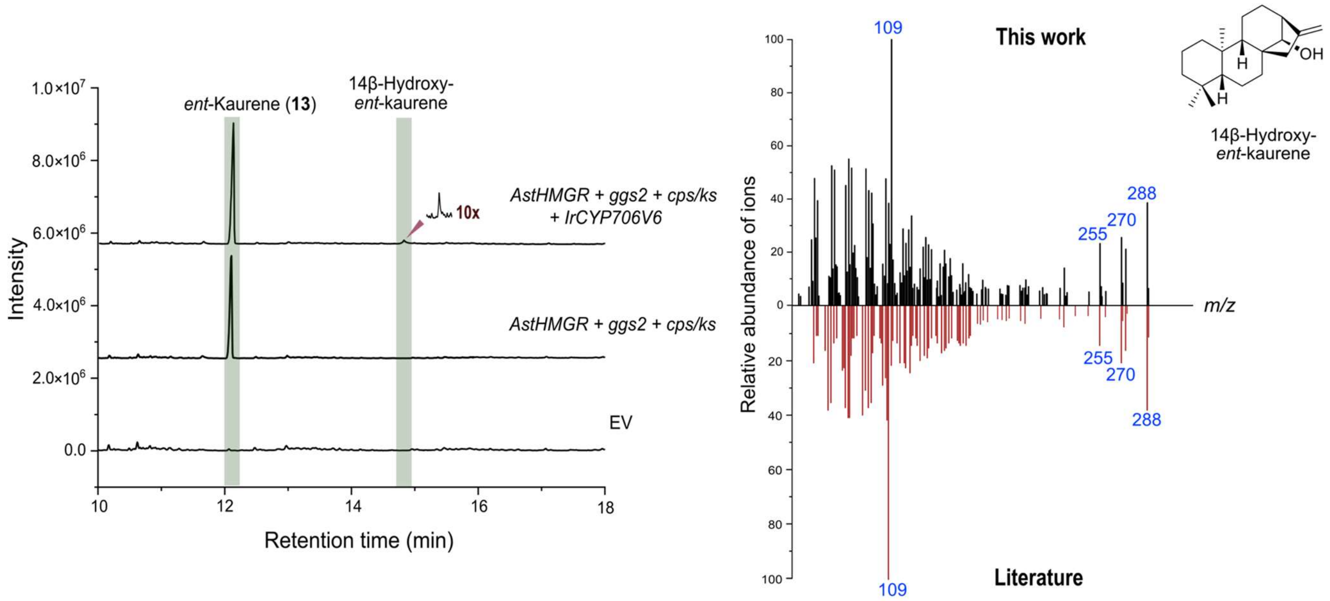

Figure S16. Confirmation that IrCYP706V6 is active in *N. benthamiana* despite its lack of activity with the panel of 20 tested substrates.

Chromatograms shown are total ion chromatograms from GC-MS. The literature mass spectrum was extracted from the figure shown in ref. [13]. The activity reported for IrCYP706V6[13] is comparably small. EV, empty vector.

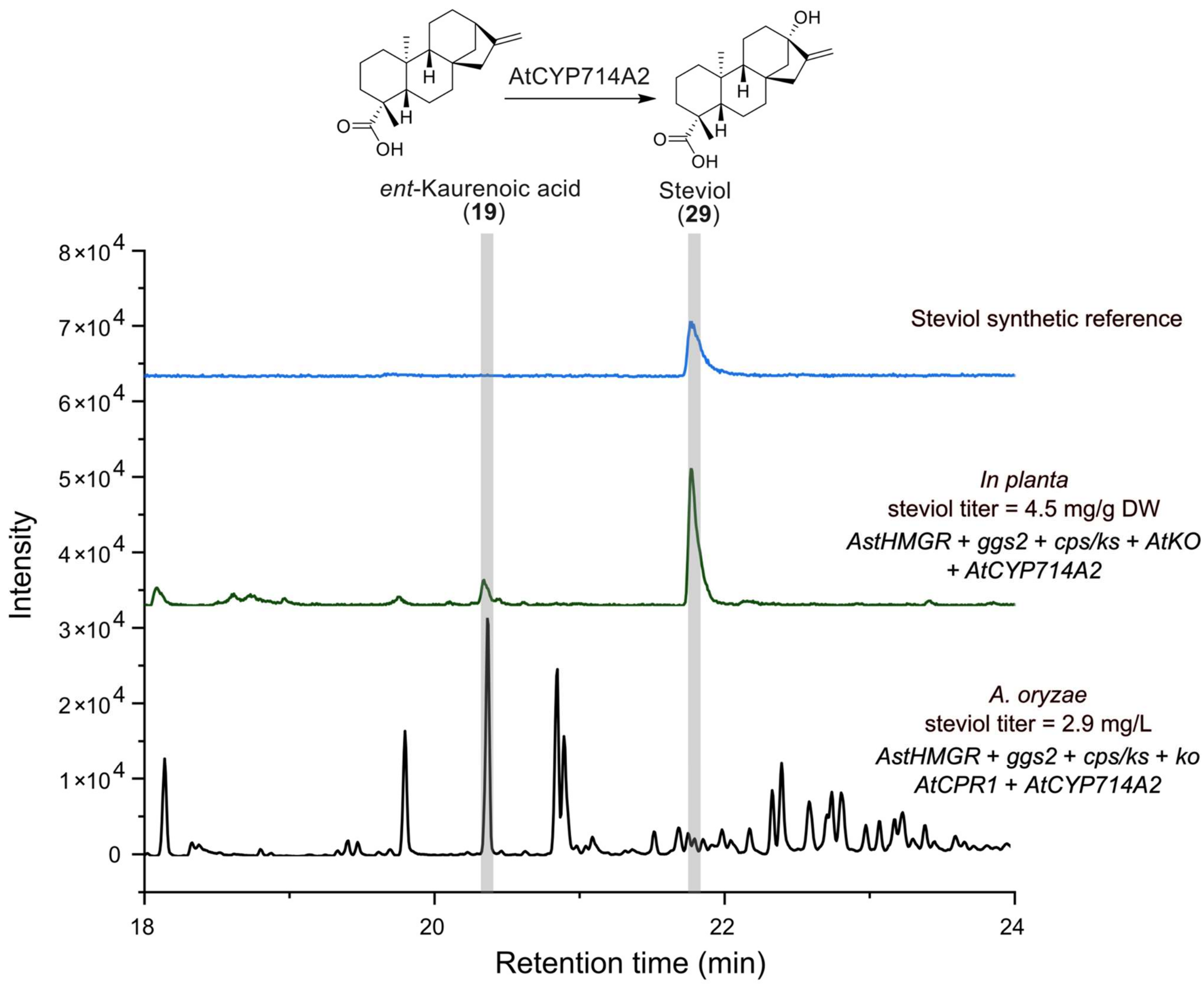

Figure S17. Enzymatic activity of AtCYP714A2 in *A. oryzae* is substantially lower than in *N. benthamiana*.

In *A. oryzae*, the substrate *ent*-kaurenoic acid (**19**) remained largely unconsumed. Data shown are GC-MS total ion chromatograms. The identity of steviol (**29**) was also confirmed by comparison of electron impact mass spectra. Measured titers for steviol (**29**) production in each system are indicated.

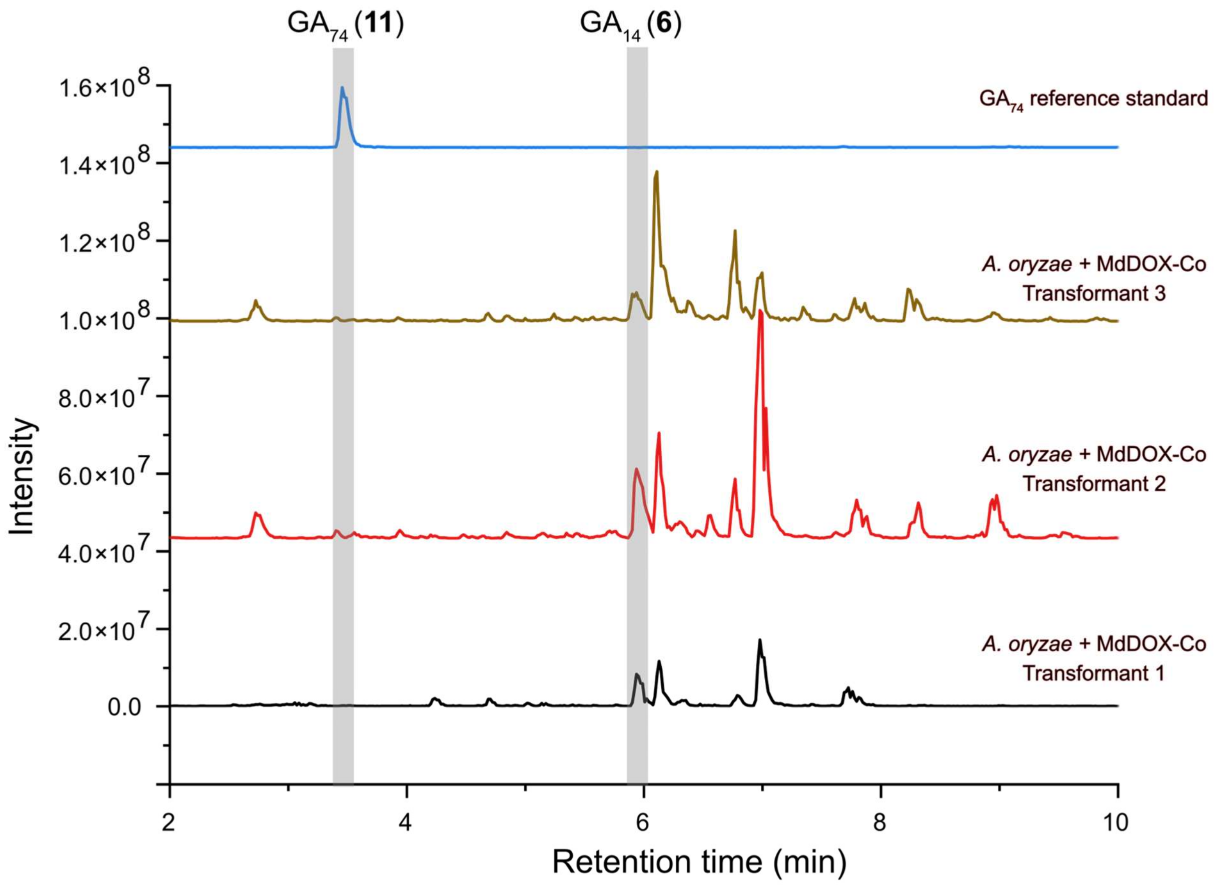

Figure S18. Lack of MdDOX-Co activity in *A. oryzae*.

Data shown are LC-MS base peak chromatograms (negative mode). While MdDOX-Co was able to convert GA_{14} (**6**) to GA_{74} (**11**) in leaf disk biotransformations (Figure 4B), this activity was not observed in *A. oryzae* strains producing GA_{14} (**6**). Over 40 transformants were screened without success.

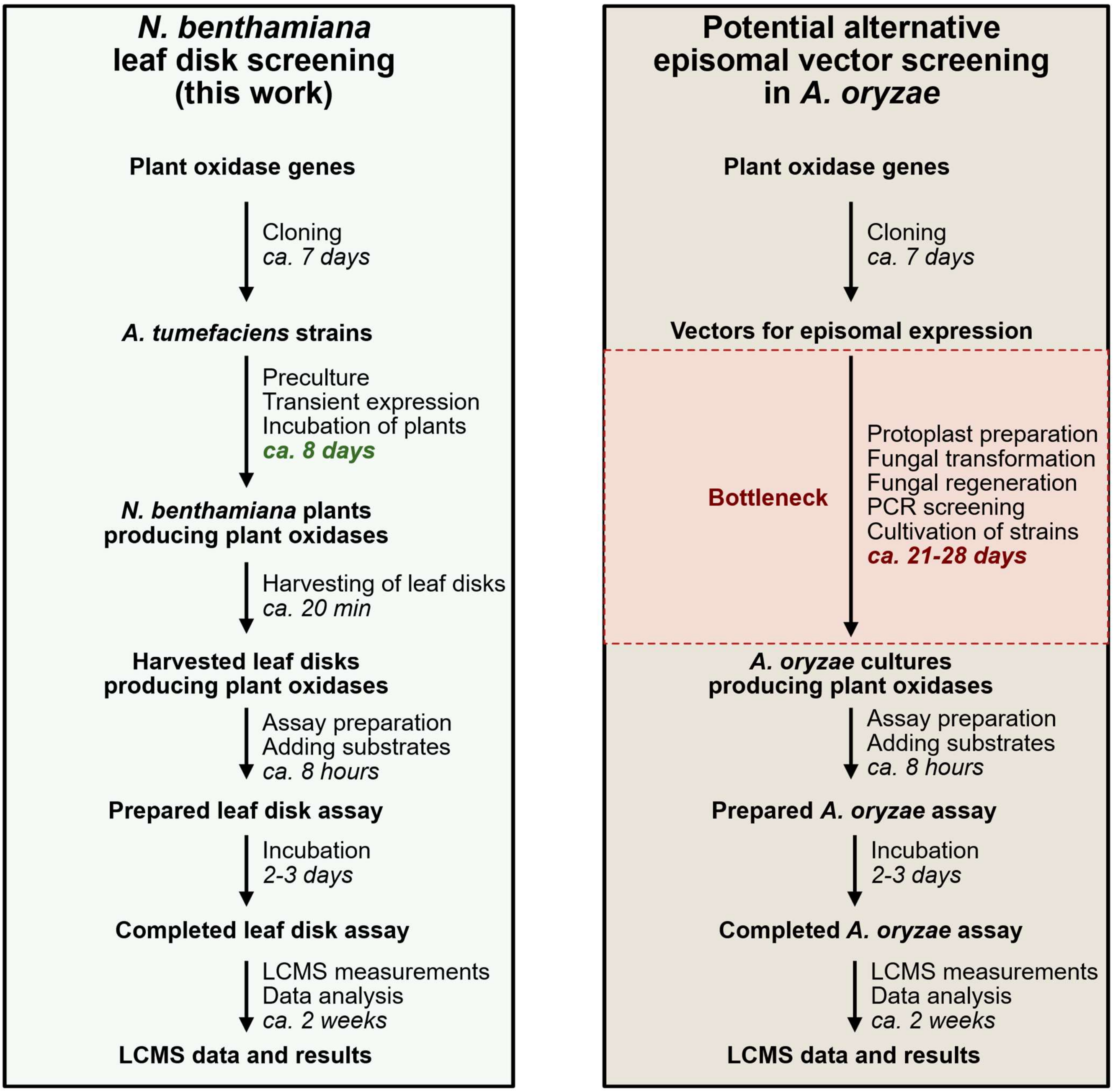

Figure S19. Comparison of the time requirements for leaf disk screening in *N. benthamiana* with a potential analogous process based on episomal vectors in the fungal host *A. oryzae*.

While transient expression in *N. benthamiana* is a very fast and easy process, fungal transformation and expression are much more time-consuming and labor-intensive.

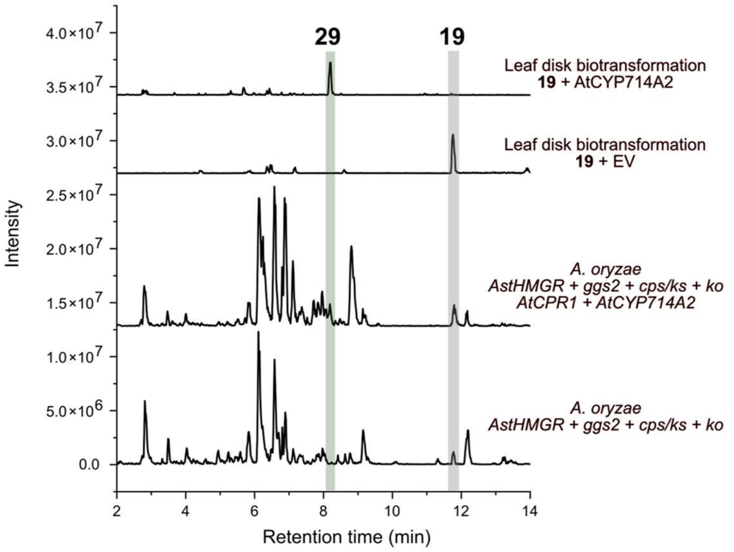

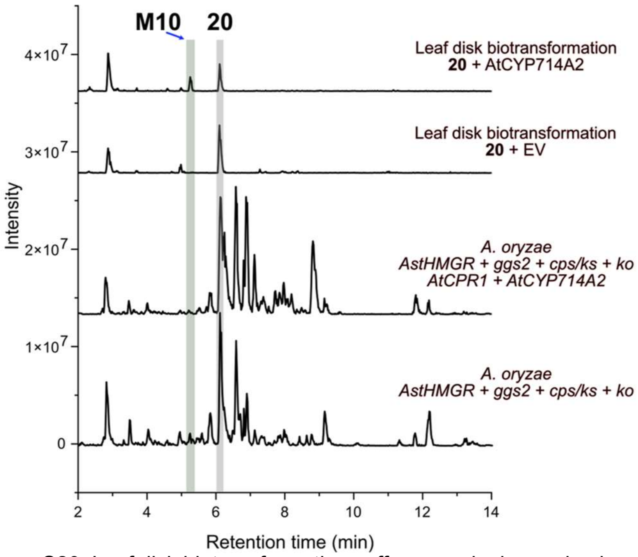

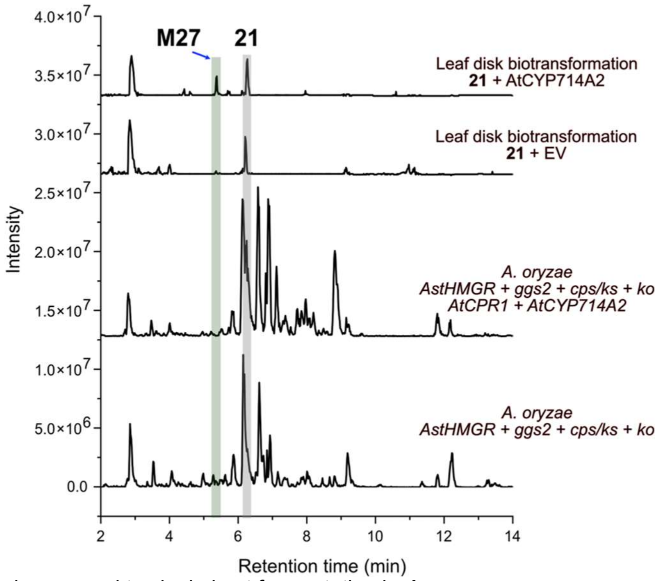

Figure S20. Leaf disk biotransformations offer a much cleaner background compared to single host fermentation in *A. oryzae*.

Data shown are LC-MS base peak chromatograms (negative mode). Substrates are indicated with gray vertical bars while products are highlighted with green vertical bars. EV, empty vector.

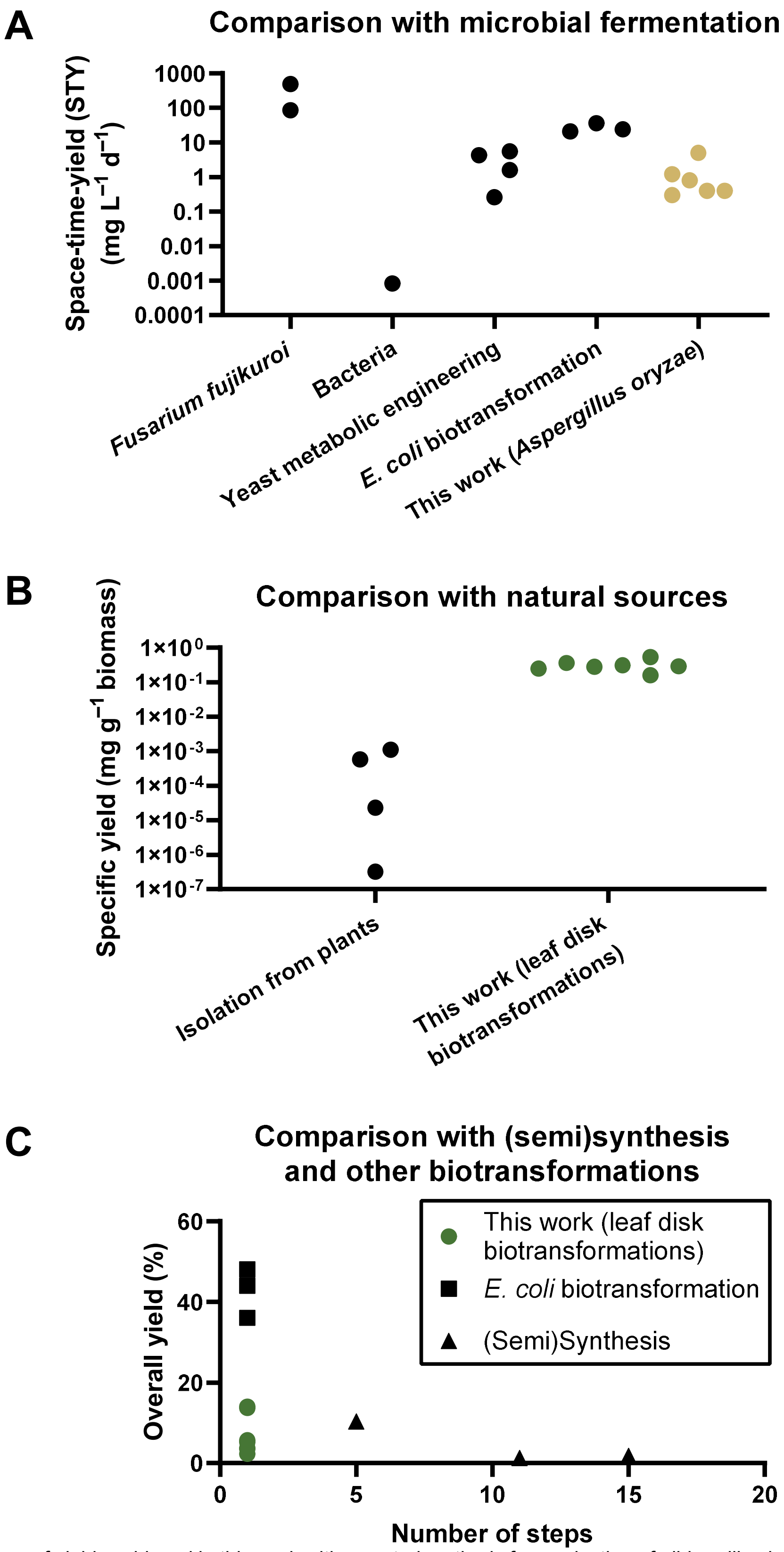

Figure S21. Comparison of yields achieved in this work with reported methods for production of gibberellins in the literature.

(A) Comparison of space-time-yields (mg L^{-1} d^{-1}) of gibberellins produced in liquid culture by microbial fermentation.

(B) Comparison of specific yields (mg g^{-1} biomass) of gibberellins isolated from natural sources with rare gibberellins produced in this work by leaf disk biotransformations.

(C) Comparison of overall yields (%) for chemical (semi)synthesis and other biotransformations with leaf disk biotransformations used in this work in relation to the total number of steps.

Details and literature references are provided in **Table S7**.

Supporting Tables

Table S1. List of compounds fully characterized by NMR spectroscopy and HRMS in this work.

Full NMR characterization data and spectra can be found in section "NMR data and spectra".

Name	Structure	Source	NMR		HRMS
			Modification	Solvent	
**Rare gibberellins**					
GA$_{12}$ (**5**)	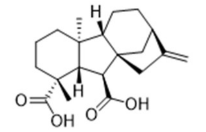	Original *A. oryzae* platform	-------	DMSO-d$_6$	Calculated for $C_{20}H_{27}O_4^-$: 331.1915<br>Found: 331.1916
GA$_{14}$ (**6**)	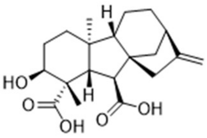	Original *A. oryzae* platform	-------	C_5D_5N<br>DMSO-d$_6$	Calculated for $C_{20}H_{27}O_5^-$: 347.1864<br>Found: 347.1853
GA$_9$ (**7**)	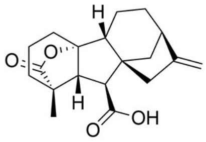	Original *A. oryzae* platform	Methylation	$CDCl_3$	Calculated for $C_{19}H_{23}O_4^-$: 315.1602<br>Found: 315.1600
GA$_{13}$ (**8**)	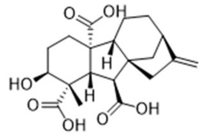	Original *A. oryzae* platform	-------	C_5D_5N<br>DMSO-d$_6$	Calculated for $C_{20}H_{25}O_7^-$: 377.1606<br>Found: 377.1599
GA$_{36}$ (**9**)	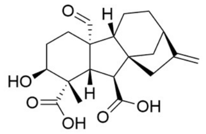	Original *A. oryzae* platform	Methylation	$CDCl_3$	Calculated for $C_{20}H_{25}O_6^-$: 361.1657<br>Found: 361.1637
GA$_{123}$ (**10**)	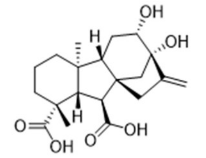	Chemoenzymatic synthesis	-------<br>Methylation	DMSO-d$_6$<br>$CDCl_3$	Calculated for $C_{20}H_{27}O_6^-$: 363.1813<br>Found: 363.1794
GA$_{74}$ (**11**)	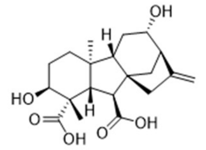	Chemoenzymatic synthesis	-------<br>Methylation	DMSO-d$_6$<br>$CDCl_3$	Calculated for $C_{20}H_{27}O_6^-$: 363.1813<br>Found: 363.1821
15β-Hydroxy-GA$_{12}$ (**12**)	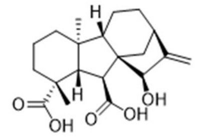	*A. oryzae* with *IrCYP706V7*	Methylation	MeOD	Calculated for $C_{20}H_{27}O_5^-$: 347.1864<br>Found: 347.1881
**Common gibberellins**					
GA$_3$ (**1**)	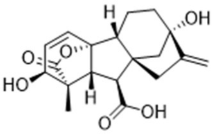	Original *A. oryzae* platform	-------	C_5D_5N	Calculated for $C_{19}H_{21}O_6^-$: 345.1344<br>Found: 345.1335
GA$_4$ (**2**)	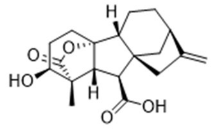	Original *A. oryzae* platform	-------	C_5D_5N<br>DMSO-d$_6$	Calculated for $C_{19}H_{23}O_5^-$: 331.1551<br>Found: 331.1575
GA$_1$ (**4**)	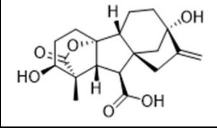	Original *A. oryzae* platform	-------	C_5D_5N	Calculated for $C_{19}H_{23}O_6^-$: 347.1500<br>Found: 347.1519

***Ent*-kaurene derivatives**					
16α,17-Dihydroxy-*ent*-kauranoic acid (**20**)	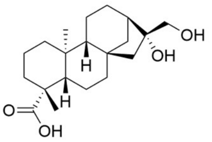	Original *A. oryzae* platform	-------	DMSO-d_6	Calculated for $C_{20}H_{31}O_4^-$: 335.2228<br>Found: 335.2216
16β,17-Dihydroxy-*ent*-kauranoic acid (**21**)	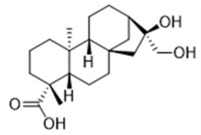	Original *A. oryzae* platform	-------	DMSO-d_6	Calculated for $C_{20}H_{31}O_4^-$: 335.2228<br>Found: 335.2211
7β-Hydroxy-*ent*-kaurenoic acid (**22**)	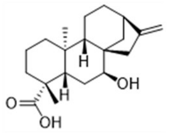	Original *A. oryzae* platform	-------	$CDCl_3$	Calculated for $C_{20}H_{29}O_3^-$: 317.2122<br>Found: 317.2129
				MeOD	
7β-Hydroxy-16-*epi*-*ent*-kauran-17,19-dioic acid (**32**)	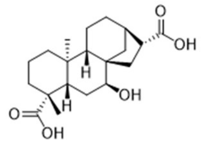	Chemoenzymatic synthesis	-------	DMSO-d_6	Calculated for $C_{20}H_{29}O_5^-$: 349.2020<br>Found: 349.2000
			Methylation	$CDCl_3$	
7β,17-Dihydroxy-16-*epi*-*ent*-kauranoic acid (**31**)	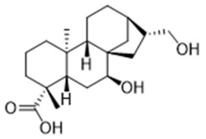	Chemoenzymatic synthesis	-------	DMSO-d_6	Calculated for $C_{20}H_{31}O_4^-$: 335.2228<br>Found: 335.2201
			Methylation	$CDCl_3$	
7β,16α,17-Trihydroxy-*ent*-kauranoic acid (**26**)	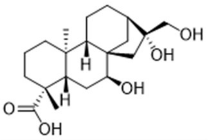	Original *A. oryzae* platform	-------	DMSO-d_6	Calculated for $C_{20}H_{31}O_5^-$: 351.2177<br>Found: 351.2159
		Chemoenzymatic synthesis	Methylation	$CDCl_3$	
6β,7β-Dihydroxy-*ent*-kaurenoic acid (**23**)	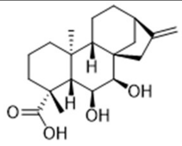	Original *A. oryzae* platform	-------	C_5D_5N	Calculated for $C_{20}H_{29}O_4^-$: 333.2071<br>Found: 333.2065
				MeOD	
6β,7β,16α-Trihydroxy-*ent*-kauranoic acid (**27**)	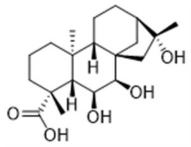	Original *A. oryzae* platform	-------	C_5D_5N	Calculated for $C_{20}H_{31}O_5^-$: 351.2177<br>Found: 351.2152
				DMSO-d_6	
6β,7β,13-Trihydroxy-*ent*-kaurenoic acid (**34**)	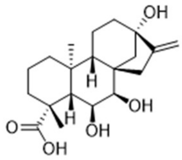	Chemoenzymatic synthesis	-------	C_5D_5N	Calculated for $C_{20}H_{29}O_5^-$: 349.2020<br>Found: 349.2036
6β,7β,17-Trihydroxy-16-*epi*-*ent*-kauranoic acid (**33**)	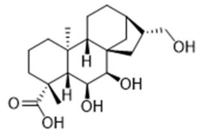	Chemoenzymatic synthesis	-------	MeOD	Calculated for $C_{20}H_{31}O_5^-$: 351.2177<br>Found: 351.2180
15β-Hydroxy-*ent*-kaurenoic acid (**35**)	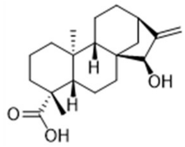	*A. oryzae* with *IrCYP706V7*	-------	$CDCl_3$	Calculated for $C_{20}H_{29}O_3^-$: 317.2122<br>Found: 317.2123
7β,15β-Dihydroxy-*ent*-kaurenoic acid (**36**)	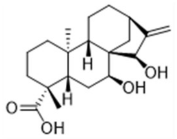	*A. oryzae* with *IrCYP706V7*	-------	C_5D_5N	Calculated for $C_{20}H_{29}O_4^-$: 333.2071<br>Found: 333.2102

7β,15β-Dihydroxy-*ent*-kaurenolide (**42**)	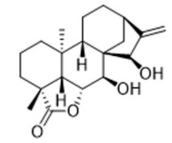	*A. oryzae* with *IrCYP706V7*	-------	MeOD	Calculated for $C_{21}H_{29}O_6^-$: 377.1970 ($[M+HCOOH-H]^-$)<br><br>Found: 377.1932
3β,7β,15β-Trihydroxy-*ent*-kaurenoic acid (**44**)	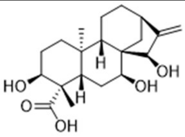	*A. oryzae* with *IrCYP706V7*	-------	MeOD	Calculated for $C_{20}H_{29}O_5^-$: 349.2020<br><br>Found: 349.2032
1β,7β,15β-Trihydroxy-*ent*-kaurenoic acid (**43**)	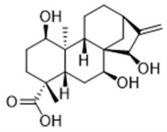	*A. oryzae* with *IrCYP706V7*	-------	MeOD	Calculated for $C_{20}H_{29}O_5^-$: 349.2020<br><br>Found: 349.2011
6β,7β,15β-Trihydroxy-*ent*-kaurenoic acid (**45**)	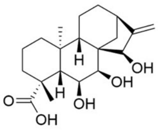	*A. oryzae* with *IrCYP706V7*	Methylation	$CDCl_3$	Calculated for $C_{20}H_{29}O_5^-$: 349.2020<br><br>Found: 349.2018
**Other diterpenoids**					
1,10-Didehydro-20-nor-GA$_{14}$ (**28**)	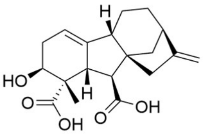	Original *A. oryzae* platform	Methylation	$CDCl_3$	Calculated for $C_{19}H_{23}O_5^-$: 331.1551<br><br>Found: 331.1556
Fujenoic acid (**24**)	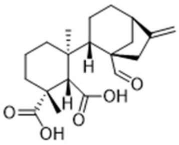	Original *A. oryzae* platform	-------	DMSO-d_6	Calculated for $C_{20}H_{27}O_5^-$: 347.1864<br><br>Found: 347.1853
				C_5D_5N	
			Methylation	$CDCl_3$	
Fujenoic triacid (**25**)	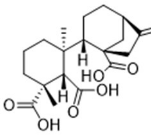	Original *A. oryzae* platform	Methylation	$CDCl_3$	Calculated for $C_{20}H_{27}O_6^-$: 363.1813<br><br>Found: 363.1793
ent-Labd-8(17)-en-15,18-dioic acid (**17**)	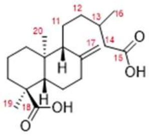	Original *A. oryzae* platform	-------	DMSO-d_6	Calculated for $C_{20}H_{31}O_4^-$: 335.2228<br><br>Found: 335.2228
13,18-Dihydroxy-*ent*-labd-8(17)-en-15-oic acid (**18**)	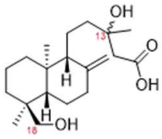	Original *A. oryzae* platform	-------	MeOD	Calculated for $C_{20}H_{33}O_4^-$: 337.2384<br><br>Found: 337.2359
Steviol (**29**)	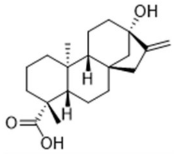	Stevioside hydrolysis	-------	$CDCl_3$	Calculated for $C_{20}H_{29}O_3^-$: 317.2122<br><br>Found: 317.2108
Isosteviol (**30**)	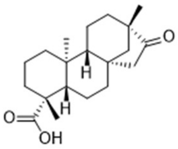	Stevioside hydrolysis	-------	$CDCl_3$	Calculated for $C_{20}H_{29}O_3^-$: 317.2122<br><br>Found: 317.2114

Table S2. Summary of constructed vectors and main products in *A. oryzae* NSAR1.

	pTYGS·*argB·AstHMGR·ggs2·cps/ks*			pTYGS·*adeA·ko·P450-1·P450-2*			pTYGS·*sC·des·P450-3*		**Main products**
**Expt**	***AstHMGR***	***ggs2***	***cps/ks***	***ko***	***P450-1***	***P450-2***	***des***	***P450-3***	
**1**	✓	✓	✓						*ent*-kaurene (**13**) & shunts
**2**	✓	✓	✓	✓					*ent*-kaurenoic acid (**19**) & shunts
**3**	✓	✓	✓	✓	✓				GA_{14} (**6**) + side products
**4**	✓	✓	✓	✓	✓	✓			GA_4 (**2**) + GA_{13} (**8**) + GA_{36} (**9**)
**5**	✓	✓	✓	✓	✓	✓	✓		GA_4 (**2**) + GA_7 (**3**)
**6**	✓	✓	✓	✓	✓	✓	✓	✓	GA_1 (**4**) + GA_3 (**1**) + GA_4 (**2**)
**7**	✓	✓	✓	✓	✓	✓		✓	GA_4 (**2**) + GA_1 (**4**)

Table S3. List of ten previously reported diterpenoid oxidases screened in this work. CYP, cytochrome P450 monooxygenase; ODD, oxoglutarate-dependent dioxygenase.

Enzyme	Type	Native substrate	Reference
AtGA3ox1	ODD	GA_9 (**7**)	[14]
AtGA20ox1	ODD	GA_{12} (**5**)	[15]
RoCYP76AH22	CYP	Abietatriene	[16,17]
MdDOX-Co	ODD	GA_4 (**2**) & GA_{12} (**5**) & GA_9 (**7**)	[18]
AtCYP72A9	CYP	GA_{12} (**5**) & GA_9 (**7**)	[19]
AtCYP714A2	CYP	*Ent*-kaurenoic acid (**19**)	[20]
AtCYP714A1	CYP	GA_{12} (**5**)	[20]
At2g36690	ODD	GA_{12} (**5**)	[21,22]
IrCYP706V6	CYP	*Ent*-kaurene (**13**)	[13]
IrCYP706V7	CYP	*Ent*-kaurene (**13**)	[13]

Table S4. Summary of metabolites produced during leaf disk screening of the ten plant oxidases with the 20 substrates.

A total of 91 product peaks were observed, which were assigned to 65 unique metabolites numbered from M1 to M65. For metabolites that are identical to compounds from the main manuscript the corresponding compound number is provided in brackets. For each metabolite, the relative LC-MS peak area (normalized to the summed up peak areas of all products and unconsumed substrate) as well as the mass shift relative to the substrate are indicated.

	13,18-Dihydroxy-*ent*-labd-8(17)-en-15-oic acid **(18)**	*ent*-Labd-8(17)-en-15,18-dioic acid **(17)**	Fujenoic acid **(24)**	Steviol **(29)**	Isosteviol **(30)**	*Ent*-kaurenoic acid **(19)**	7β-Hydroxy-*ent*-kaurenoic acid **(22)**	6β,7β-Dihydroxy-*ent*-kaurenoic acid **(23)**	16α,17-Dihydroxy-*ent*-kauranoic acid **(20)**	16β,17-Dihydroxy-*ent*-kauranoic acid **(21)**	7β,16α,17-Trihydroxy-*ent*-kauranoic acid **(26)**	6β,7β,16α-Trihydroxy-*ent*-kauranoic acid **(27)**	GA_{12} **(5)**	GA_{14} **(6)**	GA_{36} **(9)**	GA_{13} **(8)**	GA_{4} **(2)**	GA_{7} **(3)**	GA_{1} **(4)**	GA_{3} **(1)**
AtGA3ox1													**M39** (**6**)<br>9.8%, +16		**M52**<br>4.0%, +16		**M58** (**4**)<br>1.0%, +16	**M63** (**1**)<br>1.1%, +16		
AtGA20ox1													**M40** (**7**)<br>1.1%, –16		**M53** (**2**)<br>14.9%, –30<br>**M54** (**8**)<br>0.8%, +16	**M56**<br>1.1%, +16				
RoCYP76 AH22																				
MdDOX-Co													**M38**<br>27.4%, +16<br>**M41**<br>4.8%, +32<br>**M42**<br>0.4%, +32	**M48** (**11**)<br>21.5%, +16<br>**M50**<br>0.7%, +16			**M59**<br>13.2%, +16<br>**M60**<br>7.8%, +32<br>**M61**<br>5.1%, +30	**M64**<br>27.1%, +16<br>**M65**<br>3.5%, +16		
AtCYP72A9						**M1** (**20**)<br>27.2%, +34<br>**M5** (**29**)<br>1.0%, +16	**M17** (**26**)<br>22.4%, +34<br>**M19**<br>3.9%, +16	**M26** (**34**)<br>3.6%, +16<br>**M24**<br>2.7%, +34<br>**M20** (**33**)<br>2.2%, +18					**M34**<br>8.0%, +16<br>**M33**<br>4.8%, +34<br>**M35**<br>3.9%, +32<br>**M36**<br>1.1%, +34<br>**M31**<br>0.9%, +18	**M45**<br>5.5%, +18<br>**M46**<br>1.5%, +34<br>**M44**<br>1.0%, +18<br>**M47**<br>0.4%, +16			**M58** (**4**)<br>38.4%, +16	**M63** (**1**)<br>44.7%, +16		
AtCYP714 A2				**M10**<br>3.1%, +34<br>**M6**<br>2.3%, +16<br>**M4**<br>1.7%, +18		**M5** (**29**)<br>84.8%, +16<br>**M8**<br>10.1%, +32	**M19**<br>93.1%, +16	**M26** (**34**)<br>50.1%,+16<br>**M21**<br>0.7%, +34	**M10**<br>35.6%, +16	**M27**<br>32.8%, +16	**M28**<br>2.6%, +16	**M29**<br>14.4%, +16	**M37**(**10**)<br>48.7%, +32<br>**M38**<br>2.8%, +16	**M48** (**11**)<br>15.1%, +16<br>**M49**<br>4.1%, +16						
AtCYP714 A1				**M6**<br>25.3%, +16<br>**M4**<br>14.2%, +18<br>**M10**<br>8.8%, +34	**M12**<br>3.8%, +16<br>**M13**<br>2.5%, +16	**M1** (**20**)<br>49.6%, +34<br>**M2**<br>11.3%, +34<br>**M3**<br>7.0%, +18<br>**M4**<br>5.5%, +34<br>**M5** (**29**)<br>4.9%, +16<br>**M6**<br>4.1%, +32<br>**M7**<br>1.6%, +32	**M15** (**32**)<br>16.2%, +32<br>**M16** (**31**)<br>10.0%, +18<br>**M17** (**26**)<br>7.2%, +34<br>**M18**<br>0.7%, +48	**M20** (**33**)<br>18.3%, +18<br>**M21**<br>2.1%, +34<br>**M22**<br>2.1%, +32<br>**M23** (**27**)<br>1.8%, +18<br>**M24**<br>1.2%, +34<br>**M25**<br>0.7%, +48	**M10**<br>7.3%, +16	**M27**<br>5.6%, +16		**M29**<br>16.4%, +16	**M30**<br>31.8%, +32<br>**M31**<br>1.1%, +18<br>**M32**<br>1.0%, +48<br>**M33**<br>0.9%, +34	**M43**<br>28.0%, +32<br>**M44**<br>0.3%, +18	**M51**<br>8.7%, +32	**M55**<br>4.4%, +32	**M57**<br>5.6%, +18	**M62**<br>4.2%, +18		
At2g36690													**M38**<br>0.5%, +16							
IrCYP706 V6																				
IrCYP706 V7				**M11**<br>13.2%, +16	**M14**<br>4.8%, +16	**M9** (**35**)<br>95.1%, +16														

Table S5. List of GC-MS electron impact fragmentation patterns compared to literature where available.

Compound	Data source	Characteristic ions, *m/z* (relative intensity)
**Gibberellins**		
**GA_{45} (38)** 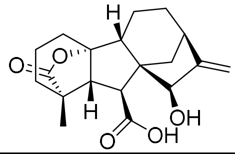	This work	418 ($M^{+\bullet}$) (100), 403 (16), 358 (36), 284 (16), 269 (19), 225 (23), 156 (48)
	Reference [23]	418 ($M^{+\bullet}$) (100), 403 (19), 358 (36), 284 (22), 269 (23), 225 (23), 156 (51)
**GA_{63} (39)** 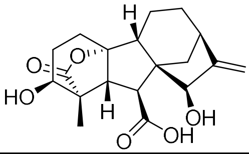	This work	506 ($M^{+\bullet}$) (100), 491 (48), 446 (44), 416 (23), 287 (38), 223 (41), 156 (67)
	Reference [23]	506 ($M^{+\bullet}$) (100), 491 (49), 446 (42), 416 (29), 287 (37), 223 (21), 156 (45)
**GA_{65} (41)** 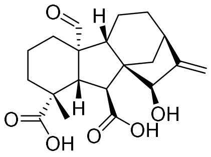	This work	462 ($M^{+\bullet}$) (8), 447 (23), 430 (21), 402 (70), 374 (58), 341 (30), 312 (50), 284 (100), 225 (79), 156 (95)
	Reference [23]	462 ($M^{+\bullet}$) (18), 447 (21), 430 (20), 402 (71), 374 (51), 341 (19), 312 (60), 284 (100), 225 (40), 156 (75)
	Reference [24]	462 ($M^{+\bullet}$) (9), 447 (24), 430 (17), 402 (67), 374 (52), 341 (20), 312 (51), 284 (100), 225 (46), 156 (100)
**GA_{64} (40)** 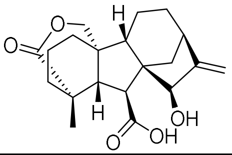	This work	432 ($M^{+\bullet}$) (38), 417 (24), 400 (16), 372 (17), 310 (26), 156 (100)
	Reference [23]	432 ($M^{+\bullet}$) (28), 417 (15), 400 (13), 372 (12), 310 (20), 156 (100)
**15β-hydroxy-GA_{14} (37)** 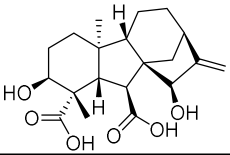	This work	536 ($M^{+\bullet}$) (5), 521 (22), 504 (49), 476 (18), 414 (15), 386 (32), 375 (19), 296 (20), 285 (19), 237 (27), 156 (100)
	Reference [24]	536 ($M^{+\bullet}$) (3), 521 (16), 504 (38), 476 (15), 414 (15), 387 (22), 375 (16), 296 (18), 285 (20), 237 (14), 156 (100)
**15β-hydroxy-GA_{12} (12)** 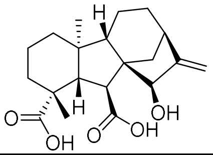	This work	448 ($M^{+\bullet}$) (2), 433 (15), 416 (86), 401 (6), 388 (56), 373 (10), 326 (18), 298 (27), 284 (10), 239 (28), 156 (100)
	Reference [24]	448 ($M^{+\bullet}$) (3), 433 (12), 416 (90), 401 (10), 388 (52), 373 (10), 326 (23), 298 (38), 284 (14), 239 (32), 156 (100)
**GA_{53} (M34)** 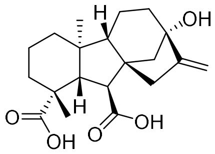	This work	448 ($M^{+\bullet}$) (33), 416 (21), 389 (26), 329 (16), 207 (100), 181 (89)
	Reference [23]	448 ($M^{+\bullet}$) (63), 416 (19), 389 (31), 329 (10), 207 (100), 181 (76)
**GA_{111} (M38)** 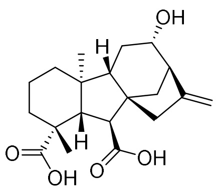	This work	448 ($M^{+\bullet}$) (4), 416 (49), 388 (46), 356 (18), 326 (25), 298 (100), 239 (76)
	Reference [23]	448 ($M^{+\bullet}$) (4), 416 (75), 388 (71), 356 (23), 326 (43), 298 (100), 239 (71)
**GA_{83} (M45)** 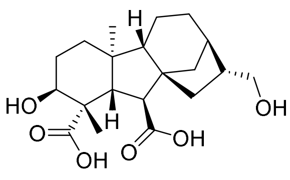	This work	523 ($M^{+\bullet}$–15) (15), 481 (10), 377 (100), 348 (56), 259 (62), 231 (90), 129 (67)
	Reference [23]	523 ($M^{+\bullet}$–15) (10), 481 (8), 377 (100), 348 (46), 259 (43), 231 (86), 129 (34)

**GA_{58} (M59)** 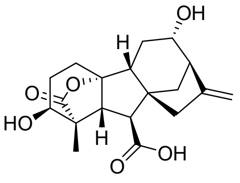	This work	506 ($M^{+\bullet}$) (20), 416 (48), 384 (70), 356 (100), 282 (85), 223 (96)
	Reference [23]	506 ($M^{+\bullet}$) (35), 416 (93), 384 (93), 356 (100), 282 (89), 223 (92)
**GA_{49} (M60)** 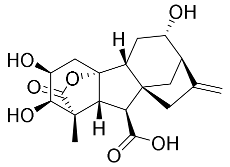	This work	594 ($M^{+\bullet}$) (52), 504 (12), 457 (13), 419 (14), 370 (27), 311 (31), 269 (22), 217 (55), 191 (71), 147 (100)
	Reference [23]	594 ($M^{+\bullet}$) (100), 504 (33), 457 (23), 419 (26), 370 (39), 311 (28), 269 (48), 217 (65), 191 (92), 147 (64)
**GA_{30} (M64)** 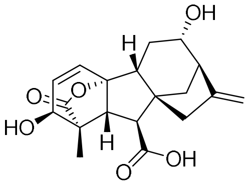	This work	504 ($M^{+\bullet}$) (6), 414 (8), 369 (18), 355 (15), 311 (24), 280 (28), 221 (100), 193 (59)
	Reference [23]	504 ($M^{+\bullet}$) (29), 414 (26), 369 (50), 355 (17), 311 (21), 280 (37), 221 (100), 193 (46)
**M30** 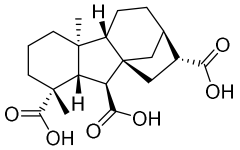	This work	374 (100), 359 (21), 342 (12), 314 (64), 287 (85), 259 (54), 227 (68), 199 (76)
	Reference [20]	374 (100), 359 (16), 342 (15), 314 (47), 287 (61), 259 (55), 227 (85), 199 (93)
**M33** 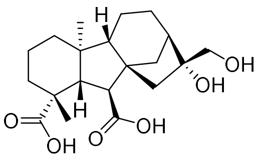	This work	523 ($M^{+\bullet}$–15) (1), 435 (100), 375 (65)
	Reference [25]	523 ($M^{+\bullet}$–15) (1), 435 (100), 375 (66)
**M41** 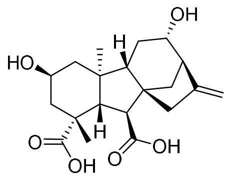	This work	521 ($M^{+\bullet}$–15) (3), 504 (45), 476 (84), 444 (23), 414 (26), 386 (92), 297 (100)
**M35** 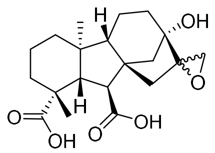	This work	464 ($M^{+\bullet}$) (15), 449 (41), 432 (19), 407 (16), 389 (100), 375 (36), 357 (42), 329 (45), 301 (58)
**M43** 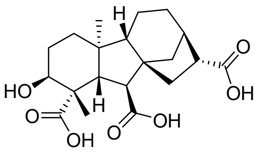	This work	479 ($M^{+\bullet}$–15) (10), 438 (31), 351 (28), 333 (100), 304 (55), 245 (67)
**M51** 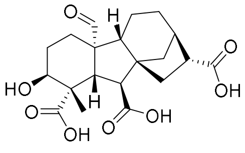	This work	508 ($M^{+\bullet}$) (2), 476 (6), 448 (14), 420 (17), 358 (31), 330 (100), 298 (31), 271 (80)

**M55** 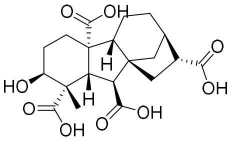	This work	523 ($M^{+\bullet}$–15) (9), 482 (25), 447 (9), 418 (14), 395 (32), 329 (16), 269 (21), 159 (31), 129 (100)
**M61**<br>Unknown	This work	534 ($M^{+\bullet}$) (67), 519 (29), 474 (25), 459 (42), 385 (100), 325 (69), 309 (89), 173 (83)
**M65**<br>Unknown	This work	504 ($M^{+\bullet}$) (6), 414 (14), 282 (37), 369 (100), 221 (75), 193 (89), 157 (87), 129 (89)
***ent*-Kaurene derivatives**		
**M11** 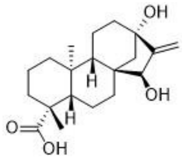	This work	492 ($M^{+\bullet}$) (24), 402 (20), 387 (18), 363 (36), 312 (7), 281 (66), 244 (45), 229 (100), 167 (12)
	Reference [24]	492 ($M^{+\bullet}$) (21), 402 (24), 387 (9), 363 (26), 312 (4), 281 (76), 244 (47), 229 (100), 167 (14)
**M19** 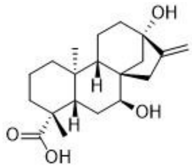	This work	492 ($M^{+\bullet}$) (67), 477 (5), 402 (22), 343 (14), 281 (100), 208 (24), 195 (27), 193 (40), 167 (29)
	Reference [20]	492 ($M^{+\bullet}$) (59), 477 (6), 402 (8), 343 (3), 281 (41), 208 (17), 195 (24), 193 (25), 167 (21), 73 (100)
**M24** 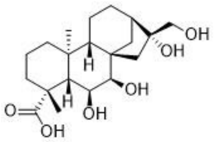	This work	567 (27), 477 (100), 417 (9), 387 (9), 269 (15), 209 (37), 191 (16), 147 (78), 129 (14), 117 (21), 103 (21)
	Reference [26]	567 (34), 477 (100), 417 (5), 387 (10), 269 (32), 209 (13), 191 (13), 147 (24), 129 (15), 117 (15), 103 (15)
**M4** 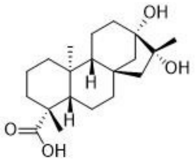	This work	494 ($M^{+\bullet}$) (5), 399 (6), 363 (100), 283 (21), 218 (20), 147 (41)
**M6** 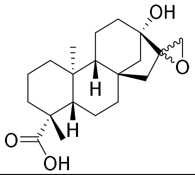	This work	420 ($M^{+\bullet}$) (21), 405 (100), 288 (28), 209 (68), 131 (74)
**M10** 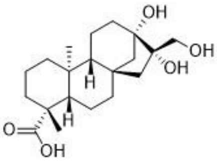	This work	567 ($M^{+\bullet}$–15) (1), 479 (11), 363 (100), 301 (6), 147 (26)
**M22** 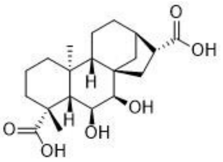	This work	523 ($M^{+\bullet}$–15) (90), 361 (19), 299 (17), 269 (100), 209 (72)
**M27** 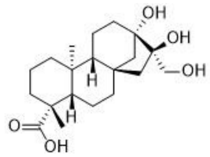	This work	479 (20), 363 (100), 195 (15), 147 (29)

Table S6. Identification of GA_{18} based on ^1H NMR comparison of isolated GA_{18} after methylation with literature data. The ^1H NMR spectrum is shown in **Figure S12**.

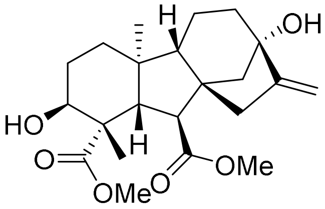

GA_{18} dimethyl ester

	Reference [12]<br>Measured in $CDCl_3$, 298K	Our data<br>Measured in $CDCl_3$, 298K
**Position**	**δ_H (J/Hz)<br>500 MHz**	**δ_H (J/Hz)<br>600 MHz**
20	0.70 s	0.70 s
18	1.17 s	1.17 s
5	2.37 d (12.4)	2.37 d (12.4)
6	3.34 d (12.4)	3.34 d (12.4)
CH_3 (O-Me)	3.69 s	3.69 s
CH_3 (O-Me)	3.70 s	3.70 s
3	4.14 t (2.9)	4.14 br s
17	4.93 t (2.3)<br>5.13 t (2.5)	4.93 br s<br>5.14 dd (2.7, 1.9)

Table S7. Comparison of amounts of gibberellins produced in this work and in previous studies.

Data is visualized in **Figure S21**. Other work was not included because no yields were provided[27] or no reasonable comparison was possible.[28]

Method	Compound	Titer [mg/L]	Amount isolated / synthesized [mg]	Space-time yield [mg L^{-1} d^{-1}]	Specific yield [mg g^{-1} biomass]	Overall yield / steps	Comment	Ref.
**This work**								
Original *A. oryzae* platform	GA_{12} (**5**)	6	24	1.2				This work
Original *A. oryzae* platform	GA_{14} (**6**)	25	75	5.0				This work
Original *A. oryzae* platform	GA_{9} (**7**)	2	2	0.4				This work
Original *A. oryzae* platform	GA_{13} (**8**)	4	12	0.8				This work
Original *A. oryzae* platform	GA_{36} (**9**)	2	6	0.4				This work
Chemoenzymatic synthesis	GA_{123} (**10**)		1.2		2.9E-1	13.7% 1 step		This work
Chemoenzymatic synthesis	GA_{74} (**11**)		1.5		2.8E-1	5.7% 1 step		This work
Chemoenzymatic synthesis	**31**		1.1		2.5E-1	3.7% 1 step		This work
Chemoenzymatic synthesis	**32**		1.6		3.6E-1	5.2% 1 step		This work
Chemoenzymatic synthesis	**26**		0.7		1.6E-1	2.3% 1 step		This work
Chemoenzymatic synthesis	**33**		1.9		3.1E-1	3.6% 1 step		This work
Chemoenzymatic synthesis	**34**		2.2		5.4E-1	14.0% 1 step		This work
A. oryzae with *IrCYP706V7*	15β-Hydroxy-GA_{12} (**12**)	1.5	3	0.3				This work
**Microbial fermentation**								
Fusarium fujikuroi	GA_{3} (**1**)	3900		490				29
Fusarium fujikuroi mutant	GA_{4} (**2**)	599		86				30
Bacteria	GA_{9} (**7**)	0.0016		0.0008				31
Yeast metabolic engineering	GA_{3} (**1**)	12.8		4.3				32
Yeast metabolic engineering	GA_{4} (**2**)	16.4		5.5				32
Yeast metabolic engineering	GA_{9} (**7**)	4.7		1.6				32
Yeast metabolic engineering	GA_{7} (**3**)	0.8		0.3				32
E. coli biotransformation	GA_{1} (**4**)	71.2		36		36.1% 1 step	Biotransformation of 180 mg/L steviol	33
E. coli biotransformation	GA_{53} (**M34**)	94.8		24		48.1% 1 step	Biotransformation of 180 mg/L steviol	33
E. coli biotransformation	GA_{20}	82.9		21		44.1% 1 step	Biotransformation of 180 mg/L steviol	33
**Isolation from plants**								
Plant isolation	GA_{1} (**4**)		2		2.3E-5		Isolated from 87 kg of bean seeds	34
Plant isolation	GA_{19}		14		3.2E-7		Isolated from 44 t of bamboo shoots	35
Plant isolation	GA_{18}		35		5.8E-4		Isolated from 60 kg of lupin seeds	36
Plant isolation	GA_{32}		38		1.1E-3		Isolated from 35 kg of peach seeds	37
**(Semi)Synthesis**								
Synthesis	GA_{4} (**2**)		not stated			1.8% 15 steps	Racemic total synthesis	38
Synthesis	GA_{6}		65			10.3% 5 steps	Semisynthesis from GA_{5} methyl ester	39
Synthesis	GA_{18} methyl ester		2.1			1.3% 11 steps	Semisynthesis from andrographolide	12

Table S8. List of primers used in this study

Overhangs for cloning are in bold and underlined.

Genes not listed here were ordered as synthetic gene fragments with overhangs for cloning as listed in **Table S9** and directly used without prior PCR amplification.

**Name**	**Sequence**	**Purpose**
AstHMGR-Peno-F	**CGACTGACCAATTCCGCAGCTCGTCAAAGG**ATGGCGCCCGAGAAAATGCC	For yeast homologous recombination cloning of *AstHMGR* into pTYGS-*argB* under P_{eno} promotor
AstHMGR-Peno-R	**CTGGTAGACGTCATATAATCATACGGCGCG**TCAGCAGGCGATCTTGGACA	
ko-Padh-F	**TCTTTCAACACAAGATCCCAAAGTCAAAGG**ATGCCGCTAATGGACGTTCA	For yeast homologous recombination cloning of *ko* into pTYGS-*adeA* under P_{adh} promotor
ko-Padh-R	**CTATGCGTTATGAACATGTTCCCTGGCGCG**TCATTCATCTCTCAGTGATC	
AstHMGR-PamyB-F	**AACAATAAACCCCACAGCAAGCTCCGAATT**ATGGCGCCCGAGAAAATGCC	For yeast homologous recombination cloning of *AstHMGR* into pTYGS-*argB* under P_{amyB} promotor
AstHMGR-PamyB-R	**CACCCTTCACGAGCTACTACAGATCCCCGG**TCAGCAGGCGATCTTGGACA	
AtCPR1-Padh-F	**TCTTTCAACACAAGATCCCAAAGTCAAAGG**ATGACTTCTGCTTTGTATGC	For yeast homologous recombination cloning of *AtCPR1* into pTYGS-*adeA* under P_{adh} promotor
AtCPR1-Padh-R	**CTATGCGTTATGAACATGTTCCCTGGCGCG**TCACCAGACATCTCTGAG	
IrCYP706V7-PgpdA-F	**CAGCTACCCCGCTTGAGCAGACATCACCGG**ATGAAAGCGGCGTTACTAA	For yeast homologous recombination cloning of *IrCYP706V7* into pTYGS-*adeA* under P_{gpdA} promotor
IrCYP706V7-PgpdA-R	**ATGTCCATATCATCAATCATGACCGGCGCG**CTAGTTATATAGCTCCGGAT	
AtCYP714A2-PgpdA-F	**CAGCTACCCCGCTTGAGCAGACATCACCGG**ATGGAGAGTTTGGTTGTTCATACGG	For yeast homologous recombination cloning of *AtCYP714A2* into pTYGS-*adeA* under P_{gpdA} promotor
AtCYP714A2-PgpdA-R	**ATGTCCATATCATCAATCATGACCGGCGCG**TCAAACAACCCTAATGACAACACCATG	
MdDOX-Co-PgpdA F	**CAGCTACCCCGCTTGAGCAGACATCACCGG**ATGGAGACATTAGATCAGAA	For yeast homologous recombination cloning of *MdDOX-Co* into pTYGS-*adeA* under P_{gpdA} promotor
MdDOX-Co-PgpdA R	**ATGTCCATATCATCAATCATGACCGGCGCG**TTAGCTACTCGAGAGGCT	
Bjks-PgpdA-F	**CAGCTACCCCGCTTGAGCAGACATCACCGG**ATGATCCAGACCGAACGTGCAG	For yeast homologous recombination cloning of *Bjks* into pTYGS-*sC* under P_{gpdA} promotor
Bjks -PgpdA-R	**ATGTCCATATCATCAATCATGACCGGCGCG**TCAAGCCGGTGCACGCTGA	
ggs2-pEAQ-F	**GCCCAAATTCGCGACCGG**ATGGCTGAACAACAGATCTC	For In-Fusion cloning of *ggs2* into pEAQ-HT
ggs2-pEAQ-R	**CAGAGTTAAAGGCCTCGA**CTAGGTTTCCAGCTTCAGG	
cps/ks pEAQ-F	**GCCCAAATTCGCGACCGG**ATGCCTGGCAAAATCGAGA	For In-Fusion cloning of *cps/ks* into pEAQ-HT
cps/ks pEAQ-R	**CAGAGTTAAAGGCCTCGA**TCACTTCATGCTGCTTGAAA	
AtGA20ox1-pEAQ-F	**GCCCAAATTCGCGACCGG**ATGGCCGTAAGTTTCGTAAC	For In-Fusion cloning of *AtGA20ox1* into pEAQ-HT
AtGA20ox1-pEAQ-R	**CAGAGTTAAAGGCCTCGA**TTAGATGGGTTTGGTGAGC	
AtGA3ox1-pHREAC-F	**CACCACAGGTCTCGAAAA**ATGCCTGCTATGTTAACAG	For Golden Gate cloning of *AtGA3ox1* into pHREAC
AtGA3ox1-pHREAC-R	**CACCACAGGTCTCGAGCG**TCATTCTTCTCTGTGATTTCTA	

Table S9. List of gene sequences used in this study.

Overhangs for cloning are in bold and underlined.

Gene name and information	Nucleotide Sequence
ggs2<br>• from *Fusarium fujikuroi*<br>• overhang for yeast homologous recombination cloning into pTYGS-*argB* under P_{adh} promotor	**TCTTTCAACACAAGATCCCAAAGTCAAAGG**ATGGCTGAACAACAGATCTCCAACCTTCTTTCAATGTTTGATGCTTCTCACGCAAGCCAGAAGTTGGAGATTACGGTTCA<br>GATGATGGATACCTACCATTACAGAGAAACTCCTCCAGACTCTTCCTCTTCAGAAGGCGGTTCCTTATCTCGCTATGATGAGCGACGGGTCTCCCTTCCGCTCTCTCACA<br>ATGCAGCCTCCCCAGACATAGTCTCCCAGTTATGCTTCTCAACAGCTATGAGCTCGGAGCTCAATCACAGGTGGAAGTCACAGCGCCTCAAGGTTGCTGACTCTCCCTAC<br>AACTACATCCTGACTCTTCCATCTAAAGGTATTCGTGGGGCTTTCATTGACTCACTGAATGTCTGGCTCGAGGTCCCCGAAGACGAGACCTCGGTGATCAAAGAGGTGAT<br>TGGCATGCTCCACAACTCGTCTCTCATAATCGATGACTTCCAAGACAACTCCCCACTTCGGCGGGGCAAGCCATCTACACATACTGTCTTCGGTCCAGCACAAGCAATCA<br>ACACAGCAACATATGTCATCGTCAAGGCCATCGAGAAAATACAGGATATCGTCGGTCACGATGCATTGGCAGATGTAACTGGCACTATAACCACAATCTTCCAGGGTCAG<br>GCAATGGATCTGTGGTGGACTGCTAATGCCATTGTTCCGTCTATCCAAGAATATCTCCTGATGGTCAATGACAAGACTGGTGCCCTGTTCAGGTTATCGCTTGAACTACT<br>GGCGCTGAACTCTGAAGCATCCATCAGTGACAGCGCGCTTGAATCTCTCAGCAGCGCTGTCTCACTGCTCGGGCAGTATTTCCAGATAAGAGATGATTACATGAATCTCA<br>TTGACAACAAGTATACTGATCAGAAAGGATTTTGCGAGGATCTGGACGAGGGGAAATACTCGTTGACTCTAATCCATGCTCTGCAGACCGACTCCAGCGACCTTCTCACC<br>AACATCTTATCGATGAGAAGAGTCCAAGGAAAACTTACGGCGCAGCAAAAGATGCTGGTTTTGGAAGTGATGAAAACAAACGGCAGCTTAGACTGGACGAGCAAACTCCT<br>TGGCATGCTGCACACAAGAGTTGTAGCCGAGATTGAGAGCCTGGAAGTTAGCACGAAGAGGGATAACCATGCATTGAGGGCTTTAGTGGAACGCCTGAAGCTGGAAACCT<br>AG**CGCGCCAGGGAACATGTTCATAACGCATAG**
cps/ks<br>• from *Fusarium fujikuroi*<br>• overhang for yeast homologous recombination cloning into pTYGS-*argB* under P_{gpdA} promotor	**CAGCTACCCCGCTTGAGCAGACATCACCGG**ATGCCTGGCAAAATCGAGAACGGCACCCCCAAAGACCTCAAGACAGGAAATGACTTTGTATCAGCAGCAAAGTCGCTGCT<br>GGACCGCGCCTTCAAGAGCCATCATAGCTACTATGGTCTATGTTCCACCAGCTGTCAGGTATATGACACTGCTTGGGTGGCCATGATACCCAAGACGAGAGACAATGTCA<br>AGCAATGGCTGTTTCCTGAGTGCTTCCACTATCTTCTGAAGACTCAGGCCGCTGATGGTAGTTGGGGTAGTCTCCCCACGACCCAGACTGCTGGTATTCTAGACACTGCT<br>TCAGCTGTGCTTGCACTCTTATGTCACGCACAAGAGCCTCTGCAGATACTGGACGTCTCGCCAGACGAGATGGGTCTCAGGATCGAGCACGGGGTAACTTCTTTGAAGCG<br>ACAGCTAGCTGTTTGGAACGATGTTGAAGATACCAATCACATTGGTGTCGAGTTTATTATTCCAGCTTTACTTTCCATGCTTGAGAAAGAGCTCGACGTGCCATCTTTTG<br>AGTTTCCTTGCAGAAGTATCTTGGAGAGGATGCACGGAGAGAAGCTCGGCCATTTCGATTTGGAGCAAGTATATGGCAAGCCGTCTTCACTACTTCACTCCCTTGAAGCC<br>TTTCTTGGAAAGCTTGACTTTGATCGCCTTTCTCACCATCTTTATCATGGCTCTATGATGGCTTCACCTTCTTCCACTGCAGCCTACCTCATTGGTGCAACCAAGTGGGA<br>CGACGAGGCTGAAGATTACCTGAGGCACGTTATGAGAAATGGTGCTGGTCACGGGAATGGTGGCATCTCAGGCACCTTTCCTACAACACATTTTGAGTGTAGCTGGATCA<br>TAGCGACACTCCTGAAGGGTGGCTTCACTTTGAAACAAATCGATGGTGATGGCTTGCGCGGCTTGTCAACTATACTTCTCGAAGCGTTGAGGGACGAGAATGGAGTAATT<br>GGCTTTGCACCCCGTACTGCTGATGTTGATGACACTGCAAAAGCTCTTCTGGCATTGAGCCTTGTCAATCAACCTGTCAGCCCAGATATCATGATCAAGGTCTTTGAAGG<br>AAAAGATCACTTCACCACGTTCGGATCTGAACGTGATCCCAGCTTGACTTCCAACCTCCATGTGCTGCTGAGTCTCCTGAAGCAGTCCAATCTATCGCAATACCACCCAC<br>AAATCCTGAAGACCACGTTGTTCACTTGCCGATGGTGGTGGGGCAGCGACCATTGCGTCAAAGATAAATGGAATTTGAGTCATCTATACCCAACCATGCTTCTGGTGGAA<br>GCTTTCACCGAGGTACTTCATCTCATTGATGGTGGCGAGCTTTCTAGTCTCTTTGACGAGAGCTTCAAGTGCAAGATCGGCCTGTCAATCTTCCAGGCTGTACTACGTAT<br>CATCCTCACCCAAGATAACGACGGATCTTGGAGAGGATATCGCGAGCAAACATGCTACGCCATTCTAGCTCTTGTACAGGCACGCCATGTATGTTTCTTCACTCACATGG<br>TGGACAGGCTACAGAGTTGCGTCGACCGTGGCTTTTCGTGGCTCAAGTCCTGCAGCTTTCATTCTCAGGACCTCACTTGGACGTCAAAGACAGCATATGAGGTTGGGTTT<br>GTTGCTGAAGCGTACAAGCTAGCAGCCCTTCAGTCAGCAAGCCTAGAGGTGCCCGCCGCTACTATTGGCCACAGCGTCACCTCGGCGGTTCCATCATCGGACTTGGAGAA<br>GTACATGAGGCTTGTGCGCAAGACTGCTTTGTTCTCGCCACTGGACGAGTGGGGACTCATGGCGTCAATCATTGAGTCCTCGTTCTTCGTGCCCCTTCTACAAGCTCAGC<br>GCGTTGAGATTTATCCGAGGGACAACATCAAGGTCGACGAGGACAAATACCTGAGCATCATTCCTTTTACTTGGGTCGGATGCAACAACAGATCCCGCACATTTGCATCT<br>AACAGATGGCTCTACGATATGATGTACCTGTCCCTCCTGGGCTACCAGACCGATGAGTACATGGAAGCTGTCGCTGGGCCGGTATTCGGTGATGTCTCTCTGCTTCACCA<br>GACCATCGACAAGGTCATAGACAACACAATGGGAAACTTAGCCAGGGCCAATGGTACCGTGCACAGTGGCAATGGTCATCAGCATGAGTCACCCAACATTGGACAAGTTG<br>AGGATACTCTCACTCGCTTCACCAACAGCGTGCTCAACCACAAAGATGTTCTGAATTCAAGCTCTTCTGACCAAGACACTCTTCGCCGAGAATTCAGAACGTTCATGCAT<br>GCCCATATCACACAGATAGAGGACAACAGTCGCTTCTCCAAGCAGGCCTCCAGCGACGCTTTCTCTTCCCCAGAACAATCGTACTTCCAATGGGTCAACAGTACAGGAGG<br>CAGTCACGTCGCCTGCGCGTACTCATTCGCTTTCTCCAACTGCCTAATGTCAGCGAATCTGCTCCAAGGCAAGGACGCATTTCCCTCCGGCACTCAGAAATATCTCATTT<br>CATCCGTTATGCGTCACGCTACAAATATGTGTCGTATGTACAACGACTTTGGGTCCATAGCTCGCGATAACGCTGAGCGGAATGTGAACTCGATCCACTTCCCCGAGTTC<br>ACGTTATGTAACGGAACGTCACAGAATCTCGATGAAAGGAAAGAGAGACTTTTAAAGATAGCTACGTATGAGCAAGGTTATCTTGATCGTGCTCTCGAAGCTCTGGAACG<br>ACAGTCTCGTGATGATGCAGGTGATCGTGCTGGGTCTAAGGATATGAGGAAGCTCAAGATTGTAAAACTGTTCTGTGATGTCACCGACTTGTATGATCAGCTTTACGTGA<br>TCAAGGACCTTTCAAGCAGCATGAAGTGA**CGCGCCGGTCATGATTGATGATATGGACAT**
ko<br>• from *Fusarium fujikuroi*<br>• overhang for yeast homologous recombination cloning into pTYGS-*argB* under P_{eno} promotor	**CGACTGACCAATTCCGCAGCTCGTCAAAGG**ATGCCGCTAATGGACGTTCACTGGCTGATCTACGTGGCCTTTGGCGCTTGGTTATGCTCTTATGTCATCCATGTCCTATC<br>GTCCTCTTCTACAGTCAAAGTGCCCGTCGTAGGCTACCGCAGCGTCTTTGAGCCTACATGGCTTCTCCGTTTGCGCTTTGTTTGGGAAGGGGGATCTATCATCGGCCAAG<br>GCTACAACAAATTTAAAGACTCTATCTTCCAGGTGCGAAAGCTTGGTACCGATATCGTCATCATCCCGCCAAACTACATCGATGAGGTCAGAAAGCTGTCCCAAGACAAG<br>ACTCGCTCGGTCGAGCCCTTCATCAATGACTTTGCGGGACAGTATACACGGGGCATGGTCTTTCTGCAAAGTGATTTGCAGAACCGTGTGATTCAGCAGCGGTTGACGCC<br>AAAACTCGTATCGTTGACAAAGGTAATGAAGGAGGAGCTTGACTATGCCTTGACCAAAGAGATGCCTGACATGAAGAATGATGAATGGGTTGAAGTCGACATTTCTTCCA<br>TCATGGTCAGGCTCATATCACGCATCTCAGCCAGAGTGTTTCTCGGTCCAGAGCACTGCCGCAACCAAGAATGGTTGACGACCACTGCAGAGTACAGCGAGAGCCTGTTC<br>ATAACTGGCTTTATTCTCCGCGTTGTCCCCCATATTCTAAGACCATTCATAGCCCCGCTGCTACCCTCCTACAGAACACTACTTCGCAACGTCTCGTCAGGTCGAAGAGT<br>TATTGGAGACATCATTCGCTCCCAGCAAGGTGATGGCAACGAGGACATCCTGTCATGGATGAGGGATGCTGCGACAGGGGAAGAAAGCAAATTGACAACATTGCCCAGC<br>GGATGCTTATCCTGAGTCTCGCGTCTATTCACACTACGGCAATGACGATGACGCATGCTATGTATGACTTATGTGCTTGCCCTGAGTACATAGAGCCTCTTAGAGATGAG<br>GTCAAAAGTGTCGTTGGCGCTAGTGGTTGGGACAAGACGGCGTTGAATCGATTCCACAAACTCGACAGCTTTCTCAAAGAGTCACAACGCTTCAACCCCGTGTTCCTCTT<br>AACGTTCAATCGCATTTATCACCAATCCATGACACTCTCAGATGGCACCAACATCCCATCAGGCACTCGCATCGCGGTTCCCTCTCACGCGATGCTTCAGGACTCAGCGC<br>ATGTCCCAGGCCCGACGCCACCAACCGAGTTTGATGGATTTAGATACTCAAAGATTCGCTCAGACTCAAACTATGCACAGAAATATCTCTTCTCCATGACTGATTCTAGT<br>AACATGGCGTTTGGGTATGGGAAATACGCCTGCCCAGGGCGGTTCTATGCATCTAATGAGATGAAGCTGACTTTGGCGATACTCCTTTTACAATTTGAGTTCAAGTTGCC<br>AGATGGGAAAGGAAGACCACGAAATATCACTATTGATAGTGACATGATACCTGATCCGAGAGCTAGGCTGTGCGTTAGGAAGCGATCACTGAGAGATGAATGA**CGCGCCG<br>TATGATTATATGACGTCTACCAG**
P450-1<br>• from *Fusarium fujikuroi*<br>• overhang for yeast homologous recombination cloning into pTYGS-*adeA* under P_{gpdA} promotor	**CAGCTACCCCGCTTGAGCAGACATCACCGG**ATGGCGAACCATTCTTCTTCTTACTACCATGAGTTCTACAAGGACCACTCTCATACCGTCTTGACGTTGATGAGTGAGAA<br>ACCGGTCATATTACCATCTCTCATCTTGGGAACTTGTGCCGTTCTGTTGTGCATTCAATGGCTTAAACCTCAGCCGTTGATCATGGTGAACGGTAGAAAGTTTGGCGAAC<br>TGTCCAACGTCCGCGCCAAGAGGGACTTCACCTTTGGGGCTCGGCAACTCCTAGAAAAAGGCCTTAAGATGAGCCCCGACAAGCCATTTCGAATCATGGGCGATGTCGGG<br>GAGCTGCACATCCTGCCGCCAAAGTACGCCTACGAGGTTCGCAATAATGAAAAGCTCAGCTTCACCATGGCAGCTTTCAAGTGGTTTTATGCTCATCTTCCTGGGTTTGA<br>GGGCTTCAGAGAGGGTACCAACGAGAGTCACATCATGAAGCTGGTTGCGCGACATCAATTGACTCATCAGTTGACTCTGGTGACGGGAGCAGTCTCTGAAGAGTGCGCCT<br>TGGTGTTGAAAGATGTGTACACAGATAGTCCAGAATGGCACGACATCACCGCCAAAGACGCAAACATGAAACTCATGGCCCGCATCACCTCTCGAGTATTCCTCGGTAAG<br>GAGATGTGTCGAAACCCCCAGTGGCTTAGAATTACATCTACCTACGCCGTCATCGCCTTCCGTGCAGTAGAAGAACTGCGTCTCTGGCCATCGTGGCTCCGGCCTGTAGT<br>CCAGTGGTTCATGCCGCACTGCACACAGTCCCGAGCCCTAGTCCAAGAAGCAAGAGACCTTATCAACCCGCTTCTTGAGCGGCGACGCGAAGAGAAAGCTGAGGCTGAGA<br>GAACTGGAGAGAAGGTCACCTACAACGATGCTGTTGAGTGGTTGGATGACCTTGCGCGTGAAAAGGGTGTTGGATATGACCCCGCTTGTGCTCAGCTCTCGCTCTCAGTG<br>GCTGCTTTACATAGTACTACTGATTTCTTTACCCAGGTCATGTTTGACATTGCGCAGAATCCGGAGCTTATCGAGCCTTTGCGCGAGGAGATCATAGCTGTTTTGGGGAA<br>GCAGGGATGGTCAAAGAACTCGCTGTACAACCTGAAGCTCATGGACAGCGTCCTGAAAGAGAGTCAACGACTGAAGCCCATAGCCATTGCAAGCATGCGCCGATTCACAA<br>CGCACAACGTCAAGTTATCCGATGGCGTCATCCTACCCAAGAACAAACTCACGCTTGTATCAGCCCATCAACACTGGGATCCAGAGTACTACAAGGATCCCCTGAAGTTC<br>GATGGATACCGTTTCTTCAACATGCGGCGCGAGCCAGGCAAAGAGAGCAAAGCACAGCTCGTGAGTGCCACTCCGGATCACATGGGCTTCGGGTACGGGCTTCATGCCTG<br>TCCTGGACGGTTCTTTGCGTCTGAGGAGATCAAGATTGCACTGTCTCACATTCTGCTCAAGTACGACTTCAAGCCTGTTGAGGGGTCGAGCATGGAGCCGAGGAAGTATG<br>GCCTCAACATGAATGCCAACCCAACTGCAAAGTTATCTGTTCGTCGCAGAAAGGAGGAGATTGCGATTTGA**CGCGCCGGTCATGATTGATGATATGGACAT**
P450-2<br>• from *Fusarium fujikuroi*<br>• overhang for yeast homologous recombination cloning into pTYGS-*adeA* under P_{eno} promotor	**CGACTGACCAATTCCGCAGCTCGTCAAAGG**ATGAGCATCTTCAATATGATCACCAGCTATGCGGGTAGCCAACTTTTGCCCTTCTACATCGCCATATTCGTCTTTACTTT<br>AGTCCCATGGGCAATCCGCTTCTCTTGGCTAGAATTGCGCAAGGGCTCAGTGGTGCCACTTGCGAACCCGCCCGACTCACTGTTCGGCACCGGTAAAACCAGGAGGAGTT<br>TTGTCAAGCTTAGTAGAGAAATTCTCGCTAAAGCGAGGAGCTTGTTCCCTAATGAGCCATTTCGCTTGATTACGGACTGGGGTGAGGTTCTCATTCTCCCCCCAGACTTT<br>GCAGATGAGATTAGAAATGATCCGAGACTGAGCTTCTCCAAGGCGGCGATGCAGGATAATCATGCTGGAATACCTGGCTTTGAGACTGTTGCCCTGGTGGGTCGTGAAGA<br>CCAACTTATTCAGAAGGTGGCCCGAAAGCAGTTGACCAAGCATCTTTCCGCTGTCATAGAGCCACTATCTAGAGAGTCCACCCTCGCAGTGTCGCTCAACTTTGGAGAGA<br>CAACAGAATGGCGAGCGATACGCCTCAAGCCCGCAATTCTAGACATCATCGCCCGCATCTCGTCCAGAATCTATCTCGGCGACCAACTATGCCGCAACGAAGCTTGGCTG<br>AAGATCACAAAGACATACACCACCAACTTCTACACTGCATCTACCAACCTCCGAATGTTTCCTCGATCGATCCGTCCTCTCGCCCACTGGTTCCTCCCCGAATGCAGAAA<br>GCTTCGACAGGAGCGCAAGGATGCAATCGGTATTATTACGCCACTGATTGAGCGCCGCCGTGAGCTTCGAAGAGCTGCGATCGCAGCTGGTCAGCCTCTGCCTGTGTTCC<br>ACGACGCTATTGACTGGTCGGAACAGGAGGCAGAAGCTGCAGGCACAGGGGCCTCGTTTGACCCCGTGATCTTCCAGCTTACGCTCTCTCTTCTGGCAATTCATACGACG<br>TATGATCTCCTCCAGCAAACGATGATTGACCTTGGTCGCCACCCAGAGTATATCGAGCCTCTTAGACAGGAAGTTGTTCAACTTCTTCGTGAAGAAGGTTGGAAGAAAAC<br>AACGCTTTTCAAGATGAAGCTCCTTGACAGTGCTATCAAAGAGTCTCAGCGAATGAAGCCTGGAAGCATAGTTACCATGCGTCGCTACGTAACCGAAGACATCACCCTCT<br>CTAGCGGCCTGACCCTCAAAAAAGGACCCGCCTCAACGTTGACAACAGACGCCTCGACGATCCCAAAATCTACGATAACCCCGAGGTTTACAATCCTTATCGCTTCTAC<br>GACATGCGCTCCGAAGCCGGGAAAGACCATGGGGCACAGCTAGTATCAACTGGCTCAAACCATATGGGCTTCGGCCACGGTCAGCACTCATGCCCAGGGCGTTTCTTCGC<br>TGCGAATGAGATCAAAGTAGCGCTATGCCACATCTTGGTCAAGTATGATTGGAAGCTGTGCCCTGACACGGAGACCAAGCCTGATACCAGGGGCATGATTGCCAAGTCCA<br>GCCCTGTCACGGACATCTTGATCAAGCGTCGGGAGTCAGTTGAGTTGGATTTGGAAGCAATTTGA**CGCGCCGTATGATTATATGACGTCTACCAG**
P450-3<br>• from *Fusarium fujikuroi*<br>• overhang for yeast homologous recombination cloning into pTYGS-*sC* under P_{gpdA} promotor	**CAGCTACCCCGCTTGAGCAGACATCACCGG**ATGAGTAATTTTGTGACGTTGATTGAGCCTCTGGAGCTCACTGGCTCACGGGTTTTGCGTATCGCGGTGGCGTTCGCCGC<br>CCTTTGCGGCGCTACAGGACTCTTAGCATTCTCTTGGTGGATCTACAAGCAGTCTTCGAGTAAACCGACCTTGCCATACCCTGTAGTAGGCGATACCCACGCACAATCGT<br>TGGAGAAGAACCTCATCAAGGGGATGCAGCAGTATCGTGACTCGCCCTTCTTCTTGGCTGGATCACGCCCACCACTGCTCATCCTACCAATGTCCGTCTTTCATGAAATA<br>CACAACATGCCAAATGAATATATTTCCATCATTGTGGAGCATGAAGACAAGTTTCAGGGCAAGTACACCCACATTACCACCATCAGACCTGAGATTCCCGCCACTATTCG<br>CCAGGACTTGACTCGAAATATGCCCAACATCATACTGGAATTACAGGATGAGTTAACATATGCTTCCGATCAGTGGCCTAGAACAAGCAAATGGTCATCTGTATCTTTAT<br>ATGACATGATGCTAAGGACCGTGGCGCTTTTGAGTGGCAGAGCGTTTGTTGGTCTGCCCTTGTGTCGCGATGAAGGCTGGCTACAAGCTTCCATAGGCTACACTGTTCAA<br>TGTGTCTCAATTCGCGATCAGCTCTTTACCTGGAGTCCGGTGCTGAGACCAATCATTGGGCCGTTTTTACCCAGCGTCCGTTCGGTGCGAAGACATCTACGCTTCGCAGC<br>AGAGATAATGGCTCCTCTGATCTCCCAAGCTTTACAAGATGAAAAGCAACATCGAGCAGATACACTTCTAGCGGATCAGACGGAAGGGCGAGGTACATTTATCTCATGGC<br>TTCTTCGGCACTTACCTGAGGAGCTCAGAACCCCAGAGCAGGTCGGGCTAGATCAAATGCTGGTCTCGTTCGCCGCAATCCACACTACGACCATGGCCCTCACAAAGGTA<br>GTATGGGAGCTTGTTAAGCGGCCGGAGTACATCGAGCCGTTGAGAACCGAGATGCAAGATGTCTTTGGACCTGATGCCGTATCACCAGACATATGCATCAACAAAGAGGC<br>TCTTTCTAGGCTCCACAAGTTGGATAGCTTCATTAGAGAGGTCCAGCGCTGGTGCCCTTCAACATTTGTGACTCCAAGCAGACGCGTCATGAAGTCAATGACACTATCAA<br>ACGGTATCAAGCTACAGAGAGGAACTTCTATAGCATTCCCCGCTCACGCAATCCACATGTCGGAGGAAACGCCAACATTCTCGCCAGACTTTAGCTCTGACTTCGAGAAC<br>CCGTCTCCTCGAATCTTTGACGGGTTCCGGTACTTGAATCTCCGATCTATCAAAGGACAGGGGTCACAACACCAGGCAGCCACTACAGGTCCTGACTATCTGATCTTCAA<br>CCATGGAAAGCATGCATGCCCTGGGAGGTTCTTTGCCATATCTGAGATCAAGATGATACTCATAGAACTATTGGCAAAGTATGATTTTAGACTTGAGGATGGAAAGCCAG<br>GGCCTGAGTTGATGCGTGTTGGGACGGAGACTCGGCTGGATACCAAAGCTGGTCTTGAGATGCGAAGGAGATGA**CGCGCCGGTCATGATTGATGATATGGACAT**

des<br>• from *Fusarium fujikuroi*<br>• overhang for yeast homologous recombination cloning into pTYGS-*sC* under P_{adh} promotor	**TCTTTCAACACAAGATCCCAAAGTCAAAGG**ATGCCTCATAAAGATAATCTTCTTGAATCGCCAGTGGGCAAGAGTGTCACTGCTACTATAGCCTACCATAGCGGACCGGC<br>TCTTCCAACCTCCCCGATCGCTGGTGTCACTACGCTCCAAGACTGCACTCAGCAGGCCGTAGCAGTGACTGATATCCGCCCTTCAGTCTCGTCCTTTACCCTAGATGGTA<br>ACGGCTTCCAGGTTGTCAAACATACATCGGCGGTAGGCTCTCCGCCGTATGATCACTCGTCGTGGACAGATCCAGTCGTTCGCAAGGAAGTGTATGACCCCGAAATCATT<br>GAACTGGCAAAGTCTCTCACTGGAGCCAAGAAGGTCATGATTCTACTTGCTTCGTCTCGGAATGTTCCCTTCAAGGAGCCAGAGCTCGCCCCTCCTTATCCCATGCCTGG<br>CAAATCAAGCAGCGGCAGCAAGGAAAGGGAAGCCATCCCAGCTAATGAGCTCCCTACTACAAGGGCAAAAGGTTTCCAAAAAGGCGAAGAGGAAGGCCCAGTACGAAAGC<br>CTCATAAGGACTGGGGTCCATCCGGTGCGTGGAACACTCTCCGGAACTGGAGCCAAGAGCTCATTGATGAGGCTGGCGATATCATCAAGGCTGGCGATGAGGCTGCAAAG<br>CTGCCAGGGGGCAGAGCAAAGAACTACCAAGGCAGACGATGGGCCCTGTATACTACCTGGCGTCCACTGAAAACTGTCAAGCGGGATCCCATGGCCTATGTAGACTACTG<br>GACAGCTGATGAGGAAGATGGCGTGAGCTTCTGGCGTAACCCGCCAGGGGTGCATGGGACATTTGAGTCGGATGTACTACTTACCAAGGCTAATCCAAAGCATAAGTGGT<br>ACTGGATCAGTGACCAGACTCCGGATGAGGTTCTCCTCATGAAGATCATGGACACCGAGAGTGAGAAGGACGGGAGTGAAATAGCGGGAGGGGTTCACCACTGTTCATTT<br>CATCTGCCGGGAACTGAGAAGGAGGAAGTGAGAGAGAGCATTGAGACCAAGTTCATTGCATTCTGGTAG**CGCGCCAGGGAACATGTTCATAACGCATAG**
tHMGR<br>• from *Avena strigosa*	ATGGCGCCCGAGAAAATGCCCGAGGAGGACGAGGAAATCGTCGCCGGGGTCGTCGCAGGGAAGATCCCCTCCTACGTGCTCGAGACCAGGCTAGGCGACTGCCGCAGGGC<br>AGCCGGGATCCGCCGCGAGGCGCTGCGCCGGATCACCGGCAGGGAGATCGACGGCCTTCCCCTCGACGGCTTCGACTACGACTCGATTCTCGGACAGTGCTGCGAGATGC<br>CCGTCGGGTACGTGCAGCTGCCGGTCGGCGTCGCGGGGCCGCTCGTCCTCGACGGCCGCCGCATATACGTCCCGATGGCCACCACGGAGGGCTGCCTAATCGCCAGCACC<br>AACCGCGGATGCAAGGCCATTGCCGAGTCCGGAGGCGCATCCAGCGTCGTGTACCGCGACGGGATGACCCGCGCCCCCGTAGCCCGCTTCCCCTCCGCACGACGCGCCGC<br>AGAGCTCAAGGGCTTCCTGGAGAATCCGGCCAACTACGACACCCTGTCCGTGGTCTTTAACAGATCAAGCAGATTTGCAAGGCTGCAGGGGGTCAAGTGCGCCATGGCTG<br>GGAGGAACTTGTACATGAGGTTCACCTGCAGCACCGGGGATGCCATGGGGATGAACATGGTCTCCAAGGGCGTCCAAAATGTGCTCGACTATCTGCAGGAGGACTTCCCT<br>GACATGGACGTTGTCAGCATCTCAGGCAACTTTTGTTCCGACAAGAAATCAGCTGCTGTAAACTGGATTGAAGGCCGTGGAAAGTCCGTGGTTTGTGAGGCAGTAATCAG<br>AGAGGAAGTTGTCCACAAGGTTCTCAAGACCAACGTTCAGTCACTCGTGGAGTTGAATGTGATCAAGAACCTTGCTGGCTCAGCAGTTGCTGGTGCTCTTGGGGGTTTCA<br>ACGCCCACGCAAGCAACATCGTAACGGCTATCTTCATTGCCACTGGTCAGGATCCTGCACAGAATGTGGAGAGCTCACAGTGTATCACTATGTTGGAAGCTGTAAATGAT<br>GGCAGAGACCTTCACATCTCCGTTACAATGCCATCTATCGAGGTGGGCACAGTTGGTGGAGGCACGCAGCTGGCCTCACAGTCGGCCTGCTTGGACCTACTGGGCGTCAA<br>AGGCGCCAACAGGGAATCTCCGGGGTCGAACGCTAGGCTGCTGGCCACGGTGGTGGCTGGTGCCGTCCTAGCTGGGGAGCTGTCCCTCATCTCCGCCCAAGCTGCCGGCC<br>ATCTGGTCCAGAGCCACATGAAATACAACAGATCCAGCAAGGACATGTCCAAGATCGCCTGCTGA
AtCPR1<br>• from *Arabidopsis thaliana*	ATGACTTCTGCTTTGTATGCTTCCGATTTGTTTAAGCAGCTCAAGTCAATTATGGGGACAGATTCGTTATCCGACGATGTTGTACTTGTGATTGCAACGACGTCTTTGGC<br>ACTAGTAGCTGGATTTGTGGTGTTGTTATGGAAGAAAACGACGGCGGATCGGAGCGGGGAGCTGAAGCCTTTGATGATCCCTAAGTCTCTTATGGCTAAGGACGAGGATG<br>ATGATTTGGATTTGGGATCCGGGAAGACTAGAGTCTCTATCTTCTTCGGTACGCAGACTGGAACAGCTGAGGGATTTGCTAAGGCATTATCCGAAGAAATCAAAGCGAGA<br>TATGAAAAAGCAGCAGTCAAAGTCATTGACTTGGATGACTATGCTGCCGATGATGACCAGTATGAAGAGAAATTGAAGAAGGAAACTTTGGCATTTTTCTGTGTTGCTAC<br>TTATGGAGATGGAGAGCCTACTGACAATGCTGCCAGATTTTACAAATGGTTTACGGAGGAAAATGAACGGGATATAAAGCTTCAACAACTAGCATATGGTGTGTTTGCTC<br>TTGGTAATCGCCAATATGAACATTTTAATAAGATCGGGATAGTTCTTGATGAAGAGTTATGTAAGAAAGGTGCAAAGCGTCTTATTGAAGTCGGTCTAGGAGATGATGAT<br>CAGAGCATTGAGGATGATTTTAATGCCTGGAAAGAATCACTATGGTCTGAGCTAGACAAGCTCCTCAAAGACGAGGATGATAAAAGTGTGGCAACTCCTTATACAGCTGT<br>TATTCCTGAATACCGGGTGGTGACTCATGATCCTCGGTTTACAACTCAAAAATCAATGGAATCAAATGTGGCCAATGGAAATACTACTATTGACATTCATCATCCCTGCA<br>GAGTTGATGTTGCTGTGCAGAAGGAGCTTCACACACATGAATCTGATCGGTCTTGCATTCATCTCGAGTTCGACATATCCAGGACGGGTATTACATATGAAACAGGTGAC<br>CATGTAGGTGTATATGCTGAAAATCATGTTGAAATAGTTGAAGAAGCTGGAAAATTGCTTGGCCACTCTTTAGATTTAGTATTTTCCATACATGCTGACAAGGAAGATGG<br>CTCCCCATTGGAAAGCGCAGTGCCGCCTCCTTTCCCTGGTCCATGCACACTTGGGACTGGTTTGGCAAGATACGCAGACCTTTTGAACCCTCCTCGAAAGTCTGCGTTAG<br>TTGCCTTGGCGGCCTATGCCACTGAACCAAGTGAAGCCGAGAAACTTAAGCACCTGACATCACCTGATGGAAAGGATGAGTACTCACAATGGATTGTTGCAAGTCAGAGA<br>AGTCTTTTAGAGGTGATGGCTGCTTTTCCATCTGCAAAACCCCCACTAGGTGTATTTTTTGCTGCAATAGCTCCTCGTCTACAACCTCGTTACTACTCCATCTCATCCTC<br>GCCAAGATTGGCGCCAAGTAGAGTTCATGTTACATCCGCACTAGTATATGGTCCAACTCCTACTGGTAGAATCCACAAGGGTGTGTGTTCTACGTGGATGAAGAATGCAG<br>TTCCTGCGGAGAAAAGTCATGAATGTAGTGGAGCCCCAATCTTTATTCGAGCATCTAATTTCAAGTTACCATCCAACCCTTCAACTCCAATCGTTATGGTGGGACCTGGG<br>ACTGGGCTGGCACCTTTTAGAGGTTTTCTGCAGGAAAGGATGGCACTAAAAGAAGATGGAGAAGAACTAGGTTCATCTTTGCTCTTCTTTGGGTGTAGAAATCGACAGAT<br>GGACTTTATATACGAGGATGAGCTCAATAATTTTGTTGATCAAGGCGTAATATCTGAGCTCATCATGGCATTCTCCCGTGAAGGAGCTCAGAAGGAGTATGTTCAACATA<br>AGATGATGGAGAAGGCAGCACAAGTTTGGGATCTAATAAAGGAAGAAGGATATCTCTATGTATGCGGTGATGCTAAGGGCATGGCGAGGGACGTCCACCGAACTCTACAC<br>ACCATTGTTCAGGAGCAGGAAGGTGTGAGTTCGTCAGAGGCAGAGGCTATAGTTAAGAAACTTCAAACCGAAGGAAGATACCTCAGAGATGTCTGGTGA
ptmT2<br>• from *Streptomyces platensis*<br>• Optimized for *Aspergillus* expression<br>• overhang for yeast homologous recombination cloning into pTYGS-*argB* under P_{gpdA} promotor	**CAGCTACCCCGCTTGAGCAGACATCACCGG**ATGCTGGAAGTACCAGCACAACCTACTCCAGCTCCACGTGAAGCTGAAGCTGCTGCACTGCTGGCTGCTACCGTTGCAGA<br>CCCGTGGGGTCTGGTTGCTCCAAGCGTATACGATACCGCTCGTCTGGTAAGCCTGGCACCGTGGCTGGACGGTCATCGTGAACGCCTGGGTTACCTGGTTAAAGAACAAA<br>ACCAGGACGGTTCTTGGGGTGCTCCAGATGGCTATGGTCTGGTACCGACCCTGTCTGCAGTTGAAGCACTGCTGACCGAACTGGCACGTACTGATTCTGGCGCACCGCAC<br>CCGCCACATGATGATCTGGCTGCAGCTTGTGCAGGTGGTCTGGGTGCCCTGCAGGATGGTCTGCTGGCAGGTCCAGTTCCGGATACGATTGGTGTGGAATTTGTTGCTCC<br>TTCTCTGCTGGCGGATATCAACACCCGTCTGGCTGCCCTGACGGAACAGGCTCCAGGTAAACTGGGTGCTTGGTCTGGTACCACCCTGACGTCTCCGGCACCTGACCTGG<br>ATGGTGCTCTGCTGGCAGGTGTTCGCGAAATGACCGAACAGGCACCGCTGCCGGAAAAACTGTGGCATACCCTGGAAGCAATTACGCGTGACGGTACTCGTGGTGCACGT<br>CCACACGAAGGTGCTCCGCCACACAATGGTTCTGTAGGTTGTAGCCCGGCAGCAACTGCTGCATGGCTGGGTGCTTCTCCAGATCCAGCTGCACCAGGCGTTGCATACCT<br>GCGCGATGTACAGGCACGTTTCGGTGGTCCGGTACCGTCCATCACCCCGATCGTTTACTTCGAACAGGCTTGGGTTCTGAATTCTCTGGCCGCTTCTGGTCTGCGTTATG<br>AAGCTCCGGCAGCGCTGCTGGATAGCCTGGAAGCTGGTCTGACCGATGAAGGTATTGCAGCAGCACCGGGCCTGCCGTCTGATTCTGACGATACCGCTGCGGTACTGTTC<br>GCTCTGGCTCAGCACGGTCGTACCCACCGTCCAGACTCCCTGATGCACTTTCGTCGTGACGGTTATTTCTCTTGCTTTGGCGTAGAACGTACCCCGTCTACCTCTACCAA<br>CGCTCACATCCTGGAGGCTCTGGGCCACCACGTCACTGTACGTCCAGATGACGCAGGCCGCTATGGCGCTGAAATCCGTATGATCTCTGATTGGCTGCTGGATAACCAGC<br>TGCCAGACGGCTCTTGGATGGACAAATGGCATGCATCCCCGTATTACGCGACCGCTTGTTGCGCACTGGCTCTGGCAGAATTCGGTGGTCCATCTGCCCGTGCAGCAGTT<br>GATCGTGCTGCAGCATGGGCGCTGGAAACTCAGCGTGCAGATGGTTCTTGGGGTCGTTGGCAGGGTACGACCGAAGAAACTGCGTACATGGTTCAGCTGCTGATGCGTAC<br>TCGTACTCCGGGTTCTCCTGGTACTGTGGCACGTTCTGCAGCTCGTGGTTGCGATGCACTGCTGGCCCACGATGATCCGGCGTCTTATCCAGGTCTGTGGCATGATAAAG<br>ATATCTACGCACCAGTAACCGTTATCCGTGCAGCTCGTCTGGCTGCTCTGGCACTGGGTGGTGCTGAATCCGCTGCTTCTGGCGGTGCTTGA**CGCGCCGGTCATGATTGA**<br>**TGATATGGACAT**
Bjks<br>• from *Bradyrhizobium japonicum*<br>• Optimized for *Aspergillus* expression<br>• overhang for yeast homologous recombination cloning into pTYGS-*sC* under P_{gpdA} promotor	**CAGCTACCCCGCTTGAGCAGACATCACCGG**ATGATCCAGACCGAACGTGCAGTTCAACAAGTACTGGAATGGGGCCGCTCTCTGACCGGTTTCGCGGACGAACACGCAGT<br>TGAAGCCGTACGTGGCGGCCAGTACATCCTGCAGCGTATTCACCCGTCTCTGCGCGGTACCAGCGCGCGTACCGGTCGCGATCCGCAGGATGAAACCCTGATTGTGACCT<br>TCTACCGCGAGCTGGCTCTGCTGTTCTGGCTGGACGATTGTAACGACCTGGGTCTGATCTCCCCGGAACAGCTGGCCGCTGTTGAACAGGCGCTGGGCCAGGGTGTGCCG<br>TGTGCACTGCCAGGTTTTGAAGGCTGCGCAGTTCTGCGTGCGAGCCTGGCAACTCTGGCATACGACCGTCGTGACTACGCTCAGCTGCTGGACGATACCCGCTGTTACTC<br>CGCAGCACTGCGCGCCGGTCATGCGCAGGCTGTTGCTGCAGAGCGTTGGTCTTACGCCGAATACCTGCACAACGGCATTGACTCCATTGCCTACGCGAACGTTTTCTGTT<br>GTCTGAGCCTGCTGTGGGGCCTGGACATGGCGACTCTGCGTGCTCGTCCGGCATTTCGTCAAGTACTGCGCCTGATCTCCGCCATTGGCCGTCTGCAAAACGATCTGCAT<br>GGCTGCGACAAAGATCGTAGCGCGGGCGAAGCTGATAACGCCGTAATTCTGCTGCTGCAGCGTTACCCGGCTATGCCGGTTGTTGAGTTCCTGAACGATGAACTGGCTGG<br>CCACACTCGCATGCTGCACCGTGTAATGGCGGAAGAACGTTTCCCAGCACCGTGGGGTCCGCTGATCGAAGCTATGGCAGCTATCCGCGTTCAGTACTACCGTACGTCCA<br>CCTCCCGCTACCGTAGCGACGCAGTACGCGGCGGTCAGCGTGCACCGGCTTGA**CGCGCCGGTCATGATTGATGATATGGACAT**
IrCYP706V6<br>• from *Isodon rubescens*<br>• Overhang for Golden Gate cloning into pHREAC	**CACCACAGGTCTCGAAAA**ATGGCAGCAGTTCTGATTCTCACACTTTCCACCGTCTGGTTTTTCCAGAAGATGATCAAATCCGGAGATAAAAAGGCCGTGTCGCCACCGGG<br>GCCGCGCGGCCTGCCAATCGTGGGGTACTCGCCATTTCTCCGCCGCGACCTGCACCACCAGCTCACGGAACTGGCCGCTGAATACGGCCCTATCTACAAGCTCCGCATGG<br>GGAGCAAGCTATCCACTGTCATCAGCTCGGCTTCGCTGGCAAAAGAGGTGTTCCGTGACCATGACGCCGTCTTCGCCAACCACGATATCACGGTGGCAGCGCGCATCATC<br>TCGCTCGACTTCAACGACATCGCCTTTTCGCCCTACGGCCCGGAATGGCGCGACCGCCGAAAGATATTACTGCGGGAGATGCTAAGCAGTACCAACCTCAAGGCCTCCTG<br>CAATCTCCGGAAGGATCAAGTGCGAAATGCCGTCAAGGAGATCTATGCCGCAGCCGAGATCGGAACGCCCATTAAAATTTTCGACTTTGTTTTGAGAATTGATCTCAATC<br>TTTTAATAAACATGATCTGGGGCGGCAAGATTGGGGGCGAGCAGCGCCACCGCATCTCAGCAGGGCTATCCCCCATCGTGGCGGAGATTCAGGATTTTCTTATAAAGCCT<br>AACATCTCTGATTTTTTCCCTGTGTTAGCTAGGTTTGATTTTCAAGGAATCGCCAAGAAAACGACCGCTCTGATTCAGAAGTTTGAAGCGATTATGGAAGACGCCATTGG<br>TGAACGTATTAAGAACCCTTGCGCAGCAGAGGAGGAAGAAGCGCGGAAGGATTTCATGCAGACCCTCGTAGATCTCTTGCAGGAGGAAACTCACAAGGCTTCGCTAGGCA<br>TAGTACAAATTAAAGCCATGTTGGTAAACATACTAATTGGTGGCACAGACACGACTTCAACGATGATCGAGTGGGTGATGGCGGAGCTGCTGAACCATCCCAAAGTGATG<br>GAAGAAGTGCAACACGAACTGAATGAAGTTGTGGGGAAGAAGAACATAGTGGAAGAGTCTCACATCCCTAAACTAGTCTACTTAGACGCGGTCCTAAAGGAAACAATGCG<br>GGTGCACCCAATAGGTCCTTTATTACCCAGAACACCGAACCGGGATTGCACGGTCGGGGGATACACCATCCCCAAGGACTCCGGGGTGTACGTGAATATCTGGTCCATCC<br>AAAGAGATCCCGCAGTGTGGGACAATCCATCGGAGTTTAGGCCGGAGAGATTCTTAGCAGACGAAACCGGGAAGTGGGGTTTTAGTGGTAAAAATATGAATTATATCCCA<br>TTTGGATCGGGGAGAAGGGTGTGTGCTGGGCTGCCGTTGGCTGAAATAATGTTGAGGTACATCTTAGCTTCGCTGTTGCACTCGTTCGAGTGGCGACTGCGCGACGGCGA<br>GGAGCTGGACATGTCGGATGAGATCGTGTCGGCACTGAAGAAACGCATTCCTCTCGTCGCCATACCGCATCCCAGGCTGCCCTGCTCCAGTCTATATCAATAA**CGCTCGA**<br>**GACCTGTGGTG**
IrCYP706V7<br>• from *Isodon rubescens*<br>• Overhang for Golden Gate cloning into pHREAC	**CACCACAGGTCTCGAAAA**ATGAAAGCGGCGTTACTAATGGCAGCTCTGATTCTCACACTTGCCATCATCTGGTTTTTCCGGAAGATGATCAAATCCGGTGAAAAGGGGGC<br>CATGTCGCCGCCGGGGCCGCGCGGGCTGCCGATCGTGGGGTACTTGCCGTTTCTCCGCCGCGATATCCACTGCCAGCTCACAGAACTGGCGGCTCATTATGGCCCAATCT<br>ACAAGATTCGCGCCGGGAGCAAACTGTACACCGTGATCGGCTCCACTTCGCTGGCAAAAGAGGTGTTTCGCGACAACGACGTCGTCCTTGCCAACCGCGACATGACGATC<br>GCAGCTGGCATCGCCACCTTAGACTTCAACGACATCGCCTGGTCGCCCTACGGCCCAGGATGGCGCGACCGCCGCAAAGTATTCTCGCGGGAGATGCTGAGCCACGCAAA<br>CCTTAAGGCCTCCTACAATCTCCGAAAGGATACAATGCGAAGGACCATCGGAGAAATCTACGCCGACAAGGCAAGAACGCCCATTGGTATTTTCAAGCTGGGTTTGAAGA<br>TTGATCTCGATCTTATCTTAAACATGGTCTGGGGTGGAAAGATTGGGGCGGAGCGGCGCGACAAGATGTCAGCCGGCCTACTGCCCGTCGTGGCGGAGTTGCTGGACTTG<br>CTTTTTAAGCCTAACATCTCTGATTTCTTCCCTGTGTTAGCTAGGTTTGATTTTCAAGGAATTGCCAAGGCAATGACTGCTCAGCTTCAAAAGATTGAAGCTATGACCGA<br>AGACGCCATTGAAGAACGCATCAAGAATCCTAATGCAGGAGTGGAGAAAGAAGGGCGAAAGGATTTCATGCAGACTCTTGTAGAGCTCATGCAGGAAGAAAGTTACAAGT<br>CATCCCTAGGTATAAAGCAGATCAAAGGCATGTTGGTGGACATACTAATCGGTGGCACAGACACGACATCAACCACGGTCGAGTGGGTGATGACACAACTACTACGACAT<br>CCCAAAGTGATGAAAAAAGTGCAACAAGAACTGGATGAAGTTGTGGGGATGAACAACATAGTGGAAGAAACCCACATGCCTAAACTAGTCTACCTAGACGCGGTTATAAA<br>GGAAACAATGCGGCTTAACCCGATAGGTCCTTTAATAGCAAGAACACTGAGCCAGAGCTGCACGGTCGGGGGTTACACGATCCCAAAGGACTCCTCGGTGTTCGTAAATG<br>TGTGGTCCCTCCAAAGGGACCCTGTGGCGTGGGACGATCCATCGGAGTTCAAGCCGGAGAGATTCCTAGAAGGCGGCGGAGCTGGGAATCCGGCCGATTTTAGTAGCAAT<br>AATTTCAACTTTATTCCATTTGGATCGGGGAGAAGGATGTGTGCCGGCCTGCAGTTGGCCGAAAGAATGATGATGTATATATTGGGGTCGCTGTTGCACTCTTTTGAGTG<br>GCGGCTGCGCGACGGCGACGAGCTAGACATGTCCGAGGAGCTGGTGACGGCGTTGAGGAAGCGCAATCCTCTTTATGTCGTTCCCATTCCCAGGTTGCCGCATCCGGAGC<br>TATATAACTAG**CGCTCGAGACCTGTGGTG**
MdDOX-Co<br>• from *Malus domestica*	**CACCACAGGTCTCGAAAA**ATGGAGACATTAGATCAGAATCTTGTTTCAAGCTGGTTTGATGTTCAATCTGTTCCCCAAACCTTTGTCCACCCACCTGAAAAGCGGCCCGG<br>TAACAGTATTGACGTTCCTCCGTGCAAGAACATTCCGGTAGTTGATCTTGGCAGCCGTGATCGCAGTCACACAATTCAACAAATTTCCAAGGCTAGCCAAGACTTCGGAT<br>TTTTCCAGGTCTTCAATCATGGGGTTTGCAAGAAGTTGATTGATGACATAATGAGTATTTCCAAGGAGTTTCATAAAATGCCCCGAAAAGATAAGATAATTGAAGGCTCA

• Overhang for Golden Gate cloning into pHREAC	AAGGACCCTAGTGGAAGATGCAAGTTCTACACAAGCAGTGAAAACTATGCGAATGAAGAGGTTCACTATTGGCGAGATGCATTGACTCACCCTGCTCATTCTTCTGAAAA<br>CTACATGCAGTTTTTGCCTCAAAAACCTACTCAATACCGAGAAGTTTTCAAGGCATACGTGGATGAGGTAAGAAACATGGGTTCTATGATTTTGGAGATGCTTGCTGAAG<br>GGTTGGGACTGAGCAAAGAGTTCTTCAATGGTGGACTTAGTGAAAATCCAACGCTGCTGTCCAACCATTATCCGCCATGCCCAGATCCTACTTTAACCTTGGGATTAACG<br>AAACACCGCGATCCGAGCCTCATAACCATTTTACTTCAAGATTCAGAAGGACTTCAAGTCTTCAAAGATGGGAATTGGATTGGTGTTGAGCCTATTTCTAGTGGCTTTGT<br>TGTTAACATAGGCTACGTCATGCAGATGATCAGCAACTCGAAGTTTAAAGGCGCTGACCATCGAGTGGTGACAAATTCAAGAGCTGCAAGGACAACGATTGCGTACTTCA<br>TTTATCCGTCTAATGAAACCGTTATAGAACCTGCAAATGTTTTGTGCAATCCACCACTGTACAGATCCATGAAGTTTACGGAGTTCCTTCAACACTTCAAATCGAAAGCT<br>GCCAACGACGAAGAAATGTCGAAGGTTTTAAGCCTCTCGAGTAGCTAA**CGCTCGAGACCTGTGGTG**
RoCYP76AH22<br>• from *Rosmarinus officinalis*<br>• Overhang for Golden Gate cloning into pHREAC	**CACCACAGGTCTCGAAAA**ATGGATTCTTTTCCTCTTCTCGCTGCGCTCTTCTTCATCCTCGCTGCGACATGGTTCATTTCCTTCCGGCGCCCGAGGAACCTCCCCCCGGG<br>GCCTTTCCCCTATCCGATCGTCGGAAACATGCTGCAGCTCGGCACGCAACCCCACGAGACATTCGCCAAACTCTCCAAGAAATACGGCCCTCTCATGTCAATCCACCTCG<br>GCAGCCTATACACCGTCATCGTCTCCTCCCCCGAGATGGCCAAAGAAATCATGCACAAATACGGGCAAGTCTTCTCCGGCAGAACCGTAGCGCAGGCGGTCCACGCGTGC<br>GGCCACGACAAGATCTCGATGGGGTTCCTCCCCGTGGGGGGCGAGTGGCGCGACATGCGCAAGATATGCAAAGAACAGATGTTCTCGCATCAGAGCATGGAAGACAGCCA<br>GTGGCTCCGCAAGCAGAAGCTGCAGCAGCTGCTCGAATACGCCCAGAAATGCTCCGAGCGCGGCCGGGCAATCGATATCCGTGAGGCCGCCTTCATCACCACGCTCAACC<br>TCATGTCTGCCACTCTCTTCTCCATGCAGGCGACTGAATTCGACTCCAAAGTCACCATGGAATTCAAGGAGATCATCGAGGGTGTGGCCAGCATCGTTGGCGTGCCCAAC<br>TTCGCCGATTACTTCCCCATCCTGAGGCCGTTCGACCCCCAGGGGGTGAAGCGCAGGGCGGATGTTTACTTCGGCAGATTGCTGGCCATAATCGAGGGATTTCTCAACGA<br>AAGAGTCGAATCCAGACGGACCAATCCGAATGCTCCTAAGAAGGATGACTTTCTCGAAACGCTTGTGGATACCCTTCAGACGAACGACAACAAGTTGAAGACCGATCACC<br>TCACACATCTCATGCTGGACTTGTTTGTTGGAGGATCTGAAACAAGCACGACAGAGATCGAGTGGATAATGTGGGAGTTACTCGCTAACCCGGAGAAGATGGCGAAGATG<br>AAAGCAGAGCTGAAGAGCGTGATGGGAGAGGAGAAAGTAGTGGACGAGTCGCAGATGCCGAGGCTGCCATATCTGCAAGCAGTGGTGAAGGAATCGATGCGGCTCCACCC<br>GCCGGGCCCTCTGCTTCTTCCTCGCAAAGCAGAGAGCGATCAGGTGGTGAACGGCTACCTCATCCCCAAGGGAGCTCAGGTGCTGATCAACGCGTGGGCTATAGGCAGGG<br>ACCACAGCATCTGGAAGAATCCGGACTCCTTTGAGCCTGAACGCTTCCTCGATCAGAAGATAGACTTCAAGGGCACGGATTACGAGCTCATCCCCTTCGGGTCGGGTAGA<br>AGGGTCTGTCCGGGTATGCCGTTGGCCAACCGGATTCTGCATACGGTCACCGCCACTCTGGTTCACAACTTCGATTGGAAGCTCGAACGGCCTGAAGCAAGTGATGCGCA<br>CCGTGGTGTGTTGTTTGGCTTTGCGGTGCGGAGGGCGGTTCCACTCAAGATCGTTCCGTTTAAGGTATAA**CGCTCGAGACCTGTGGTG**
AtCYP714A1<br>• from *Arabidopsis thaliana*<br>• Overhang for Golden Gate cloning into pHREAC	**CACCACAGGTCTCGAAAA**ATGGAGAATTTTATGGTAGAGATGGCCAAGACCATTTCGTGGATAGTTGTAATAGGAGTGTTAGGTTTAGGGATTCGTGTTTACGGTAAAGT<br>GATGGCGGAGCAATGGAGGATGCGGAGGAAGCTGACGATGCAGGGCGTGAAGGGTCCTCCGCCGTCGCTATTTCGTGGAAACGTGCCGGAGATGCAAAAAATCCAGTCAC<br>AAATAATGAGTAACTCTAAACACTACTCCGGTGATAATATCATCGCCCATGACTACACTTCTTCTCTCTTTCCATATCTCGACCACTGGCGAAAACAATACGGAGAGTG<br>TACACGTATTCGACGGGGGTGAAGCAACACTTGTACATGAACCACCCGGAGTTGGTGAAAGAACTTAACCAAGCCAACACTCTTAACCTTGGCAAAGTCTCTTACGTCAC<br>CAAACGCCTTAAATCCATTCTTGGCCGTGGTGTTATCACCTCTAATGGGCCTCATTGGGCCCATCAACGTCGTATCATTGCACCTGAGTTTTTCCTCGACAAAGTCAAGG<br>GAATGGTGGGATTGGTGGTGGAATCAGCGATGCCAATGCTGAGTAAATGGGAAGAGATGATGAAAAGAGAAGGAGAAATGGTGTGTGACATAATTGTAGACGAAGACCTA<br>AGAGCTGCCTCTGCTGACGTTATCTCTAGAGCTTGCTTTGGGAGCTCTTTCTCCAAAGGCAAAGAGATCTTCTCTAAGCTTAGATGTCTTCAAAAGGCCATCACTCACAA<br>CAACATCCTCTTCAGCCTCAATGGCTTCACTGATGTTGTGTTCGGGACTAAGAAGCATGGGAACGGGAAGATTGATGAGCTAGAGAGACACATTGAGTCTTTGATATGGG<br>AAACCGTTAAAGAAAGAGAAAGGGAATGTGTGGGAGATCACAAGAAGGATCTAATGCAGTTGATACTAGAAGGGGCCAGGAGTAGTTGTGATGGCAACTTGGAGGACAAG<br>ACACAATCTTACAAAAGTTTCGTGGTGGACAATTGCAAGAGCATCTATTTTGCCGGGCATGAGACGAGTGCGGTTGCTGTCTCTTGGTGTCTTATGCTCCTCGCTCTCAA<br>CCCTTCTTGGCAAACTCGTATCCGCGATGAAGTCTTTCTTCATTGCAAGAACGGTATACCTGACGCAGACTCTATTTCCAACCTCAAAACGGTGACAATGGTTATCCAGG<br>AAACGTTGAGGCTATACCCACCAGCAGCATTCGTGTCGAGAGAAGCCCTTGAGGACACAAAACTCGGAAACCTCGTGGTGCCAAAGGGAGTGTGCATCTGGACGTTGATC<br>CCTACATTGCACAGAGATCCTGAGATATGGGGAGCTGACGCAAATGAATTCAATCCAGAAAGATTTAGCGAAGGAGTCTCTAAAGCCTGCAAACACCCTCAGTCATTCGT<br>CCCATTTGGCTTAGGGACAAGGTTGTGTTTAGGAAAGAATTTTGGTATGATGGAGCTCAAGGTTCTTGTCTCACTTATTGTGTCAAGGTTTAGCTTTACTCTCTCTCCCA<br>CATATCAACACTCTCCGGTGTTTAGAATGCTTGTAGAGCCTCAACATGGTGTTGTCATTAGAGTTCTGAGACAATAA**CGCTCGAGACCTGTGGTG**
AtCYP714A2<br>• from *Arabidopsis thaliana*<br>• Overhang for Golden Gate cloning into pHREAC	**CACCACAGGTCTCGAAAA**ATGGAGAGTTTGGTTGTTCATACGGTAAATGCAATTTGGTGCATAGTTATTGTCGGAATCTTCAGCGTAGGTTATCATGTGTATGGAAGAGC<br>GGTGGTGGAGCAGTGGAGGATGCGGAGGAGTTTAAAGTTGCAAGGCGTGAAGGGTCCTCCACCGTCGATCTTTAACGGCAATGTGTCGGAGATGCAACGGATTCAGTCGG<br>AGGCTAAACACTGTTCCGGCGATAACATCATTTCTCATGACTATTCTTCTTCTCTATTTCCTCATTTCGATCACTGGCGAAAACAATACGGAAGGATTTACACATACTCA<br>ACGGGGTTAAAGCAGCACCTTTACATAAACCACCCGGAAATGGTGAAGGAGCTTAGCCAAACCAACACACTTAACCTTGGTAGAATCACTCACATCACCAAACGCCTTAA<br>CCCCATTCTCGGCAATGGCATCATCACCTCTAATGGGCCTCATTGGGCCCATCAACGTCGTATCATTGCCTATGAGTTTACCCACGACAAAATCAAGGGAATGGTTGGTT<br>TAATGGTGGAATCTGCCATGCCAATGTTGAACAAATGGGAAGAGATGGTGAAAAGAGGAGGAGAAATGGGTTGTGACATAAGAGTGGACGAAGACCTTAAGGATGTCTCA<br>GCTGATGTCATCGCTAAGGCTTGCTTTGGGAGCTCTTTTTCAAAAGGCAAAGCAATATTCTCTATGATTAGGGATCTTTTAACCGCCATTACTAAACGAAGCGTCCTCTT<br>CAGATTCAATGGCTTCACTGATATGGTGTTTGGAAGTAAGAAGCATGGTGATGTGGATATTGATGCGCTTGAGATGGAATTAGAATCTTCTATATGGGAAACGGTTAAGG<br>AGAGGGAAATTGAATGTAAGGATACTCACAAGAAGGATCTAATGCAGTTGATACTCGAGGGAGCGATGCGAAGCTGCGATGGTAACTTGTGGGACAAGTCAGCCTATAGA<br>CGGTTTGTGGTGGACAATTGCAAGAGCATCTATTTCGCCGGACATGATTCAACCGCAGTCTCAGTGTCTTGGTGCCTTATGCTCCTCGCTCTCAATCCTAGTTGGCAGGT<br>TAAAATTCGCGATGAAATCTTGAGTTCTTGCAAGAATGGCATTCCCGACGCAGAATCAATTCCTAATCTCAAAACGGTGACAATGGTAATACAAGAAACAATGAGACTAT<br>ACCCACCAGCACCAATCGTGGGAAGAGAAGCATCCAAAGACATAAGACTTGGAGACCTTGTGGTGCCAAAAGGAGTGTGCATTTGGACACTCATTCCTGCCTTACACCGA<br>GACCCCGAGATCTGGGGACCAGACGCAAACGACTTCAAGCCAGAGAGGTTTAGTGAGGGAATCTCTAAGGCTTGCAAATACCCTCAGTCATACATCCCATTTGGCCTTGG<br>ACCAAGAACATGCGTAGGCAAAAACTTTGGTATGATGGAAGTGAAAGTGCTTGTTTCACTTATTGTCTCAAAGTTCAGTTTTACTCTTTCCCCGACTTATCAGCACTCTC<br>CAAGCCATAAACTCCTTGTAGAGCCTCAACATGGTGTTGTCATTAGGGTTGTTTGA**CGCTCGAGACCTGTGGTG**
AtCYP72A9<br>• from *Arabidopsis thaliana*<br>• Overhang for Golden Gate cloning into pHREAC	**CACCACAGGTCTCGAAAA**ATGGAGATAGTAATTGCATCATTGGCTTTAGTTGTTGTATTGTGGTGTATATGGAGAATTTTGGAGTGGGTTTGGCTTAAACCTAAGATGCT<br>TGAGAGTTACCTGAGAAGACAAGGTCTTGTCGGAACTCGTTACACGCCTCTTGTAGGAGATGTGAGAAGGAGTTTTAGCATGTTGAAGGAAGCAAGATCCAAACCGATGA<br>AACCAACGGATGATCTCATCTCACTTGTCATGCCTTACTCTTTTCACATGCTCAACACTTACGGGAAGACATTTTTCACATGGTCAGGACCCATACCTGCCATAACAATA<br>ATGAATCCACAGCTGATCAAAGAAGTGTACAACAAGTTTTATGATTTCGAGAAGACGCACACGTTTCCTTTGACTTCATTGCTAACTGATGGACTTGCTAATGCCGATGG<br>AGATAAATGGGTGAAACACCGGAAGATTATCAACCCGGCATTCCATTTTGAGAAGATTAAGAATATGGTCCCTACGTTTTATAAAAGTTGCATCGAGGTCATGTGTGAAT<br>GGGAGAAGTTAGTTTCAGATAAAGGGTCGTCGTGCGAGCTTGATGTTTGGCCATGGATTGTGAATATGACCGGAGATGTGATCTCTCGAACCGCTTTTGGTAGCAGTTAC<br>AAAGAAGGACAGAGGATATTCATATTACAAGCAGAATTAGCCCACCTCATCATACTAGCTCTTGGAAAGAATTACATTCCTGCATACCGGCATTTCCCAACTAAGAATAA<br>CAGAAGGATGAAAACAATAGTCAAAGAAATCCAAGTCATACTAAGAGGGATCATTAGCCATAGAGAAAAGGCGAGAGACGCTGGAGAAGCACCGAGTGACGATTTGTTAG<br>GTATACTTCTTAAATCGAATTCAGAGCAAAGTAAAGGAAATGGACTGAACATGGAAGAAATCATGGAAGAGTGCAAGTTGTTTTATTTTGCGGGACAAGAGACGACTTCA<br>GTACTTTTGGCTTGGACTATGGTTTTGTTAAGCCAACACCAAGACTGGCAAGCTCGTGCACGAGAGGAAGTGATGCAAGTTTTTGGCCATAACAAACCTGATCTACAAGG<br>CATTAACCAACTCAAAGTTATGACAATGATTATATATGAAGTCCTGAGGCTATATCCTCCAGTAATCCAGATGAACAGAGCGACTCACAAAGAGATAAAGCTAGGTGATA<br>TGACACTACCAGGCGGTATTCAAGTCCATATGCCTGTTCTGCTAATCCACCGTGACACTAAGCTTTGGGGCGATGATGCAGCGGAGTTCAAGCCCGAGAGGTTCAAAGAC<br>GGGATCGCAAAGGCAACAAAGAACCAGGTCTGCTTCTTGCCTTTCGGGTGGGGACCGAGGATTTGCATCGGTCAGAACTTCGCTCTGTTGGAAGCAAAGATGGCTCTGGC<br>TTTGATTCTTCAGAGATTCTCCTTCGAGCTTTCACCTTCCTATGTTCATTCACCTTACAGAGTCTTCACTATACATCCACAGTGTGGGGCTCATCTTATCCTGCACAAGC<br>TTTGA**CGCTCGAGACCTGTGGTG**
At2g36690<br>• from *Arabidopsis thaliana*<br>• Overhang for Golden Gate cloning into pHREAC	**CACCACAGGTCTCGAAAA**ATGTCGCCGTCAATGATAGCTCCGGTGATGGAAGAAGAGAACAAGAAATACCAAAAAGGCGTGAAACATCTTTGTGAAAATGGTCTGACCAA<br>AGTACCAACCAAGTATATATGGCCTGAACCTGACCGACCCATCTTGACAAAATCGGACAAGCTCATCAAACCAAACAAAAACCTAAAGCTCCCCCTCATAGATTTCGCTG<br>AGCTCCTTGGACCTAACCGGCCTCATGTTCTACGAACCATAGCCGAGGCCTGCAAAACCTACGGCTTCTTTCAGGTTGTGAATCATGGGATGGAAGGAGACGTGAGCAAG<br>AATATGATAGACGTGTGTAAGAGATTCTTTGAGCTTCCGTACGAGGAGAGATCCAAGTACATGTCGTCGGACATGTCGGCTCCAGTACGATACGGCACGAGTTTCAACCA<br>GATCAAAGACAATGTCTTTTGTTGGAGAGACTTCTTGAAACTCTATGCACATCCACTCCCTGACTATCTTCCTCATTGGCCTTCTTCTCCTTCTGACTTCAGGAGTTCGG<br>CGGCTACATACGCTAAAGAGACGAAAGAGATGTTCGAAATGATGGTCAAAGCCATCTTAGAAAGTCTTGAAATTGATGGCAGTGATGAGGCGGCTAAGGAGTTGGAAGAG<br>GGAAGTCAGGTGGTGGTGGTGAACTGTTATCCACCGTGTCCGGAGCCAGAGCTGACGCTAGGGATGCCACCGCACTCAGACTACGGCTTCTTGACGCTTCTACTTCAAGA<br>CGAAGTCGAAGGTCTTCAAATTCTGTATCGTGACGAGTGGGTCACCGTTGATCCCATCCCTGGCTCTTTTGTTGTCAACGTAGGCGATCATCTCGAGATATTTAGCAATG<br>GGAGGTATAAAAGCGTGCTACATAGGGTATTGGTGAACTCTACAAAACCTAGGATATCGGTGGCGTCGCTACACAGCTTCCCTTTGACTTCTGTCGTAAAGCCATCGCCG<br>AAACTCGTCGATAAACATAATCCATCACAGTATATGGACACTGATTTTACCACTTTTCTTCAGTATATAACTTCTCGTGAACCCAAATGGAAGAATTTTCTTGAATCACG<br>CAAGATTCACCATGTATGA**CGCTCGAGACCTGTGGTG**
AtGA3ox1<br>• from *Arabidopsis thaliana*	ATGCCTGCTATGTTAACAGATGTGTTTAGAGGCCATCCCATTCACCTCCCACACTCTCACATACCTGACTTCACATCTCTCCGGGAGCTCCCGGATTCTTACAAGTGGAC<br>CCCTAAAGACGATCTCCTCTTCTCCGCTGCTCCTTCTCCTCCGGCCACCGGTGAAAACATCCCTCTCATCGACCTCGACCACCCGGACGCGACTAACCAAATCGGTCATG<br>CATGTAGAACTTGGGGTGCCTTCCAAATCTCAAACCACGGCGTGCCTTTGGGACTTCTCCAAGACATTGAGTTTCTCACCGGTAGTCTCTTCGGGCTACCTGTCCAACGC<br>AAGCTTAAGTCTGCTCGGTCGGAGACAGGTGTGTCCGGCTACGGCGTCGCTCGTATCGCATCTTTCTTCAATAAGCAAATGTGGTCCGAAGGTTTCACCATCACTGGCTC<br>GCCTCTCAACGATTTCCGTAAACTTTGGCCCCAACATCACCTCAACTACTGCGATATCGTTGAAGAGTACGAGGAACATATGAAAAAGTTGGCATCGAAATTGATGTGGT<br>TAGCACTAAATTCACTTGGGGTCAGCGAAGAAGACATTGAATGGGCCAGTCTCAGTTCAGATTTAAACTGGGCCCAAGCTGCTCTCCAGCTAAATCACTACCCGGTTTGT<br>CCTGAACCGGACCGAGCCATGGGTCTAGCAGCTCATACCGACTCCACCCTCCTAACCATTCTGTACCAGAACAATACCGCCGGTCTACAAGTATTTCGCGATGATCTTGG<br>TTGGGTCACCGTGCCACCGTTTCCTGGCTCGCTCGTGGTTAACGTTGGTGACCTCTTCCACATCCTATCCAATGGATTGTTTAAAAGCGTGTTGCACCGCGCTCGGGTTA<br>ACCAAACCAGAGCCCGGTTATCTGTAGCATTCCTTTGGGGTCCGCAATCTGATATCAAGATATCACCTGTACCGAAGCTGGTTAGTCCCGTTGAATCGCCTCTATACCAA<br>TCGGTGACATGGAAAGAGTATCTTCGAACAAAAGCAACTCACTTCAACAAAGCTCTTTCAATGATTAGAAATCACAGAGAAGAATGA
AtGA20ox1<br>• from *Arabidopsis thaliana*	ATGGCCGTAAGTTTCGTAACAACATCTCCTGAGGAAGAAGACAAACCGAAGCTAGGCCTTGGAAATATTCAAACTCCGTTAATCTTCAACCCTTCAATGCTTAACCTTCA<br>AGCCAATATCCCAAACCAATTCATCTGGCCTGACGACGAAAAACCTTCCATCAACGTTCTCGAGCTTGATGTTCCTCTCATCGACCTTCAAAACCTTCTCTCTGATCCAT<br>CCTCCACTTTAGATGCTTCGAGACTGATCTCTGAGGCCTGTAAGAAGCACGGTTTCTTCCTCGTGGTCAATCACGGCATCAGCGAGGAGCTTATTTCAGACGCTCATGAA<br>TACACGAGCCGCTTCTTTGATATGCCTCTCTCCGAAAAACAGAGGGTTCTTAGAAAATCCGGTGAGAGTGTTGGCTACGCAAGCAGTTTCACCGGACGCTTCTCCACCAA<br>GCTTCCATGGAAGGAGACCCTTTCTTTCCGGTTTTGCGACGACATGAGCCGCTCAAAATCCGTTCAAGATTACTTCTGCGATGCGTTGGGACATGGGTTTCAGCCATTTG<br>GGAAGGTGTATCAAGAGTATTGTGAAGCAATGAGTTCTCTATCACTGAAGATCATGGAGCTTCTGGGGCTAAGTTTAGGCGTAAAACGGGACTACTTTAGAGAGTTTTTC<br>GAAGAAAACGATTCAATAATGAGACTGAATTACTACCCTCCATGTATAAAACCAGATCTCACACTAGGAACAGGACCTCATTGTGATCCAACATCTCTTACCATCCTTCA<br>CCAAGACCATGTTAATGGCCTTCAAGTCTTTGTGGAAAATCAATGGCGCTCCATTCGTCCCAACCCCAAGGCCTTTGTGGTCAATATCGGCGATACTTTCATGGCTCTAT<br>CGAACGATAGATACAAGAGCTGCTTGCACCGGGCGGTGGTGAACAGCGAGAGCGAGAGGAAATCACTTGCATTCTTCTTGTGTCCGAAAAAAGACAGAGTAGTGACGCCA<br>CCGAGAGAGCTTTTGGACAGCATCACATCAAGAAGATACCCTGACTTCACATGGTCTATGTTCCTTGAGTTCACTCAGAAACATTATAGAGCAGACATGAACACTCTCCA<br>AGCCTTTTCAGATTGGCTCACCAAACCCATCTAA

NMR data and spectra

Gibberellin A_3 (Gibberellic acid) (1)

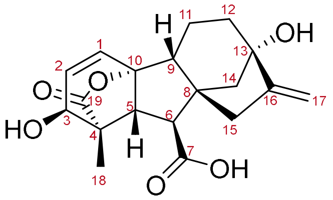

GA$_3$ (**1**)
Chemical Formula: $C_{19}H_{22}O_6$
Exact Mass: 346.1416

	Reference in C_5D_5N [40]		Measured in C_5D_5N, 298K	
**Pos.**	δ_C 50.1 MHz	δ_H (*J/Hz*) 200 MHz	δ_C 151 MHz	δ_H (*J/Hz*) 600 MHz
**1**	131.9	6.47	132.7	6.47, d (9.2)
**2**	133.8	6.19	134.7	6.20, dd (9.2, 3.6)
**3**	69.5	4.57	70.4	4.56, d (3.6)
**4**	54.0	-	54.9	-
**5**	53.0	3.94	53.9	3.93, d (10.8)
**6**	51.6	3.32	52.5	3.31, d (10.8)
**7**	174.4	-	175.3	-
**8**	50.1	-	51.0	-
**9**	51.1	2.08	52.0	2.07, overlapped
**10**	90.8	-	91.6	-
**11**	17.1	1.75<br>2.15	18.0	1.73, m<br>2.13, overlapped
**12**	39.4	2.05<br>2.42	40.3	2.02, overlapped<br>2.42, overlapped
**13**	77.3	-	78.1	-
**14**	45.0	2.35<br>2.35	45.9	2.33, dd (10.8, 2.5)<br>2.36, d (10.8)
**15**	43.5	2.43<br>2.82	44.4	2.41, overlapped<br>2.81, ddd (15.4, 2.9, 2.9)
**16**	158.5	-	159.7	-
**17**	106.5	5.03<br>5.63	107.1	5.03, br s<br>5.63, br s
**18**	15.1	1.79	15.9	1.77, s
**19**	179.3	-	180.2	-

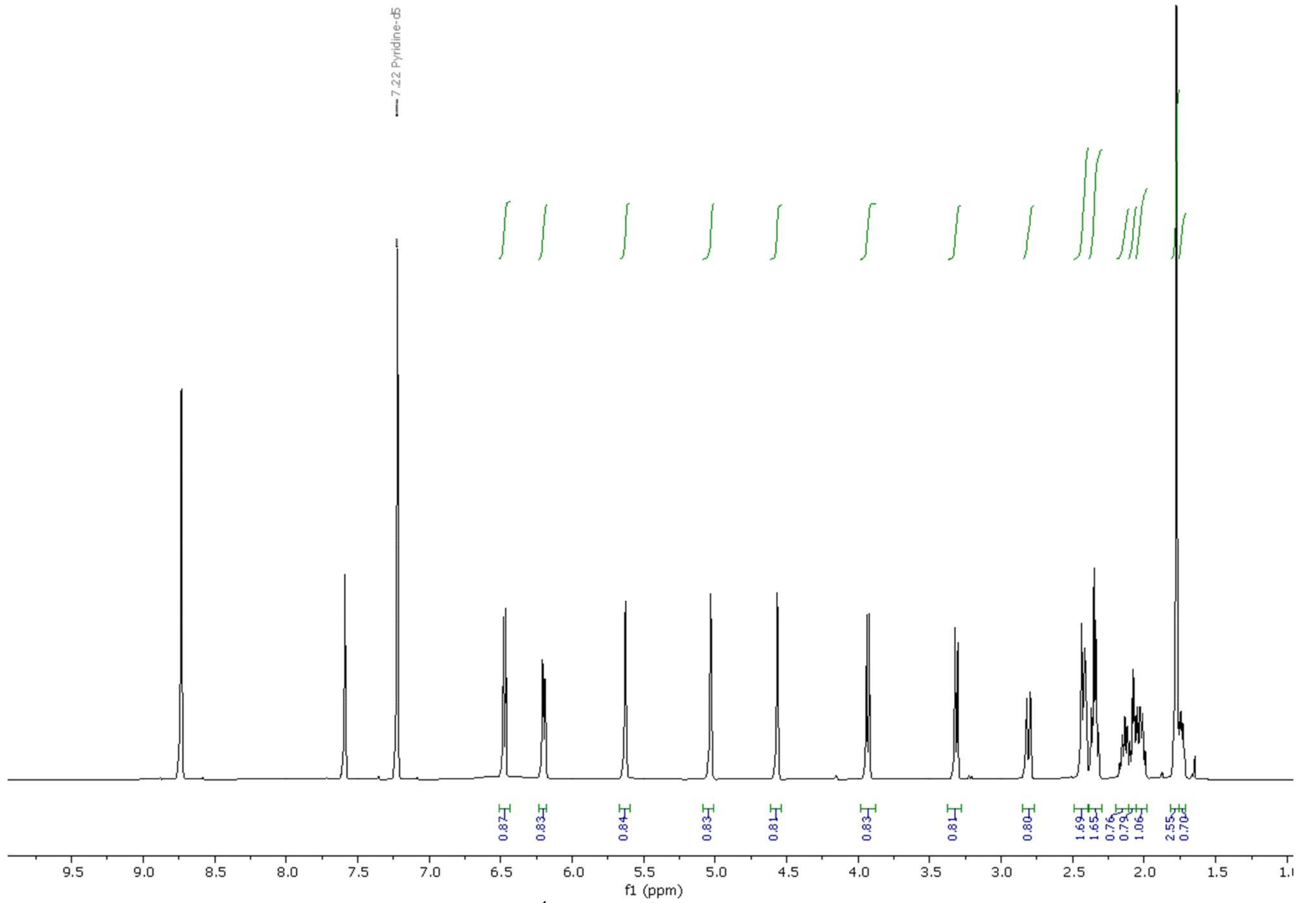

Figure N1.A ^{1}H NMR of **1** in d_5-pyridine at 600 MHz.

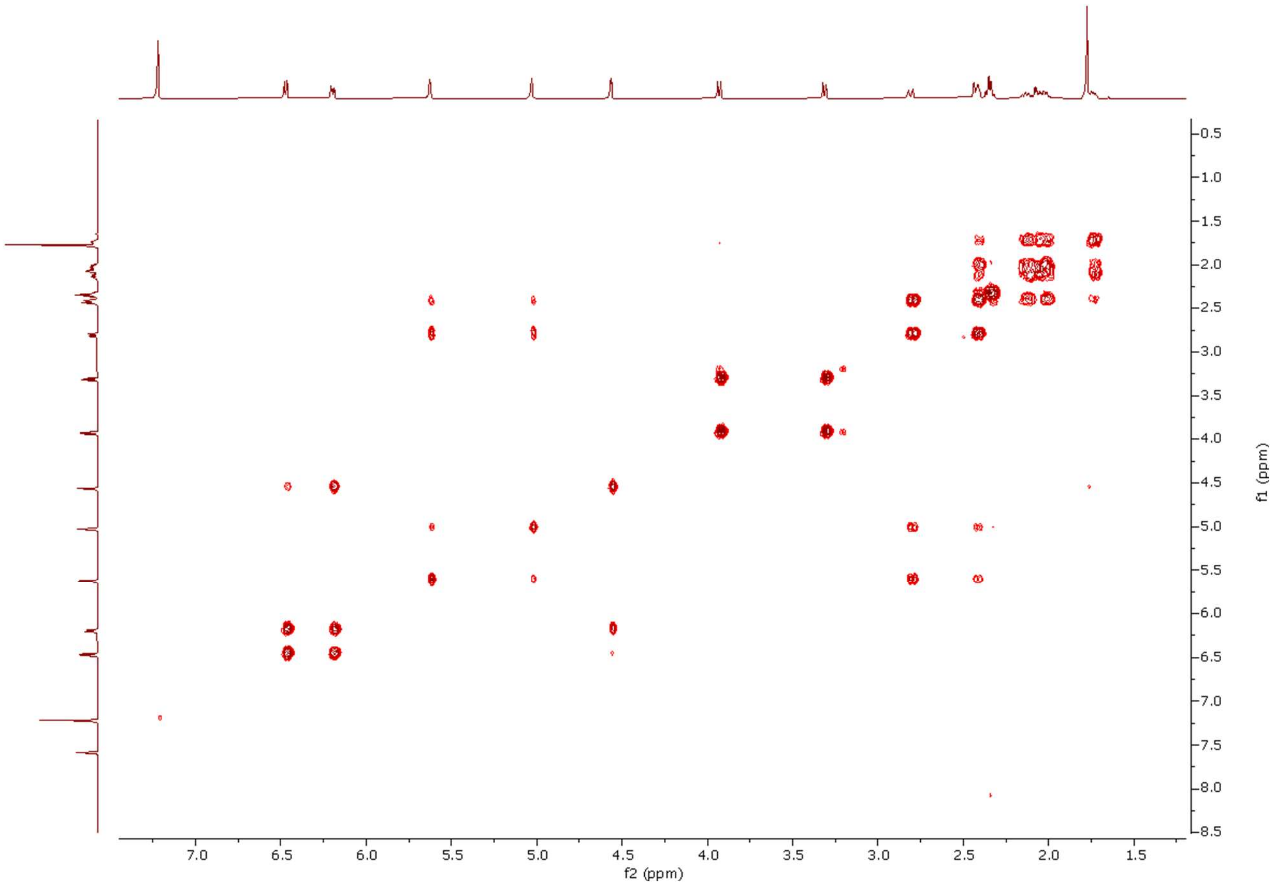

Figure N1.B COSY NMR of **1** in d_5-pyridine at 600 MHz.

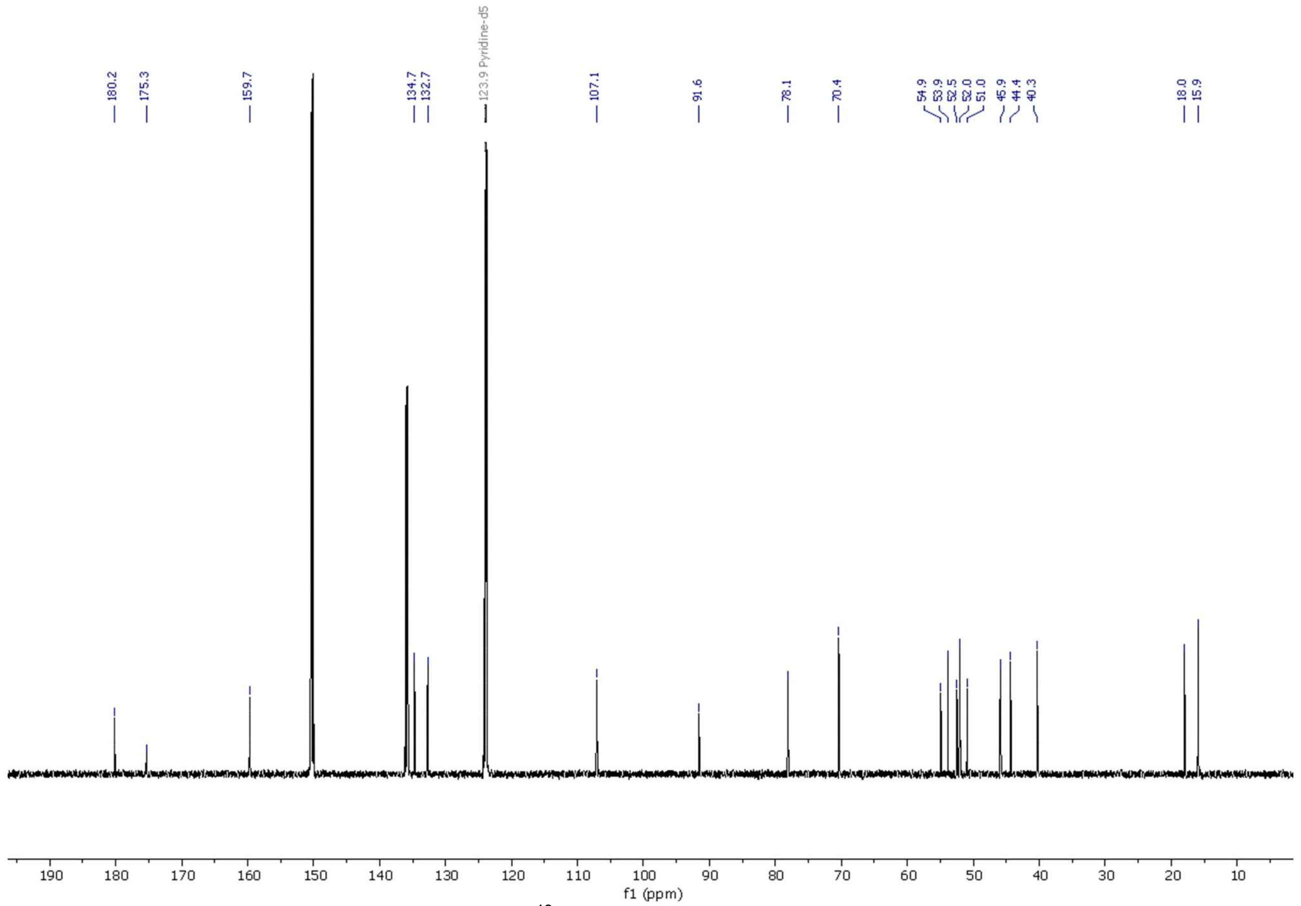

Figure N1.C ^{13}C NMR of **1** in d_5-pyridine at 151 MHz.

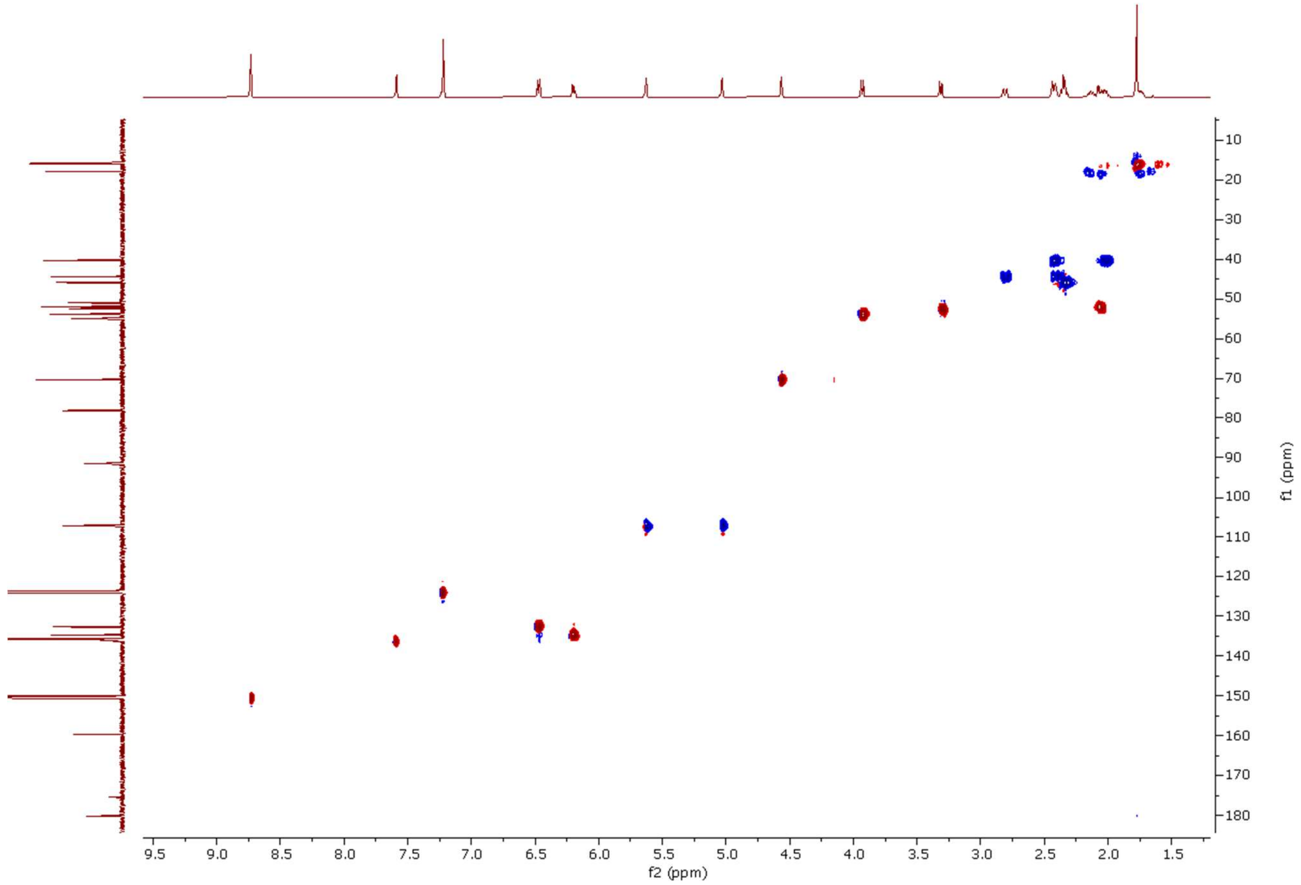

Figure N1.D HSQC NMR of **1** in d_5-pyridine.

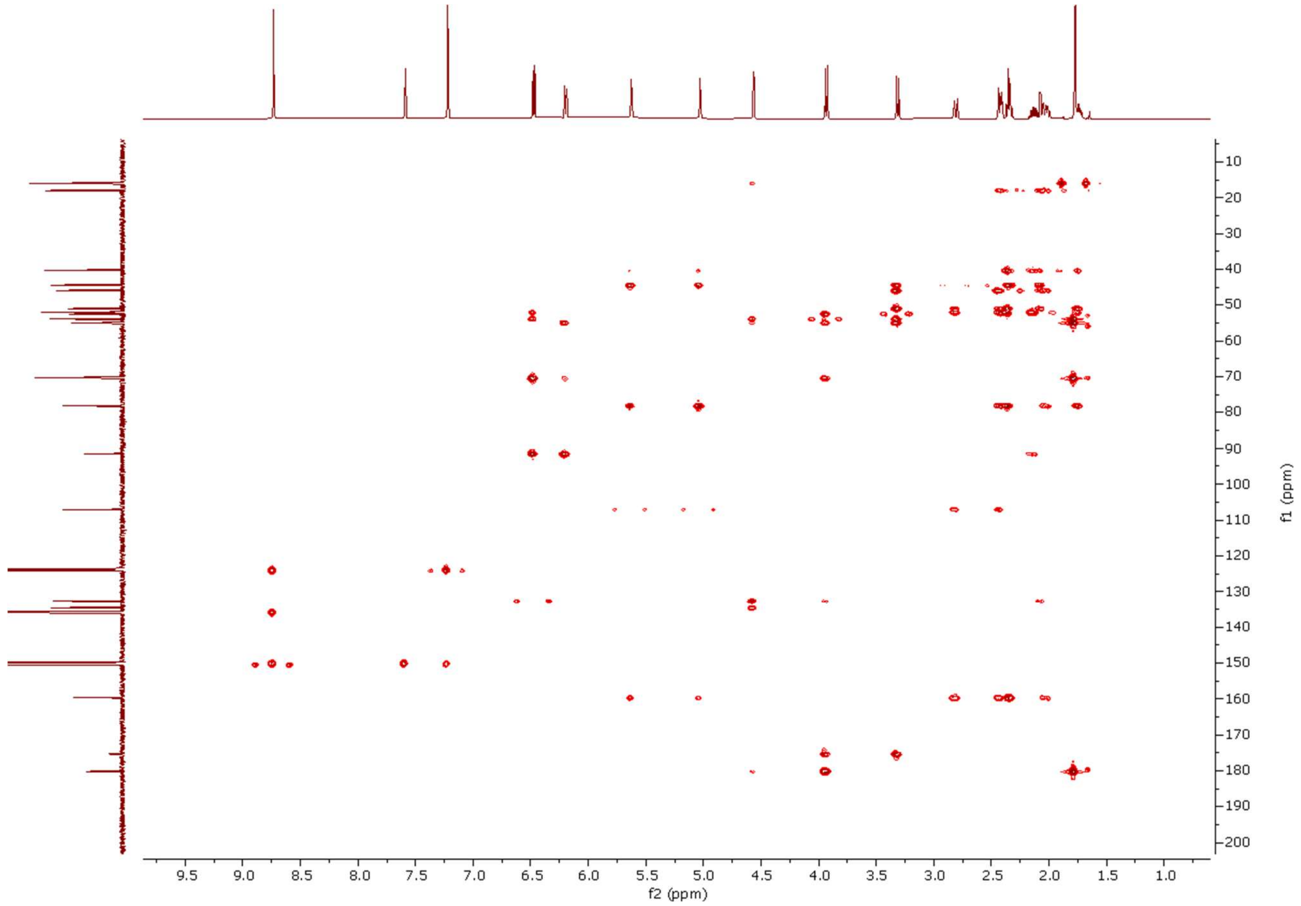

Figure N1.E HMBC NMR of **1** in d5-pyridine.

Gibberellin A_4 (2)

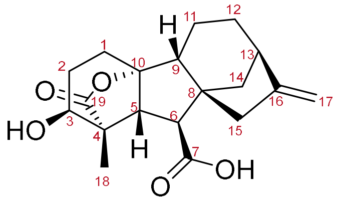

GA$_4$ (**2**)
Chemical Formula: $C_{19}H_{24}O_5$
Exact Mass: 332.1624

	Reference in C_5D_5N[41] (only δ_C reported)	Measured in C_5D_5N, 298K		Measured in DMSO-D_6, 298K	
**Pos.**	δ_C 25.15 MHz	δ_C 151 MHz	δ_H (*J/Hz*) 600 MHz	δ_C 151 MHz	δ_H (*J/Hz*) 600 MHz
**1**	28.2	28.6	2.01, overlapped	26.8	1.73, overlapped<br>1.86, m
**2**	29.2	29.7	2.01, overlapped	28.0	1.55, overlapped<br>1.70, overlapped
**3**	70.0	70.3	4.15, br s	68.5	3.54, br d (3.5)
**4**	55.5	56.0	-	54.2	-
**5**	51.9	52.3	3.90, d (10.8)	50.5	3.03, d (10.8)
**6**	52.8	53.4	3.18, d (10.8)	51.6	2.40, d (10.8)
**7**	175.2	175.9	-	173.5	-
**8**	51.5	51.9	-	50.4	-
**9**	54.0	54.4	1.91, overlapped	52.8	1.78, dd (9.5, 8.4)
**10**	94.1	94.6	-	93.6	-
**11**	16.5	16.9	1.54, m<br>1.83, overlapped	15.7	1.49, m
**12**	31.8	32.2	1.37, m<br>2.02, overlapped	31.0	1.29, m<br>2.02, m
**13**	39.4	39.8	2.57, dd (6.2, 6.2)	38.4	2.57, dd (7.8, 5.6)
**14**	37.4	37.7	1.83, overlapped<br>1.92, overlapped	36.6	1.55, overlapped<br>1.59, dd (11.2, 2.5)
**15**	45.0	45.5	2.33, br d (15.3)<br>2.53, br d (15.3)	43.9	1.96, ddd (15.4, 2.5, 2.5)<br>2.11, br d (15.4)
**16**	157.7	158.3	-	156.9	-
**17**	107.2	107.6	4.88, br s<br>5.00, br s	107.1	4.84, br s<br>4.95, br s
**18**	15.5	16.1	1.66, s	14.7	0.99
**19**	179.0	179.6	-	178.1	-

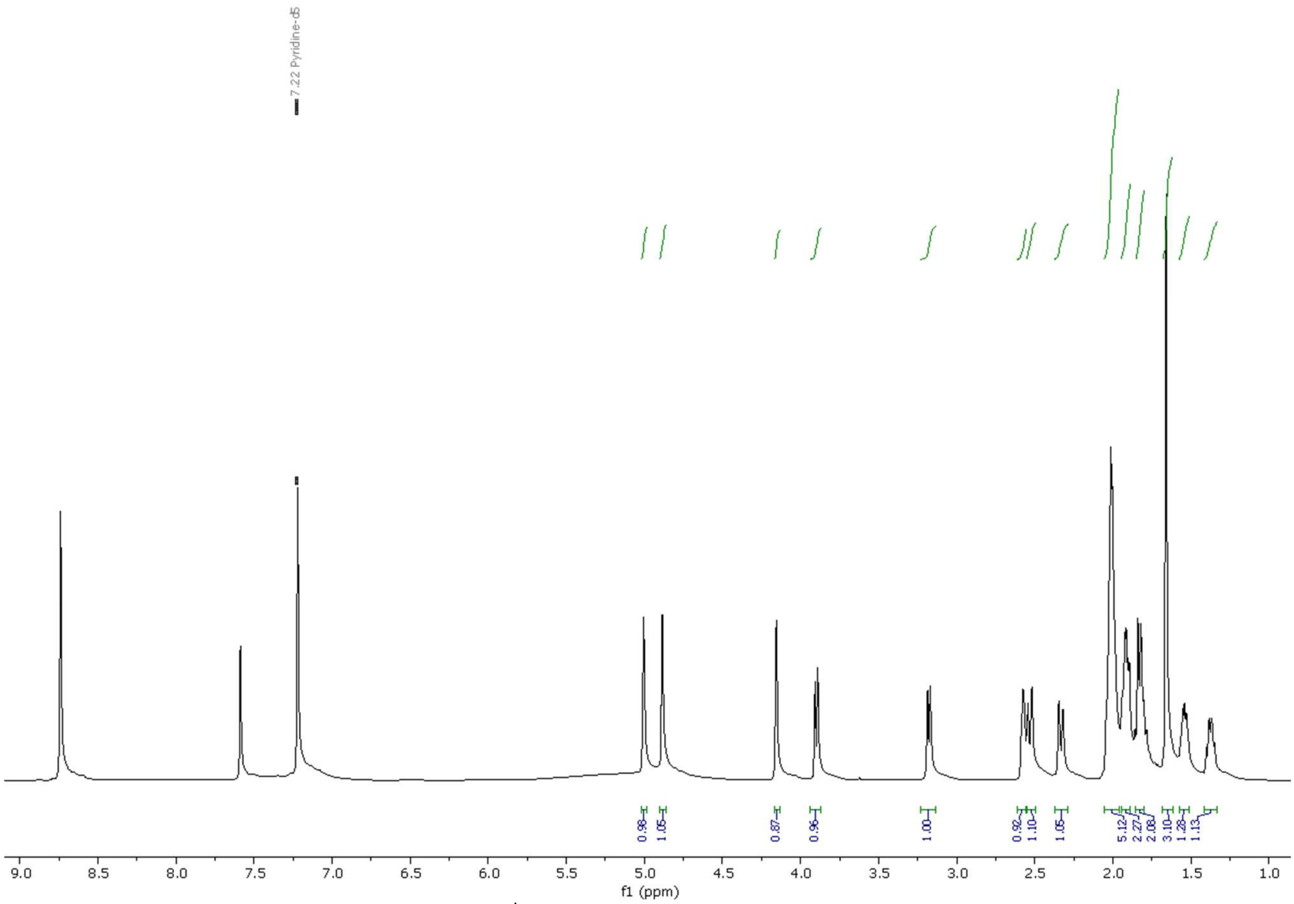

Figure N2.A ^{1}H NMR of **2** in d5-pyridine at 600 MHz.

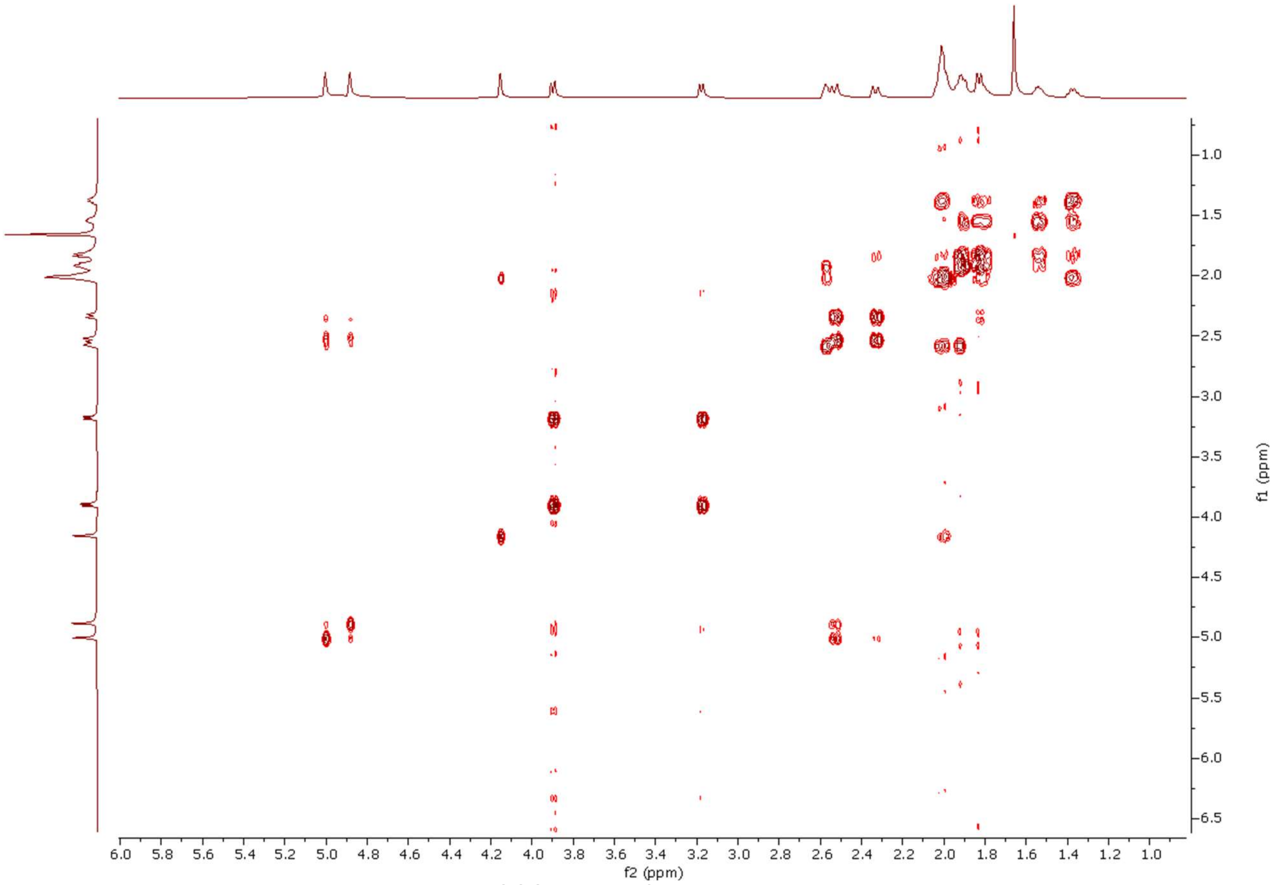

Figure N2.B COSY NMR of **2** in d5-pyridine at 600 MHz.

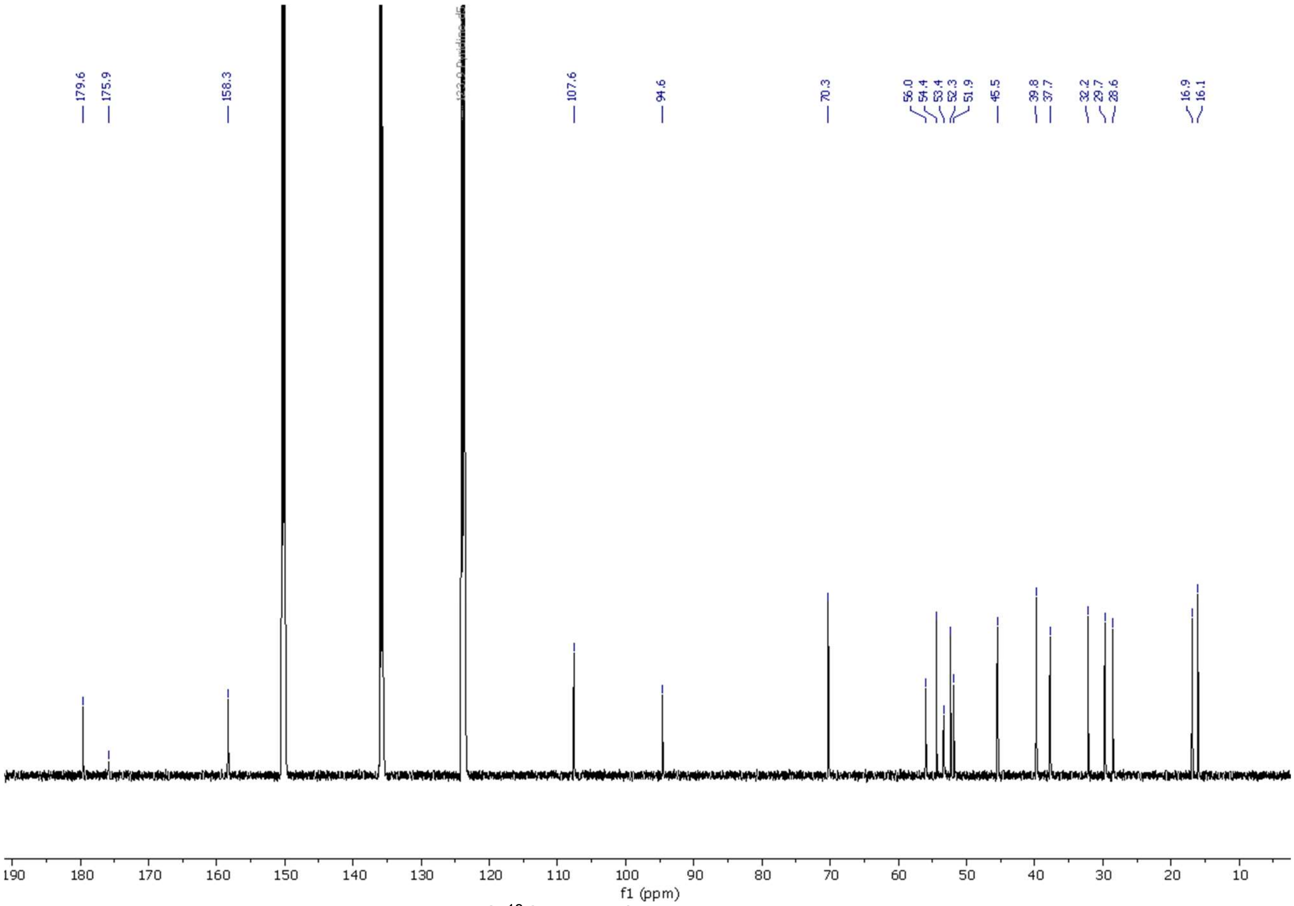

Figure N2.C ^{13}C NMR of **2** in d_5-pyridine at 151 MHz.

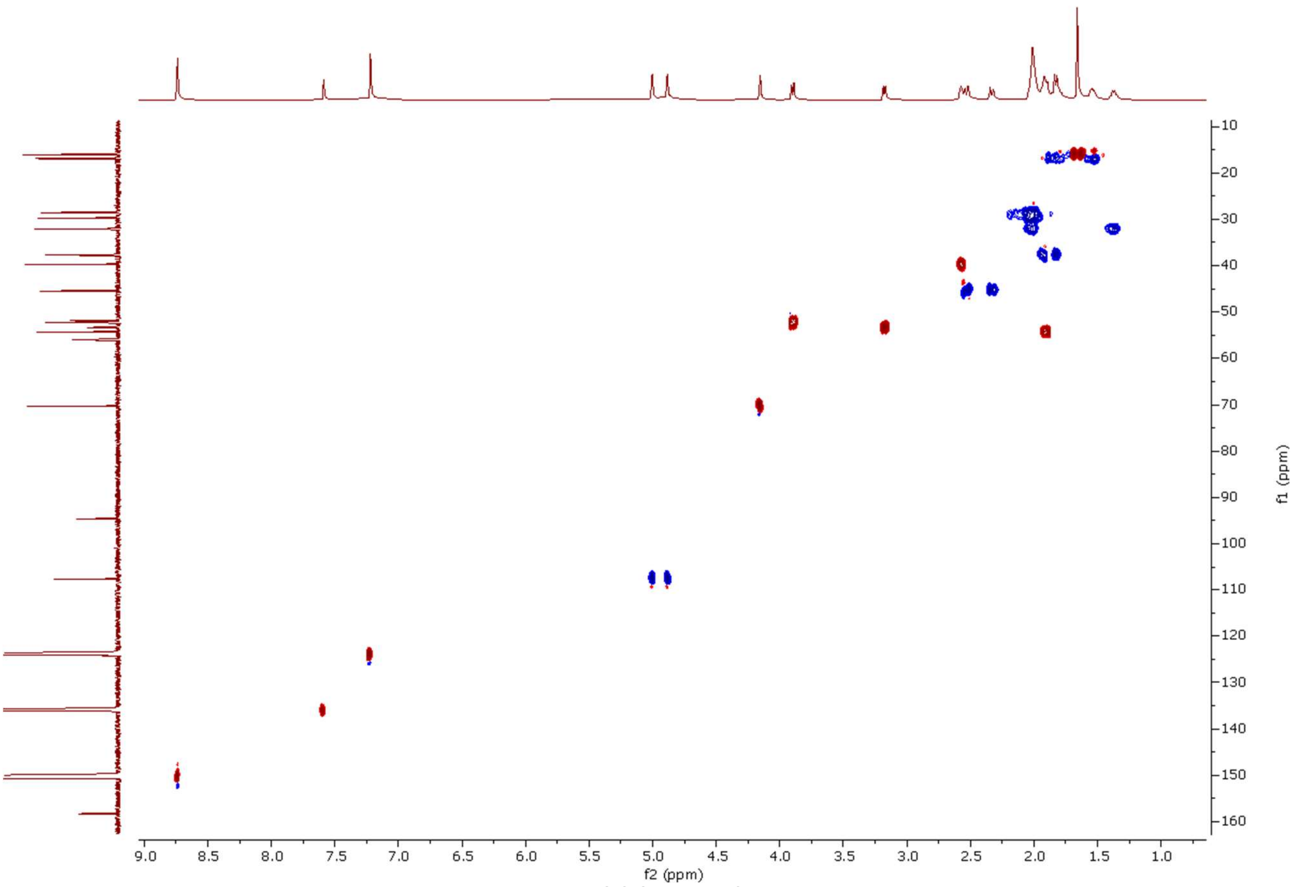

Figure N2.D. HSQC NMR of **2** in d_5-pyridine.

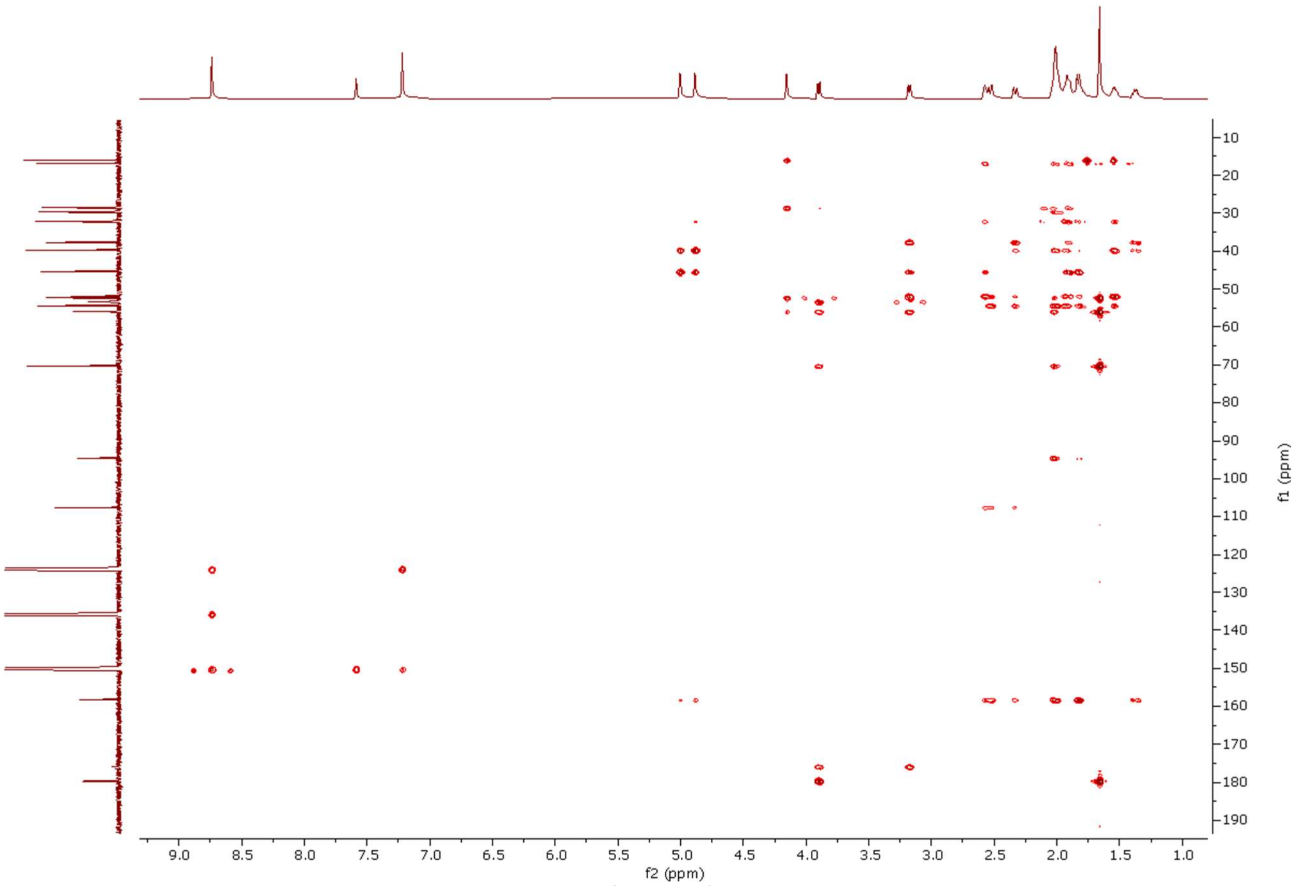

Figure N2.E HMBC NMR of **2** in d_5-pyridine.

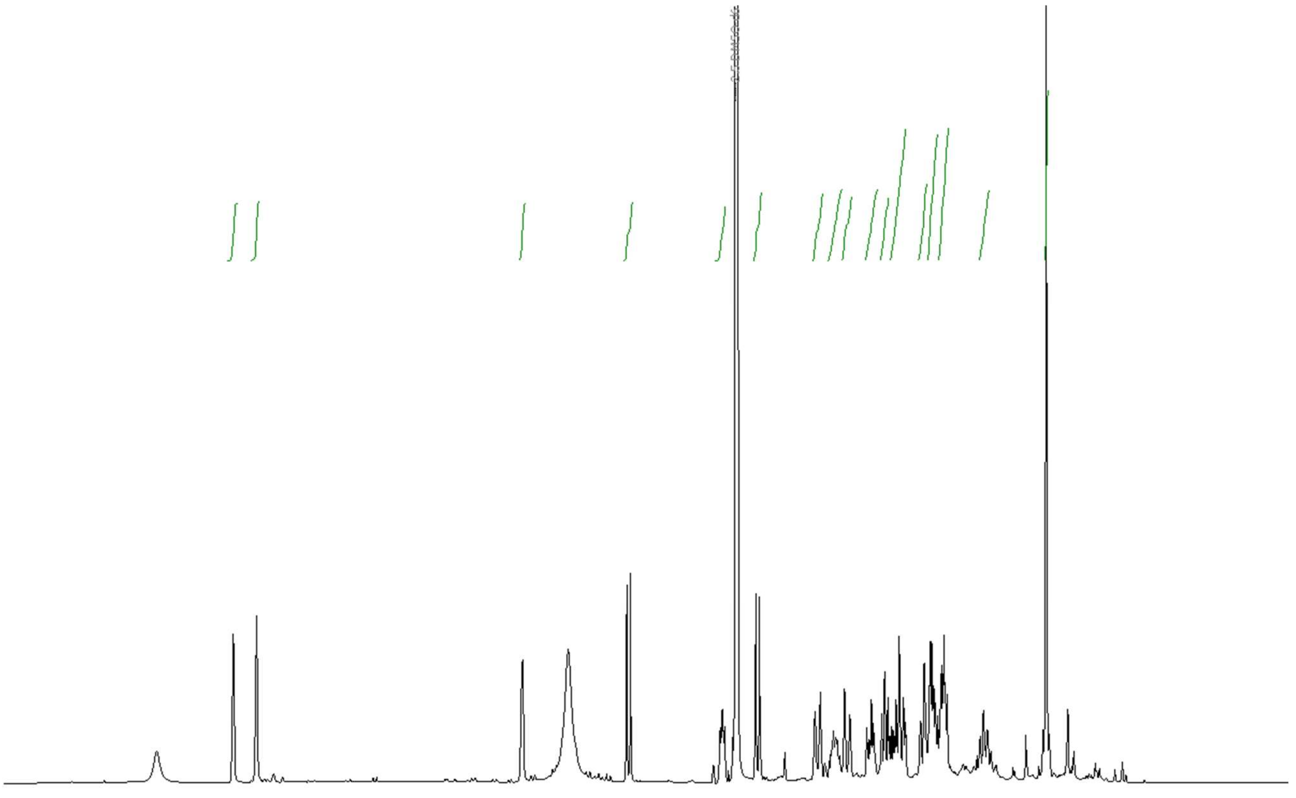

Figure N2.F ^{1}H NMR of **2** in DMSO-d_6 at 600 MHz.

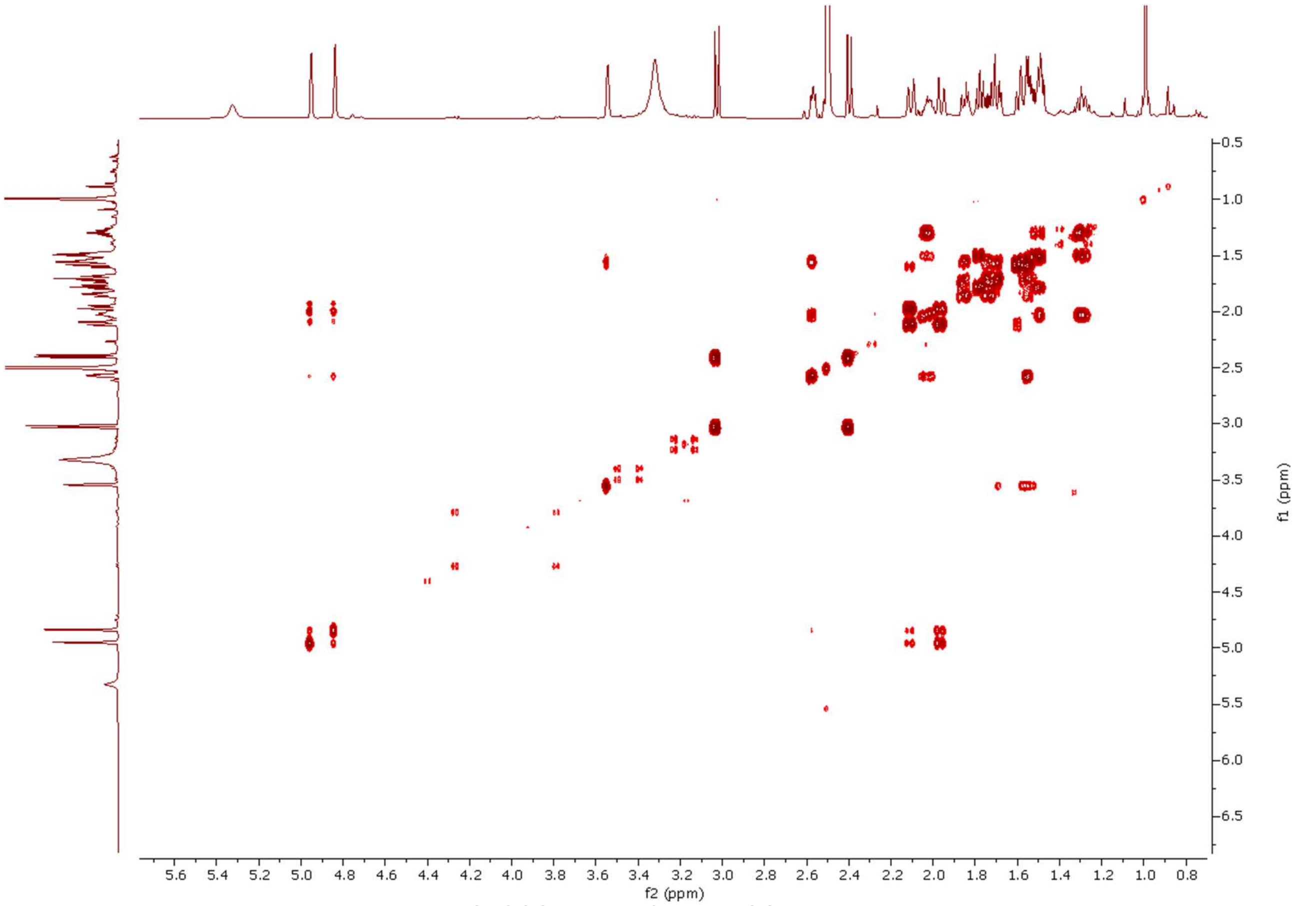

Figure N2.G. COSY NMR of **2** in DMSO-d$_6$ at 600 MHz.

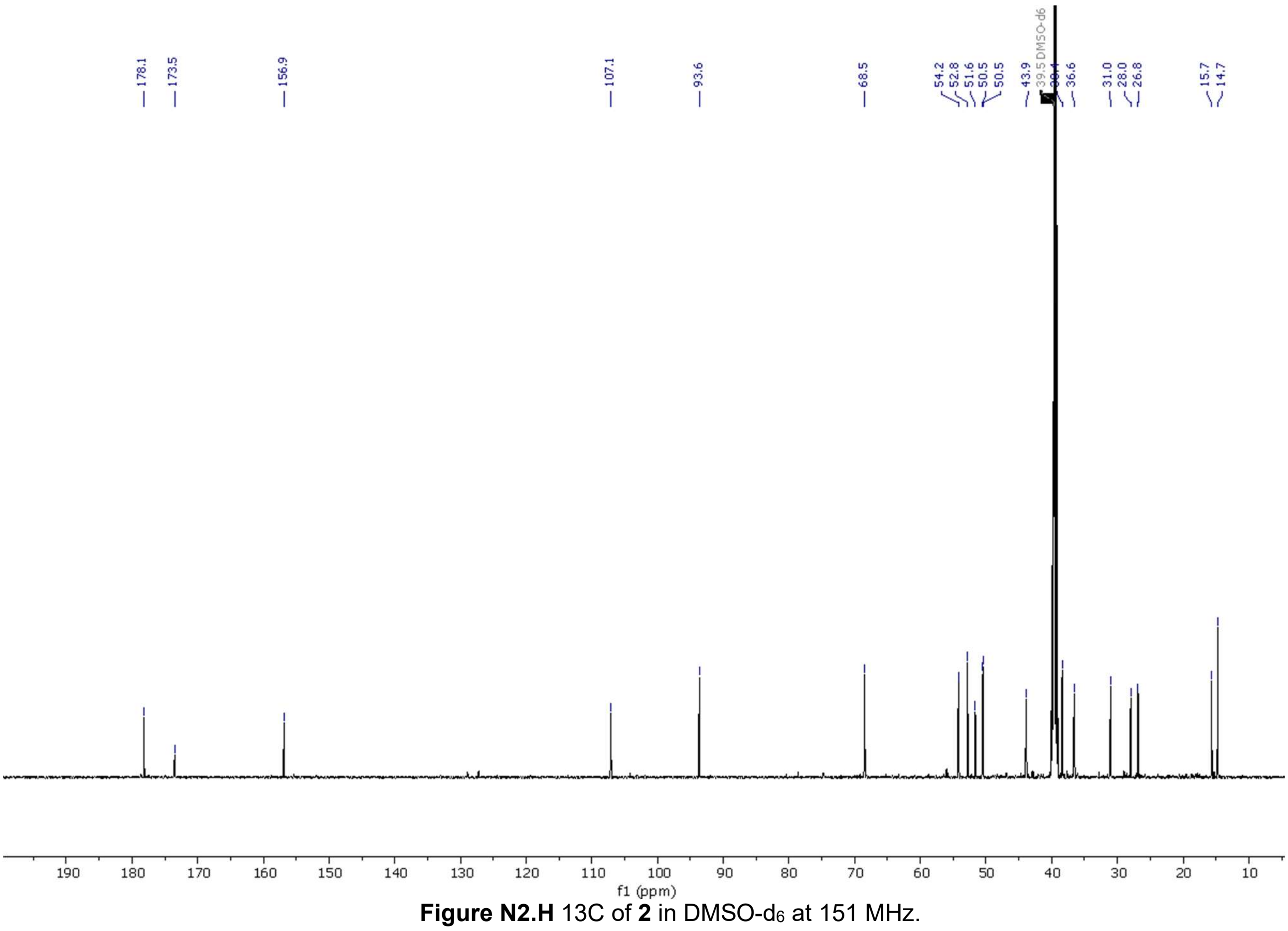

Figure N2.H 13C of **2** in DMSO-d$_6$ at 151 MHz.

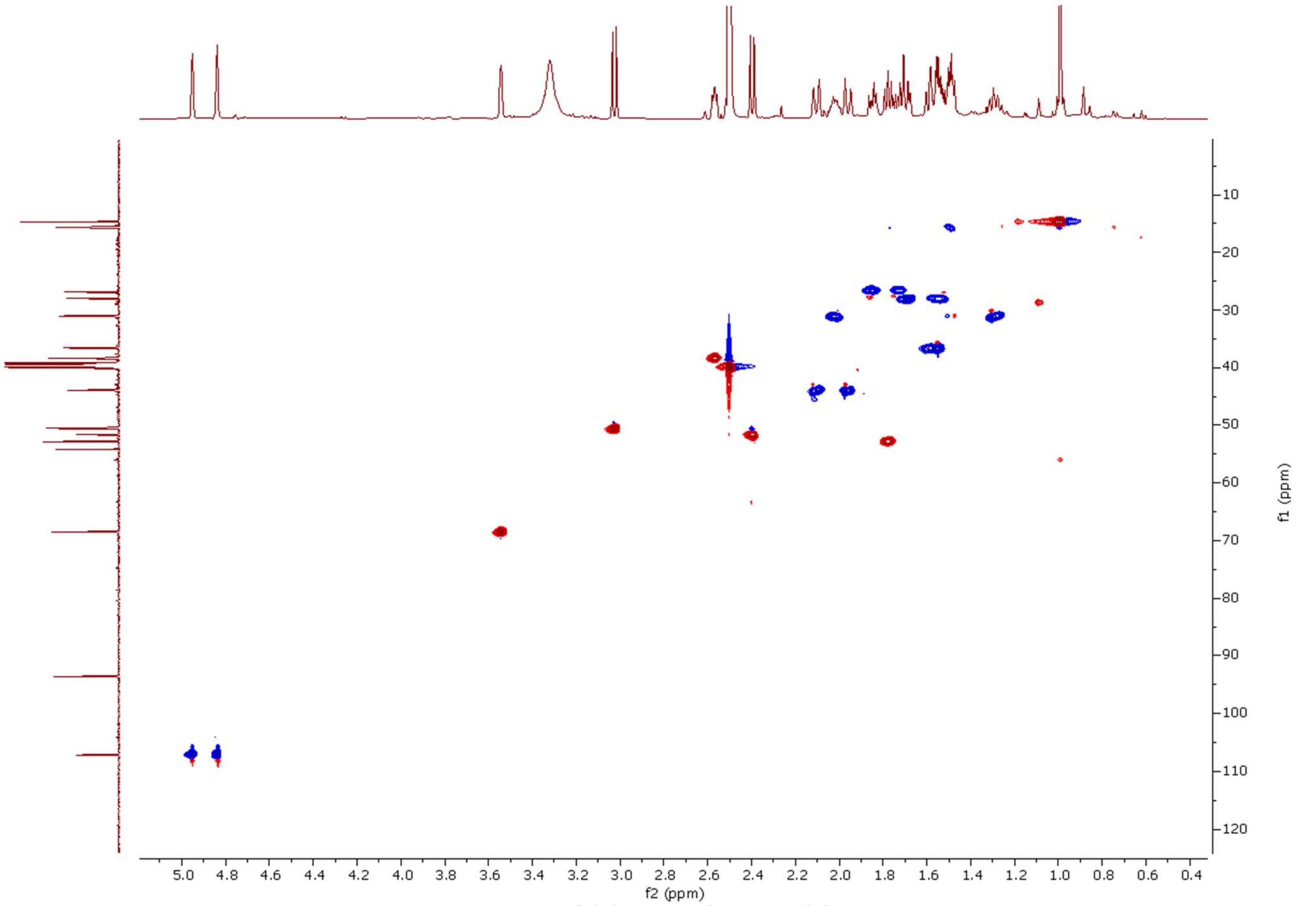

Figure N2.I HSQC NMR of **2** in DMSO-d$_6$.

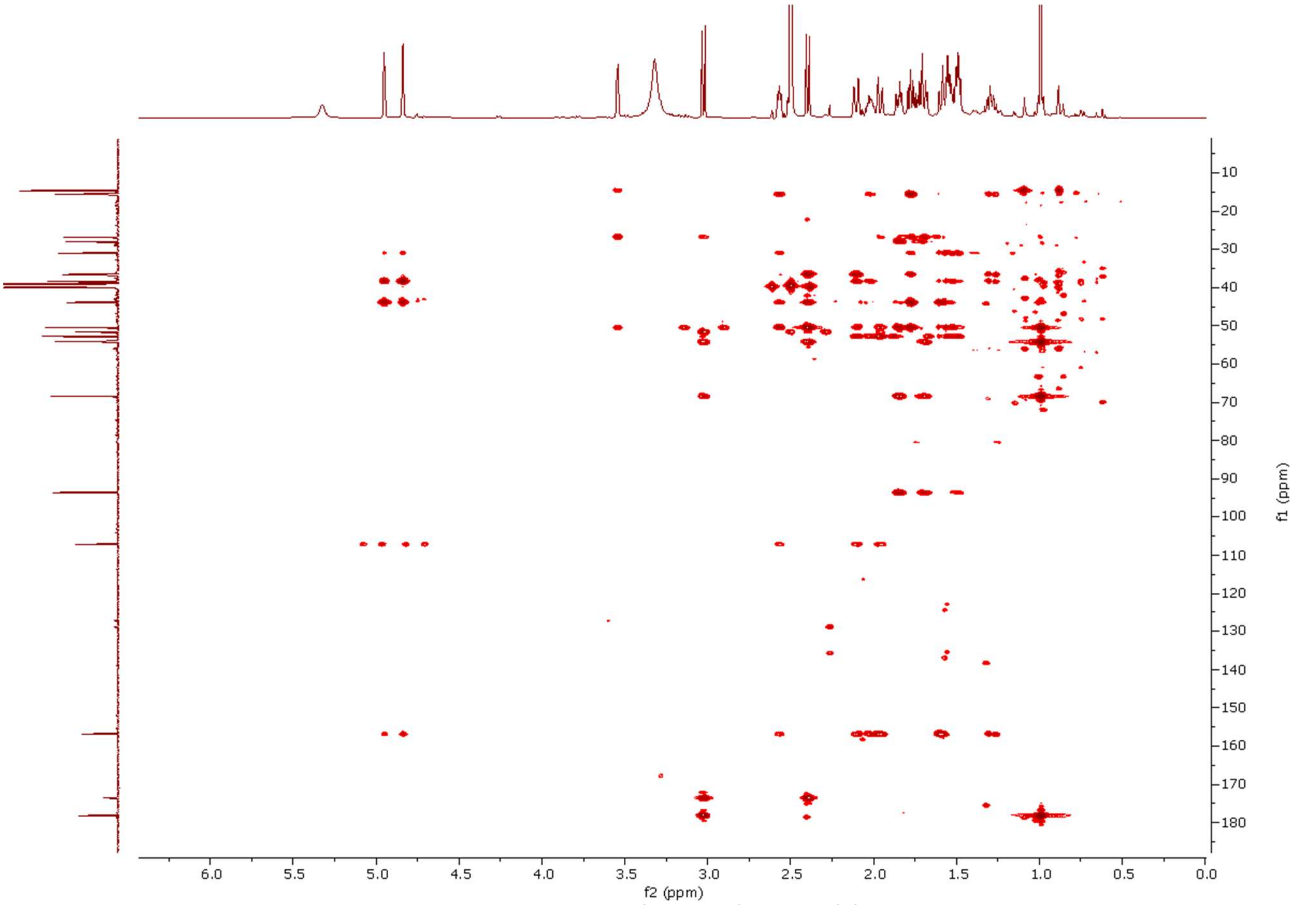

Figure N2.J HMBC NMR of **2** in DMSO-d$_6$.

Gibberellin A_1 (4)

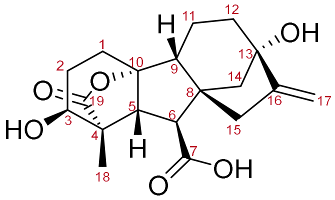

GA$_1$ (**4**)
Chemical Formula: $C_{19}H_{24}O_6$
Exact Mass: 348.1573

	**Reference in C_5D_5N [41] (only δ_C reported)**	**Measured in C_5D_5N, 298K**	
**Pos.**	δ_C 25.15 MHz	δ_C 126 MHz	δ_H *(J/Hz)* 500 MHz
**1**	28.1	28.7	2.03, overlapped
**2**	29.2	29.9	2.03, overlapped
**3**	70.0	70.5	4.16, br s
**4**	55.5	56.2	-
**5**	52.5	53.1	3.95, d (10.2)
**6**	52.7	53.3	3.23, d (10.2)
**7**	175.2	175.9	-
**8**	49.8	50.3	-
**9**	53.5	54.0	2.04, overlapped
**10**	93.9	94.6	-
**11**	18.0	18.5	1.71, m<br>2.15, m
**12**	39.9	40.6	2.04, overlapped<br>2.44, ddd (12.5, 8.5, 2.8)
**13**	77.9	78.5	-
**14**	46.3	46.9	2.34, d (10.7)<br>2.40, dd (10.7, 2.8)
**15**	44.0	44.6	2.51, br d (15.6)<br>2.86, ddd (15.6, 2.8, 2.8)
**16**	159.2	159.9	-
**17**	106.5	107.1	5.06, br s<br>5.63, br s
**18**	15.6	16.2	1.66, s
**19**	179.0	179.8	-

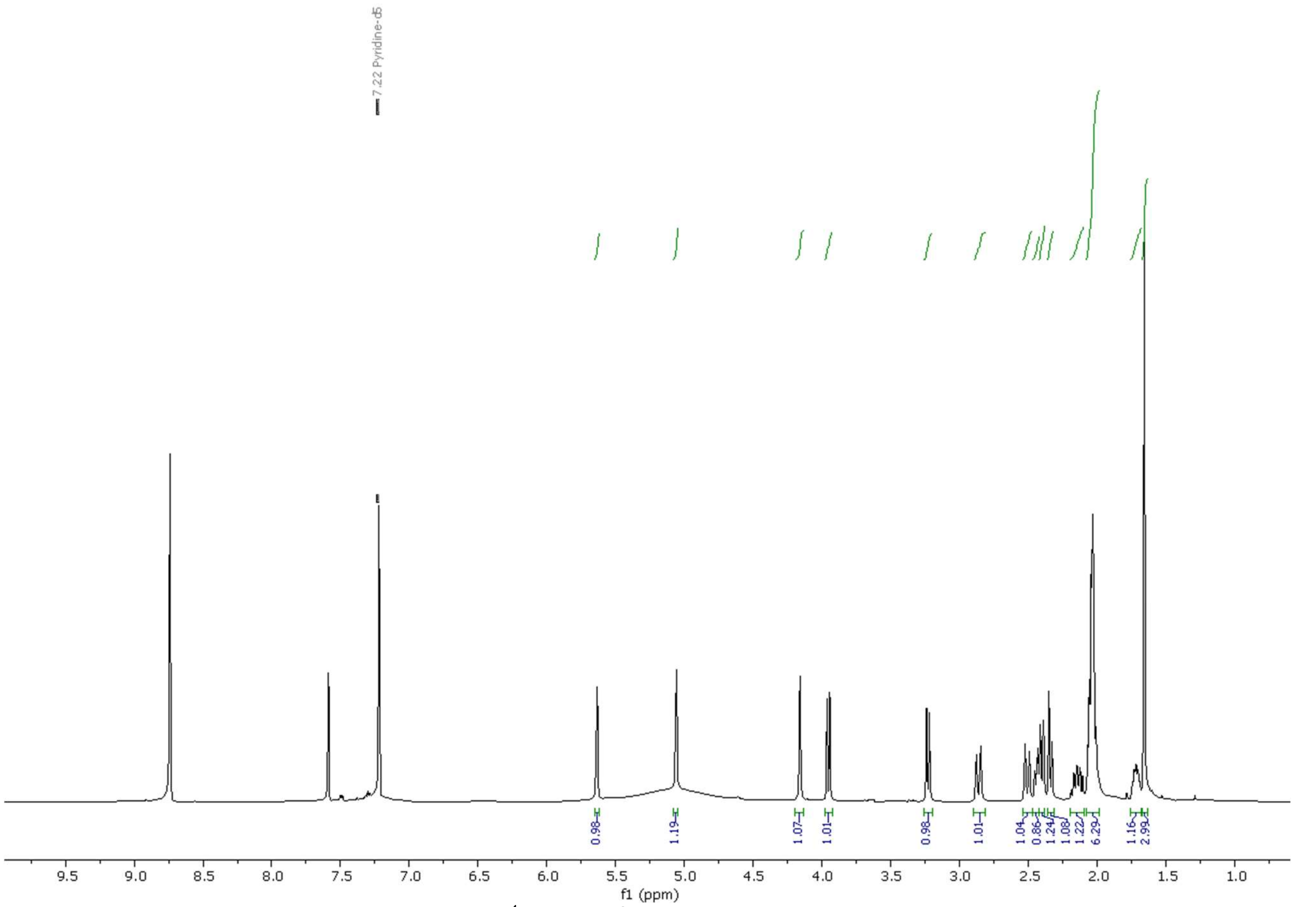

Figure N4.A ^{1}H NMR of **4** in d_5-pyridine at 500 MHz.

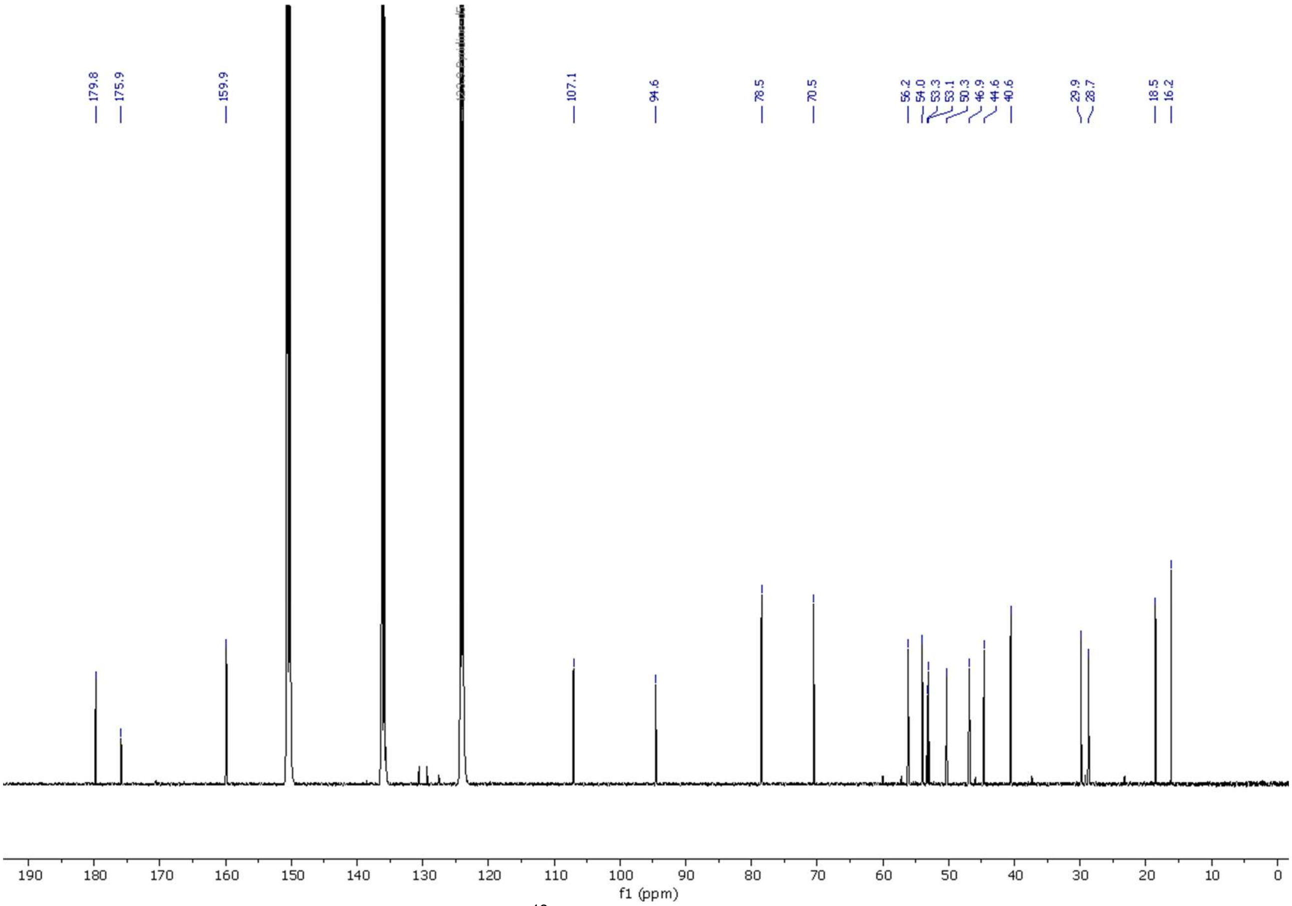

Figure N4.B ^{13}C NMR of **4** in d_5-pyridine at 126 MHz.

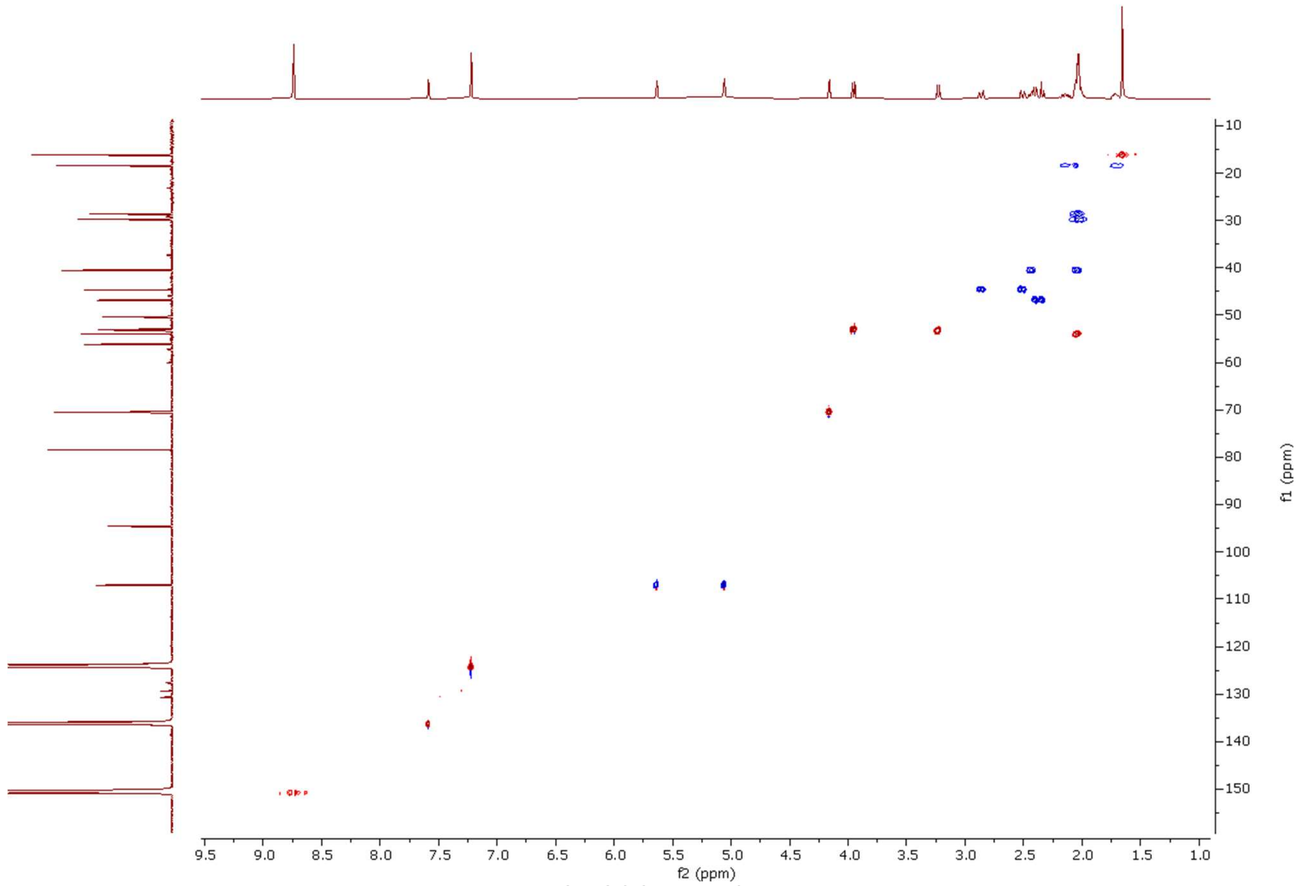

Figure N4.C HSQC NMR of **4** in d$_5$-pyridine.

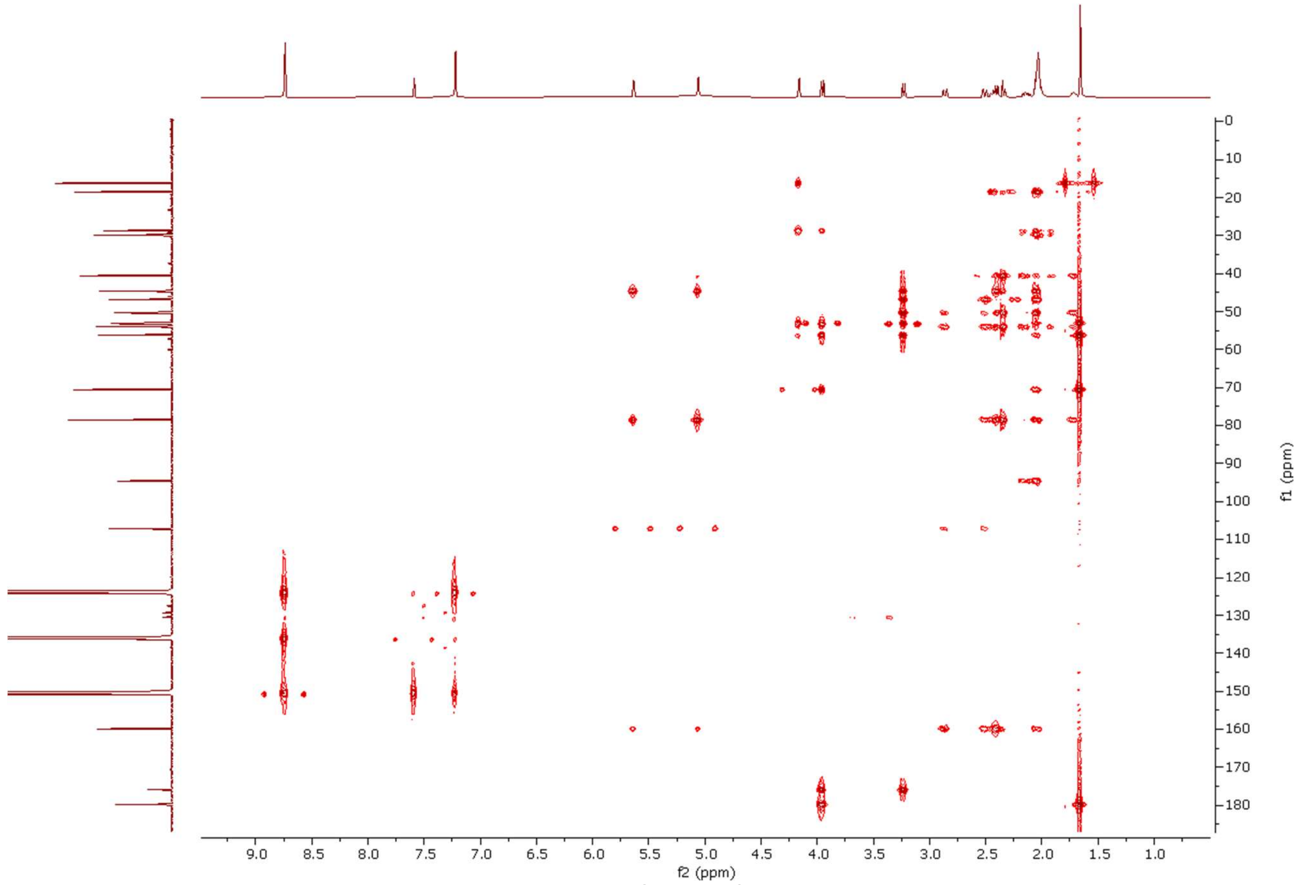

Figure N4.D HMBC NMR of **4** in d$_5$-pyridine.

Gibberellin A_{12} (5)

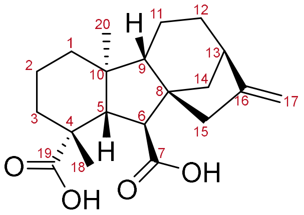

GA$_{12}$ (**5**)
Chemical Formula: $C_{20}H_{28}O_4$
Exact Mass: 332.1988

	Reference in C_5D_5N [42] (only δ_C reported)	Measured in DMSO-d$_6$, 298K	
**Pos.**	δ_C 50.1 MHz	δ_C 151 MHz	δ_H *(J/Hz)* 600 MHz
**1**	40.4	39.7	0.98, overlapped<br>1.57, overlapped
**2**	20.6	19.5	1.37, overlapped<br>1.72, m
**3**	39.5	38.0	0.94, overlapped<br>2.03, overlapped
**4**	44.6	43.8	-
**5**	57.7	56.2	1.67, d (12.5)
**6**	52.3	50.6	3.16, d (12.5)
**7**	178.0	176.3	-
**8**	49.9	48.5	-
**9**	57.4	56.9	1.20, dd (7.5, 7.5)
**10**	45.0	43.4	-
**11**	17.3	16.5	1.33, overlapped
**12**	32.5	31.8	1.32, overlapped<br>1.87, m
**13**	40.6	39.4	2.52, overlapped
**14**	38.8	38.1	1.46, dd (11.2, 5.3)<br>1.56, overlapped
**15**	47.1	45.5	2.03, overlapped
**16**	157.5	156.9	-
**17**	105.8	105.6	4.78, br s<br>4.87, br s
**18**	30.1	28.9	1.08, s
**19**	180.4	178.7	-
**20**	15.7	15.0	0.74, s

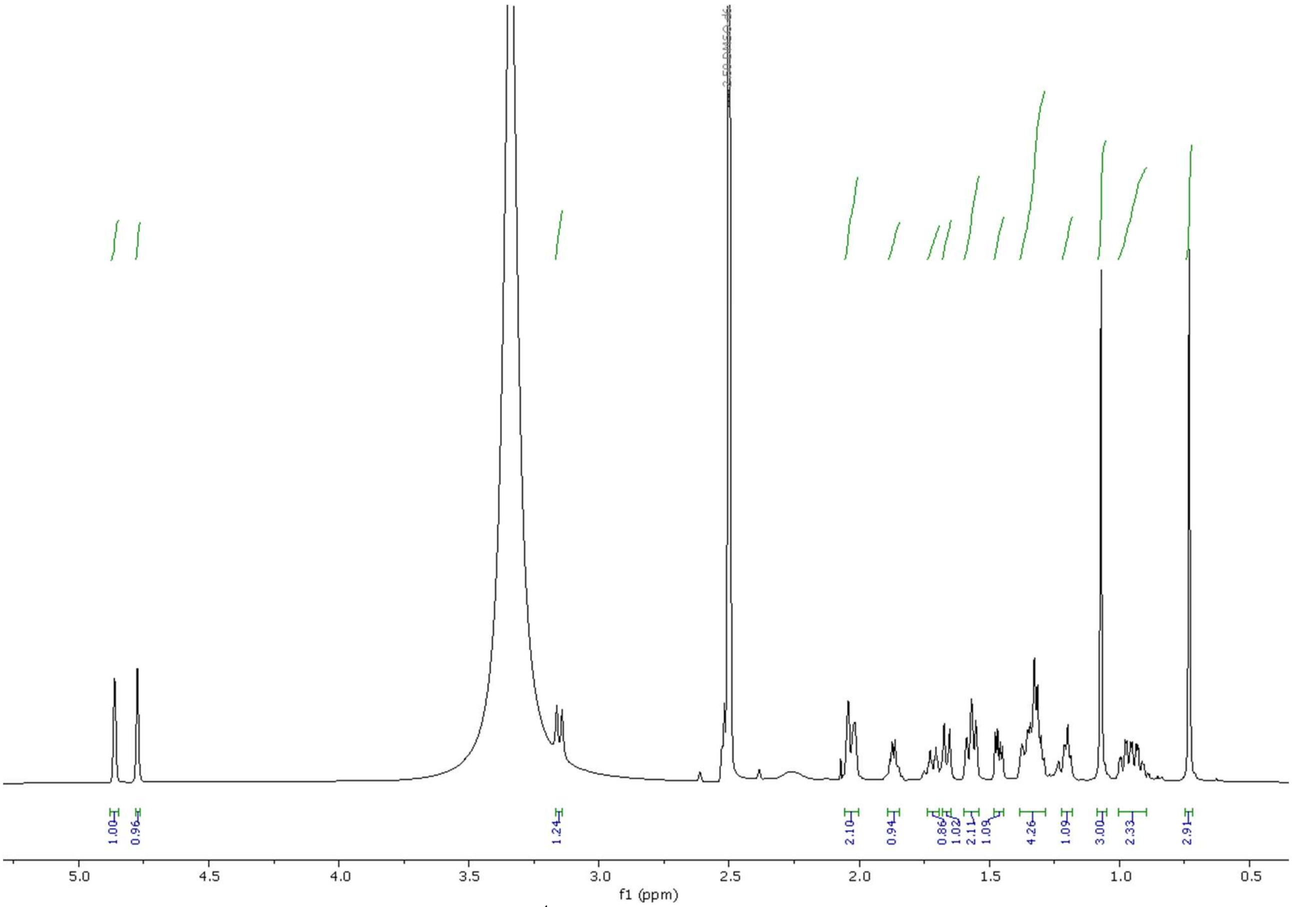

Figure N5.A ^{1}H NMR of **5** in DMSO-d$_6$ at 600 MHz.

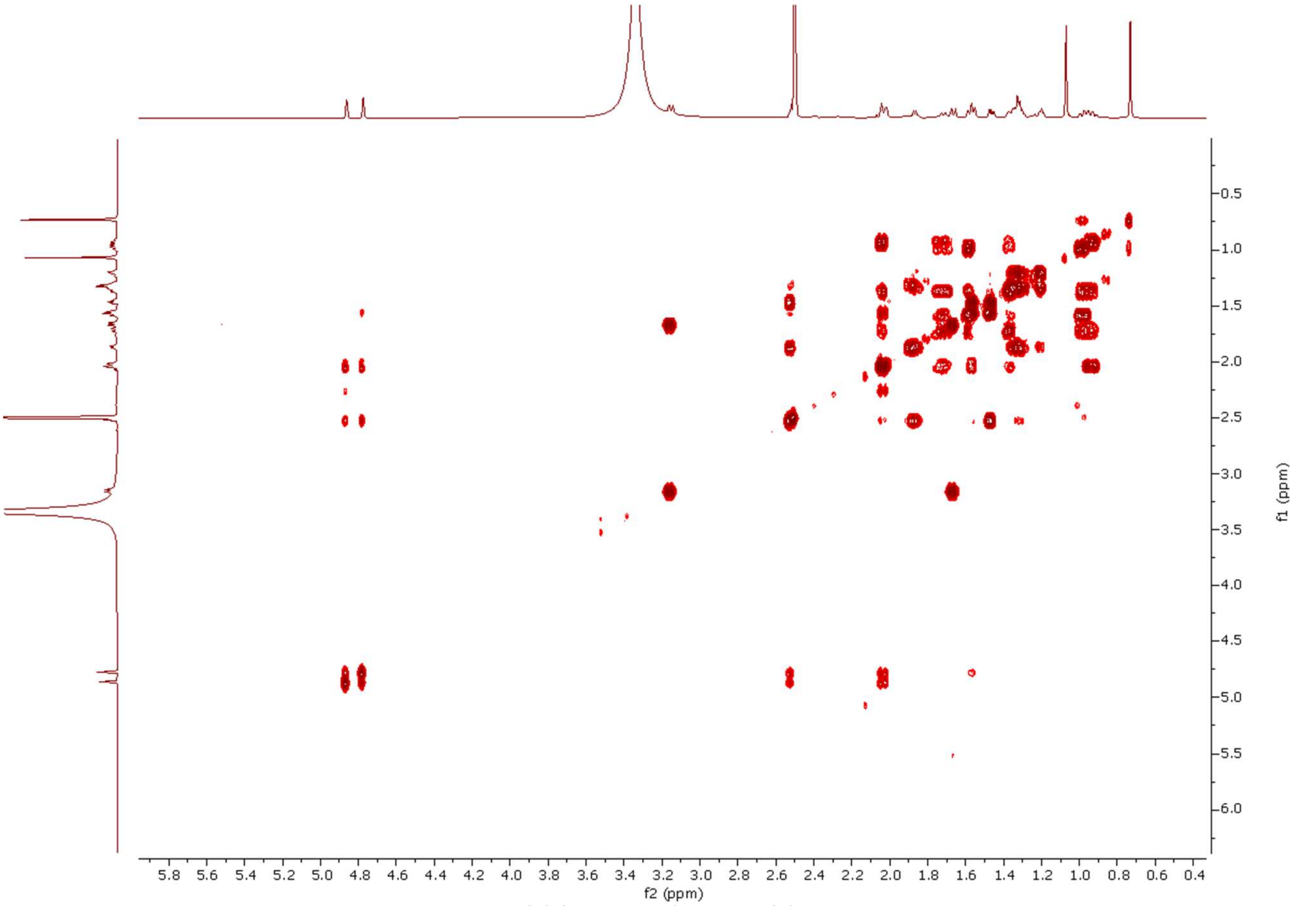

Figure N5.B COSY NMR of **5** in DMSO-d$_6$ at 600 MHz.

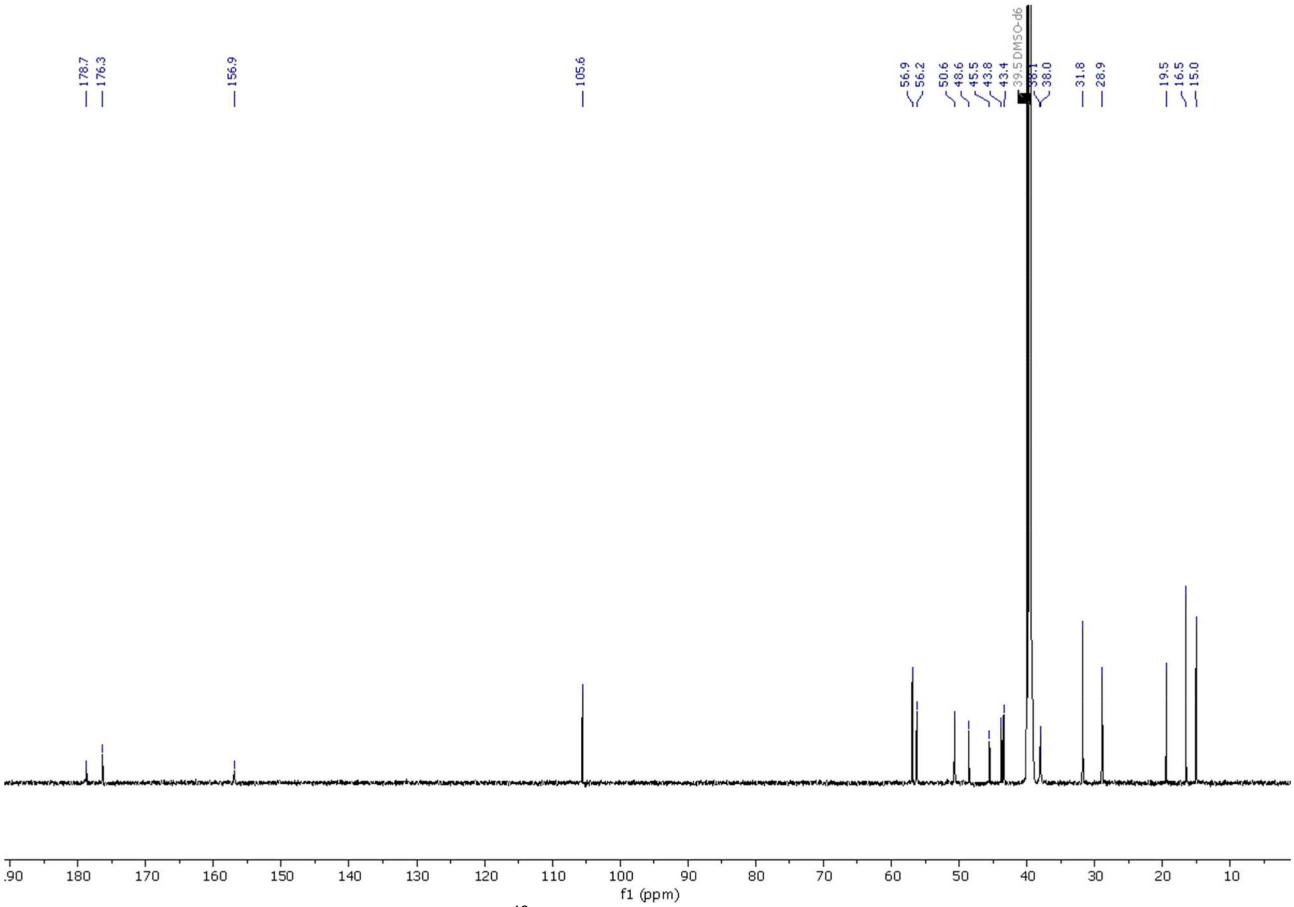

Figure N5.C ^{13}C NMR of **5** in DMSO-d_6 at 151 MHz.

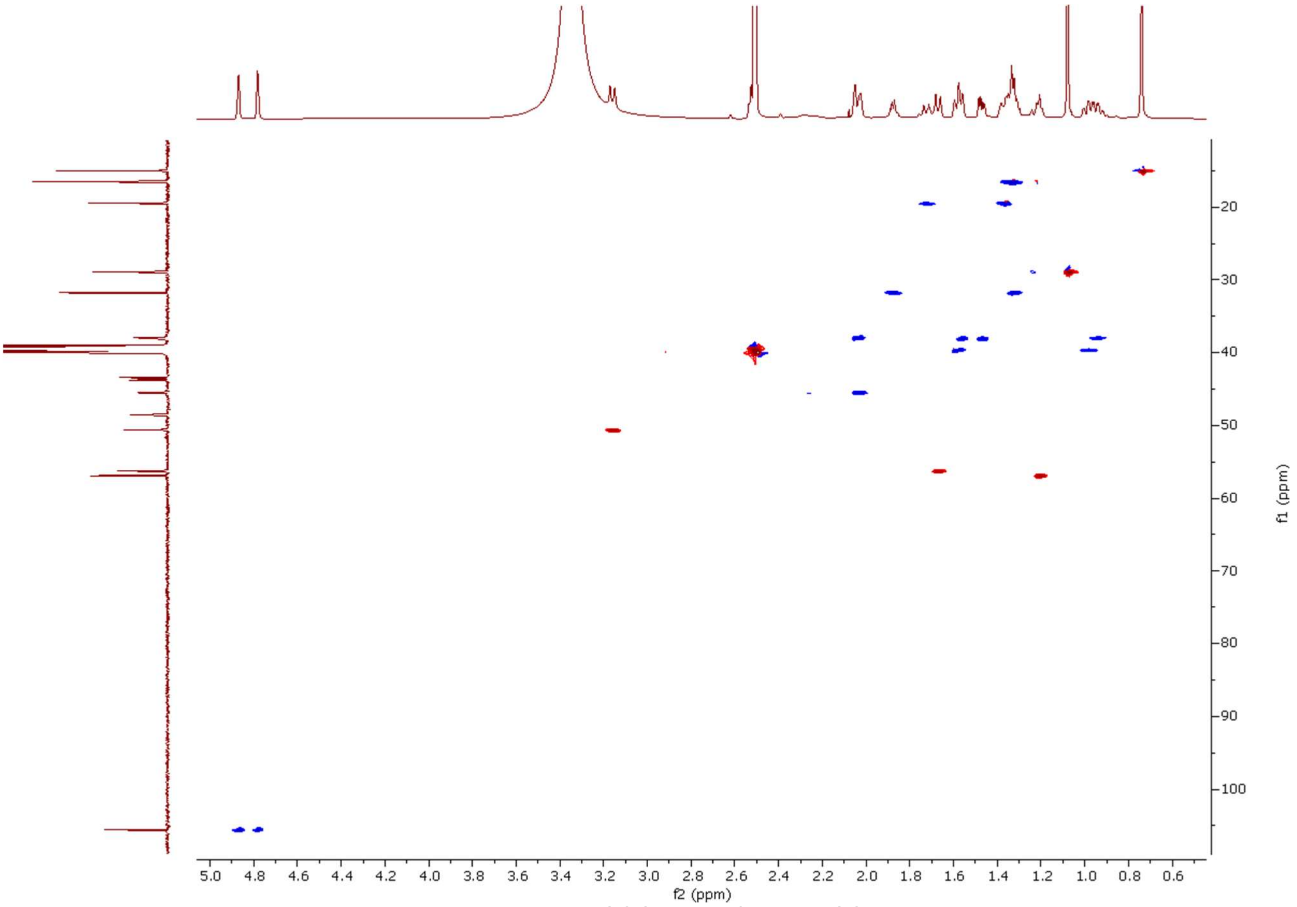

Figure N5.D HSQC NMR of **5** in DMSO-d_6.

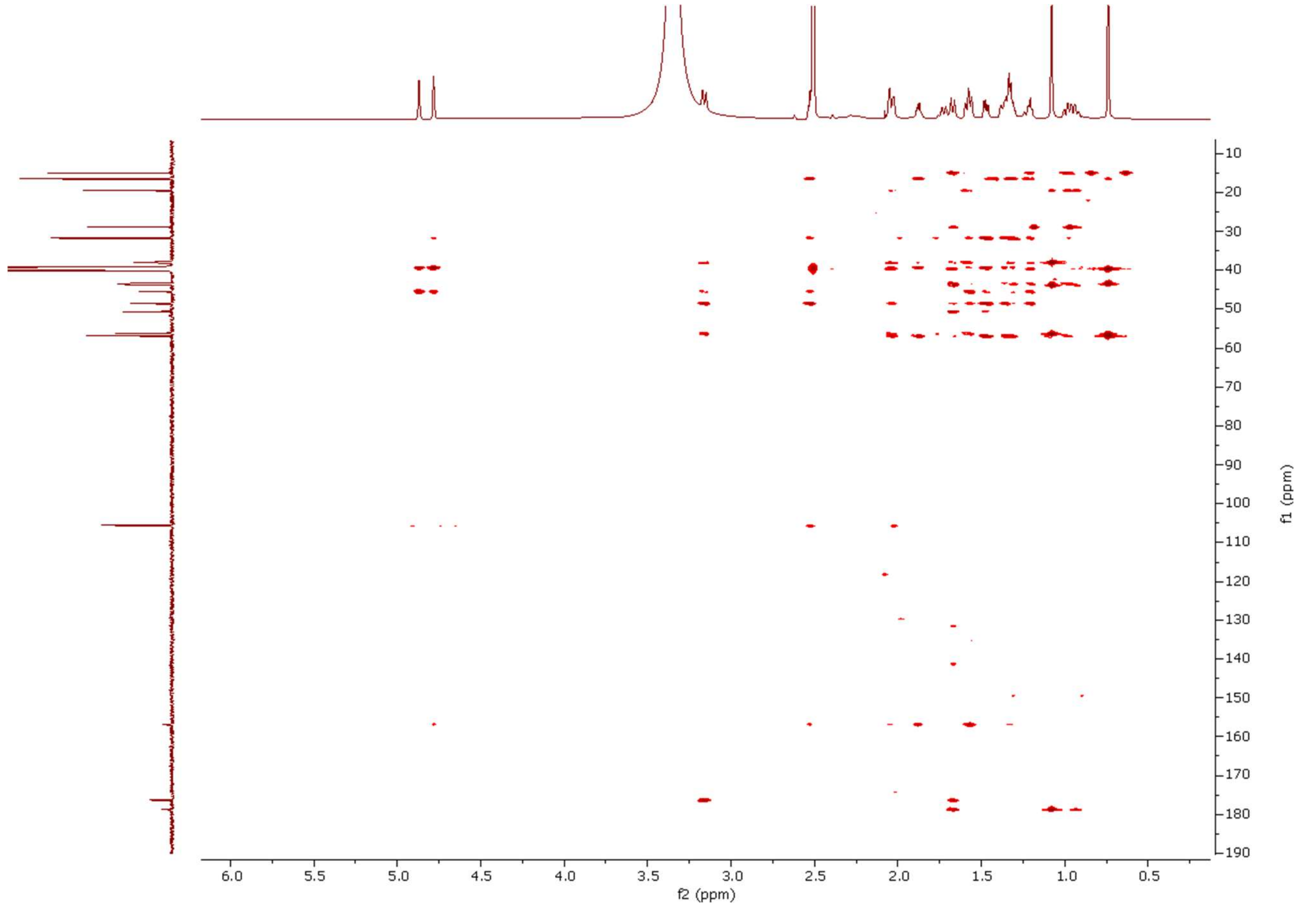

Figure N5.E HMBC NMR of **5** in DMSO-d6.

Gibberellin A_{14} (6)

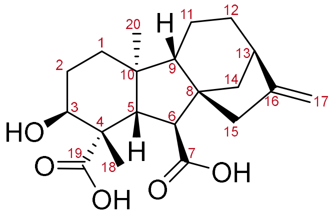

GA$_{14}$ (**6**)
Chemical Formula: $C_{20}H_{28}O_5$
Exact Mass: 348.1937

Pos.	Reference in C_5D_5N [41] (only δ_C reported)	Measured in C_5D_5N, 298K		Measured in DMSO-d$_6$, 298K		
	δ_C 25.15 MHz	δ_C 151 MHz	δ_H (J/Hz) 600 MHz	δ_C 151 MHz	δ_H (J/Hz) 600 MHz	Selected NOESY correlations to H [a]
**1**	28.4	35.0	1.60, m<br>2.02, overlapped	33.4	1.28, overlapped<br>1.36, overlapped	
**2**	34.6	28.9	a 2.11, m<br>b 2.70, overlapped	26.9	a 1.47, overlapped<br>b 1.94, m	
**3**	71.0	71.5	4.82, br dd (2.7, 2.6)	69.3	3.81, br dd (3.1, 2.5)	H-2a, H-2b, H$_3$-18
**4**	49.5	50.0	-	47.8	-	-
**5**	50.2	50.7	3.20, d (12.6)	48.8	2.17, d (12.6)	H$_3$-18, H-9, H-6
**6**	51.9	52.4	4.21, d (12.6)	50.3	3.18, d (12.6)	H$_3$-20, H-14a, H-14b
**7**	177.9	178.4	-	176.2	-	-
**8**	50.0	50.4	-	48.9	-	-
**9**	57.6	58.1	1.69, br dd (7.5, 7.5)	56.8	1.24, overlapped	H-5
**10**	44.6	45.1	-	43.5	-	-
**11**	17.3	17.6	1.40, overlapped<br>1.56, overlapped	16.4	1.33, overlapped<br>1.38, overlapped	
**12**	32.6	33.0	1.45, overlapped<br>1.83, overlapped	31.8	1.35, overlapped<br>1.82, m	
**13**	40.6	41.0	2.58, overlapped	39.6	2.53, m	H-14, H-12
**14**	39.8	40.2	1.82, overlapped<br>1.94, dd (11.2, 5.2)	39.0	a 1.48, overlapped<br>b 1.60, dd (11.3, 2.5)	
**15**	47.1	47.5	2.57, overlapped<br>2.69, overlapped	46.0	2.03, m<br>2.06, m	
**16**	157.3	157.8	-	156.2	-	-
**17**	105.7	106.1	4.89, br s<br>4.96, br s	105.7	4.80, br s<br>4.87, br s	
**18**	25.4	25.9	2.05, s	24.1	1.08, s	H-5, H-3
**19**	180.6	181.2	-	178.9	-	-
**20**	15.5	16.0	1.23, s	14.9	0.73, s	H-14b, H-2b, H-6

[a] Key NOESY correlations are shown in blue text.

Yellow highlighted data are possibly swapped assignments in the referenced data. Our assignments are supported by HMBC correlations.

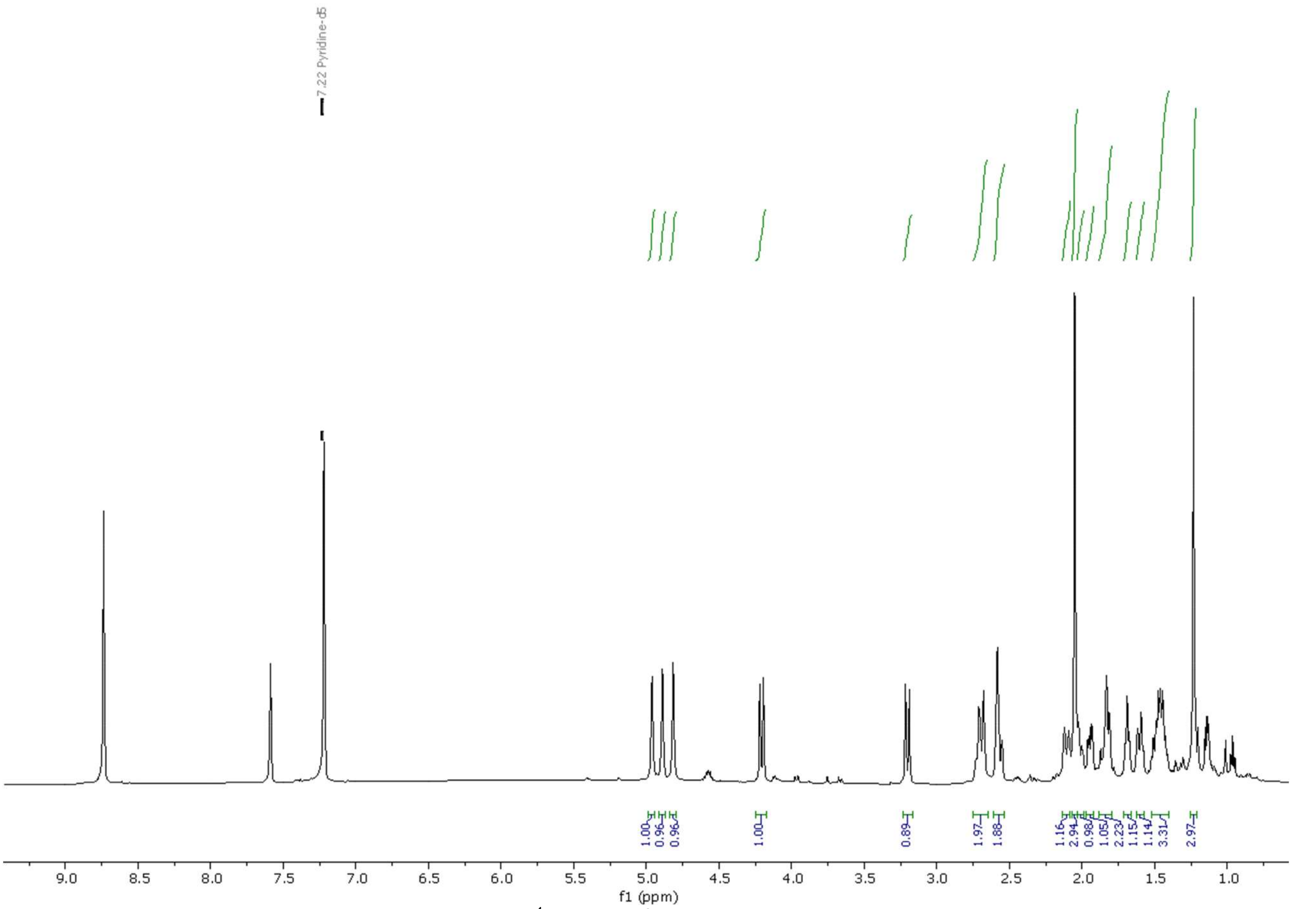

Figure N6.A ^{1}H NMR of **6** in pyridine-d$_5$ at 600 MHz.

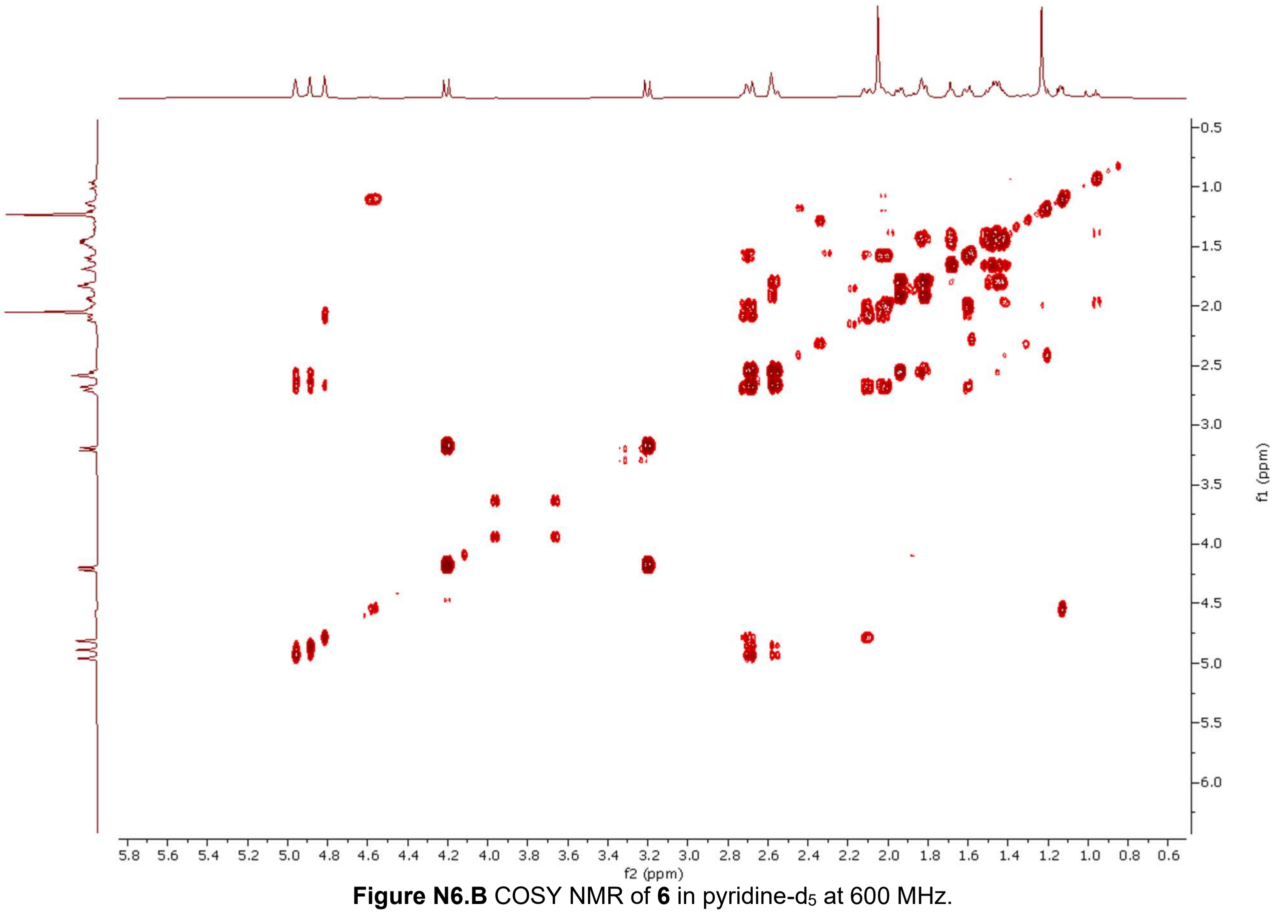

Figure N6.B COSY NMR of **6** in pyridine-d$_5$ at 600 MHz.

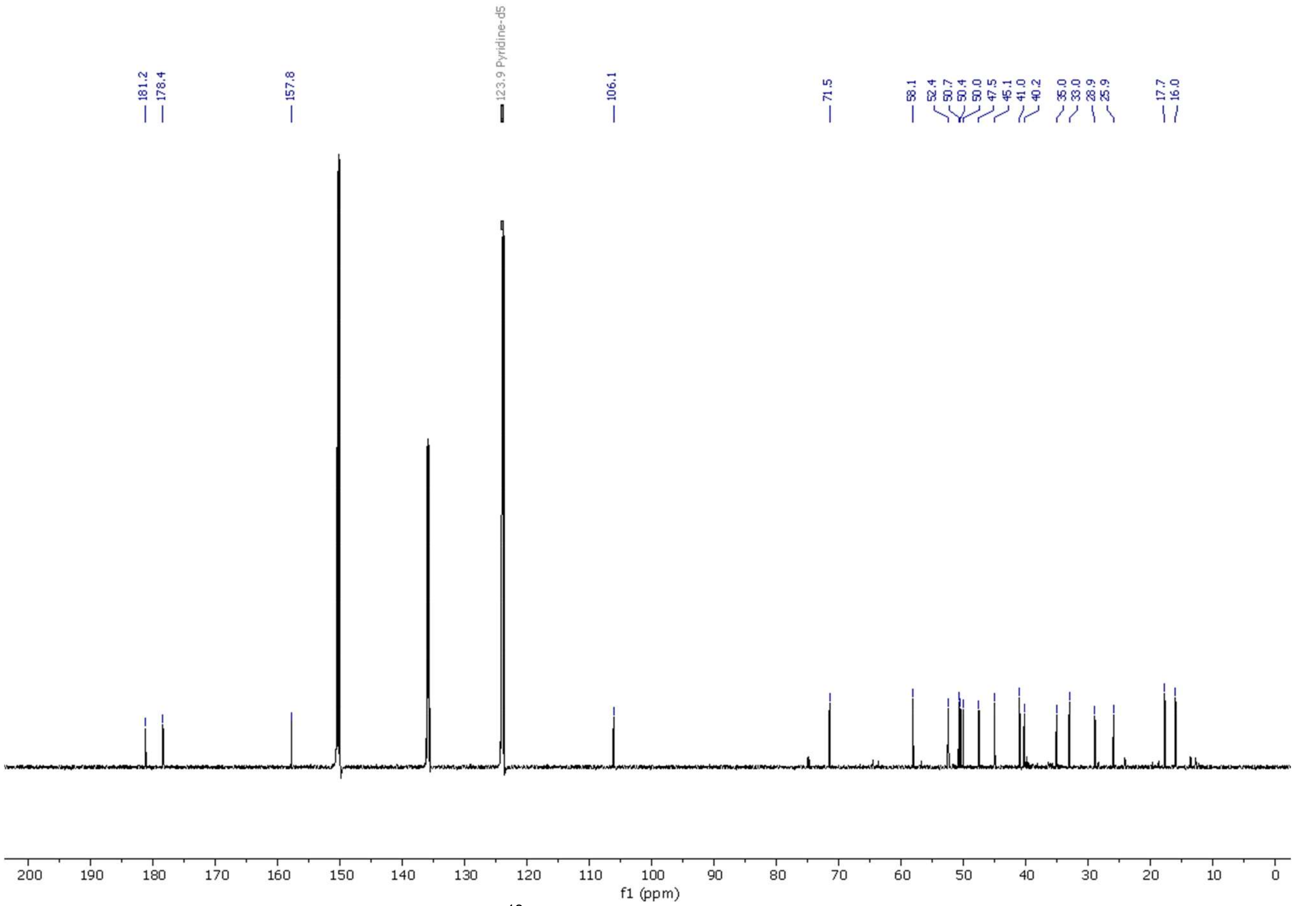

Figure N6.C ^{13}C NMR of **6** in pyridine-d_5 at 151 MHz.

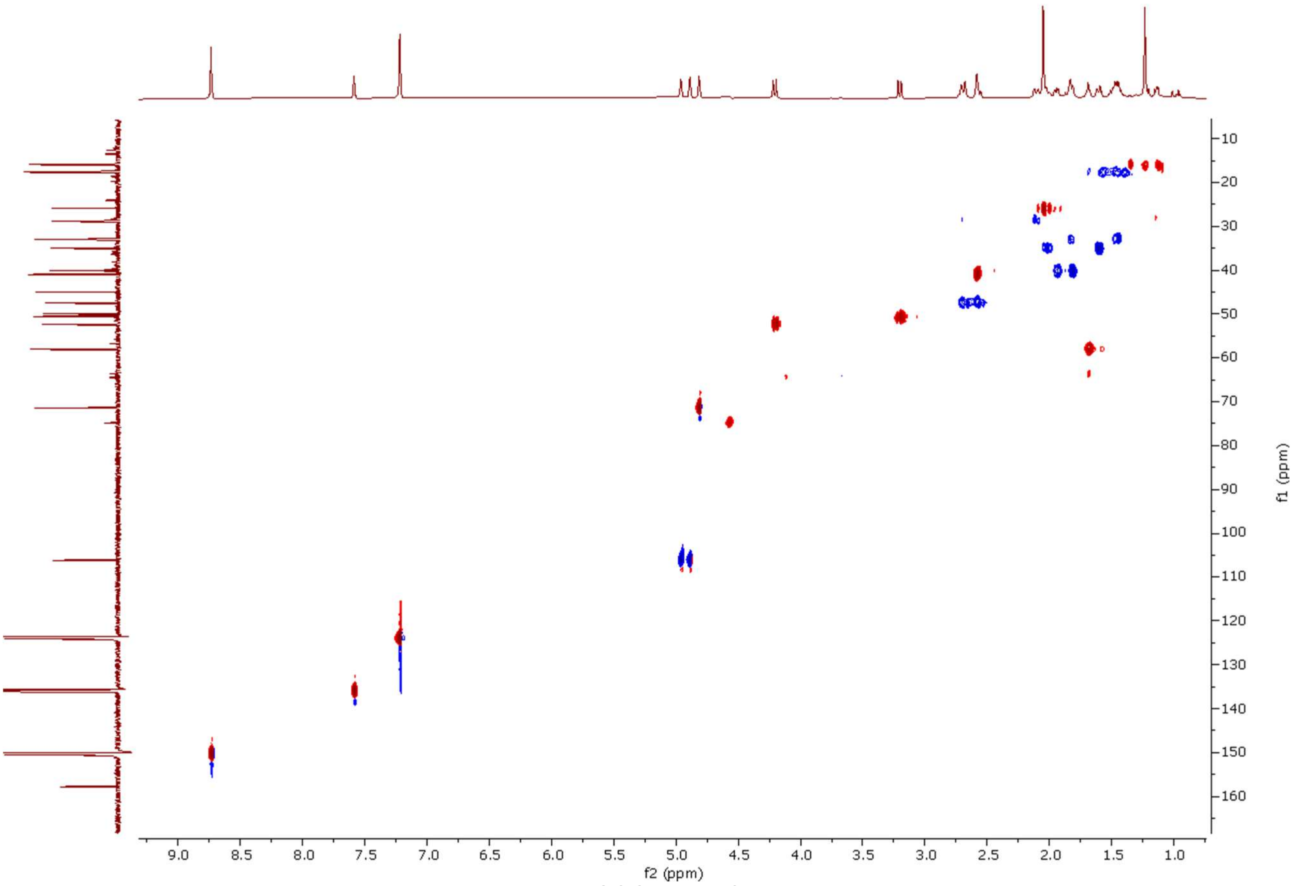

Figure N6.D HSQC NMR of **6** in pyridine-d_5.

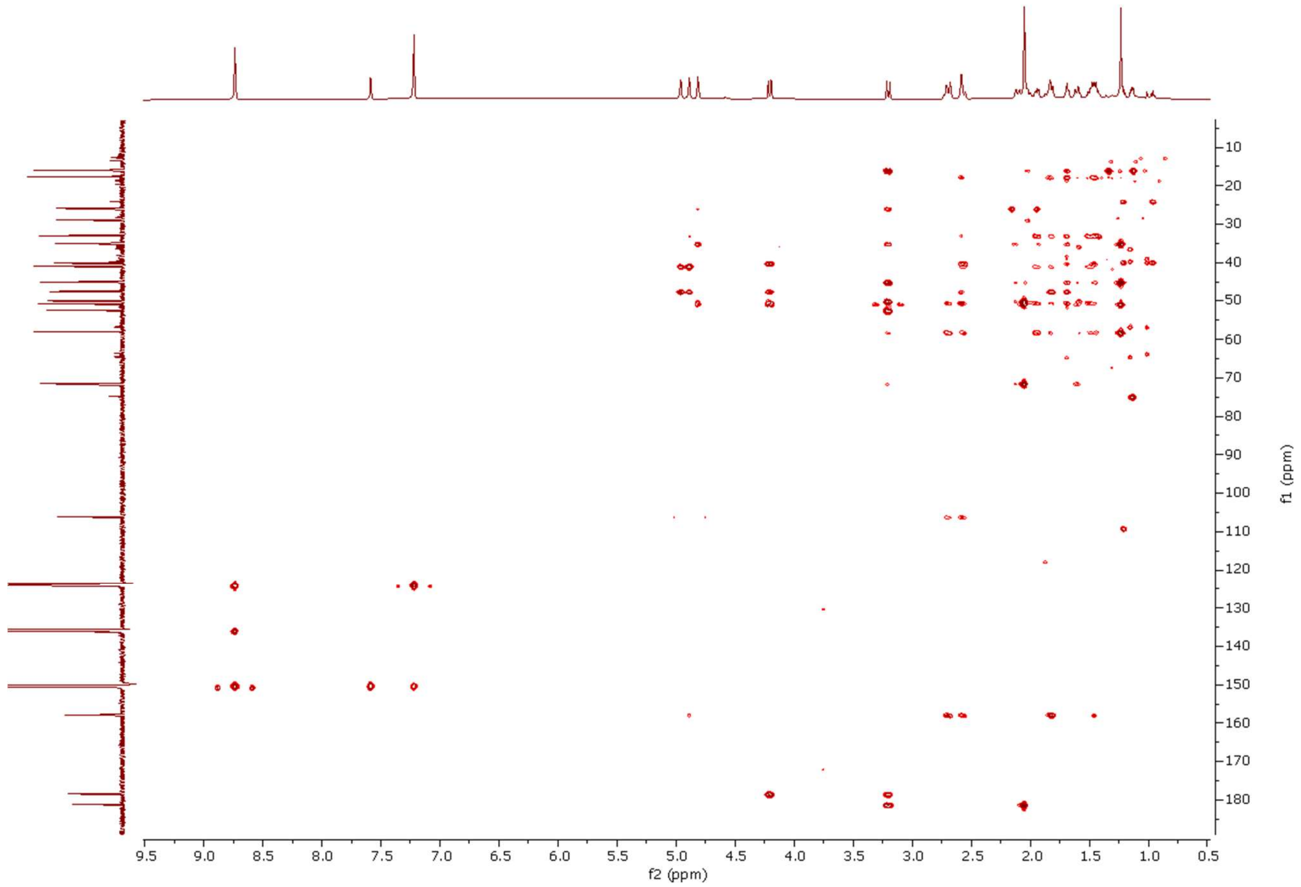

Figure N6.E HMBC NMR of **6** in pyridine-d5.

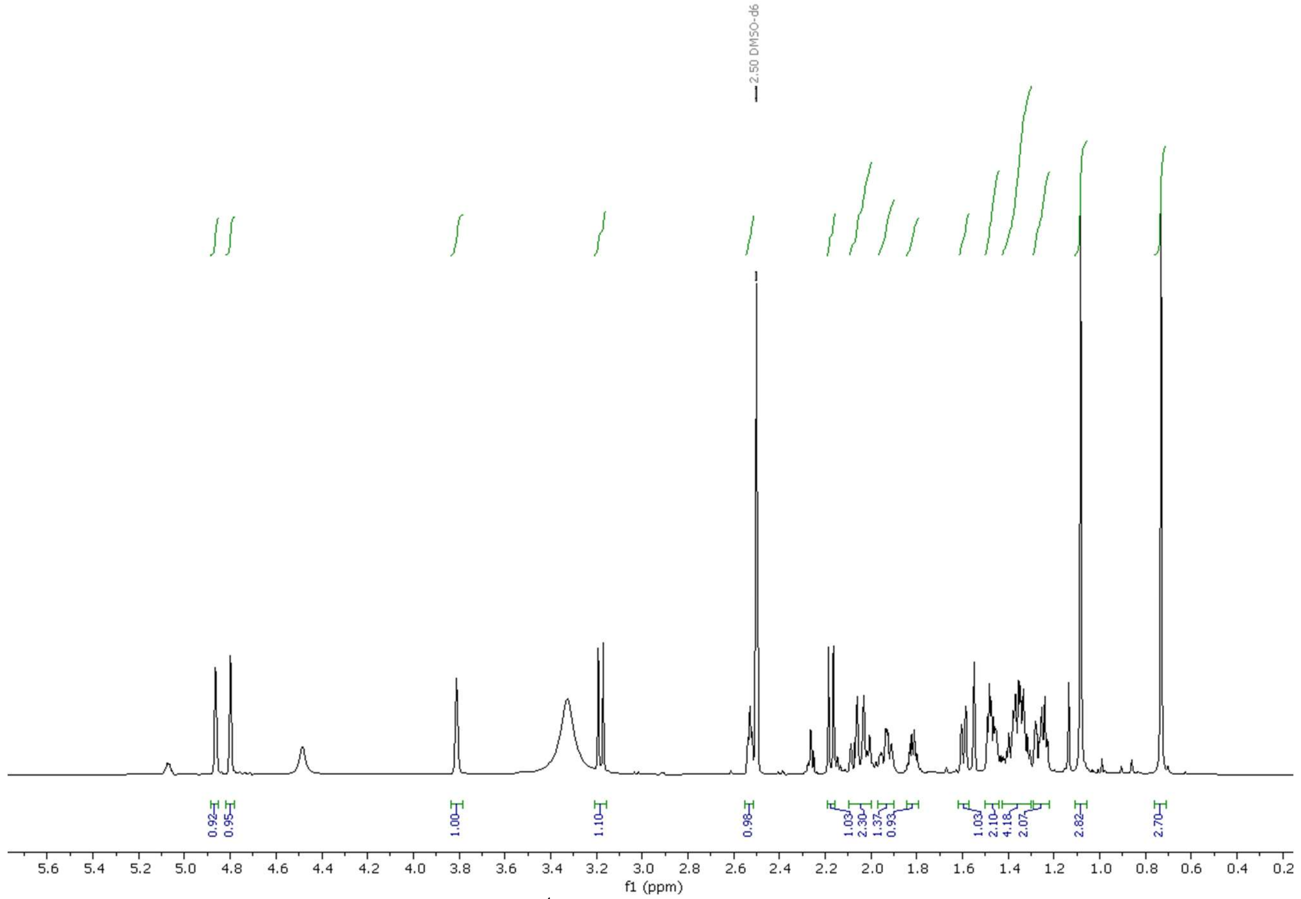

Figure N6.F ^{1}H NMR of **6** in DMSO-d6 at 600 MHz.

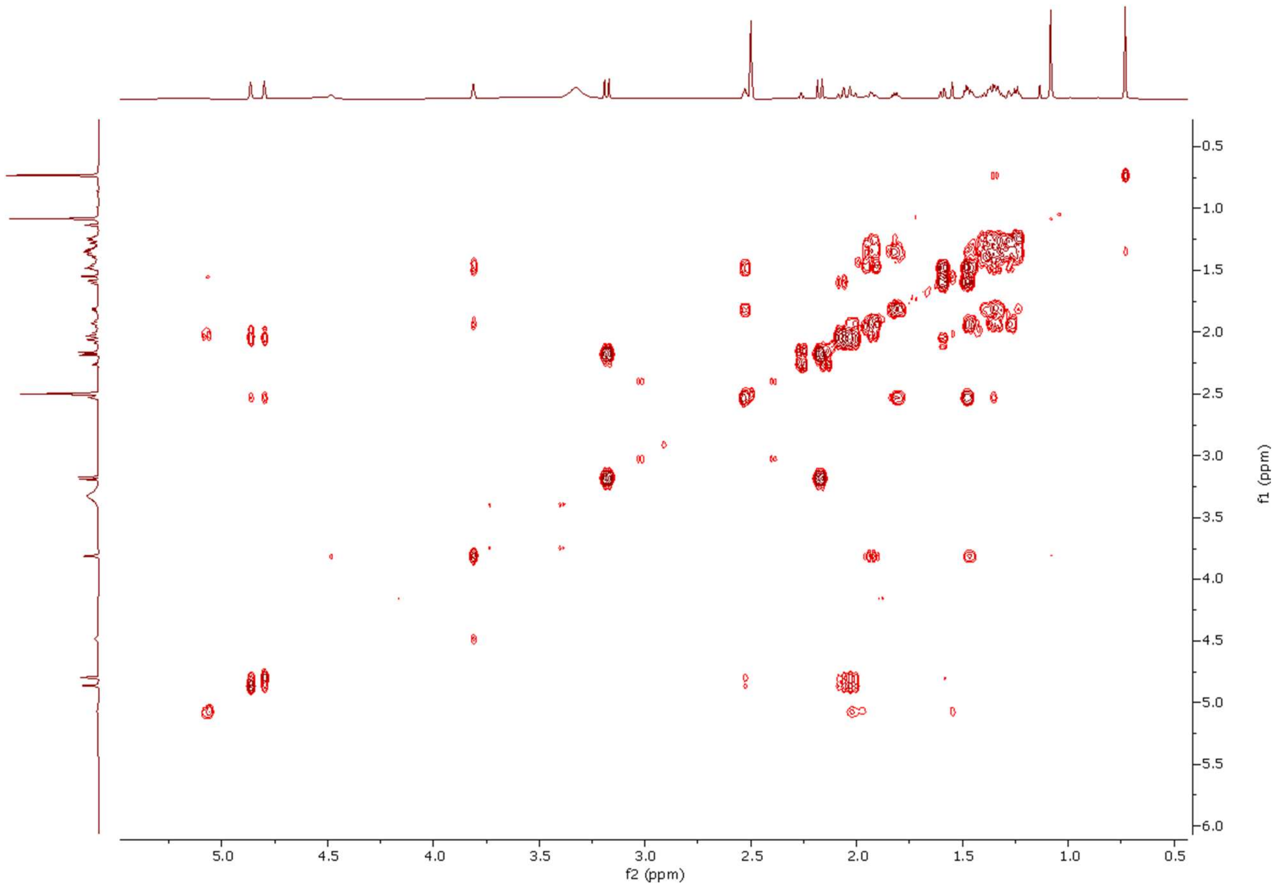

Figure N6.G COSY NMR of **6** in DMSO-d$_6$ at 600 MHz.

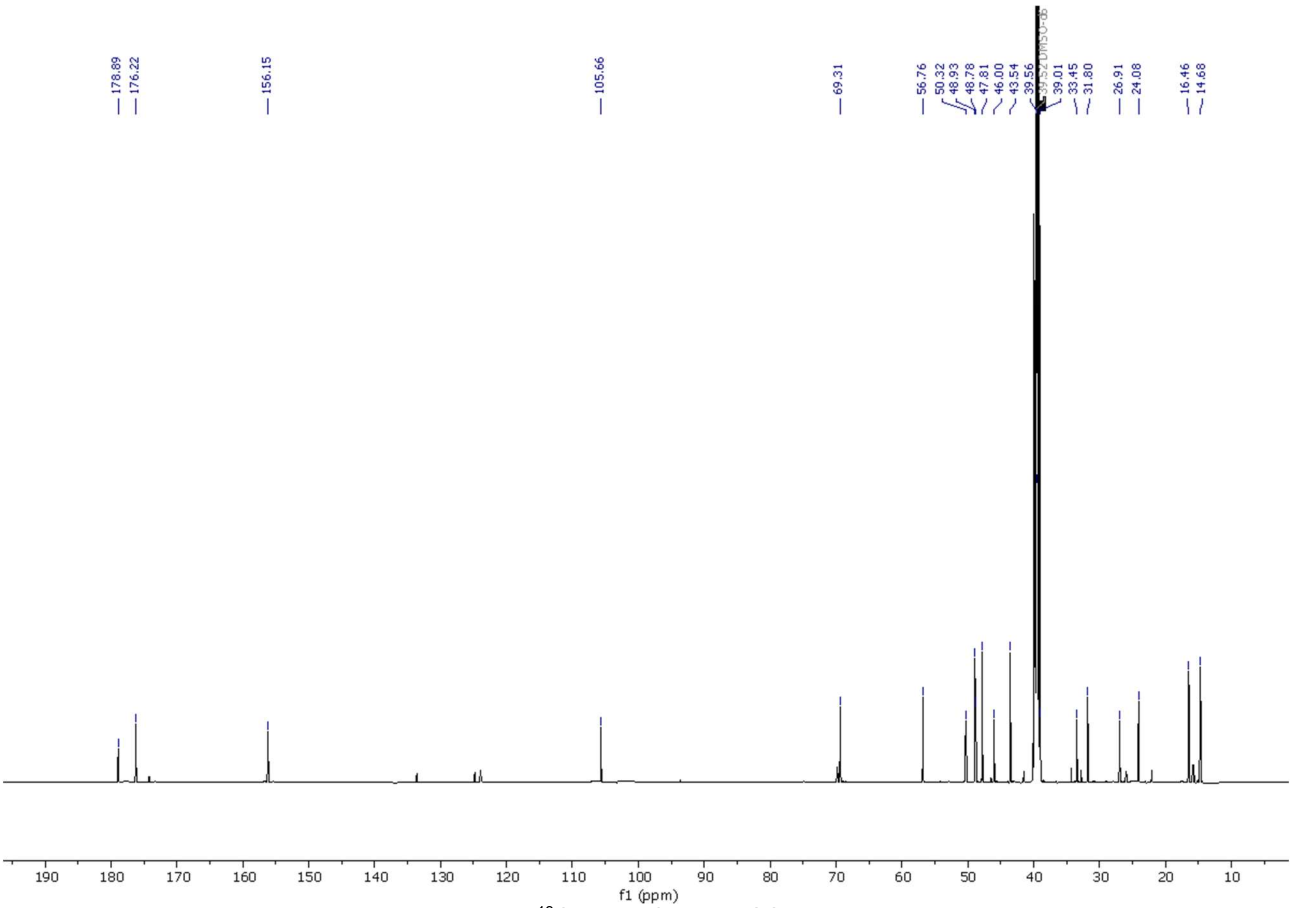

Figure N6.H ^{13}C NMR of **6** in DMSO-d$_6$ at 151 MHz.

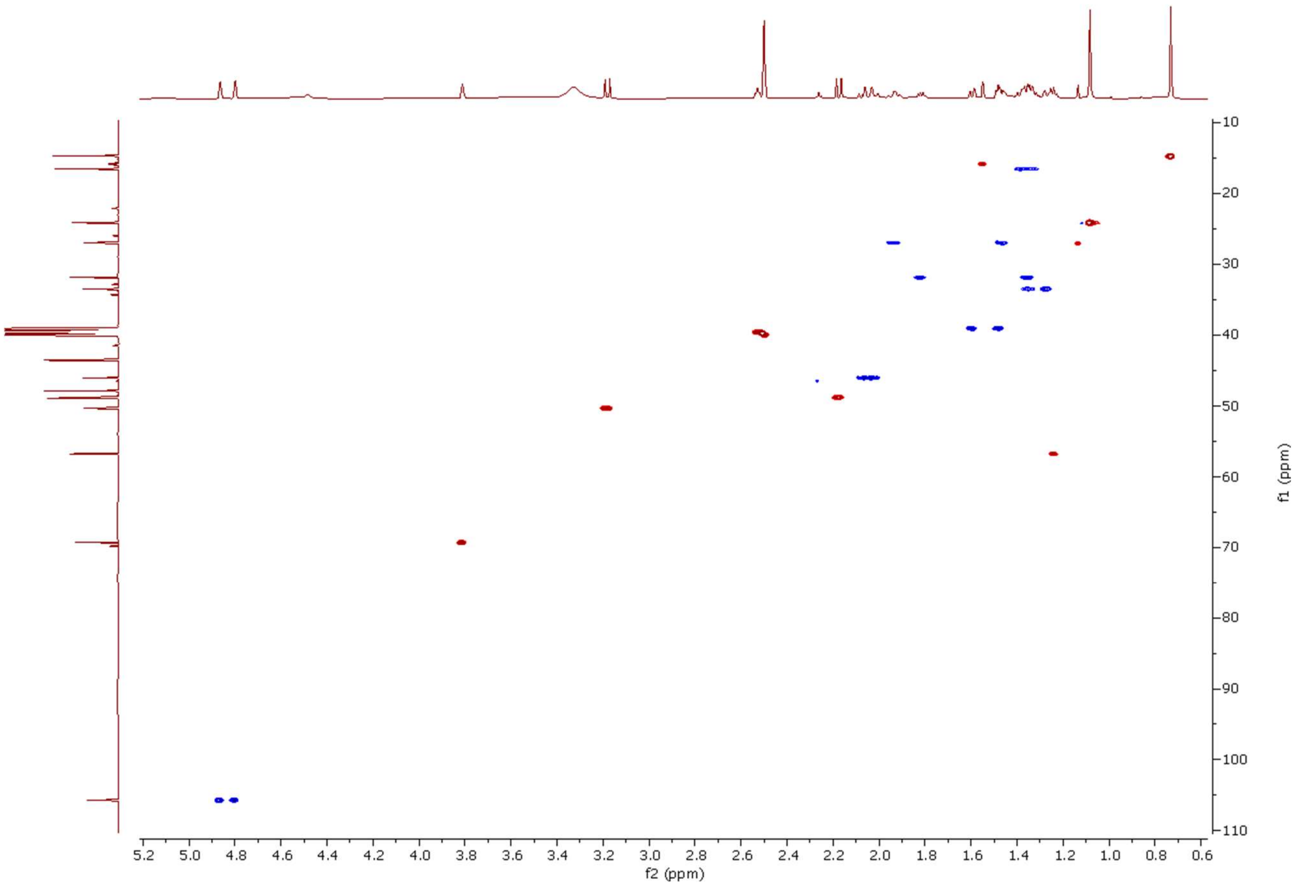

Figure N6.I HSQC NMR of **6** in DMSO-d_6.

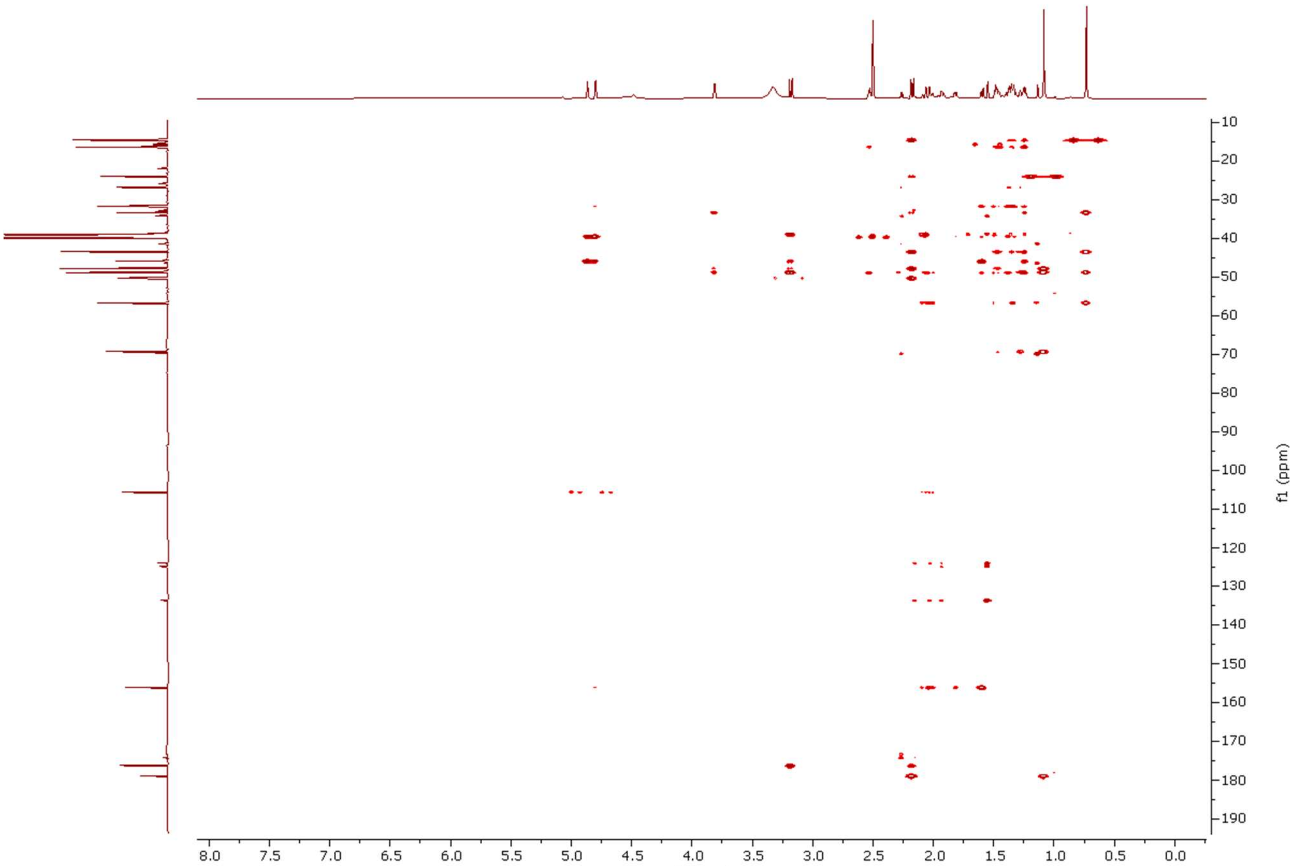

Figure N6.J HMBC NMR of **6** in DMSO-d_6.

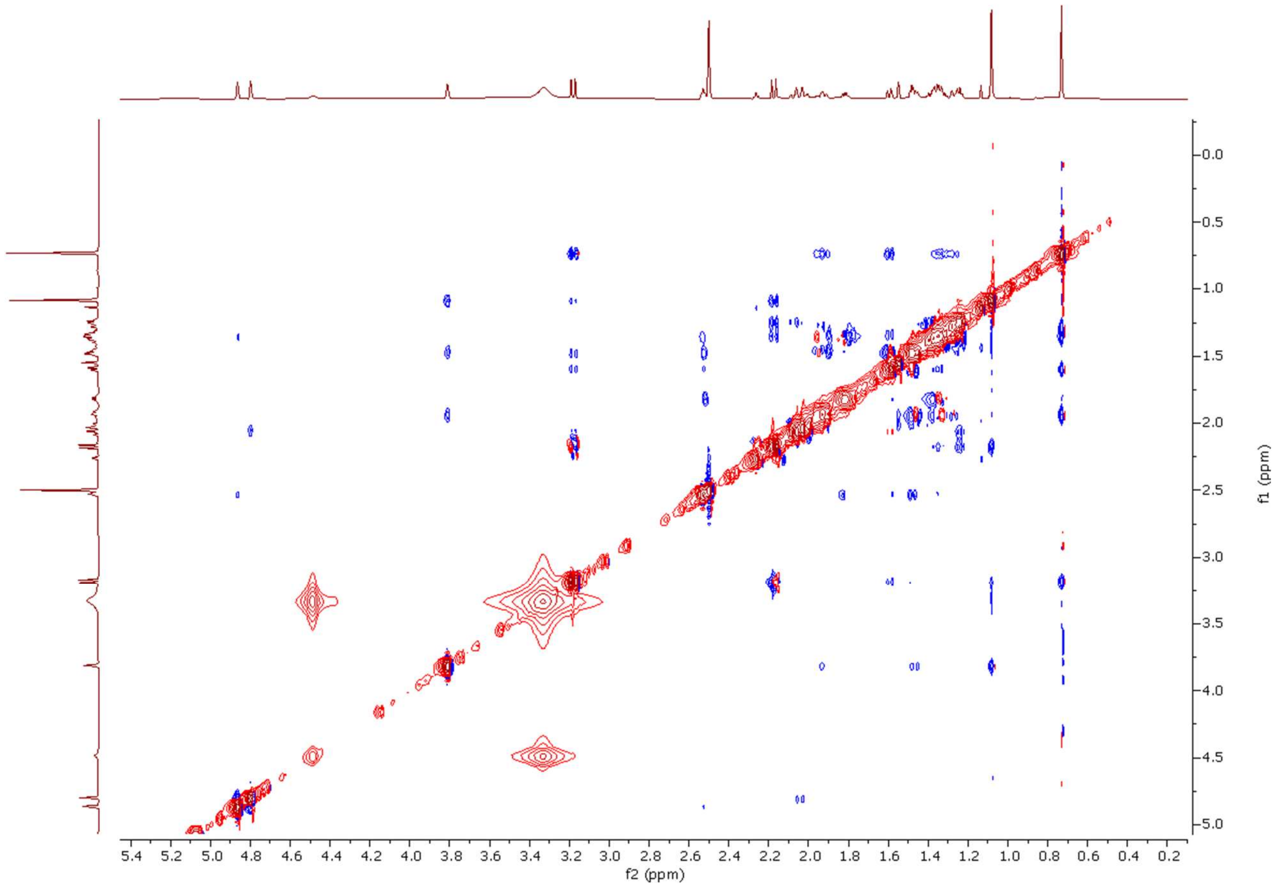

Figure N6.K NOESY NMR of **6** in DMSO-d6 at 600 MHz.

Gibberellin A_9 methyl ester (7a)

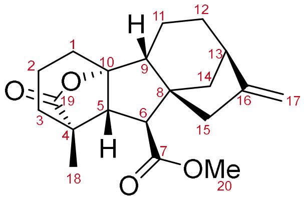

GA_9 methyl ester (**7a**)
Chemical Formula: $C_{20}H_{26}O_4$
Exact Mass: 330.1831

	Measured in $CDCl_3$, 298K		
**Pos.**	δ_C 151 MHz	δ_H (*J/Hz*) 600 MHz	**Selected NOESY correlations to H** [a]
**1**	30.9	ax 1.45, m<br>eq 2.06, overlapped	H-5
**2**	19.6	1.60, overlapped<br>1.80, overlapped	
**3**	34.5	1.56, overlapped<br>1.70, overlapped	
**4**	49.2	-	-
**5**	58.2	2.55, d (10.5)	H-9, H_3-18, H-1ax
**6**	52.0	2.71, d (10.5)	H-14a, H-14b, H_3-18
**7**	173.6 [HMBC]	-	-
**8**	51.6	-	-
**9**	54.1	1.77, overlapped	
**10**	93.6	-	-
**11**	16.3	1.51, overlapped<br>1.70, overlapped	
**12**	31.6	a 1.37, m<br>b 2.06, overlapped	
**13**	39.1	2.63, dd (7.1, 6.2)	H-12a, H-14a, H-12b, H-17a
**14**	37.0	a 1.64, overlapped<br>b 1.75, overlapped	H-6<br>H-6
**15**	44.8	a 1.89, ddd (15.2, 2.7, 2.7)<br>b 2.06, overlapped	H-15b<br>H-15a
**16**	157.1	-	-
**17**	107.5	b 4.85, br s<br>a 4.97, br s	H-17a, H-15a, H-15b<br>H-17b, H-12a, H-13
**18**	17.4	1.08, s	
**19**	179.6	-	-
**20**	52.1	3.71, s	H-15a

[a] Key NOESY correlations are shown in blue text.

[HMBC] = detected based on HMBC.

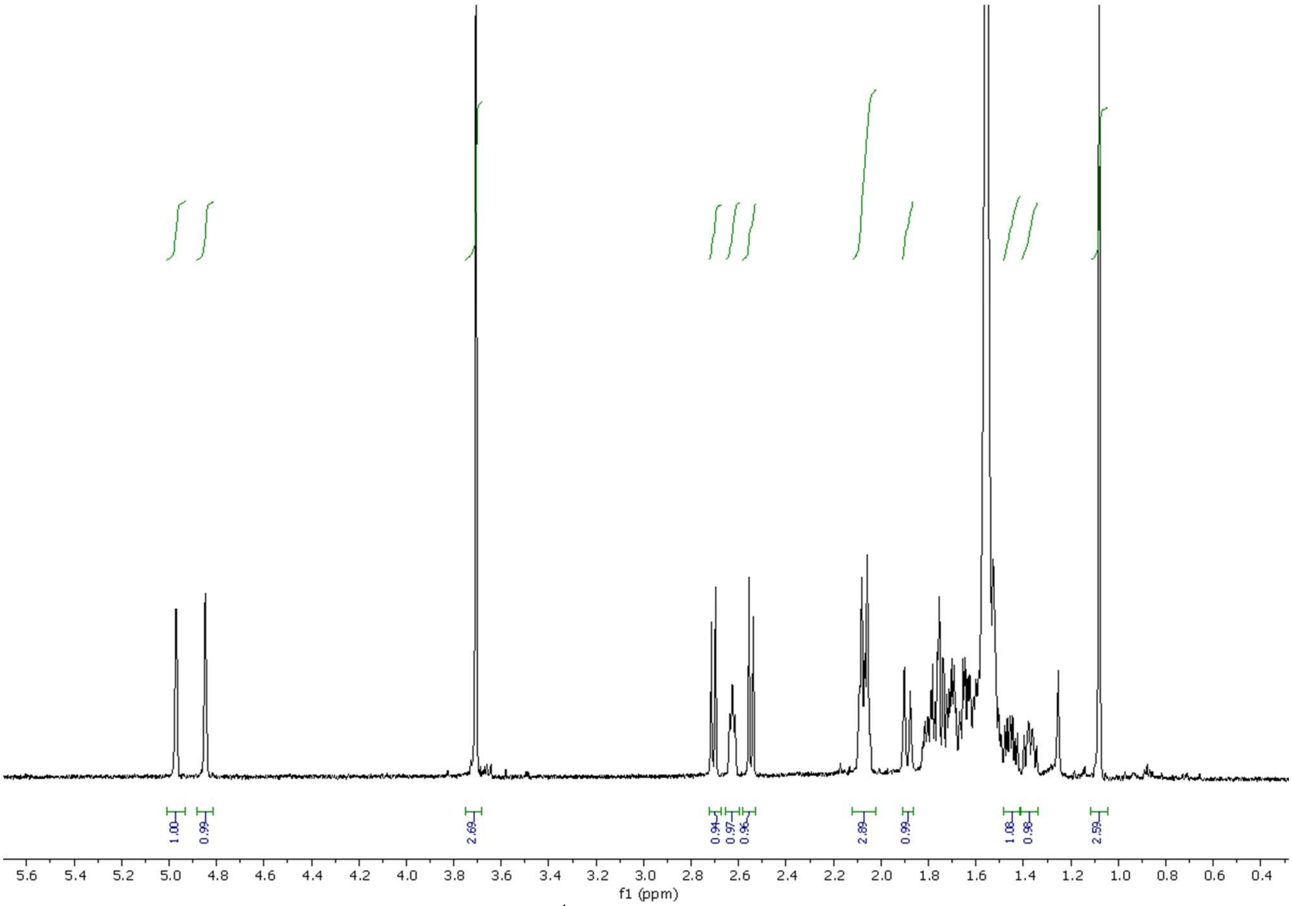

Figure N7a.A ^{1}H NMR of **7a** in $CDCl_3$ at 600 MHz.

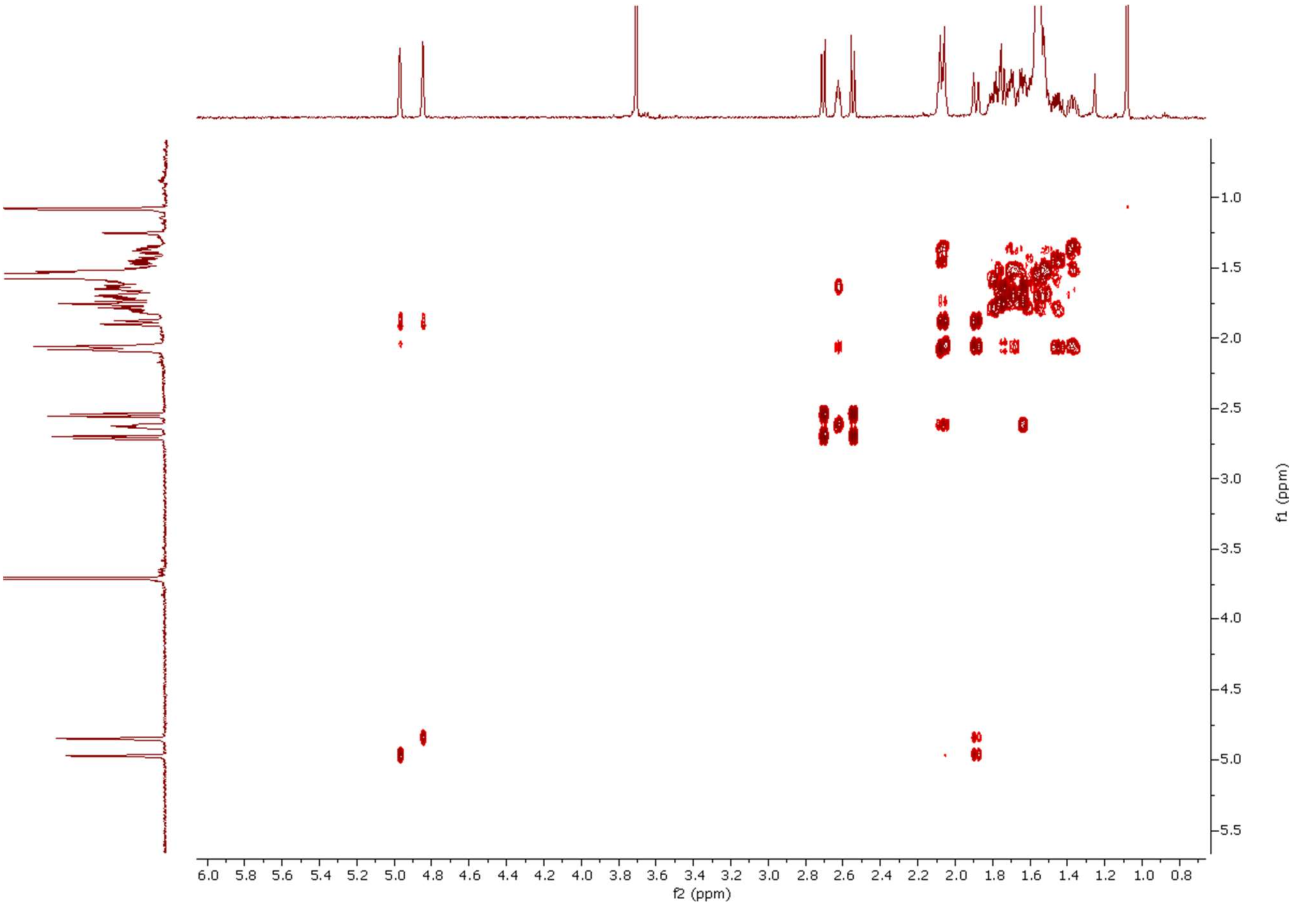

Figure N7a.B COSY NMR of **7a** in $CDCl_3$ at 600 MHz.

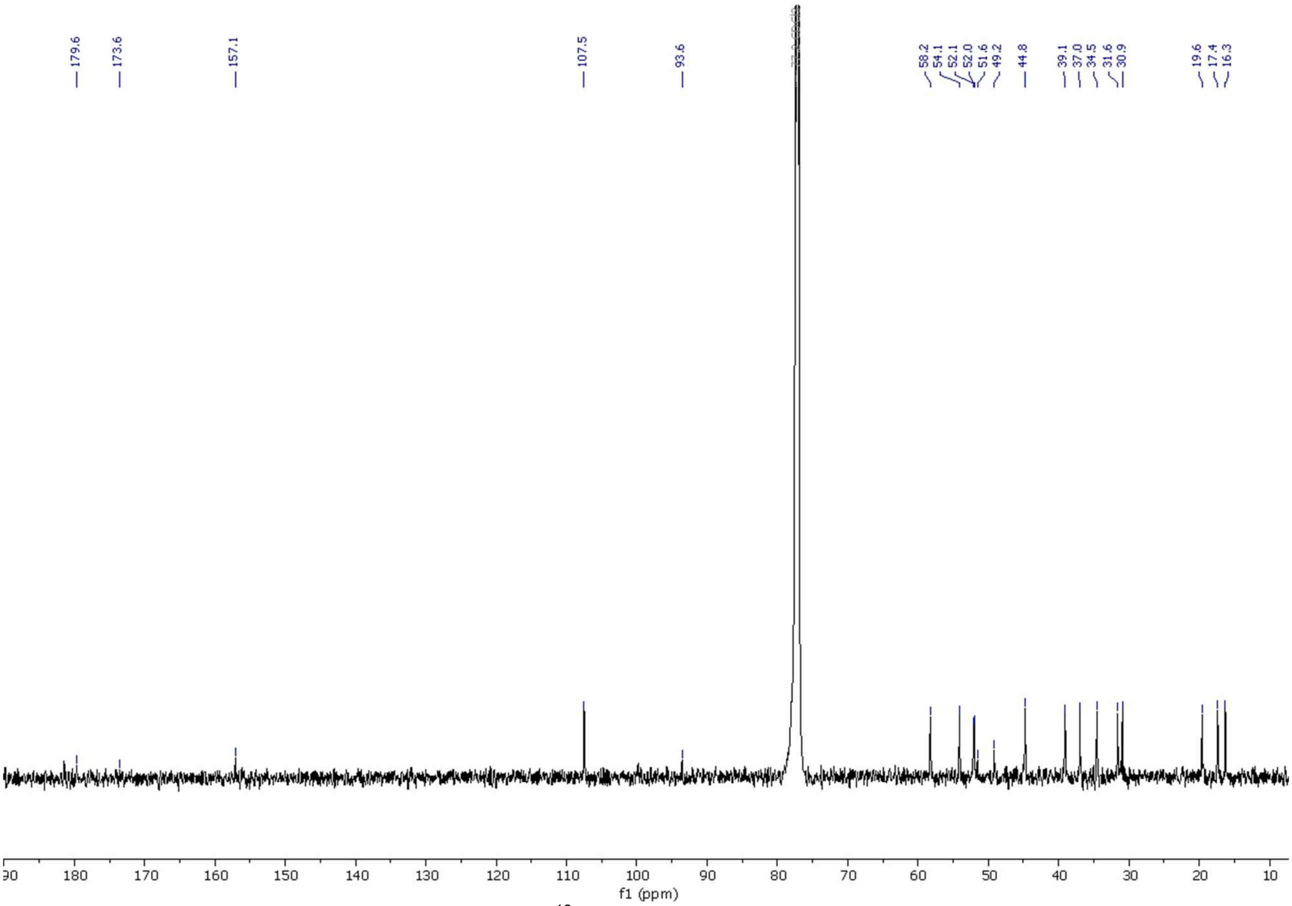

Figure N7a.C ^{13}C NMR of **7a** in $CDCl_3$ at 151 MHz.

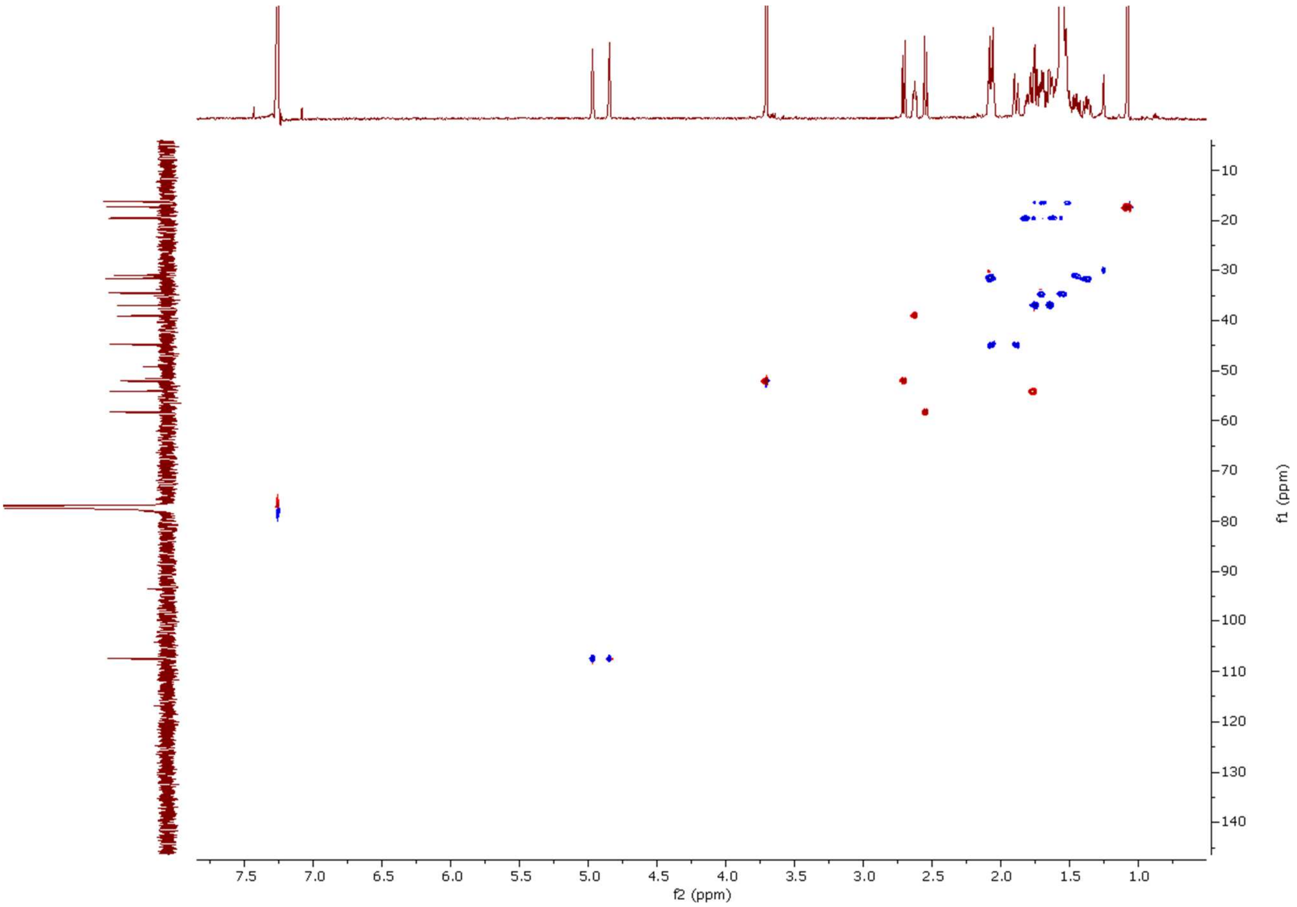

Figure N7a.D HSQC NMR of **7a** in $CDCl_3$.

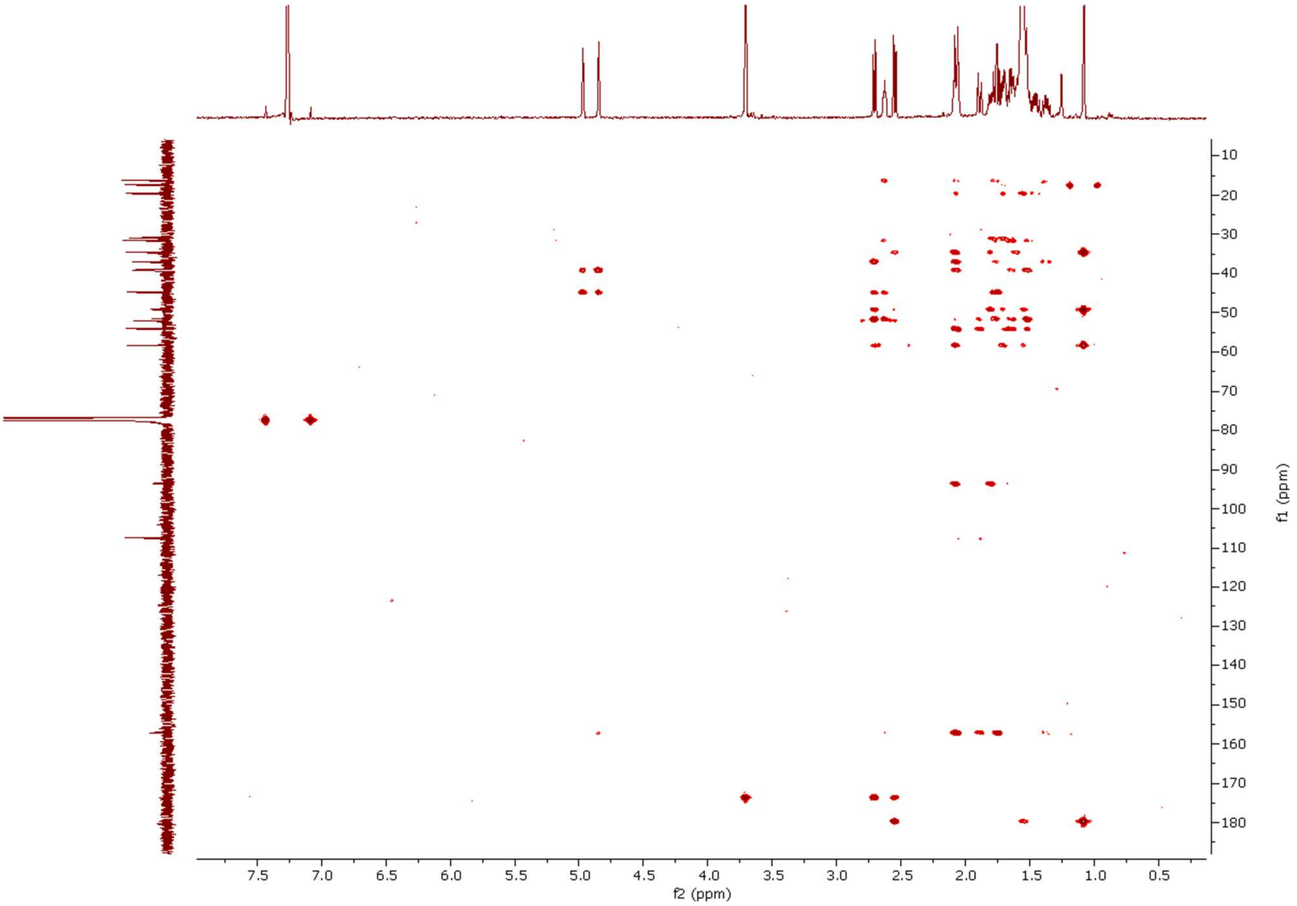

Figure N7a.E HMBC NMR of **7a** in $CDCl_3$.

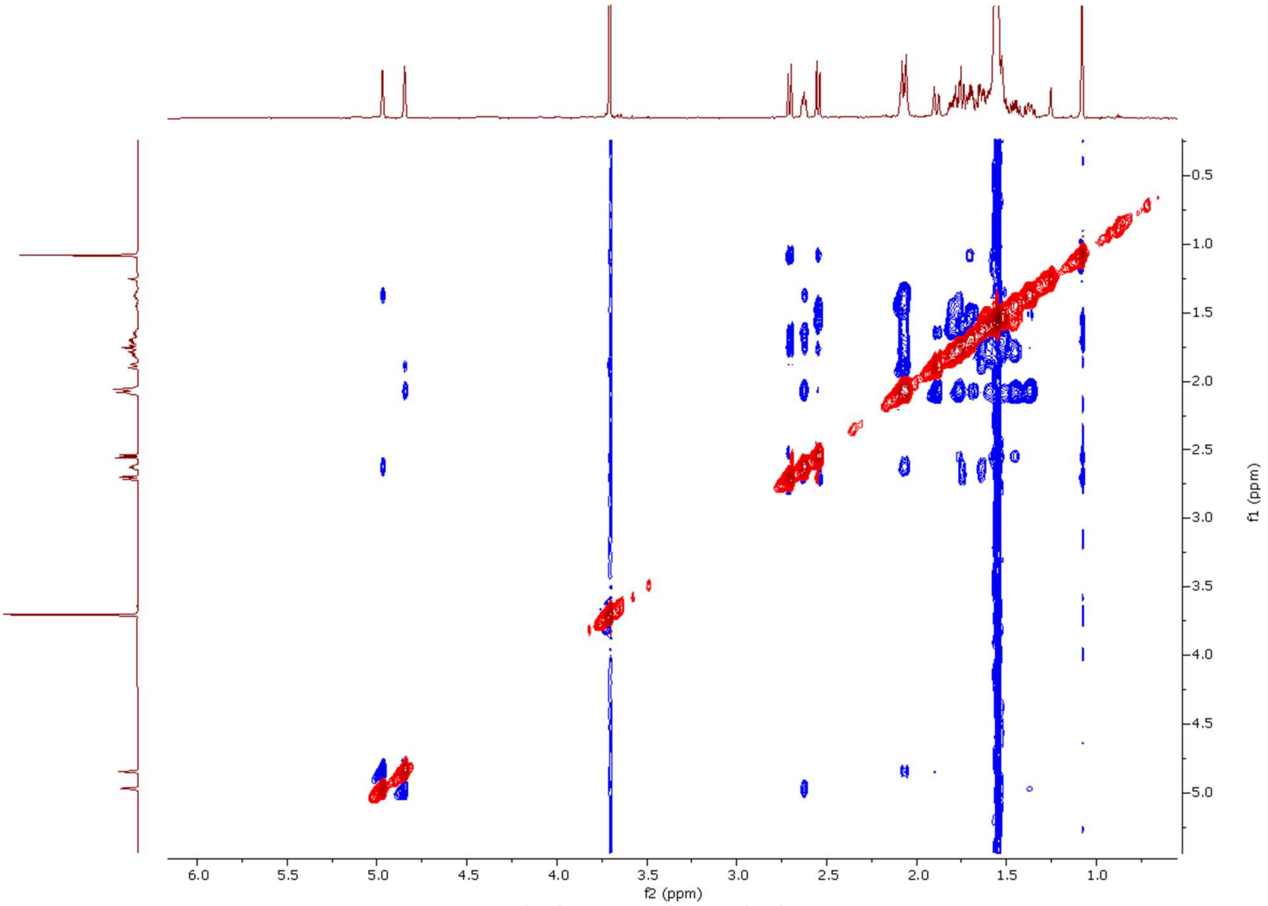

Figure N7a.F NOESY NMR of **7a** in $CDCl_3$ at 600 MHz.

Gibberellin A_{13} (8)

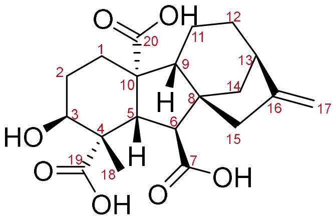

GA$_{13}$ (**8**)
Chemical Formula: $C_{20}H_{26}O_7$
Exact Mass: 378.1679

Pos.	Reference in C_5D_5N [41] (only δ_C reported) δ_C 25.15 MHz	Measured in C_5D_5N, 298K δ_C 126 MHz	δ_H (J/Hz) 500 MHz	Select. NOESY corr. to H	Measured in DMSO-D$_6$, 298K δ_C 151 MHz	δ_H (J/Hz) 600 MHz	Select. NOESY corr. to H [a]
**1**	30.8	32.3	ax 2.15, overlapped<br>eq 2.83, ddd (12.2, 3.6, 3.6)	H-1eq<br>H-1ax	30.4	ax 1.31, overlapped<br>eq 2.04, overlapped	H-5
**2**	32.2	31.4	a 2.34, m<br>b 3.35, m	H-2b<br>H-2a	29.1	a 1.53, m<br>b 2.06, overlapped	
**3**	71.2	71.7	eq 4.71, dd (2.9, 2.9)	H_3-18, H-2a, H-2b	69.3	3.69, br s	H-2b
**4**	50.6	51.2	-	-	48.8	-	-
**5**	50.4	50.8	3.52, d (12.7)	H-9, H_3-18, H-6	49.3	2.33, d (12.7)	H-1ax, H-9, H-6
**6**	52.1	52.5	5.03, d (12.7)	H-14a, H-14b, H-5	50.3	3.68, d (12.7)	H-14b, H-5
**7**	177.1	178.5	-	-	176.3	-	-
**8**	50.6	51.1	-	-	49.6	-	-
**9**	57.0	57.4	1.96, overlapped	H-5	55.8	1.42, overlapped	H-5
**10**	57.6	58.2	-	-	55.9	-	-
**11**	19.4	19.8	1.76, m<br>1.78, m		18.2	1.27, overlapped<br>1.44, overlapped	
**12**	32.2	32.5	a 1.39, m<br>b 1.94, overlapped	H-13<br>H-13	31.3	1.25, overlapped<br>1.82, m	
**13**	40.2	40.7	2.54, dd (6.3, 6.3)	H-12a, H-12b, H-17a	39.4	2.49, overlapped	
**14**	36.9	37.3	a 1.90, dd (11.4, 5.2)<br>b 2.26, dd (11.4, 1.7)	H-6<br>H-6	36.8	a 1.34, overlapped<br>b 1.66 br d (11.5)	
**15**	47.3	47.8	a 2.61, br d (16.0)<br>b 2.74, ddd (16.0, 2.9, 2.9)	H-17b<br>H-17b	46.0	2.00, overlapped<br>2.05, overlapped	
**16**	157.9	158.6	-	-	156.5	-	-
**17**	105.9	106.1	b 4.87, br s<br>a 4.95, dd (2.9, 1.7)	H-15a, H-15b<br>H-13	105.7	b 4.78, br s<br>a 4.85, br s	H-13
**18**	24.8	25.4	2.18, s	H-3, H-5	23.6	1.11, s	
**19**	178.0	179.0	-	-	176.5	-	-
**20**	178.5	177.7	-	-	175.5	-	-

[a] Key NOESY correlations are shown in blue text.

Yellow highlighted data are possibly swapped assignments in the referenced data. Our assignments are supported by HMBC correlations.

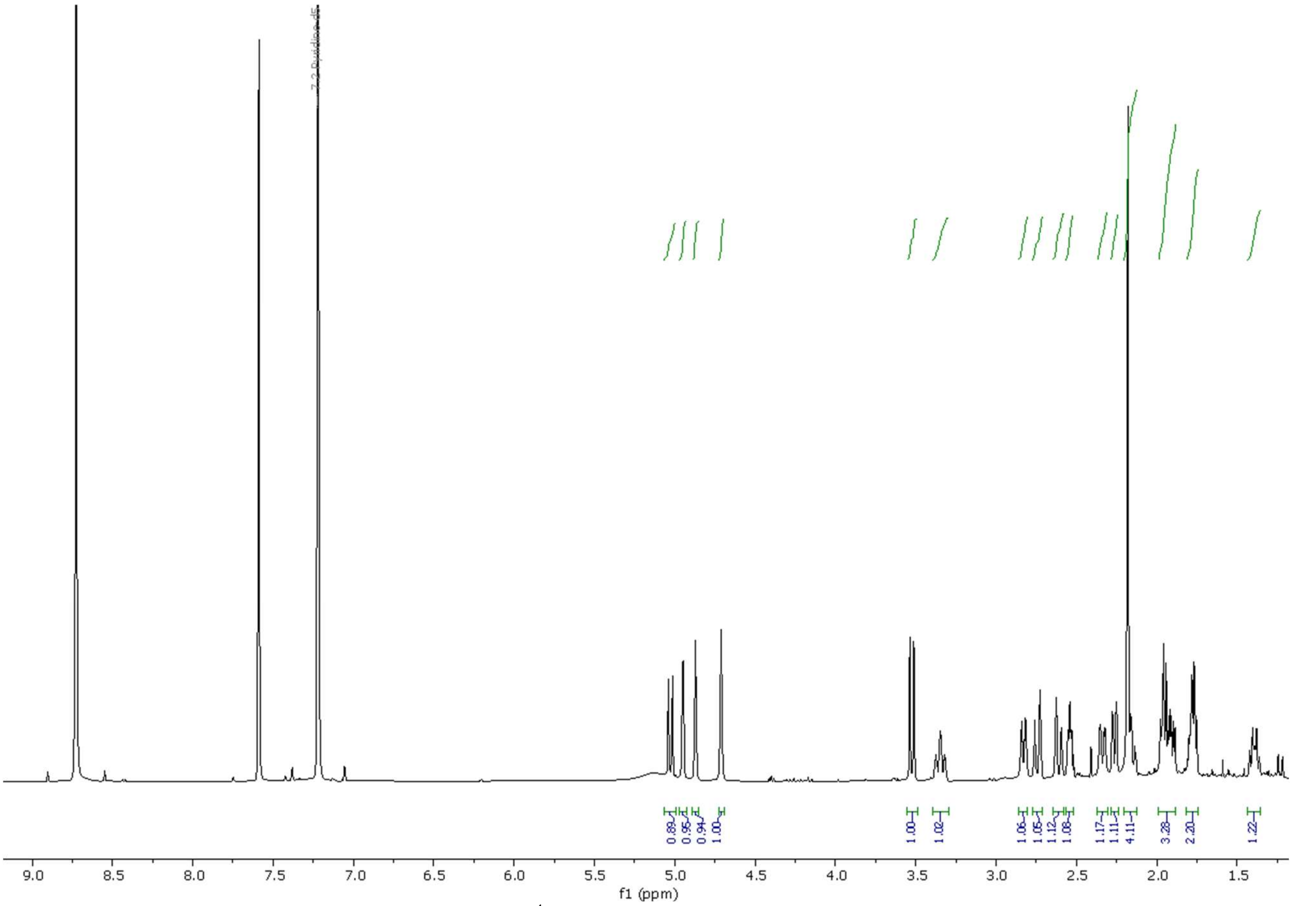

Figure N8.A ^{1}H NMR of **8** in d_5-pyridine at 500 MHz.

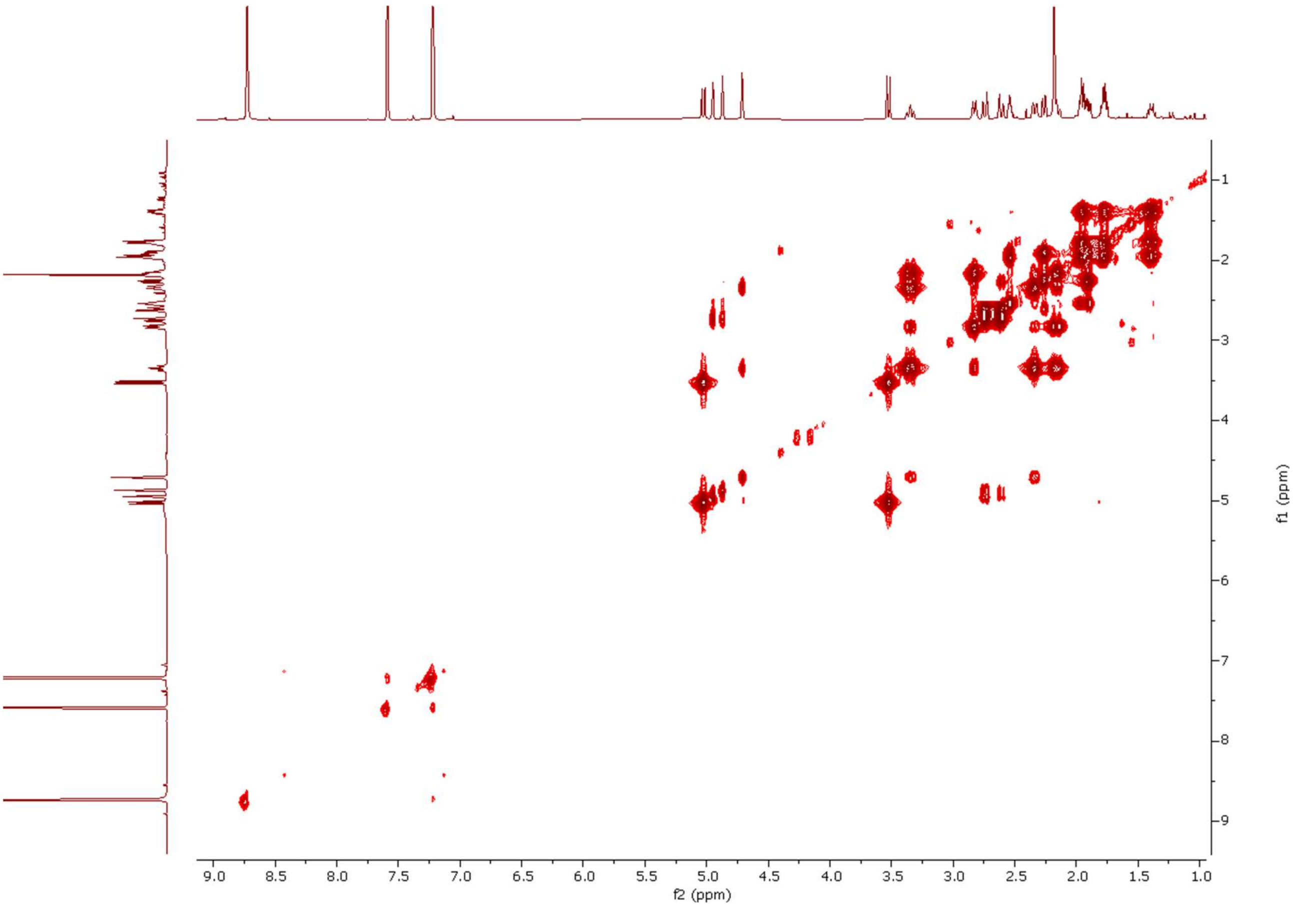

Figure N8.B COSY NMR of **8** in d_5-pyridine at 500 MHz.

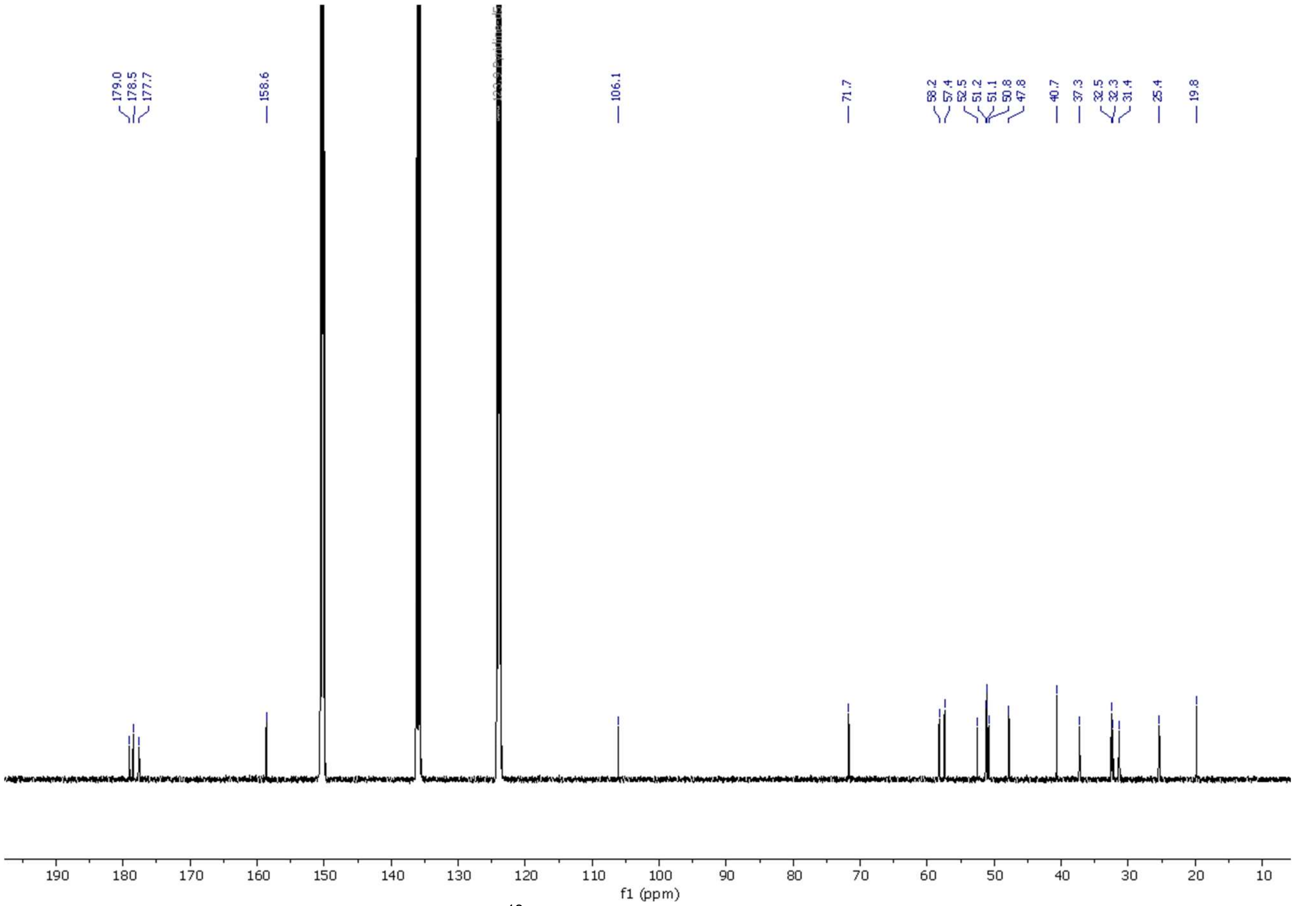

Figure N8.C ^{13}C NMR of **8** in d5-pyridine at 126 MHz.

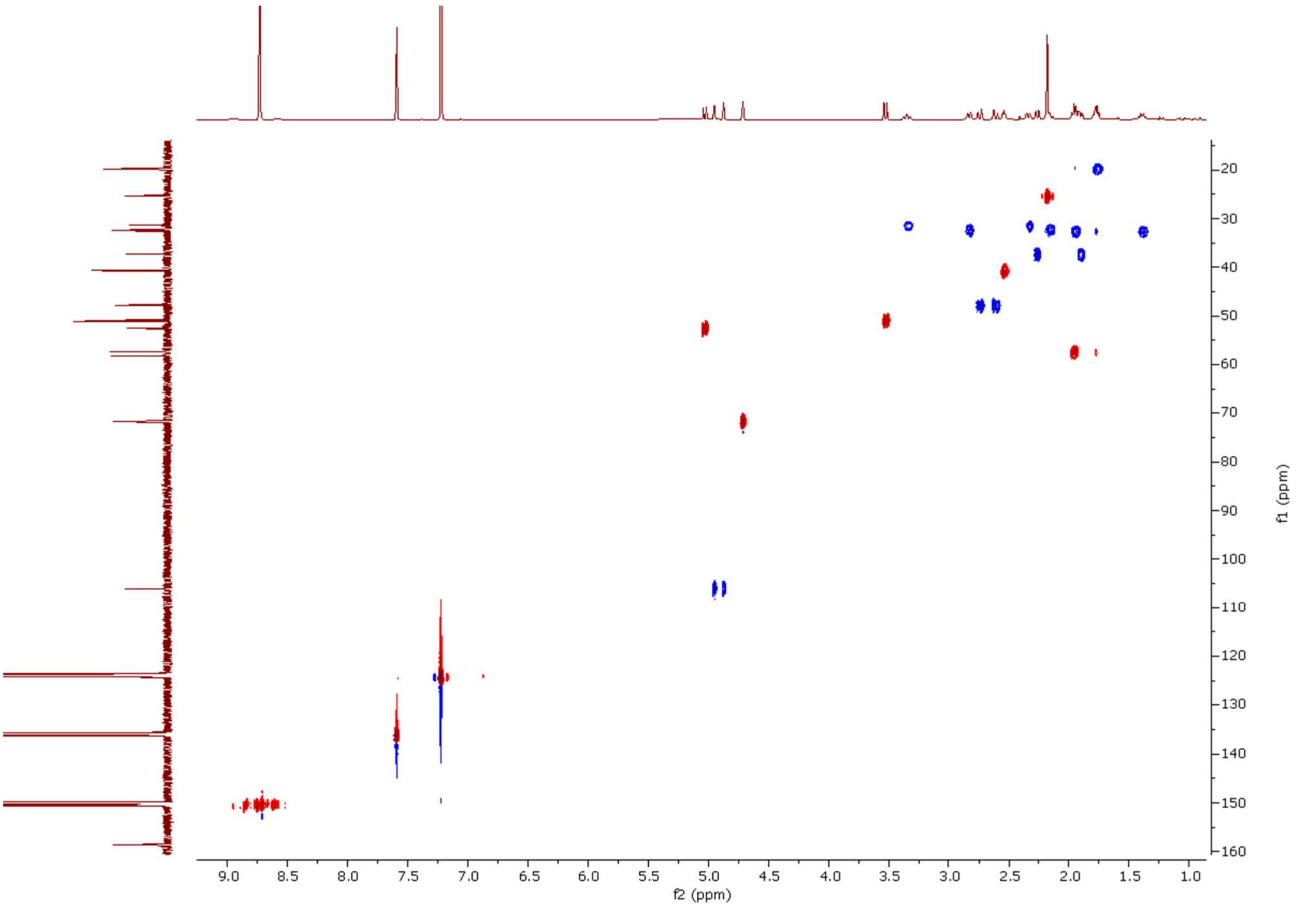

Figure N8.D HSQC NMR of **8** in d5-pyridine.

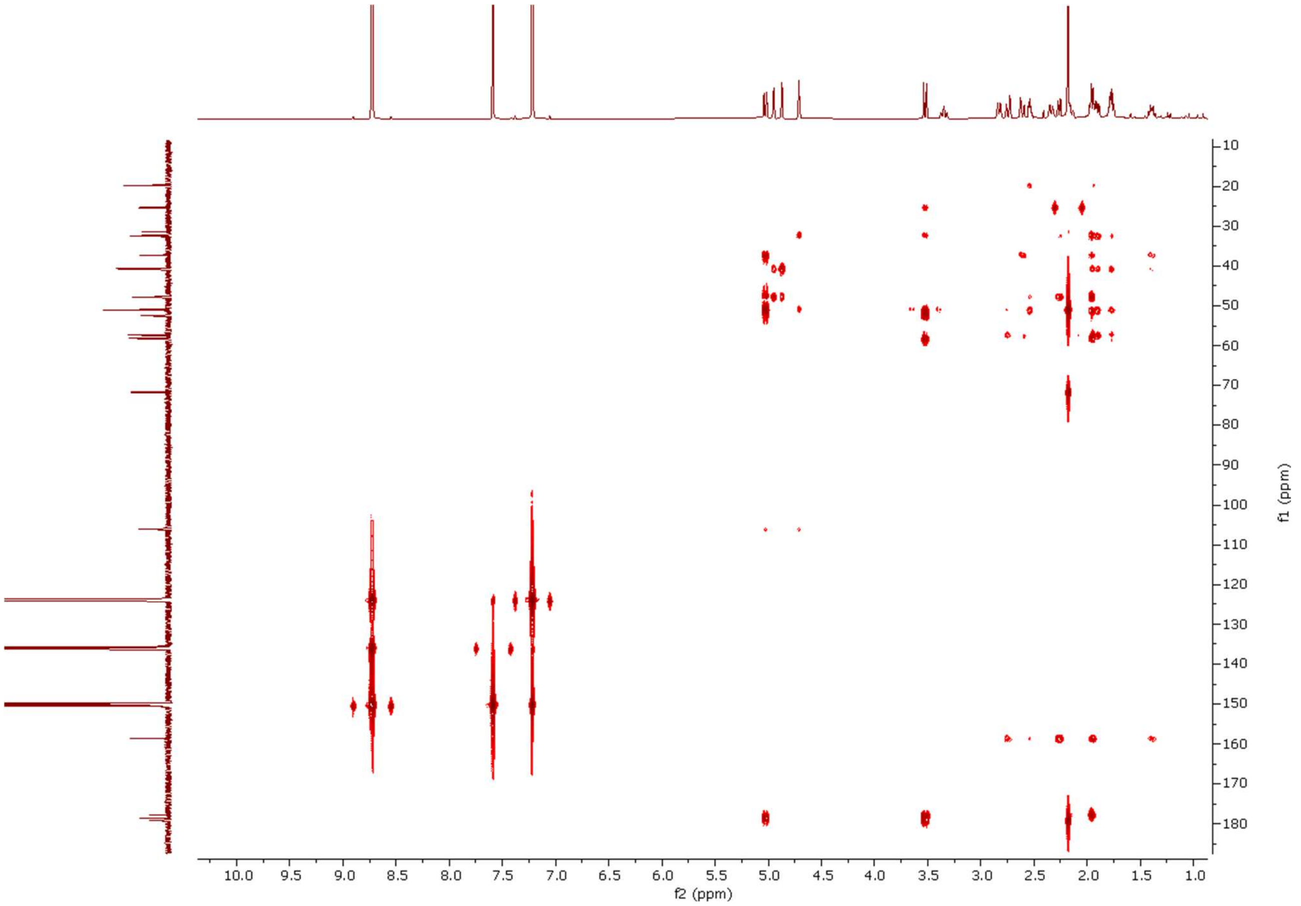

Figure N8.E HMBC NMR of **8** in d_5-pyridine.

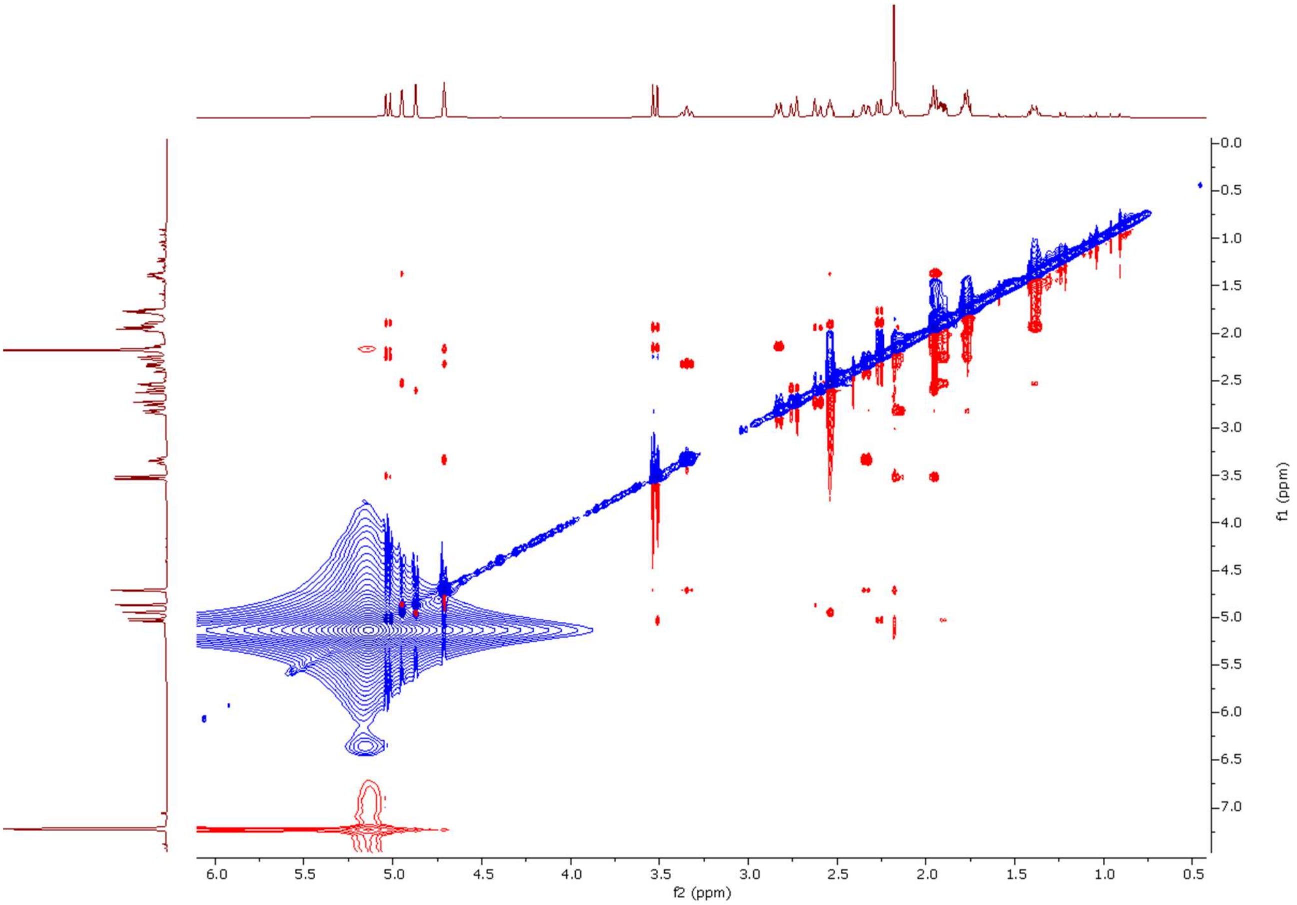

Figure N8.F NOESY NMR of **8** in d_5-pyridine at 500 MHz.

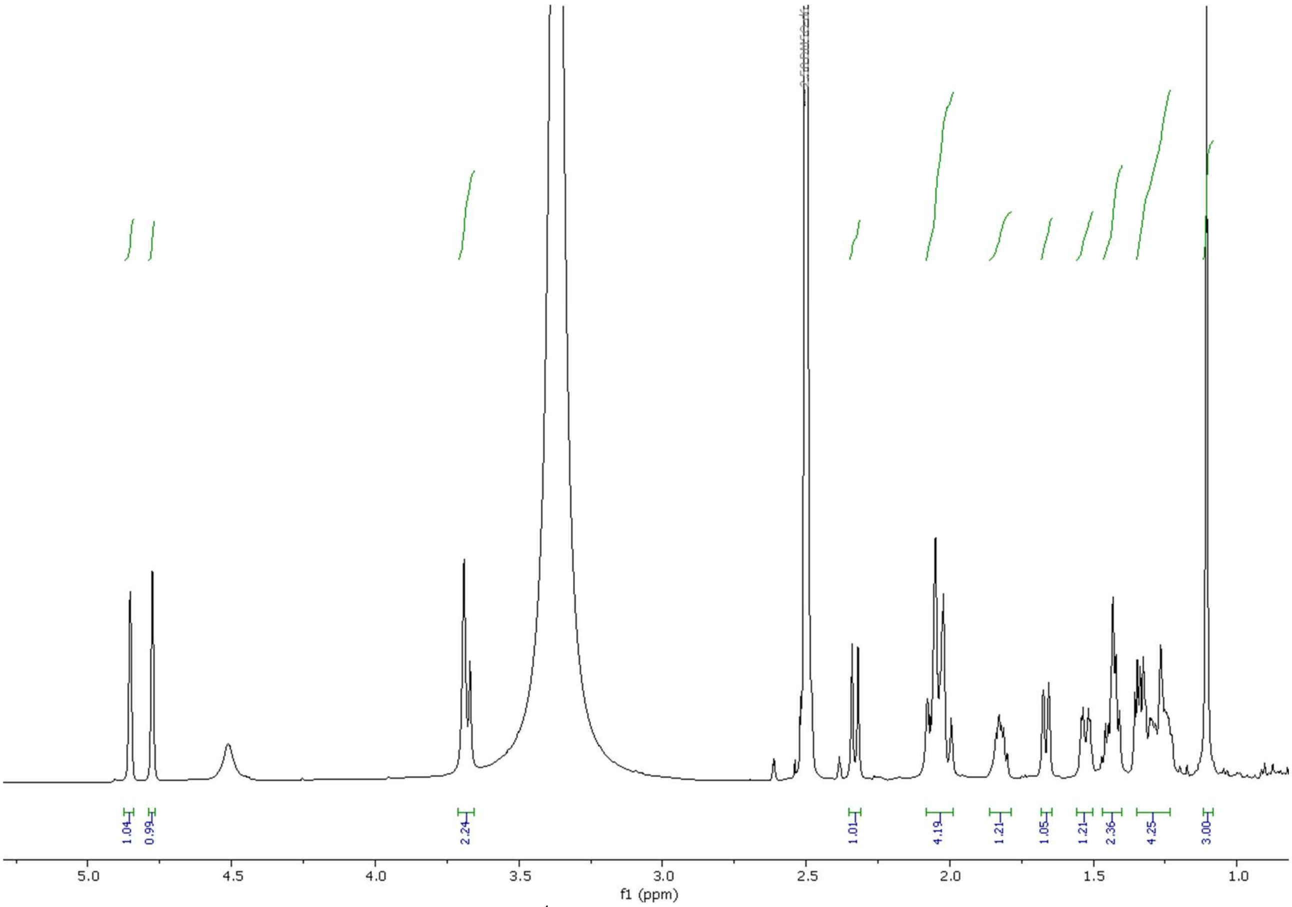

Figure N8.G ^{1}H NMR of **8** in DMSO-d_6 at 600 MHz.

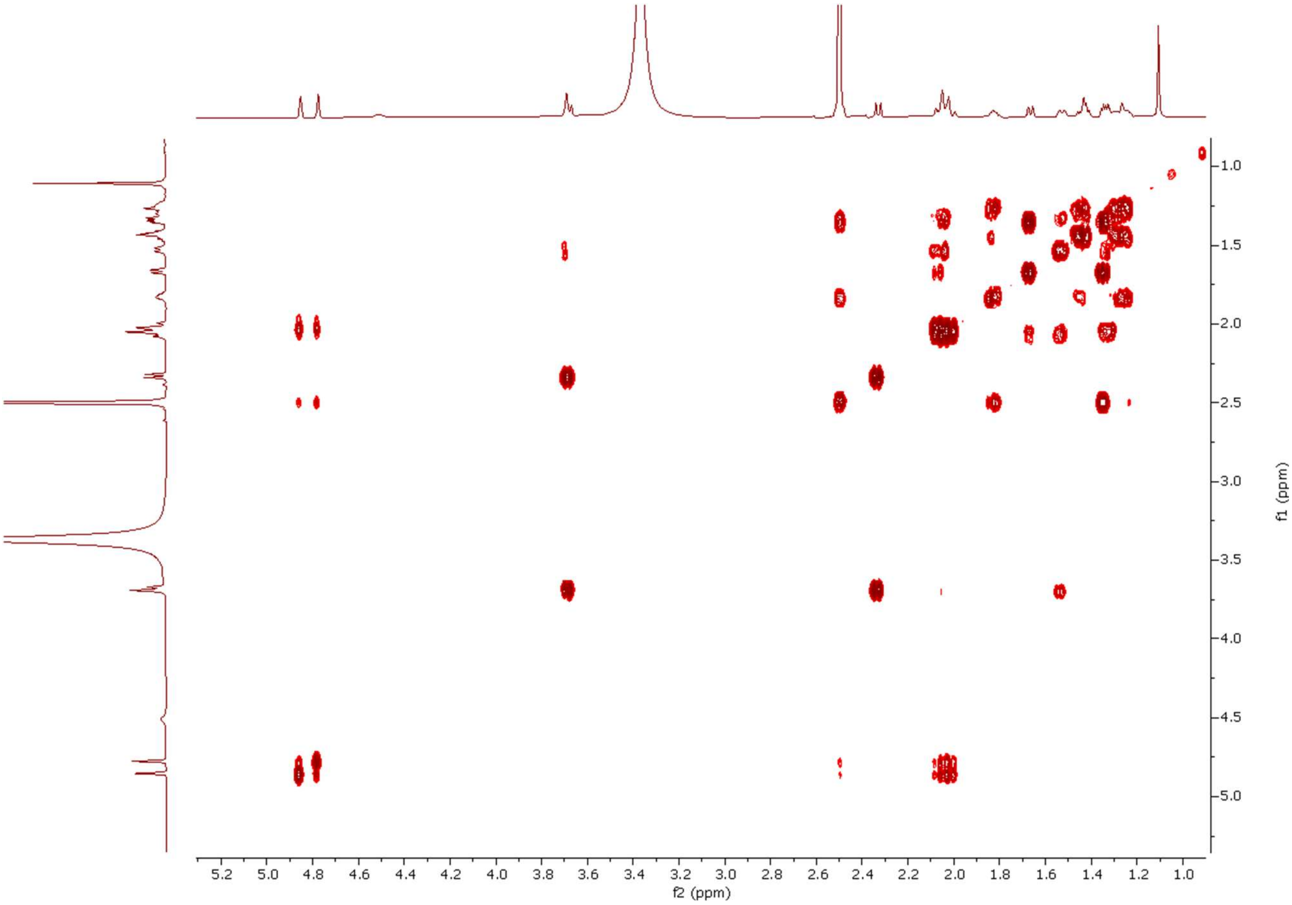

Figure N8.H COSY NMR of **8** in DMSO-d_6 at 600 MHz.

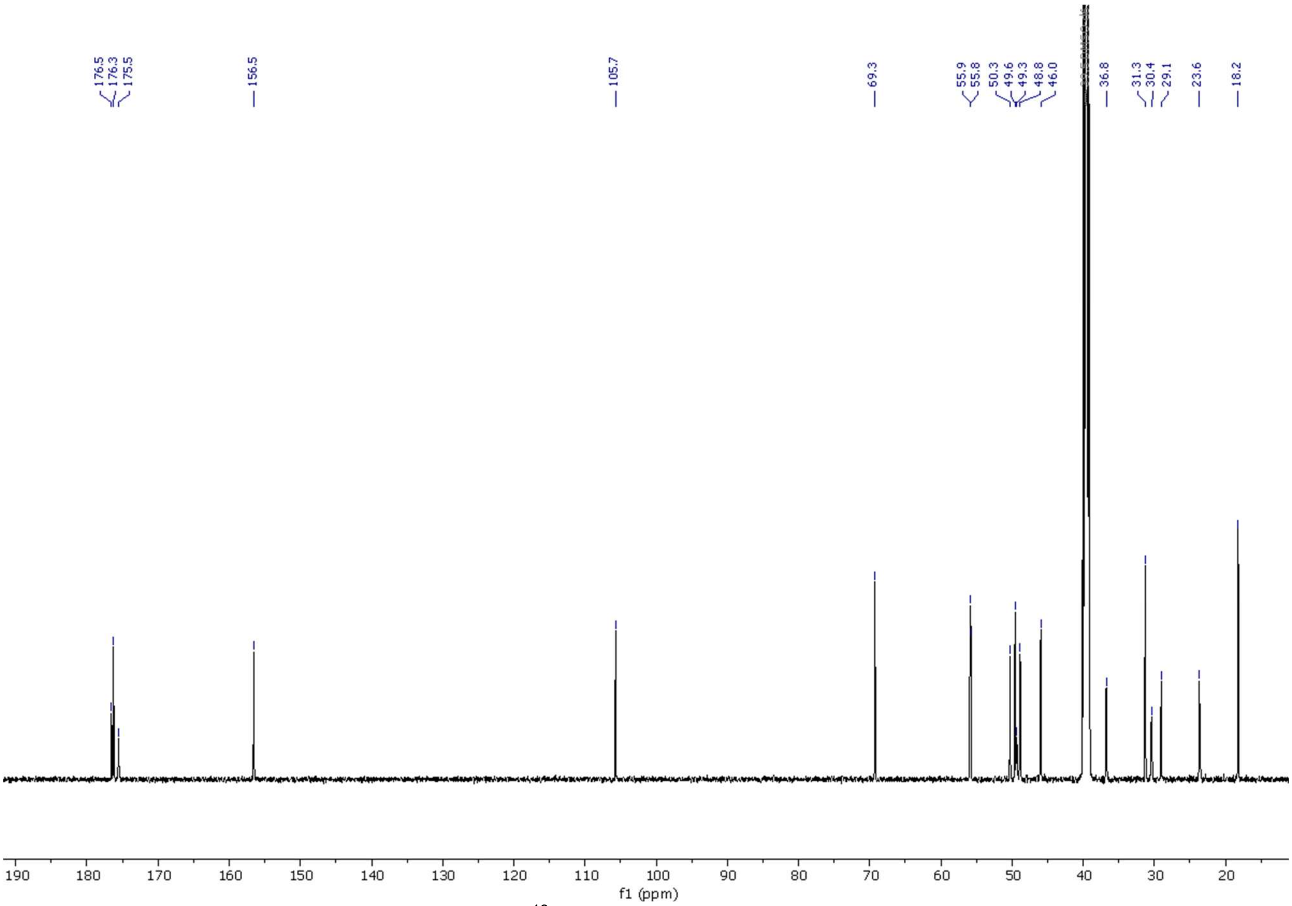

Figure N8.I ^{13}C NMR of **8** in DMSO-d$_6$ at 151 MHz.

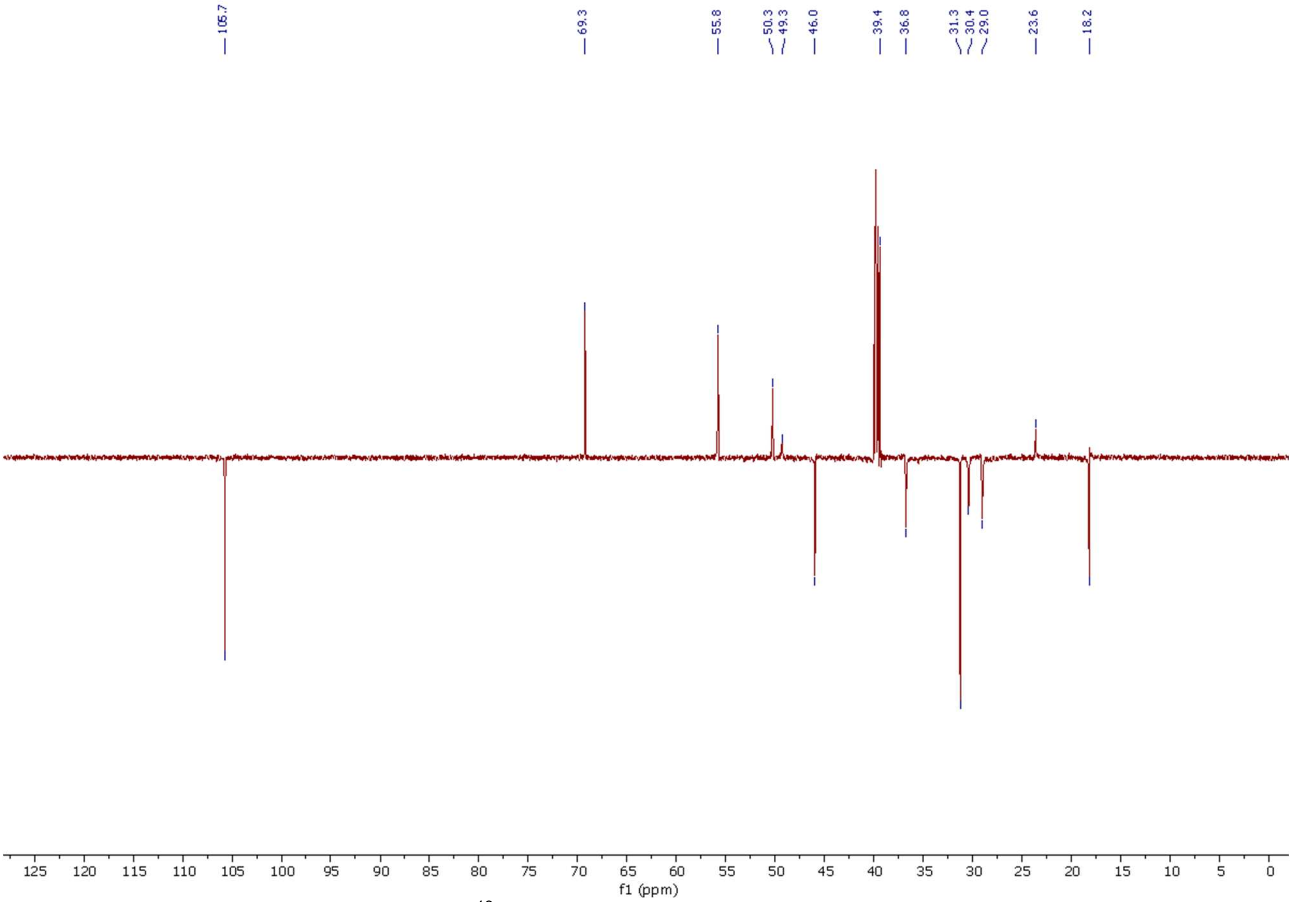

Figure N8.J ^{13}C DEPT 135 NMR of **8** in DMSO-d$_6$ at 151 MHz.

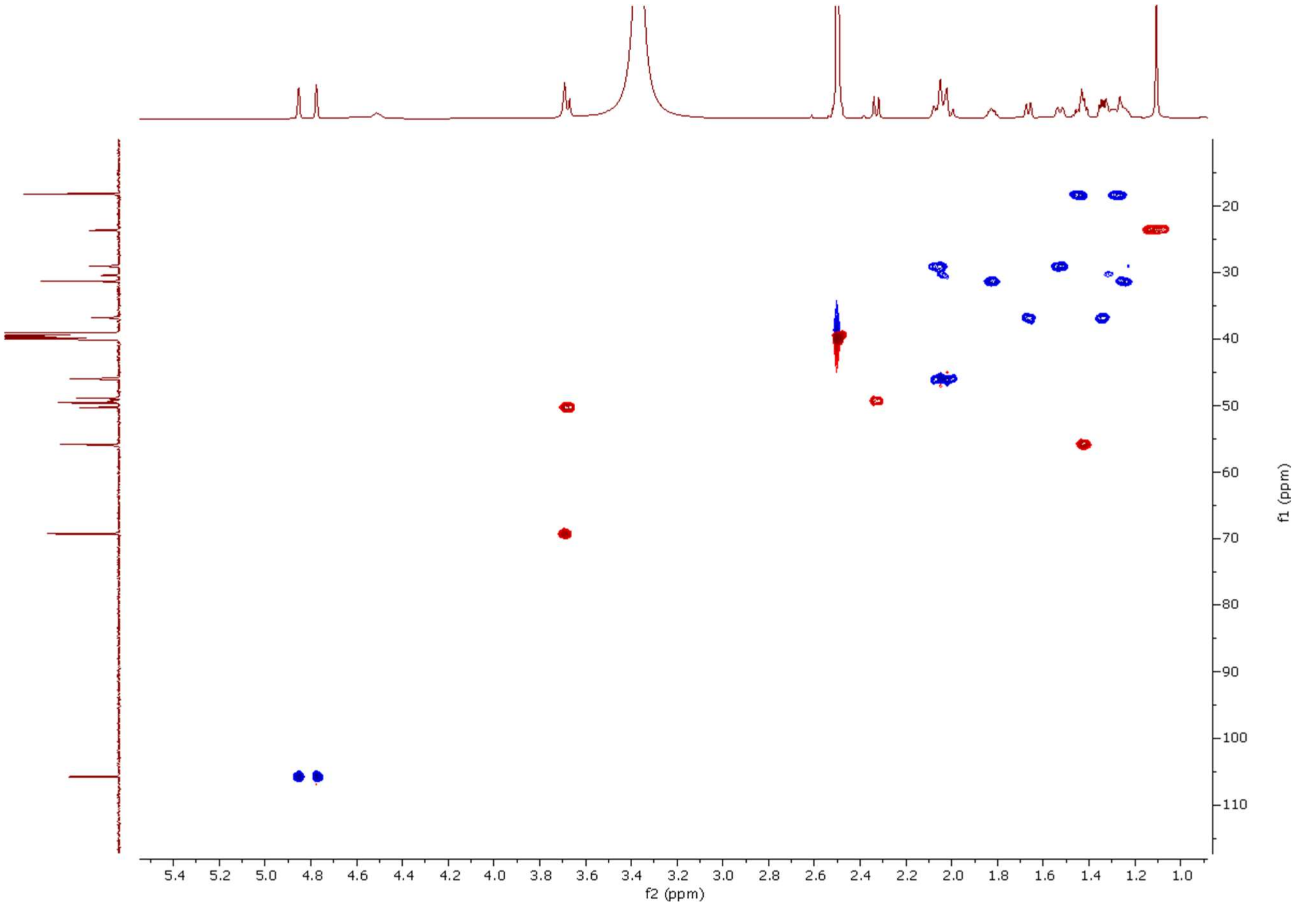

Figure N8.K HSQC NMR of **8** in DMSO-d6.

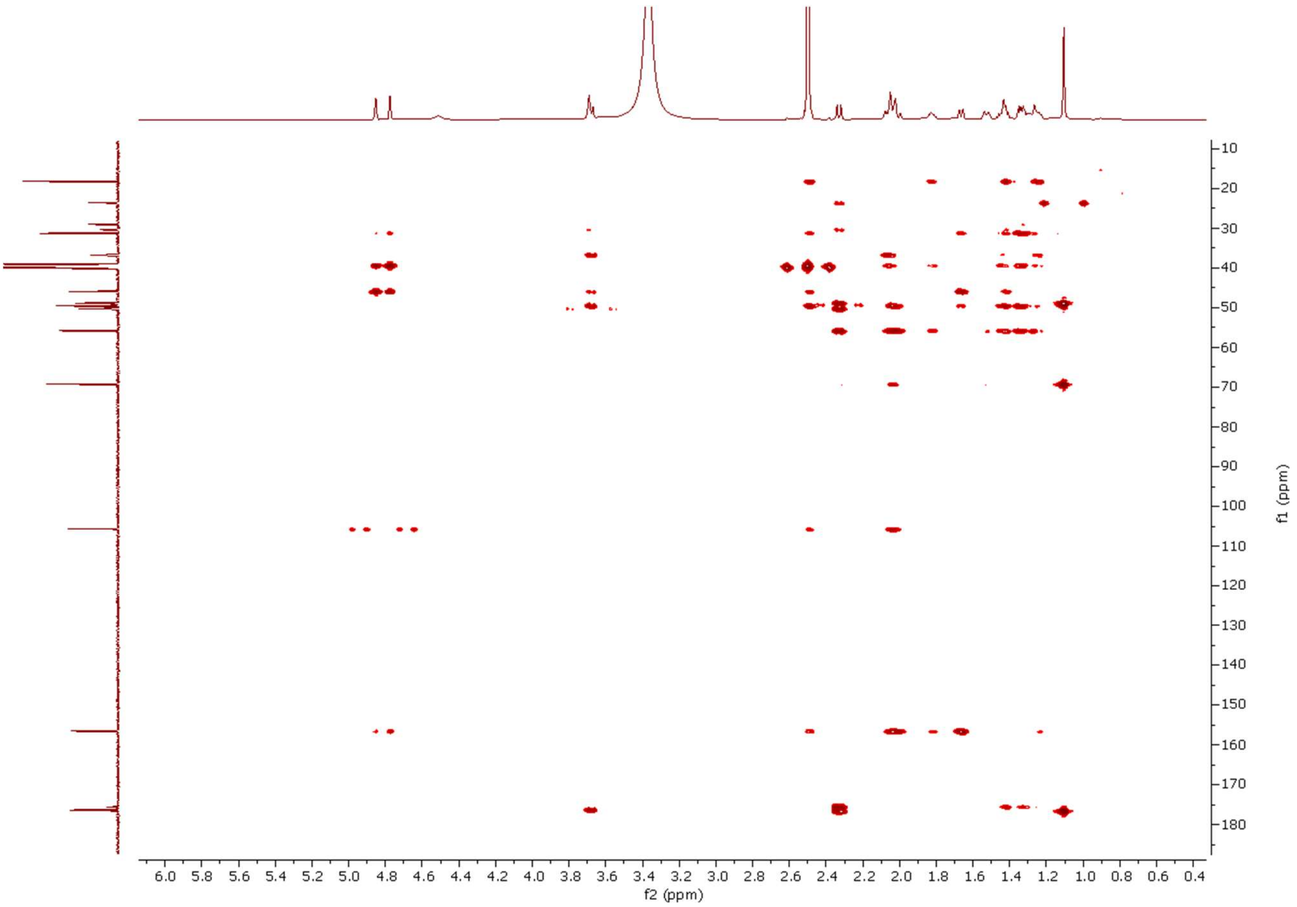

Figure N8.L HMBC NMR of **8** in DMSO-d6.

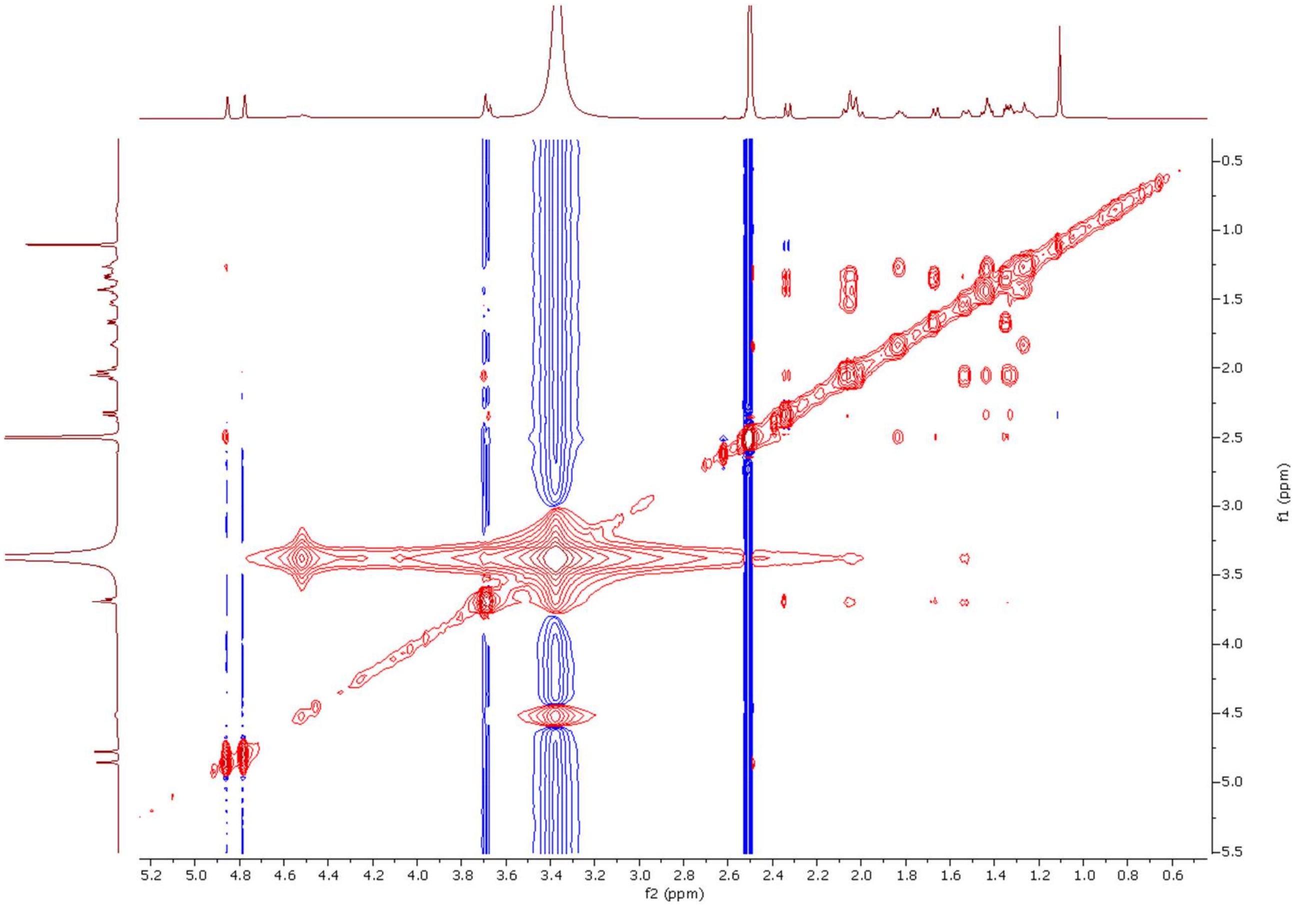

Figure N8.M NOESY NMR of **8** in DMSO-d_6 at 600 MHz.

Gibberellin A_{36} dimethyl ester (9a)

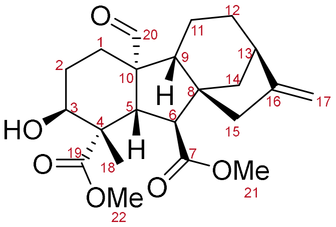

GA_{36} dimethyl ester (**9a**)
Chemical Formula: $C_{22}H_{30}O_6$
Exact Mass: 390.2042

	Measured in $CDCl_3$, 298K		
**Pos.**	δ_C 151 MHz	δ_H *(J/Hz)* 600 MHz	**Selected NOESY correlations to H** [a]
**1**	26.5	ax 1.38, overlapped<br>eq 2.17, ddd (13.0, 4.5, 2.7)	H-2a, H-1eq<br>H-1ax
**2**	28.4	a 1.72, overlapped<br>b 1.90, overlapped	H-3, H-1eq<br>H-3, H-1eq
**3**	71.3	eq 4.12, br s	H_3-18, H-2a, H-2b
**4**	49.5	-	-
**5**	48.7	2.76, d (13.0)	H-9, H_3-18
**6**	50.1	3.92, d (13.0)	H_3-20
**7**	174.9	-	-
**8**	50.2	-	-
**9**	56.4	1.68, dd (10.8, 7.4)	H-5
**10**	60.4	-	-
**11**	18.0	a 1.16, m<br>b 1.51, m	
**12**	32.0	a 1.38, overlapped<br>b 1.89, overlapped	
**13**	39.5	2.63, dd (6.0, 6.0)	H-17a, H-14a, H-14b
**14**	38.1	a 1.59, overlapped<br>b 1.71, overlapped	H-14b<br>H-14a
**15**	46.1	a 2.00, ddd (15.9, 2.9, 2.9)<br>b 2.27, br d (15.9)	H-17b, H-15b<br>H-17b, H-15a
**16**	156.1	-	-
**17**	106.7	b 4.84, br s<br>a 4.92, br s	H-15a, H-15b, H-17a<br>H-17b, H-13, H-12a
**18**	23.5	1.23, s	H-5, H-3
**19**	176.0	-	-
**20**	205.3	9.70, br s	H-6, H-14a, H-11a, H-2b, H-1eq
**21**	51.7	3.73, s	
**22**	51.8	3.64, s	

[a] Key NOESY correlations are shown in blue text.

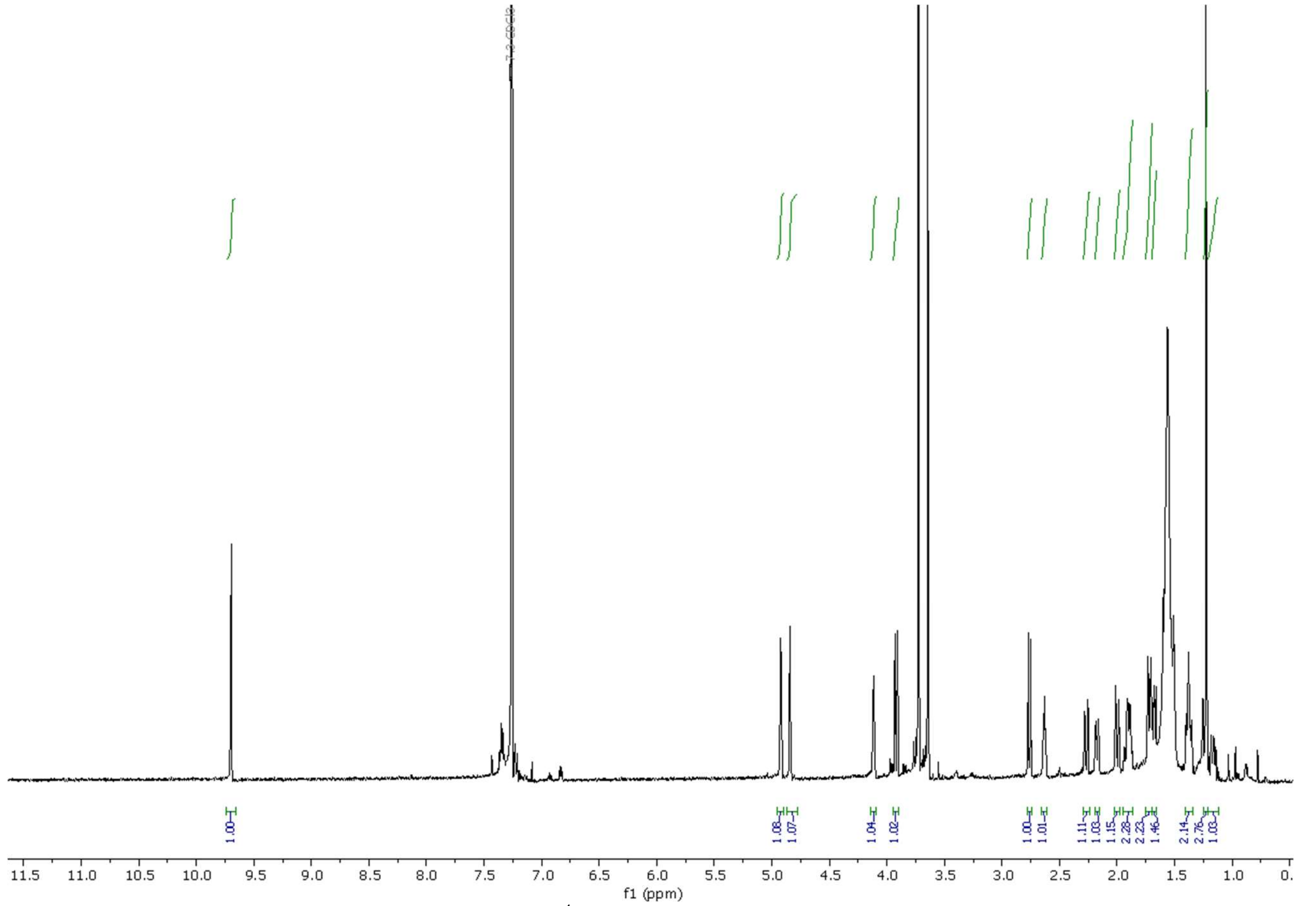

Figure N9a.A ^{1}H NMR of **9a** in $CDCl_3$ at 600 MHz.

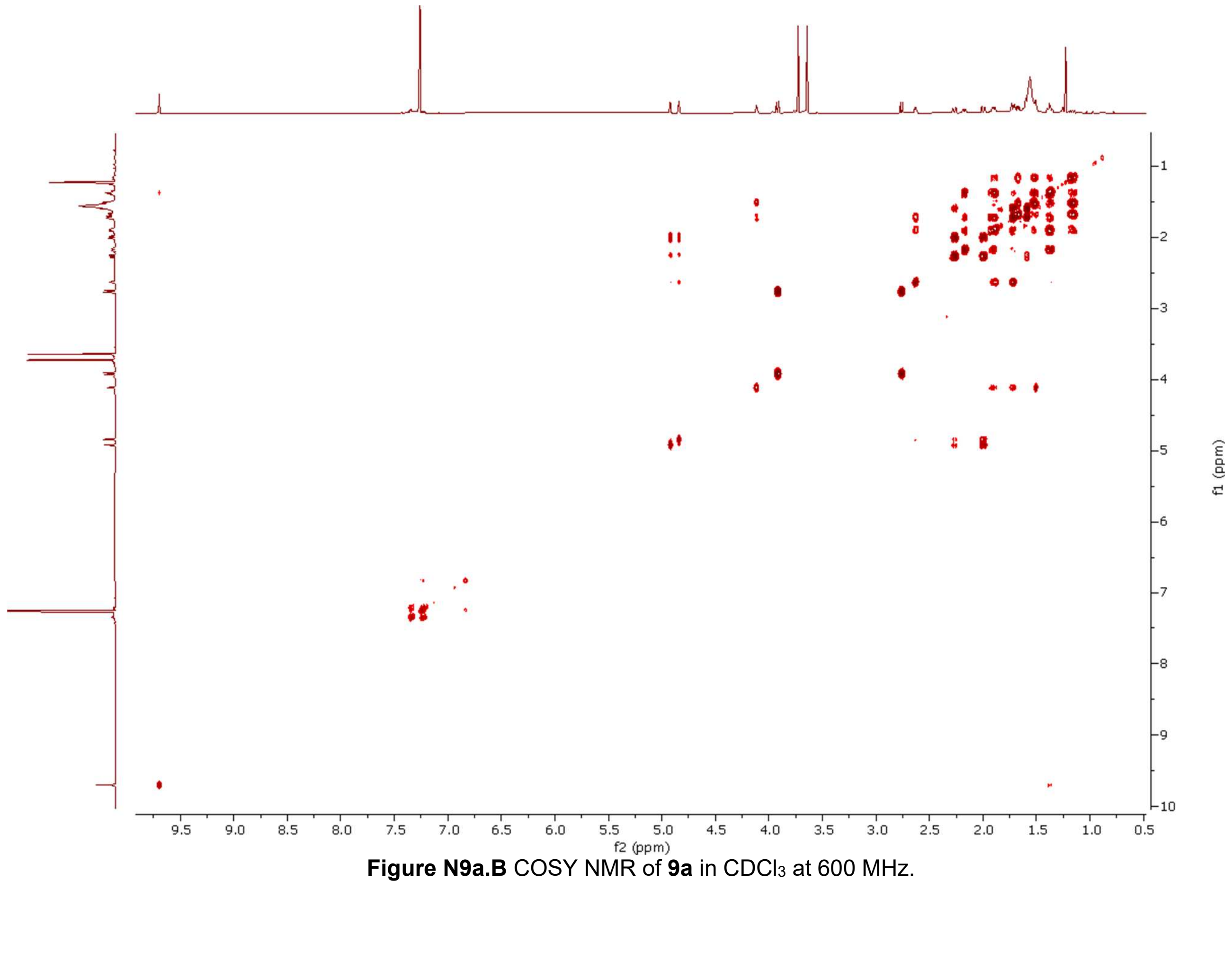

Figure N9a.B COSY NMR of **9a** in $CDCl_3$ at 600 MHz.

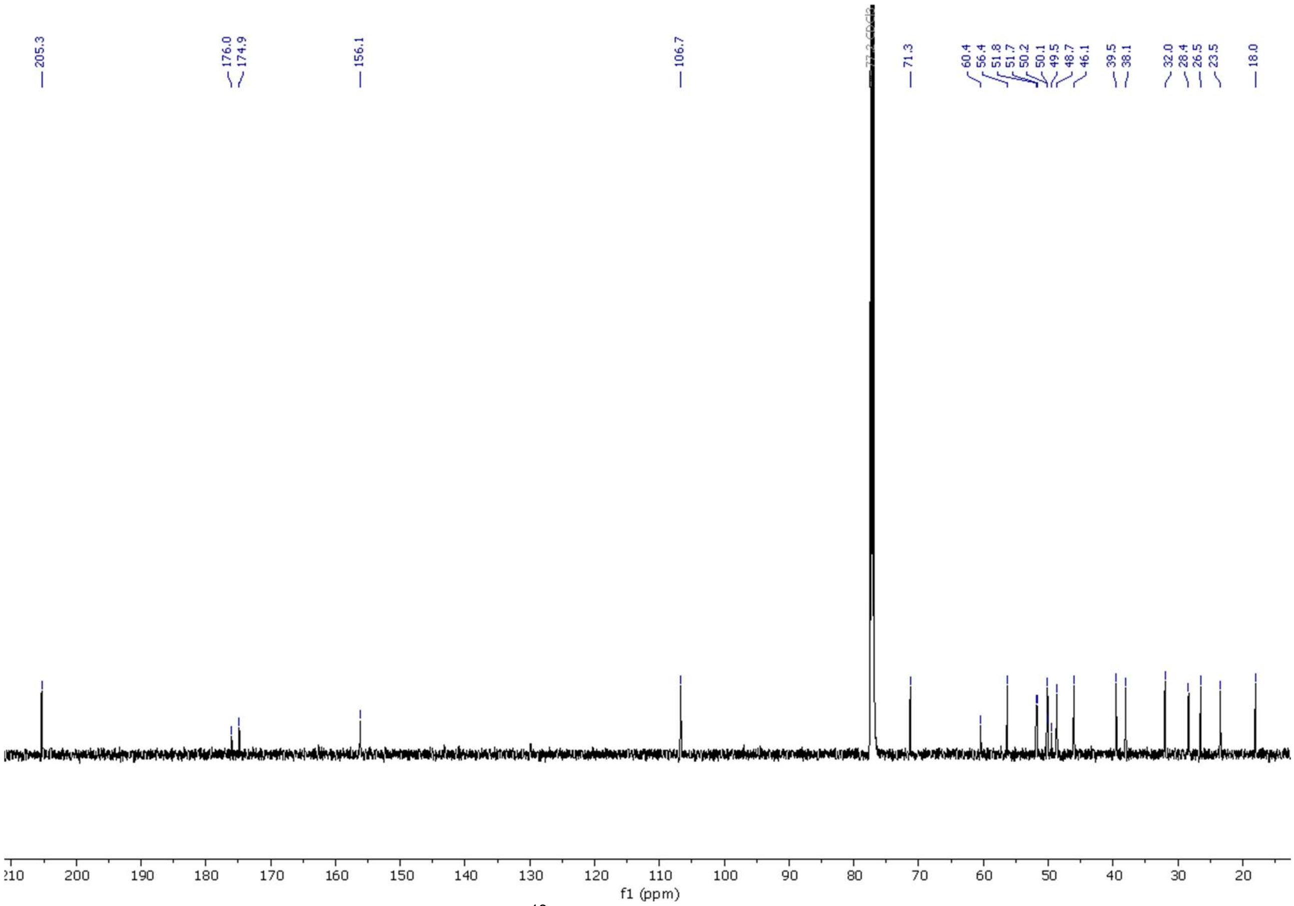

Figure N9a.C ^{13}C NMR of **9a** in $CDCl_3$ at 151 MHz.

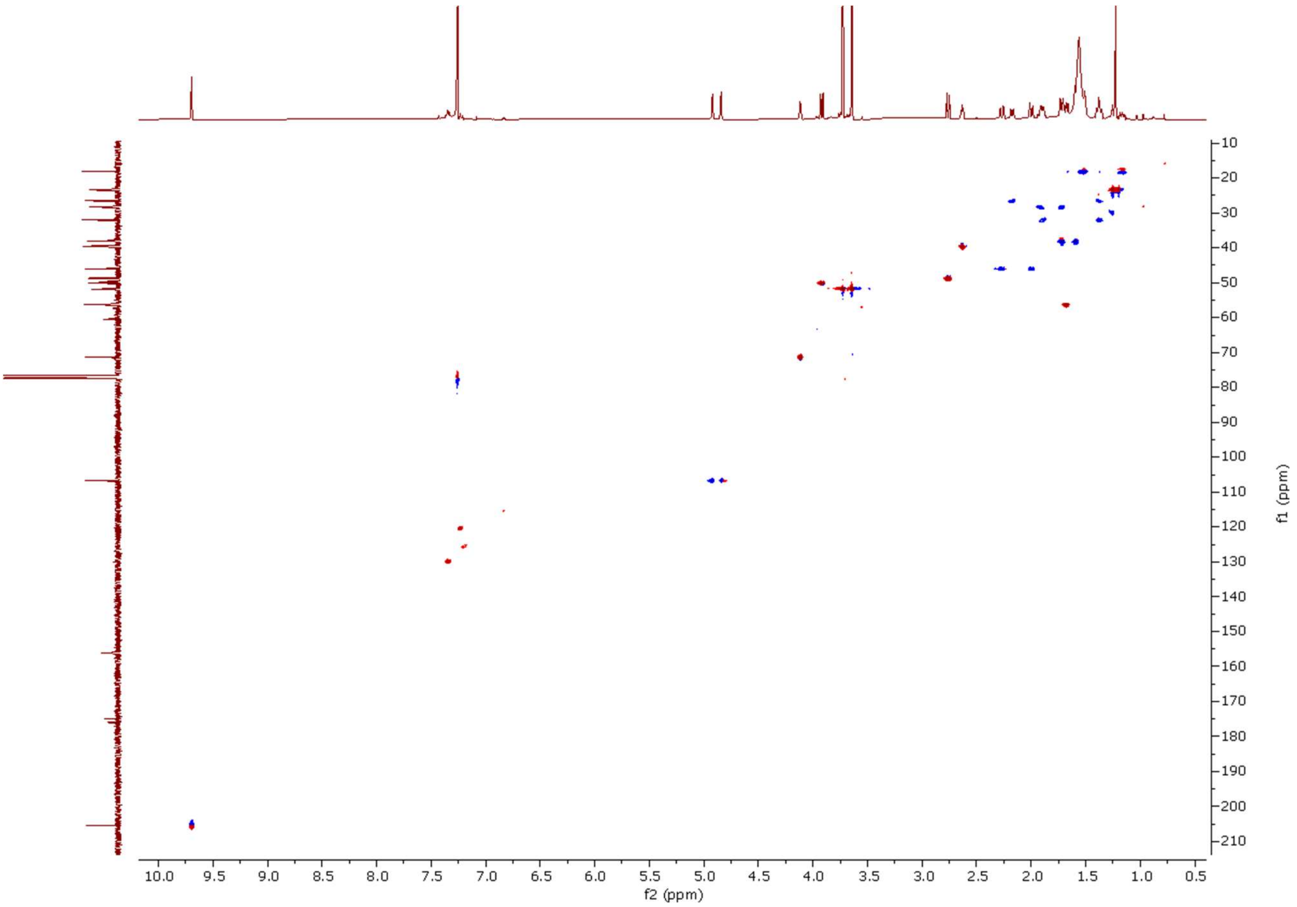

Figure N9a.D HSQC NMR of **9a** in $CDCl_3$.

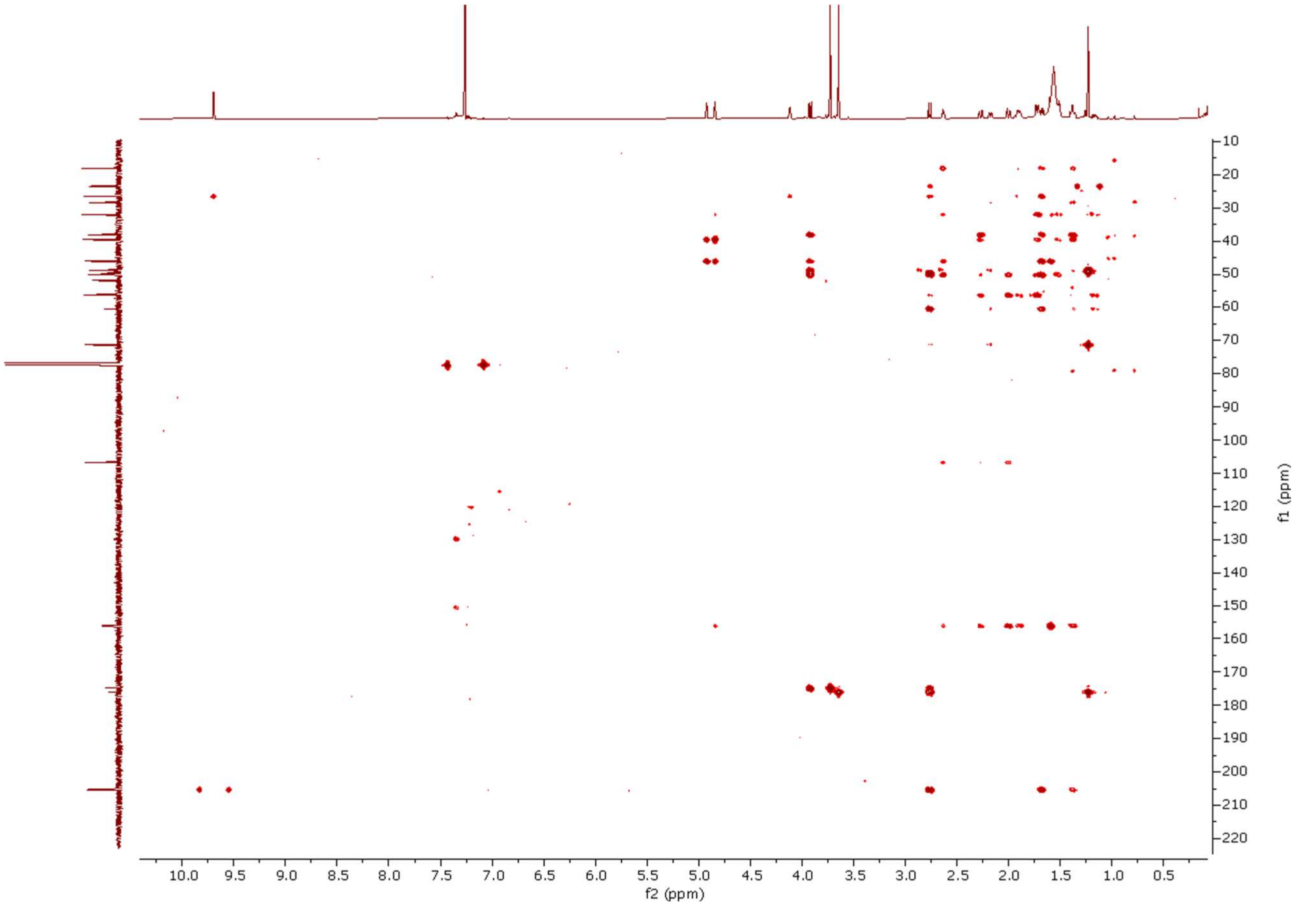

Figure N9a.E HMBC NMR of **9a** in $CDCl_3$.

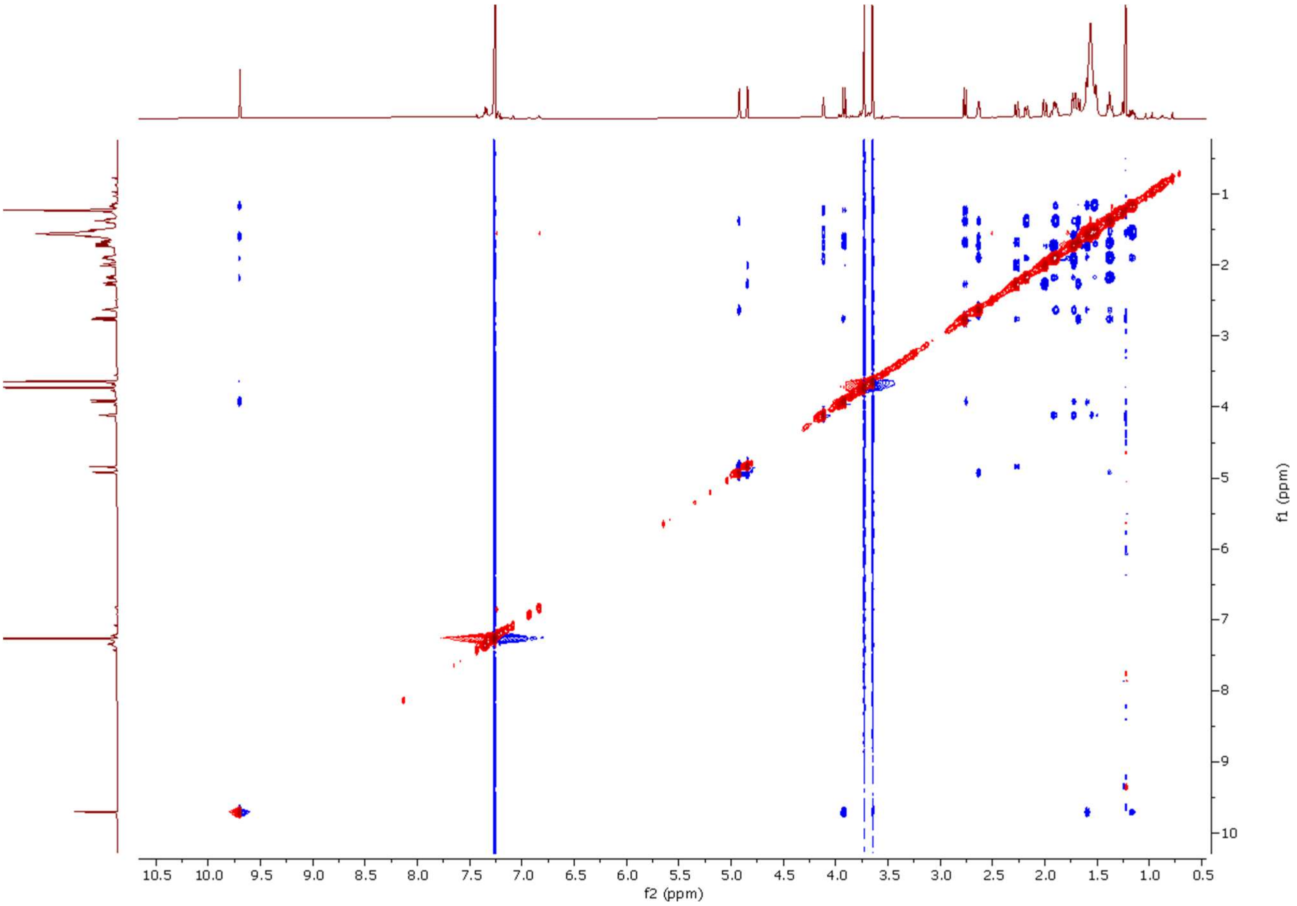

Figure N9a.F NOESY NMR of **9a** in $CDCl_3$ at 600 MHz.

Gibberellin A_{123} (10)

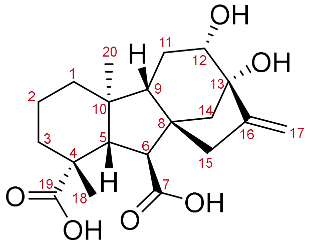

GA_{123} (**10**)
Chemical Formula: $C_{20}H_{28}O_6$
Exact Mass: 364.1886

	Measured in DMSO-d_6, 298K		
**Pos.**	δ_C 151 MHz	δ_H (*J/Hz*) 600 MHz	**Selected NOESY correlations to H** [a]
**1**	40.6	ax. 0.89, overlapped<br>eq. 1.55, overlapped	
**2**	19.7	1.23, m<br>1.71, m	
**3**	39.6	ax. 0.74, m<br>eq. 2.04, br d (13.2)	
**4**	44.5	-	-
**5**	57.2	1.34, overlapped	
**6**	49.3	2.82, d (12.9)	H_3-20
**7**	176.6	-	-
**8**	45.5	-	-
**9**	53.7	0.88, overlapped	
**10**	42.1	-	-
**11**	28.7	1.17, m<br>1.65, ddd (13.8, 7.8, 6.8)	
**12**	74.9	3.3, dd (7.8, 7.8)	
**13**	77.9	-	-
**14**	39.9	1.33, overlapped<br>1.54, overlapped	
**15**	43.0	1.80, d (15.8)<br>3.68, overlapped [COSY&NOESY]	
**16**	157.9	-	-
**17**	106.9	4.86, br s<br>5.02, br s	
**18**	28.5	1.10, s	
**19**	178.8	-	-
**20**	15.4	0.69, s	H-6

[a] Key NOESY correlations are shown in blue text.

[COSY&NOESY] = detected based on COSY and NOESY.

Complete NMR data including better NOESY data are recorded for the methylated analogue **10a**.

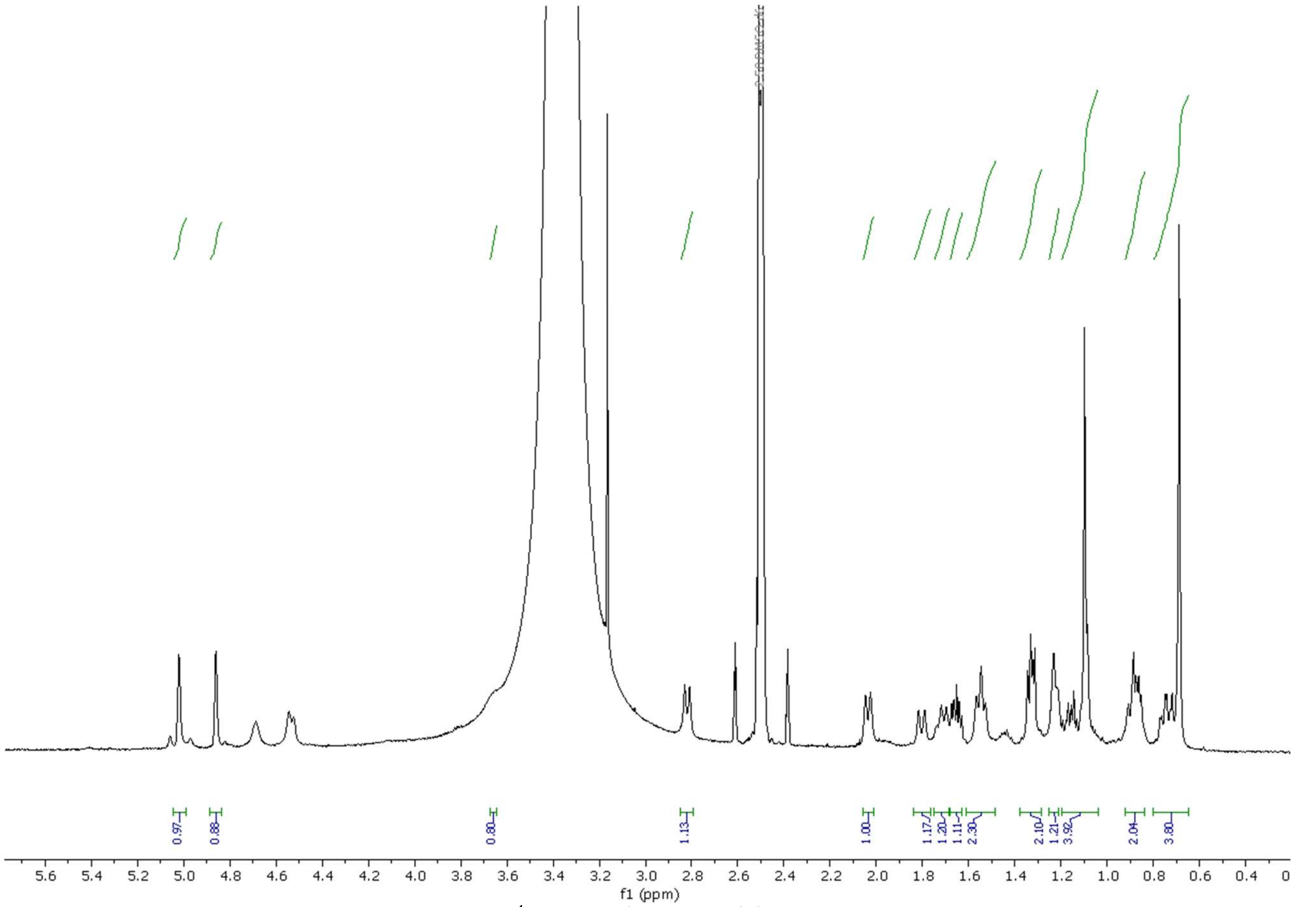

Figure N10.A ^{1}H NMR of **10** in DMSO-d_6 at 600 MHz.

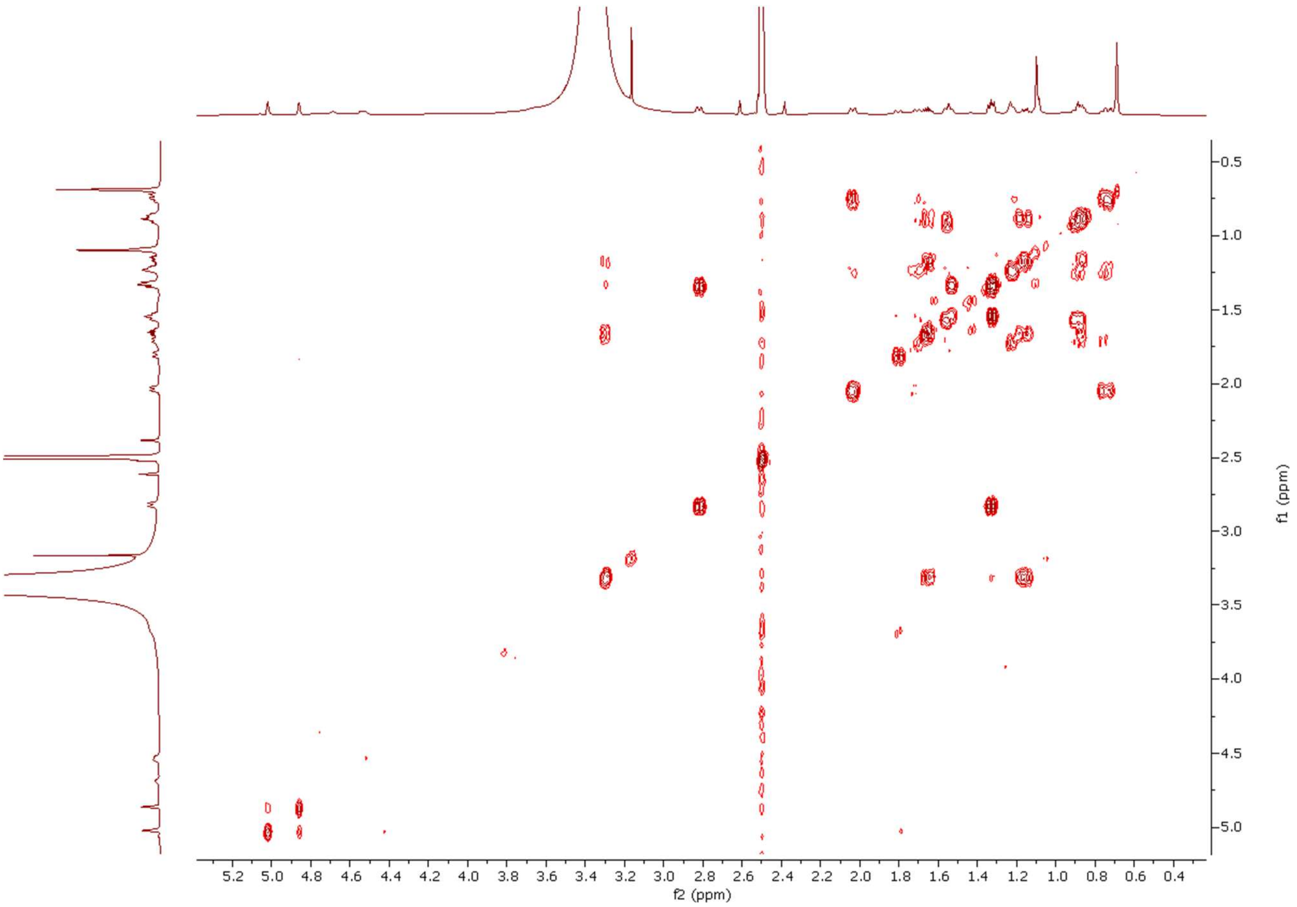

Figure N10.B COSY NMR of **10** in DMSO-d_6 at 600 MHz.

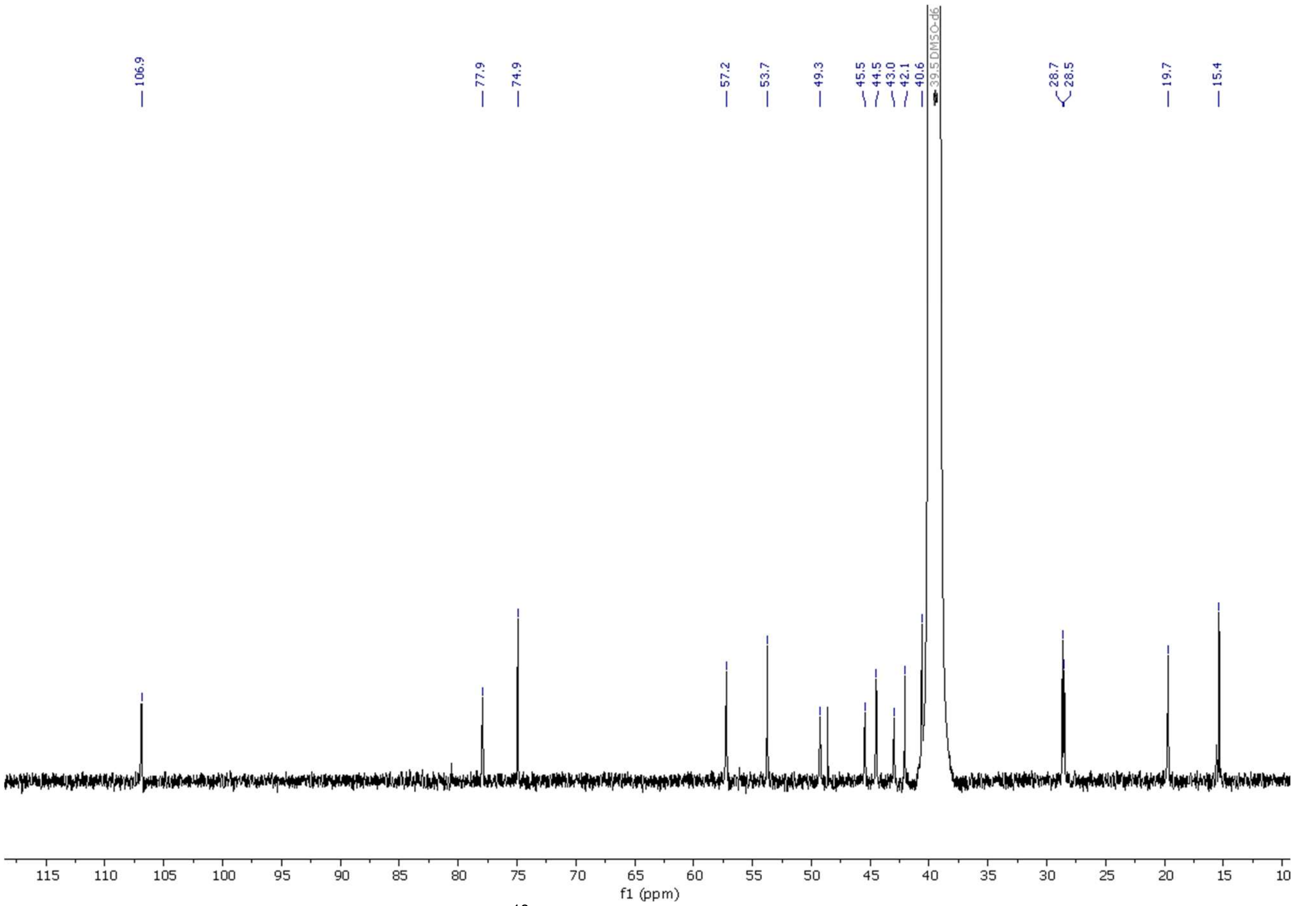

Figure N10.C ^{13}C NMR of **10** in DMSO-d_6 at 151 MHz.

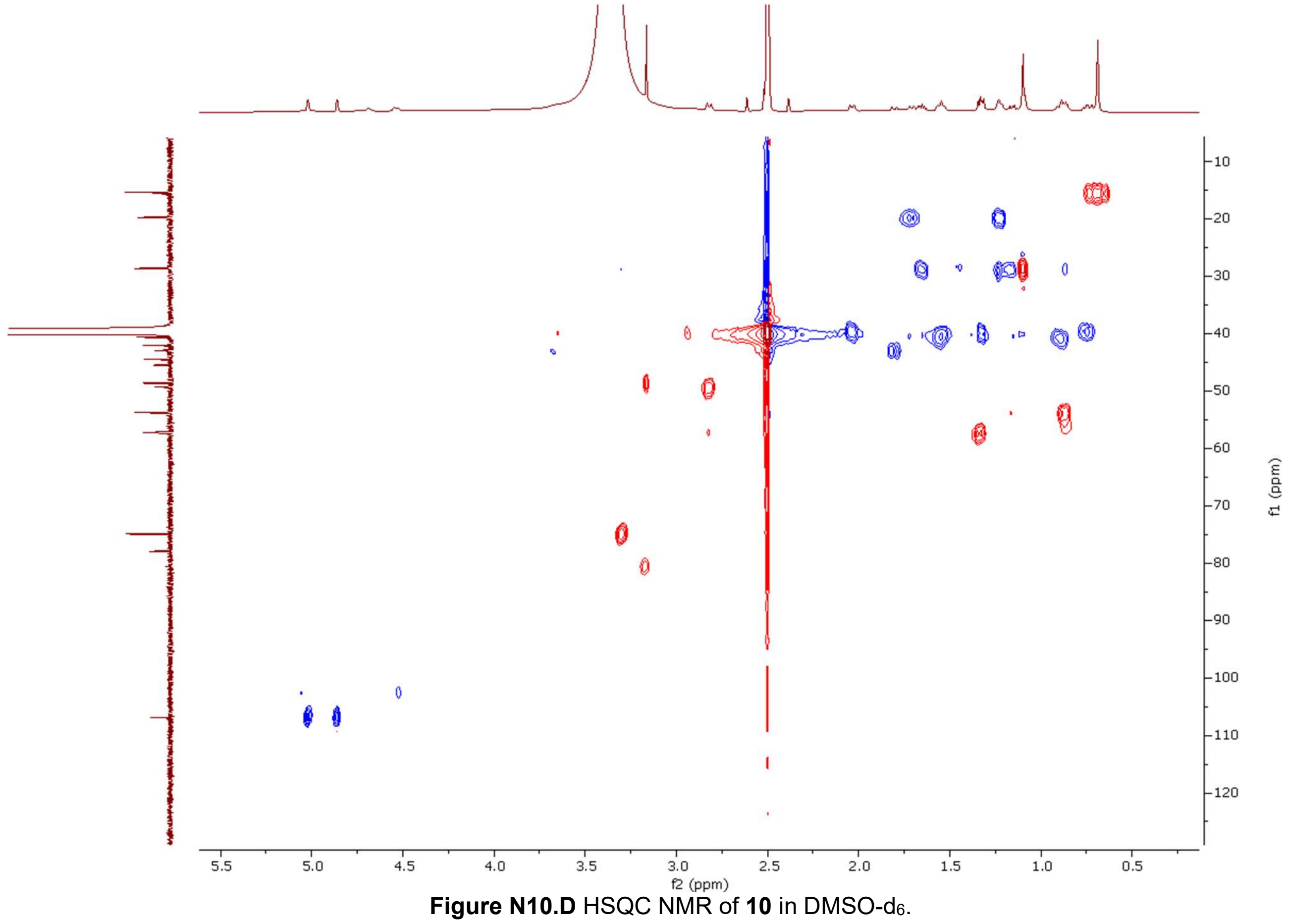

Figure N10.D HSQC NMR of **10** in DMSO-d_6.

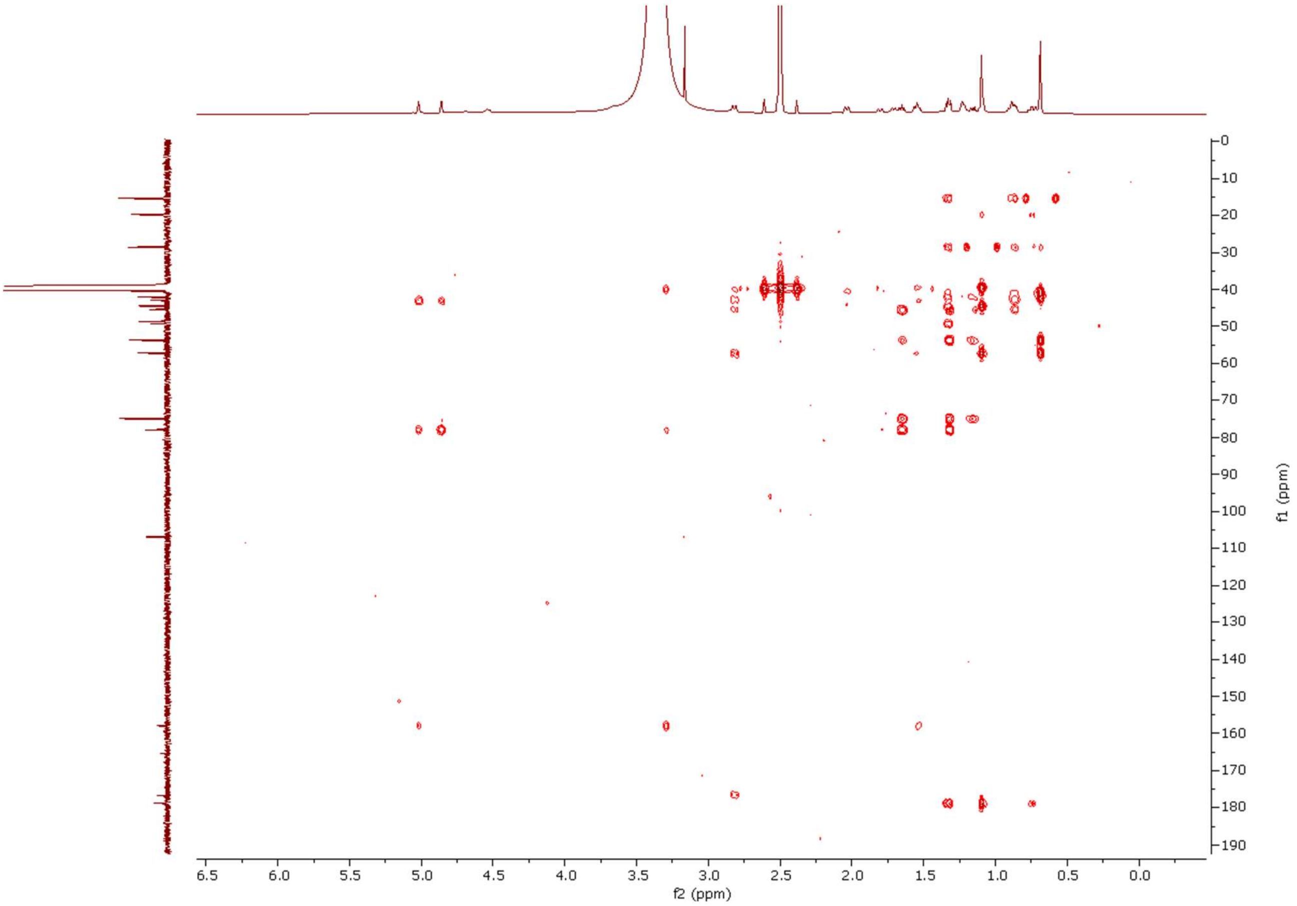

Figure N10.E HMBC NMR of **10** in DMSO-d6.

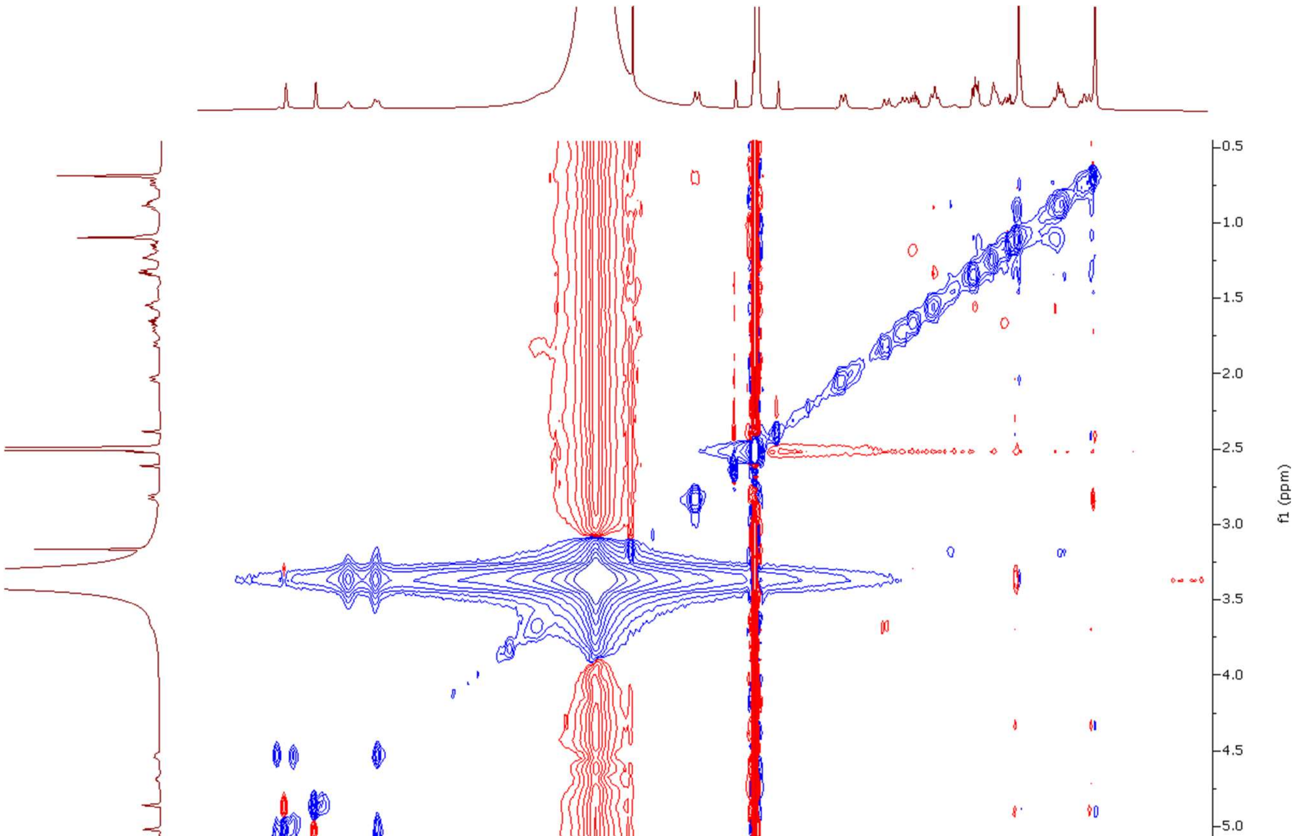

Figure N10.F NOESY NMR of **10** in DMSO-d6 at 600 MHz.

Gibberellin A_{123} dimethyl ester (10a)

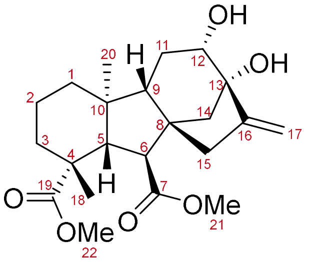

GA_{123} dimethyl ester (**10a**)
Chemical Formula: $C_{22}H_{32}O_6$
Exact Mass: 392.2199

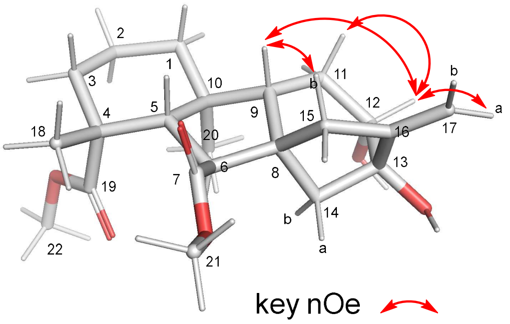

	Measured in $CDCl_3$, 298K		
**Pos.**	δ_C<br>151 MHz	δ_H (*J/Hz*)<br>600 MHz	**Selected NOESY correlations to H** [a]
**1**	39.7	ax 1.00, ddd (13.5, 13.5, 4.3)<br>eq 1.62, overlapped	H-1eq, H-9<br>H-1ax, H_3-20
**2**	19.8	a 1.49, m<br>b 1.77, m	 H_3-20
**3**	37.7	a 1.06, overlapped<br>b 2.20, overlapped	
**4**	44.6	-	-
**5**	57.1	1.87, d (12.5)	H-9
**6**	50.8	3.37, d (12.5)	H_3-20, H-14a, H-14b, H_3-18
**7**	175.5	-	-
**8**	48.1	-	-
**9**	54.4	1.28, dd (8.7, 8.7)	H-1ax, H-5, H-15b, H-11b
**10**	43.9	-	-
**11**	27.9	a 1.45, ddd (15.2, 8.7, 5.5)<br>b 1.97, ddd (15.2, 8.7, 7.4)	H_3-20, H-11b<br>H-11a, H-9
**12**	75.2	3.67, overlapped	H-17a, H-11b, H-9
**13**	80.0	-	-
**14**	41.6	a 1.56, overlapped<br>b 2.12, overlapped	H-6, H-14b<br>H_3-20, H-14a
**15**	45.2	a 2.12, overlapped<br>b 2.18, overlapped	H-17b<br>H-17b, H-9
**16**	154.0	-	-
**17**	108.8	b 5.05, br s<br>a 5.20, dd (3.2, 1.7)	H-17a, H-15b, H-15a<br>H-17b, H-12
**18**	29.1	1.09, s	H-5
**19**	177.7	-	-
**20**	14.7	0.72, s	H-6, H-14b, H-2b, H-1eq, H-11a
**21**	51.7	3.70, s	
**22**	51.6	3.67, s	

[a] Key NOESY correlations are shown in blue text.

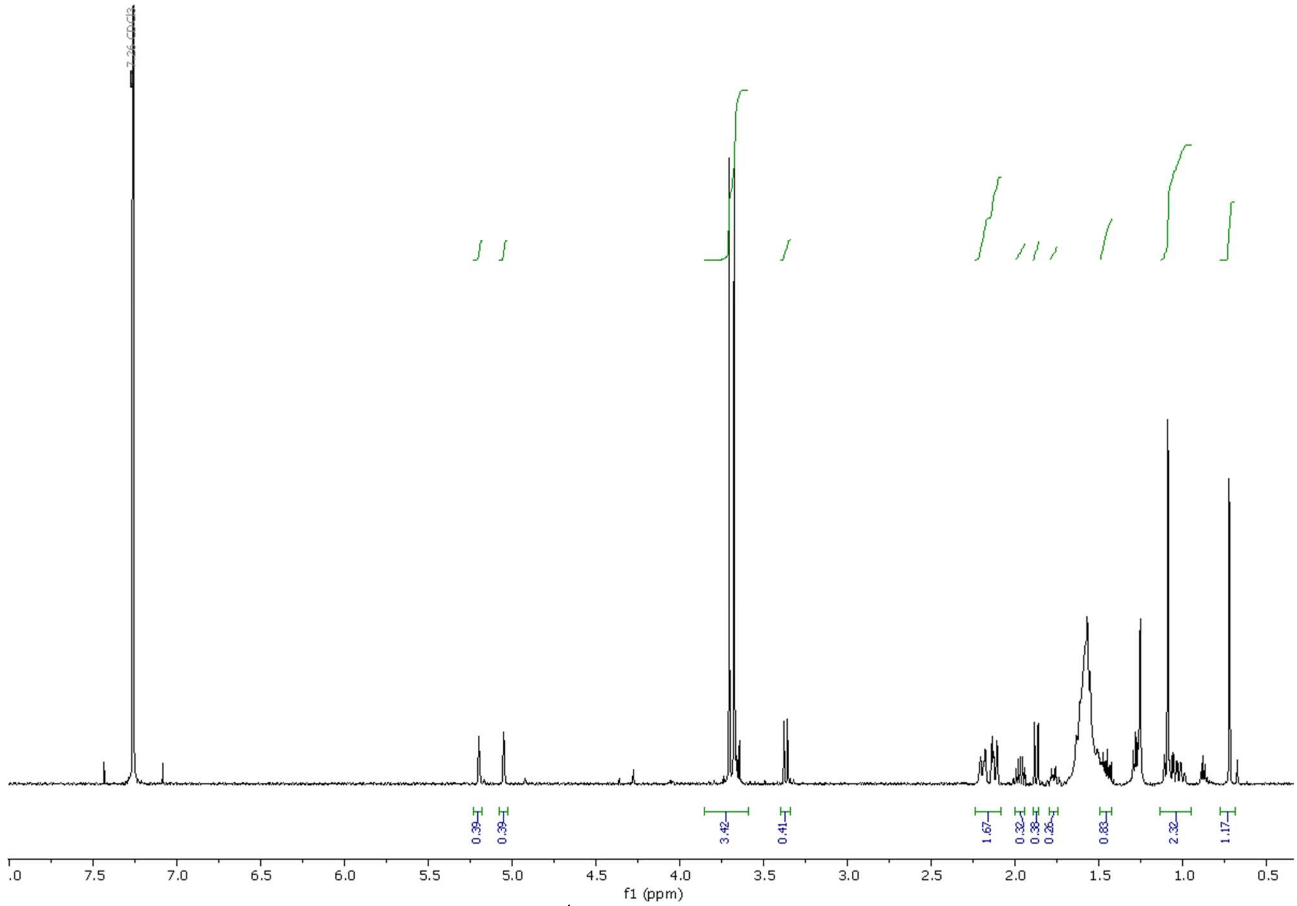

Figure N10a.A ^{1}H NMR of **10a** in $CDCl_3$ at 600 MHz.

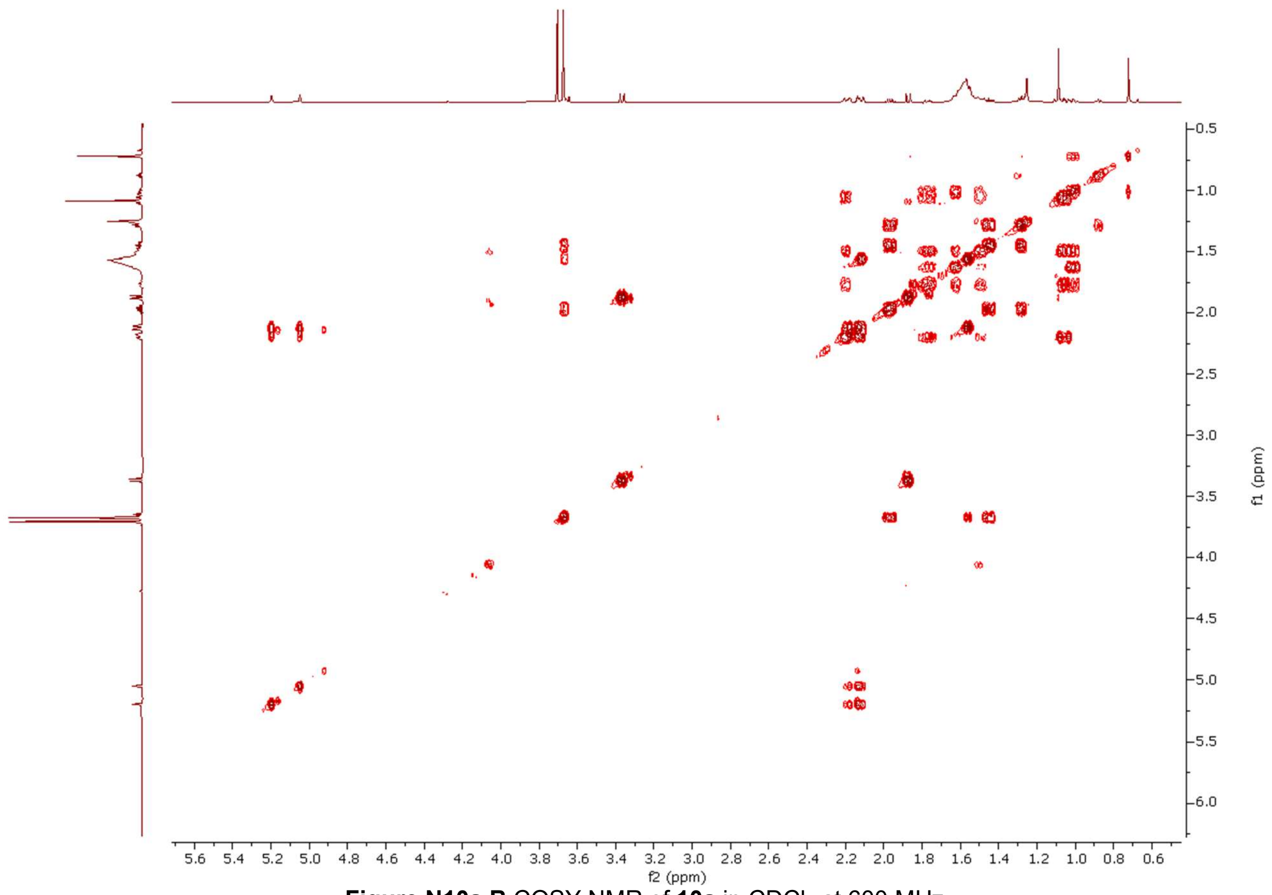

Figure N10a.B COSY NMR of **10a** in $CDCl_3$ at 600 MHz.

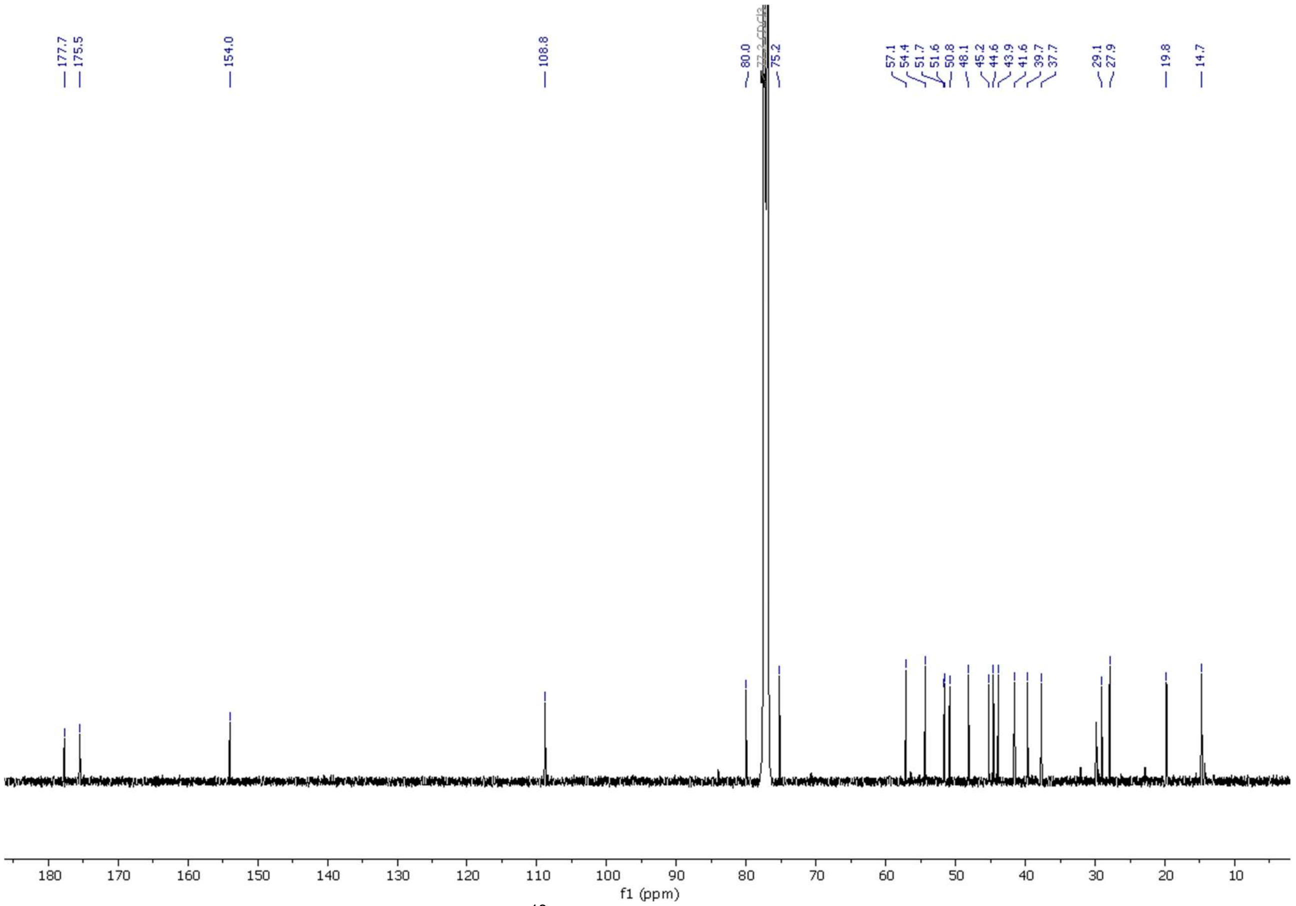

Figure N10a.C ^{13}C NMR of **10a** in $CDCl_3$ at 151 MHz.

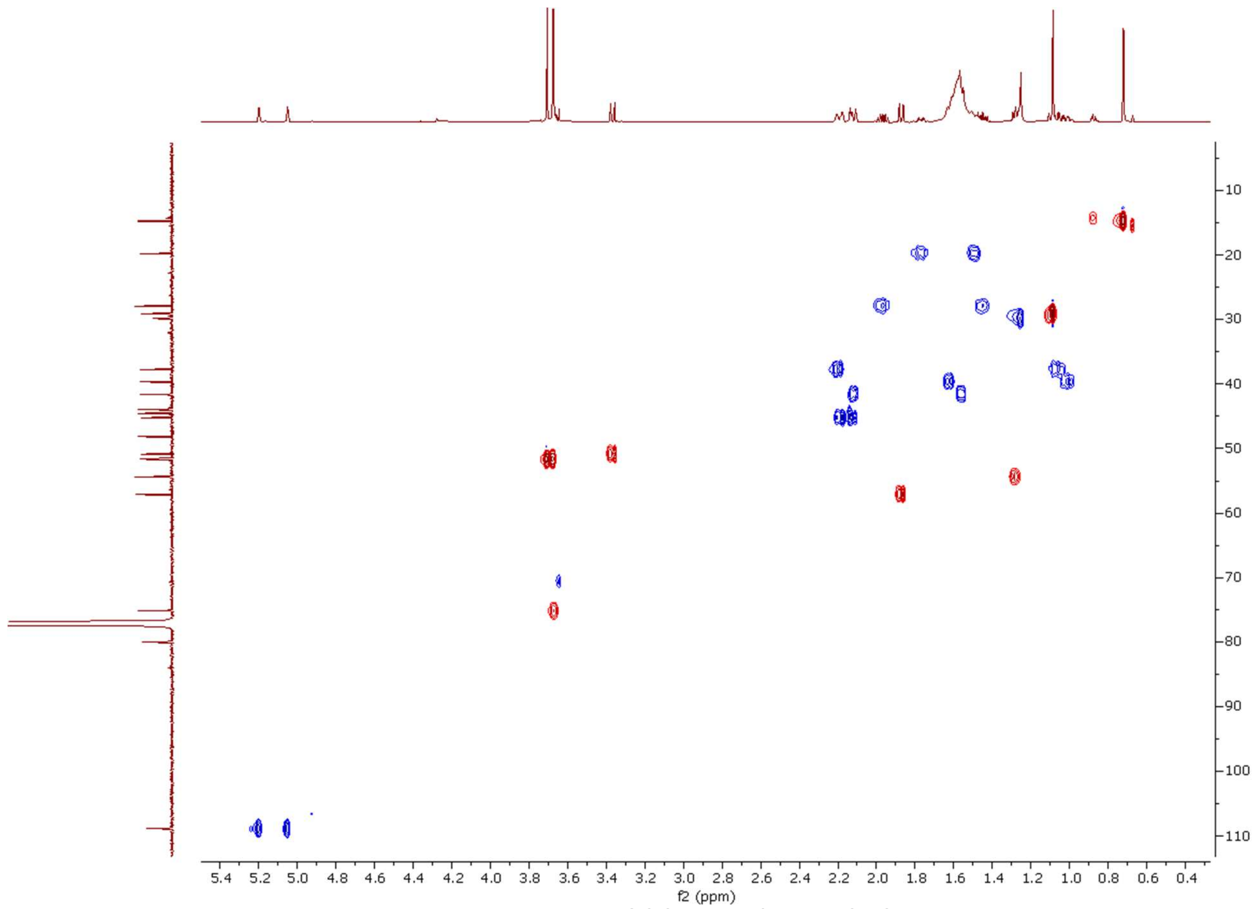

Figure N10a.D HSQC NMR of **10a** in $CDCl_3$.

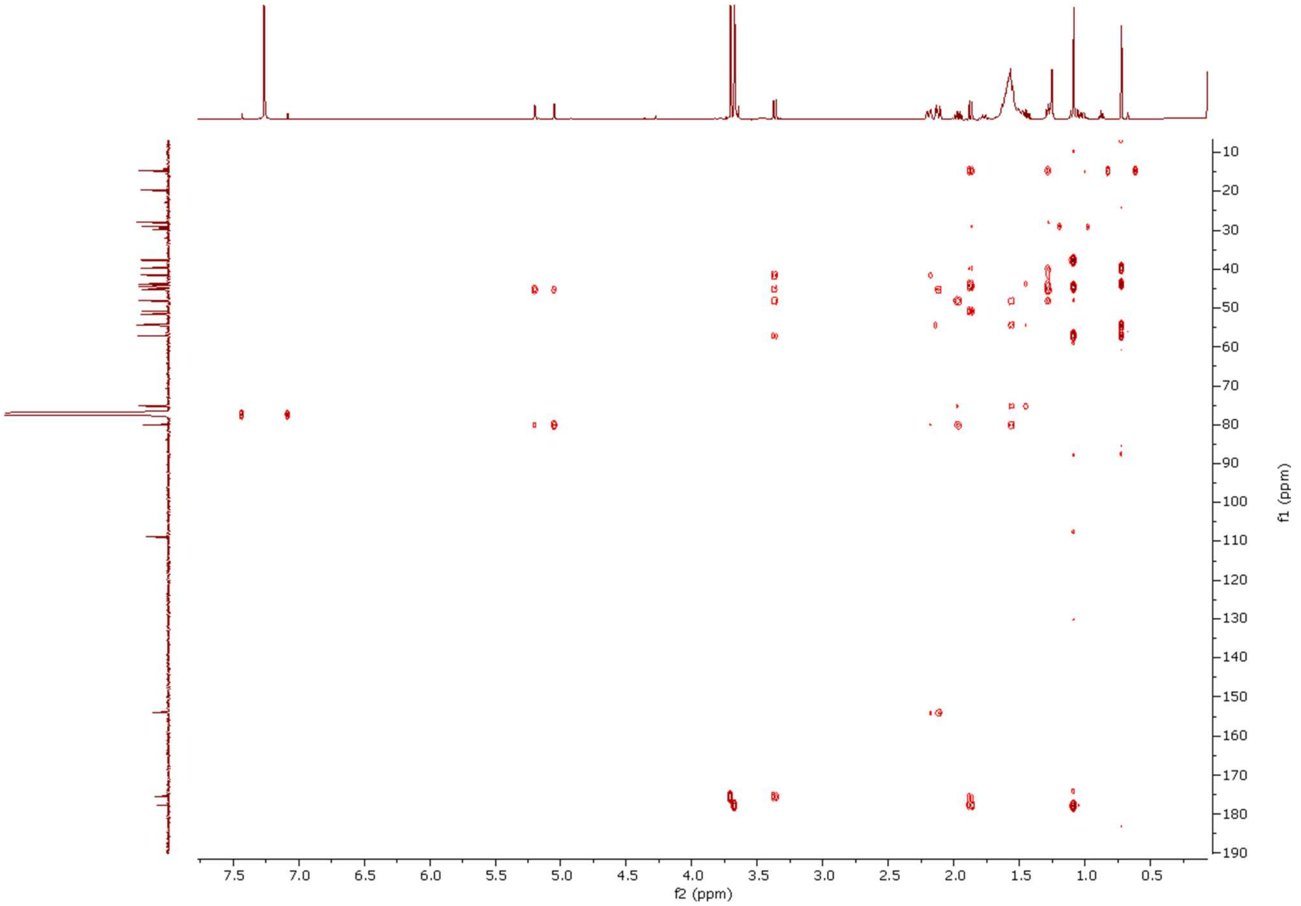

Figure N10a.E HMBC NMR of **10a** in $CDCl_3$.

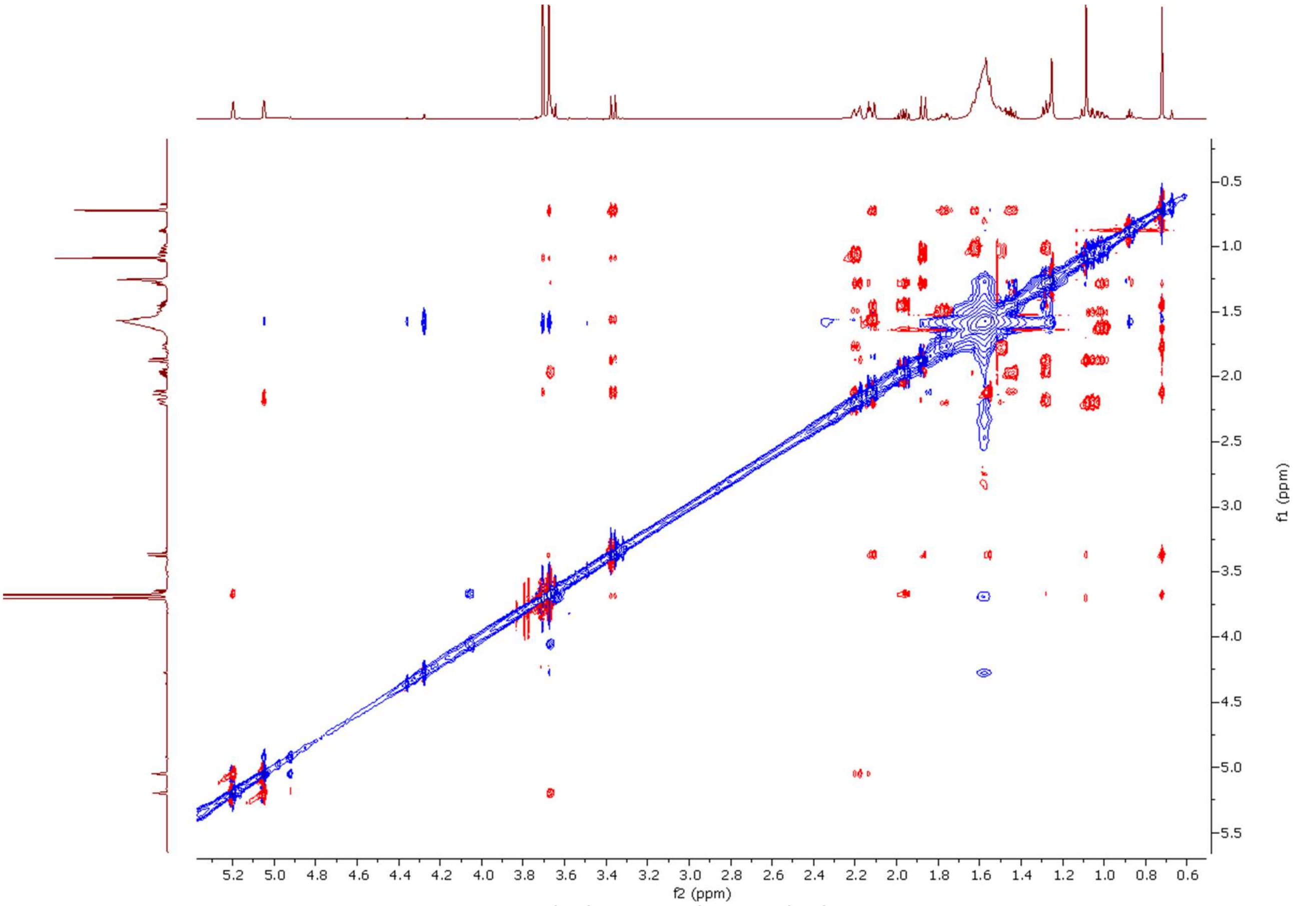

Figure N10a.F NOESY NMR of **10a** in $CDCl_3$ at 600 MHz.

Gibberellin A_{74} (11)

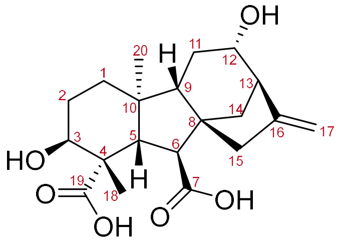

GA$_{74}$ (**11**)
Chemical Formula: $C_{20}H_{28}O_6$
Exact Mass: 364.1886

	Measured in DMSO-d$_6$, 298K		
**Pos.**	δ_C 151 MHz	δ_H (J/Hz) 600 MHz	**Selected NOESY correlations to H** [a]
**1**	34.0	ax 1.24, m<br>eq 1.37, overlapped	H-1eq<br>H-1ax
**2**	27.1	a 1.37, overlapped<br>b 2.00, m	H-2b<br>H-2a, H_3-20
**3**	70.6	3.75, br s	H_3-18, H-2a, H-2b
**4**	48.1	-	-
**5**	50.9 HMBC	1.77, overlapped	
**6**	49.3	2.93, d (12.5)	
**7**	176.6	-	-
**8**	47.5	-	-
**9**	53.6	0.85, dd (7.1, 6.9)	
**10**	42.2	-	-
**11**	28.2	a 1.03, m<br>b 1.54, overlapped	H-11b, H_3-20<br>H-11a
**12**	74.8	3.43, dd (7.8, 7.8)	H-17a
**13**	50.0	2.35, d (4.9)	H-14a
**14**	32.9	a 1.34, overlapped<br>b 1.54, overlapped	H-13
**15**	44.7 HMBC	a 1.77, overlapped<br>b n.d.	H-17b
**16**	154.9 HMBC	-	-
**17**	106.8	b 4.83, s<br>a 4.90, s	H-17a, H-15a<br>H-13, H-17b, H-12
**18**	23.7	1.13, s	
**19**	179.3	-	-
**20**	15.4	0.71, s	H-11a, H-2b

[a] Key NOESY correlations are shown in blue text.

HMBC = detected based on HMBC.

n.d.= not detected.

Complete NMR data including better NOESY data are recorded for the methylated analogue **11a**.

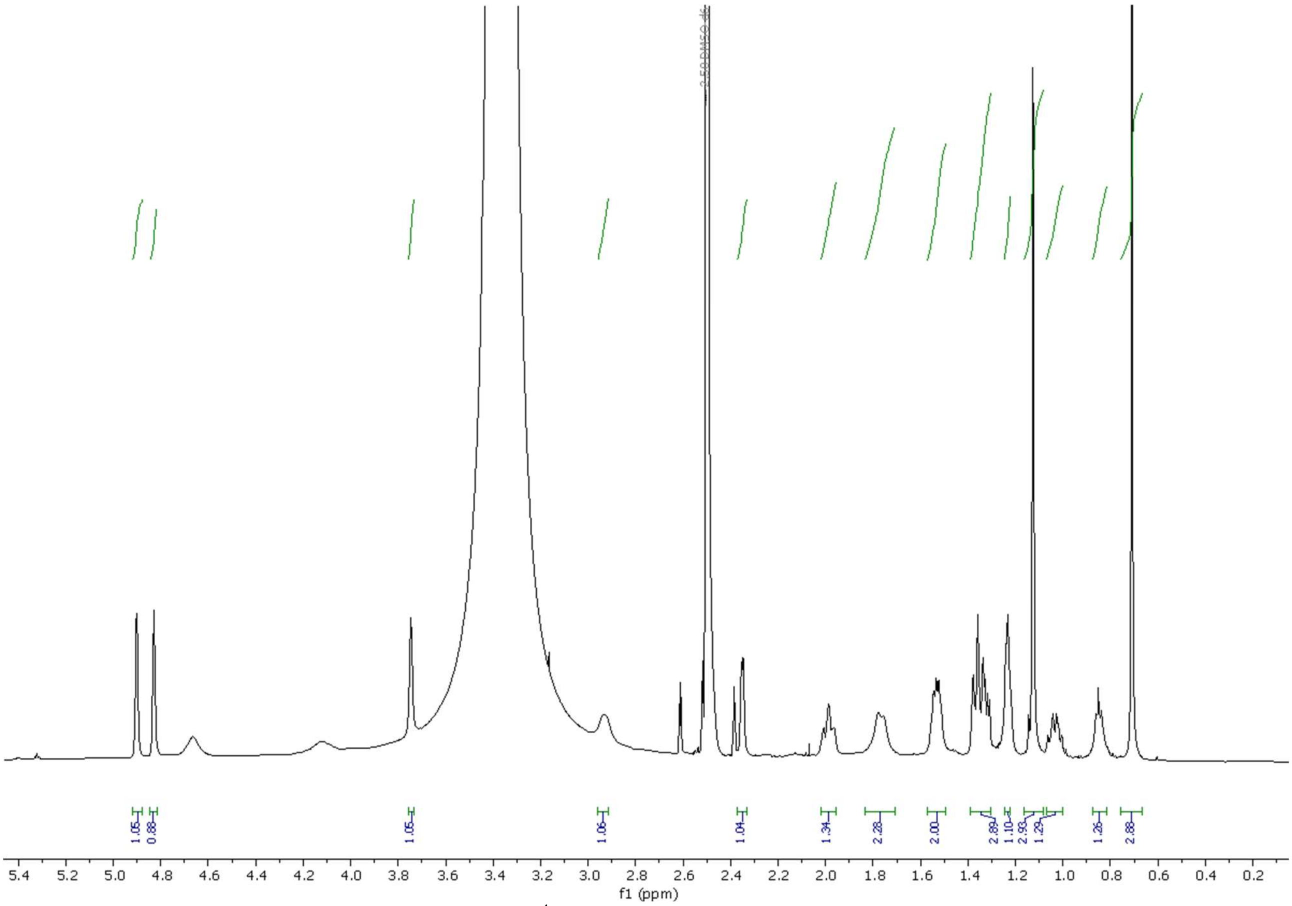

Figure N11.A ^{1}H NMR of **11** in DMSO-d_6 at 600 MHz.

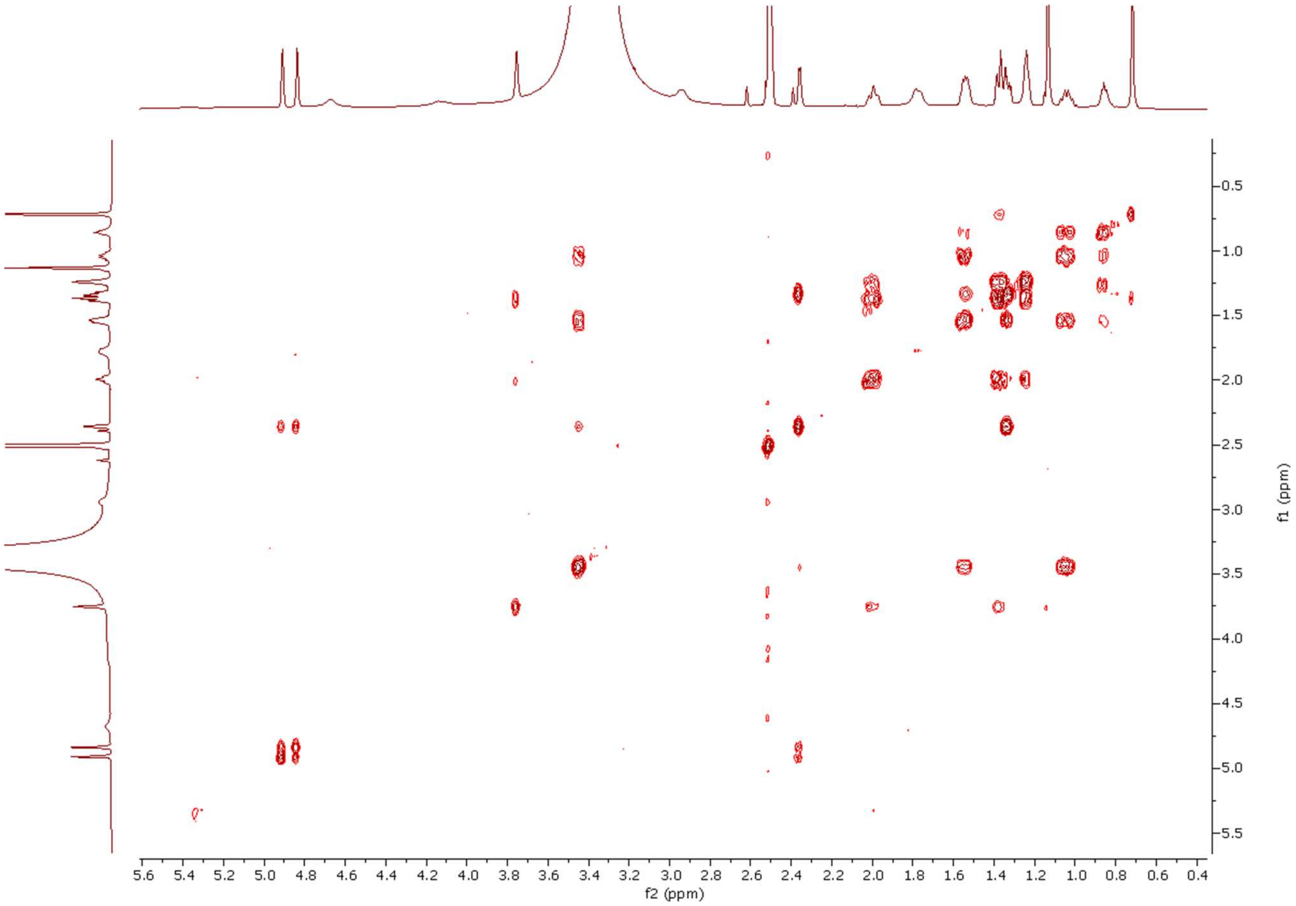

Figure N11.B COSY NMR of **11** in DMSO-d_6 at 600 MHz.

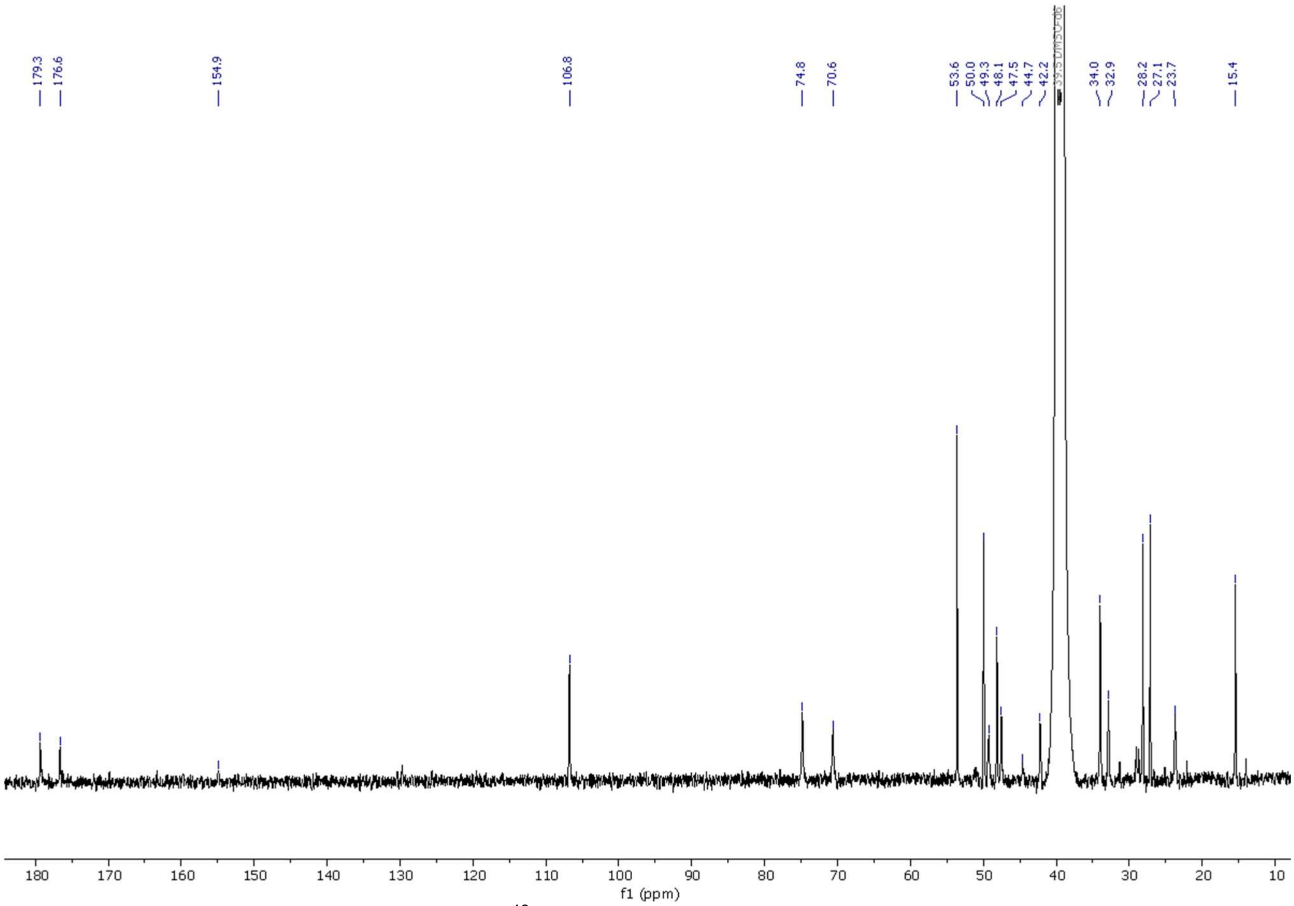

Figure N11.C ^{13}C NMR of **11** in DMSO-d_6 at 151 MHz.

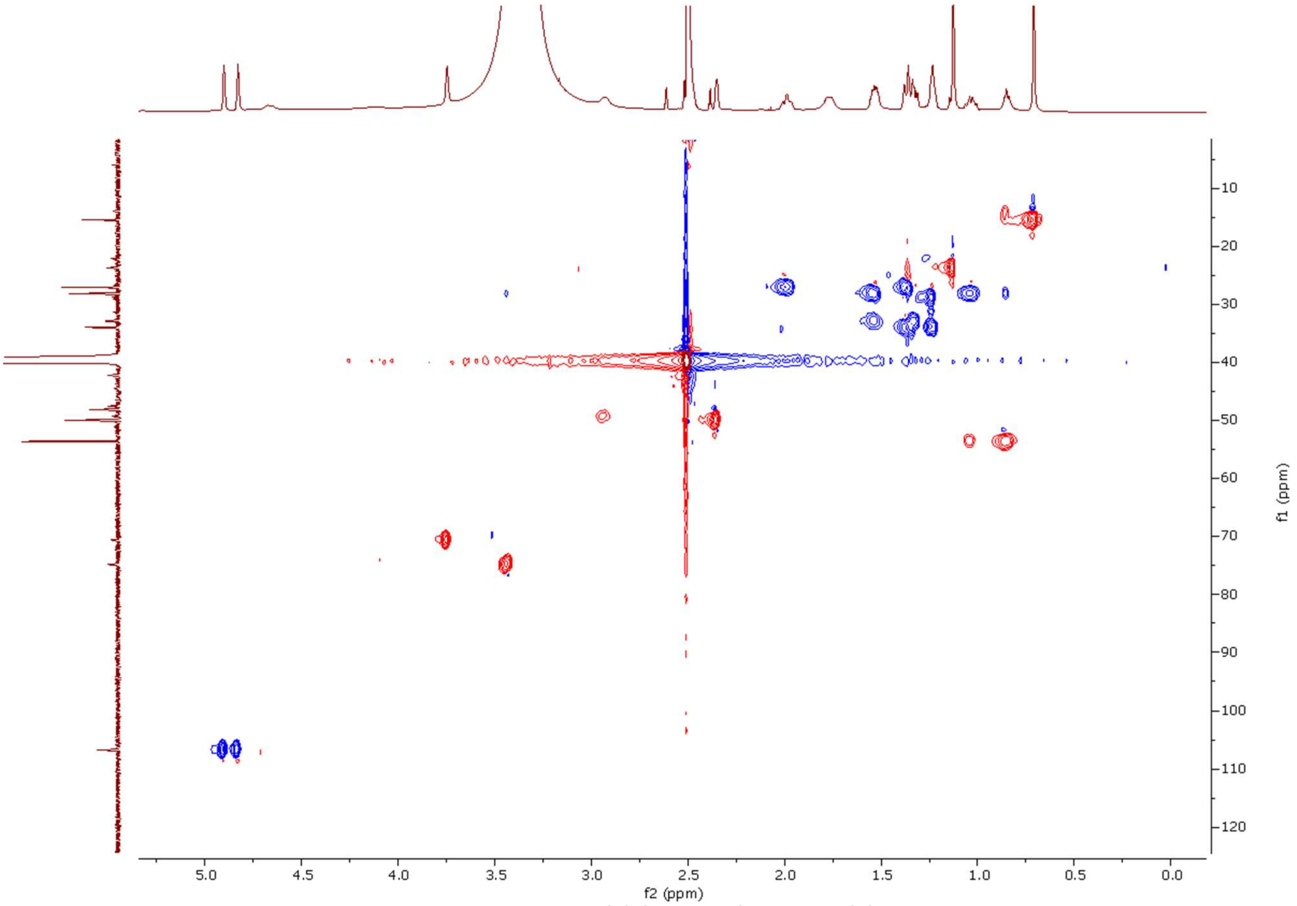

Figure N11.D HSQC NMR of **11** in DMSO-d_6.

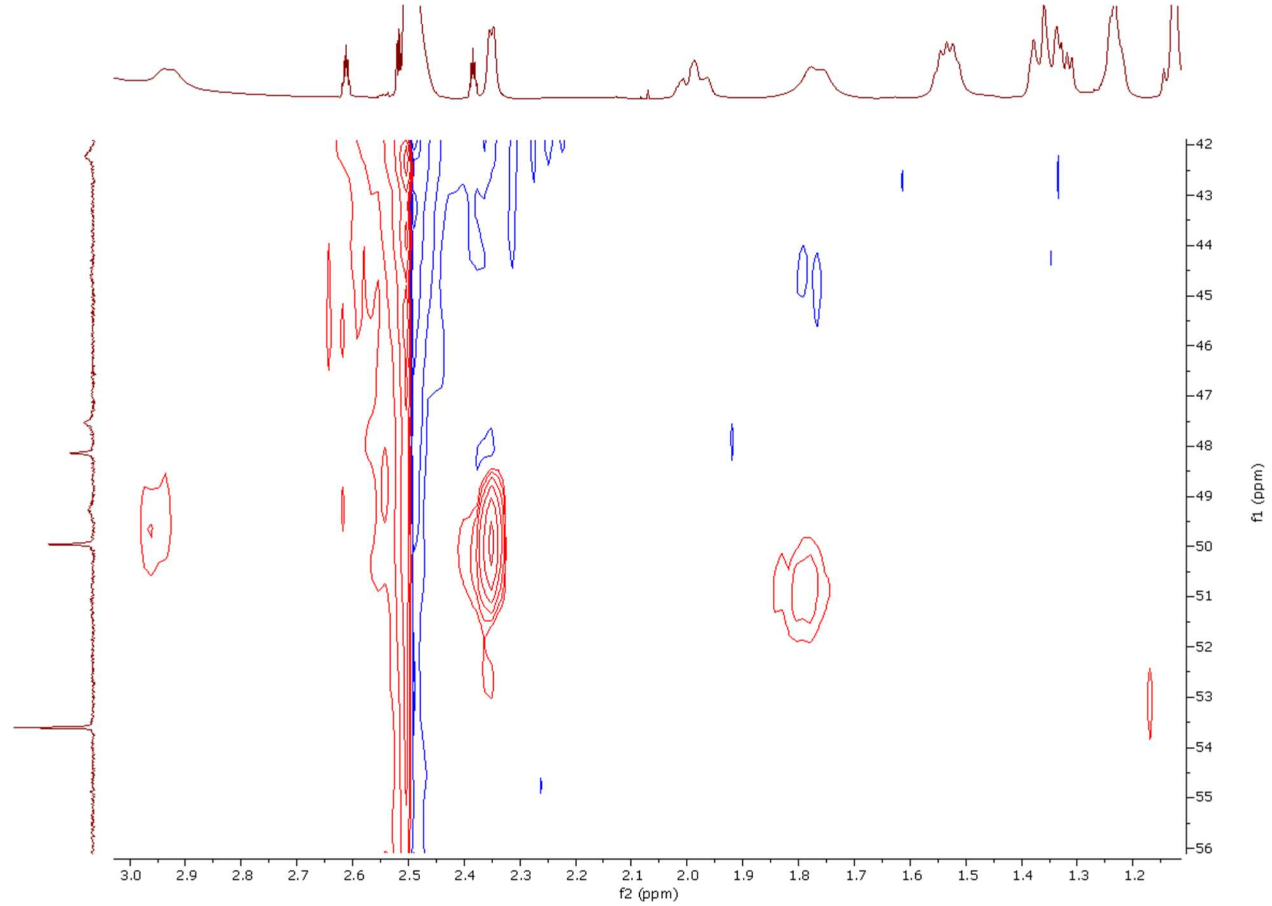

Figure N11.E HSQC (zoomed in) NMR of **11** in DMSO-d6.

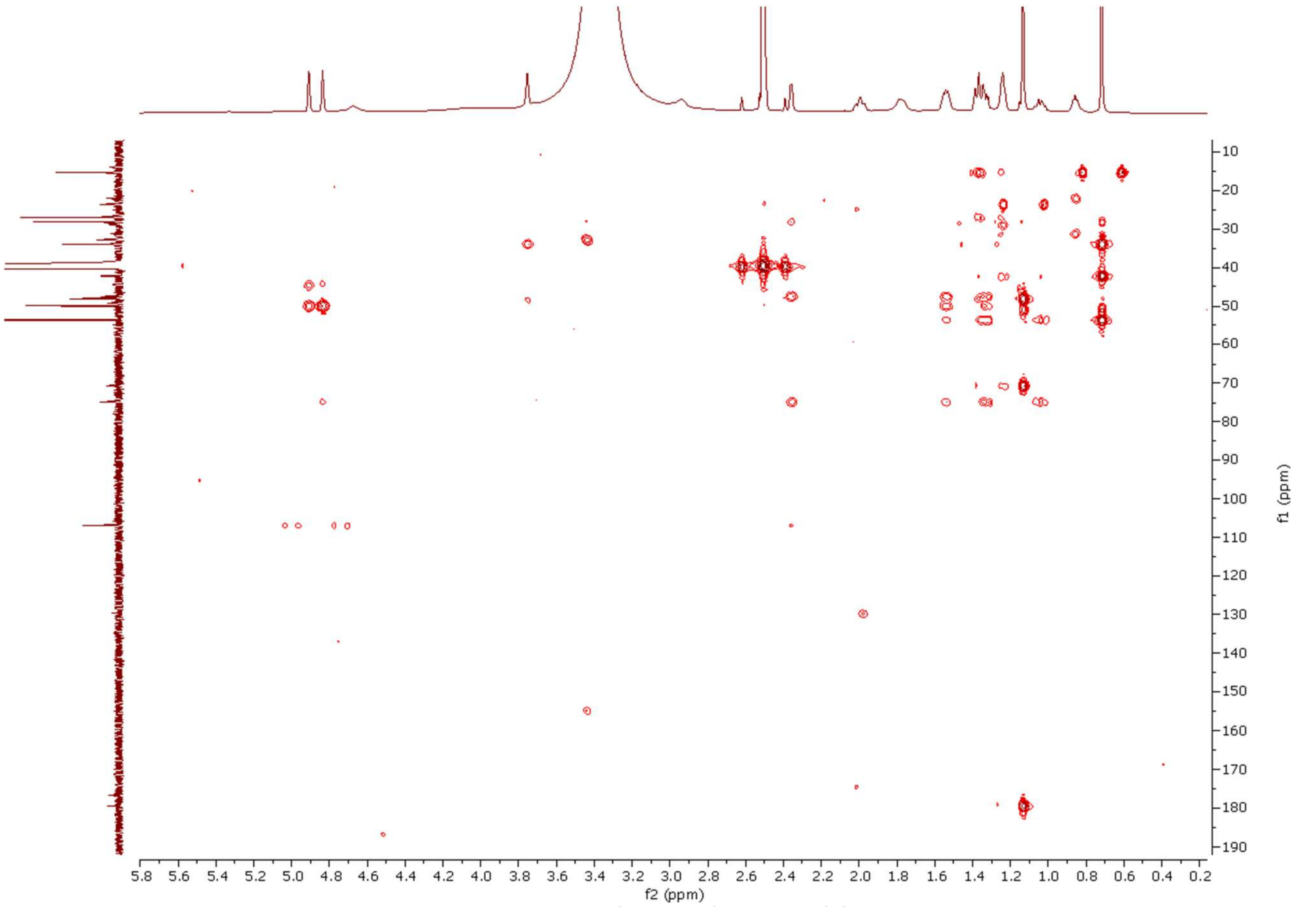

Figure N11.F HMBC NMR of **11** in DMSO-d6.

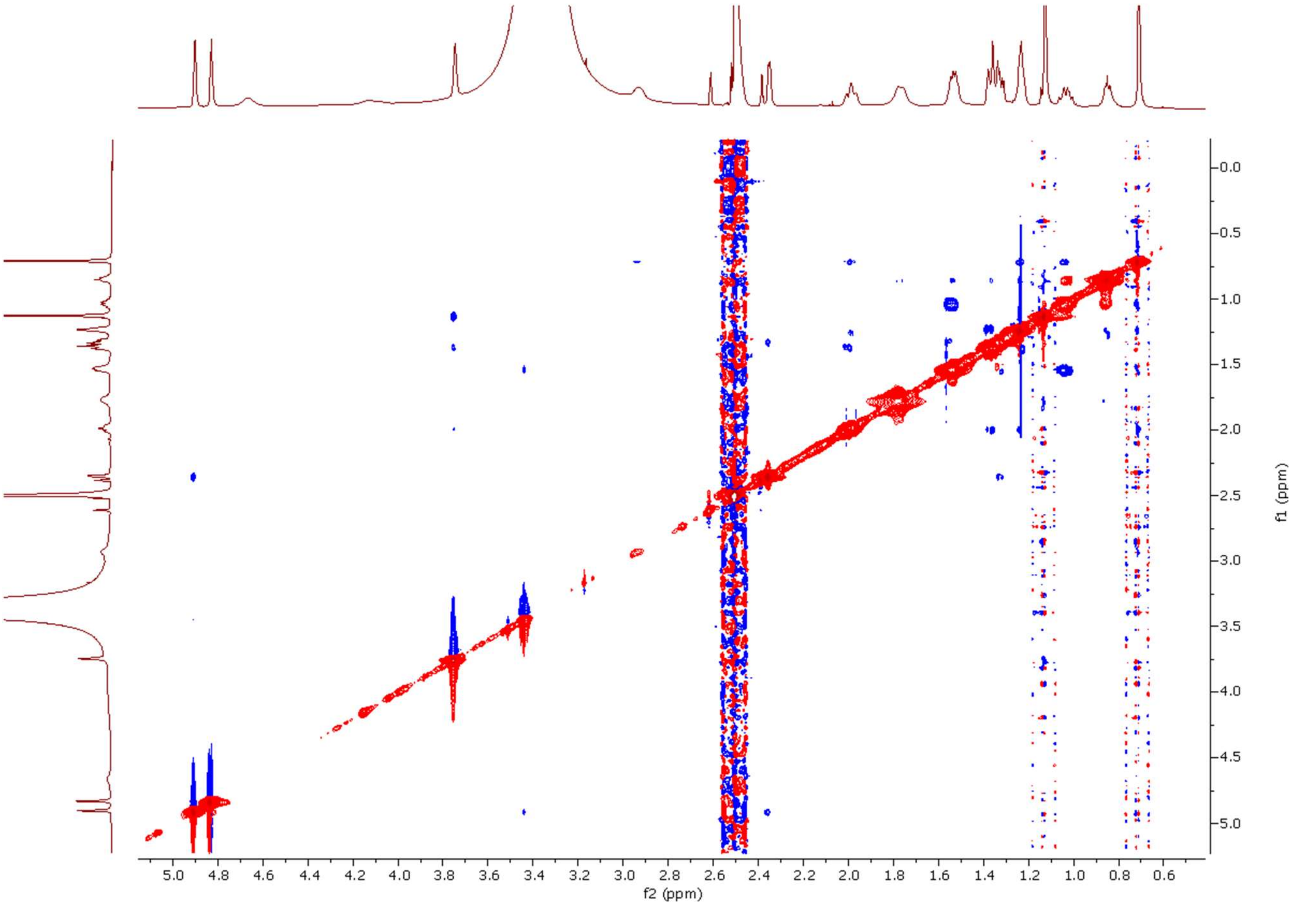

Figure N11.G NOESY NMR of **11** in DMSO-d_6 at 600 MHz.

Gibberellin A_{74} dimethyl ester (11a)

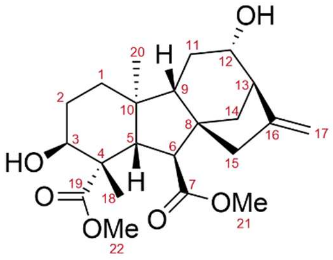

GA$_{74}$ dimethyl ester (**11a**)
Chemical Formula: $C_{22}H_{32}O_6$
Exact Mass: 392.2199

Measured in $CDCl_3$, 298K			
**Pos.**	δ_C 151 MHz	δ_H (*J/Hz*) 600 MHz	**Selected NOESY correlations to H** [a]
**1**	33.8	a 1.44, overlapped<br>b 1.47, overlapped	
**2**	27.1	a 1.67, m<br>b 2.07, m	H-2b, H-3<br>H-2a, H-3
**3**	71.4	eq 4.15, br s	H_3-18, H-2a, H-2b
**4**	48.6	-	-
**5**	49.5	2.33, d (12.9)	H_3-18
**6**	50.9	3.31, d (12.9)	H_3-20
**7**	175.1	-	-
**8**	49.4	-	-
**9**	53.5	1.24, overlapped	H-15b, H-12
**10**	43.8	-	-
**11**	28.4	a 1.24, overlapped<br>b 1.85, overlapped	
**12**	75.4	3.76, dd (7.5, 7.5)	H-11b, H-13, H-17a, H-9
**13**	50.4	2.56, br d (4.9)	H-17a, H-12, H-14a, H-14b
**14**	33.9	a 1.59, dd (11.5, 4.9)<br>b 1.83, overlapped	H-13, H-14b<br>H-13, H-14a
**15**	46.7	a 1.87, overlapped<br>b 2.13, br d (15.7)	H-17b, H-15b<br>H-9, H-15a, H-17b
**16**	152.0	-	-
**17**	108.6	b 4.94, br s<br>a 5.01, br s	H-15a, H-15b, H-17a<br>H-12, H-13, H-17b
**18**	24.4	1.21, s	H-3, H-5
**19**	177.5	-	-
**20**	14.9	0.70, s	H-6, H-2b, H-11a
**21**	51.4	3.69, s	
**22**	51.6	3.69, s	

[a] Key NOESY correlations are shown in blue text.

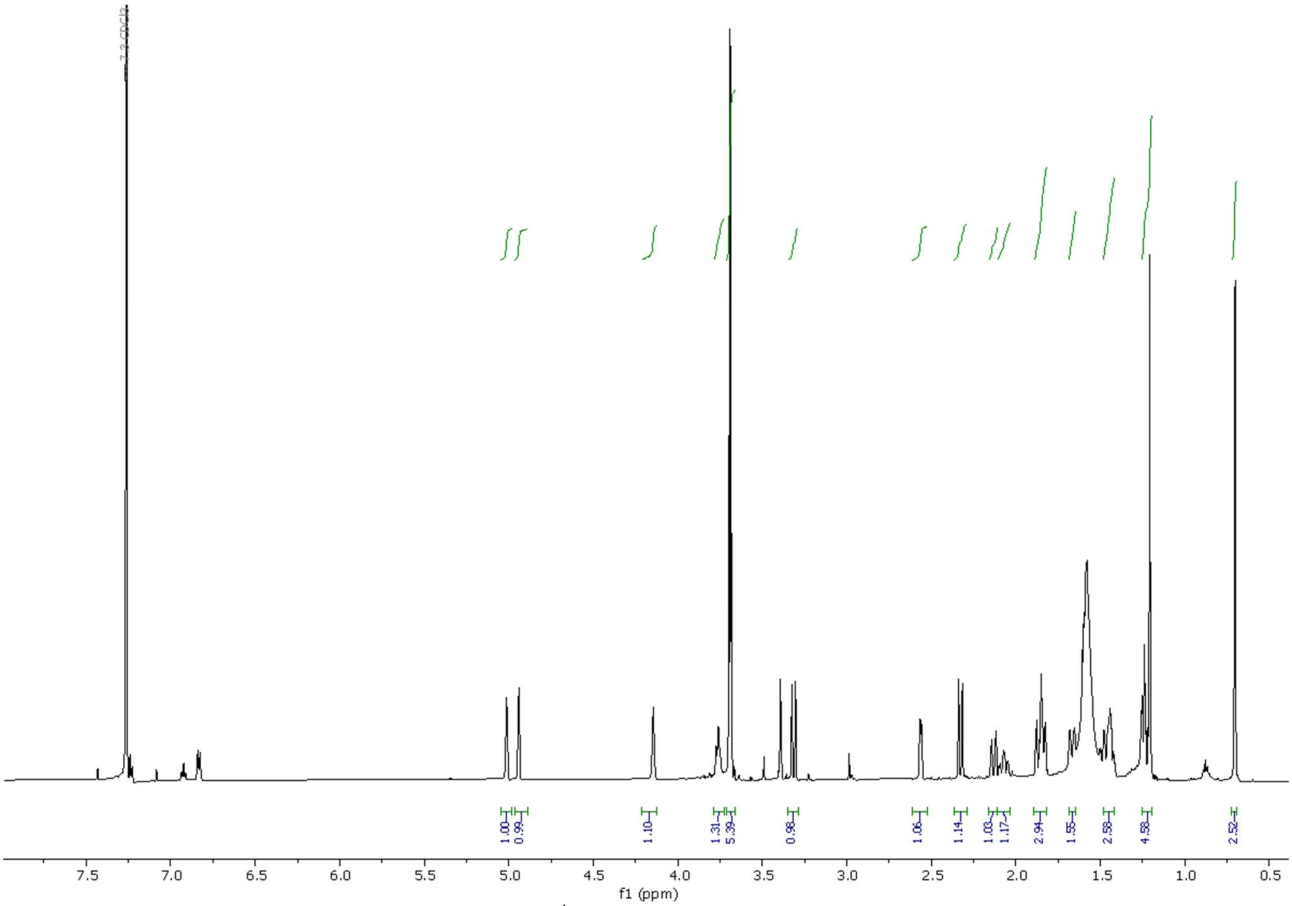

Figure N11a.A ^{1}H NMR of **11a** in $CDCl_3$ at 600 MHz.

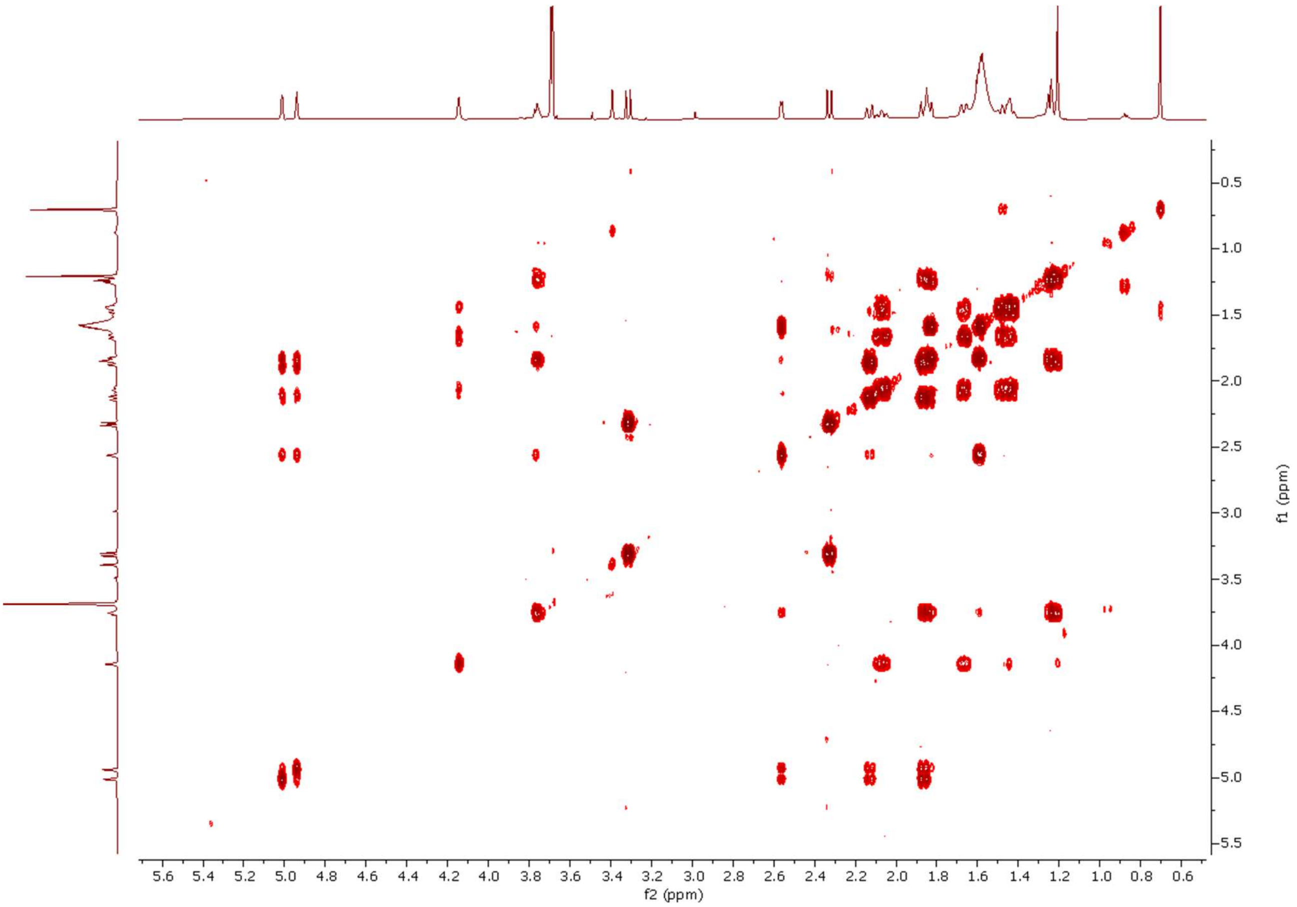

Figure N11a.B COSY NMR of **11a** in $CDCl_3$ at 600 MHz.

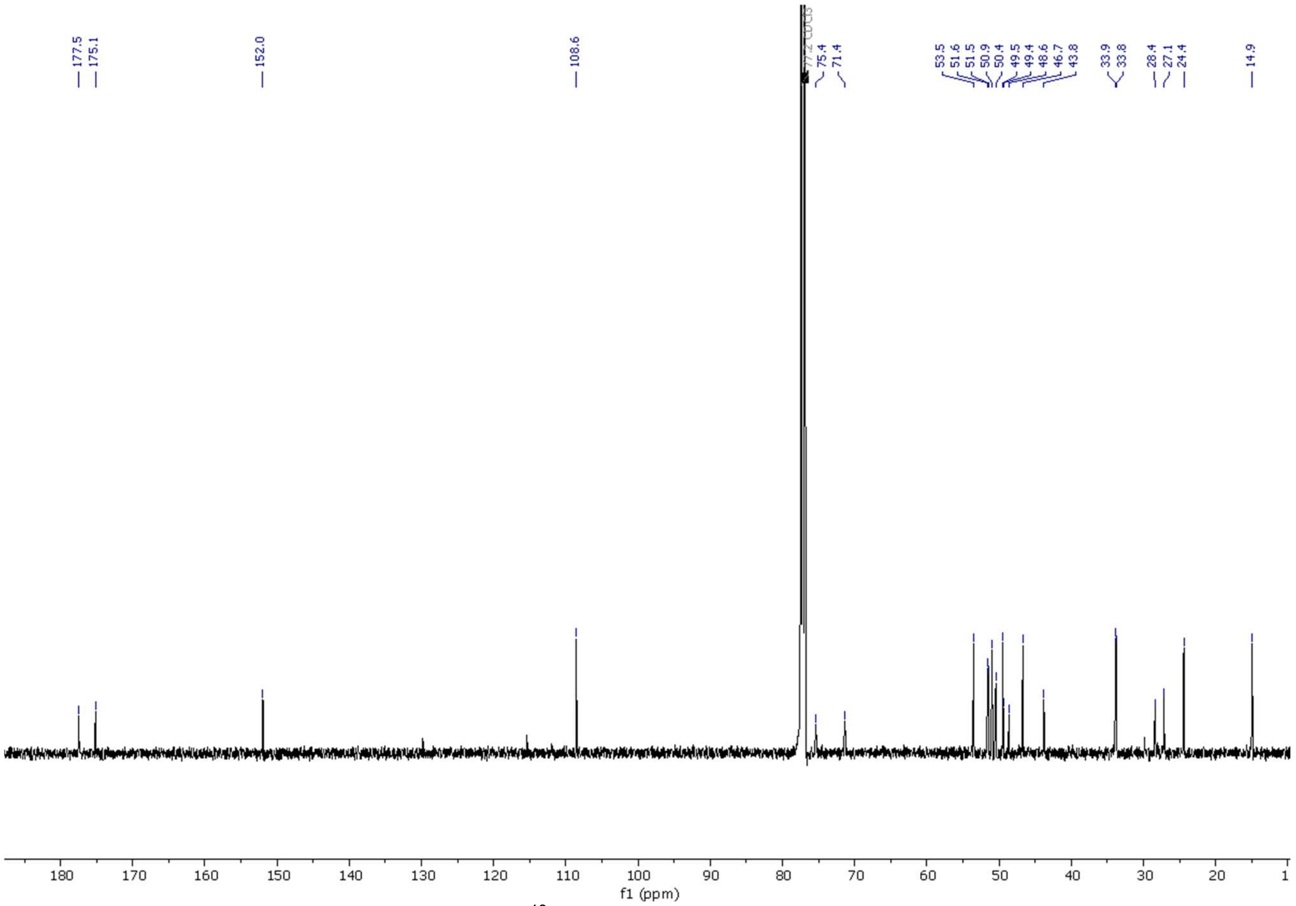

Figure N11a.C ^{13}C NMR of **11a** in $CDCl_3$ at 151 MHz.

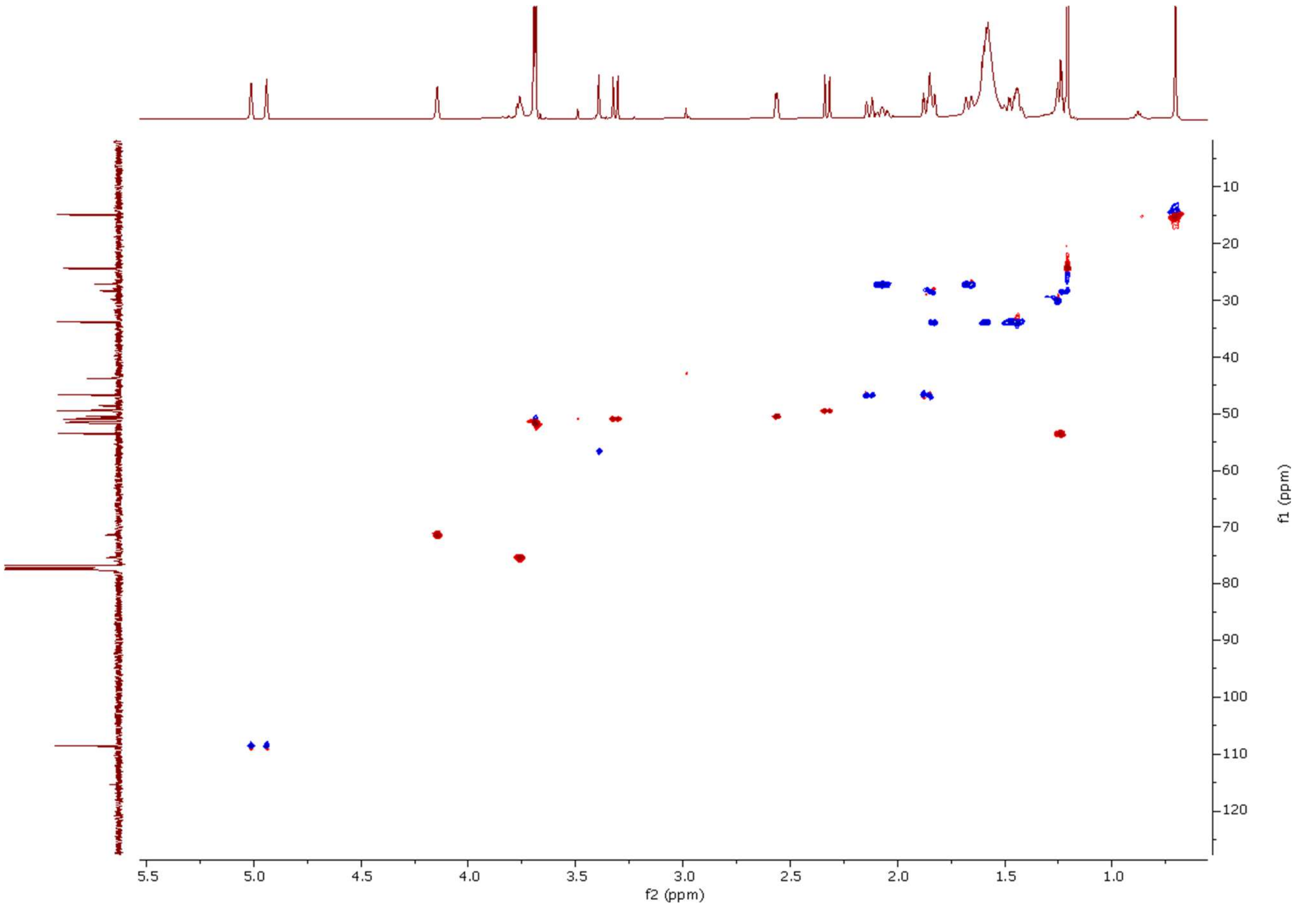

Figure N11a.D HSQC NMR of **11a** in $CDCl_3$.

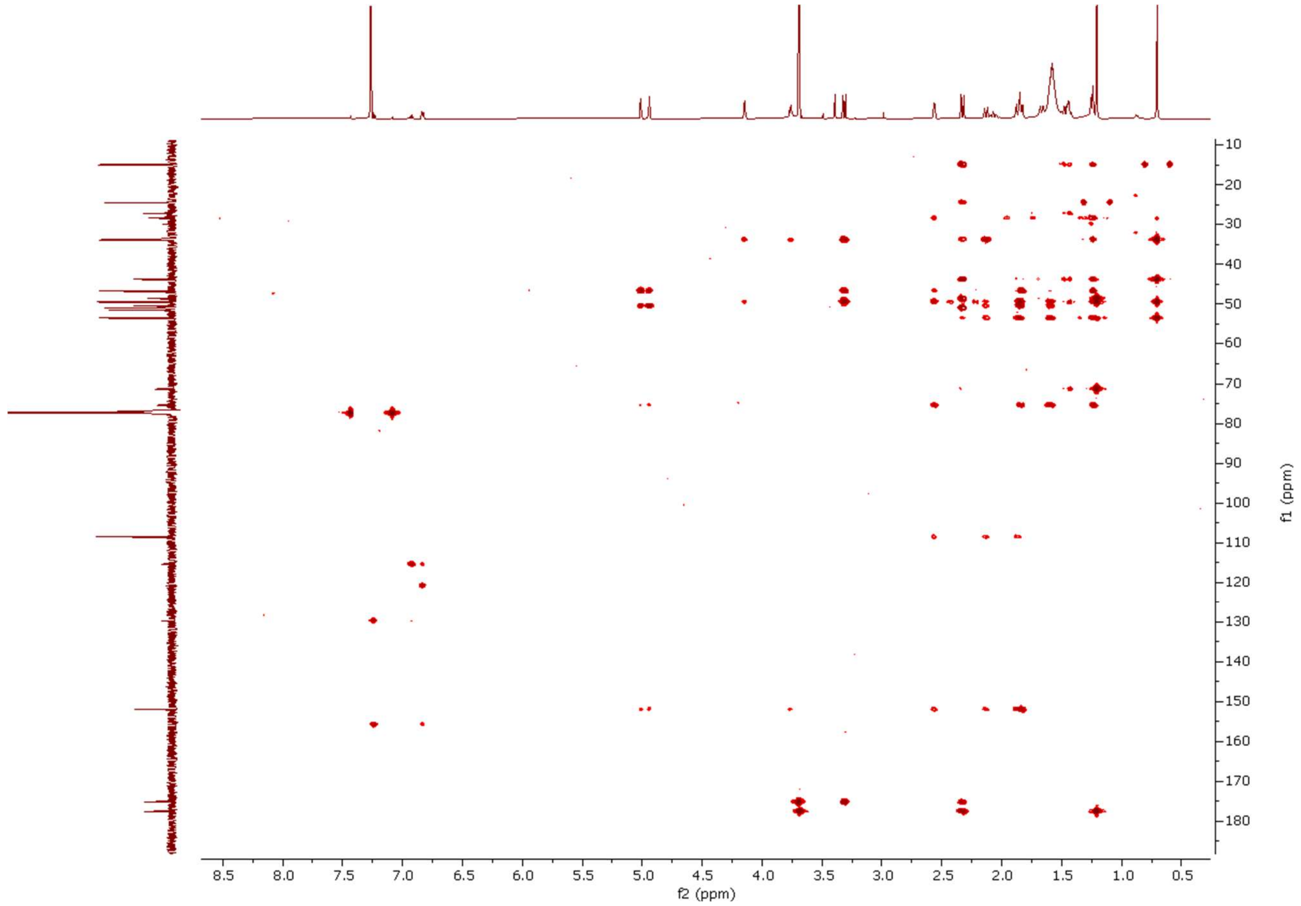

Figure N11a.E HMBC NMR of **11a** in $CDCl_3$.

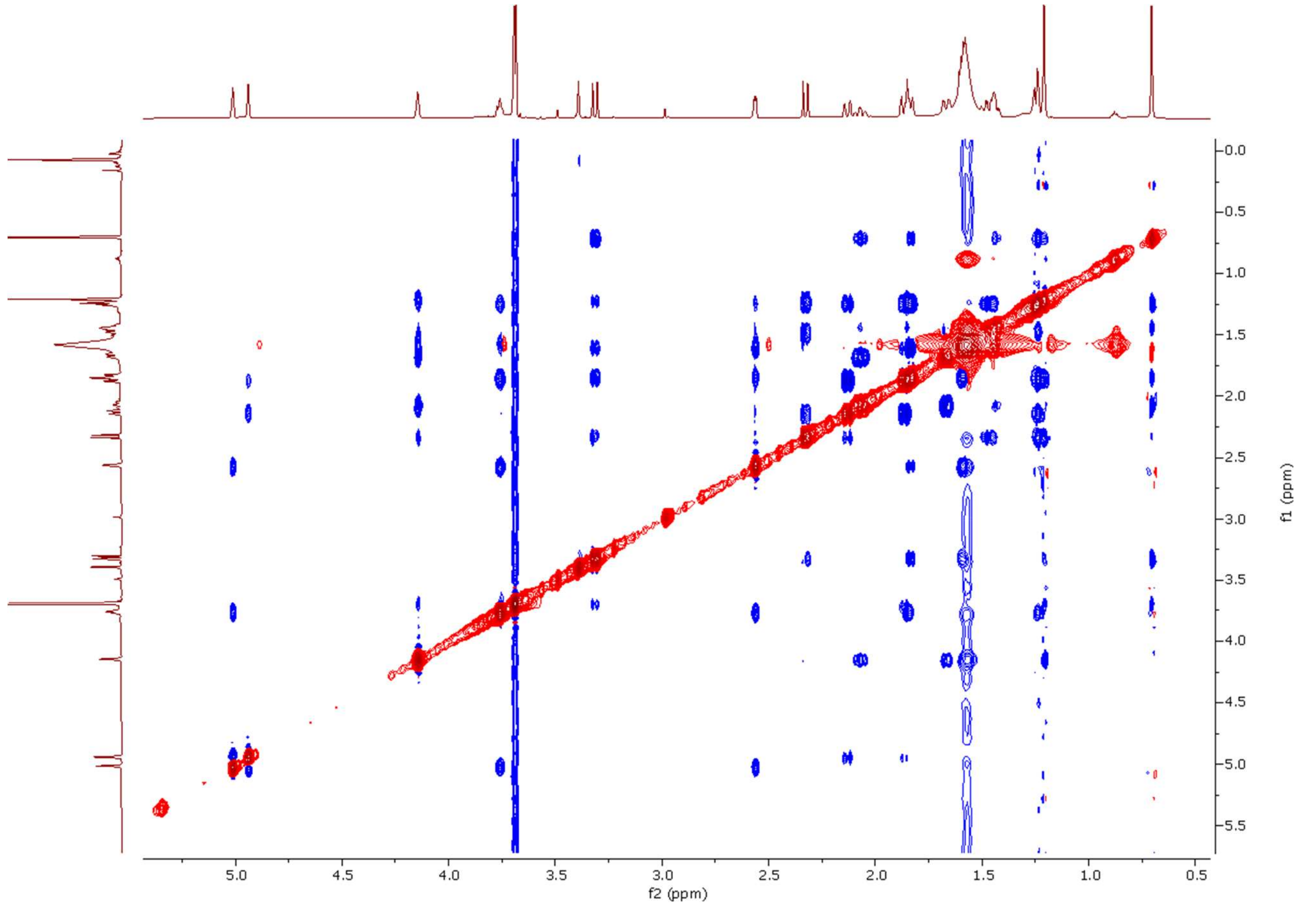

Figure N11a.F NOESY NMR of **11a** in $CDCl_3$ at 600 MHz.

15β-Hydroxy-Gibberellin A_{12} dimethyl ester (12a)

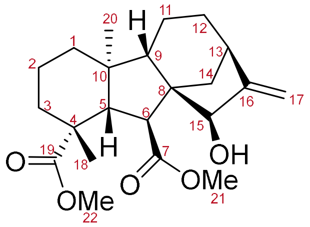

15β-Hydroxy-GA_{12} dimethyl ester (**12a**)
Chemical Formula: $C_{22}H_{32}O_5$
Exact Mass: 376.2250

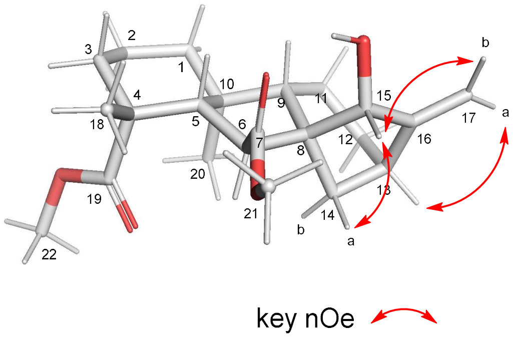

	Measured in d_4-methanol, 298K		
**Pos.**	δ_C 151 MHz	δ_H *(J/Hz)* 600 MHz	**Selected NOESY correlations to H** [a]
**1**	40.5	ax 1.18, ddd (13.2, 13.2, 4.1)<br>eq 1.69, ddd (13.2, 3.5, 3.5)	H-1eq, H-5<br>H-1ax
**2**	20.8	a 1.49, overlapped<br>b 1.79, m	H-2b<br>H-2a
**3**	38.9	a 1.09, overlapped<br>b 2.17, m	H-3b<br>H_3-18, H-3a
**4**	45.7	-	-
**5**	58.6	1.93, overlapped	H_3-18, H-1ax
**6**	52.5	3.33, overlapped	H_3-20
**7**	178.5	-	-
**8**	53.5	-	-
**9**	46.9	1.93, overlapped	
**10**	44.6	-	-
**11**	17.7	1.44, overlapped	
**12**	33.6	a 1.42, overlapped<br>b 1.94, overlapped	H-13, H-17a<br>H-13
**13**	38.9	2.56, br dd (6.0, 6.0)	H-17a, H-14a, H-14b, H-12b, H-12a
**14**	35.1	a 1.49, overlapped<br>b 1.60, d (11.8)	H-13, H-15<br>H-13, H_3-20
**15**	79.2	3.89, dd (2.7, 2.5)	H-14a, H-17b
**16**	159.3	-	-
**17**	107.9	a 4.99, ddd (2.7, 1.5, 1.5)<br>b 5.05, br s	H-13, H-12a<br>H-15
**18**	29.5	1.07, s	H-3b, H-5
**19**	179.1	-	-
**20**	16.0	0.71, s	H-14b, H-6, H_3-22, H-2b
**21**	52.3	3.73, s	H_3-18
**22**	51.8	3.65, s	H_3-20

[a] Key NOESY correlations are shown in blue text.

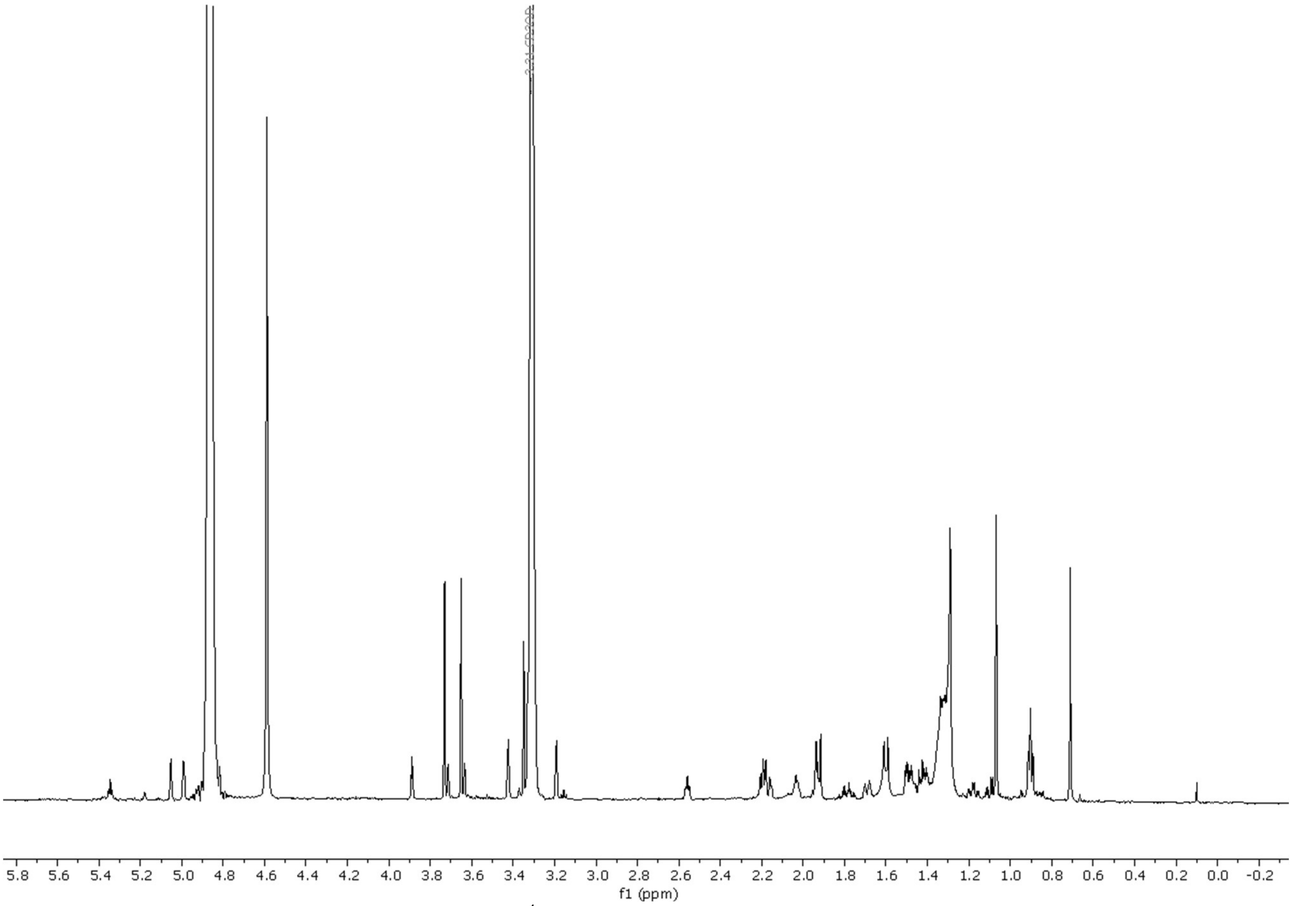

Figure N12a.A ^{1}H NMR of **12a** in d4-methanol at 600 MHz.

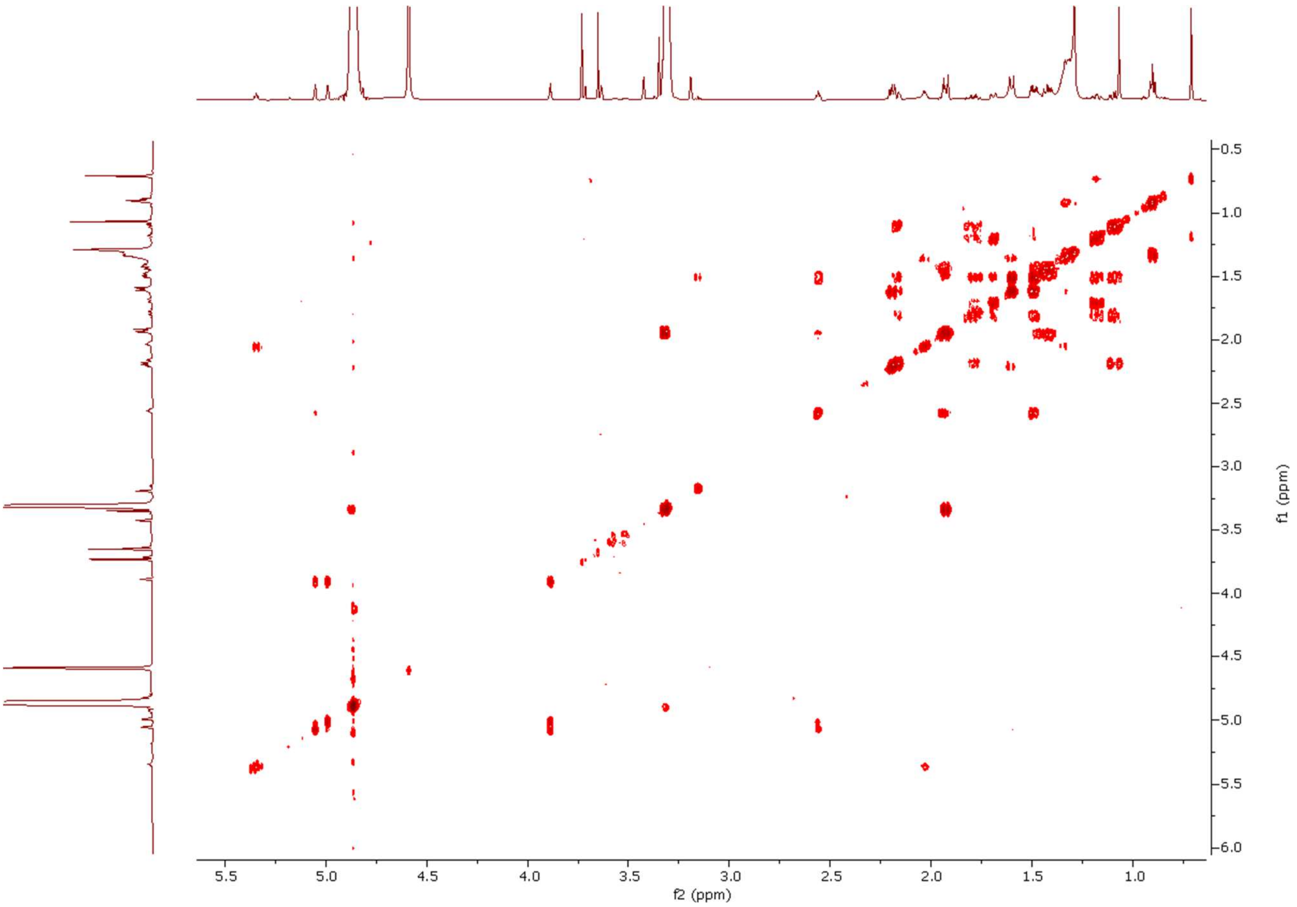

Figure N12a.B COSY NMR of **12a** in d4-methanol at 600 MHz.

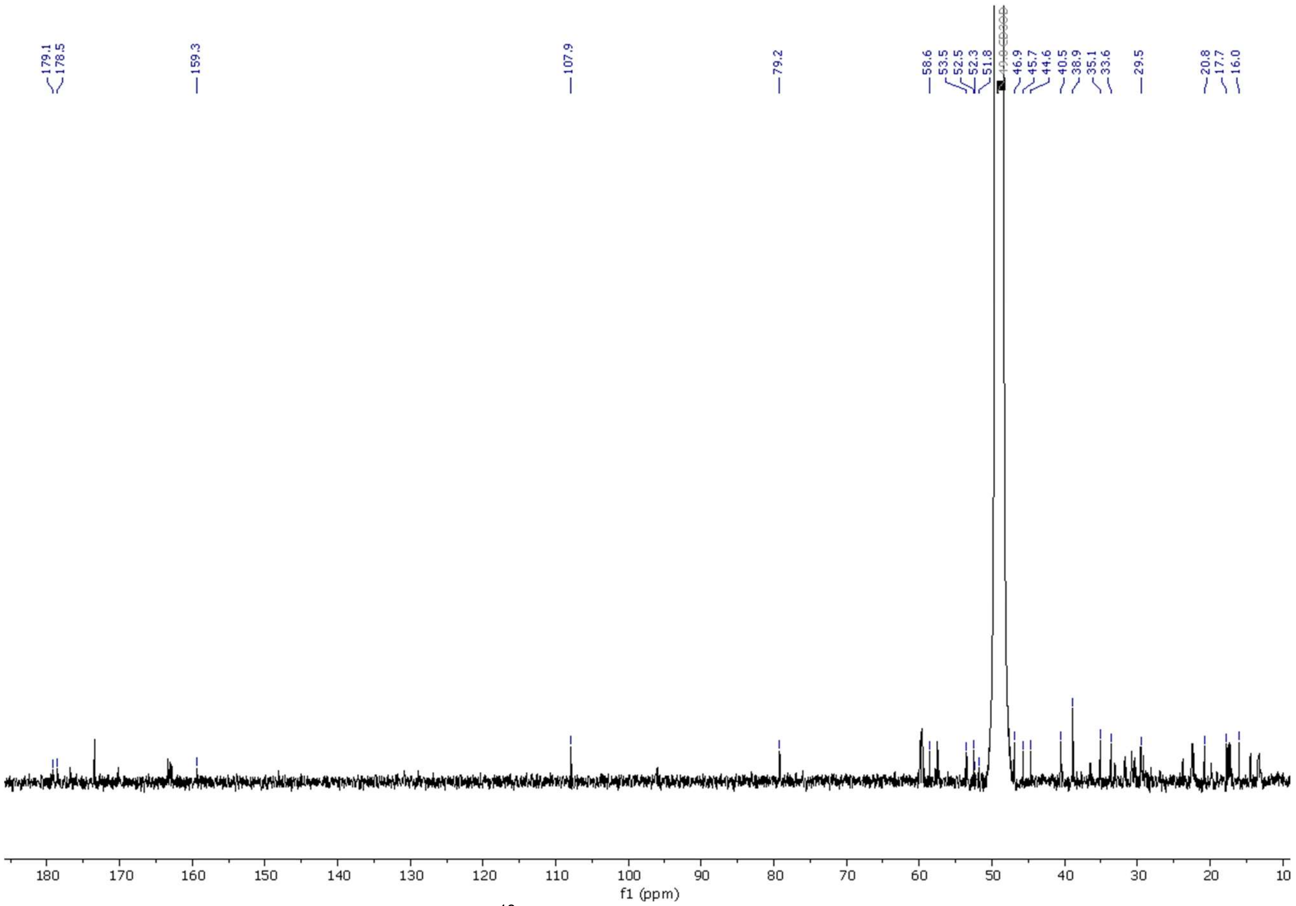

Figure N12a.C ^{13}C NMR of **12a** in d_4-methanol at 151 MHz.

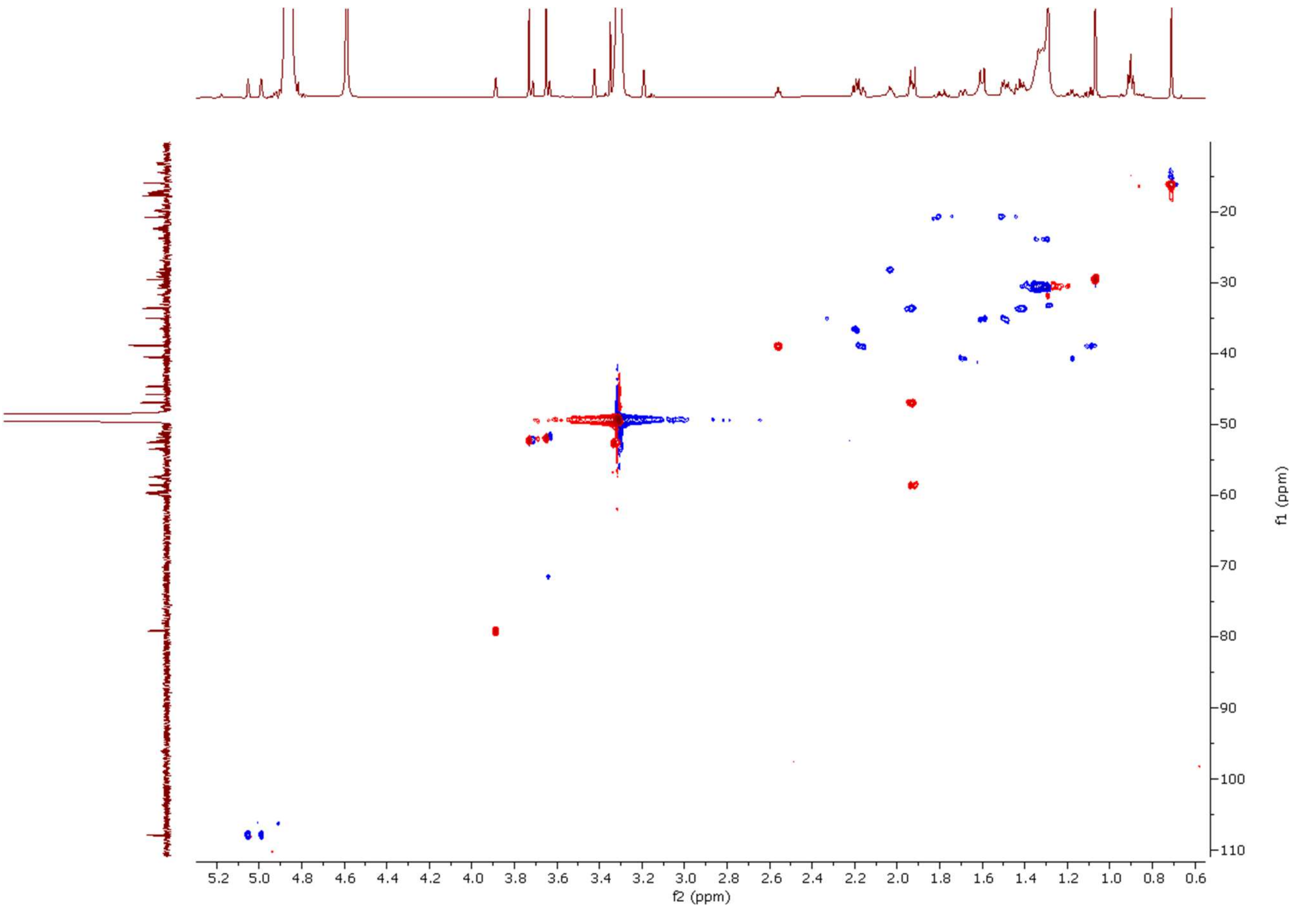

Figure N12a.D HSQC NMR of **12a** in d_4-methanol

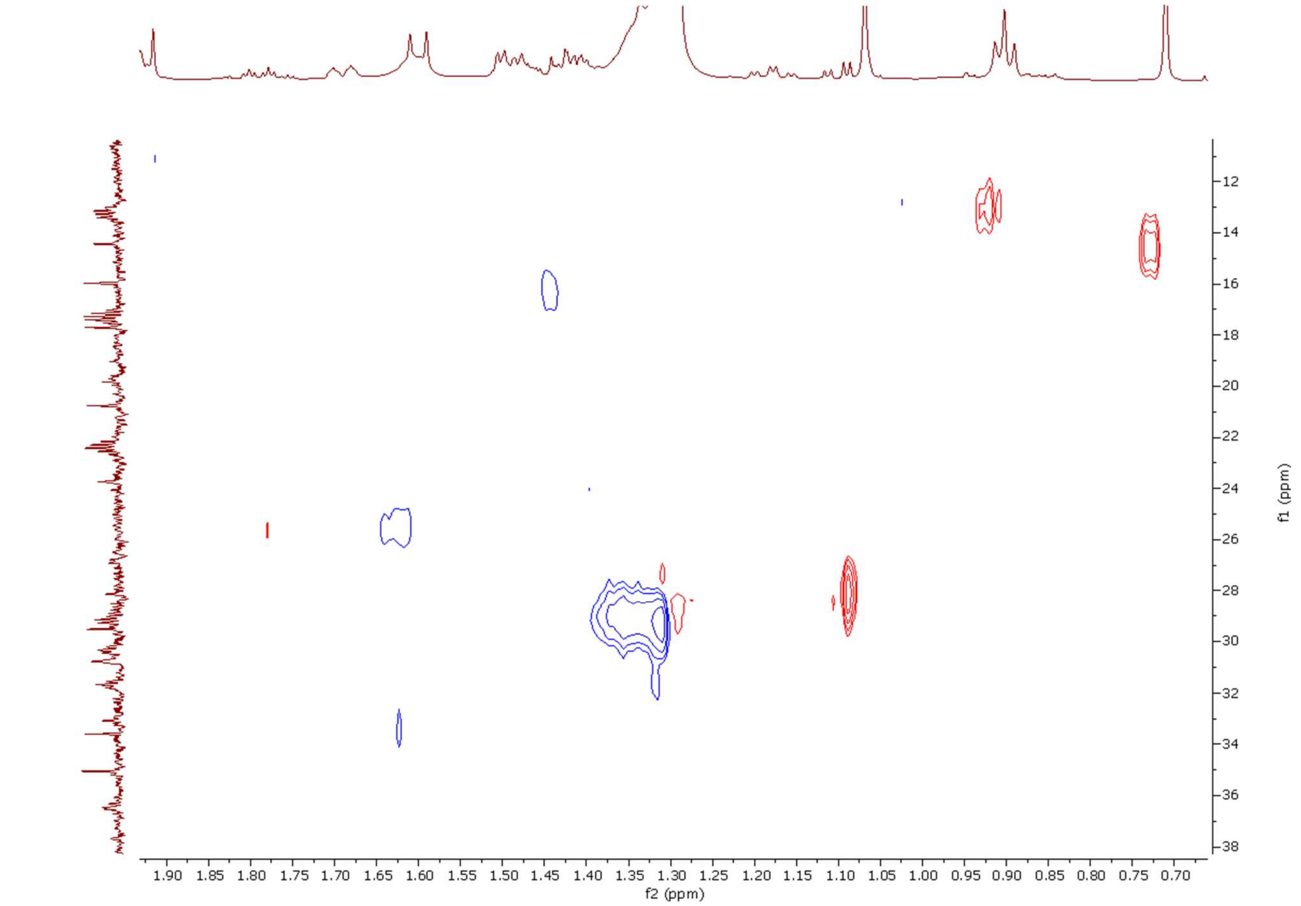

Figure N12a.E HSQC (zoomed in) NMR of **12a** in d_4-methanol.

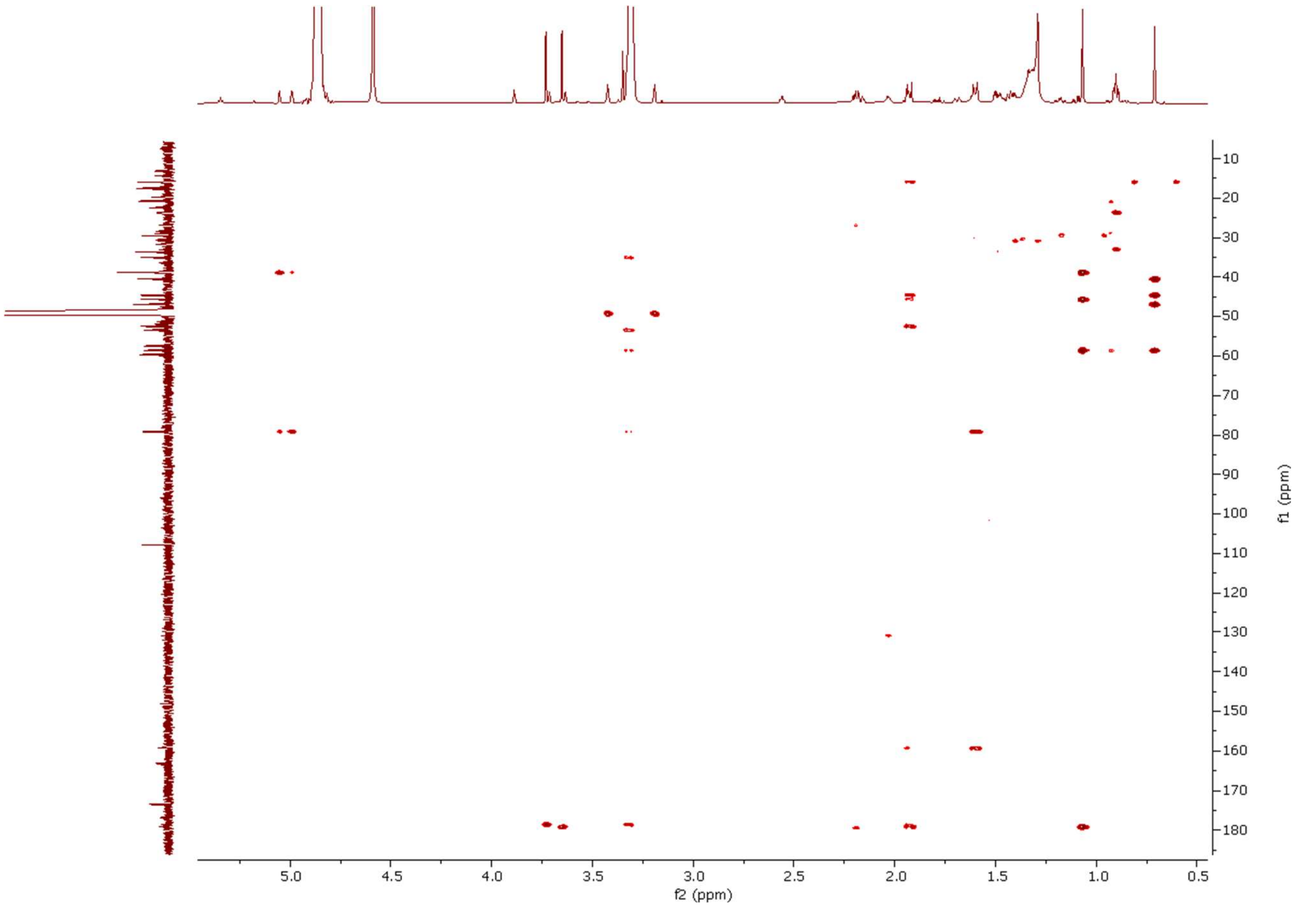

Figure N12a.F HMBC NMR of **12a** in d_4-methanol.

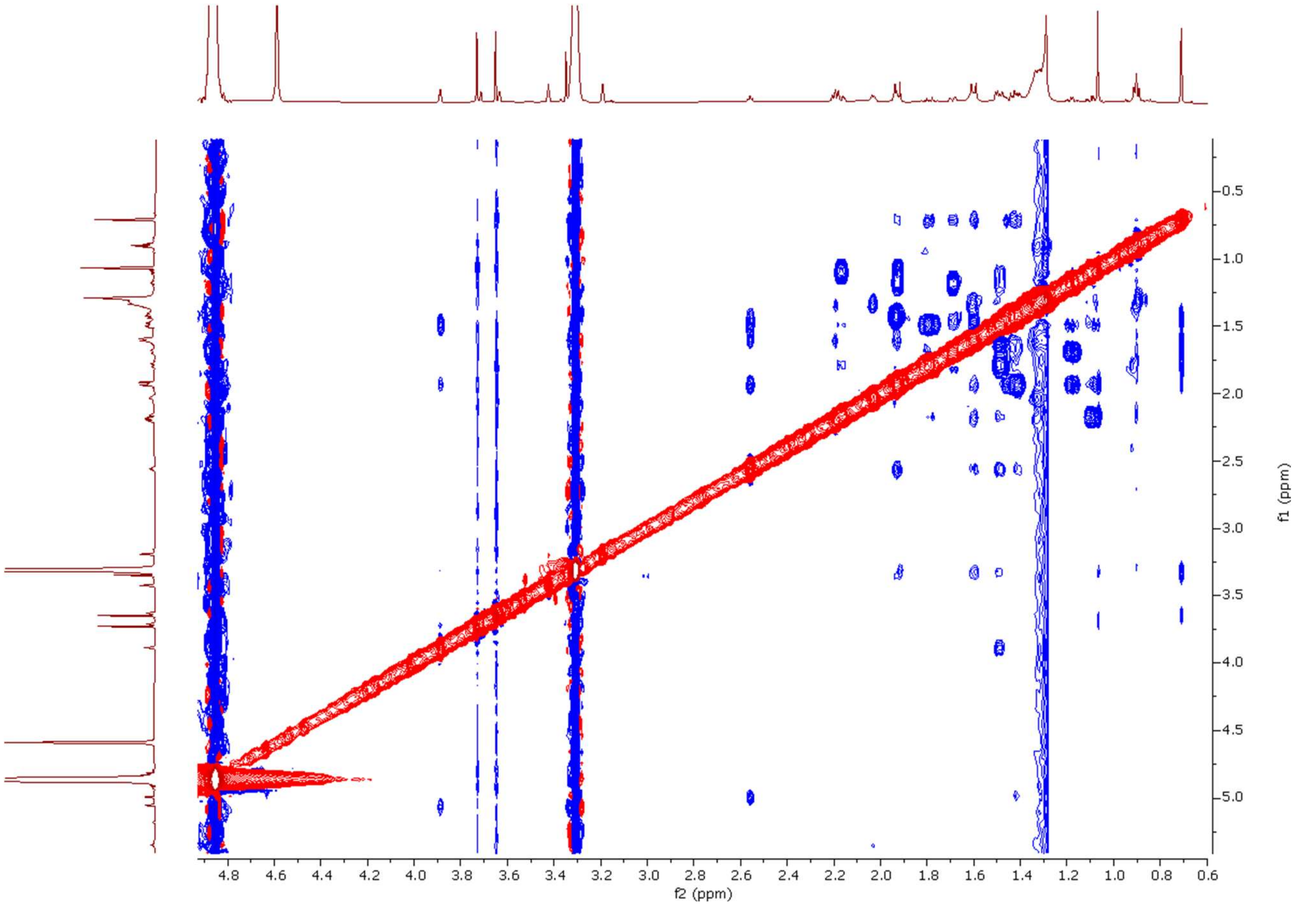

Figure N12a.G NOESY NMR of **12a** in d_4-methanol at 600 MHz.

ent-Labd-8(17)-en-15,18-dioic acid (17)

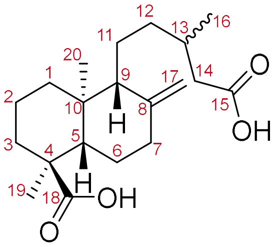

ent-Labd-8(17)-en-15,18-dioic acid (**17**)

Chemical Formula: $C_{20}H_{32}O_4$

Exact Mass: 336,23

	Reference [43]		Measured in DMSO-d$_6$, 298K		
**Pos.**	δ_C 151 MHz in $CDCl_3$	δ_H (*J/Hz*) 600 MHz in CD_3OD	δ_C 101 MHz	δ_H (*J/Hz*) 400 MHz	**Selected NOESY correlations to H** [a]
**1**	38.1	1.12 − 1.19, m<br>1.48 − 1.54, m	37.7	ax 1.05, overlapped<br>eq 1.75, br d (13.3)	H-1eq<br>H-1ax, H_3-20
**2**	18.6	1.36 − 1.42, m<br>1.59 − 1.64, m	18.1	a 1.52, overlapped<br>b 1.52, overlapped	
**3**	37.2	1.54 − 1.59, m<br>1.79, td (12.7. 4.7)	36.6	a 1.47, overlapped<br>b 1.66, m	
**4**	47.7	-	46.5	-	-
**5**	49.6	1.96, dd (12.5, 2.5)	49.2	1.86, dd (12.3, 2.9)	H-9
**6**	27.0	1.30 – 136, m<br>1.45, qd (13.0, 4.3)	26.3	a 1.24, overlapped<br>b 1.35, overlapped	
**7**	38.0	2.02, ddd (12.8, 4.2, 2.3)<br>2.35, td (13.0, 5.0)	37.4	ax 1.91, overlapped<br>eq 2.30, ddd (12.7, 4.3, 2.3)	H-7eq<br>H-7ax, H-17b
**8**	148.1	-	147.8	-	-
**9**	56.9	1.64 − 1.68, m	56.7	1.57, br d (10.8)	H-5
**10**	39.0	-	38.6	-	-
**11**	20.8	1.33 − 1.36, m<br>1.58 − 1.64, m	20.3	a 1.25, overlapped<br>b 1.45, overlapped	
**12**	35.6	1.12 − 1.19, m<br>1.83, br d (12.8)	35.3	a 0.93, m<br>b 1.41, overlapped	
**13**	30.8	1.85 − 1.92, m	30.4	1.79, overlapped	H_3-16
**14**	42.0	2.08, dd (14.8, 7.8)<br>2.23, dd (14.8, 6.4)	41.2	a 1.98, dd (14.9, 7.9)<br>b 2.19, dd (14.9, 6.0)	H-14b, H_3-16<br>H-14a, H-13
**15**	180.1	-	174.0	-	-
**16**	19.8	0.96, d (6.7)	19.9	0.88, d (6.6)	H-14a, H-13
**17**	107.1	4.53, br s<br>4.82, br s	106.9	a 4.49, br s<br>b 4.80, br s	17-b<br>H-7b, 17-a
**18**	185.8	-	179.6	-	-
**19**	16.4	1.12, s	16.5	1.02, s	H_3-20, H-2, H-6b
**20**	14.8	0.73, s	14.4	0.64, s	H-1eq, H-2, H-6b, H_3-19

[a] Key NOESY correlations are shown in blue text.

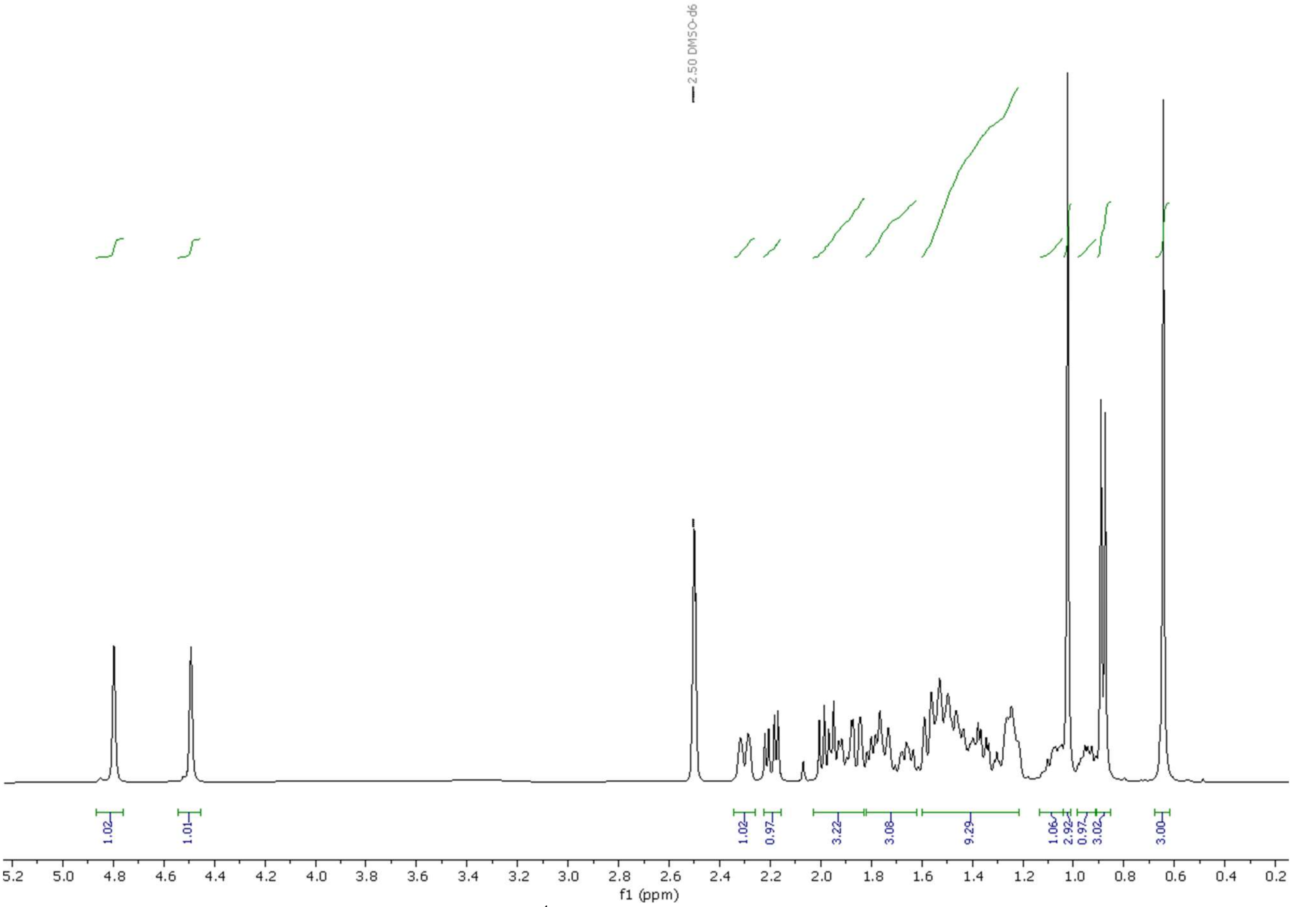

Figure N17.A ^{1}H NMR of **17** in DMSO-d$_6$ at 400 MHz.

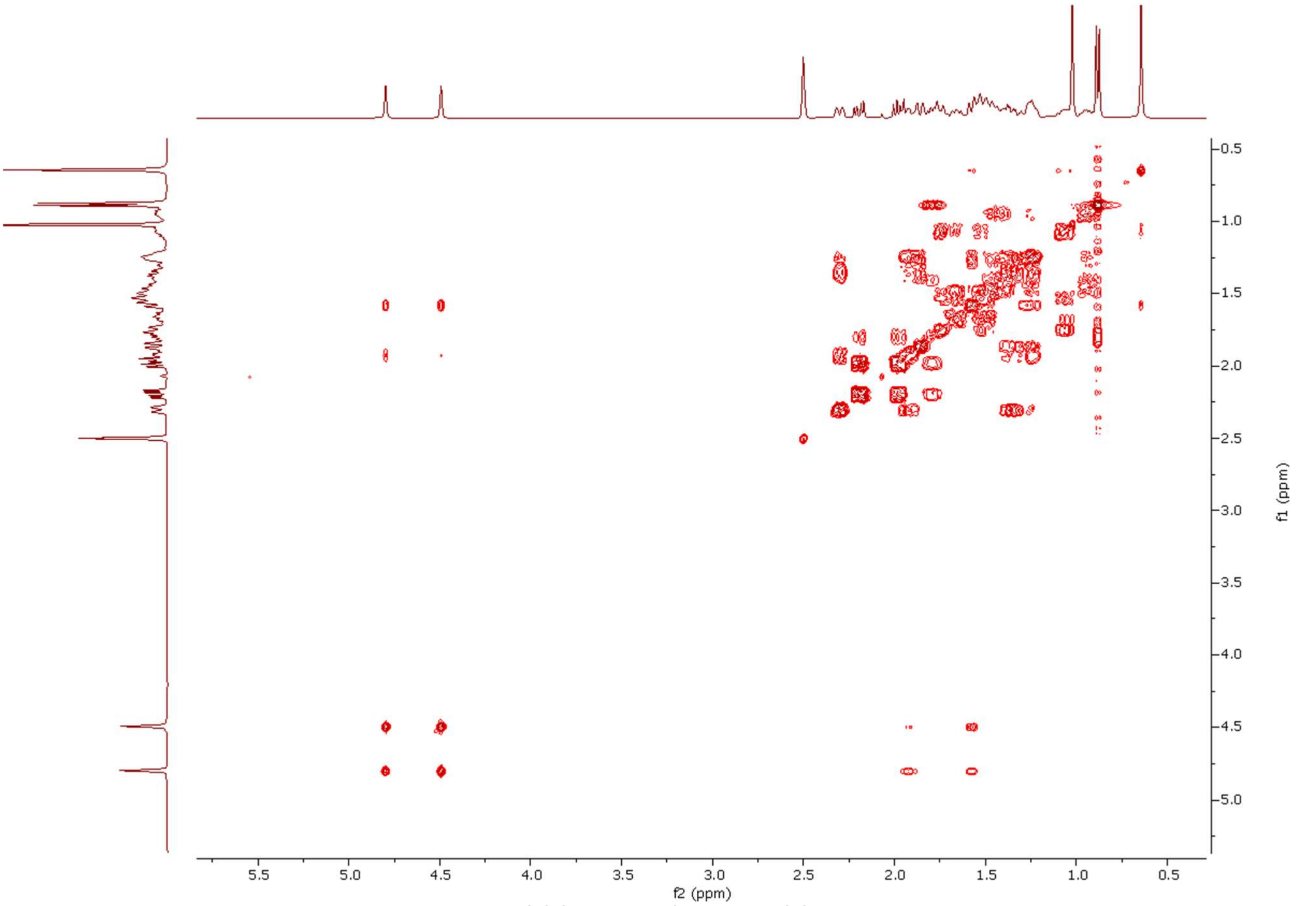

Figure N17.B COSY NMR of **17** in DMSO-d$_6$ at 400 MHz.

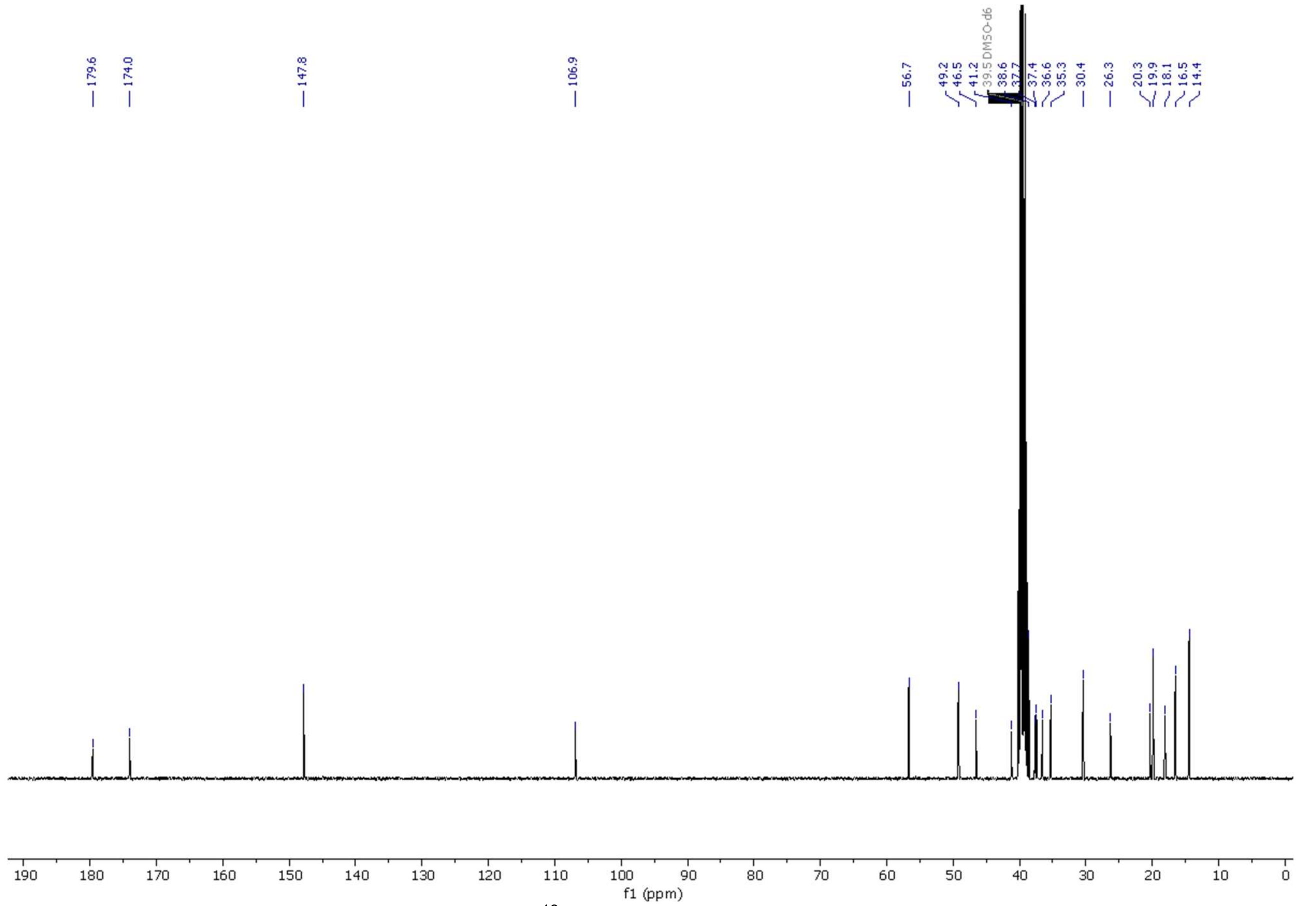

Figure N17.C ^{13}C NMR of **17** in DMSO-d$_6$ at 101 MHz.

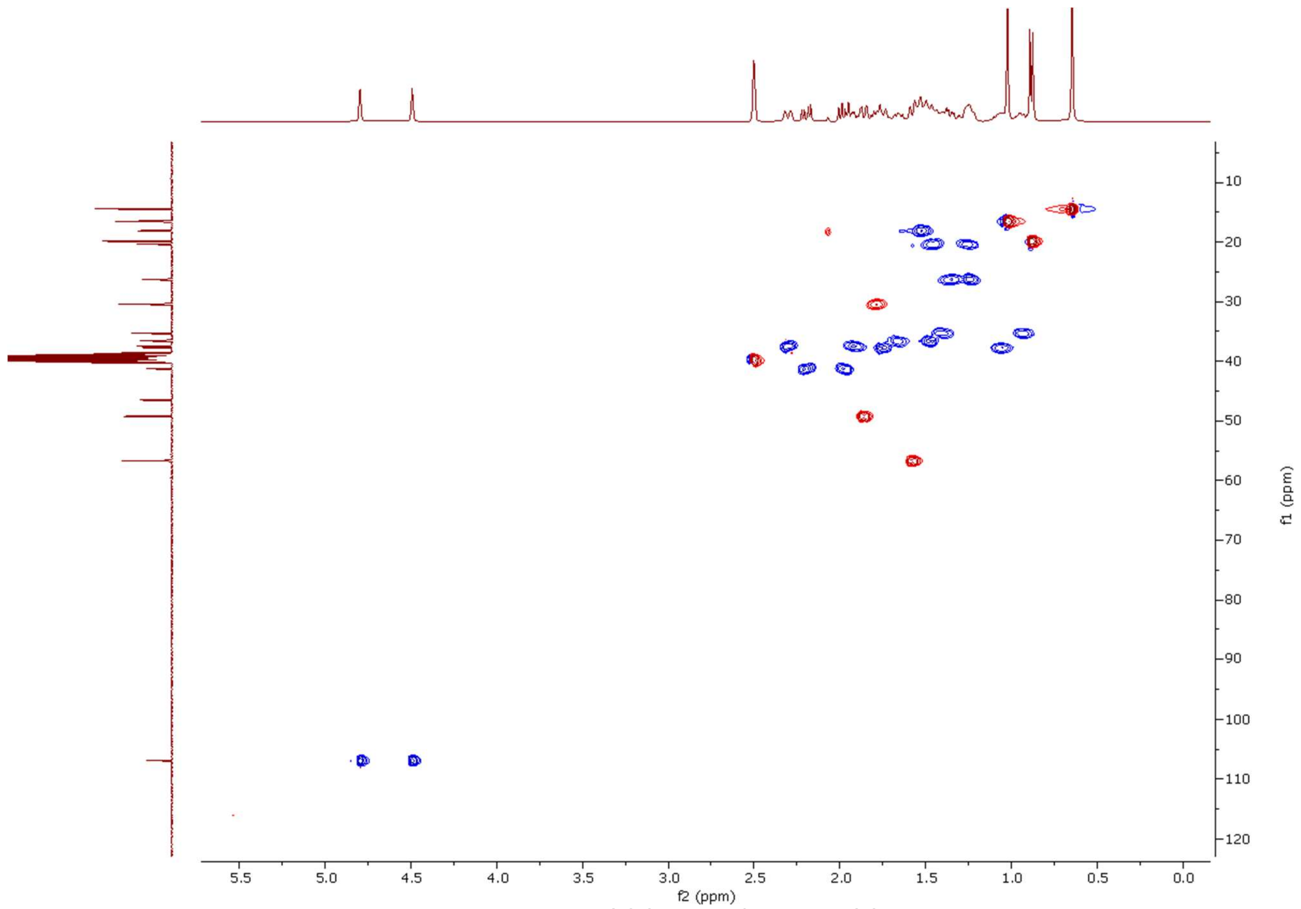

Figure N17.D HSQC NMR of **17** in DMSO-d$_6$.

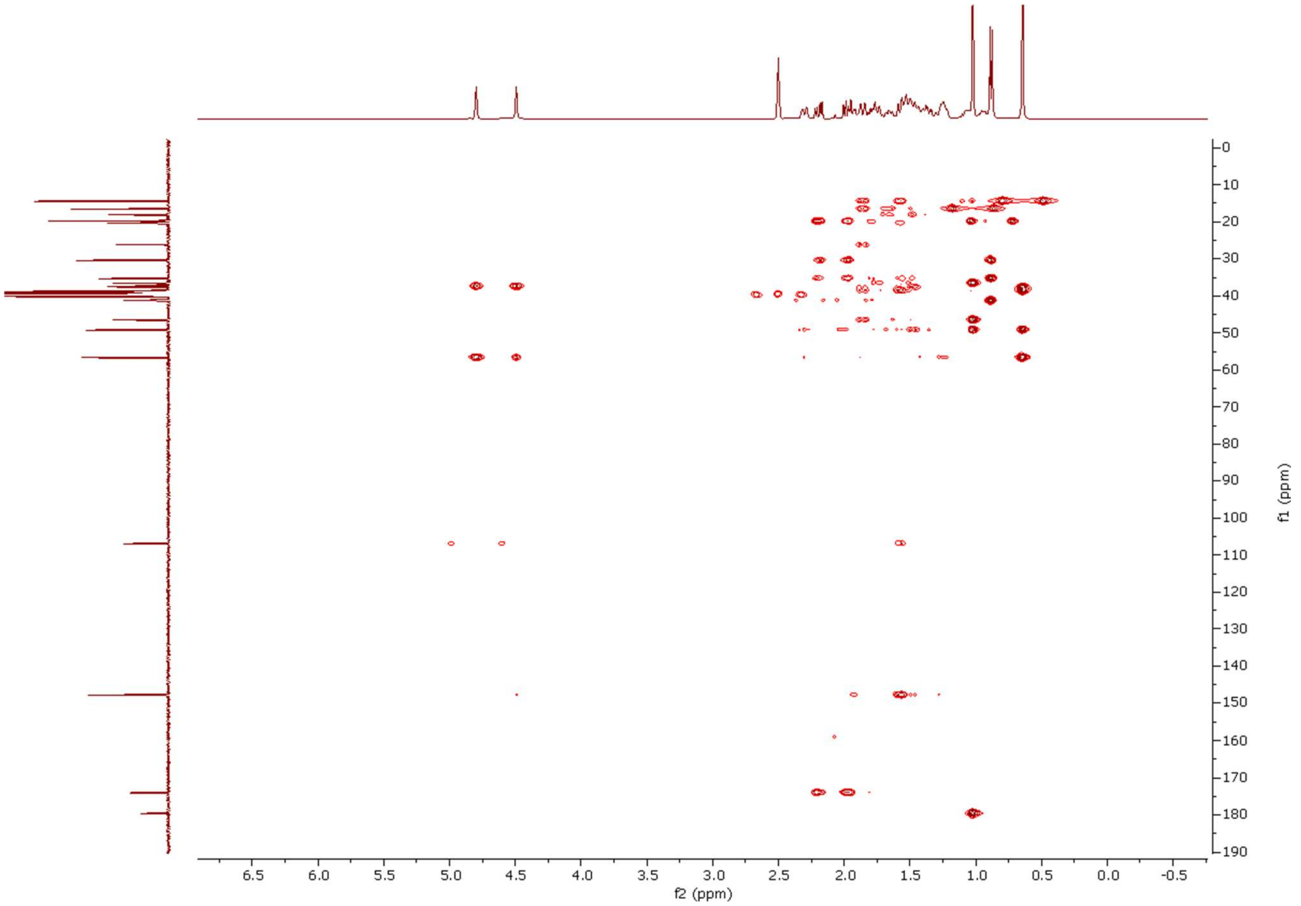

Figure N17.E HMBC NMR of **17** in DMSO-d_6.

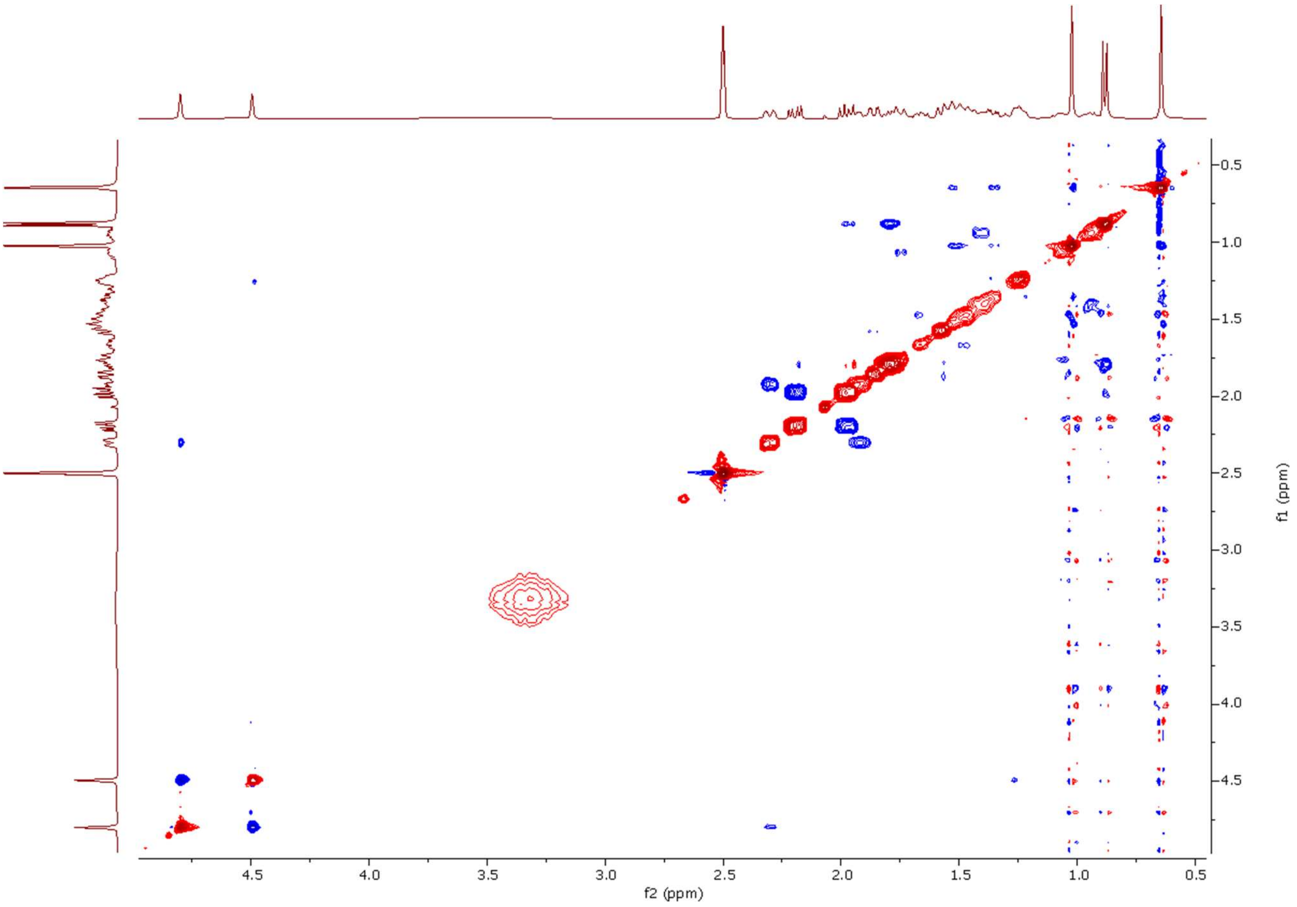

Figure N17.F NOESY NMR of **17** in DMSO-d_6 at 400 MHz.

13,18-dihydroxy-*ent*-labd-8(17)-en-15-oic acid (18)

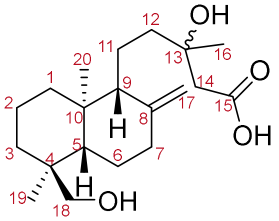

13,18-dihydroxy-*ent*-labd-8(17)-en-15-oic acid (**18**)
Chemical Formula: $C_{20}H_{34}O_4$
Exact Mass: 338.2457

	**Measured in d$_4$-methanol, 298K**		
**Pos.**	*δ$_C$* 126 MHz	*δ$_H$ (J/Hz)* 600 MHz	**Selected NOESY correlations to H [a]**
**1**	39.9	ax 1.07, ddd (13.1, 13.1, 3.5)<br>eq 1.82, ddd (13.1, 3.2, 3.2)	H-1eq, H-2a<br>H-1ax, H_3-20, H-2a
**2**	19.9	a 1.55, m<br>b 1.65, overlapped	 H-1eq
**3**	36.6	eq 1.24, ddd (13.2, 3.1, 3.1)<br>ax 1.51, overlapped	H_3-19
**4**	39.0	-	-
**5**	49.4	1.50, overlapped	
**6**	25.3	ax 1.32, m<br>eq 1.66, overlapped	H_3-20
**7**	39.2	ax 2.03, ddd (13.2, 12.7, 5.0)<br>eq 2.36, ddd (12.7, 4.3, 2.4)	H-6ax, H-6eq, H-7eq<br>H-7ax
**8**	149.8	-	-
**9**	58.9	1.62, br d (11.3)	H-1ax, H-7ax
**10**	40.8	-	-
**11**	18.9	a 1.43, m<br>b 1.65, overlapped	 H_3-20
**12**	42.0	a 1.28, overlapped<br>b 1.79, ddd (13.4, 12.8, 4.4)	
**13**	72.6	-	-
**14**	46.4	a 2.43, d (14.6)<br>b 2.44, d (14.6)	
**15**	176.0	-	-
**16**	27.2	1.27, s	H-14a, H-12b
**17**	107.2	a 4.59, dd (2.6, 1.6)<br>b 4.82, dd (2.8, 1.6)	H-11a, H-12b, H-17b<br>H-7eq, H-17a
**18**	72.0	a 2.99, d (11.1)<br>b 3.35, d (11.1)	H_3-19, H-5, H-6eq, H-3eq, H-18b<br>H_3-19, H-5, H-6eq, H-18a
**19**	18.1	0.73, s	H-6ax, H-3eq, H-18a, H-18b
**20**	15.5	0.76, s	H-11b, H-6ax

[a] Key NOESY correlations are shown in blue text.

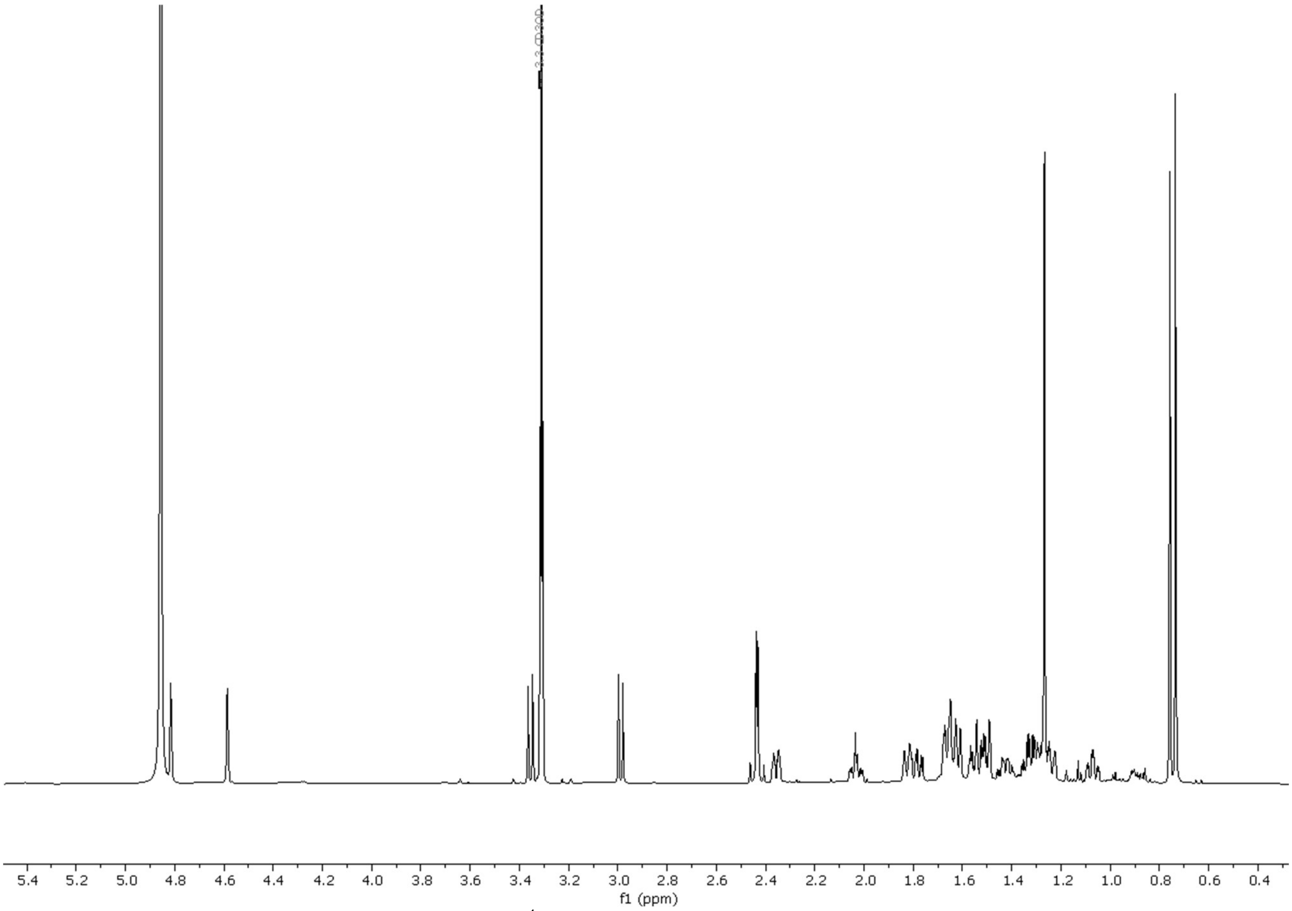

Figure N18.A ^{1}H NMR of **18** in d$_4$-methanol at 600 MHz.

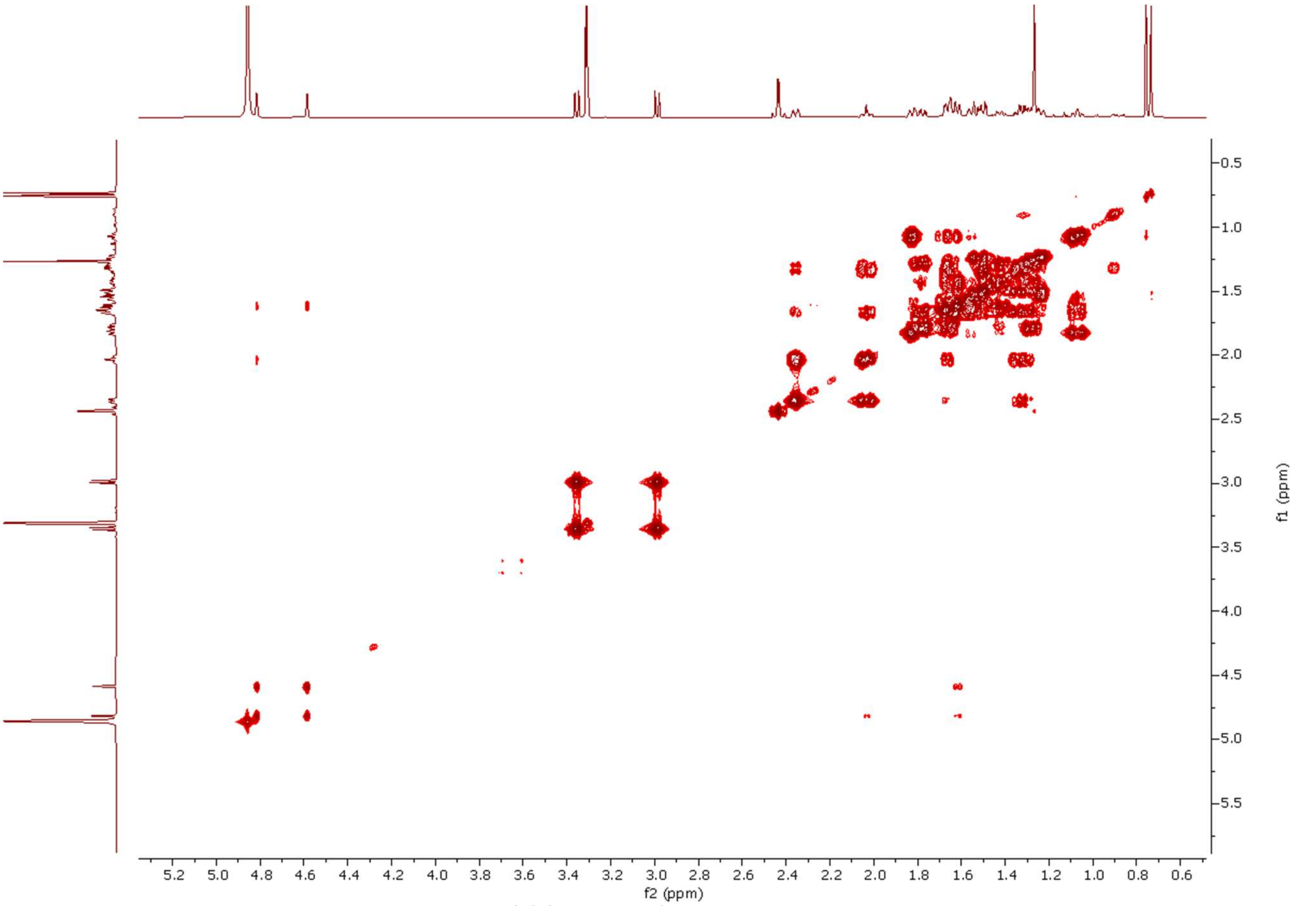

Figure N18.B COSY NMR of **18** in d$_4$-methanol at 500 MHz.

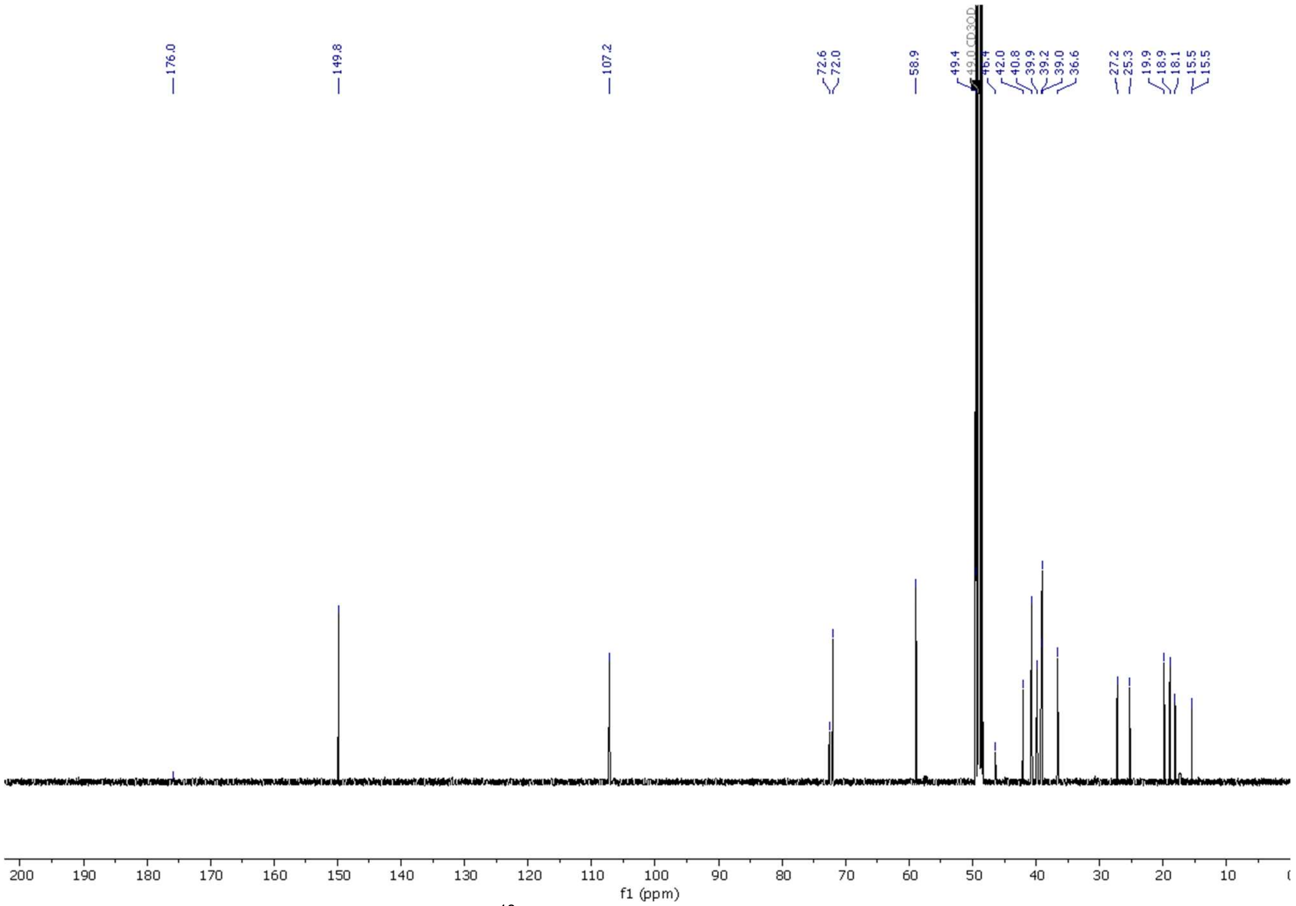

Figure N18.C ^{13}C NMR of **18** in d_4-methanol at 151 MHz.

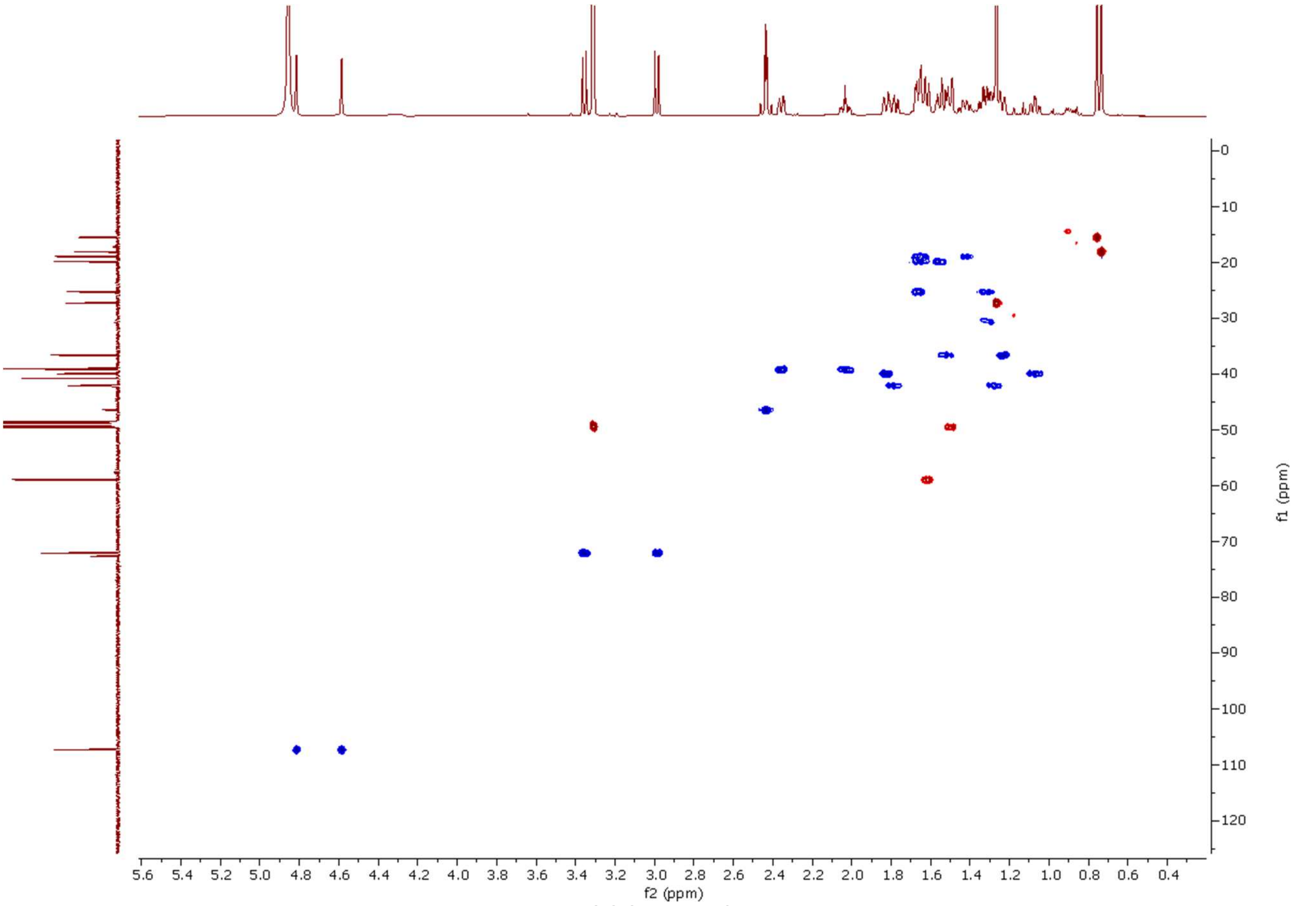

Figure N18.D HSQC NMR of **18** in d_4-methanol.

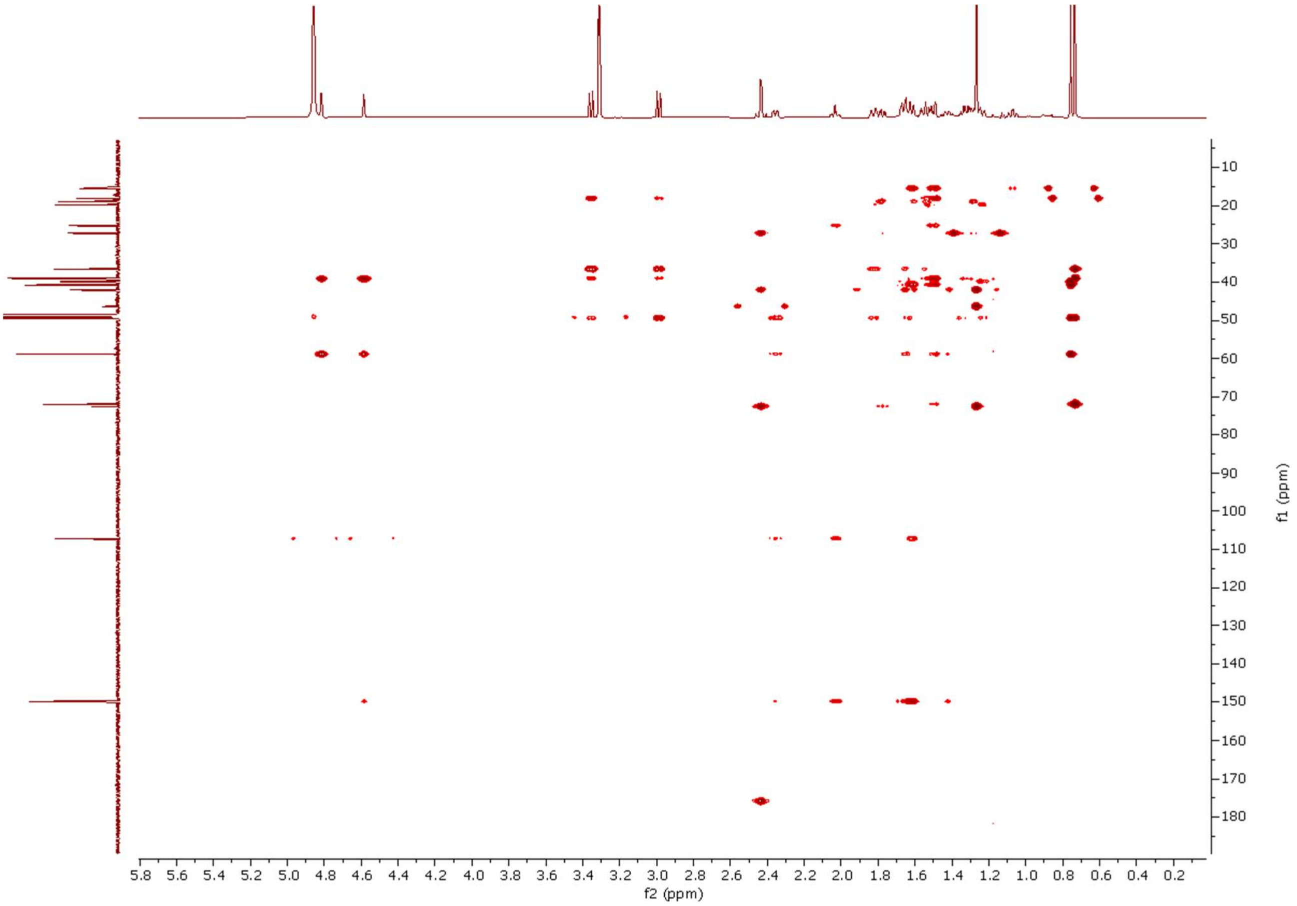

Figure N18.E HMBC NMR of **18** in d$_4$-methanol.

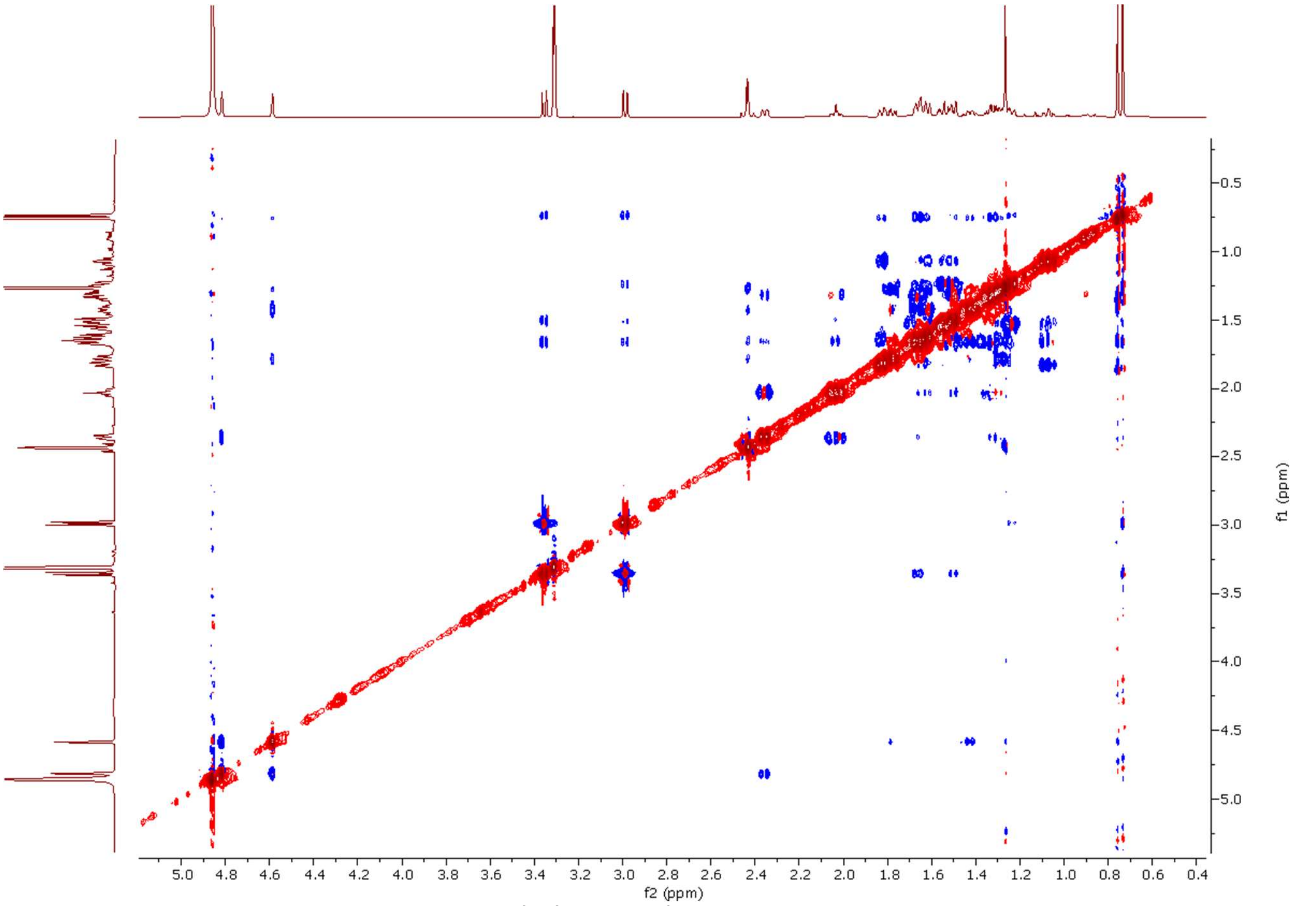

Figure N18.F NOESY NMR of **18** in d$_4$-methanol at 500 MHz.

16α,17-Dihydroxy-*ent*-kauranoic acid (20)

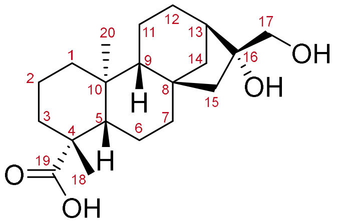

16α,17-Dihydroxy-*ent*-kauranoic acid (**20**)
Chemical Formula: $C_{20}H_{32}O_4$
Exact Mass: 336.2301

	Reference in DMSO-d$_6$ [44]		Measured in DMSO-d$_6$, 298K		
**Pos.**	δ_C 75 MHz	δ_H (*J*/Hz) 300 MHz	δ_C 151 MHz	δ_H (*J*/Hz) 600 MHz	**Selected NOESY correlations to H [a]**
**1**	41.1	n.a.	40.2	ax 0.74, ddd (13.5, 13.5 3.7)<br>eq 1.75, overlapped	H-3ax
**2**	19.8	n.a.	18.8	a 1.31, overlapped<br>b 1.78, overlapped	H-3eq<br>H-3eq
**3**	38.7	n.a.	37.7	ax 0.92, overlapped<br>eq 1.99, ddd (12.5, 3.5, 3.5)	H-1ax, H-3eq<br>H-3ax, H$_3$-18, H-2a, H-2b
**4**	43.9	-	42.8	-	-
**5**	57.0	n.a.	56.0	0.97, dd (4.0, 10.6)	
**6**	23.0	n.a.	22.0	a 1.68, overlapped<br>b 1.72, overlapped	
**7**	42.8	n.a.	41.9	a 1.34, overlapped<br>b 1.49, overlapped	
**8**	45.0	-	44.0	-	-
**9**	56.3	n.a.	55.4	0.91, overlapped	
**10**	40.1	-	39.1	-	-
**11**	19.0	n.a.	18.2	a 1.47, overlapped<br>b 1.50, overlapped	
**12**	26.8	n.a.	25.9	a 1.32, overlapped<br>b 1.53, overlapped	
**13**	45.9	n.a.	44.5	1.88, br d (3.2)	
**14**	37.8	n.a.	36.9	a 1.53, overlapped<br>b 1.72, overlapped	
**15**	53.9	n.a.	52.8	a 1.25, d (14.1)<br>b 1.39, br d (14.1)	
**16**	81.3	-	80.5	-	-
**17**	65.8	n.a.	65.3	3.39, d (11.0)<br>3.50, d (11.0)	H-15a, H-15b, H-11b, H-13<br>H-11b, H-15b
**18**	28.8	1.21, s	28.6	1.09, s	
**19**	179.7	-	178.7	-	-
**20**	15.5	0.96, s	15.4	0.87, s	

[a] Key NOESY correlations are shown in blue text.

n.a. = not assigned.

The published reference data in DMSO-d$_6$ reported only few assigned ^{1}H signals and broad unassigned multiplets that cannot be correlated to individual protons. 0.96 (s, 3H, -Me, H-20), 1, 21 (s, 3H, -Me, H-18), 3.86 (dd, AB quartet, 11 Hz, - OH, H-17), 4.34 (dd, AB quartet, 11 Hz, -OH, H-16), 11.78 (brs, 1H, -COOH, H-19), 0.96– 2.01 (m, 26H). Assignments of reference ^{1}H data in our table were made based on close similarity to our data, which were fully supported by 2D NMR measurements. Other reference signals remain unassigned.

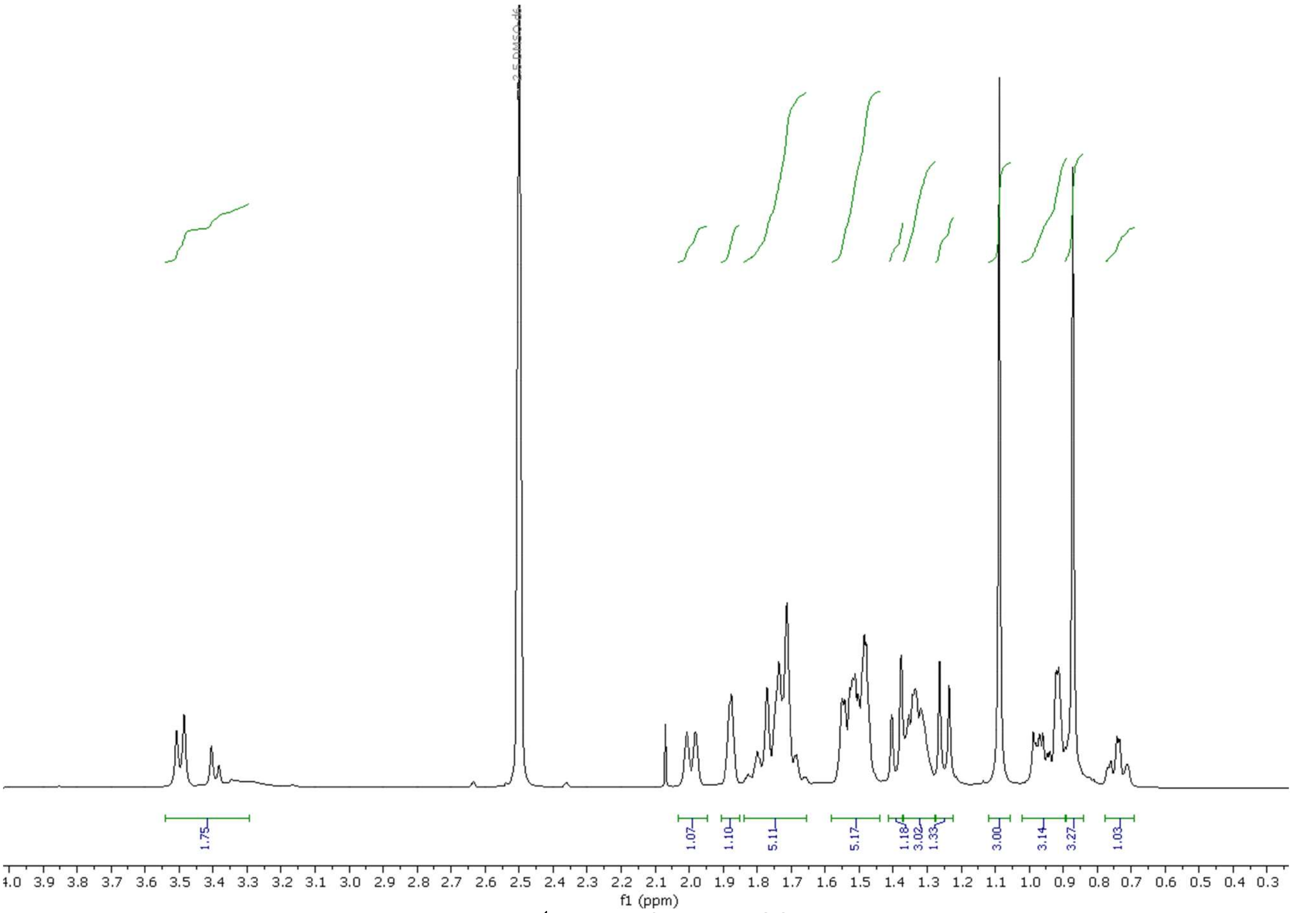

Figure N20.A ^{1}H NMR of **20** in DMSO-d_6 at 600 MHz.

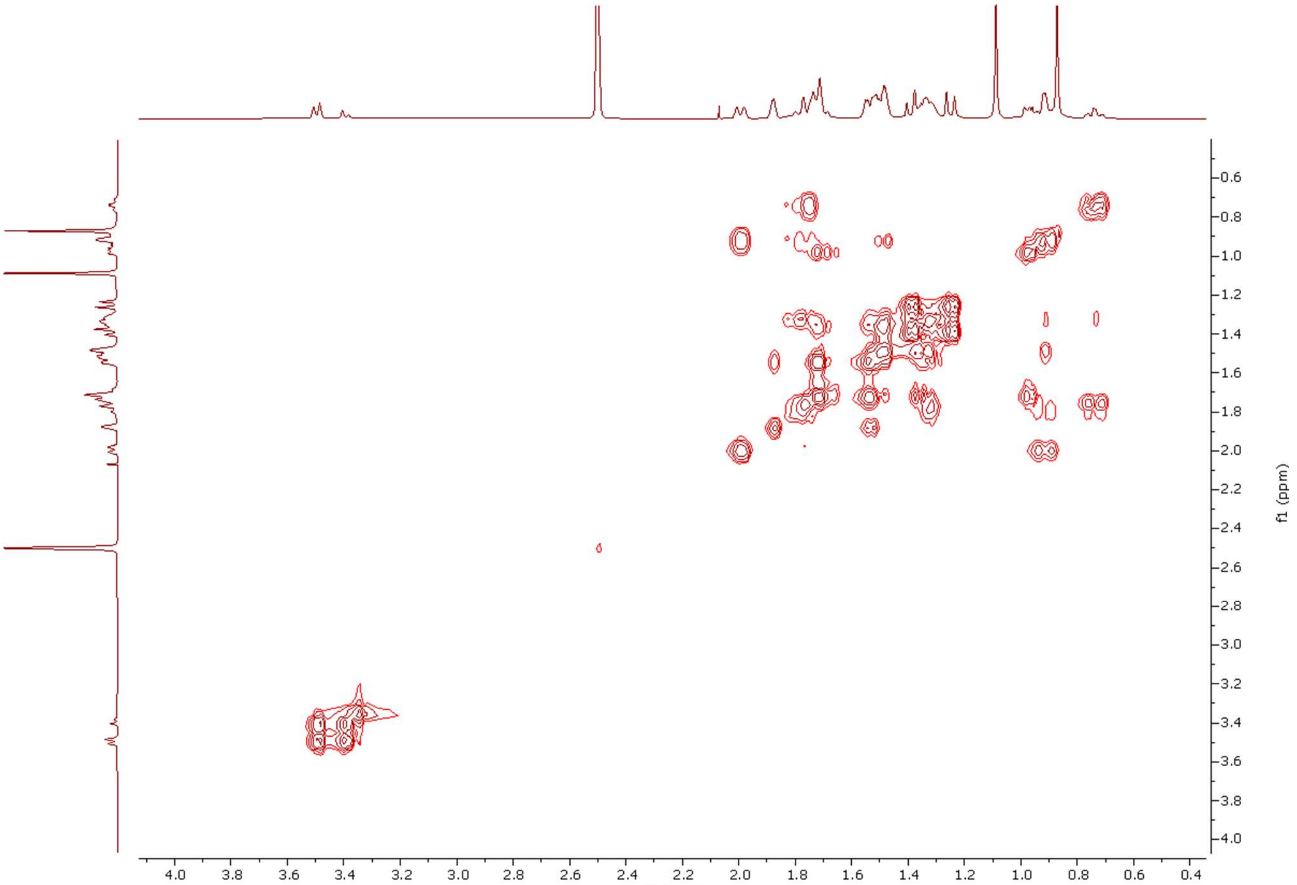

Figure N20.B COSY NMR of **20** in DMSO-d_6 at 600 MHz.

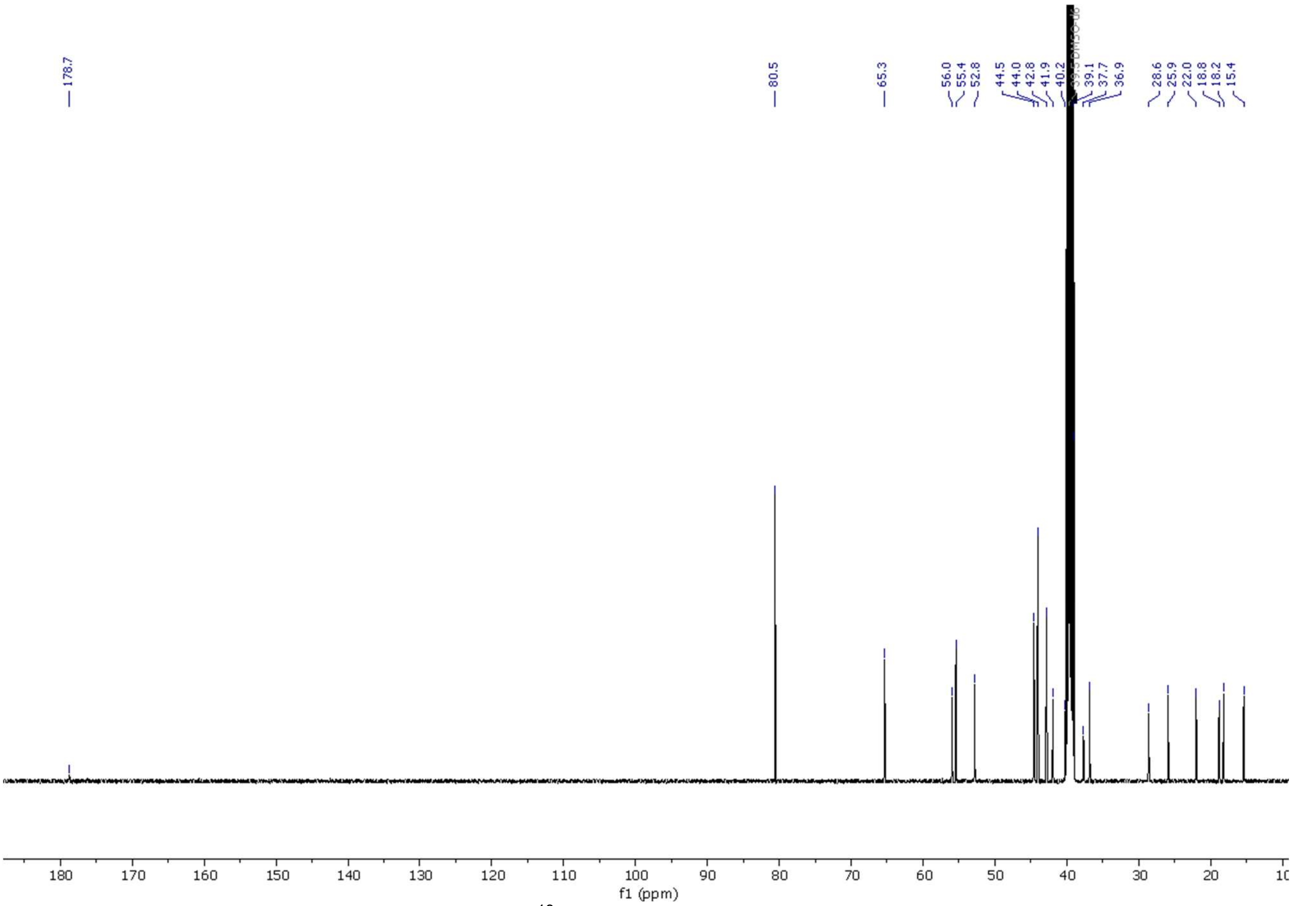

Figure N20.C ^{13}C NMR of **20** in DMSO-d$_6$ at 151 MHz.

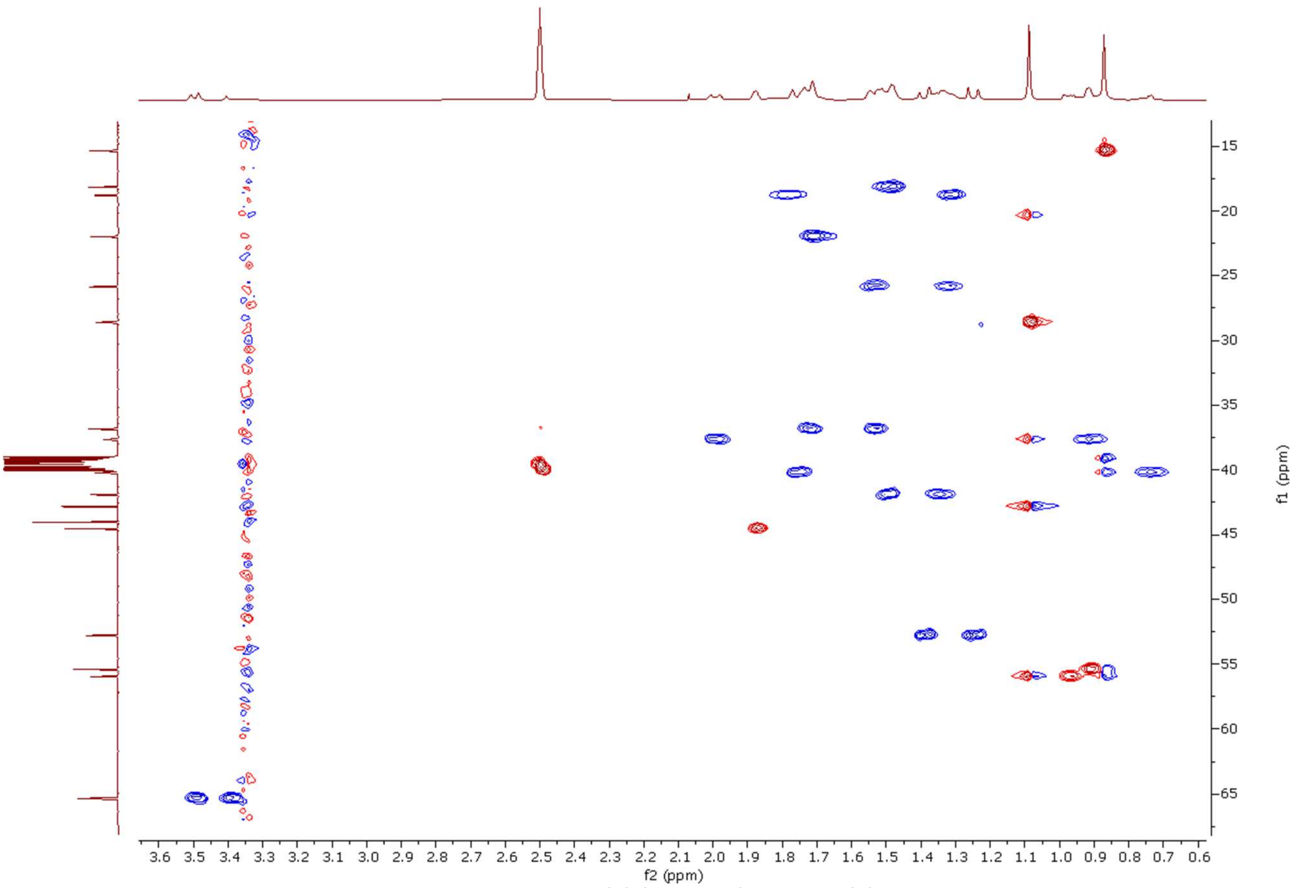

Figure N20.D HSQC NMR of **20** in DMSO-d$_6$.

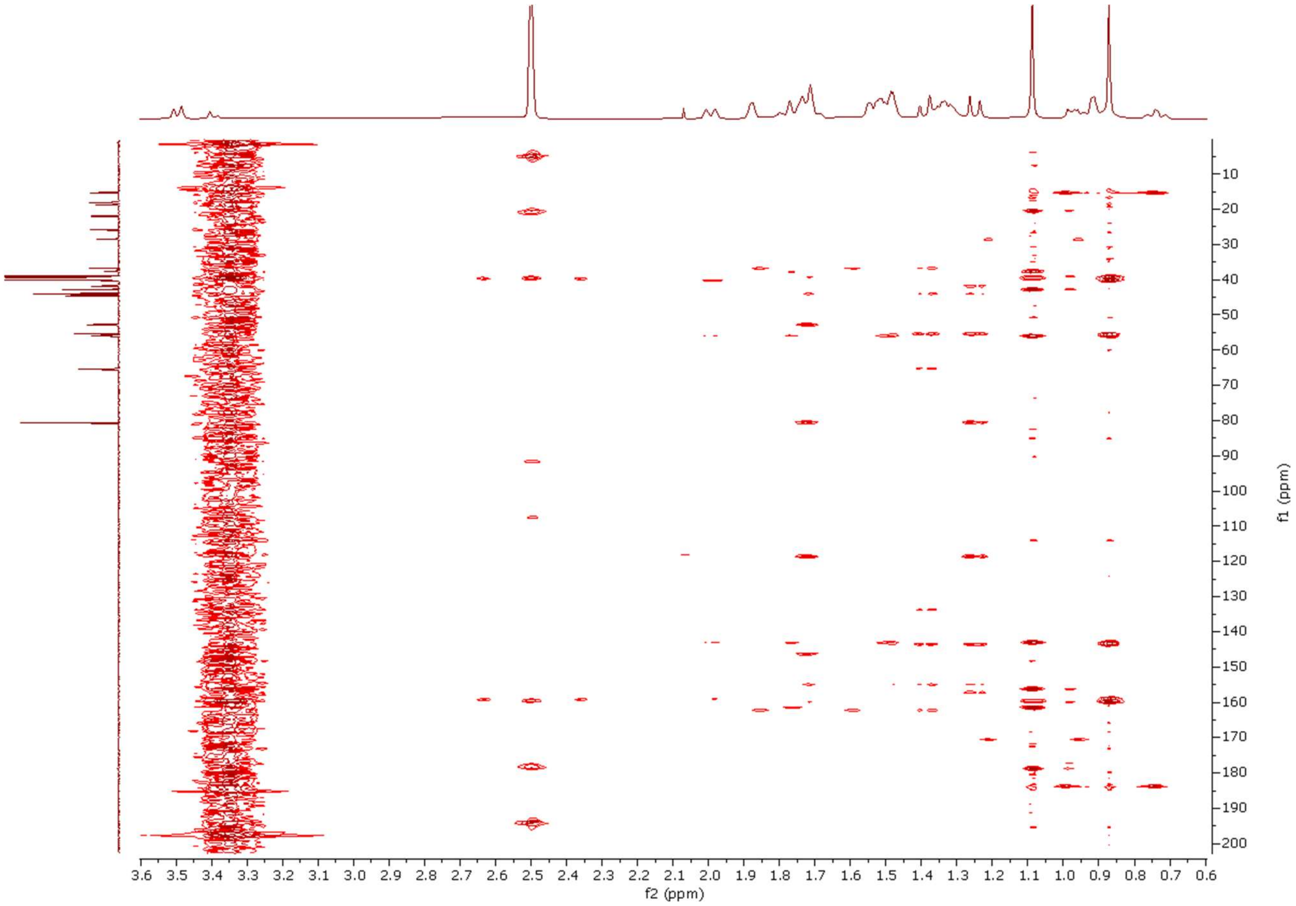

Figure N20.E HMBC NMR of **20** in DMSO-d$_6$.

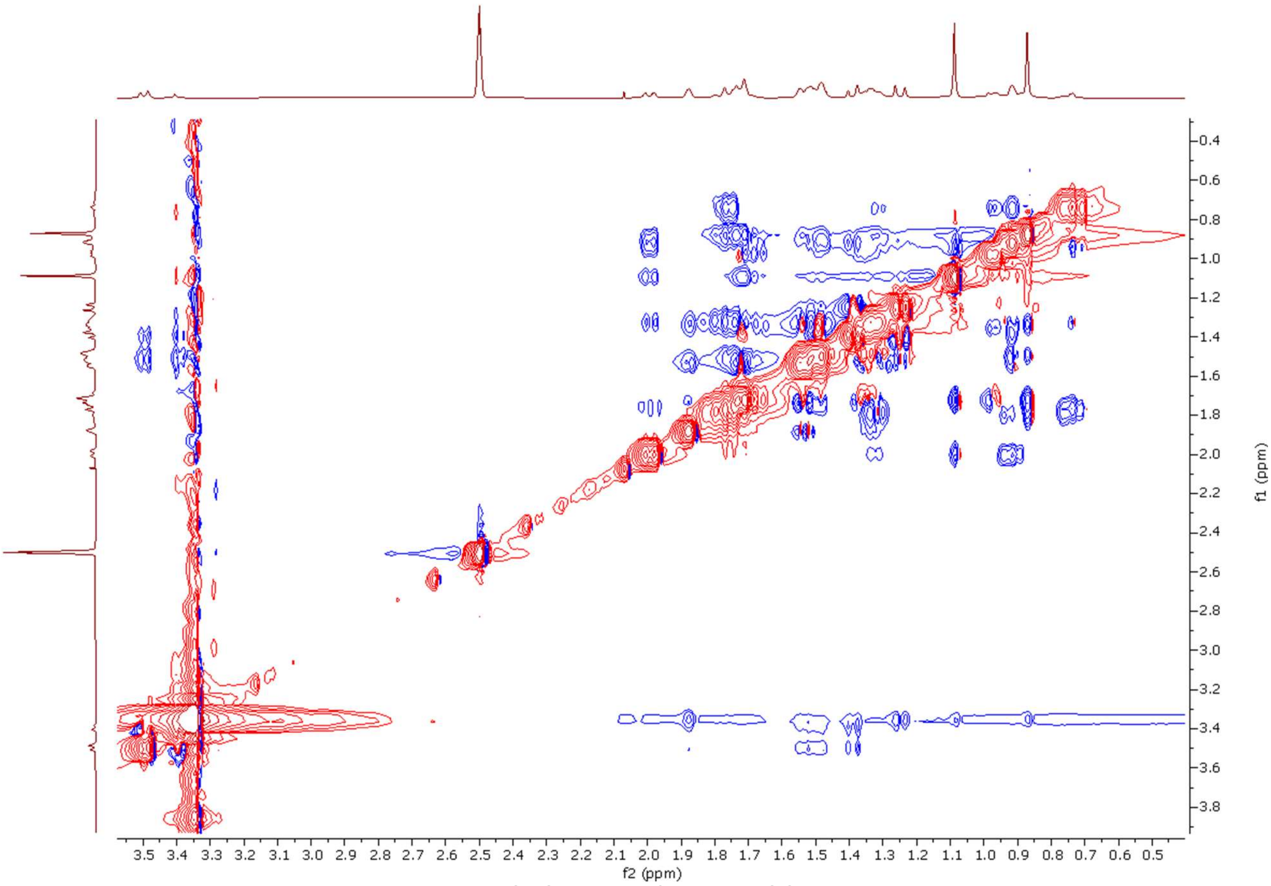

Figure N20.F NOESY NMR of **20** in DMSO-d$_6$ at 600 MHz.

16β,17-Dihydroxy-ent-kauranoic acid (21)

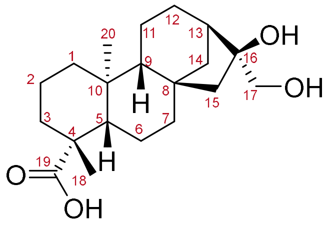

16β,17-Dihydroxy-*ent*-kauranoic acid (**21**)
Chemical Formula: $C_{20}H_{32}O_4$
Exact Mass: 336.2301

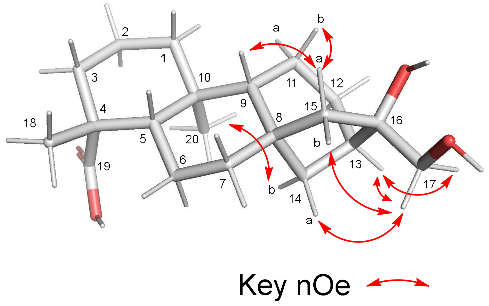

	Reference in DMSO-d$_6$ [45] (only δ_C reported)	Measured in DMSO-d$_6$, 298K		
**Pos.**	δ_C 125 MHz	δ_C 151 MHz	δ_H (J/Hz) 600 MHz	**Selected NOESY correlations to H [a]**
**1**	40.8	40.4	ax 0.77, ddd (13.5, 13.5, 3.9)<br>eq 1.80, overlapped	H-5, H-1eq
**2**	19.3	18.8	a 1.32, overlapped<br>b 1.80, overlapped	
**3**	38.1	37.7	ax 0.92, ddd (13.5, 13.5, 4.2)<br>eq 1.99, overlapped	H-3eq<br>H-3ax
**4**	n.r.	42.8	-	-
**5**	56.5	56.1	0.97, overlapped	
**6**	22.0	21.5	a 1.67, overlapped<br>b 1.69, overlapped	
**7**	n.r.	41.6	a 1.29, overlapped<br>b 1.33, overlapped	
**8**	43.6	43.1	-	-
**9**	56.3	55.8	0.97, overlapped	H-15a
**10**	39.6	39.2	-	-
**11**	19.0	18.5	a 1.45, dd (14.8, 6.1)<br>b 1.97, overlapped	H_3-20, H-1eq, H-11b<br>H-11a
**12**	27.1	26.6	a 1.29, overlapped<br>b 1.70, overlapped	H-13<br>H-13
**13**	n.r.	40.3	1.91, dd (6.5, 3.7)	H-14a, H-12a, H-12b, H-17a, H-17b
**14**	n.r.	37.7	a 1.00, overlapped<br>b 1.78, overlapped	H-13<br>H_3-20
**15**	52.7	52.3	a 1.24, dd (14.0, 2.5)<br>b 1.30, overlapped	H-11b, H-9<br>H-13, H-14a, H-17a, H-17b
**16**	79.1	78.6	-	-
**17**	69.6	69.1	a 3.12, d (10.9)<br>b 3.22, d (10.9)	H-15b, H-13, H-14a<br>H-15b, H-13, H-14a
**18**	29.1	28.6	1.09, s	H-3ax, H-3eq
**19**	179.1	178.<br>7	-	-
**20**	15.7	15.3	0.89, s	H-11a, H-14b

[a] Key NOESY correlations are shown in blue text.

n.r. = not reported.

^{13}C data of the reference are extracted from figure S17 of reference [45].

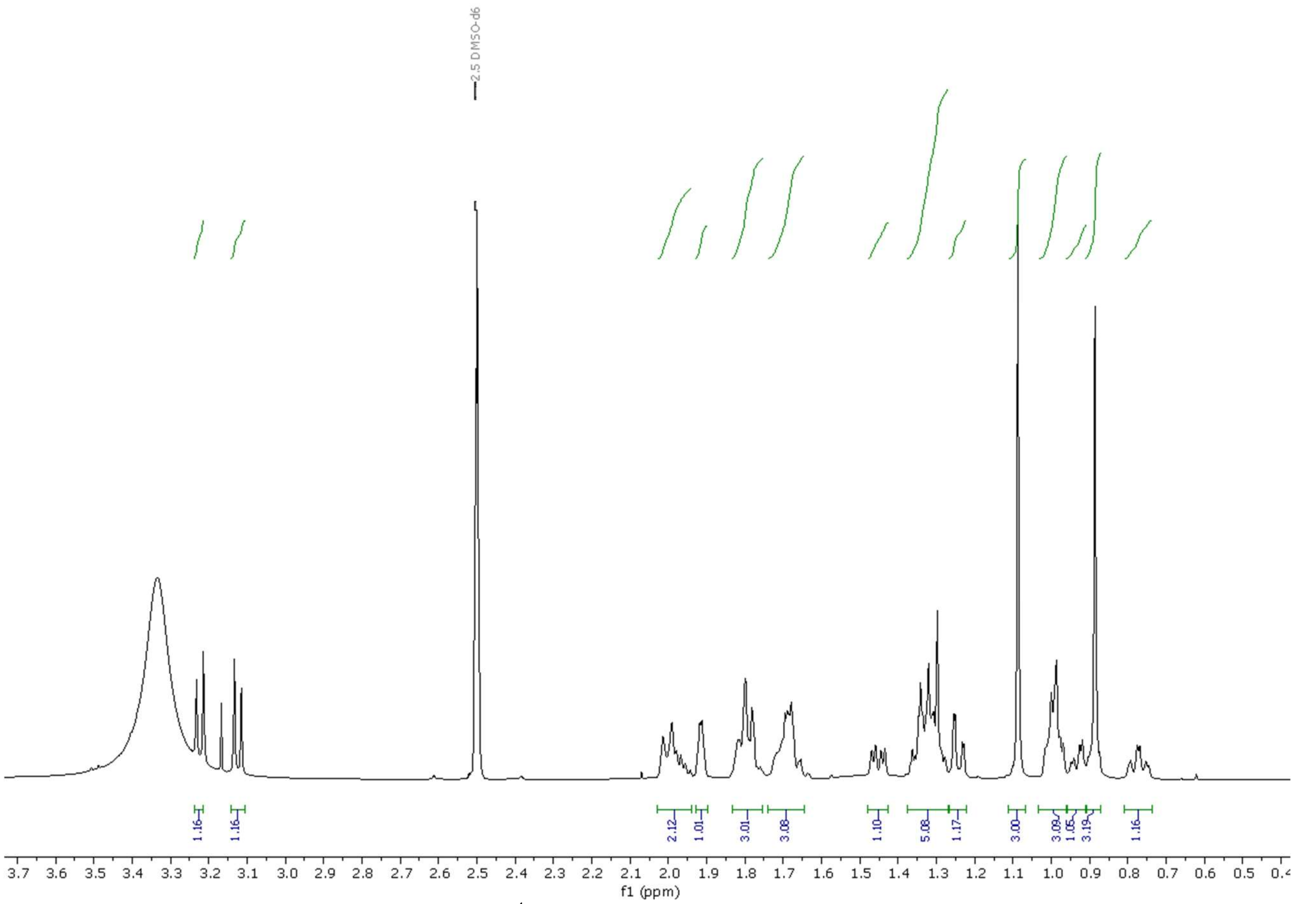

Figure N21.A ^{1}H NMR of **21** in DMSO-d$_6$ at 600 MHz.

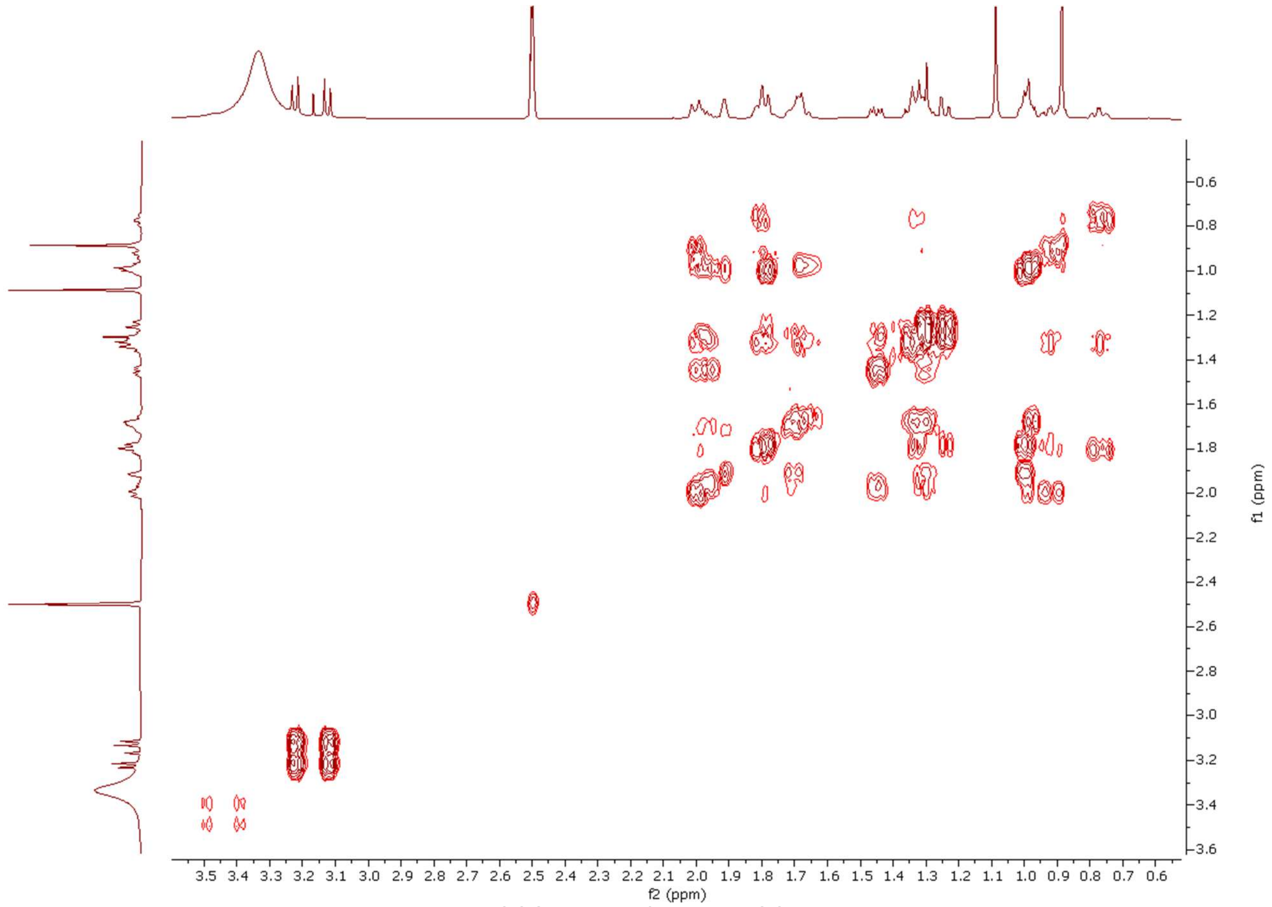

Figure N21.B COSY NMR of **21** in DMSO-d$_6$ at 600 MHz.

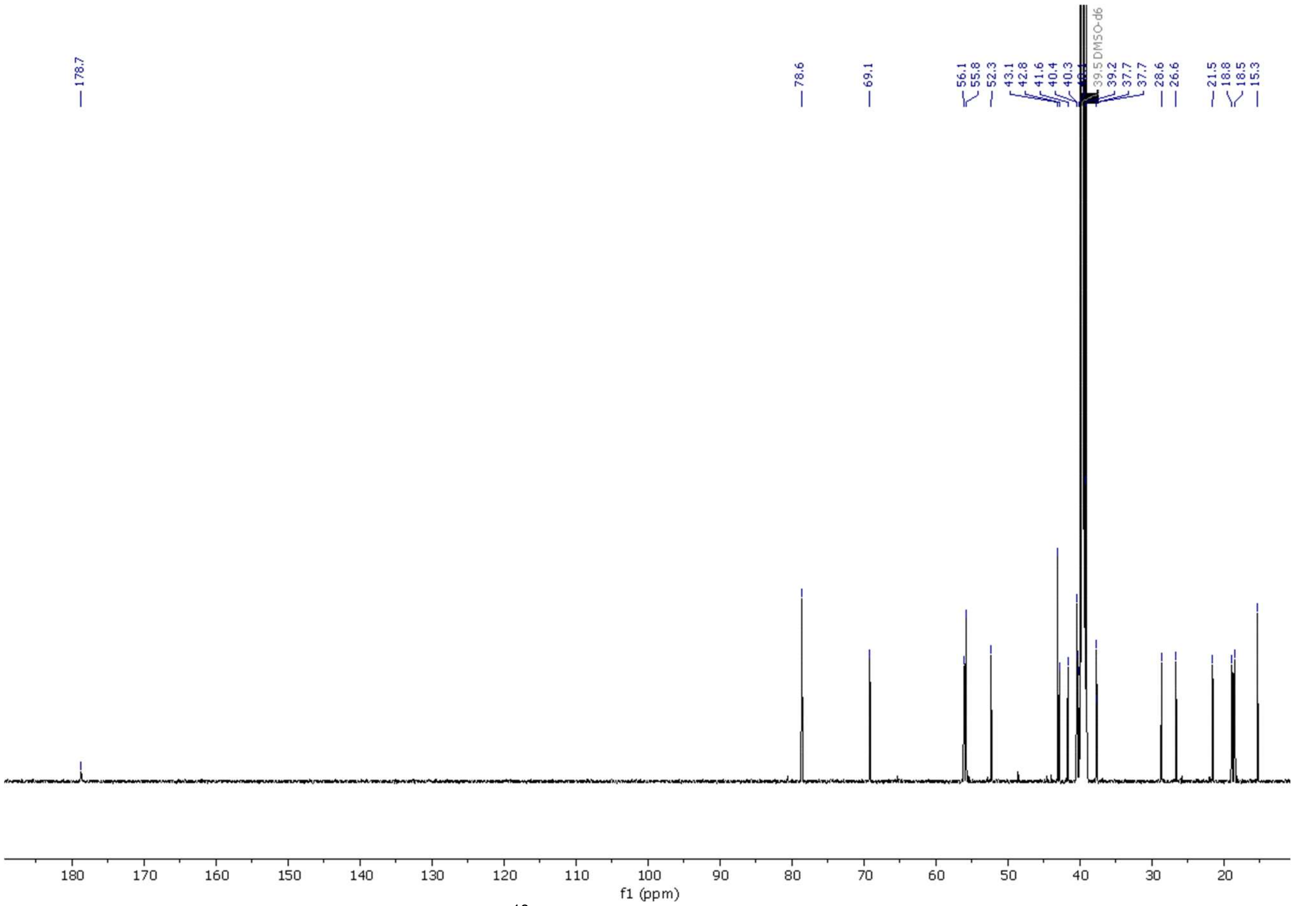

Figure N21.C ^{13}C NMR of **21** in DMSO-d_6 at 151 MHz.

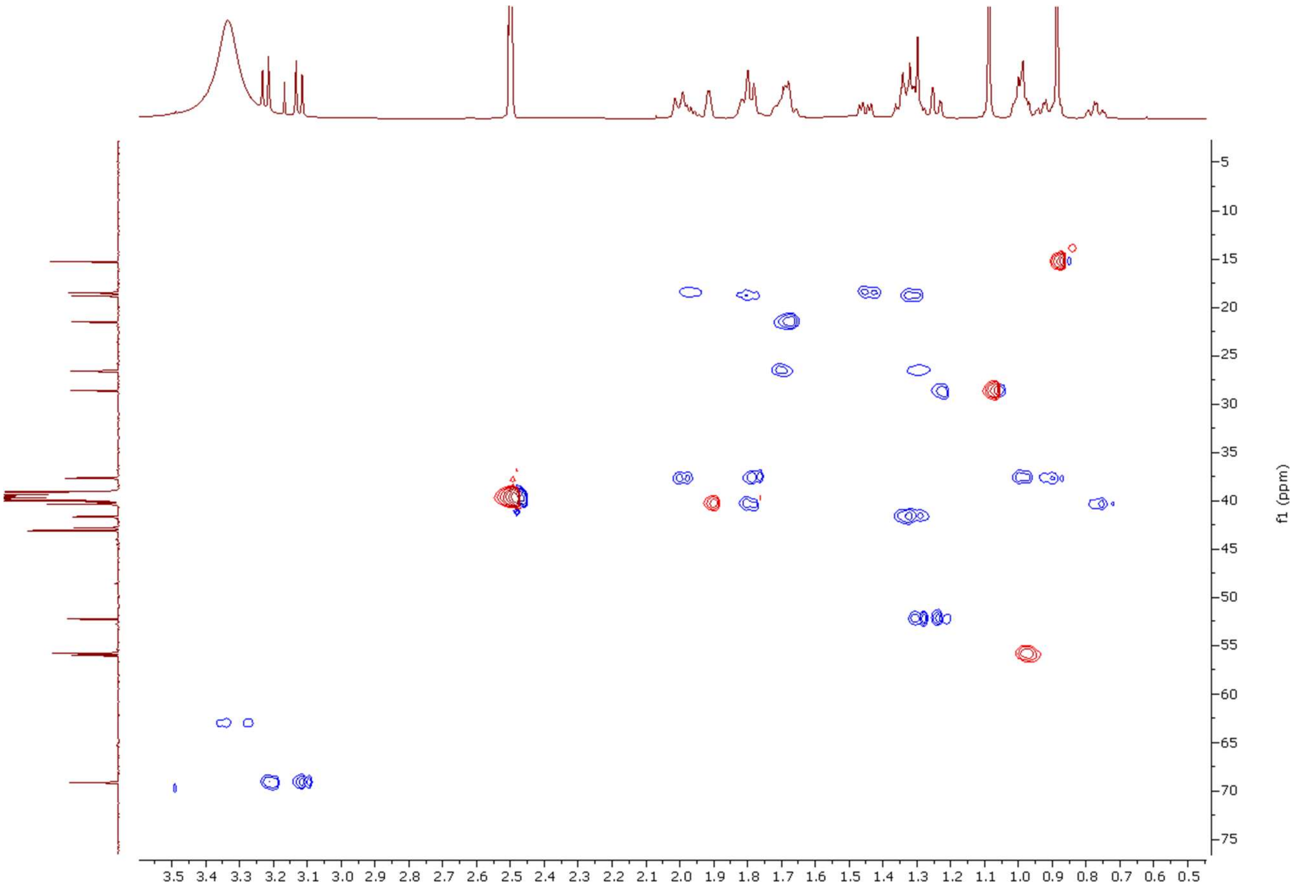

Figure N21.D HSQC NMR of **21** in DMSO-d_6.

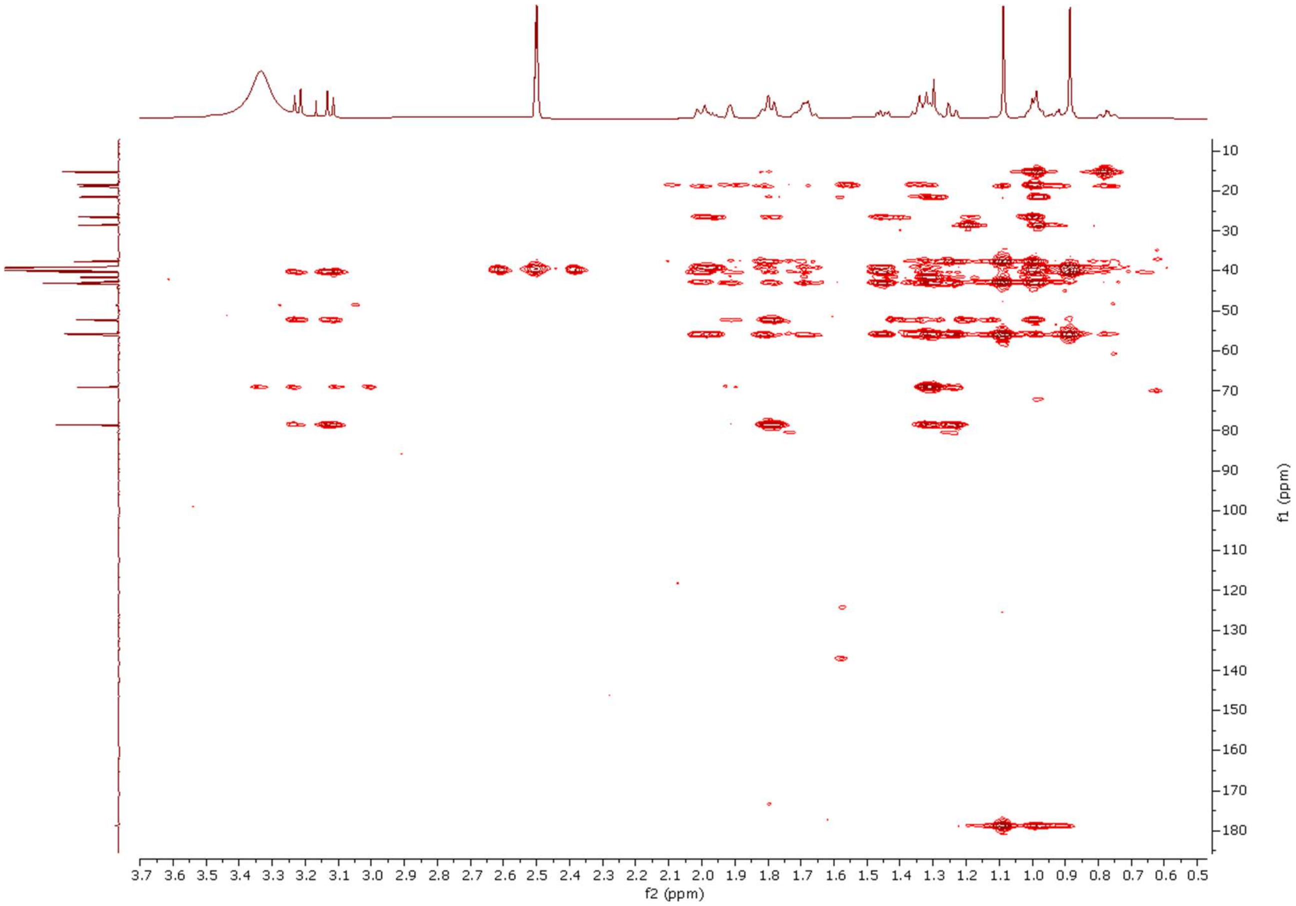

Figure N21.E HMBC NMR of **21** in DMSO-d$_6$.

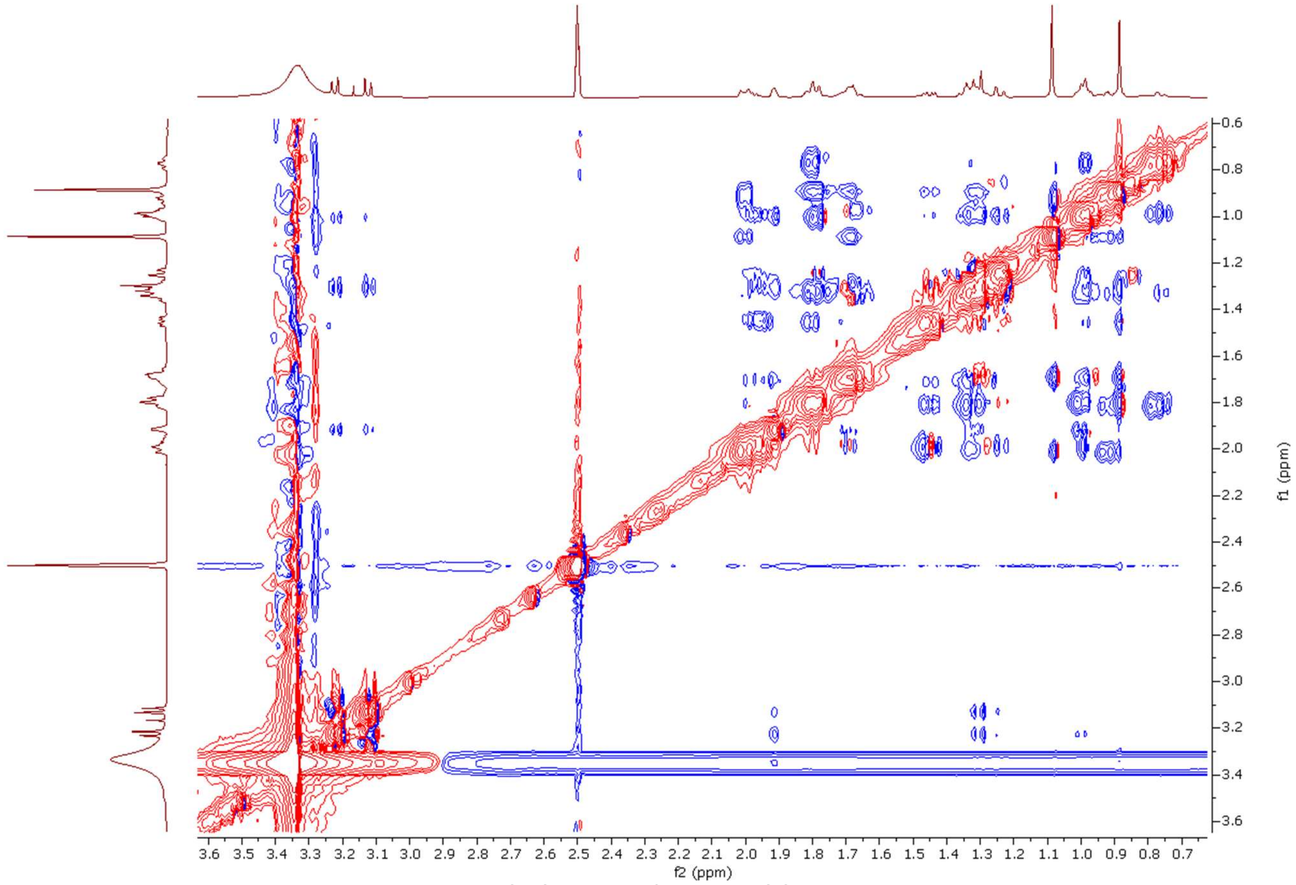

Figure N21.F NOESY NMR of **21** in DMSO-d$_6$ at 600 MHz.

7β-Hydroxy-*ent*-kaurenoic acid (22)

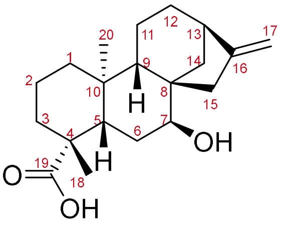

7β-Hydroxy-*ent*-kaurenoic acid (**22**)
Chemical Formula: $C_{20}H_{30}O_3$
Exact Mass: 318.2195

	**Reference in $CDCl_3$ [46]**		**Measured in $CDCl_3$, 298K**		**Measured in d_4-methanol, 298K**		
**Pos.**	δ_C 50 MHz	δ_H, (J/Hz) 200 MHz	δ_C 151 MHz	δ_H (J/Hz) 600 MHz	δ_C 151 MHz	δ_H (J/Hz) 600 MHz	**Selected NOESY correlations to H [a]**
**1**	40.4	n.r.	40.5	0.91, ddd (14.2, 14.2, 4.2)<br>1.86, overlapped	41.8	ax 0.92, ddd (13.4, 13.4, 4.1)<br>eq 1.87, overlapped	
**2**	19.1	n.r.	19.2	1.48, overlapped<br>1.87, overlapped	20.4	a 1.43, m<br>b 1.91, overlapped	
**3**	37.8	n.r.	37.9	1.09, ddd (13.6, 13.6, 4.3)<br>2.19, m	39.2	ax 1.06, ddd (13.4, 13.4, 4.2)<br>eq 2.15, ddd (13.4, 2.7, 2.7)	H-3eq<br>H-3ax
**4**	43.2	-	43.3	-	44.2	-	-
**5**	49.1	n.r.	47.3	1.77, dd (13.2, 2.2)	48.2	1.74, dd (13.2, 2.2)	H_3-18, H-9, H-6eq
**6**	29.7	n.r.	29.1	1.99, ddd (14.7, 3.8, 2.2)<br>2.12, ddd (14.7, 13.2, 2.2)	30.4	eq 1.97, ddd (14.1, 2.2, 2.2)<br>ax 2.09, ddd (14.1, 13.2, 1.5)	H-6ax, H_3-18, H-7, H-5<br>H-6eq, H-7
**7**	77.1	3.63, s	77.2	3.63, dd (3.8, 2.2)	78.0	3.53, dd (2.2, 1.5)	H-14a, H-6ax, H-6eq, H_2-15
**8**	48.3	-	48.3	-	49.4	-	-
**9**	47.2	n.r.	49.2	1.47, overlapped	50.4	1.47, overlapped	H-5
**10**	39.4	-	39.5	-	40.4	-	-
**11**	17.9	n.r.	18.1	1.58, m	19.0	1.59, m	
**12**	33.5	n.r.	33.7	1.50, overlapped<br>1.65, m	34.6	a 1.47, overlapped<br>b 1.68, m	H-13<br>H-13
**13**	43.7	2.59, m	43.8	2.68, br s	45.2	2.64, br s	H-17b, H-12b, H-12a, H-14a
**14**	38.7	n.r.	38.8	1.20, ddt (11.4, 5.0, 1.5)<br>1.86, overlapped	40.0	a 1.17, overlapped<br>b 1.89, overlapped	H-13<br>H_3-20
**15**	45.3	n.r.	45.4	2.24, m	46.6	2.23, br s	H-17a, H-7
**16**	155.1	-	155.0	-	156.7	-	-
**17**	103.7	4.80, s<br>4.82, s	103.8	4.80, s<br>4.83, s	103.8	a 4.76, br s<br>b 4.79, br s	H-17b, H_2-15<br>H-17a, H-13
**18**	28.7	1.24, s	28.9	1.24, s	29.3	1.18, s	H-6eq, H-5
**19**	160.0	-	182.5	-	182.0	-	-
**20**	15.5	0.96, s	15.7	0.96, s	16.3	0.99, s	H-1eq, H-14b, H-6ax

[a] Key NOESY correlations are shown in blue text.

n.r. = not reported.

Yellow highlighted data are possibly swapped assignments in the referenced data. Our assignments are supported by HMBC correlations.

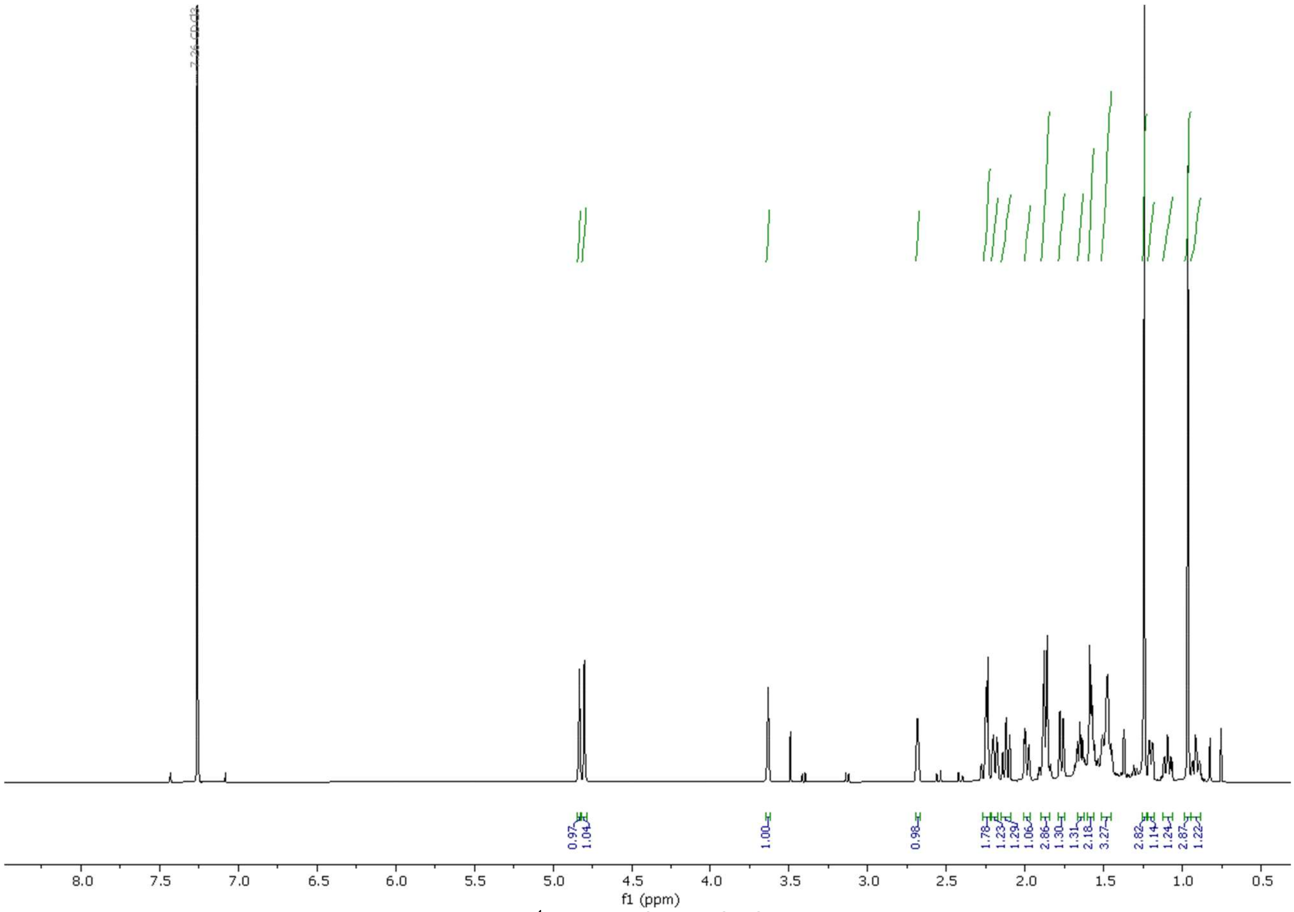

Figure N22.A ^{1}H NMR of **22** in $CDCl_3$ at 600 MHz.

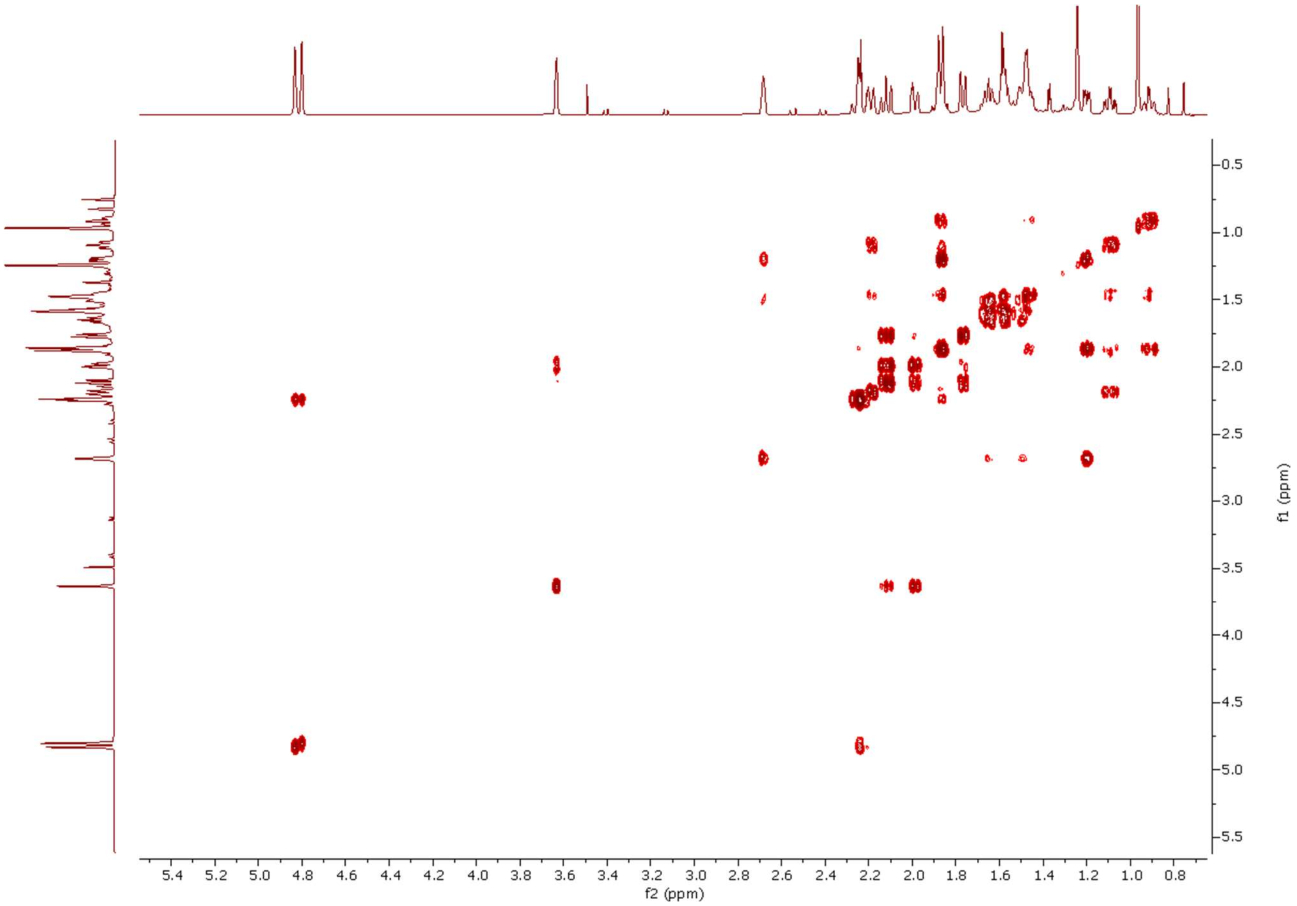

Figure N22.B COSY NMR of **22** in $CDCl_3$ at 600 MHz.

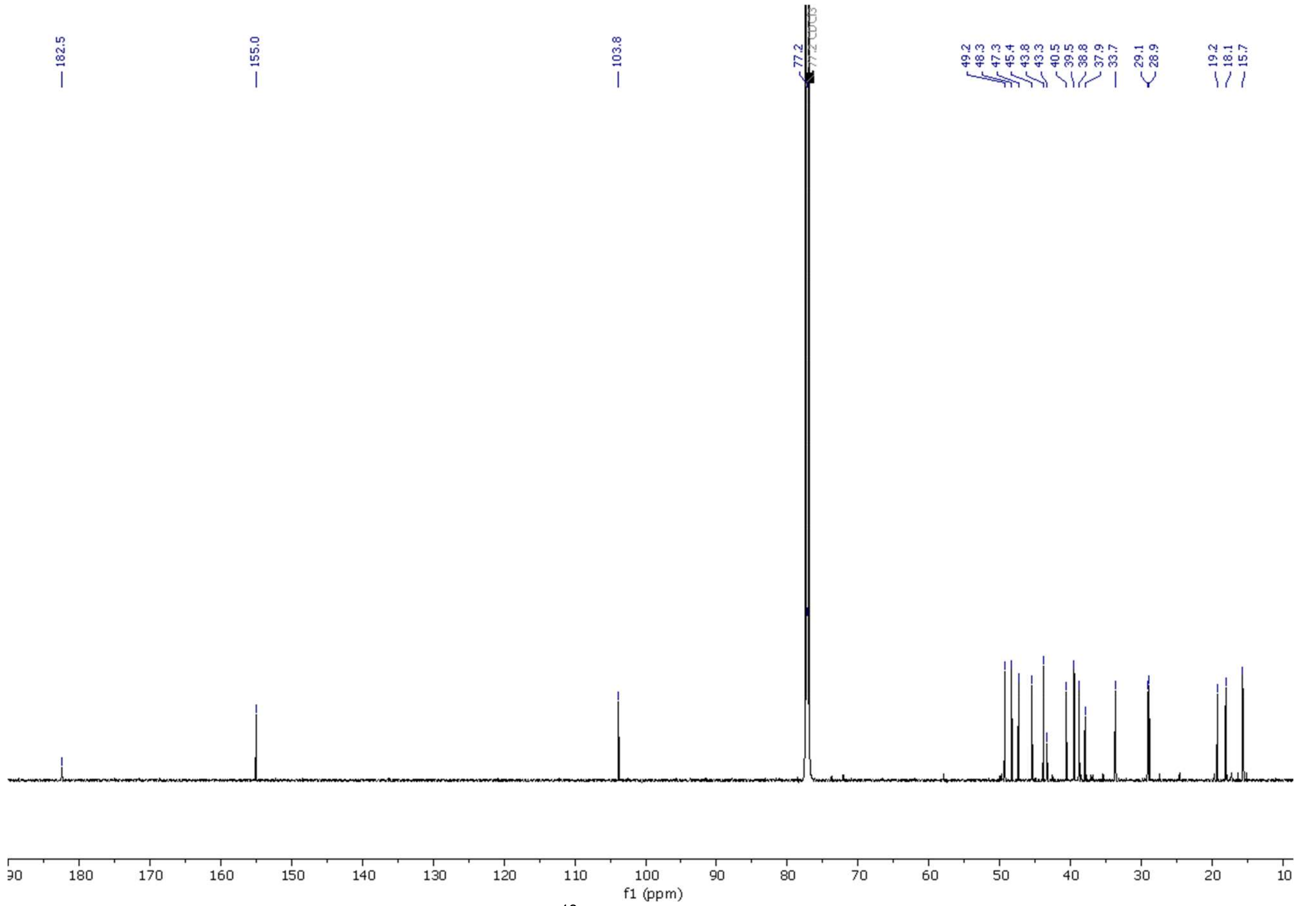

Figure N22.C ^{13}C NMR of **22** in $CDCl_3$ at 151 MHz.

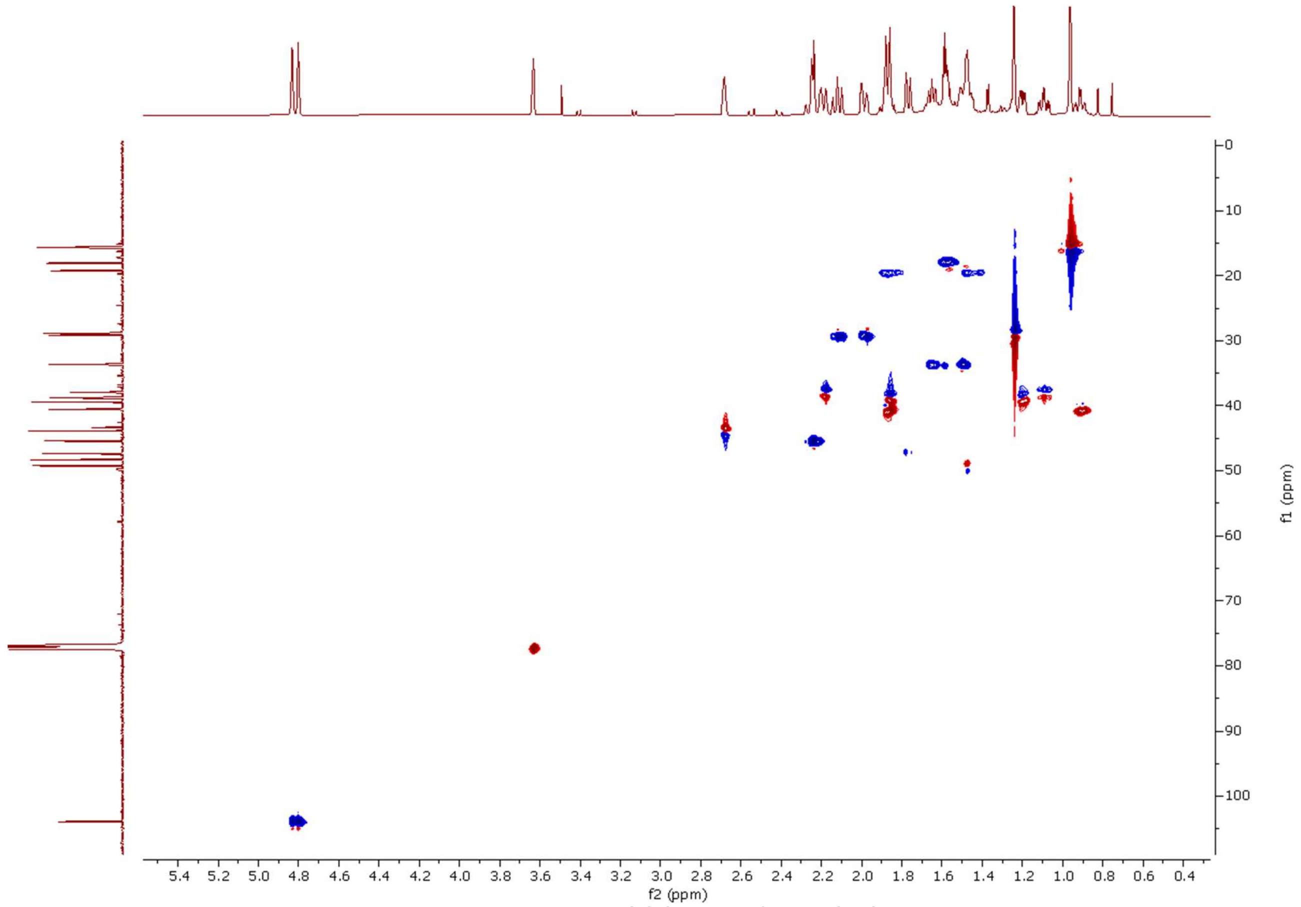

Figure N22.D HSQC NMR of **22** in $CDCl_3$.

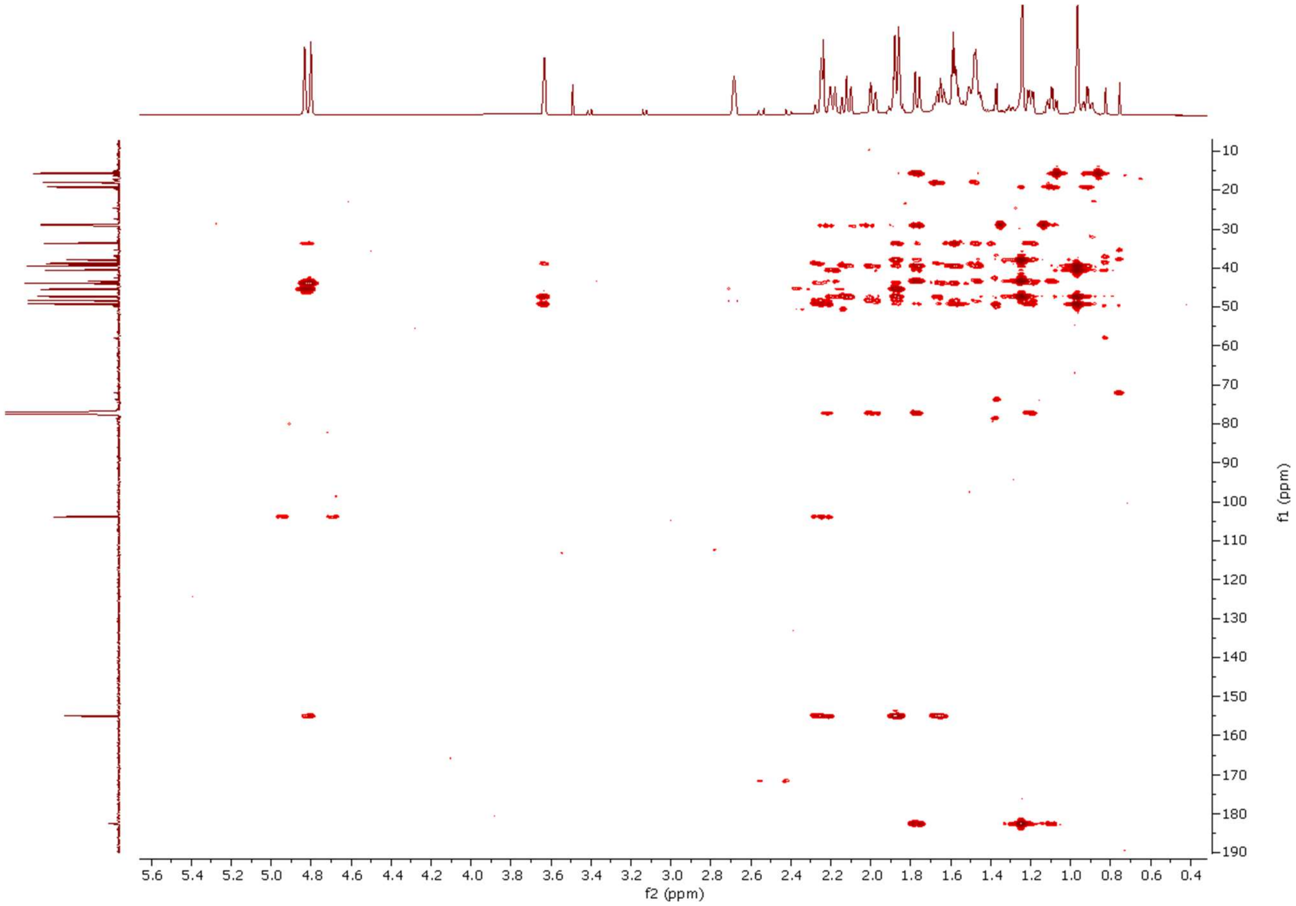

Figure N22.E HMBC NMR of **22** in $CDCl_3$.

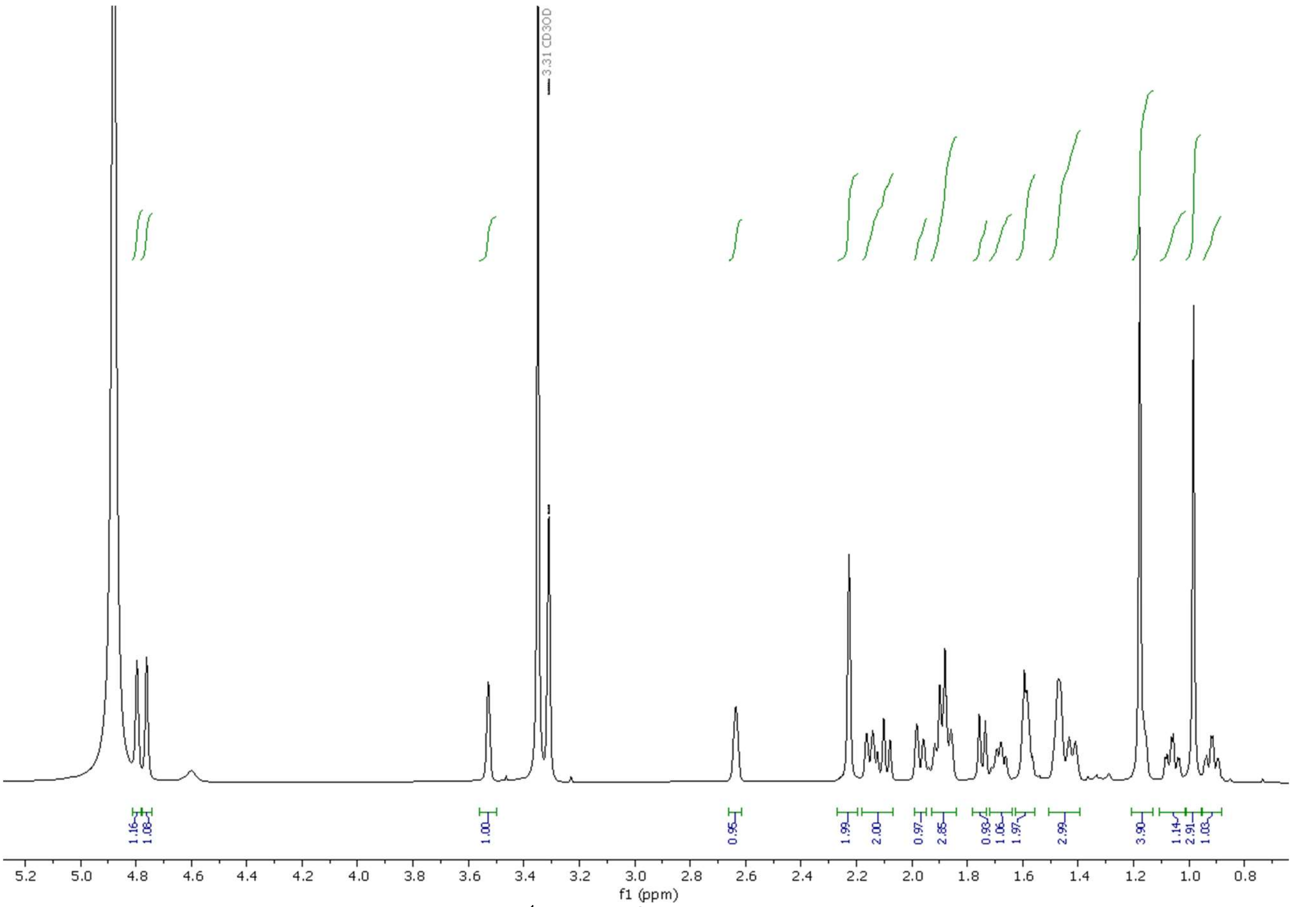

Figure N22.F ^1H NMR of **22** in d_4-methanol at 600 MHz.

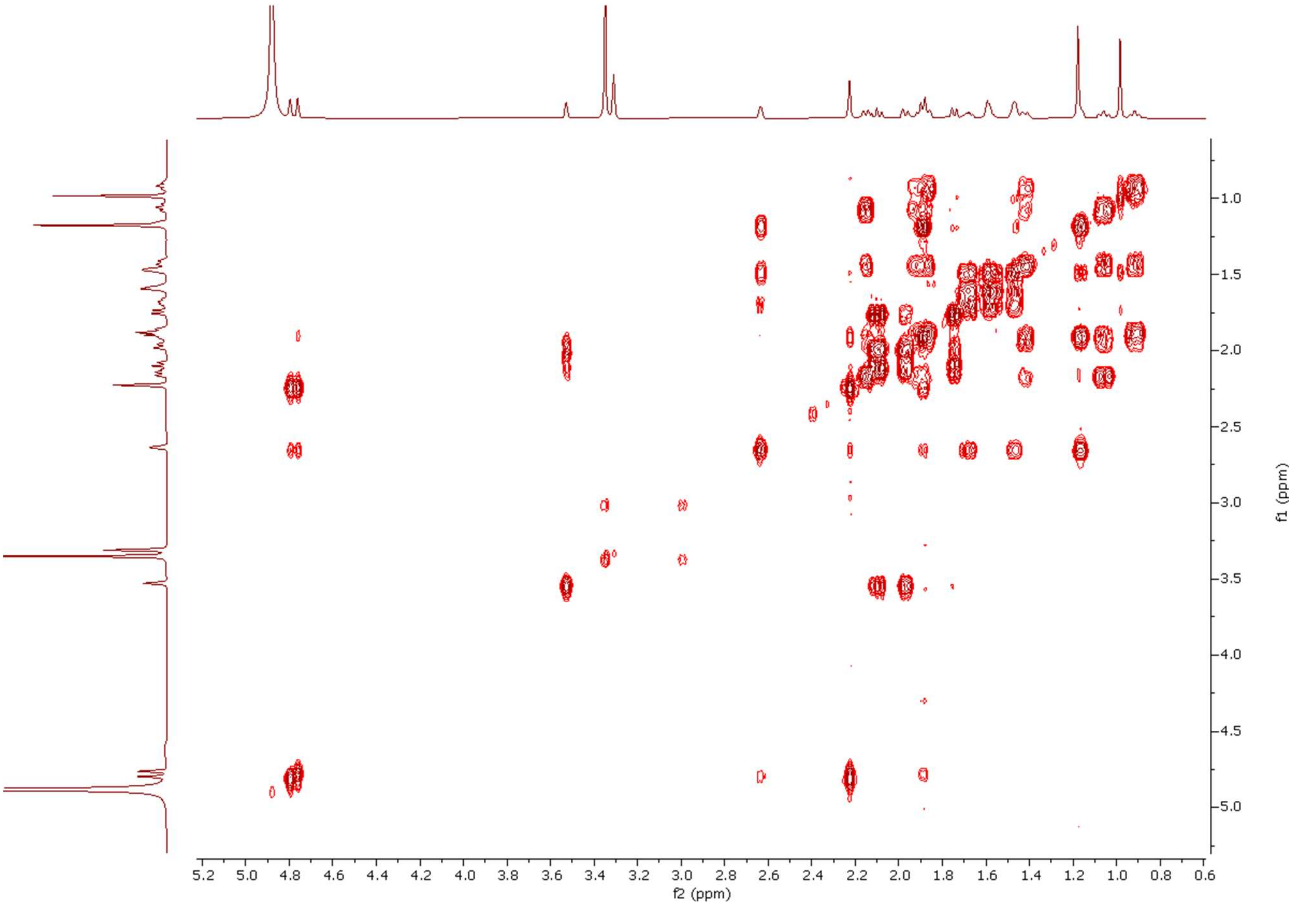

Figure N22.G COSY NMR of **22** in d_4-methanol at 600 MHz.

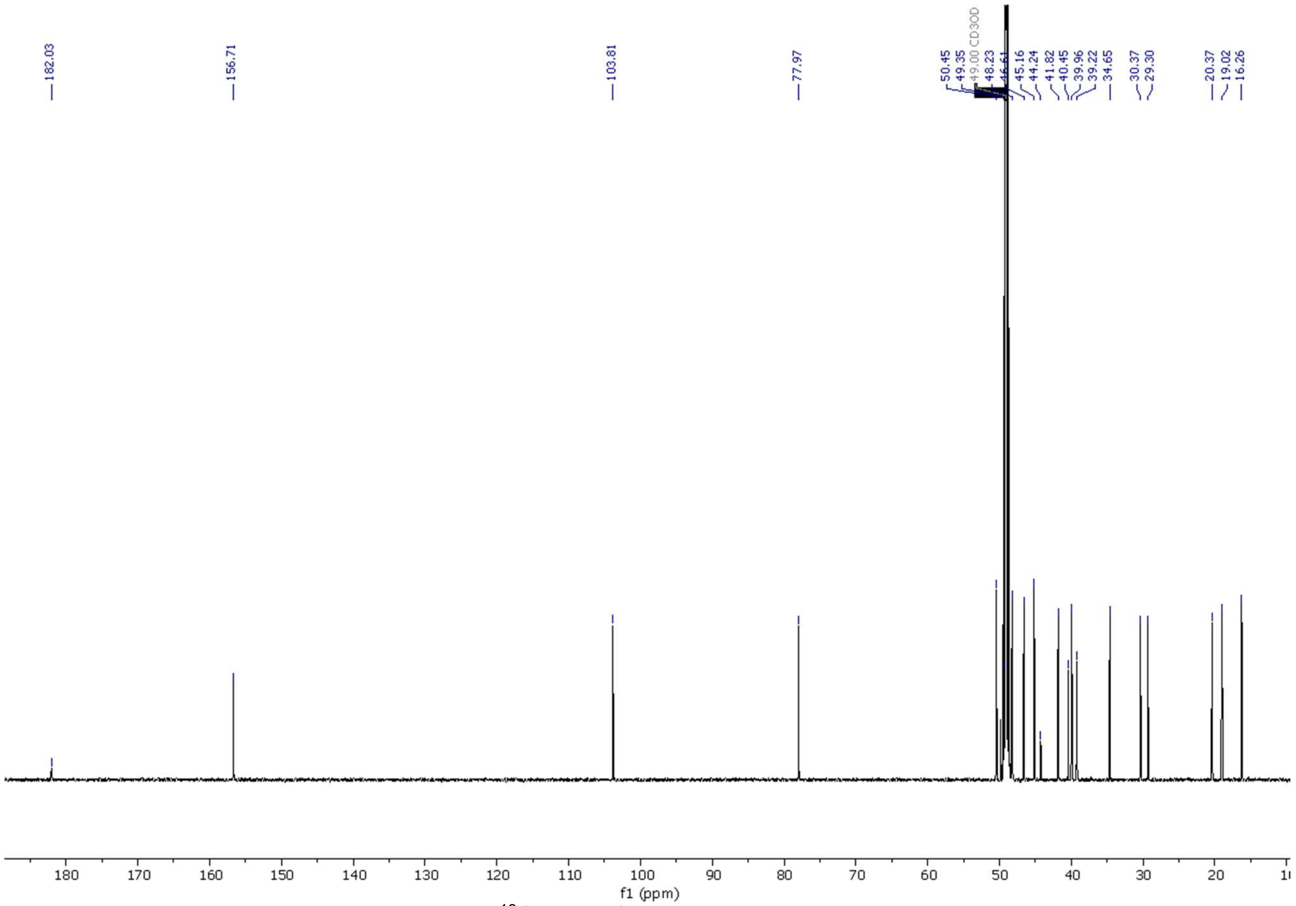

Figure N22.H ^{13}C NMR of **22** in d_4-methanol at 151 MHz.

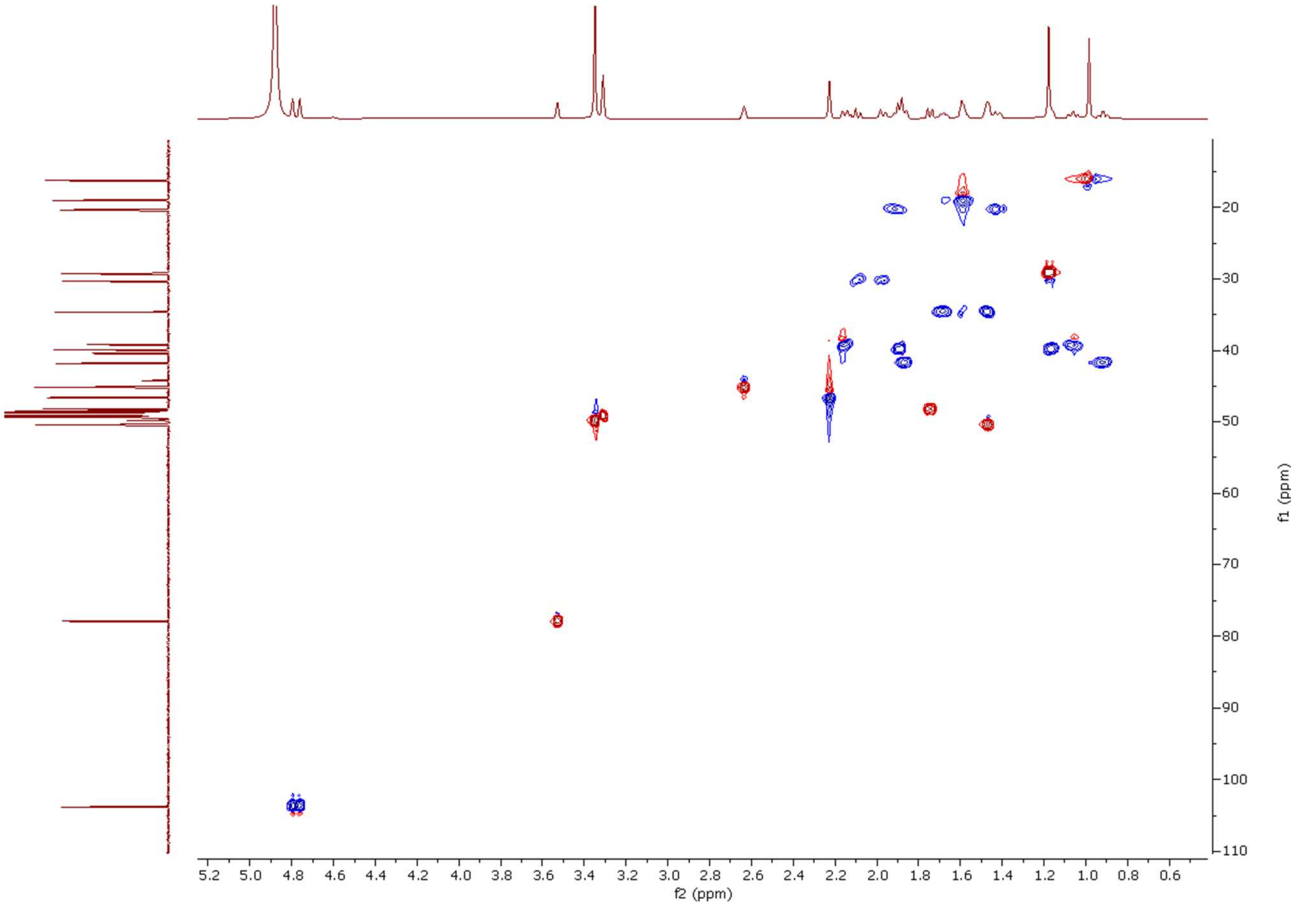

Figure N22.I HSQC NMR of **22** in d$_4$-methanol.

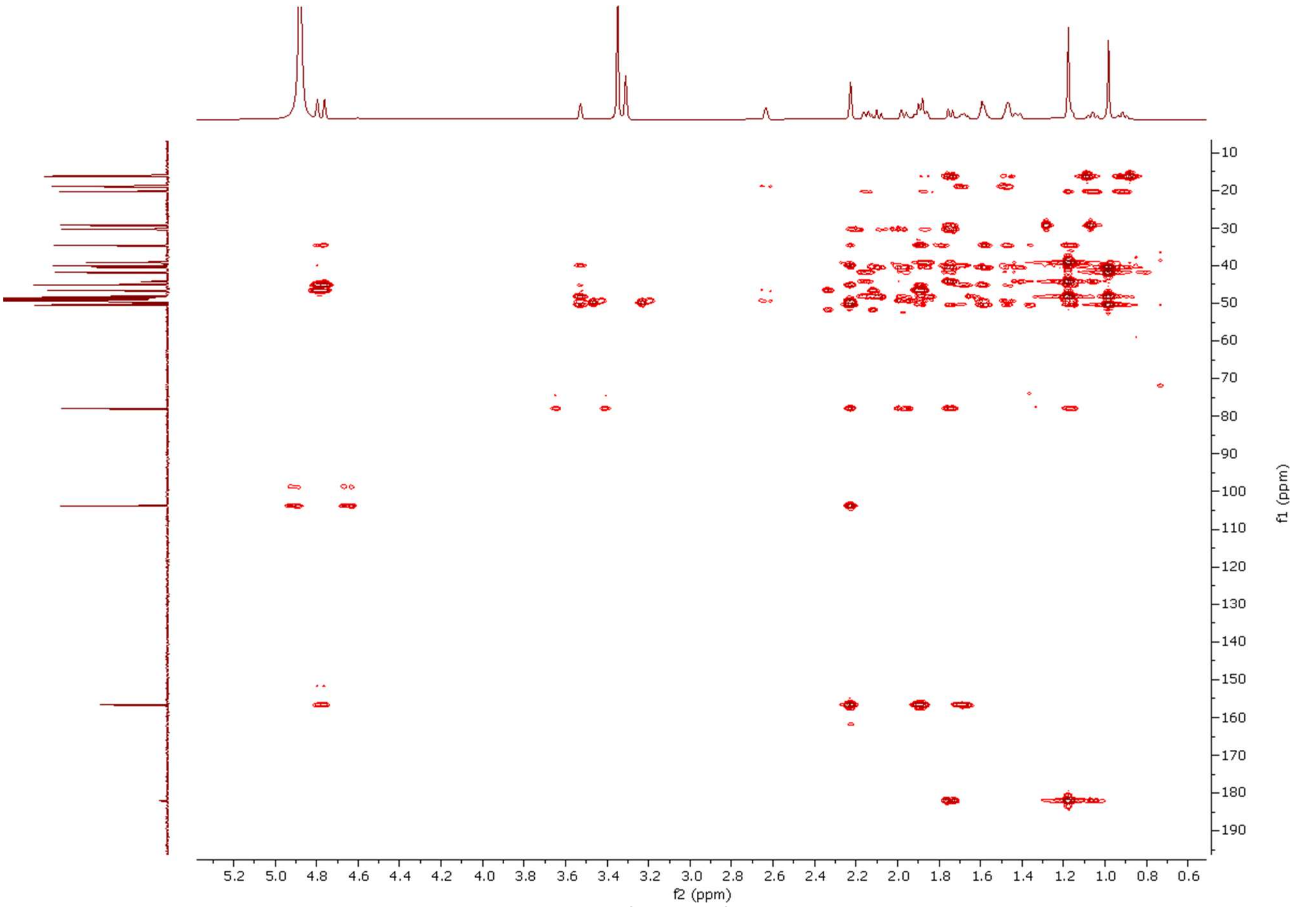

Figure N22.J HMBC NMR of **22** in d$_4$-methanol.

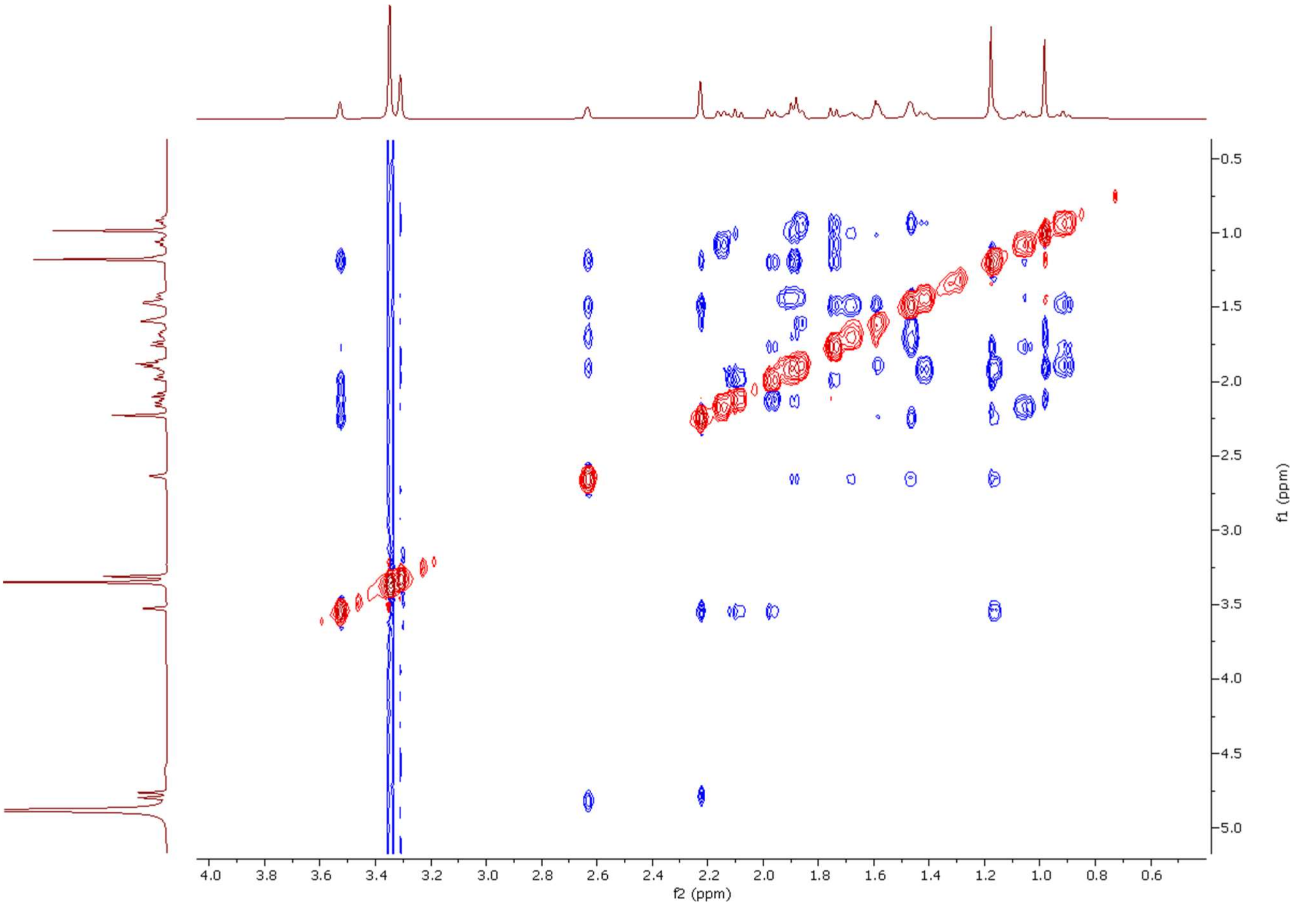

Figure N22.K NOESY NMR of **22** in d_4-methanol at 600 MHz.

6β,7β-Dihydroxy-*ent*-kaurenoic acid (23)

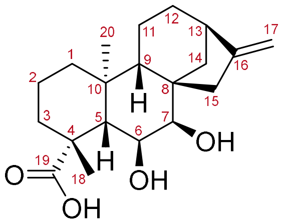

6β,7β-Dihydroxy-*ent*-kaurenoic acid (**23**)
Chemical Formula: $C_{20}H_{30}O_4$
Exact Mass: 334.2144

	**Reference In C_5D_5N [47] (only δ_C reported)**	**Measured in C_5D_5N, 298K**		**Measured in d_4-methanol, 298K**		
**Pos.**	δ_C 50.1 MHz	δ_C 151 MHz	δ_H (*J/Hz*) 400 MHz	δ_C 151 MHz	δ_H (*J/Hz*) 600 MHz	**Selected NOESY corr. to H [a]**
**1**	41.2	41.5	ax 0.97, ddd (13.3, 13.2, 3.9)<br>eq 1.87, overlapped	41.9	ax 0.89, ddd (13.4, 13.3, 4.0)<br>eq 1.86, overlapped	H-5, H-1eq<br>H-1ax
**2**	19.8	20.2	1.50, overlapped<br>2.21, m	20.2	a 1.39, m<br>b 1.83, overlapped	H-2b<br>H-2a
**3**	39.0	40.9	ax 1.24, overlapped<br>eq 2.52, ddd (13.4, 3.5, 3.5)	40.9	ax 1.11, ddd (13.4, 13.4, 4.4)<br>eq 2.13, ddd (13.4, 5.0, 3.5)	
**4**	46.2	44.9	-	45.1	-	-
**5**	52.0	52.4	2.40, d (10.9)	52.6	1.83, d (11.2)	H-6, H_3-18, H-1ax, H-9
**6**	71.8	72.2	ax 4.90, dd (10.9, 2.4)	72.5	ax 4.32, dd (11.2, 2.4)	H_3-20, H-5, H-7
**7**	81.9	82.3	eq 3.95, d (2.4)	82.8	eq 3.46, d (2.4)	H-14a, H_2-15, H-6
**8**	49.0	49.4	-	49.6	-	-
**9**	49.2	49.5	1.82, d (7.6)	49.7	1.43, dd (4.2, 4.2)	H-5
**10**	41.2	41.5	-	42.0	-	-
**11**	18.4	18.8	1.60, overlapped<br>1.69, m	19.1	1.58, m	
**12**	33.9	34.2	1.50, overlapped<br>1.59, overlapped	34.6	ax 1.48, m<br>eq 1.65, ddd (9.7, 2.1, 1.6)	
**13**	46.2	44.4	2.64, overlapped	45.0	2.66, dd (5.0, 5.0)	H-17b
**14**	44.5	39.3	a 1.25, overlapped<br>b 1.91, dd (11.6, 1.6)	39.7	a 1.25, br dd (11.2, 5.0)<br>b 1.90, dd (11.2, 2.1)	H-7
**15**	46.5	46.9	2.65, overlapped	46.6	2.28, m	H-17a, H-7
**16**	155.7	156.1	-	156.1	-	-
**17**	103.6	104.0	4.92, overlapped<br>4.94, overlapped	104.0	a 4.78, br s<br>b 4.82, ddd (3.8, 2.4, 1.2)	H_2-15<br>H-13
**18**	33.3	33.6	1.85, s	33.1	1.45, s	H-5
**19**	181.3	181.8	-	182.2	-	-
**20**	17.2	17.6	1.24, s	17.3	1.00, s	H-6

[a] Key NOESY correlations are shown in blue text.

Yellow highlighted data are possibly swapped assignments in the referenced data. Our assignments are supported by HMBC correlations.

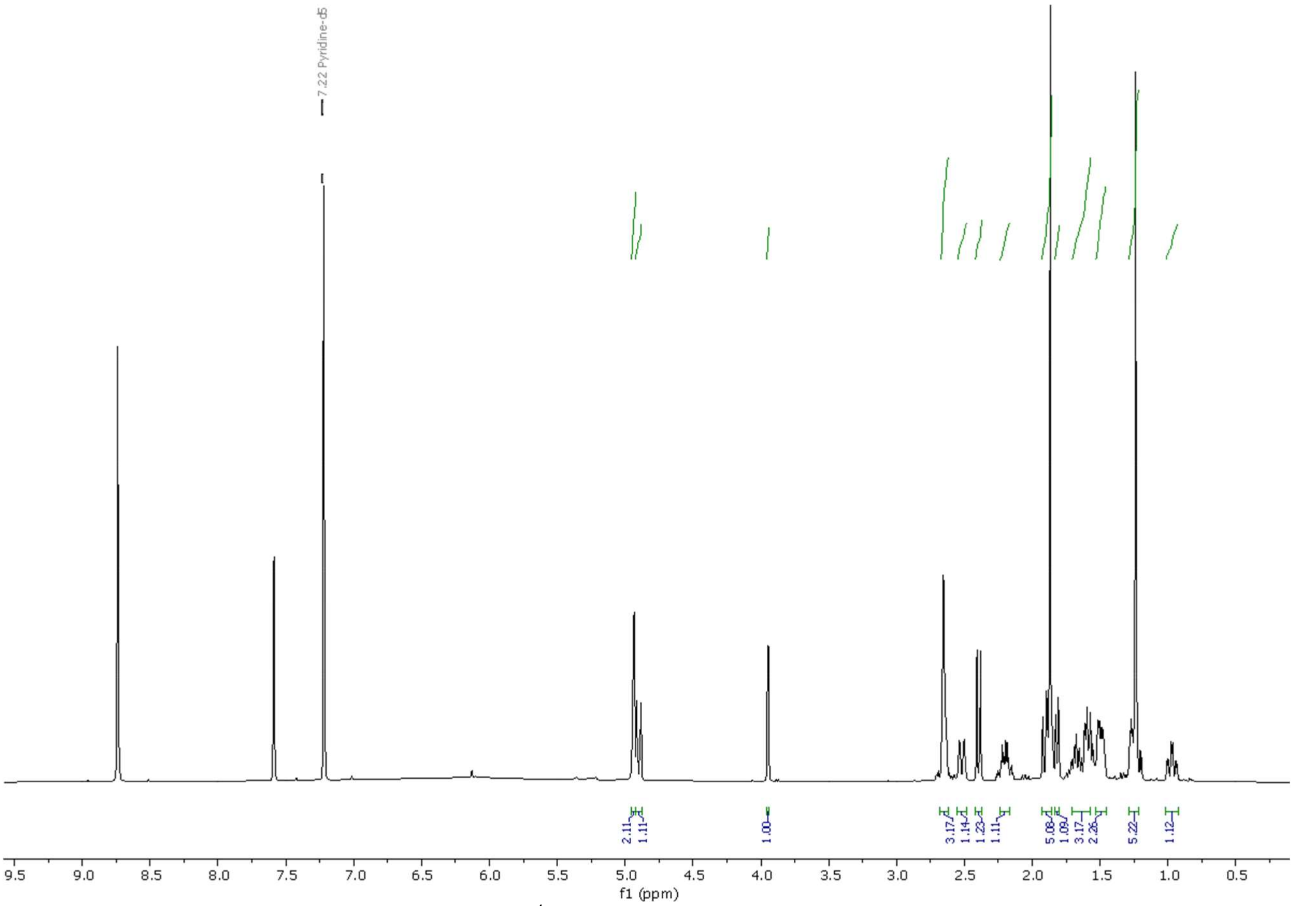

Figure N23.A ^{1}H NMR of **23** in pyridine-d_5 at 400 MHz.

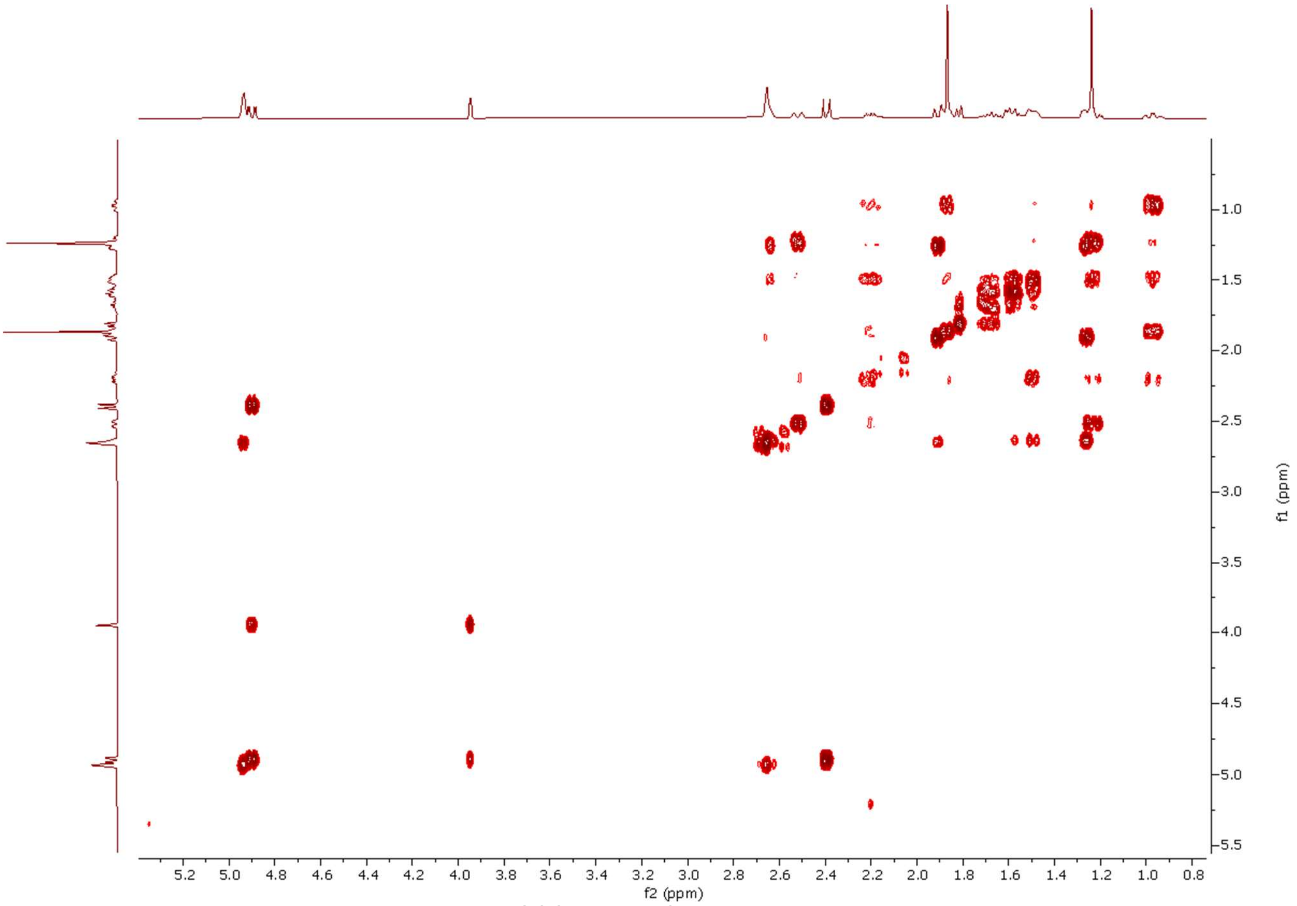

Figure N23.B COSY NMR of **23** in pyridine-d_5 at 600 MHz.

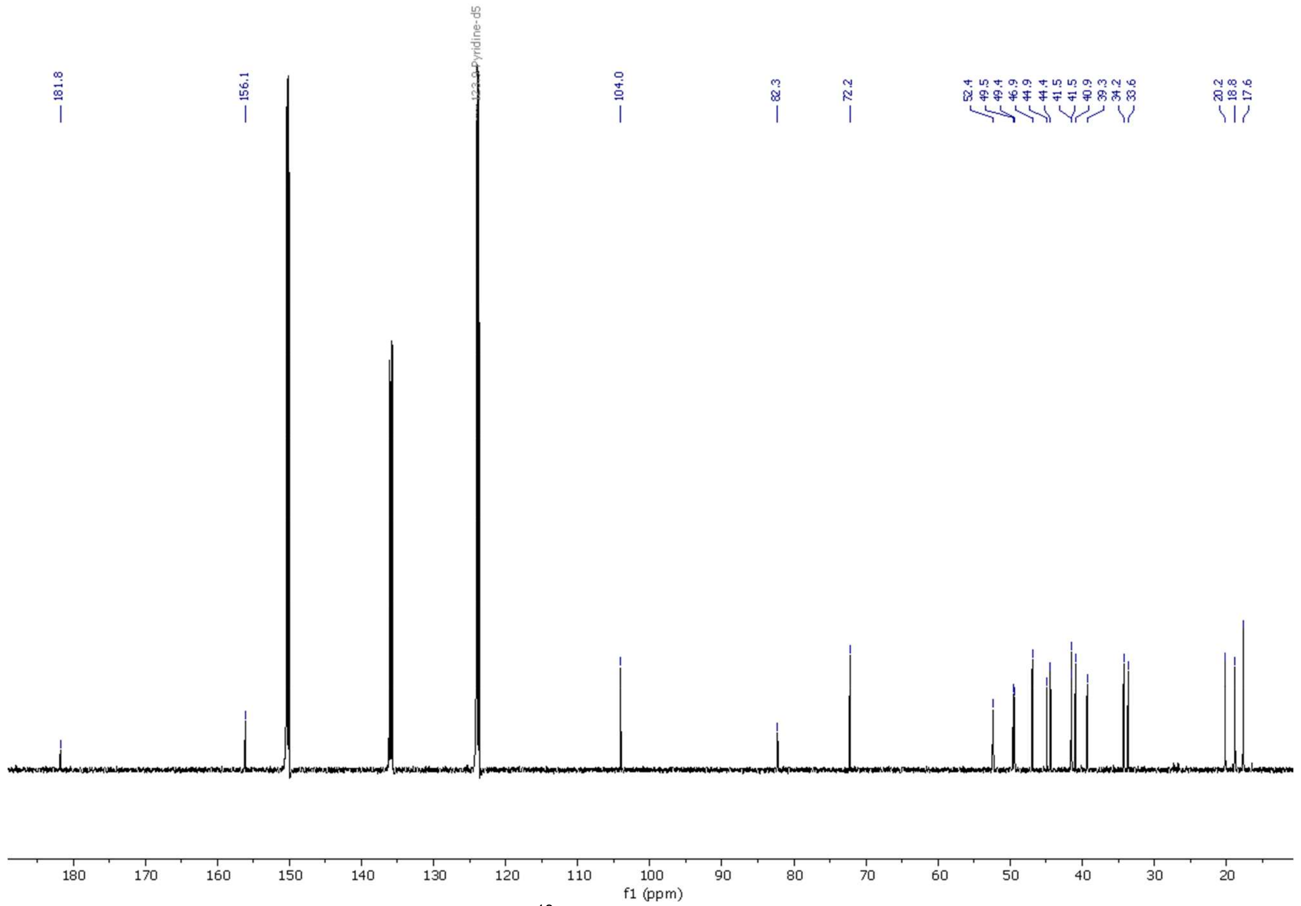

Figure N23.C ^{13}C NMR of **23** in pyridine-d_5 at 151 MHz.

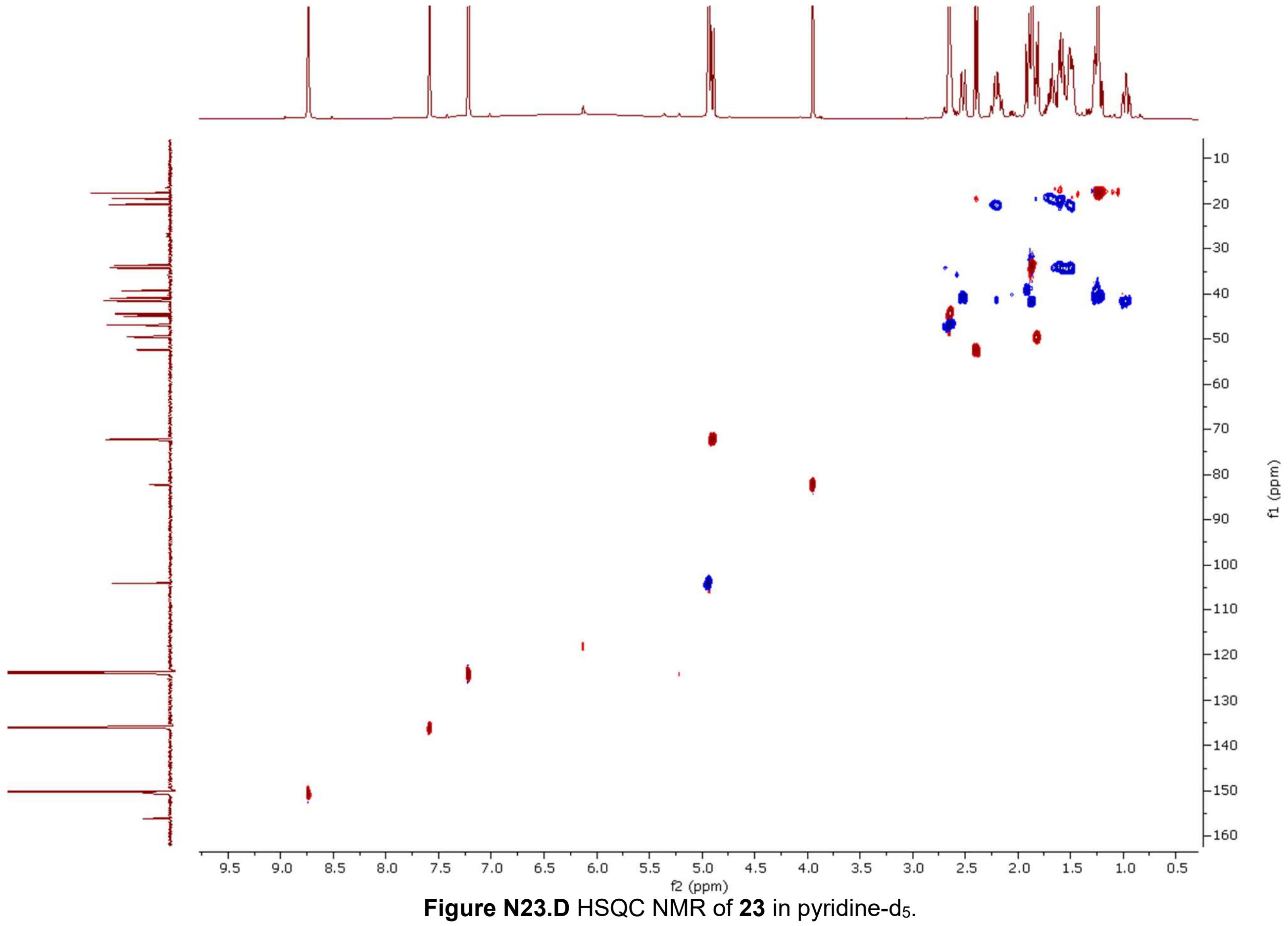

Figure N23.D HSQC NMR of **23** in pyridine-d_5.

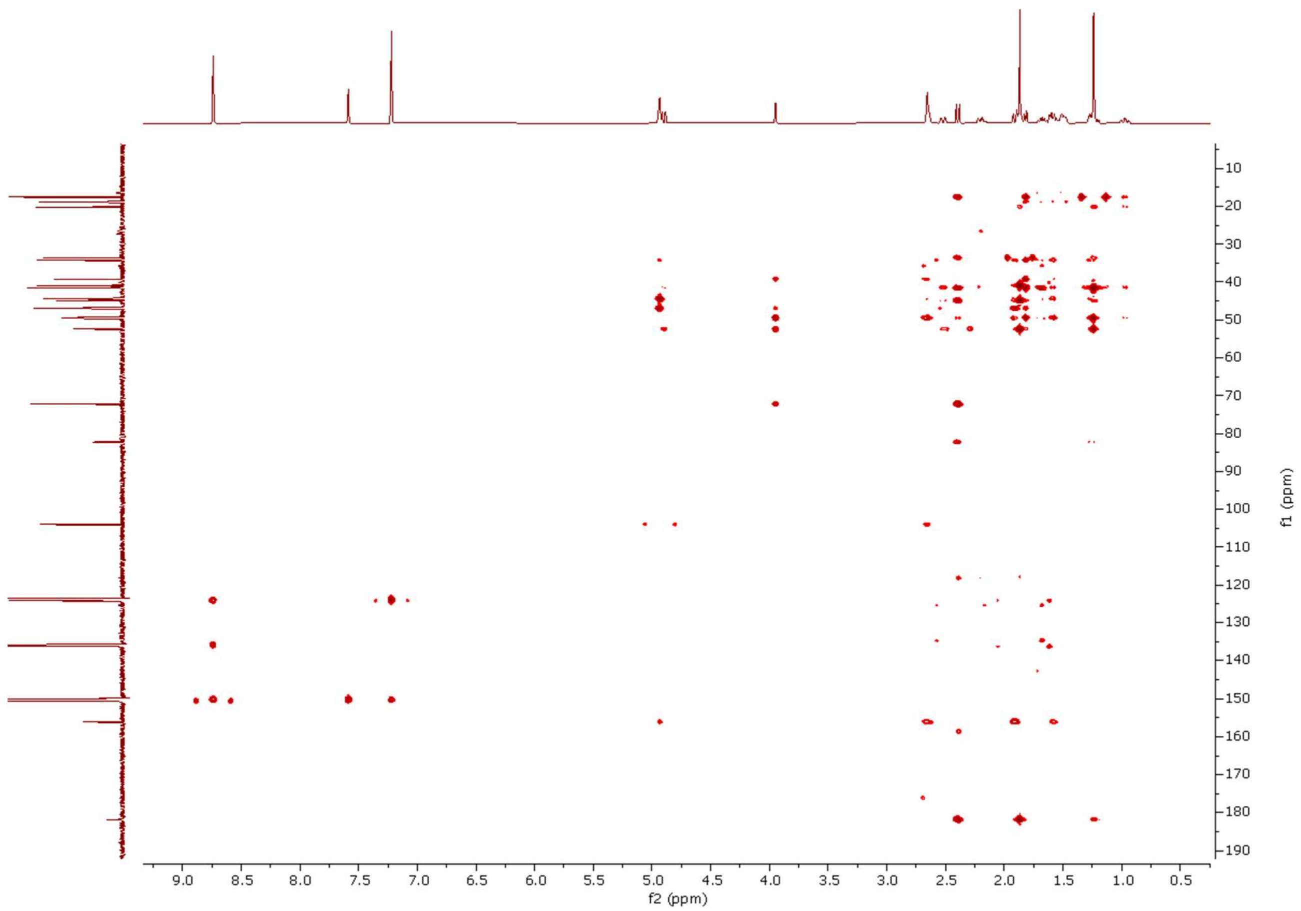

Figure N23.E HMBC NMR of **23** in pyridine-d_5.

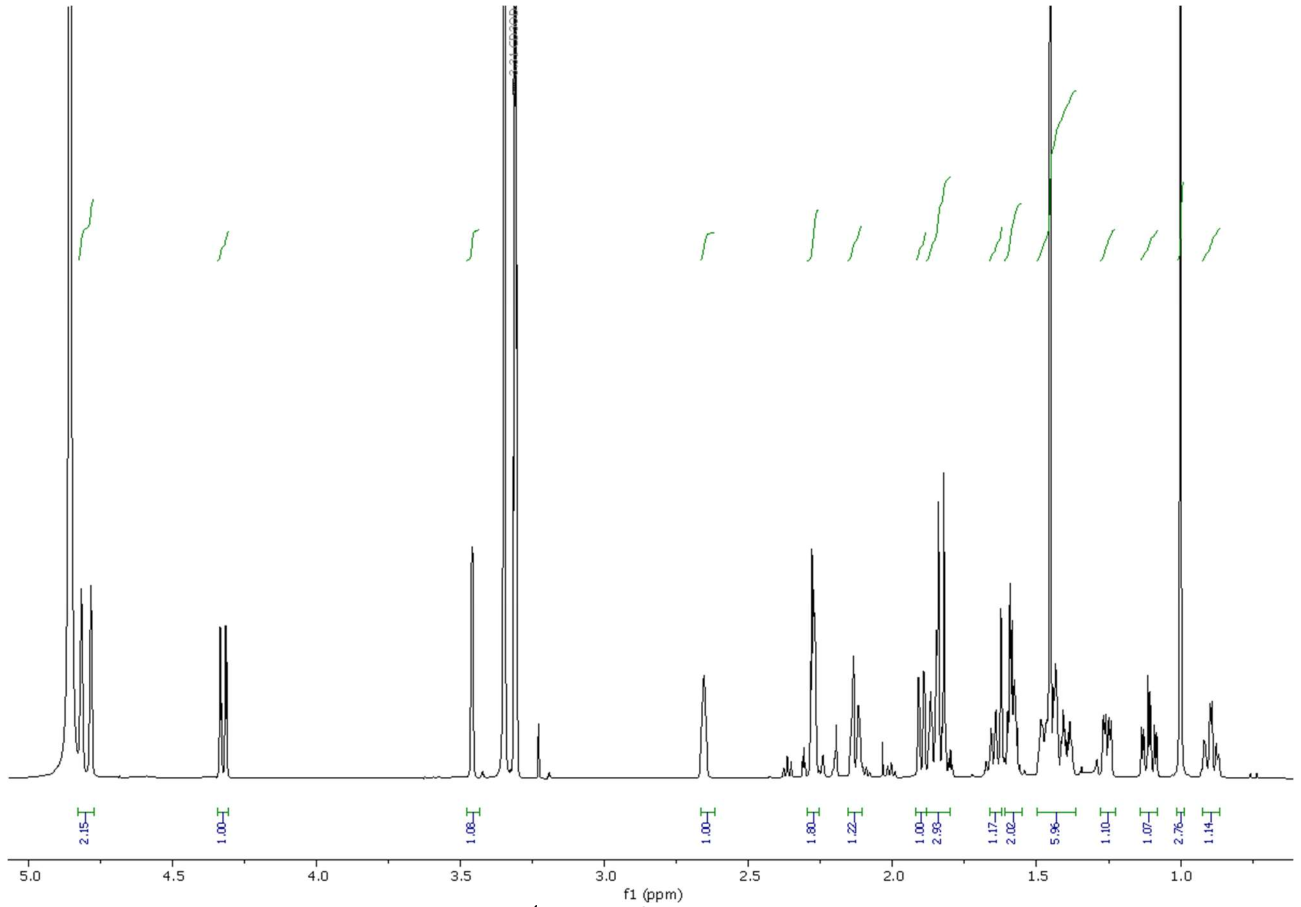

Figure N23.F ^{1}H NMR of **23** in d_4-methanol at 600 MHz.

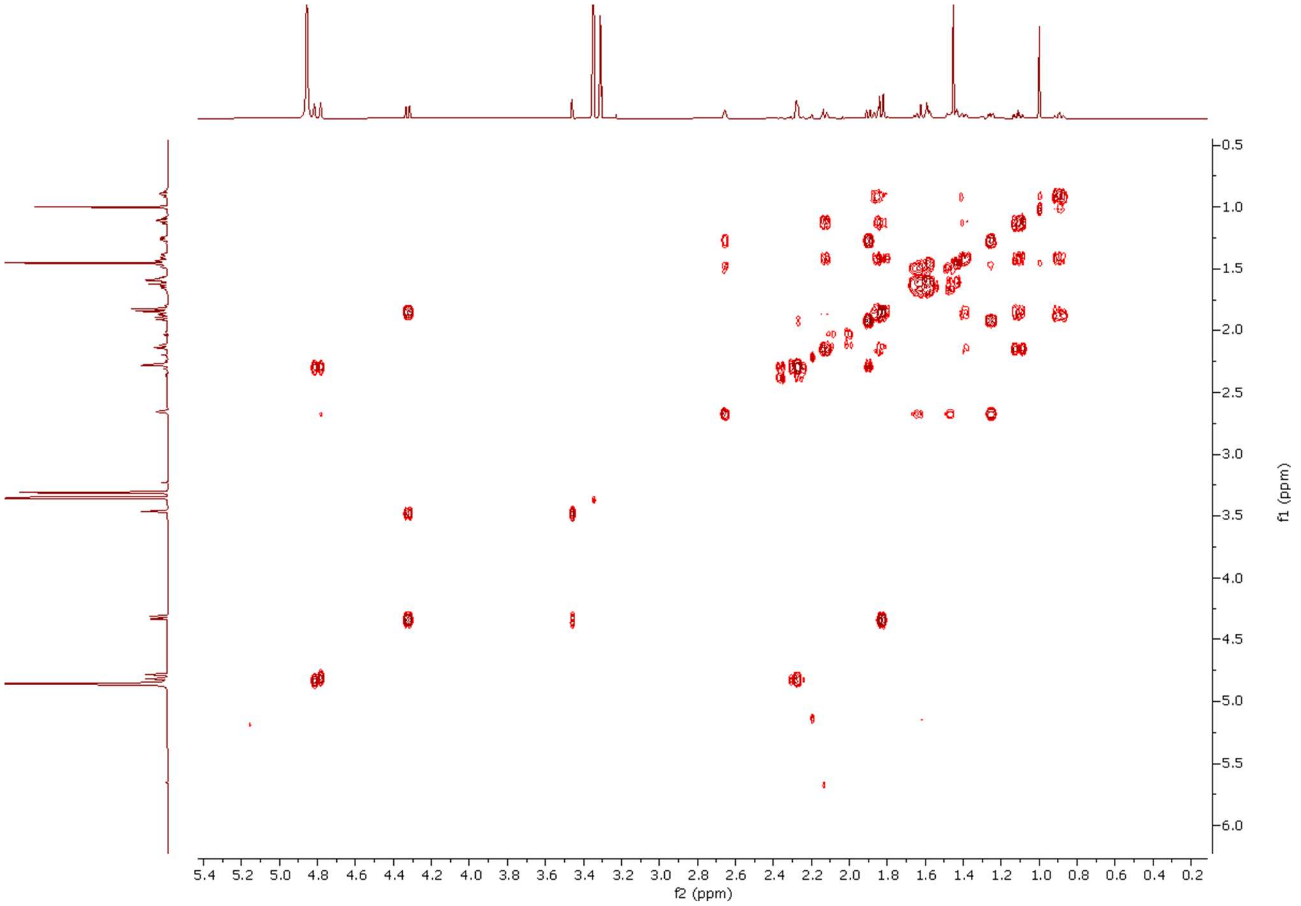

Figure N23.G COSY NMR of **23** in d4-methanol at 600 MHz.

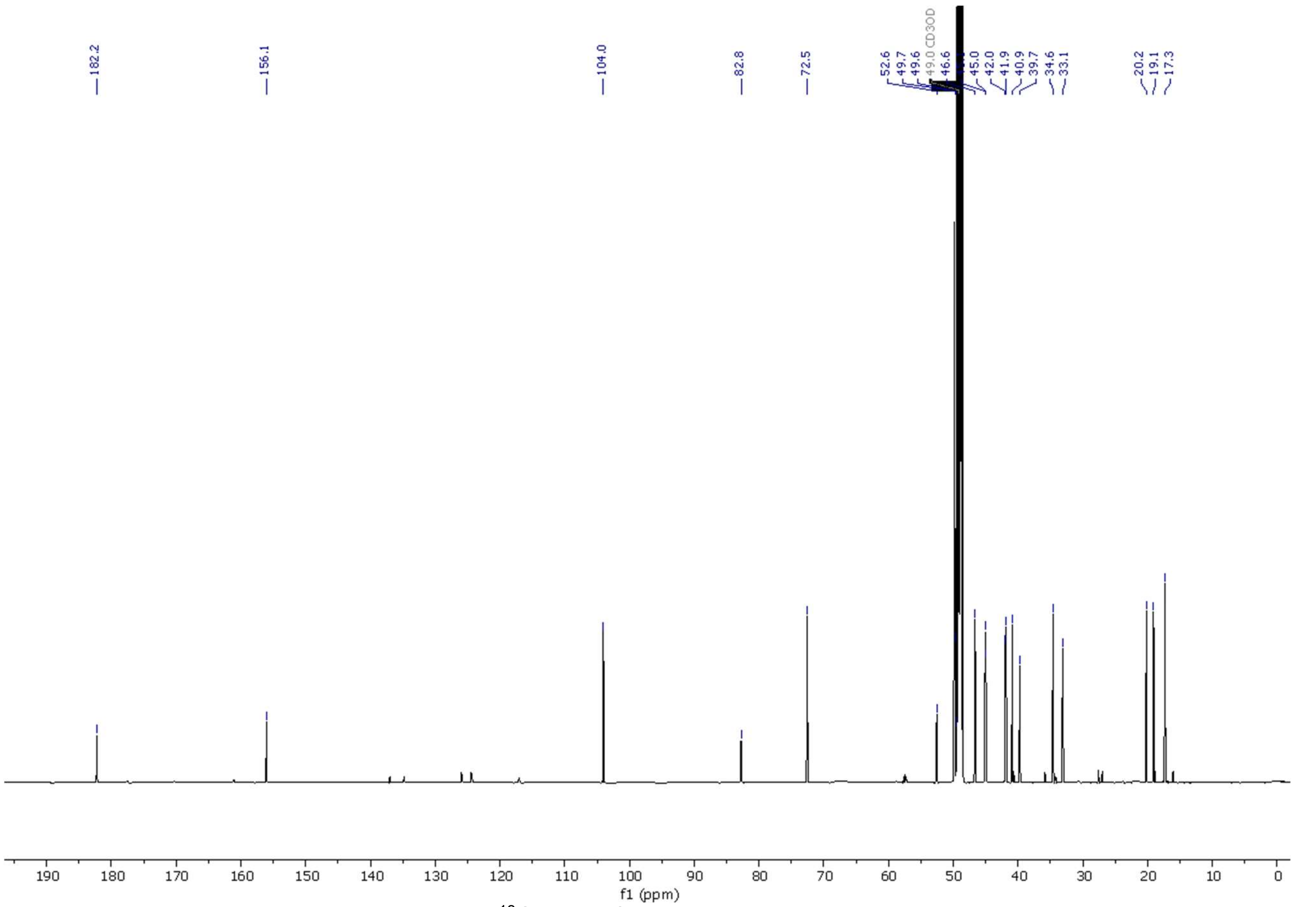

Figure N23.H ^{13}C NMR of **23** in d4-methanol at 151 MHz.

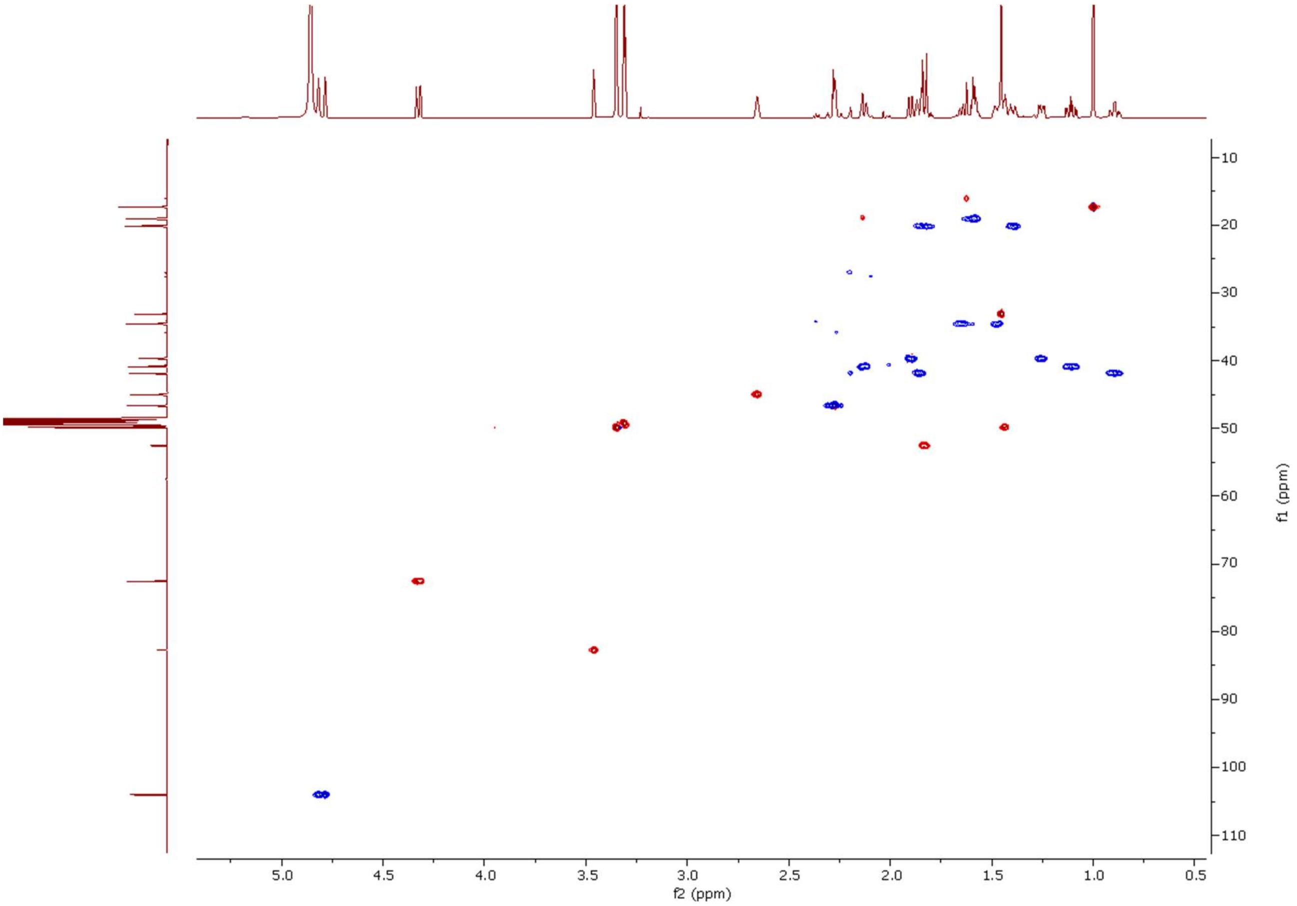

Figure N23.I HSQC NMR of **23** in d_4-methanol.

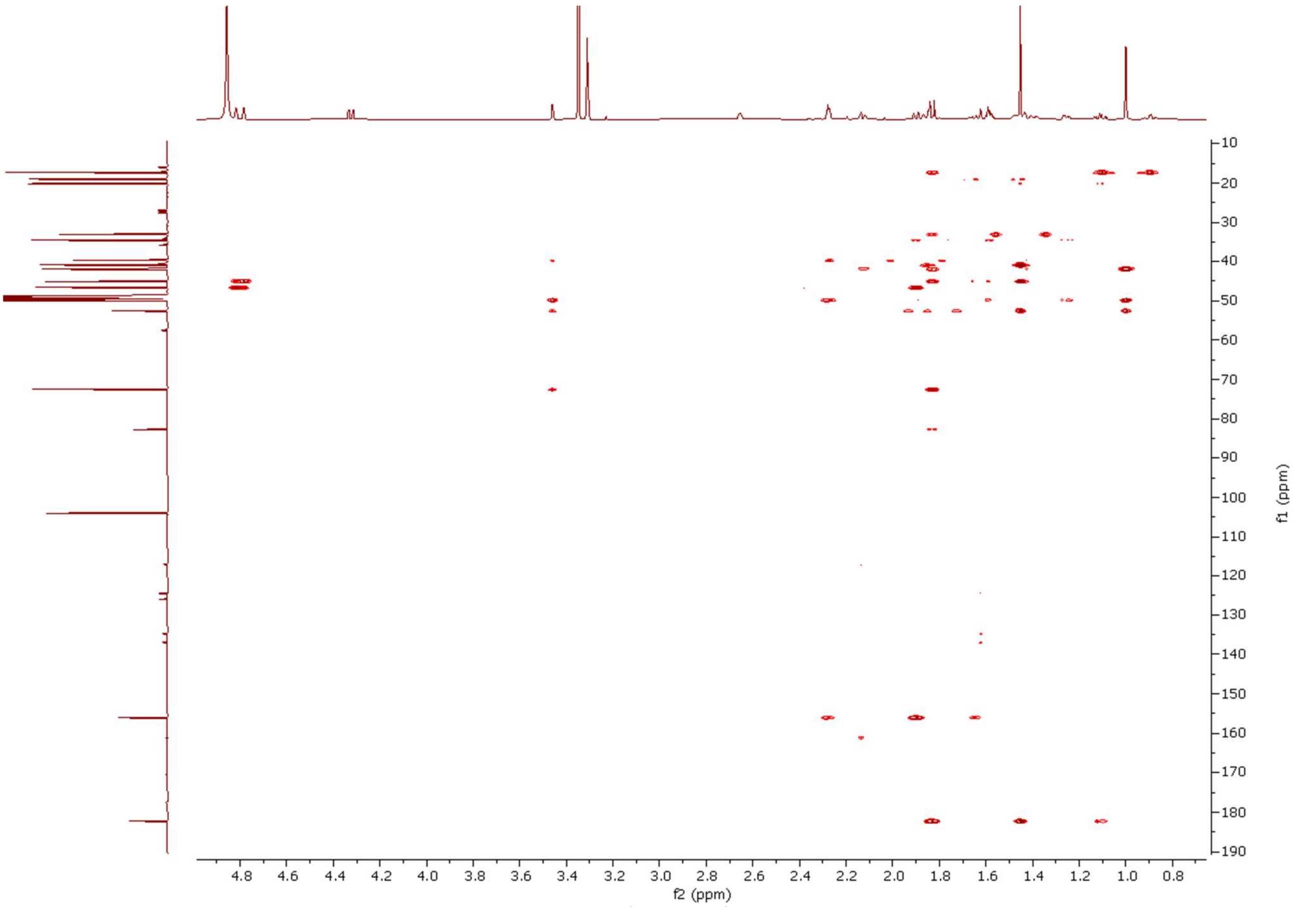

Figure N23.J HMBC NMR of **23** in d_4-methanol.

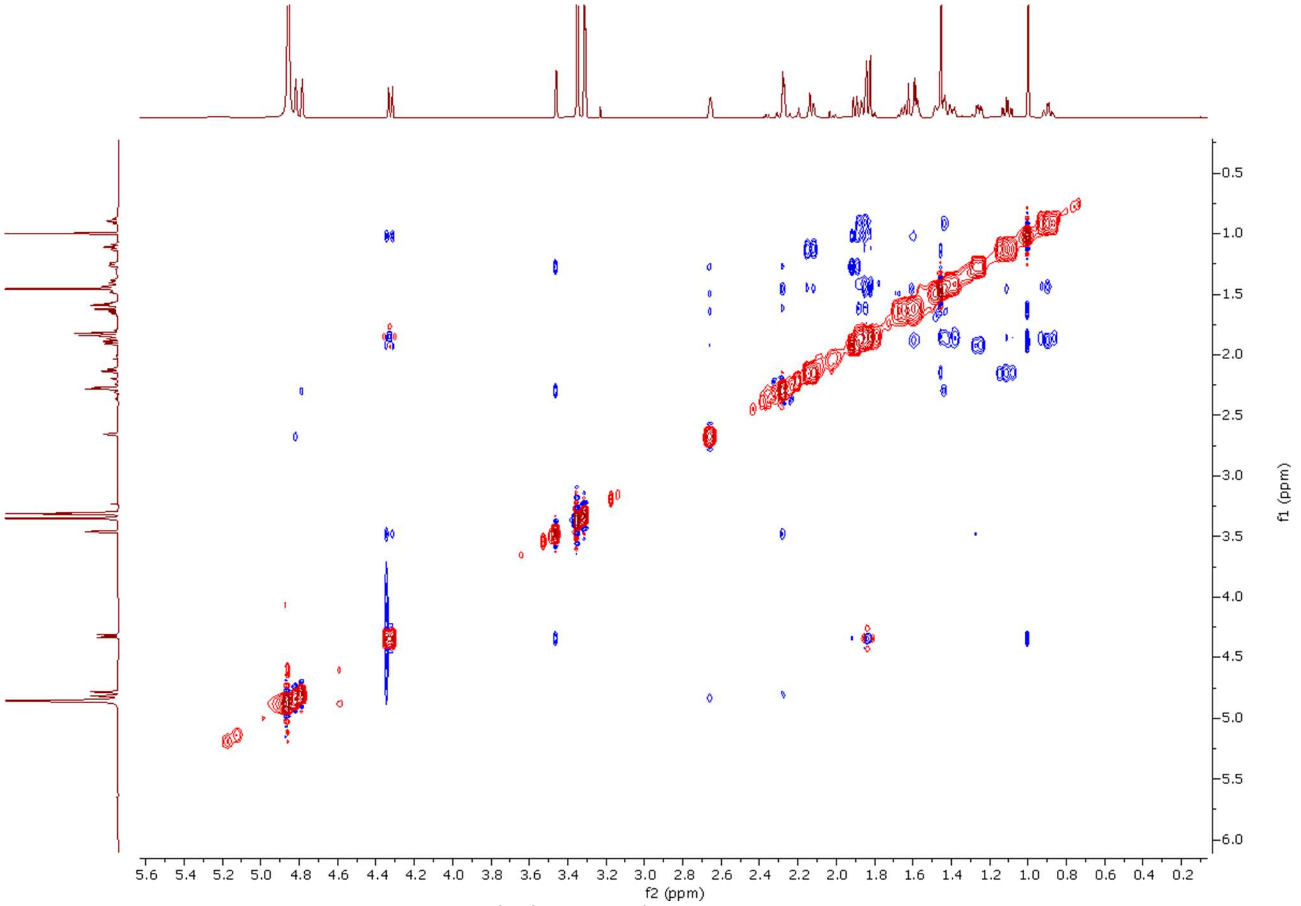

Figure N23.K NOESY NMR of **23** in d_4-methanol at 400 MHz.

Fujenoic acid (24)

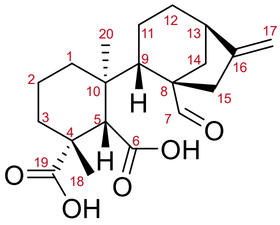

Fujenoic acid (**24**)
Chemical Formula: $C_{20}H_{28}O_5$
Exact Mass: 348.1937

	Reference in C_5D_5N [42] (only δ_C reported)	Measured in C_5D_5N, 298K		Measured in DMSO-d$_6$, 298K	
**Pos.**	δ_C 50.1 MHz	δ_C 151 MHz	δ_H (J/Hz) 600 MHz	δ_C 151 MHz	δ_H (J/Hz) 600 MHz
**1**	37.9	37.3 ^{br}	1.67, overlapped	35.9	1.53, overlapped
**2**	20.0	20.4	1.56, overlapped<br>2.13, overlapped	18.9	1.37, m<br>1.71, overlapped
**3**	37.1^{br}	38.6	1.14, br s<br>2.66, br s	36.3	1.02, overlapped<br>2.00, overlapped
**4**	45.1 ^{br}	45.7 ^{br}	-	43.8	-
**5**	60.6 ^{br}	62.0 ^{br}	3.06, br s	58.2 ^{HMBC}	2.46, br s
**6**	177.3 ^{br}	178.3 ^{br}	-	174.2	-
**7**	204.9 ^{br}	205.3	9.97, s	204.7	9.69, s
**8**	59.3 ^{br}	60.0 ^{br}	-	58.9	-
**9**	48.4 ^{br}	48.5 ^{br}	2.79, br s	47.3	2.28, br d (6.4)
**10**	43.0	43.5 ^{br}	-	41.9	-
**11**	19.8	20.4	1.93, overlapped	19.2	1.67, overlapped
**12**	32.5 ^{br}	33.6	1.52, overlapped<br>1.75, overlapped	32.3	1.45, m<br>1.70, overlapped
**13**	44.4	44.8	2.74, br s	43.4	2.74, br s
**14**	33.1	32.7 ^{br}	1.70, overlapped<br>2.05, overlapped	31.6	1.57, overlapped<br>1.75, overlapped
**15**	43.7	43.9 ^{br}	2.08, overlapped<br>2.26, d (16.8)	42.9	2.04, br d (17.1)<br>2.33, d (17.1)
**16**	152.6	153.0	-	151.8	-
**17**	104.5	104.8	b 4.75, br s<br>a 4.91, br s	104.4	b 4.76, br s<br>a 4.87, br s
**18**	30.4	30.6	1.62, s	29.9	1.22, s
**19**	179.5 ^{br}	180.4 ^{br}	-	177.7	-
**20**	21.9	22.7 ^{br}	1.54, s	20.9	1.02, s

^{HMBC} = detected based on HMBC.

^{br} = broad signal. NMR spectra recorded in C_5D_5N showed broad signals, and several overlapping protons were assigned using COSY correlations. Similar signal broadening is also observed in the published reference data, likely due to partial ring opening.

The spectra acquired in DMSO-d$_6$ displayed improved resolution. NMR data of the methylated derivative are also provided (see compound **24a**).

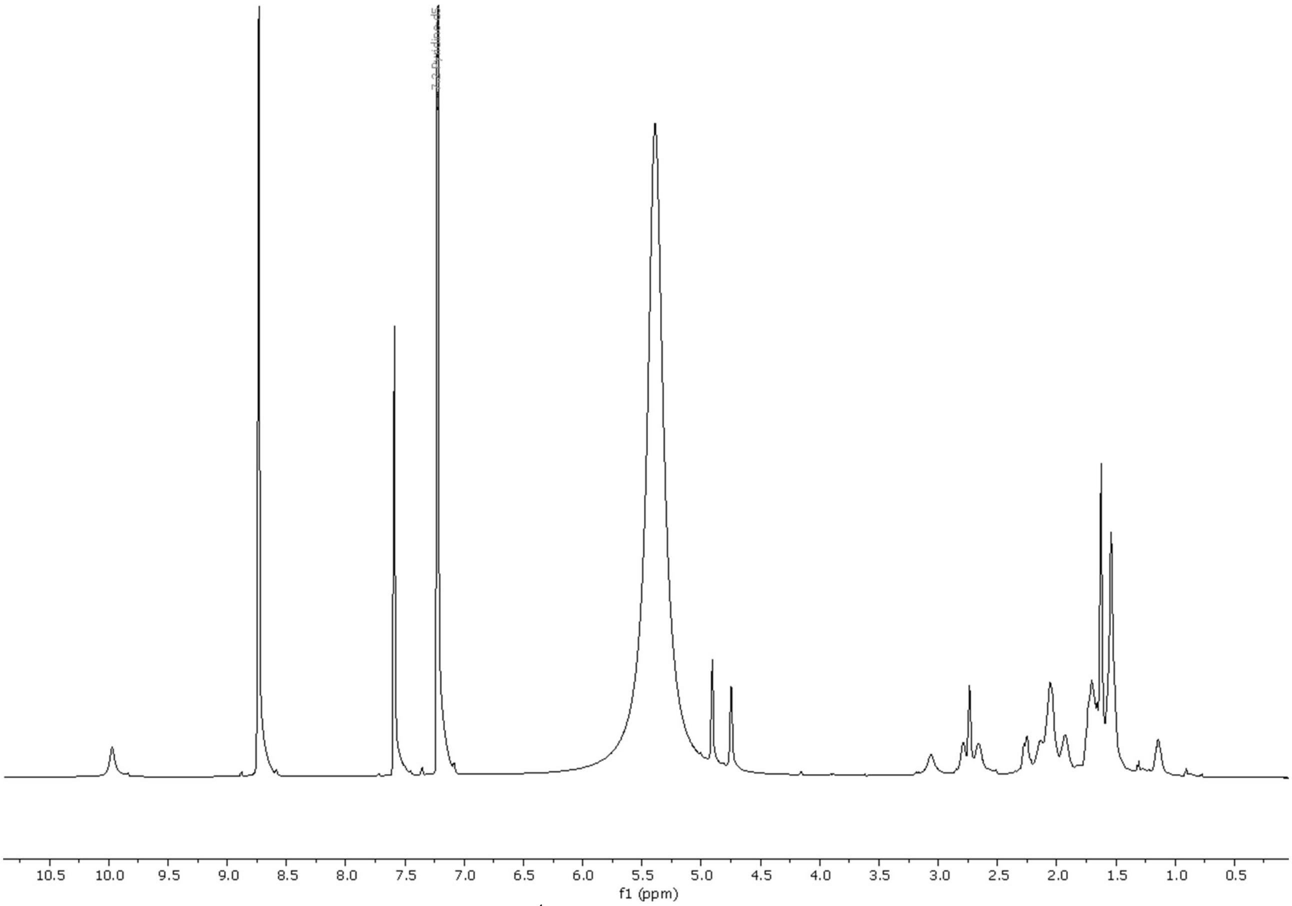

Figure N24.A ^{1}H NMR of **24** in pyridine-d_5 at 600 MHz.

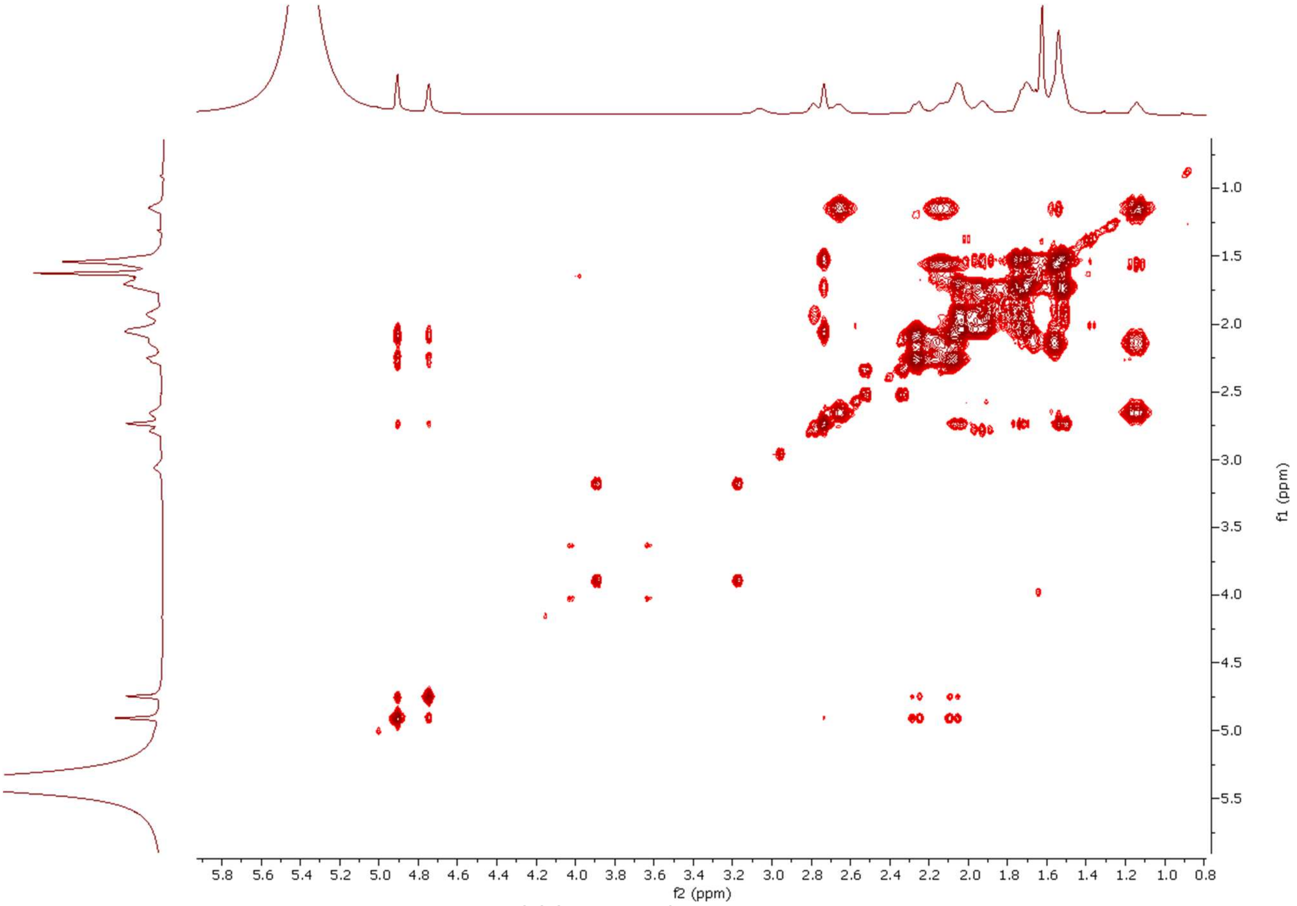

Figure N24.B COSY NMR of **24** in pyridine-d_5 at 600 MHz.

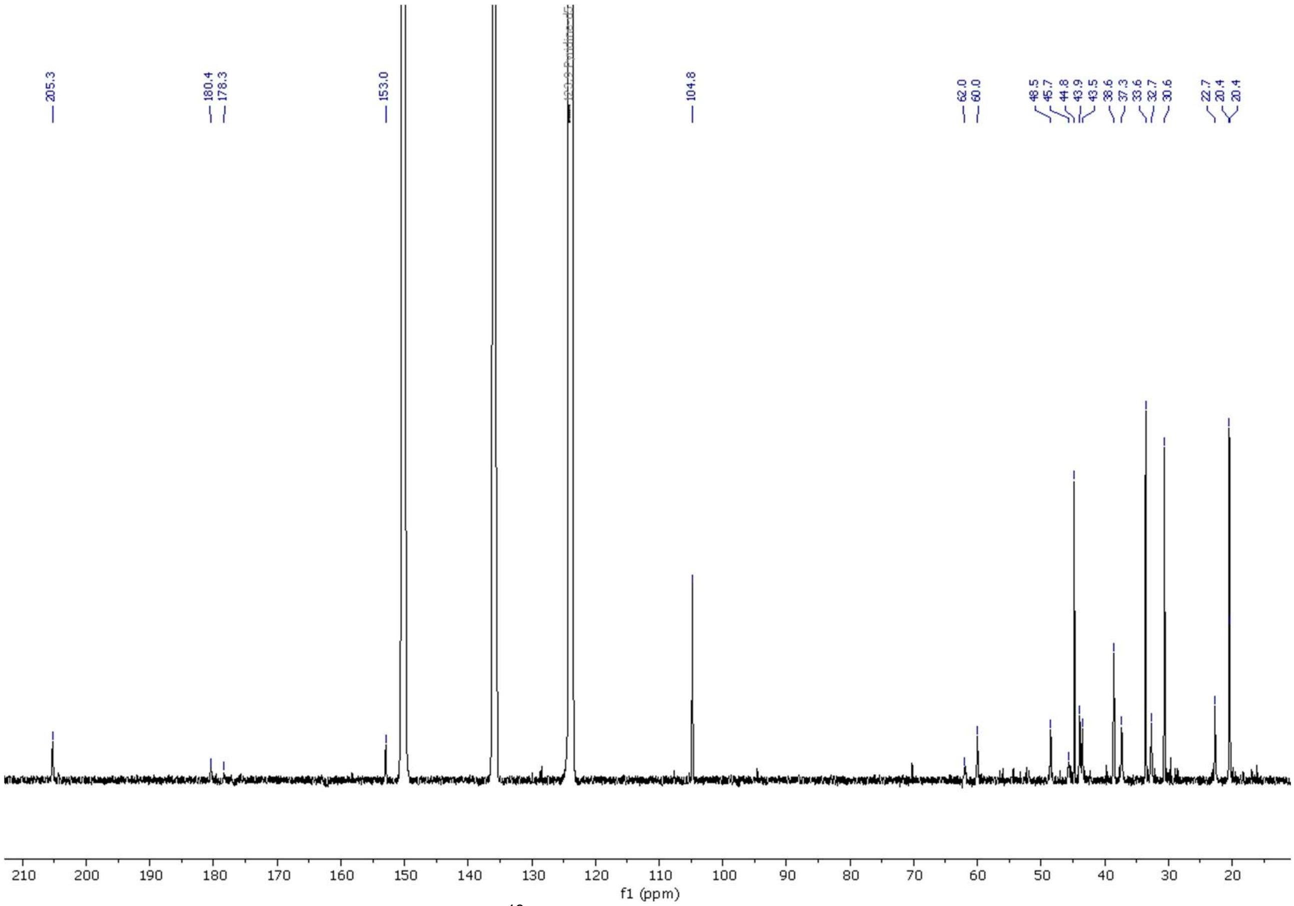

Figure N24.C ^{13}C NMR of **24** in pyridine-d_5 at 151 MHz.

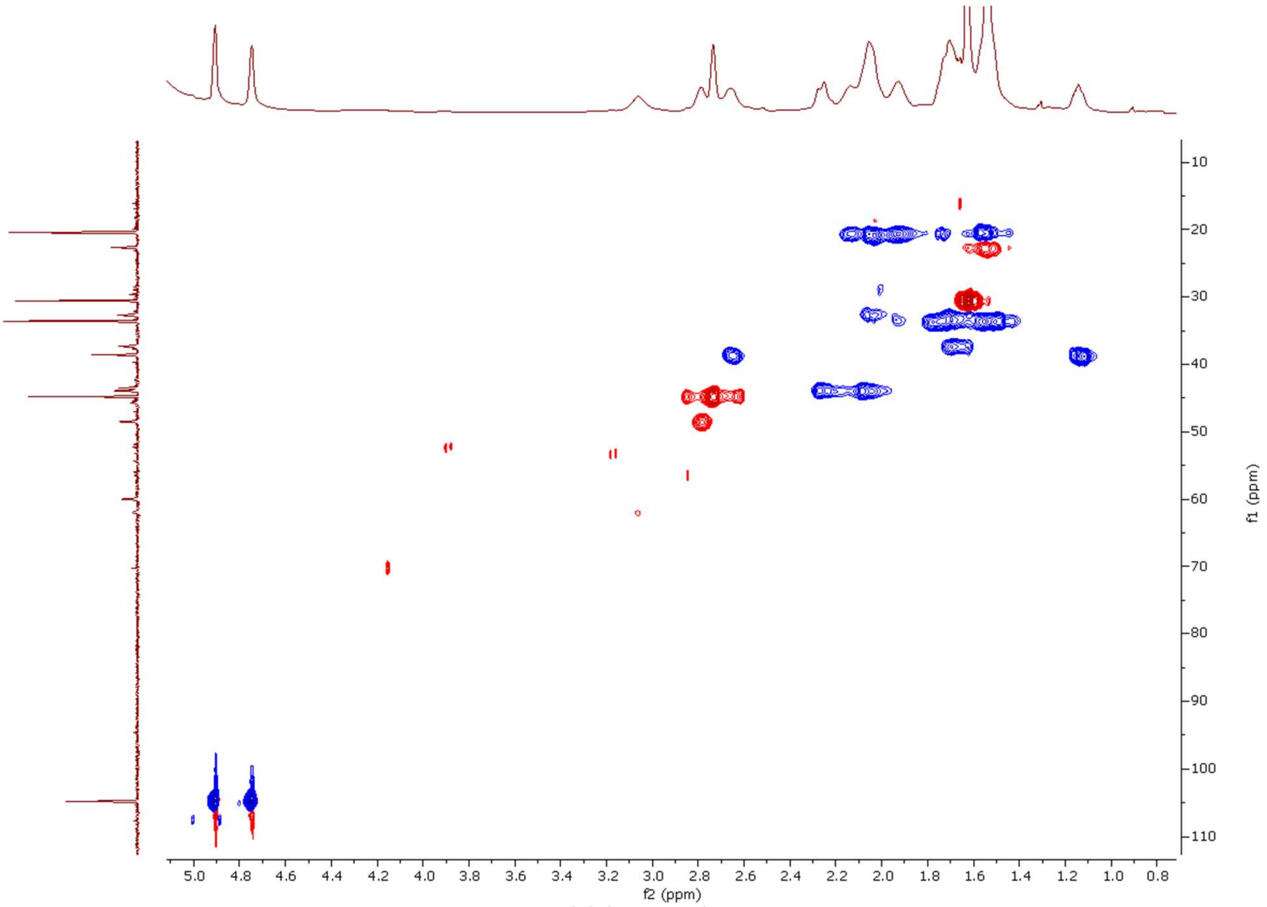

Figure N24.D HSQC NMR of **24** in pyridine-d_5.

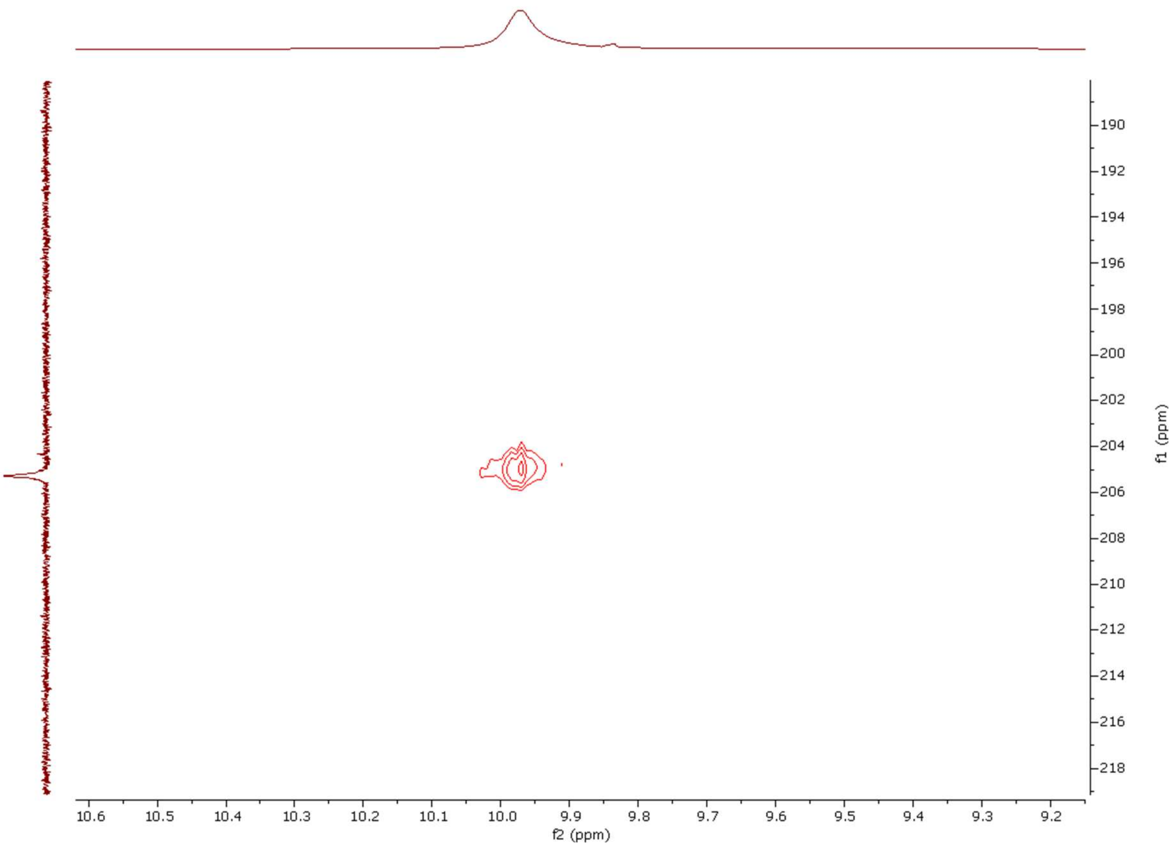

Figure N24.E HSQC (zoomed in) NMR of **24** in pyridine-d5.

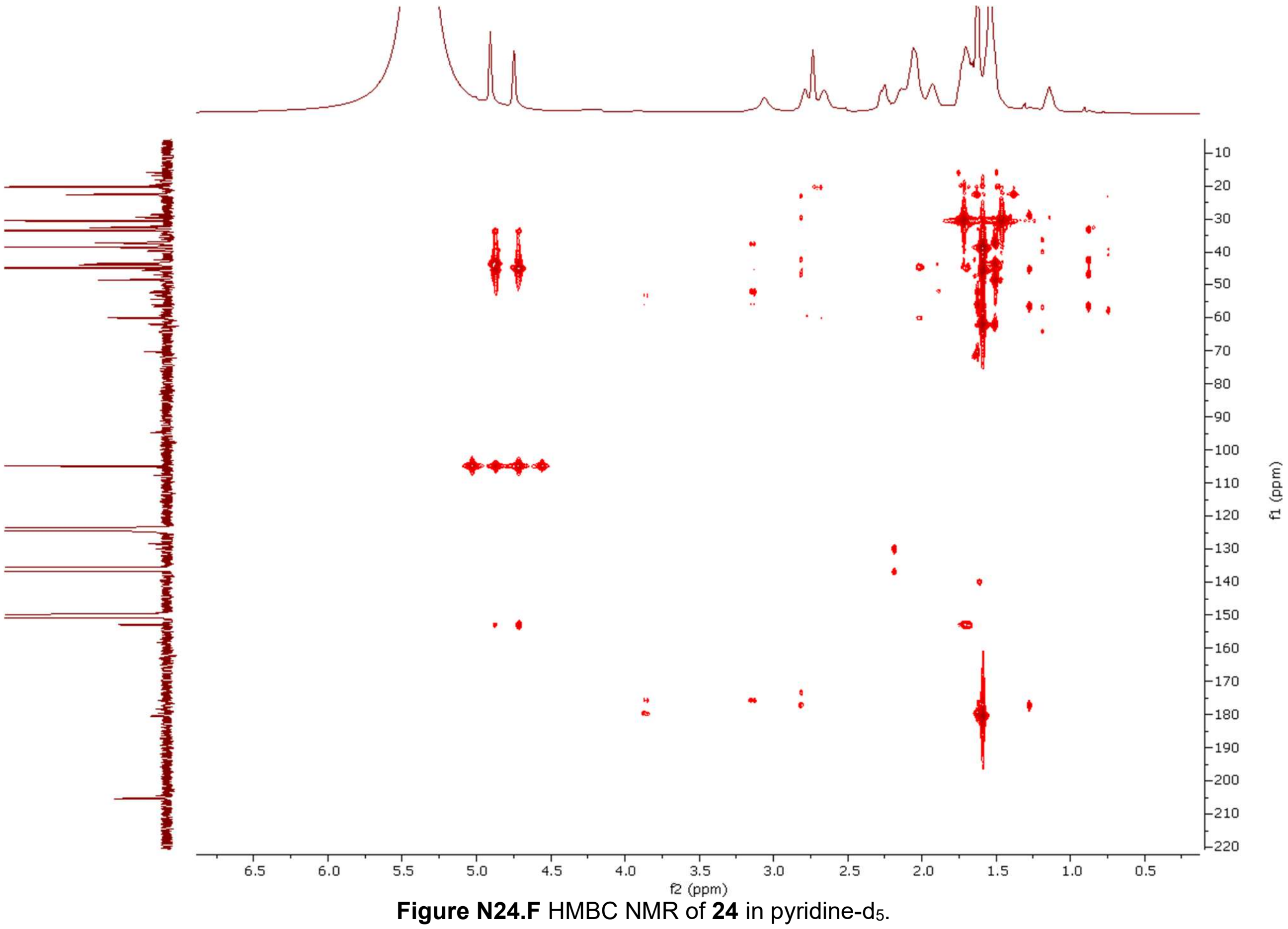

Figure N24.F HMBC NMR of **24** in pyridine-d5.

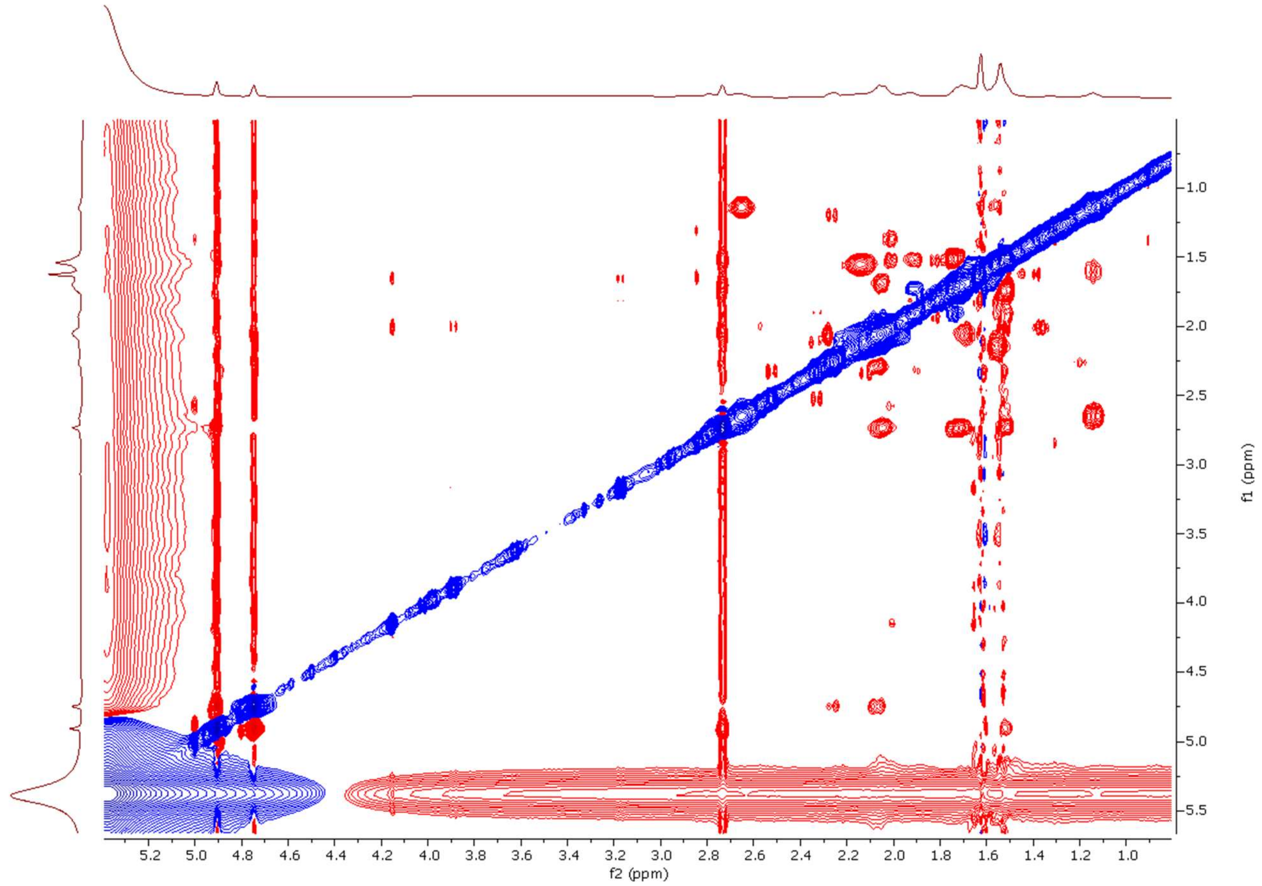

Figure N24.G NOESY NMR of **24** in pyridine-d_5 at 600 MHz.

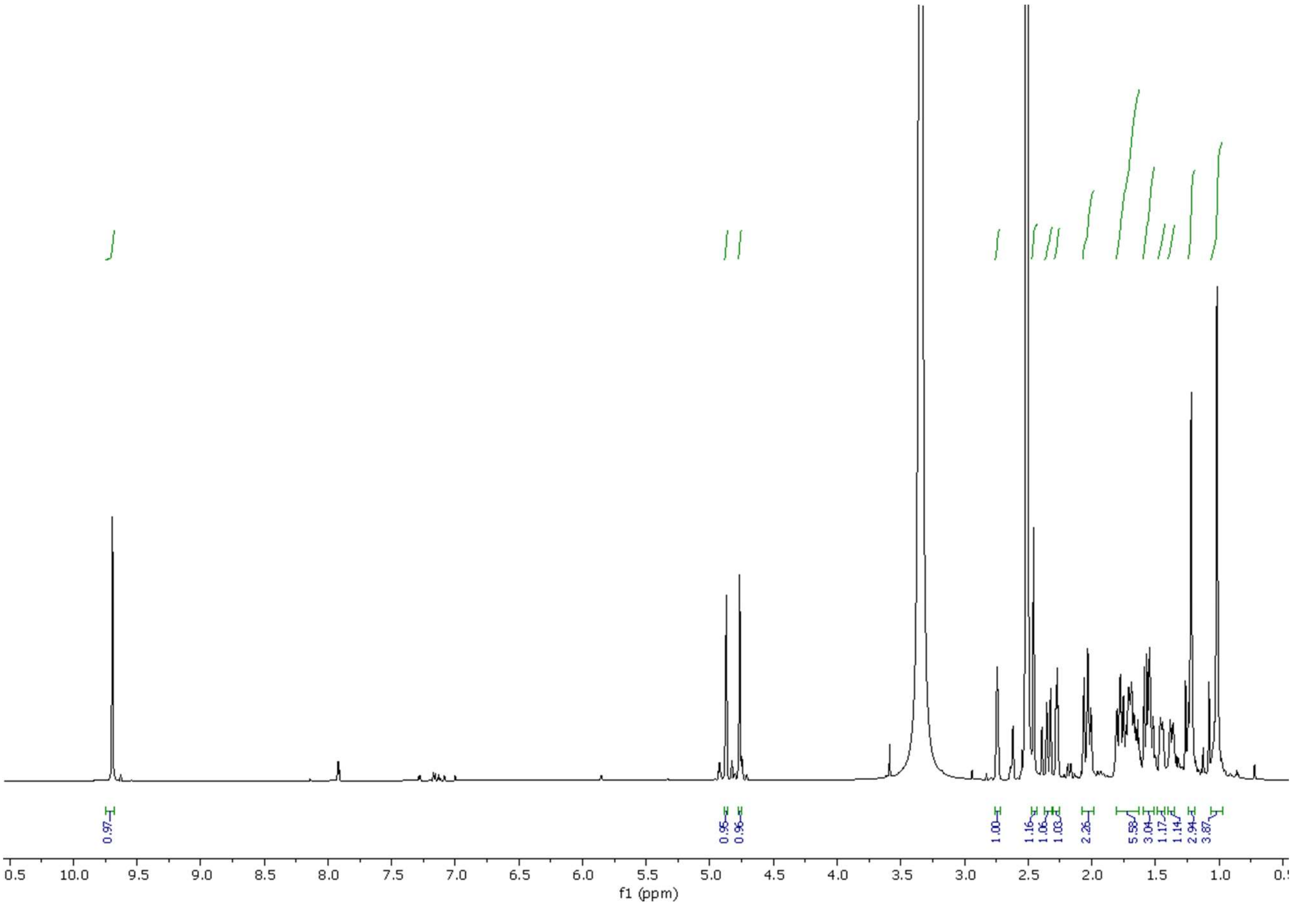

Figure N24.H ^{1}H NMR of **24** in DMSO-d_6 at 600 MHz.

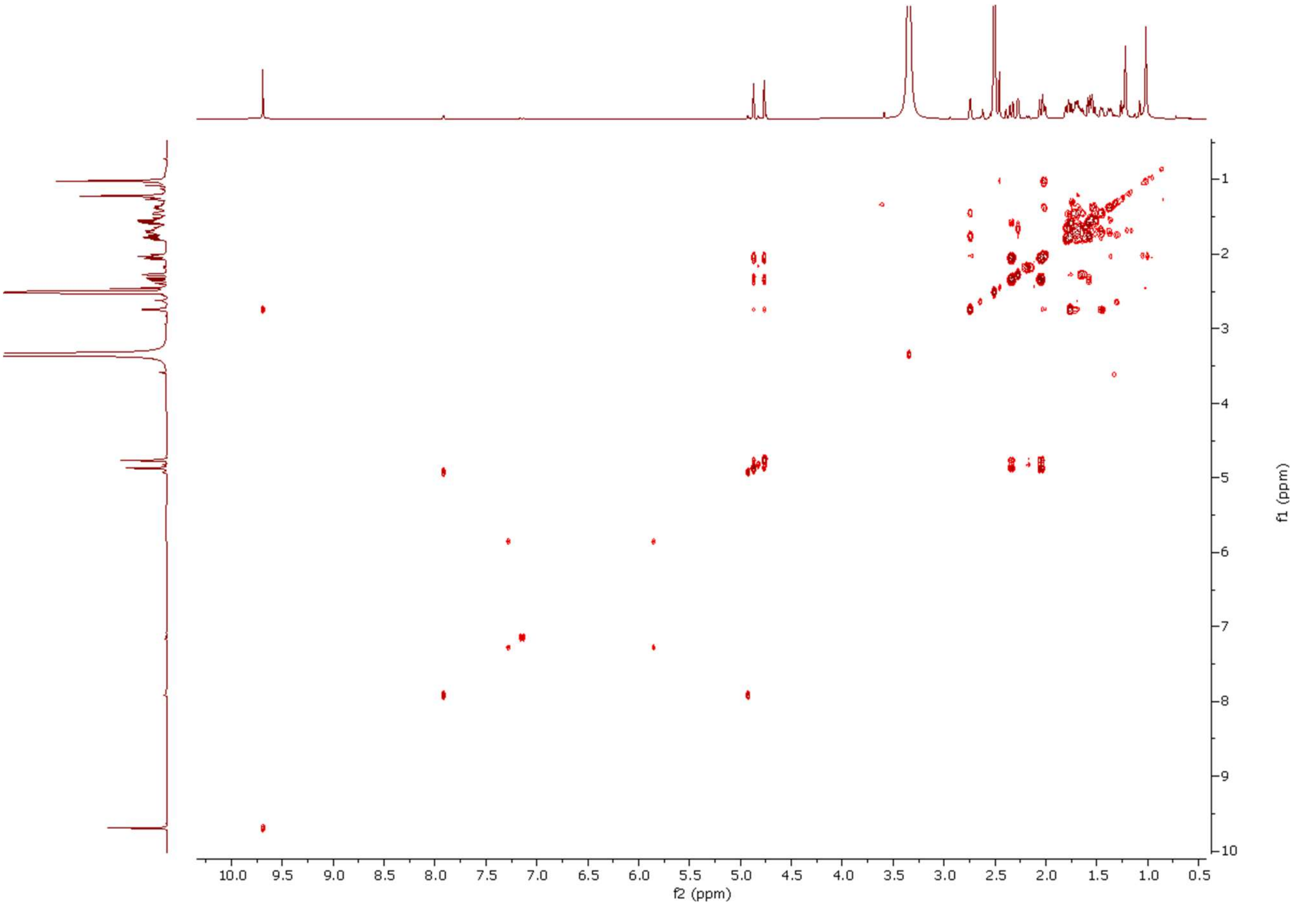

Figure N24.I COSY NMR of **24** in DMSO-d_6 at 600 MHz.

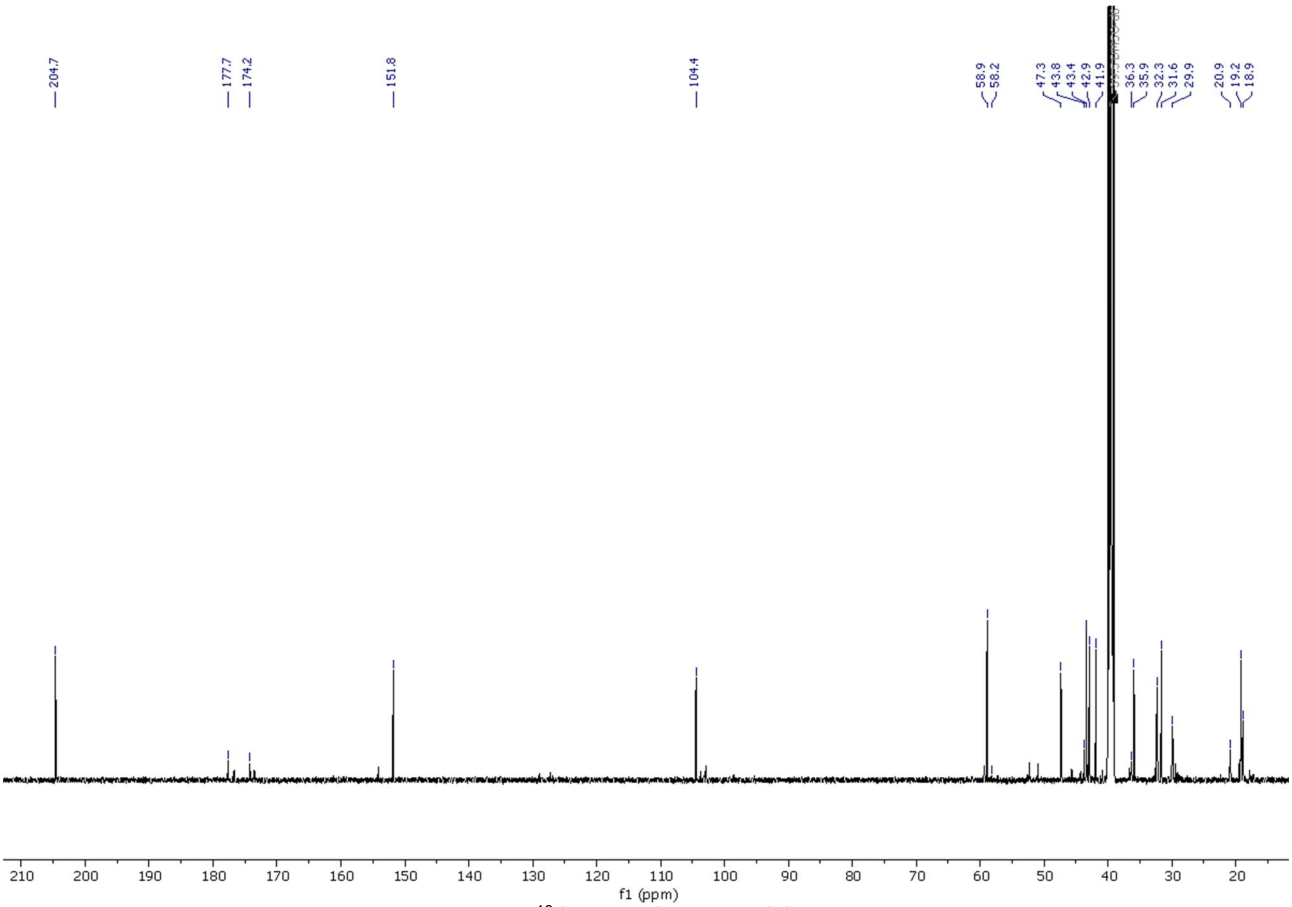

Figure N24.J ^{13}C NMR of **24** in DMSO-d_6 at 151 MHz.

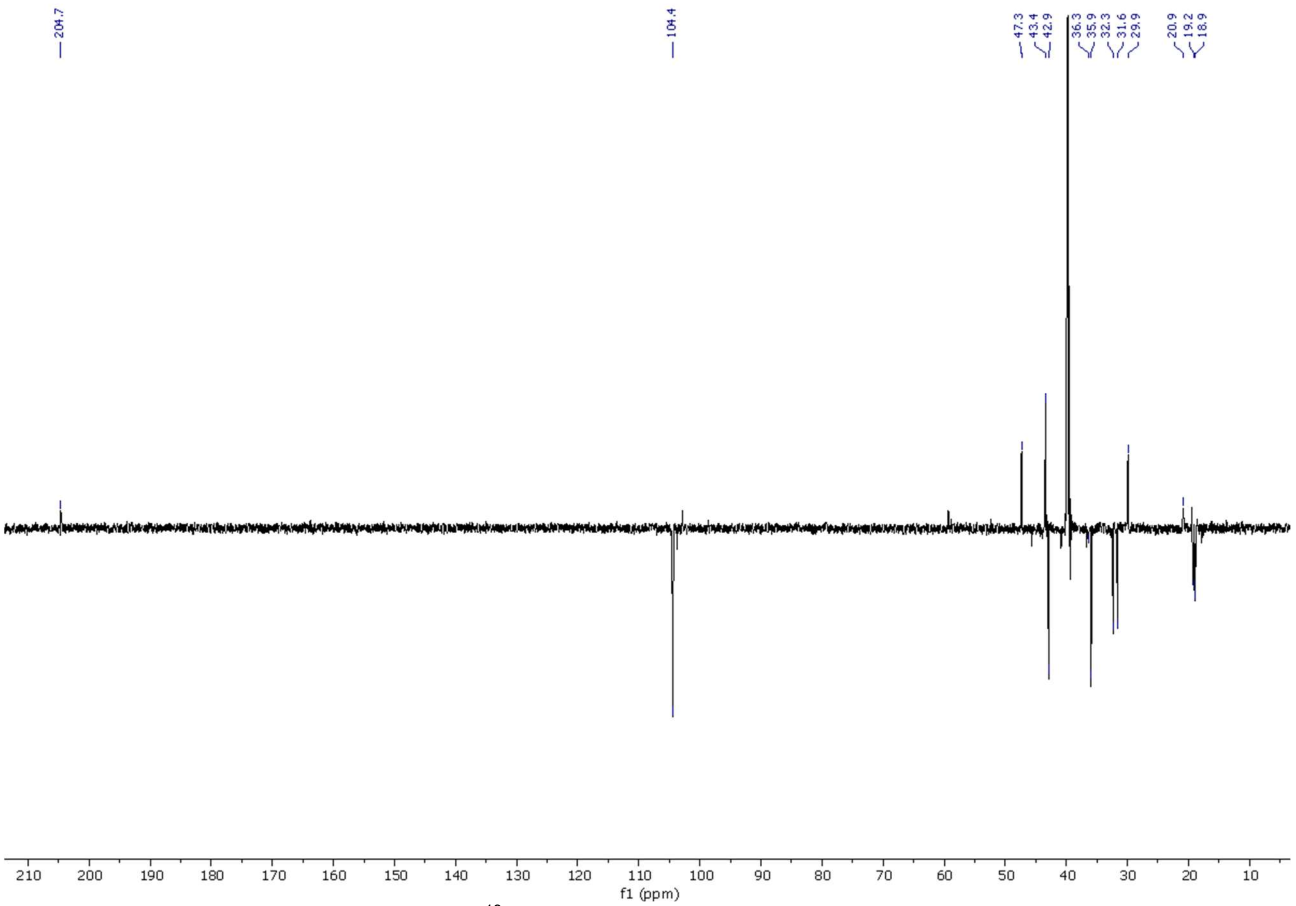

Figure N24.K ^{13}C DEPT 135 of **24** in DMSO-d_6 at 151 MHz.

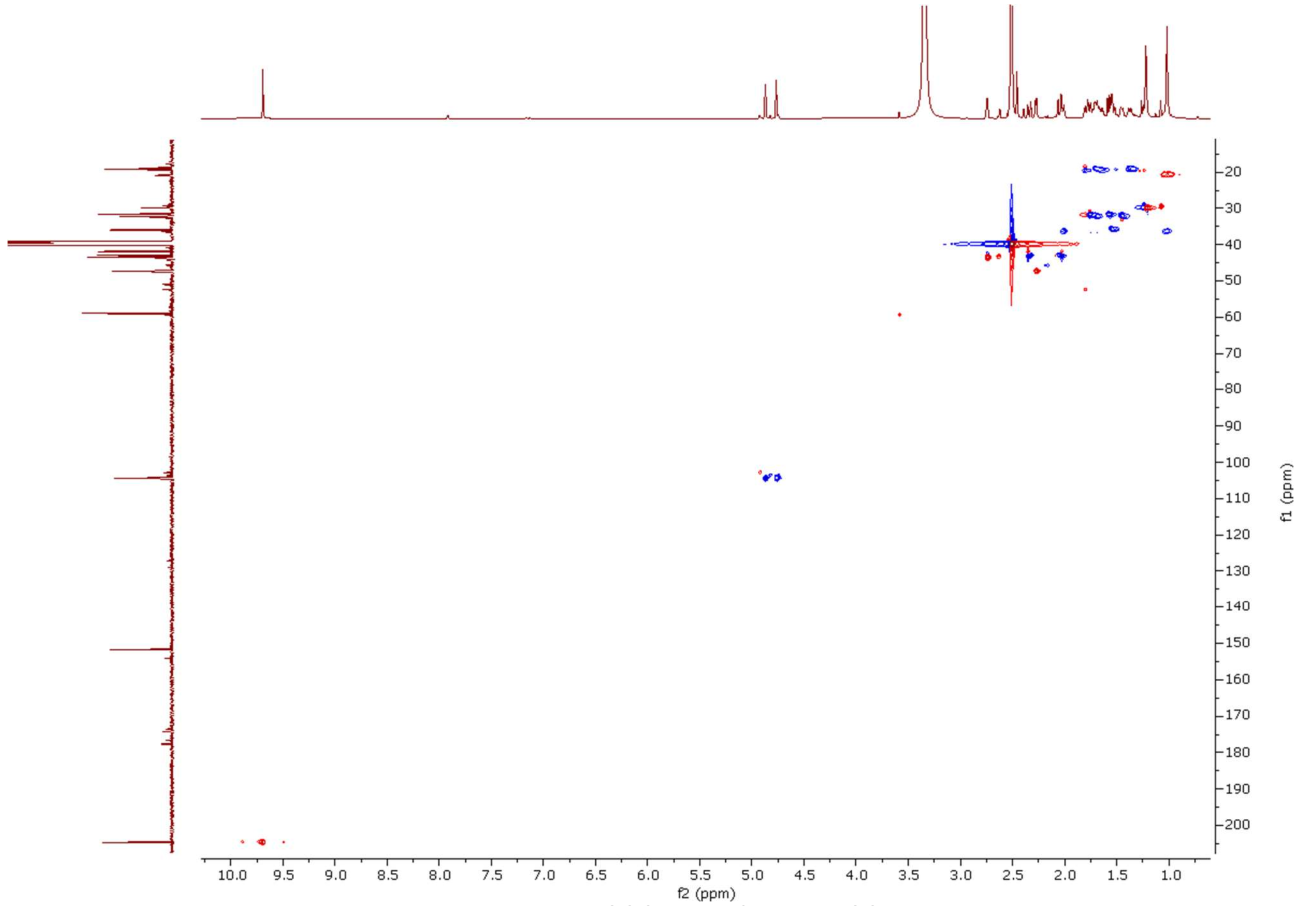

Figure N24.L HSQC NMR of **24** in DMSO-d_6.

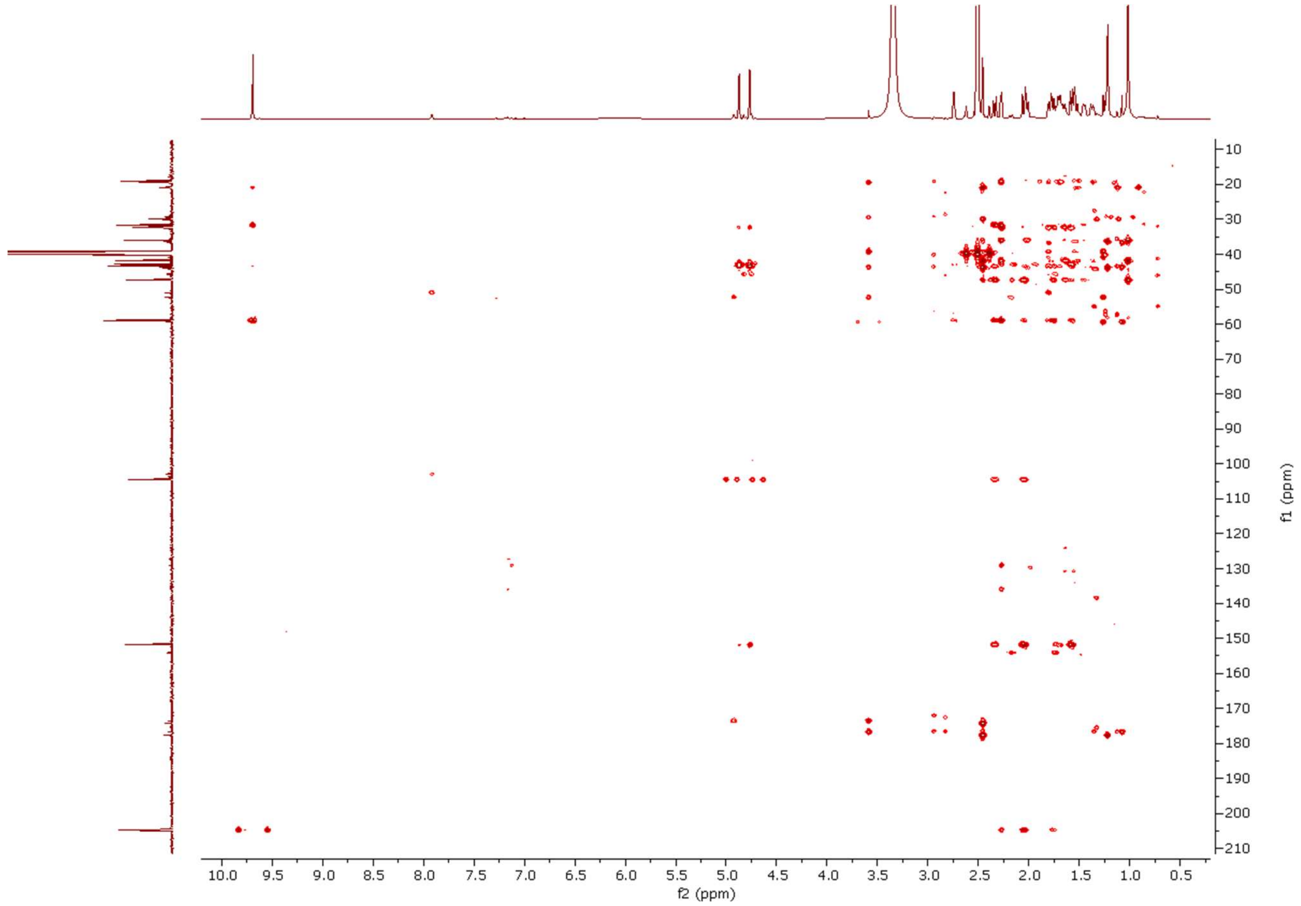

Figure N24.M HMBC NMR of **24** in DMSO-d6.

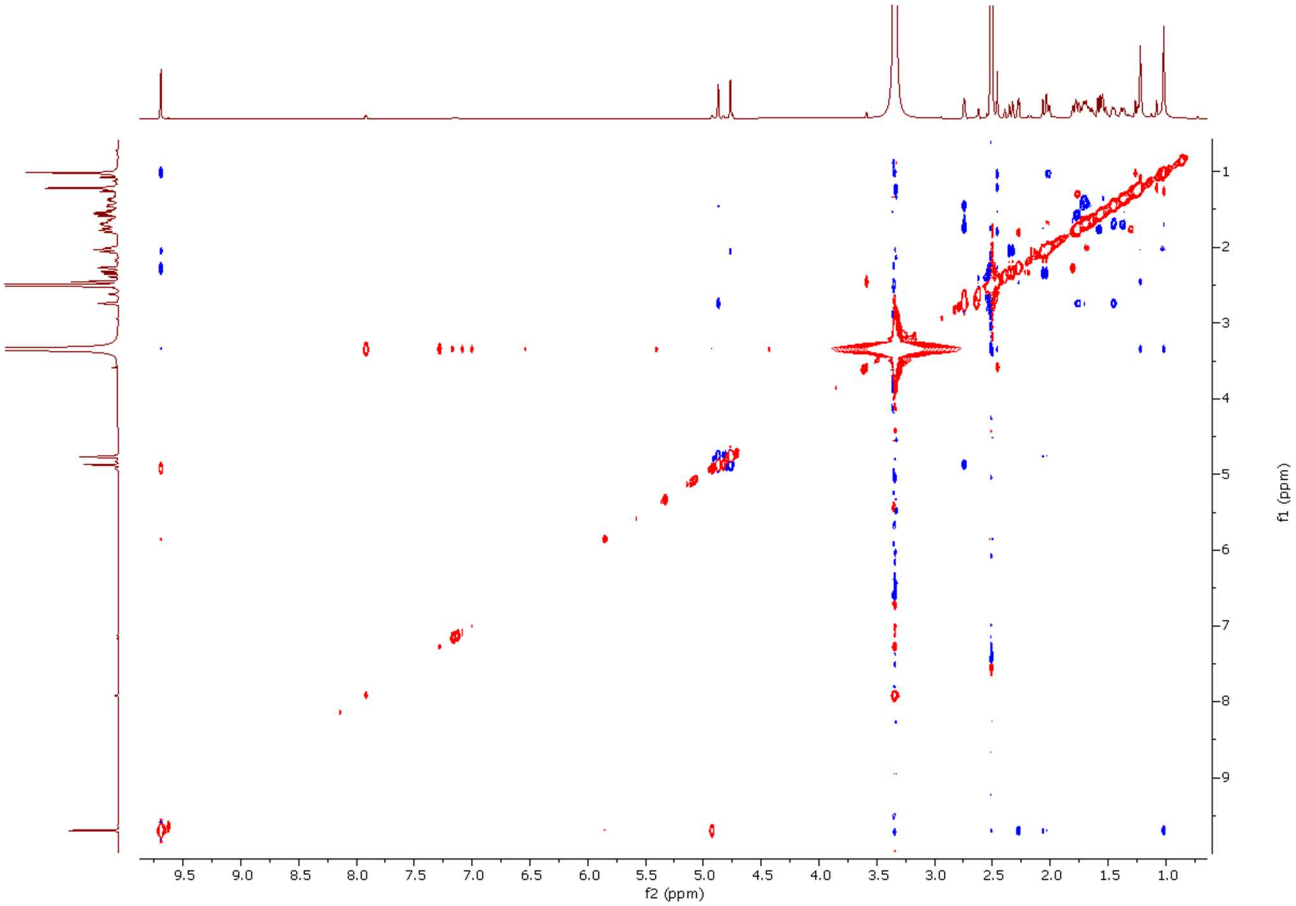

Figure N24.N NOESY NMR of **24** in DMSO-d6 at 600 MHz.

Fujenoic acid dimethyl ester (24a)

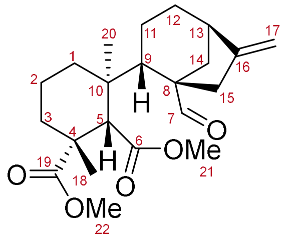

Fujenoic acid dimethyl ester (**24a**)
Chemical Formula: $C_{22}H_{32}O_5$
Exact Mass: 376.2250

	**Measured in $CDCl_3$, 298K**		
**Pos.**	δ_C 151 MHz	δ_H (*J/Hz*) 600 MHz	**Selected NOESY correlations to H** [a]
**1**	36.8	ax 1.07, overlapped<br>eq 2.20, overlapped	H-1eq<br>H-1ax
**2**	19.0	a 1.49, m<br>b 1.87, m	H-2b<br>H_3-20, H-2a
**3**	36.8	a 1.53, overlapped<br>b 1.68, overlapped	H-5, H-3b<br>H-3a
**4**	44.8	-	-
**5**	58.5	2.53, s	H_3-18, H-1ax
**6**	172.9	-	-
**7**	205.1	9.79, br s	H-15a, H_3-20
**8**	59.7	-	-
**9**	48.3	2.15, overlapped	H-15b
**10**	42.4	-	-
**11**	19.7	a 1.71, overlapped<br>b 1.79, m	
**12**	32.8	a 1.53, overlapped<br>b 1.73, overlapped	
**13**	44.0	2.79, br s	H-14b, H-17a, H-12a, H-12b
**14**	32.1	a 1.62, br d (12.3)<br>b 1.96, dd (12.3, 4.9)	H-14b<br>H-14a, H-13
**15**	43.5	a 2.16, overlapped<br>b 2.35, d (17.0)	H-7, H-15b<br>H-15a, H-9
**16**	151.8	-	-
**17**	104.8	b 4.77, br s<br>a 4.87, br s	H-17a, H-15a, H-15b<br>H-13, H-17b, H-12a
**18**	30.6	1.30, s	H-5
**19**	176.4	-	-
**20**	20.7	1.09, s	H-2b, H-7
**21**	51.2	3.71, s	
**22**	51.9	3.62, s	

[a] Key NOESY correlations are shown in blue text.

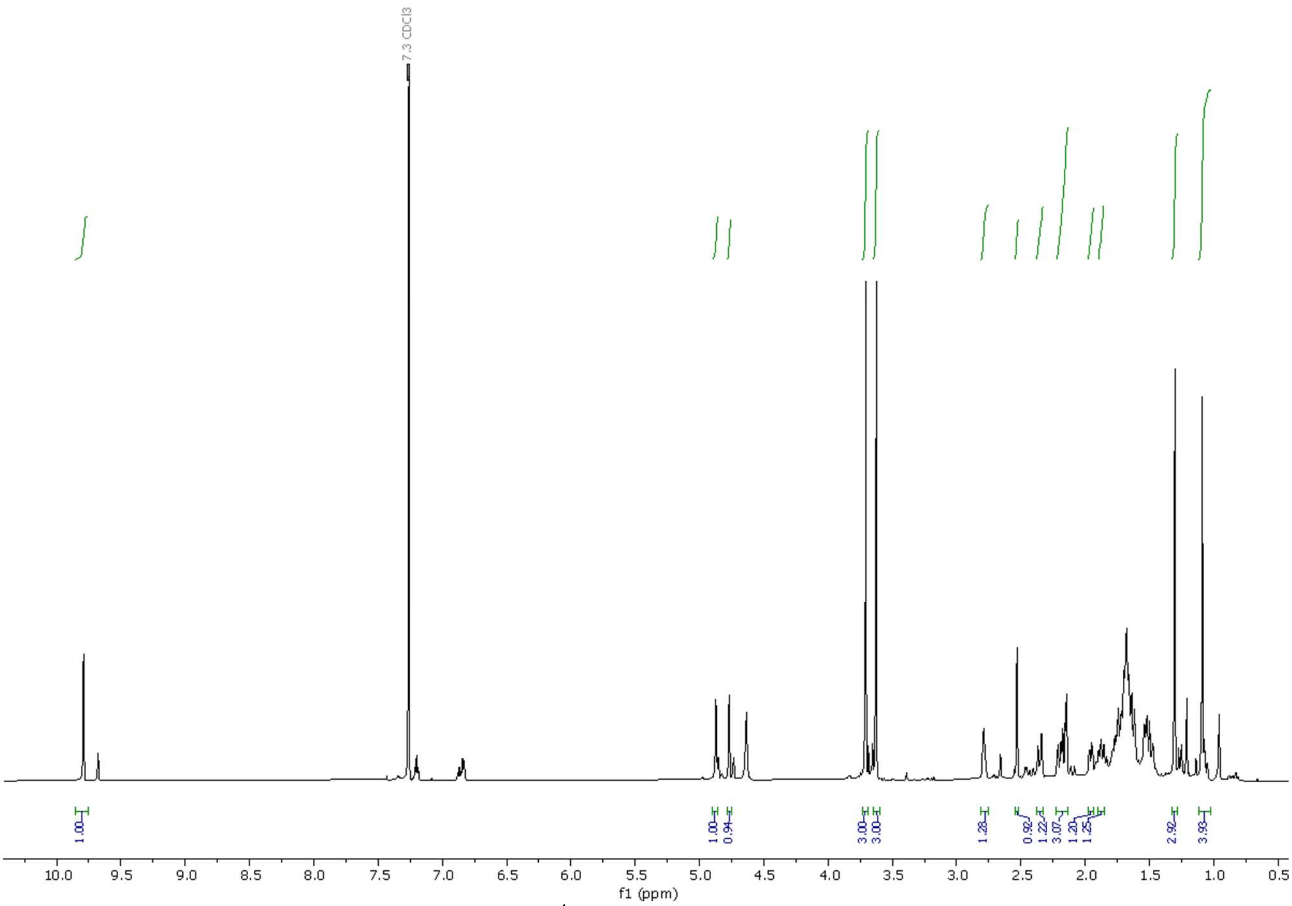

Figure N24a.A ^{1}H NMR of **24a** in $CDCl_3$ at 600 MHz.

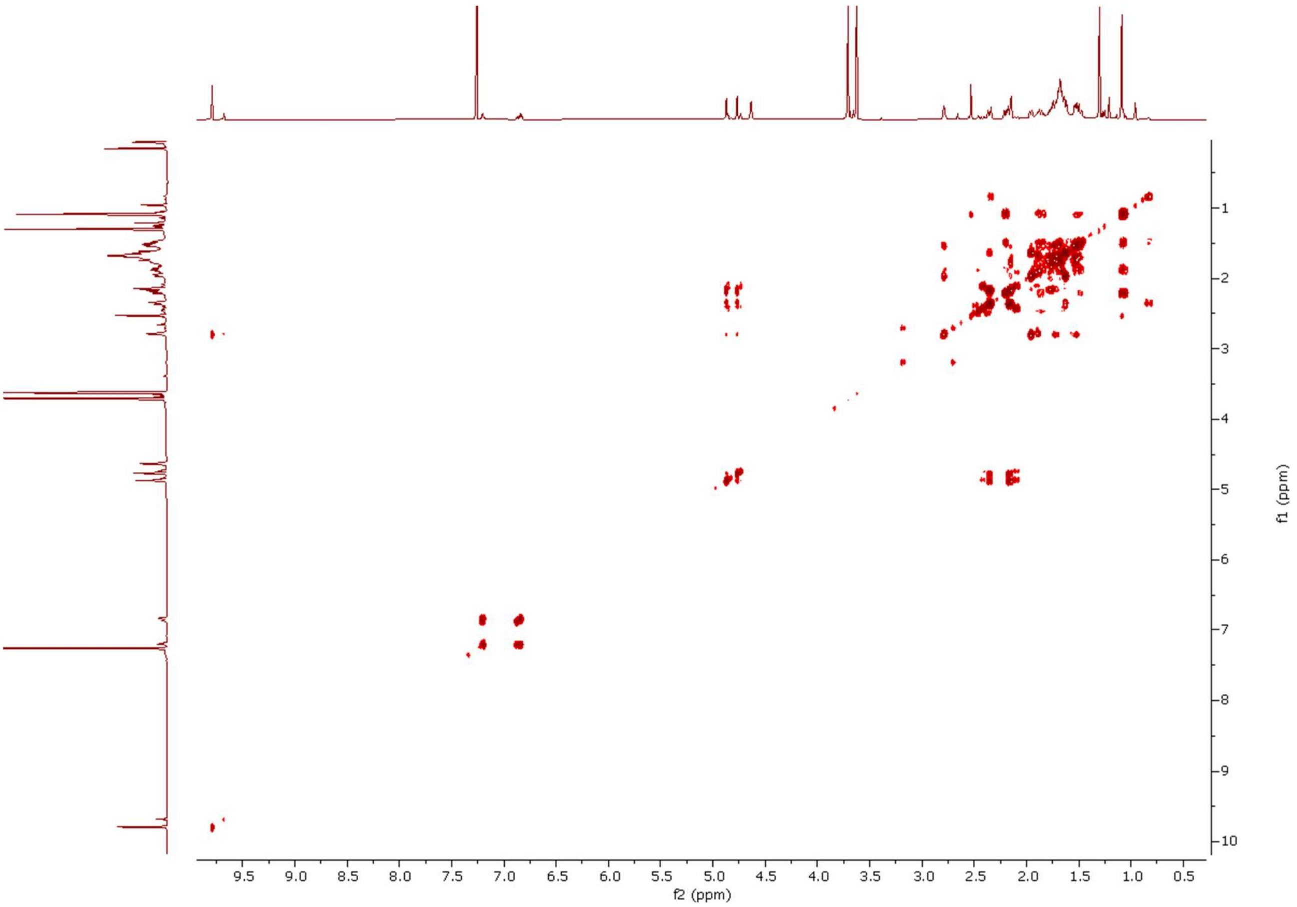

Figure N24a.B COSY NMR of **24a** in $CDCl_3$ at 600 MHz.

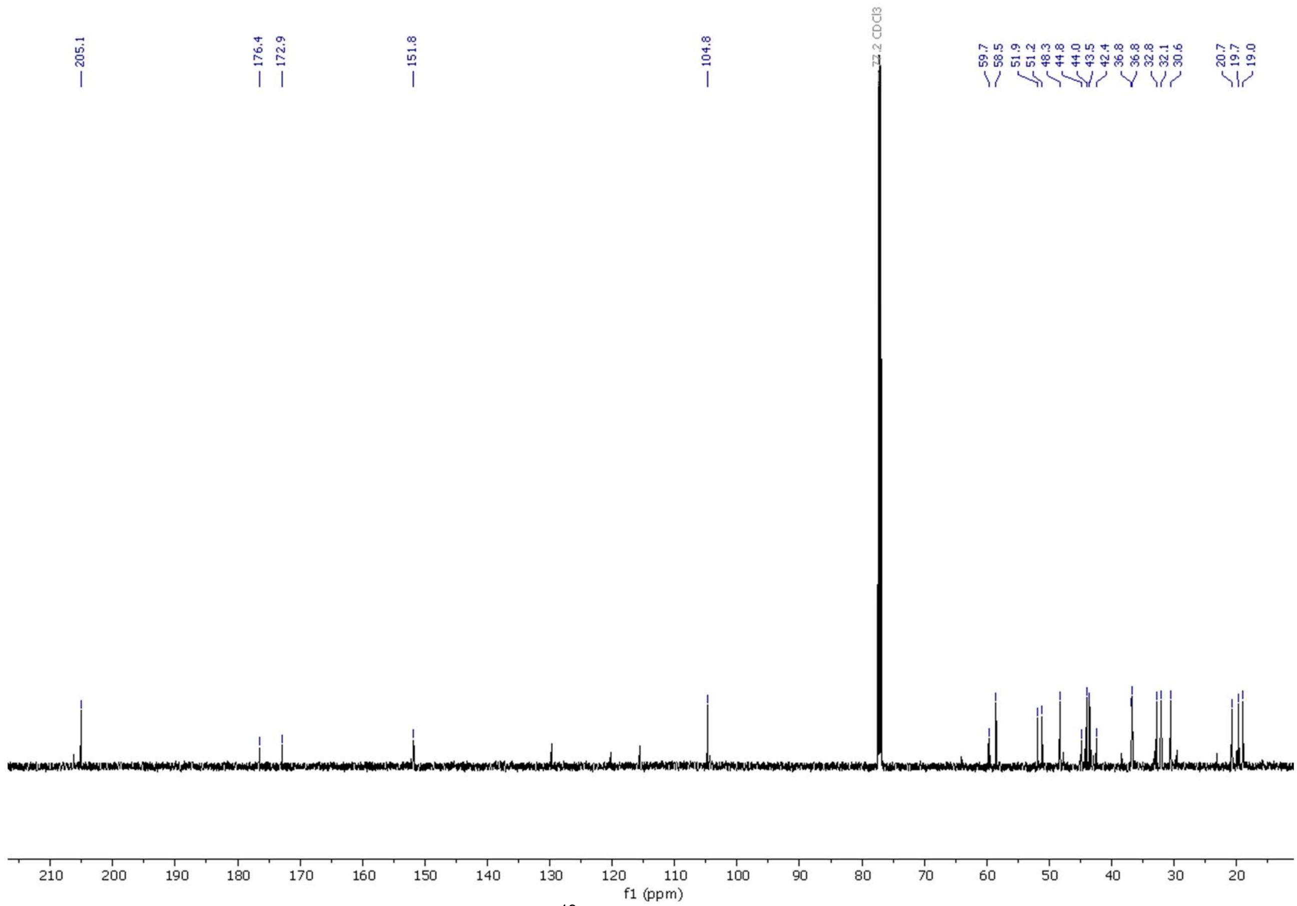

Figure N24a.C ^{13}C NMR of **24a** in $CDCl_3$ at 151 MHz.

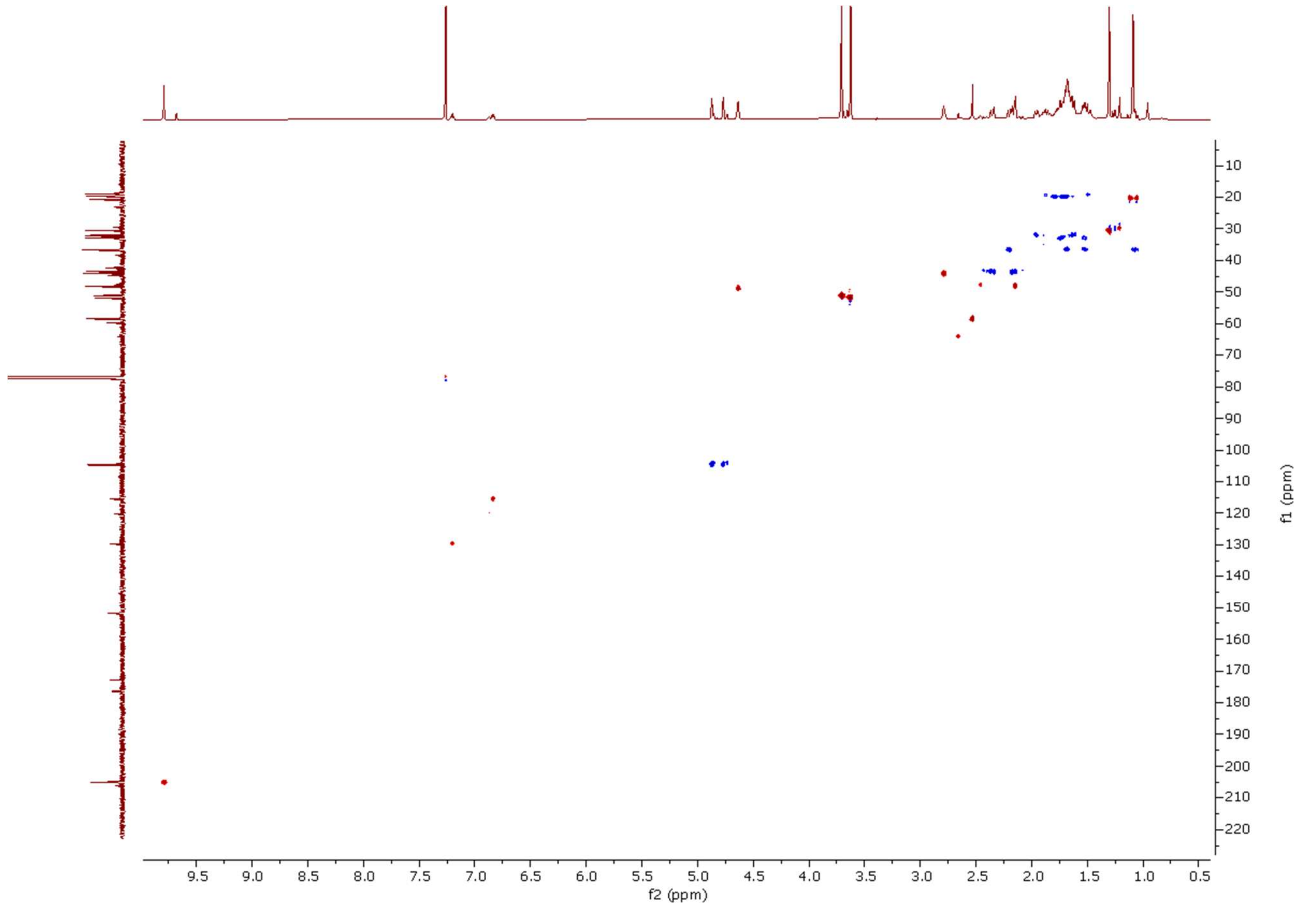

Figure N24a.D HSQC NMR of **24a** in $CDCl_3$.

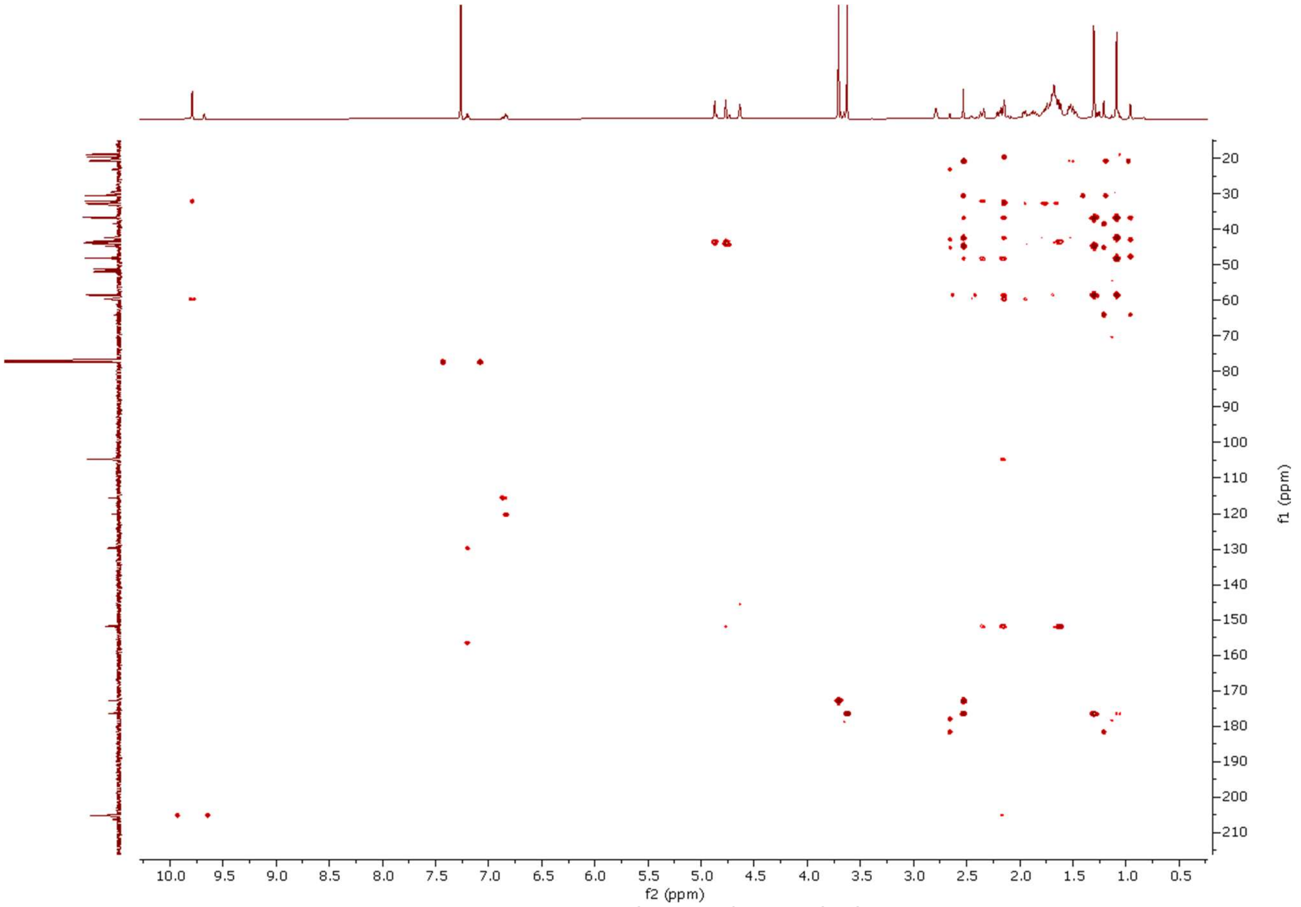

Figure N24a.E HMBC NMR of **24a** in $CDCl_3$.

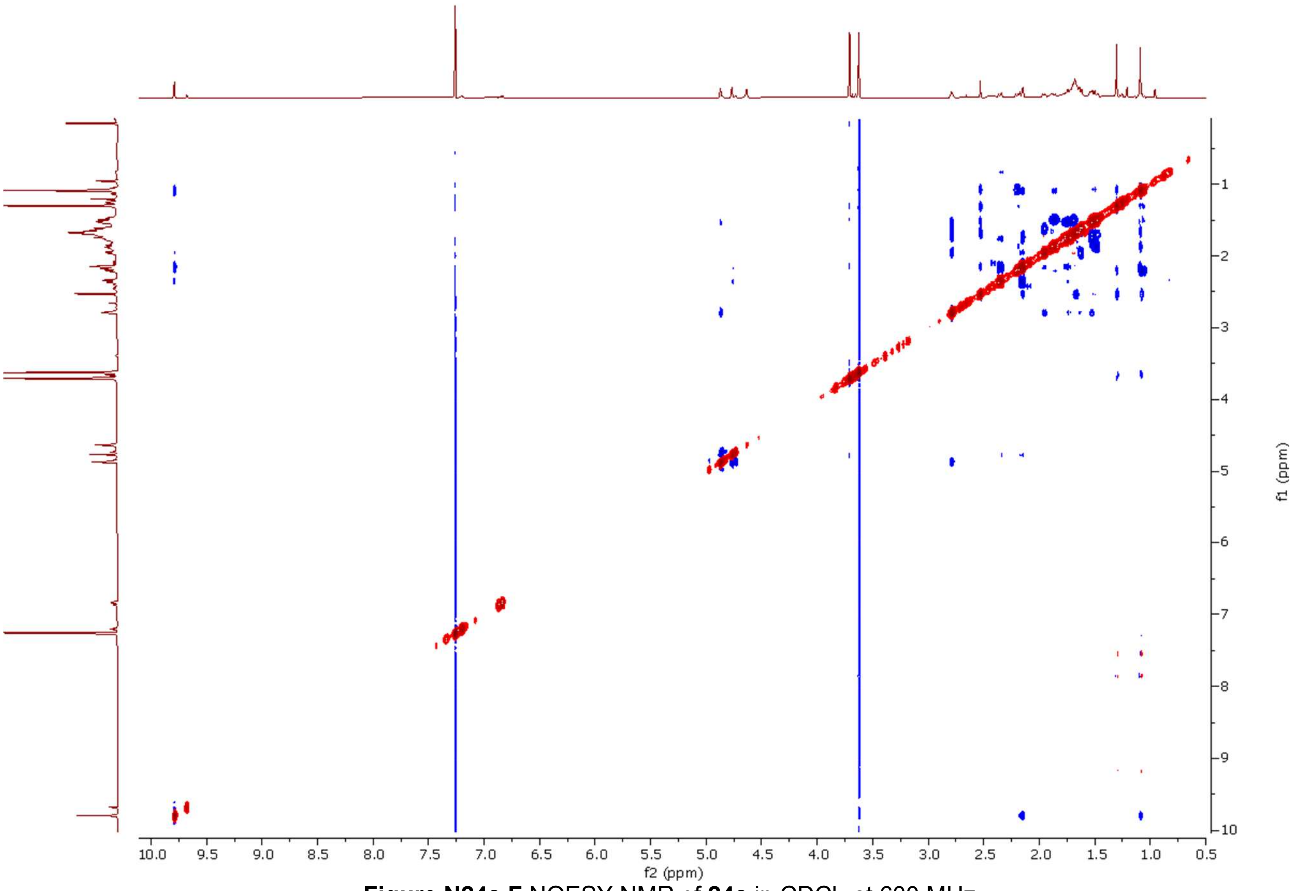

Figure N24a.F NOESY NMR of **24a** in $CDCl_3$ at 600 MHz.

Fujenoic triacid trimethyl ester (25a)

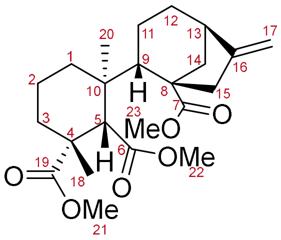

Fujenoic triacid trimethyl ester (**25a**)
Chemical Formula: $C_{23}H_{34}O_6$
Exact Mass: 406.2355

	Measured in $CDCl_3$, 298K		
**Pos.**	δ_C 151 MHz	δ_H *(J/Hz)* 600 MHz	**Selected NOESY correlations to H** [a]
**1**	36.4	ax 1.10, overlapped<br>eq 2.16, overlapped	H-1eq<br>H-1ax
**2**	19.0	a 1.48, overlapped<br>b 1.94, m	
**3**	36.6	ax 1.53 overlapped<br>eq 1.78, ddd (12.5, 3.5, 3.5)	H-3eq, H-5<br>H-3ax, H-2b, H-2a
**4**	44.5	-	-
**5**	58.8	2.55, s	H_3-18, H-3ax, H-9
**6**	172.5	-	-
**7**	178.8	-	-
**8**	53.0	-	-
**9**	48.3	2.13, d (6.6)	H-5
**10**	42.4	-	-
**11**	19.6	a 1.69, overlapped<br>b 1.73, overlapped	
**12**	32.3	a 1.45, overlapped<br>b 1.70, overlapped	H-13
**13**	43.7	2.72, br s	H-12a, H_2-14, H-17a
**14**	35.0	1.90, br s	H-13
**15**	46.9	a 2.37, ddd (17.4, 2.5, 2.5)<br>b 2.49, d (17.4)	H-15b<br>H-9, H-15a
**16**	153.1	-	-
**17**	104.0	b 4.74, br s<br>a 4.83, dd (2.5, 2.5)	H-17a, H-15a, H-15b<br>H-13, H-17b
**18**	30.3	1.27, s	H-5
**19**	176.7	-	-
**20**	18.3	1.09, s	H-2b, H_3-21, H_3-23
**21**	51.8	3.63, s	H_3-20, H_3-18
**22**	51.1	3.69, s	H-9
**23**	51.6	3.66, s	H_3-20

[a] Key NOESY correlations are shown in blue text.

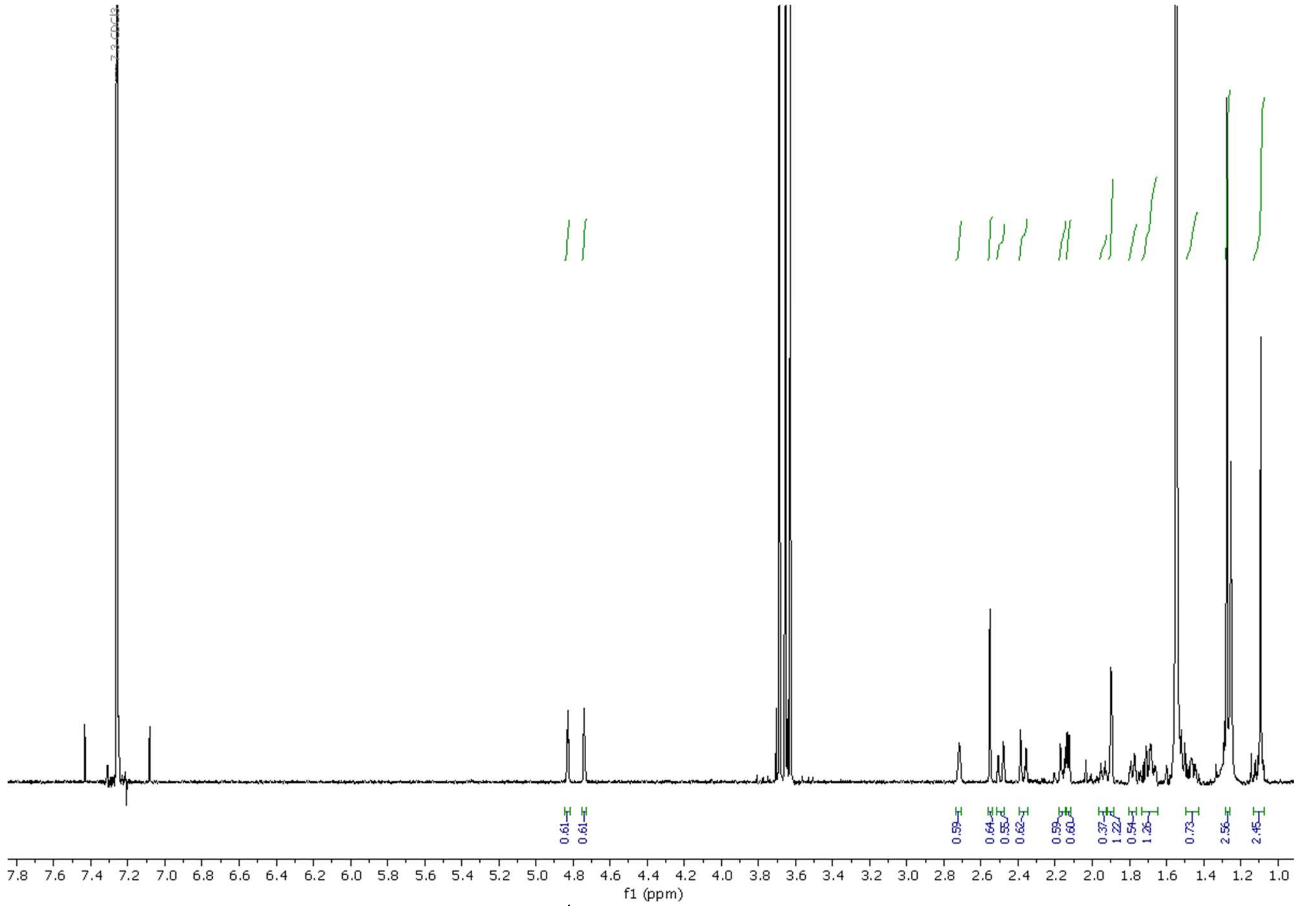

Figure N25a.A ^{1}H NMR of **25a** in $CDCl_3$ at 600 MHz.

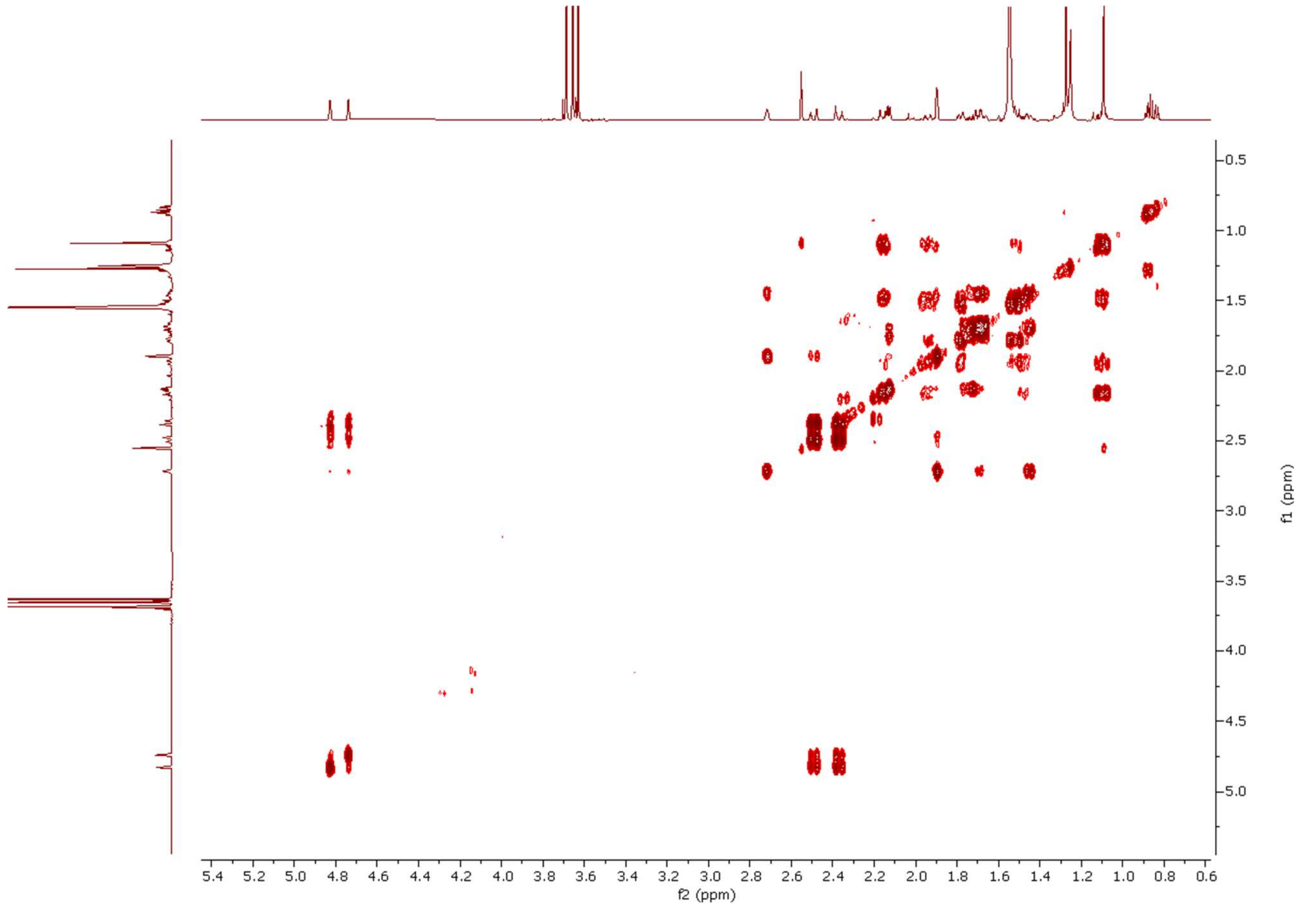

Figure N25a.B COSY NMR of **25a** in $CDCl_3$ at 600 MHz.

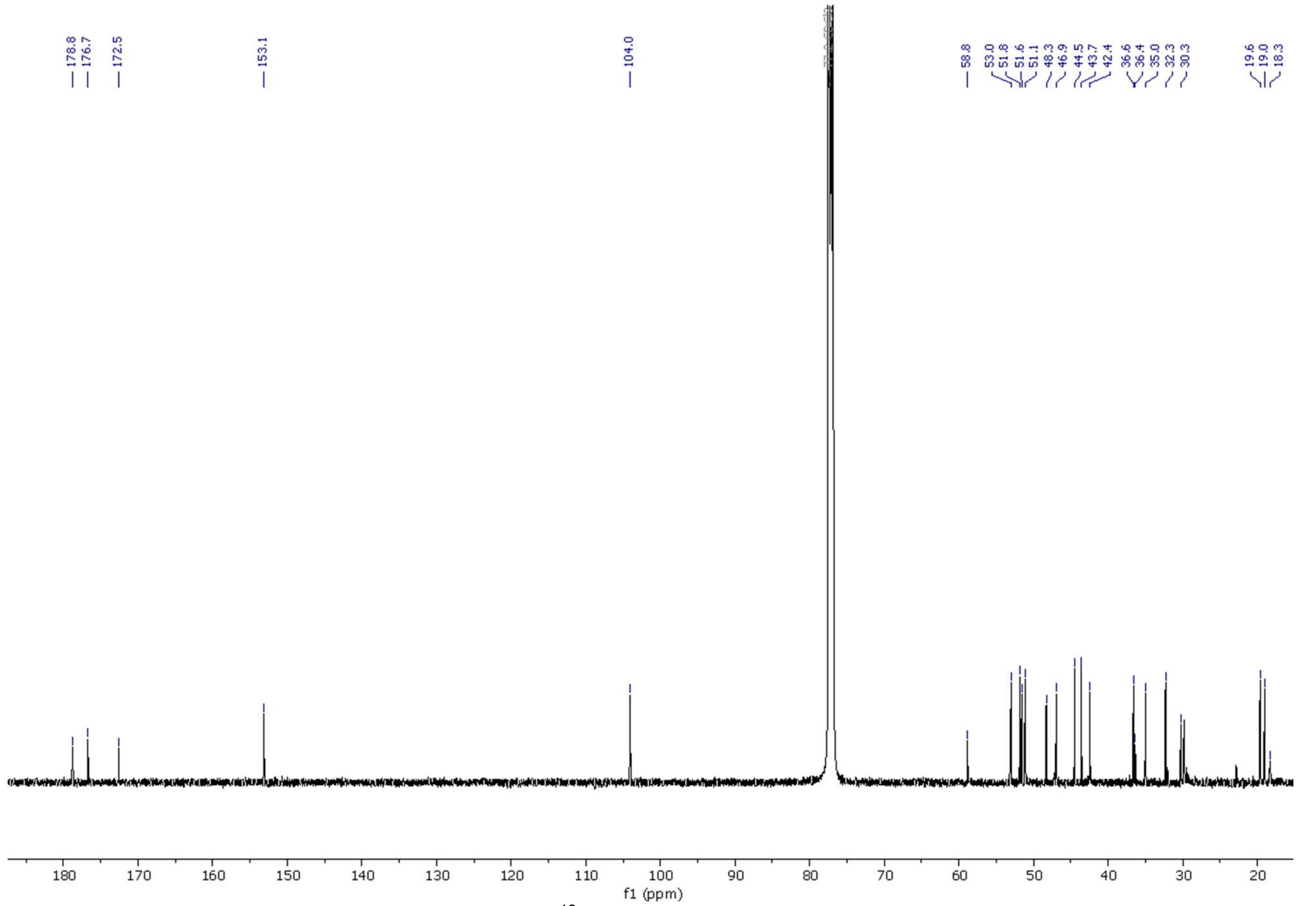

Figure N25a.C ^{13}C NMR of **25a** in $CDCl_3$ at 151 MHz.

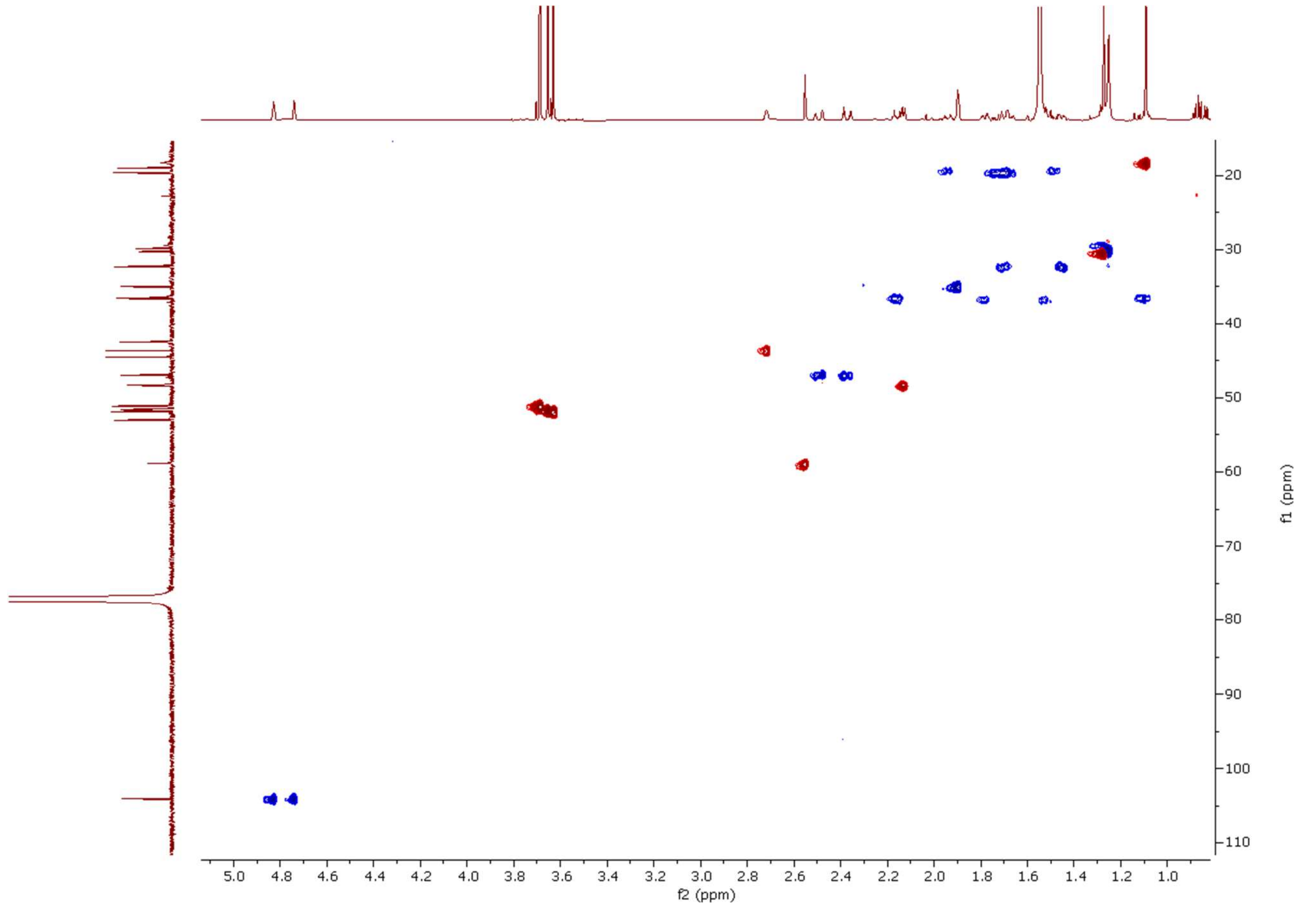

Figure N25a.D HSQC NMR of **25a** in $CDCl_3$.

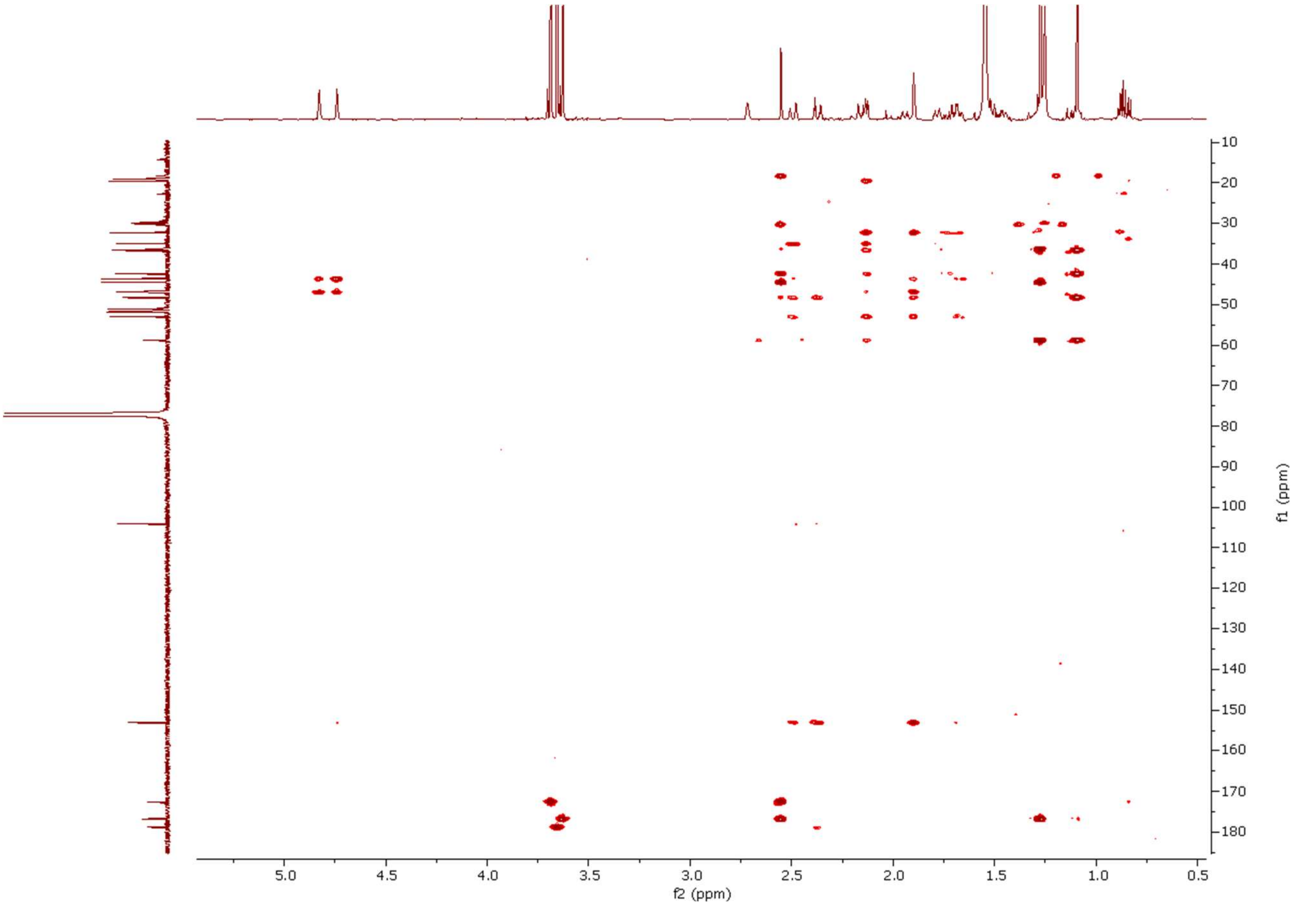

Figure N25a.E HMBC NMR of **25a** in $CDCl_3$.

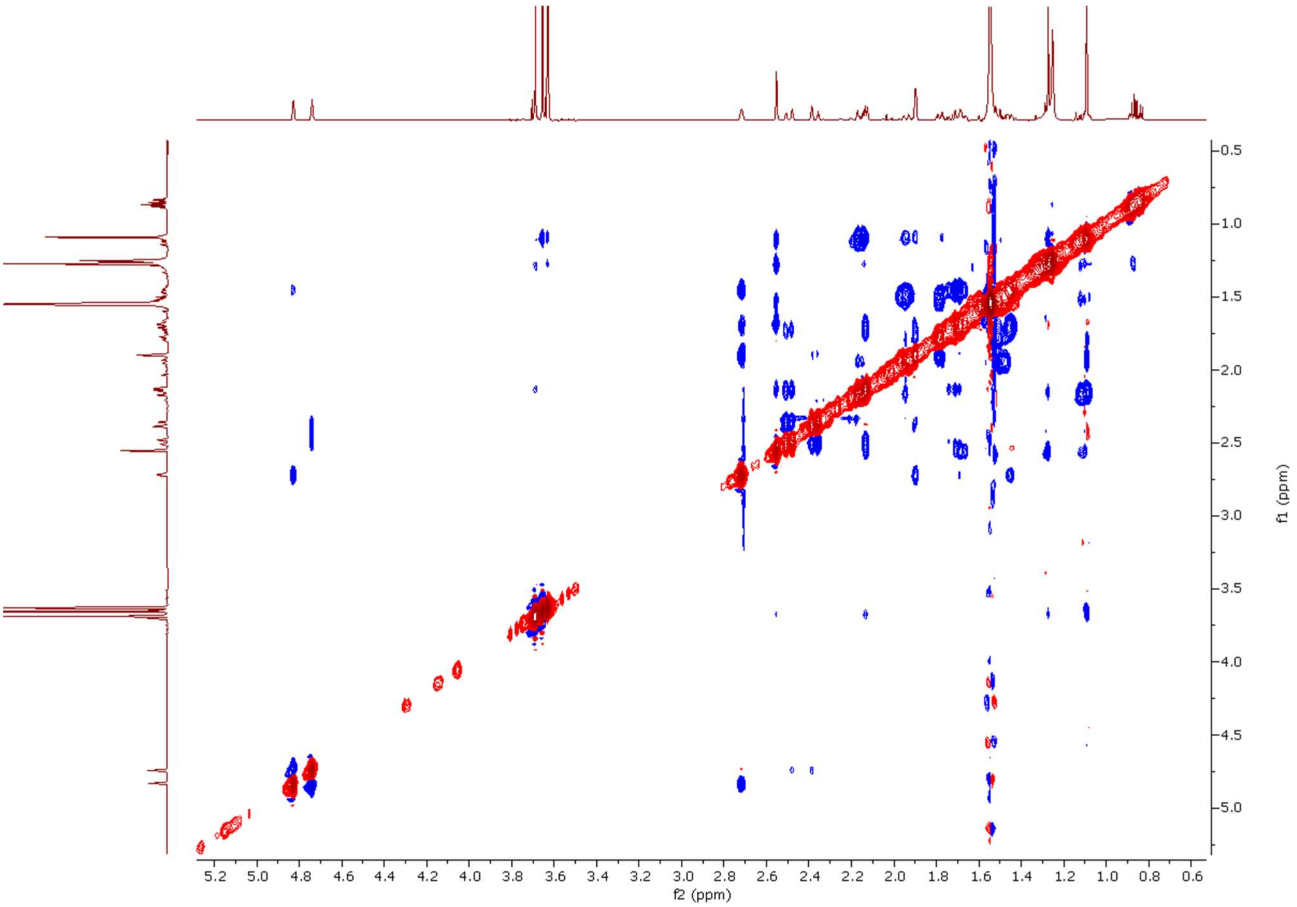

Figure N25a.F NOESY NMR of **25a** in $CDCl_3$ at 600 MHz.

7β,16α,17-Trihydroxy-*ent*-kauranoic acid (26)

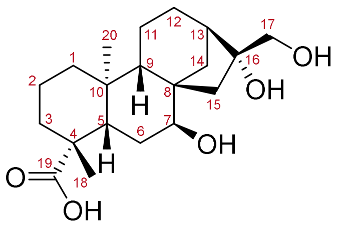

7β,16α,17-Trihydroxy-*ent*-kauranoic acid (**26**)
Chemical Formula: $C_{20}H_{32}O_5$
Exact Mass: 352.2250

Pos.	Reference in acetone-d_6 [48]		Measured in DMSO-d_6, 298K	
	δ_C<br>125 MHz	δ_H (J/Hz)<br>500 MHz	δ_C<br>151 MHz	δ_H (J/Hz)<br>600 MHz
**1**	41.3	1.76-1.80, m	40.2	ax 0.76, ddd (13.0, 13.0, 3.1)<br>eq 1.73, overlapped
**2**	19.9	1.30-1.40, m	18.9	a 1.33, overlapped<br>b 1.80, overlapped
**3**	38.9	0.93-0.95, m	37.9	ax 0.93, ddd (13.3, 12.0, 3.2)<br>eq 2.02, br d (12.0)
**4**	43.4	-	42.4	-
**5**	47.3	1.85, d (3.6)	46.1	1.67, br d (12.5)
**6**	30.0	1.88-1.90, m	29.4	a 1.77, overlapped<br>b 1.89, overlapped
**7**	77.0	3.58, s	75.2	3.40, overlapped
**8**	49.3	-	48.1	-
**9**	50.5	1.43-1.46, m	49.3	1.29, overlapped
**10**	39.9	-	38.7	-
**11**	18.8	1.40-1.42, m<br>1.47-1.51, m	17.8	1.44, overlapped
**12**	29.3	1.90-1.93, m	26.4	a 1.39, overlapped<br>b 1.54, overlapped
**13**	45.9	2.01-2.05, m	44.5	1.88, overlapped
**14**	37.0	1.61-1.65, m<br>1.70, s	36.0	1.56, overlapped
**15**	50.3	1.71-1.74, m	49.4	1.31, overlapped<br>1.57, overlapped
**16**	81.7	-	80.5	-
**17**	66.3	3.56, d (15.0)<br>3.68, d (15.0)	65.3	3.39, overlapped<br>3.48, d (11.0)
**18**	29.3	1.10, s	28.5	1.04, s
**19**	176.3	-	179.1	-
**20**	15.9	0.95, s	15.3	0.86, s

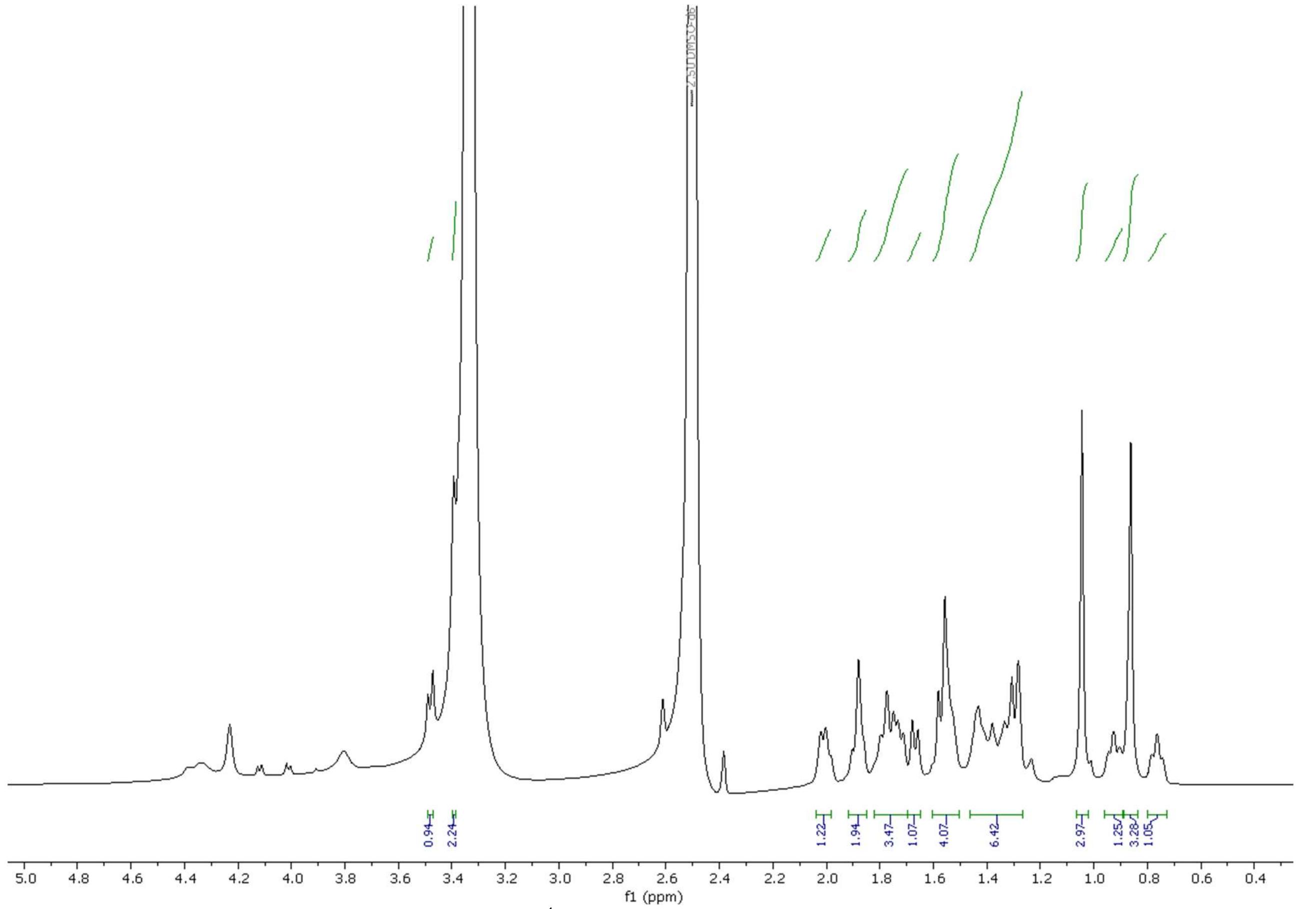

Figure N26.A ^{1}H NMR of **26** in DMSO-d$_6$ at 600 MHz.

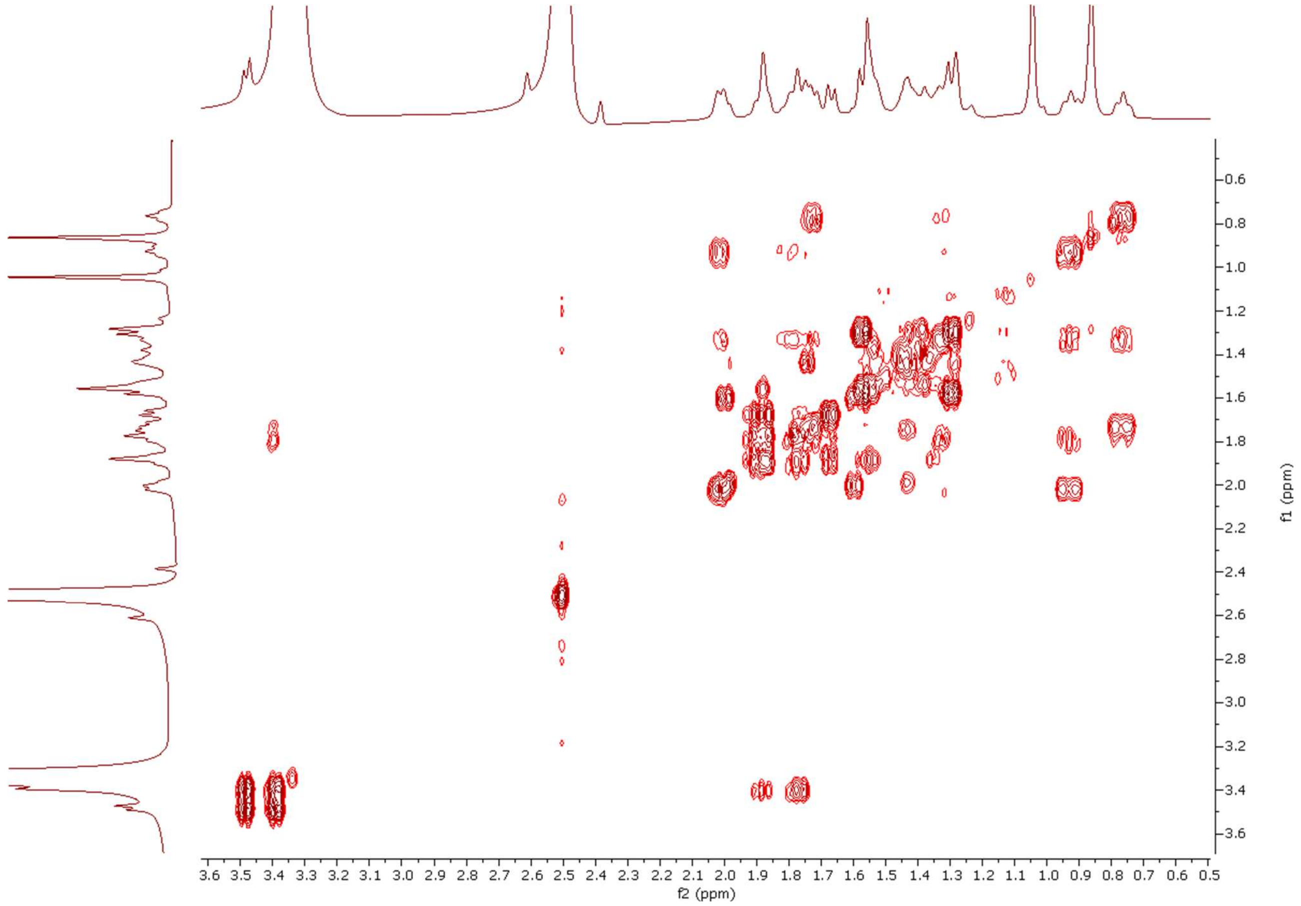

Figure N26.B COSY NMR of **26** in DMSO-d$_6$ at 600 MHz.

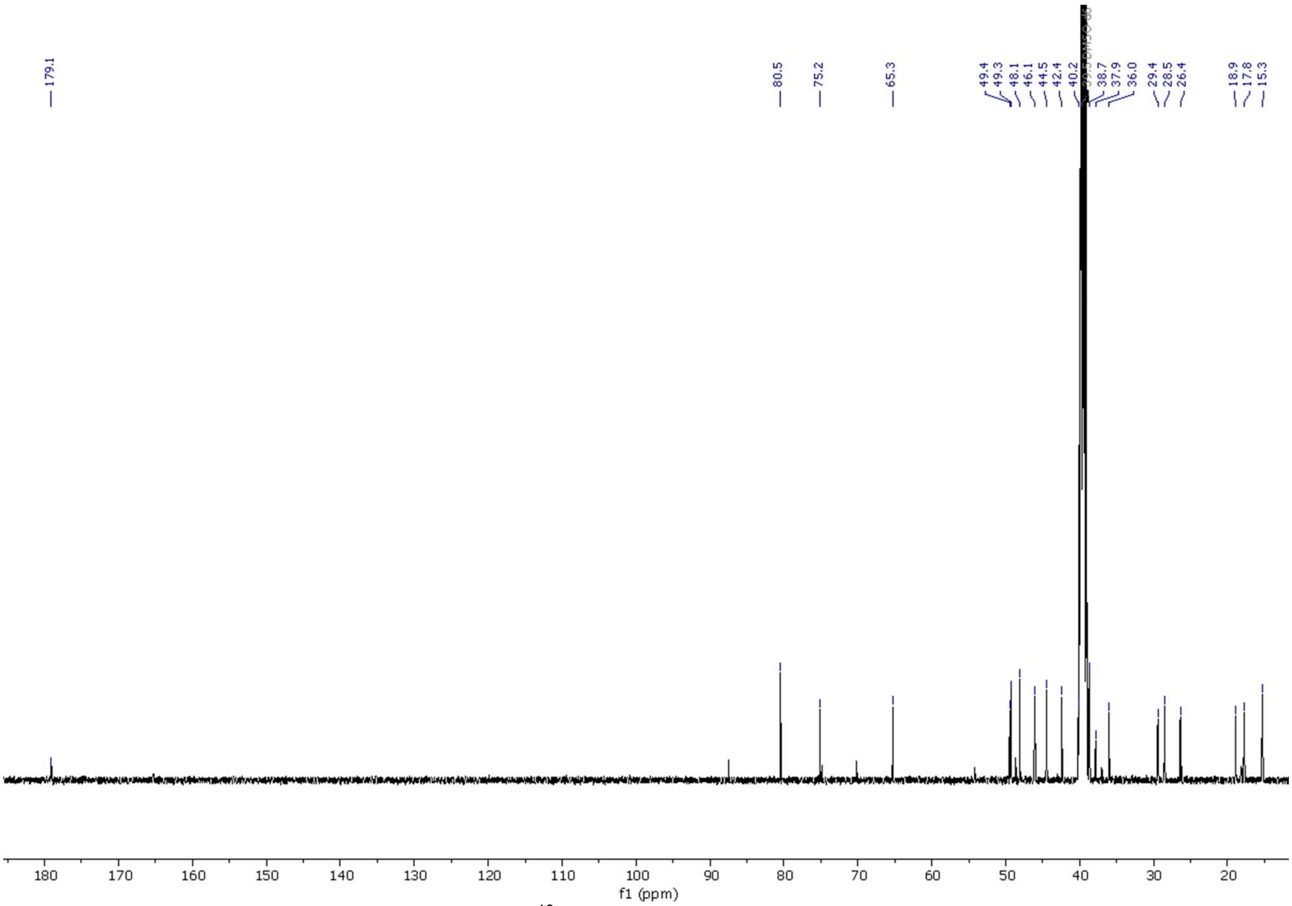

Figure N26.C ^{13}C NMR of **26** in DMSO-d$_6$ at 151 MHz.

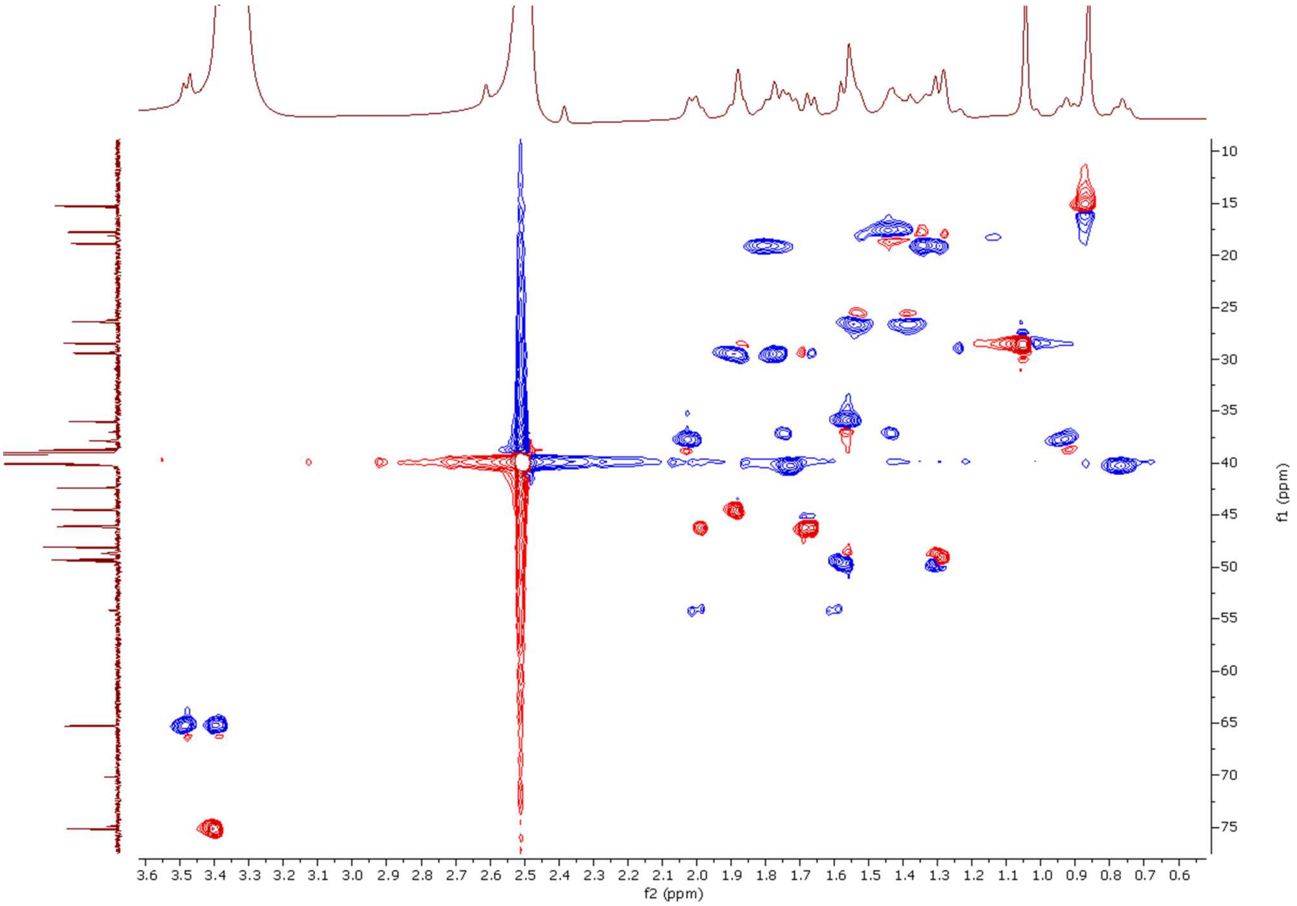

Figure N26.D HSQC NMR of **26** in DMSO-d$_6$.

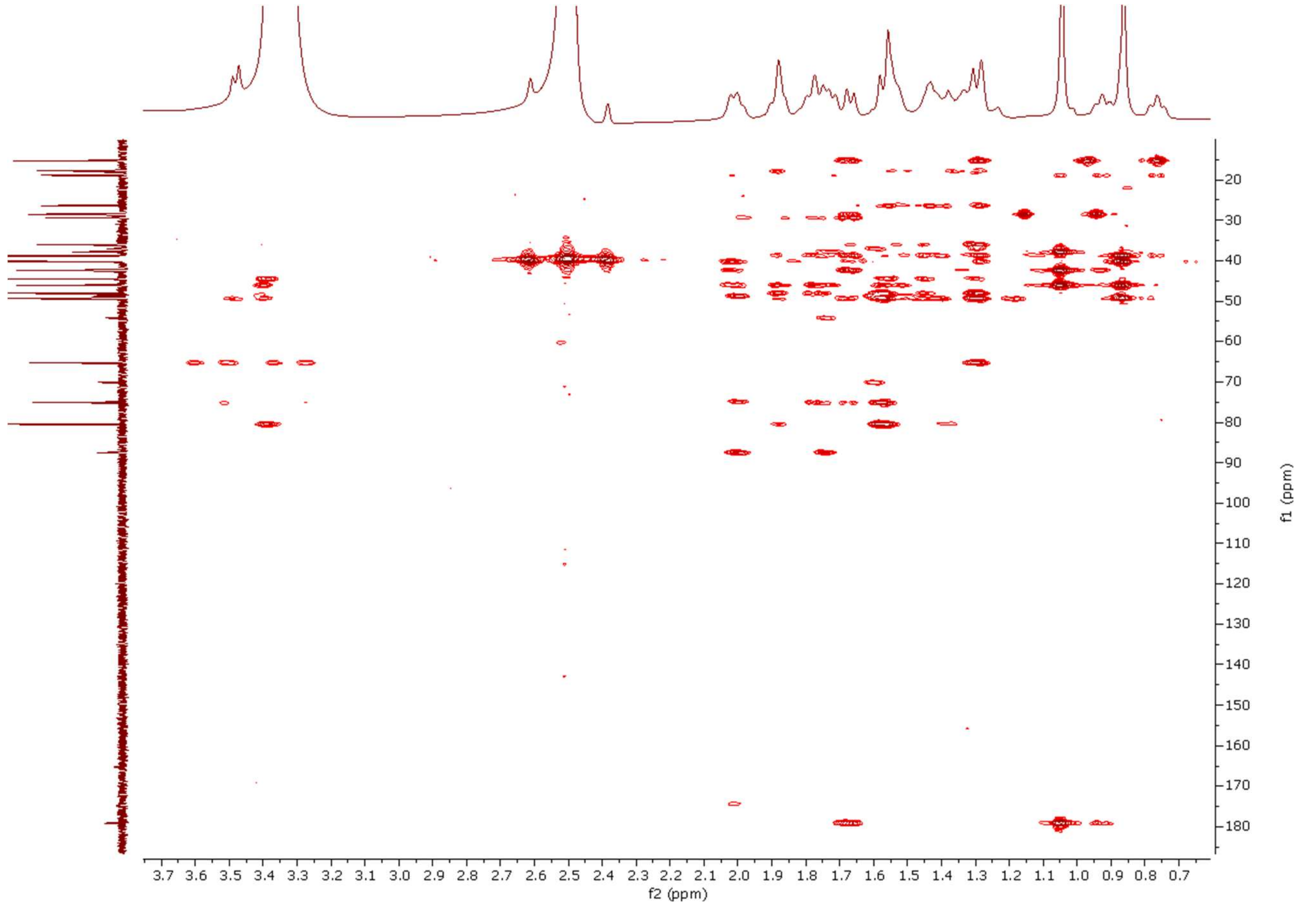

Figure N26.E HMBC NMR of **26** in DMSO-d6.

7β,16α,17-Trihydroxy-*ent*-kauranoic acid methyl ester (26a)

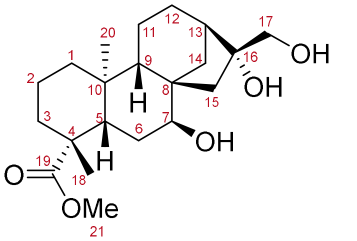

7β,16α,17-Trihydroxy-*ent*-kauranoic acid methyl ester (**26a**)
Chemical Formula: $C_{21}H_{34}O5$
Exact Mass: 366.2406

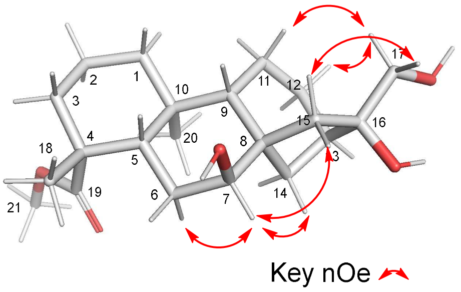

	Measured in $CDCl_3$, 298K		Selected NOESY correlations to H [a]
**Pos.**	δ_C 126 MHz	δ_H *(J/Hz)* 600 MHz	
**1**	40.5	ax 0.89, ddd (13.3, 13.3, 3.5)<br>eq 1.80, overlapped	H-2a
**2**	19.2	a 1.43, overlapped<br>b 1.83, overlapped	H-1ax
**3**	38.0	ax 1.07, ddd (13.6, 13.6, 4.2)<br>eq 2.19, ddd (13.6, 3.5, 3.5)	H-3eq<br>H-3ax, H_3-18
**4**	43.5	-	-
**5**	47.2	1.72, overlapped	H_3-18
**6**	29.3	a 1.98, overlapped<br>b 2.04, overlapped	H_3-18<br>H_3-20
**7**	77.2	3.72, br s	H-14a, H-6a, H-6b, H-15b
**7-OH**	-	1.37, br s	
**8**	48.7	-	-
**9**	49.7	1.42, overlapped	
**10**	39.2	-	-
**11**	18.1	a 1.45, overlapped<br>b 1.62, overlapped	
**12**	26.7	a 1.55, overlapped<br>b 1.59, overlapped	
**13**	45.3	2.07, overlapped	
**14**	36.4	a 1.68, overlapped<br>b 1.82, overlapped	 H_3-20
**15**	49.6	a 1.61, overlapped<br>b 1.71, overlapped	
**16**	81.8	-	-
**17**	66.3	a 3.68, dd (11.0, 5.2)<br>b 3.79, dd (11.0, 5.2)	H-15a, H-13<br>H-11a, H-12b, H-13
**17-OH**	-	1.87, br t (5.2)	
**18**	28.7	1.16, s	H-3eq, H-6a, H-5
**19**	178.3	-	
**20**	15.4	0.84, s	H-14b, H_3-21, H-6b, H-2b
**21**	51.4	3.65, s	H_3-20

[a] Key NOESY correlations are shown in blue text.

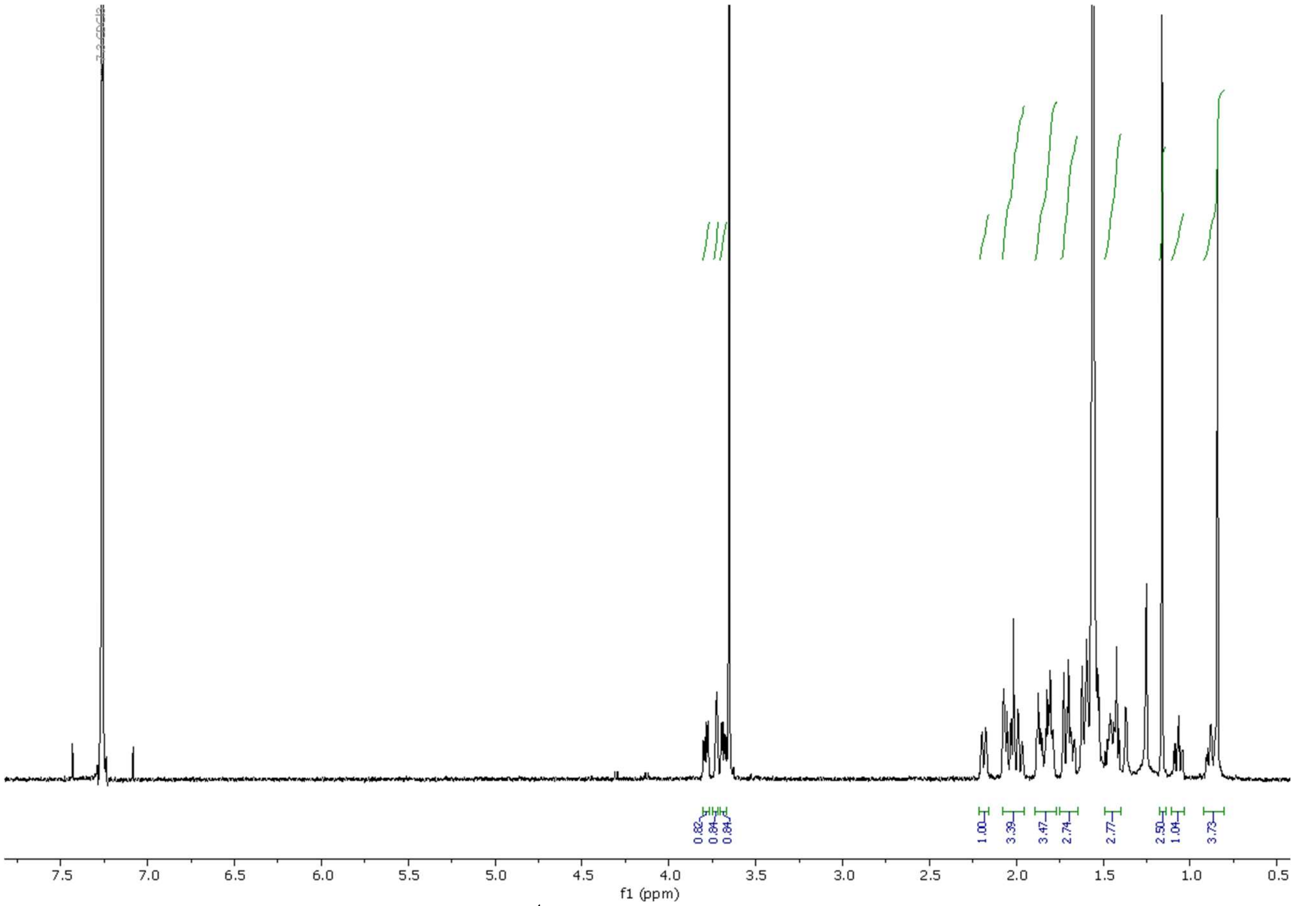

Figure N26a.A ^{1}H NMR of **26a** in $CDCl_3$ at 600 MHz.

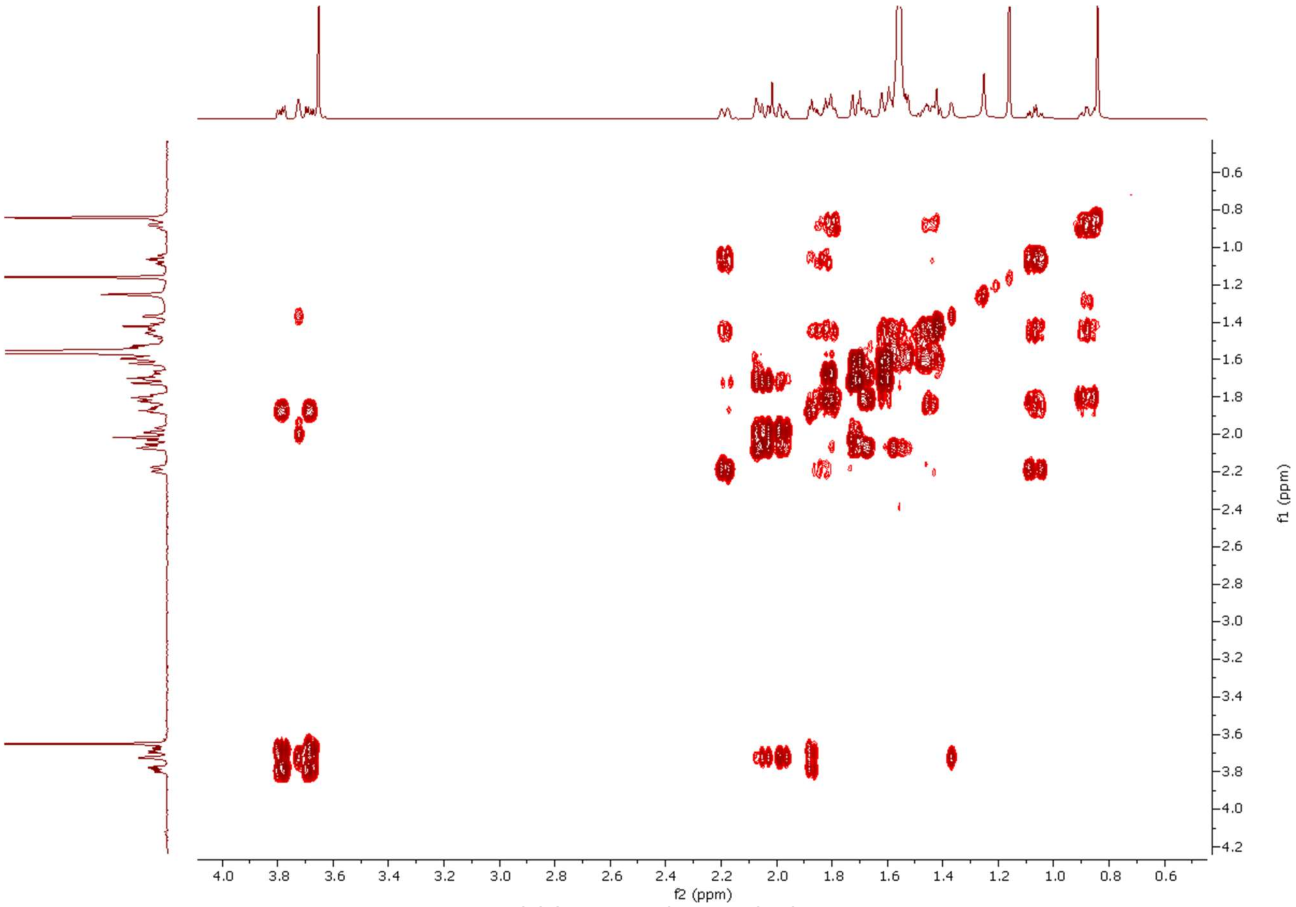

Figure N26a.B COSY NMR of **26a** in $CDCl_3$ at 600 MHz.

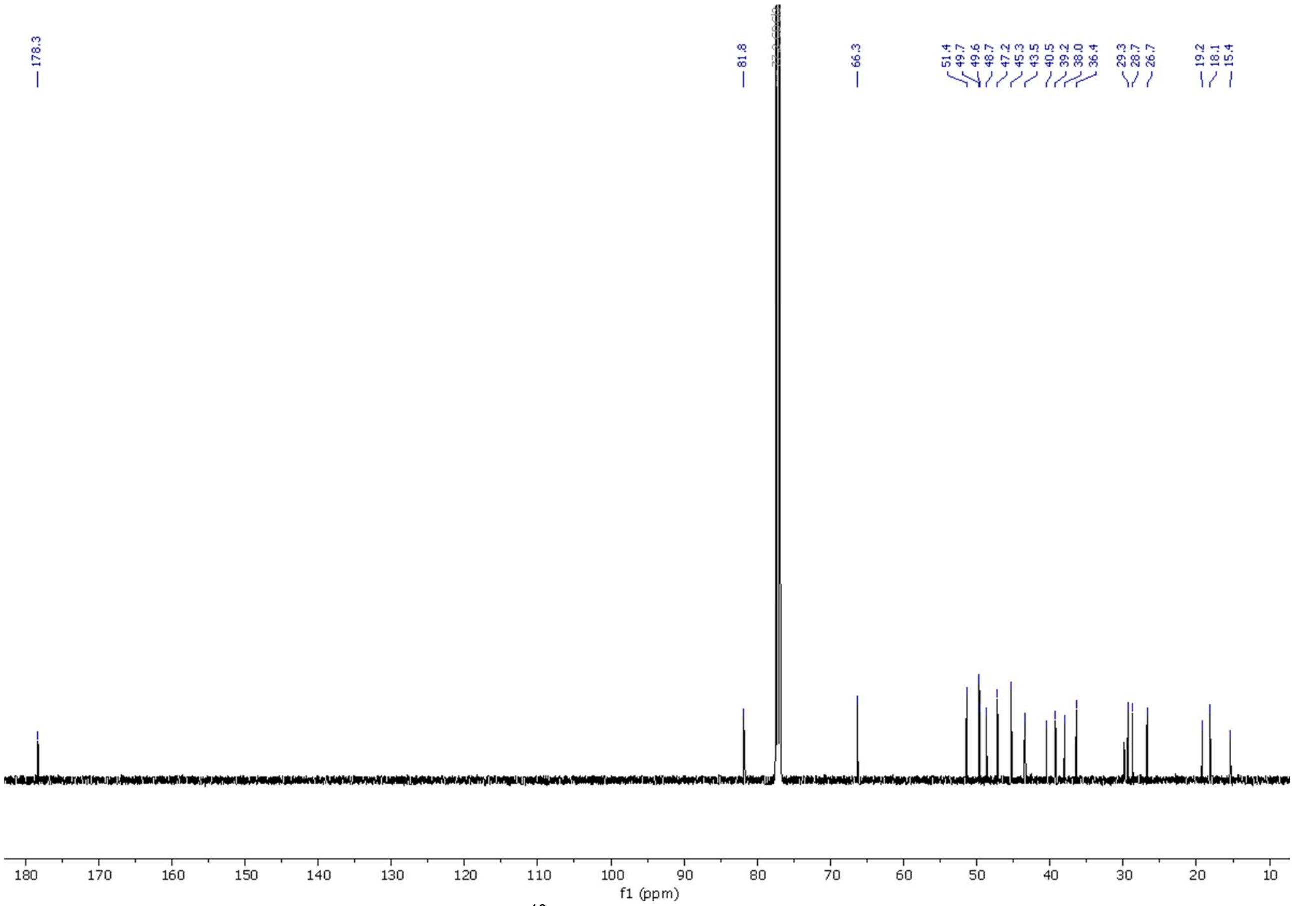

Figure N26a.C ^{13}C NMR of **26a** in $CDCl_3$ at 126 MHz.

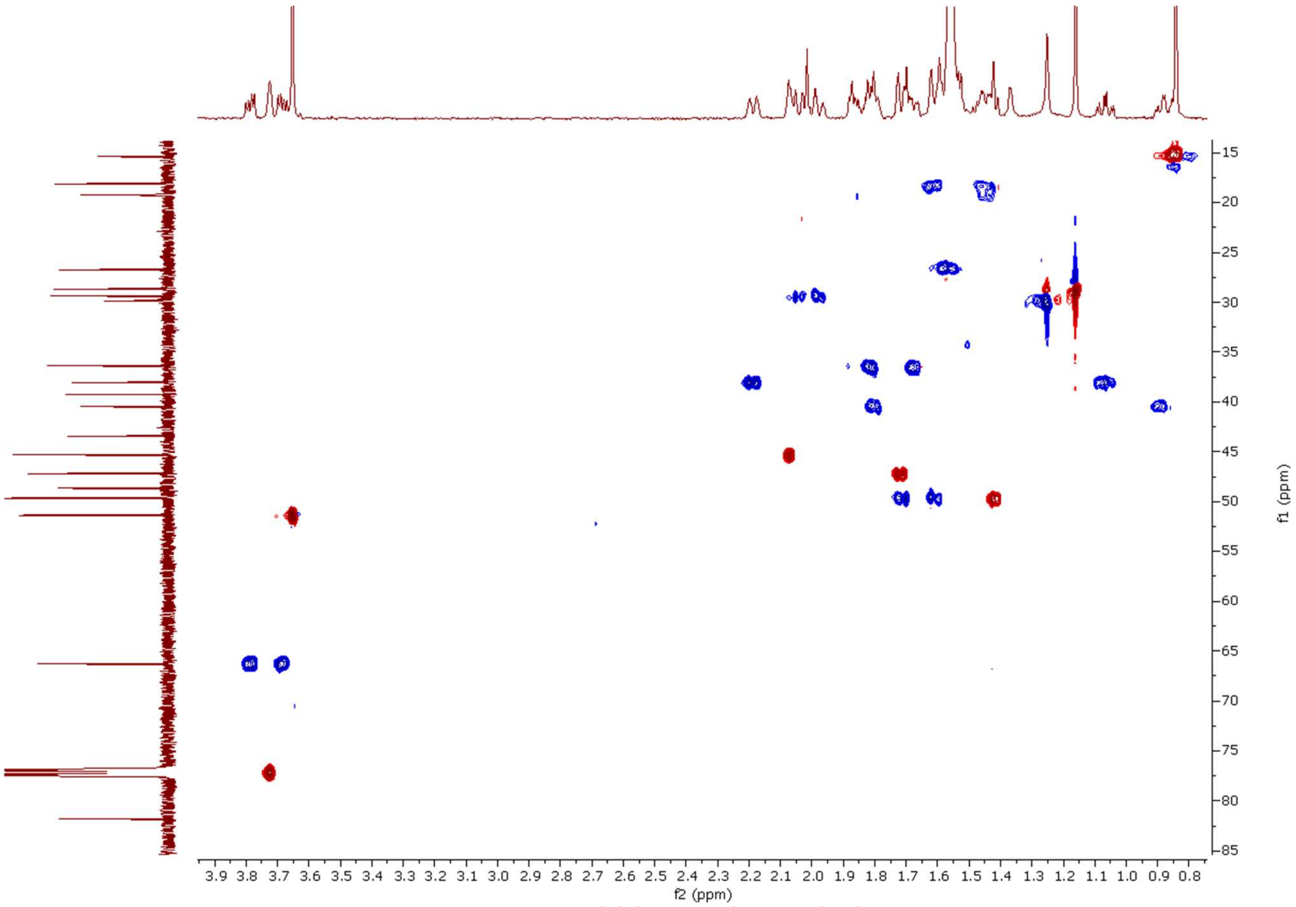

Figure N26a.D HSQC NMR of **26a** in $CDCl_3$.

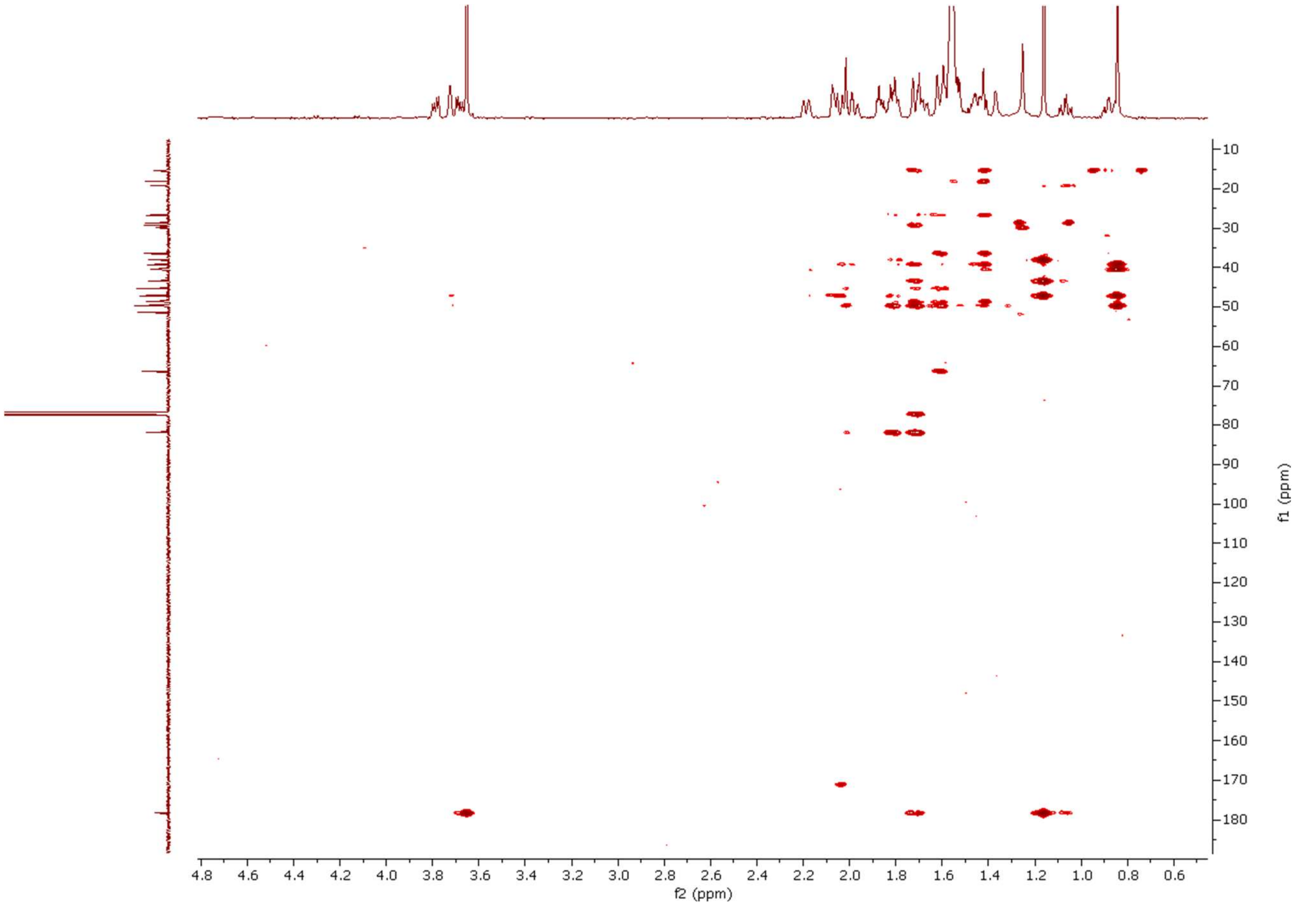

Figure N26a.E HMBC NMR of **26a** in $CDCl_3$.

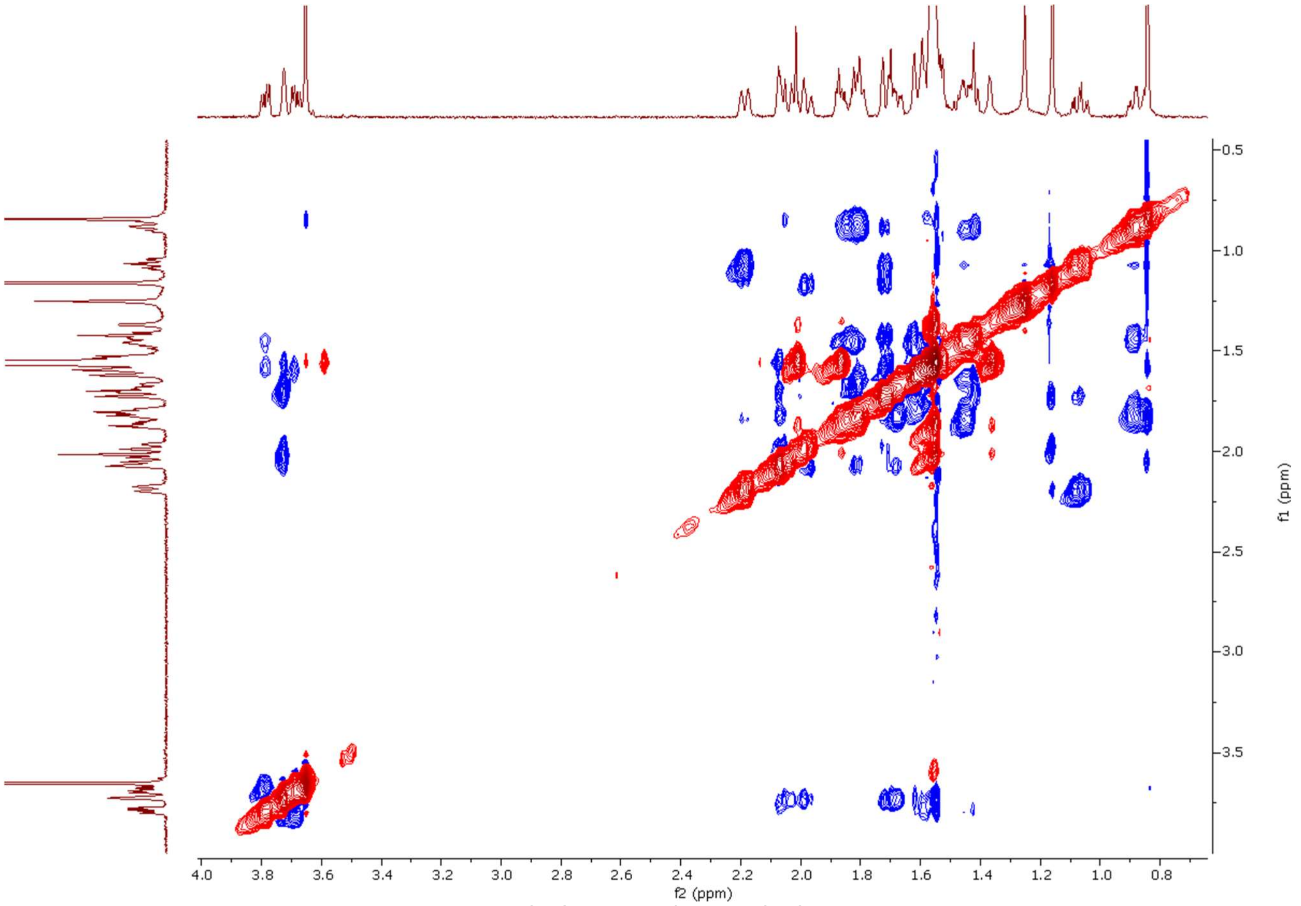

Figure N26a.F NOESY NMR of **26a** in $CDCl_3$ at 600 MHz.

6β,7β,16α-Trihydroxy-*ent*-kauranoic acid (27)

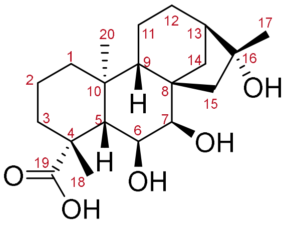

6β,7β,16α-Trihydroxy-*ent*-kauranoic acid (**27**)
Chemical Formula: $C_{20}H_{32}O_5$
Exact Mass: 352.2250

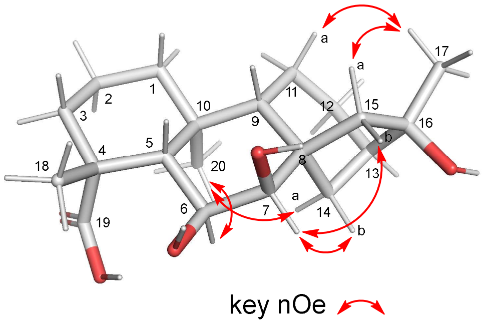

	Measured in DMSO-d_6, 298K			Measured in C_5D_5N, 298K		
**Pos.**	δ_C 151 MHz	δ_H (J/Hz) 500 MHz	**Selected NOESY correlations to H** [a]	δ_C 151 MHz	δ_H (J/Hz) 600 MHz	**Selected NOESY correlations to H** [a]
**1**	40.5	ax 0.72, ddd (13.3, 13.3, 3.6)<br>eq 1.69, overlapped	H-1eq<br>H-1ax	41.6	ax 1.04, ddd (13.3, 13.3, 3.6)<br>eq 1.91, overlapped	H-1eq<br>H-1ax
**2**	18.9	a 1.27, m<br>b 1.73, overlapped		20.2	a 1.53, overlapped<br>b 2.27, overlapped	H-2b<br>H-2a
**3**	39.8	ax 0.92, overlapped<br>eq 1.97, ddd (13.2, 3.4, 3.4)		41.1	ax 1.26, overlapped<br>eq 2.54, br d (13.2)	H-3eq<br>H-3ax
**4**	43.4	-	-	45.0	-	-
**5**	50.5	1.66, d (10.9)		52.4	2.43, d (10.9)	
**6**	70.6	ax 4.07, br d (10.9)	H_3-20, H-14b	72.4	4.98, br d (10.9)	H_3-20
**7**	80.8	3.30, overlapped		83.0	4.18, br s	H-15b, H-14b
**8**	48.9	-	-	50.4	-	-
**9**	48.9	1.21, overlapped	H-5	50.5	1.83, d (6.5)	
**10**	40.2	-	-	41.5	-	-
**11**	17.5	a 1.40, overlapped<br>b 1.42, overlapped		18.8	a 1.62, overlapped<br>b 1.64, overlapped	
**12**	27.1	a 1.39, overlapped<br>b 1.46, overlapped		28.2	a 1.55, overlapped<br>b 1.65, overlapped	
**13**	47.5	1.73, overlapped		49.5	2.19, br s	
**14**	36.1	a 1.60, d (11.1)<br>b 1.64, overlapped		37.6	a 1.99, d (11.1)<br>b 2.26, overlapped	H_3-20, H-14b<br>H-7, H-14a
**15**	54.3	a 1.35, d (14.5)<br>b 1.78, d (14.5)	H-15b<br>H-15a	56.1	a 2.07, d (14.5)<br>b 2.70, d (14.5)	H-15b<br>H-15a
**16**	76.4	-	-	77.9	-	-
**17**	24.4	1.21, s	H-11a, H-15a	25.4	1.62, s	H-15a, H-13
**18**	32.6	1.31, s		33.7	1.89, s	
**19**	179.6 [HMBC]	-	-	181.9	-	-
**20**	16.6	0.90, s	H-1eq	17.6	1.32, s	

[a] Key NOESY correlations are shown in blue text.

[HMBC] = detected based on HMBC.

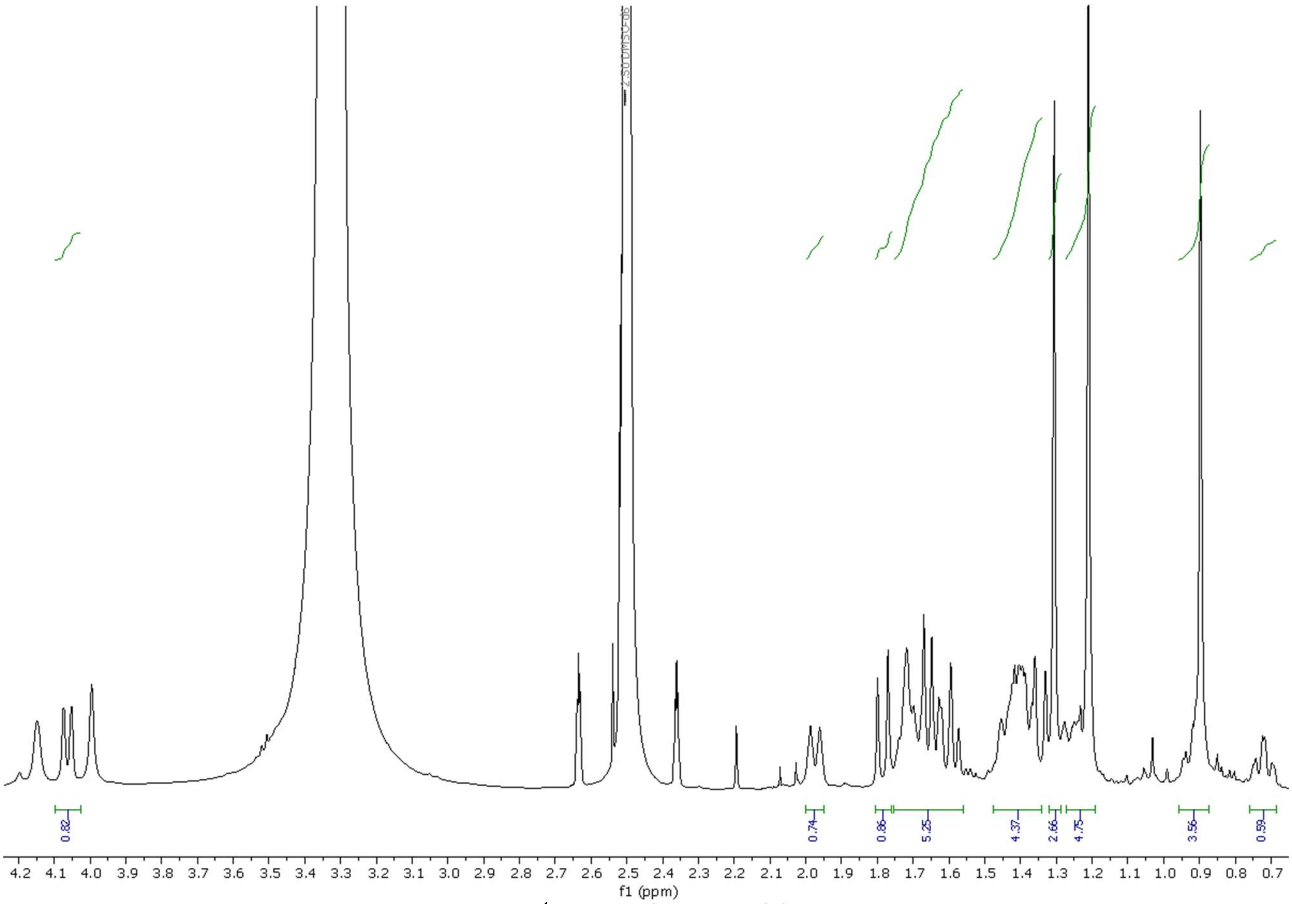

Figure N27.A ^{1}H NMR of **27** in DMSO-d_6 at 500 MHz.

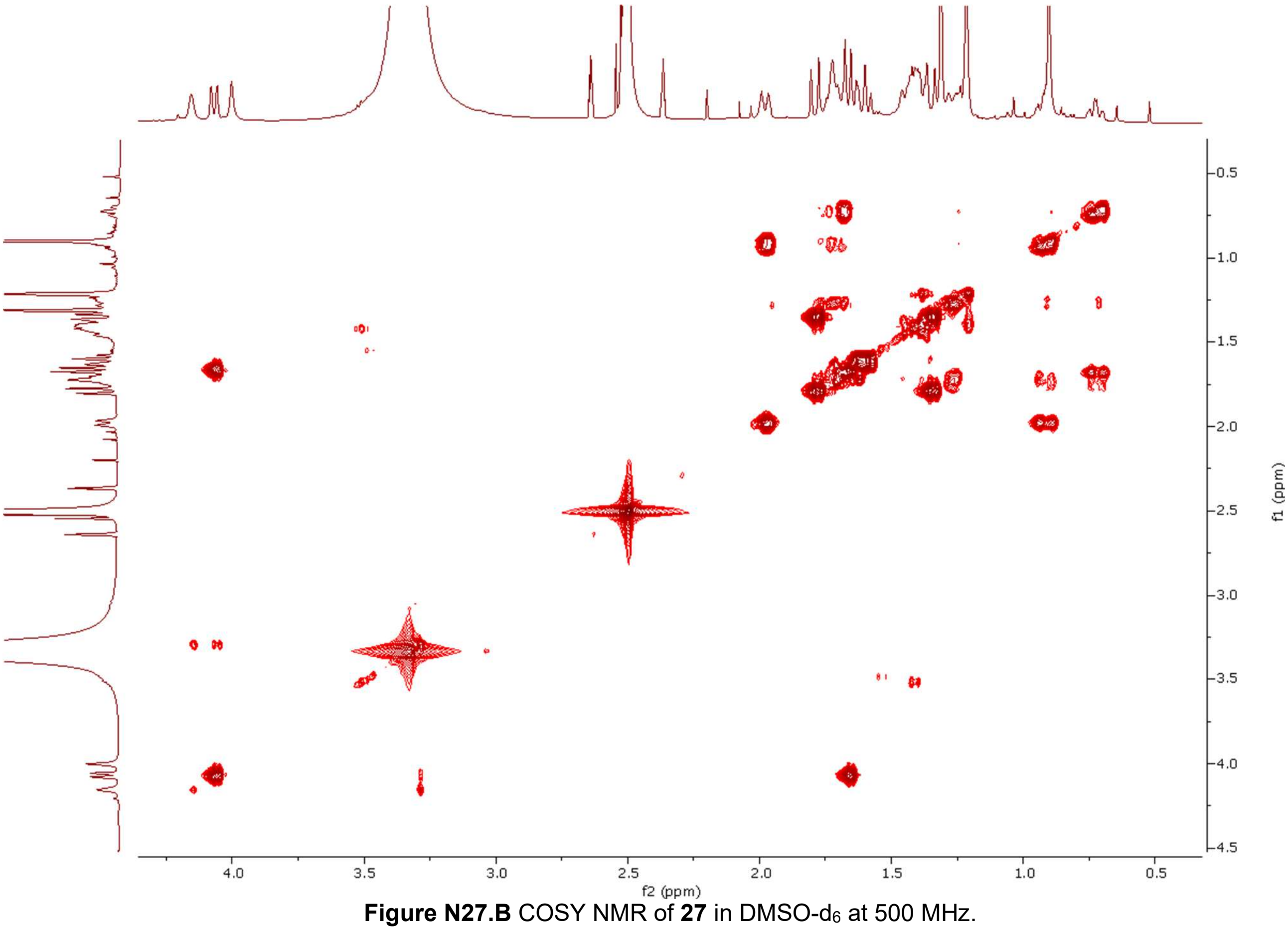

Figure N27.B COSY NMR of **27** in DMSO-d_6 at 500 MHz.

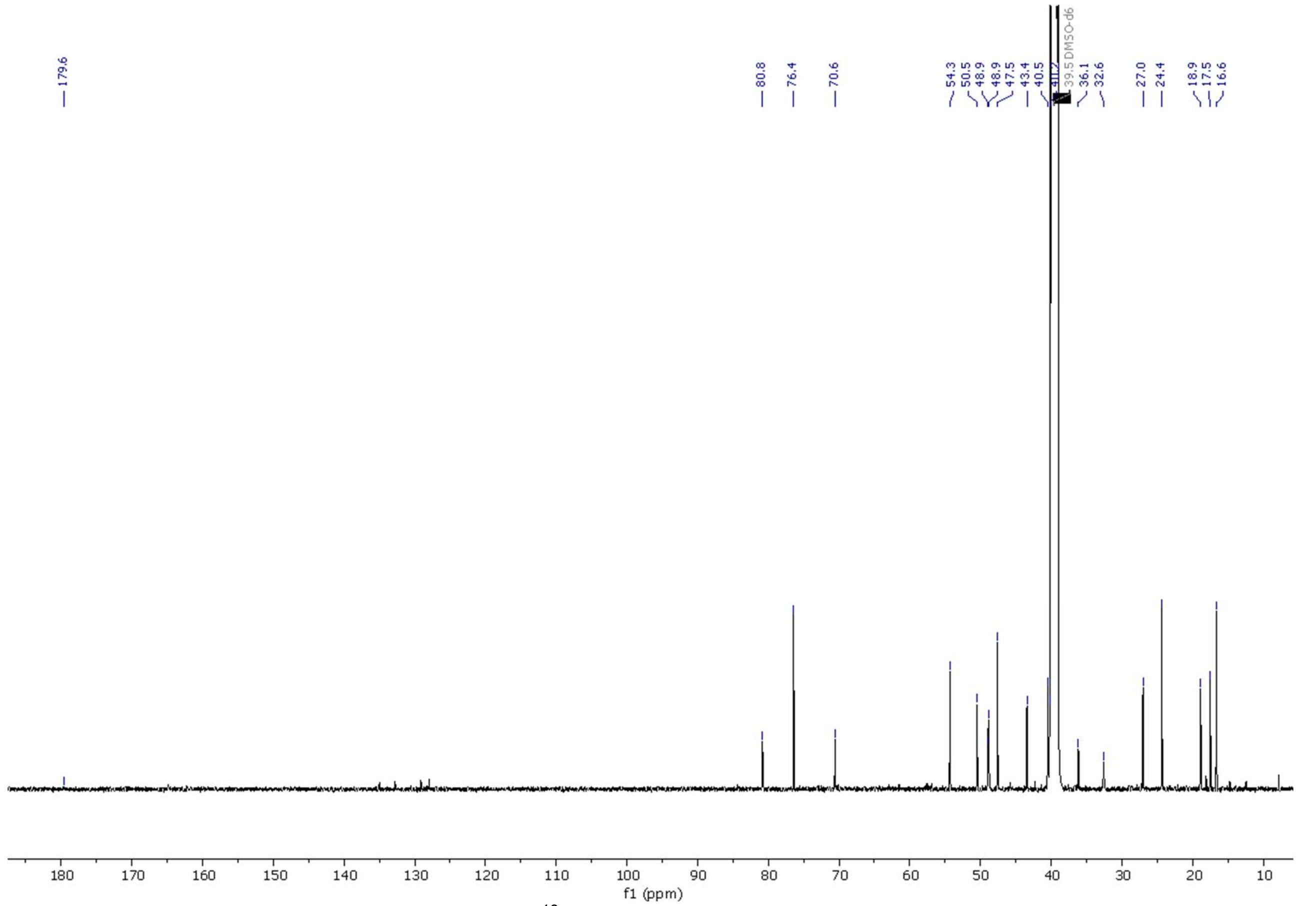

Figure N27.C ^{13}C NMR of **27** in DMSO-d_6 at 151 MHz.

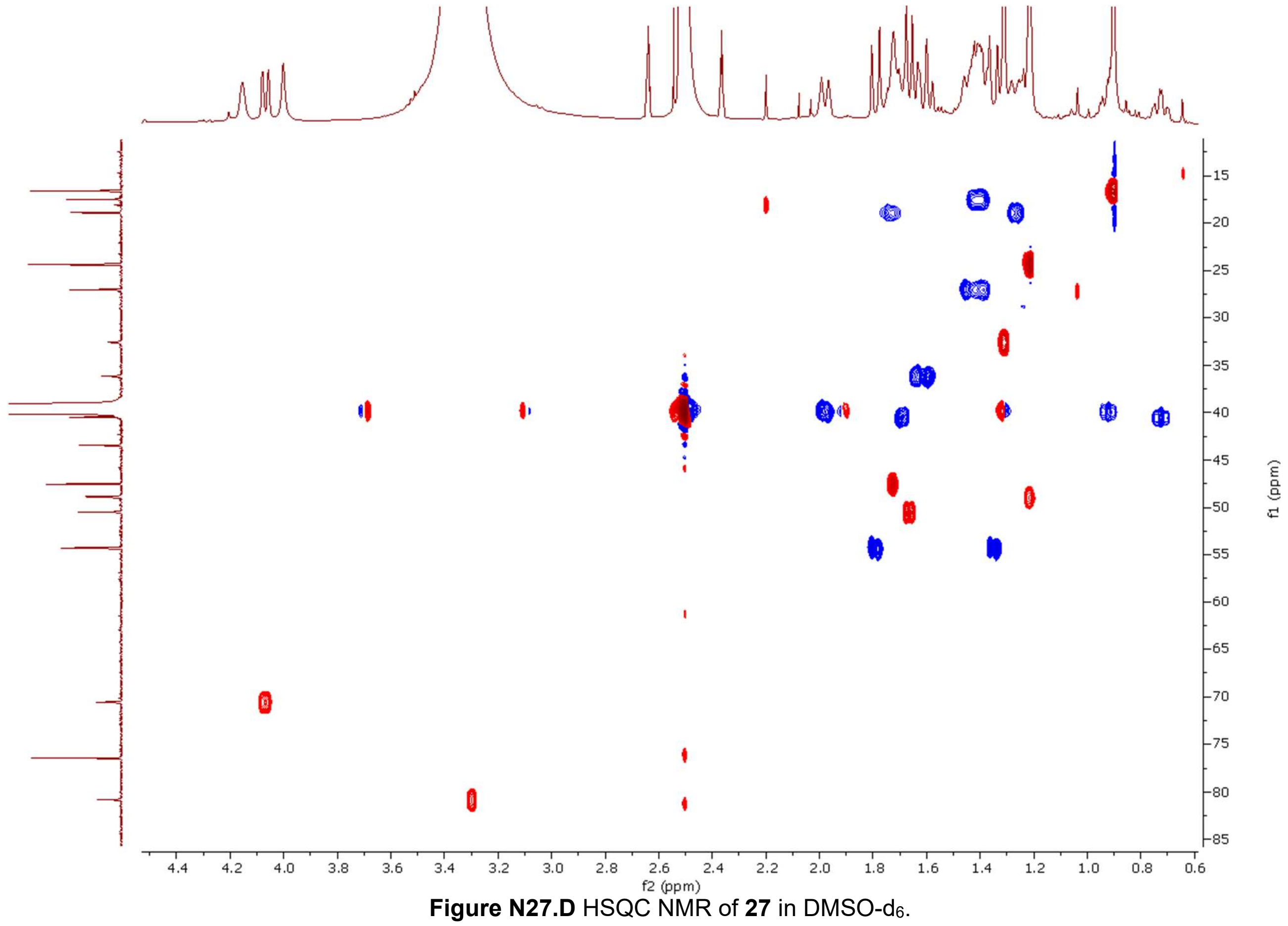

Figure N27.D HSQC NMR of **27** in DMSO-d_6.

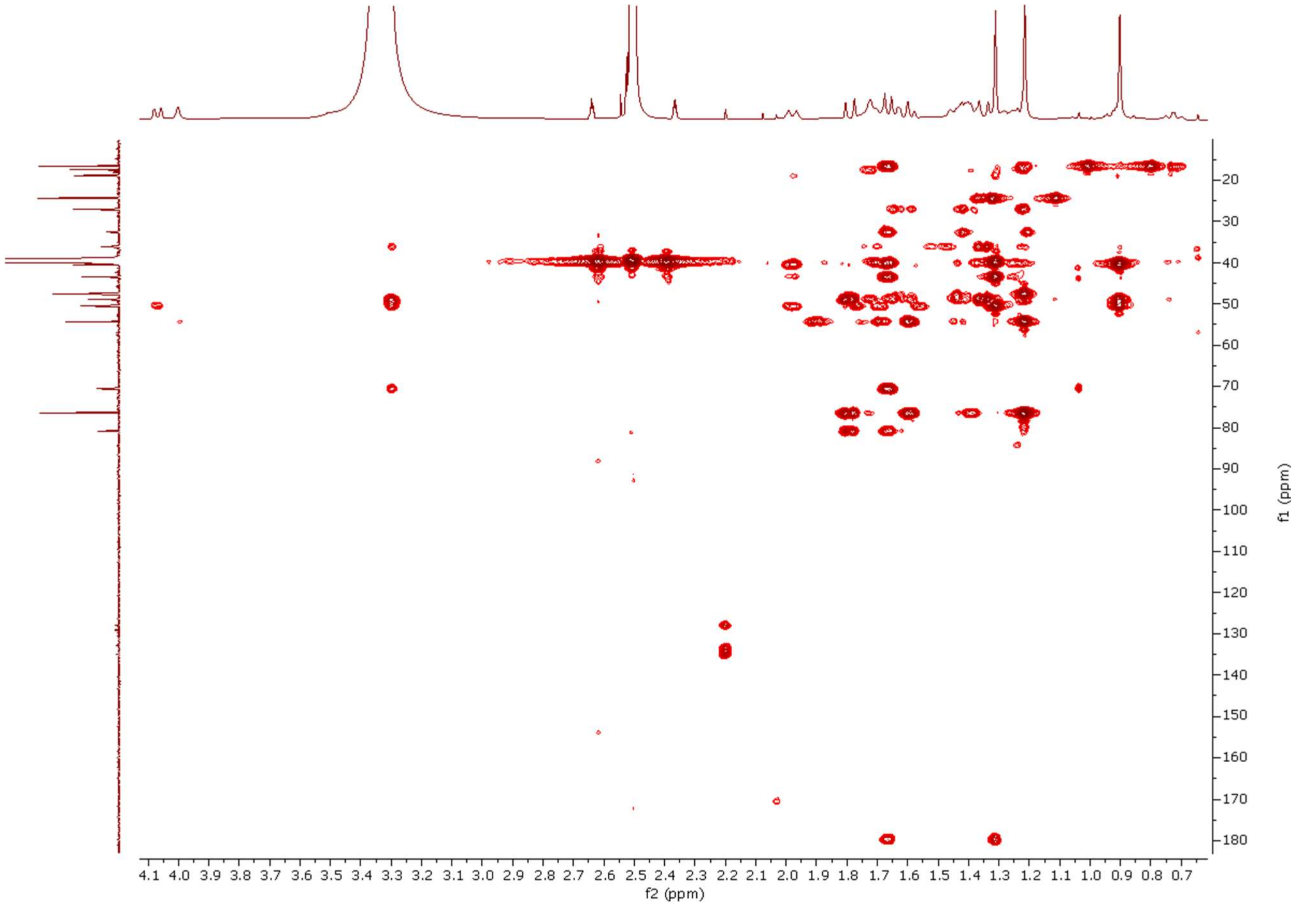

Figure N27.E HMBC NMR of **27** in DMSO-d6.

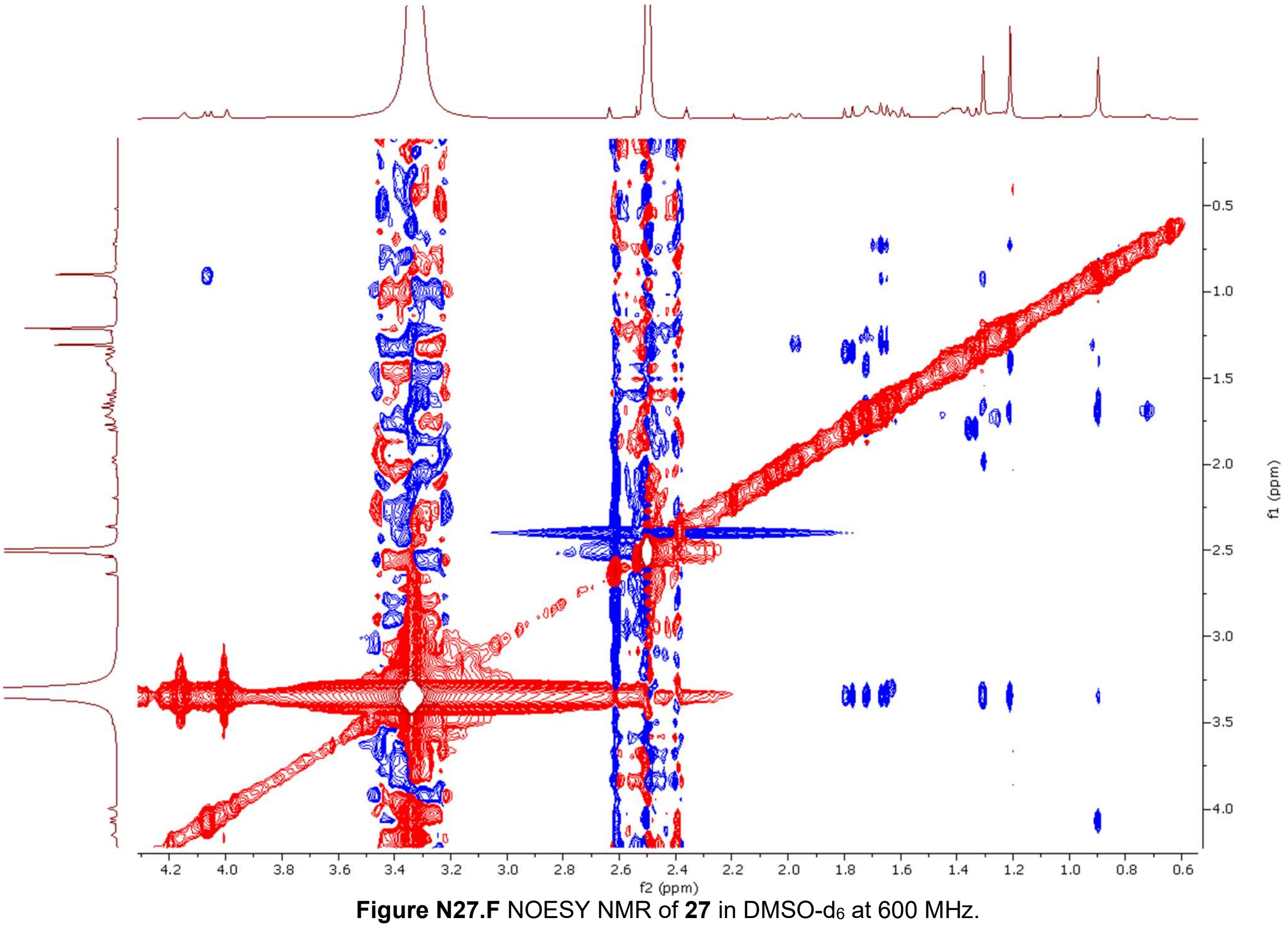

Figure N27.F NOESY NMR of **27** in DMSO-d6 at 600 MHz.

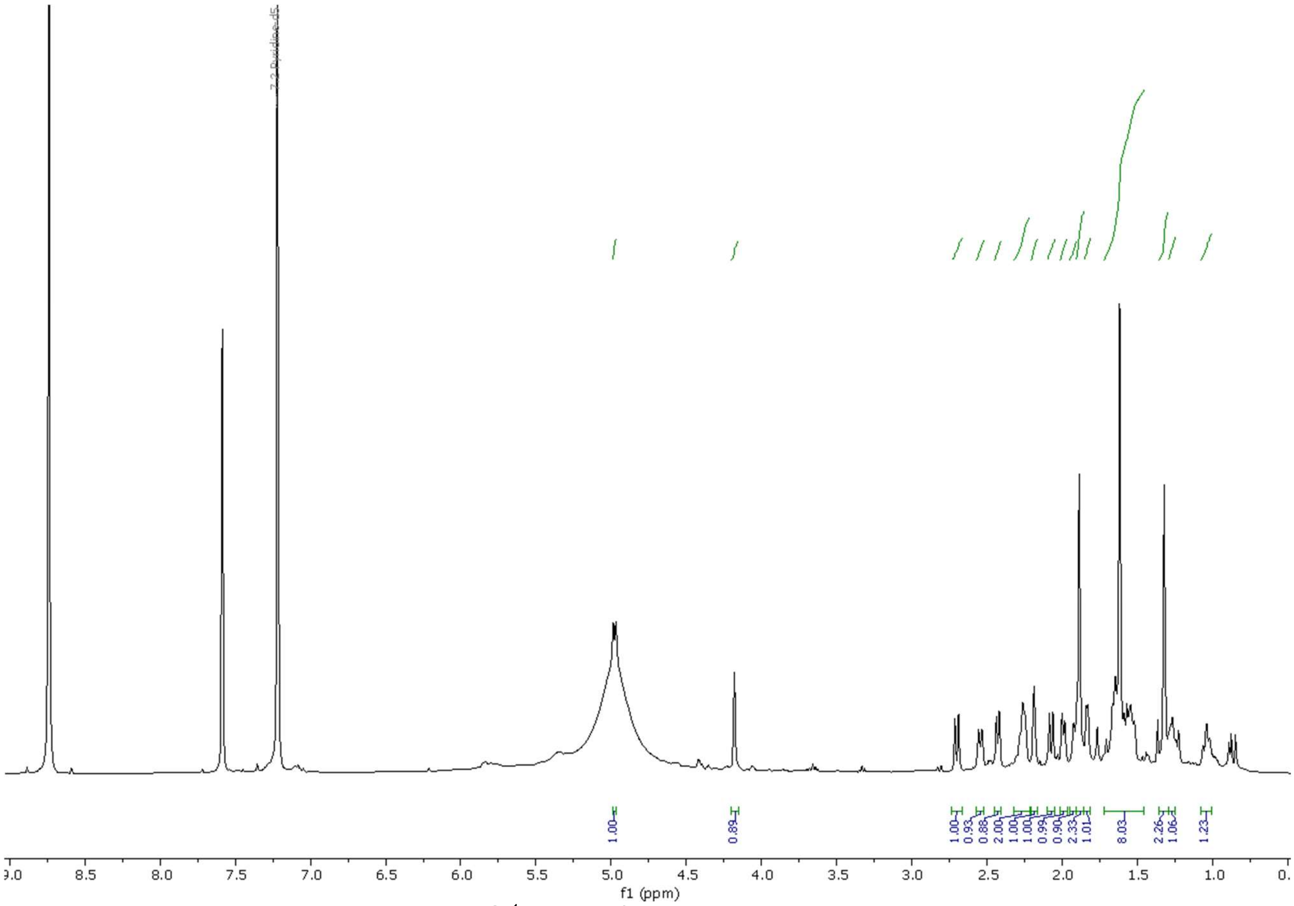

Figure N27.G ^{1}H NMR of **27** in pyridine-d_5 at 600 MHz.

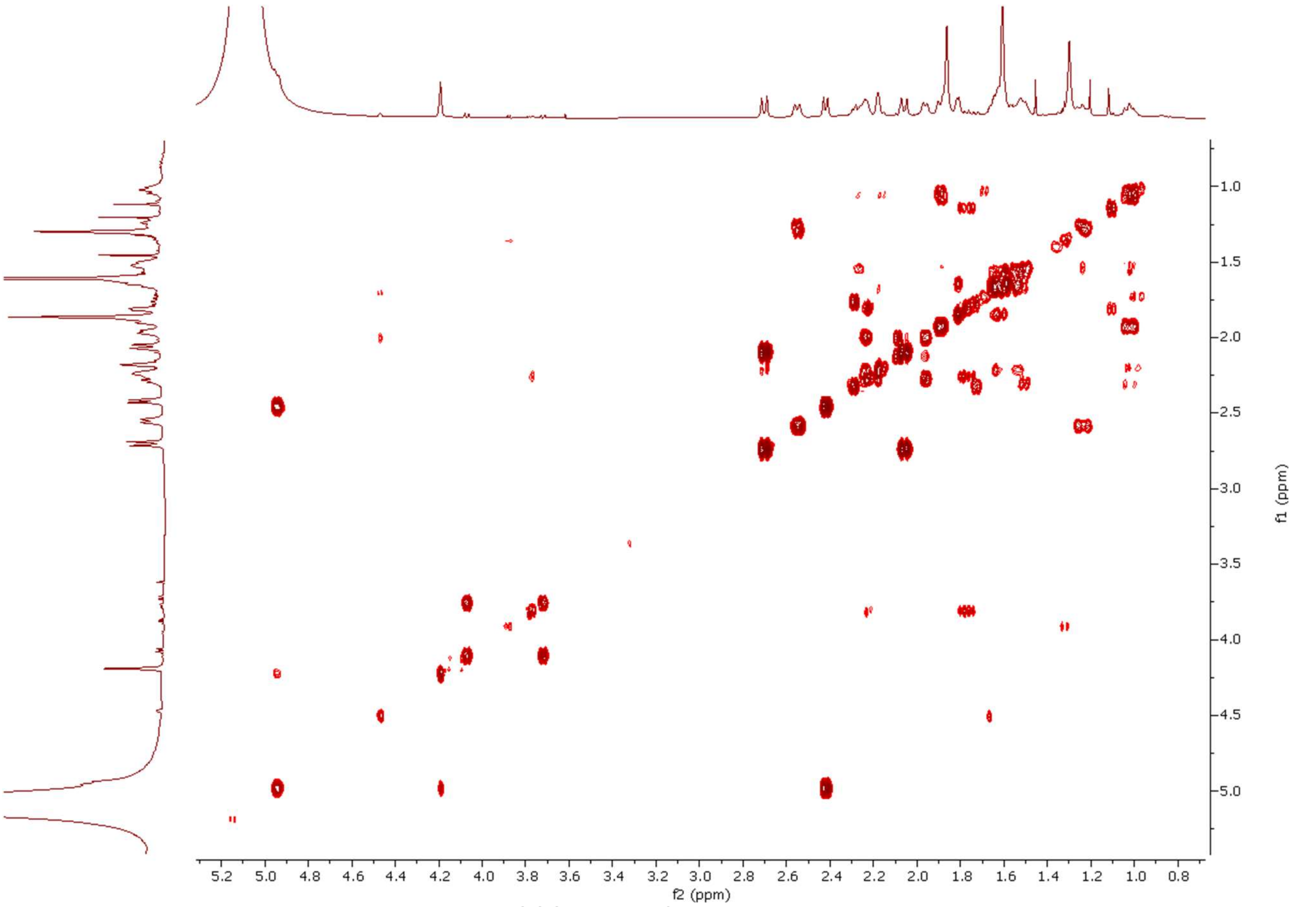

Figure N27.H COSY NMR of **27** in pyridine-d_5 at 600 MHz.

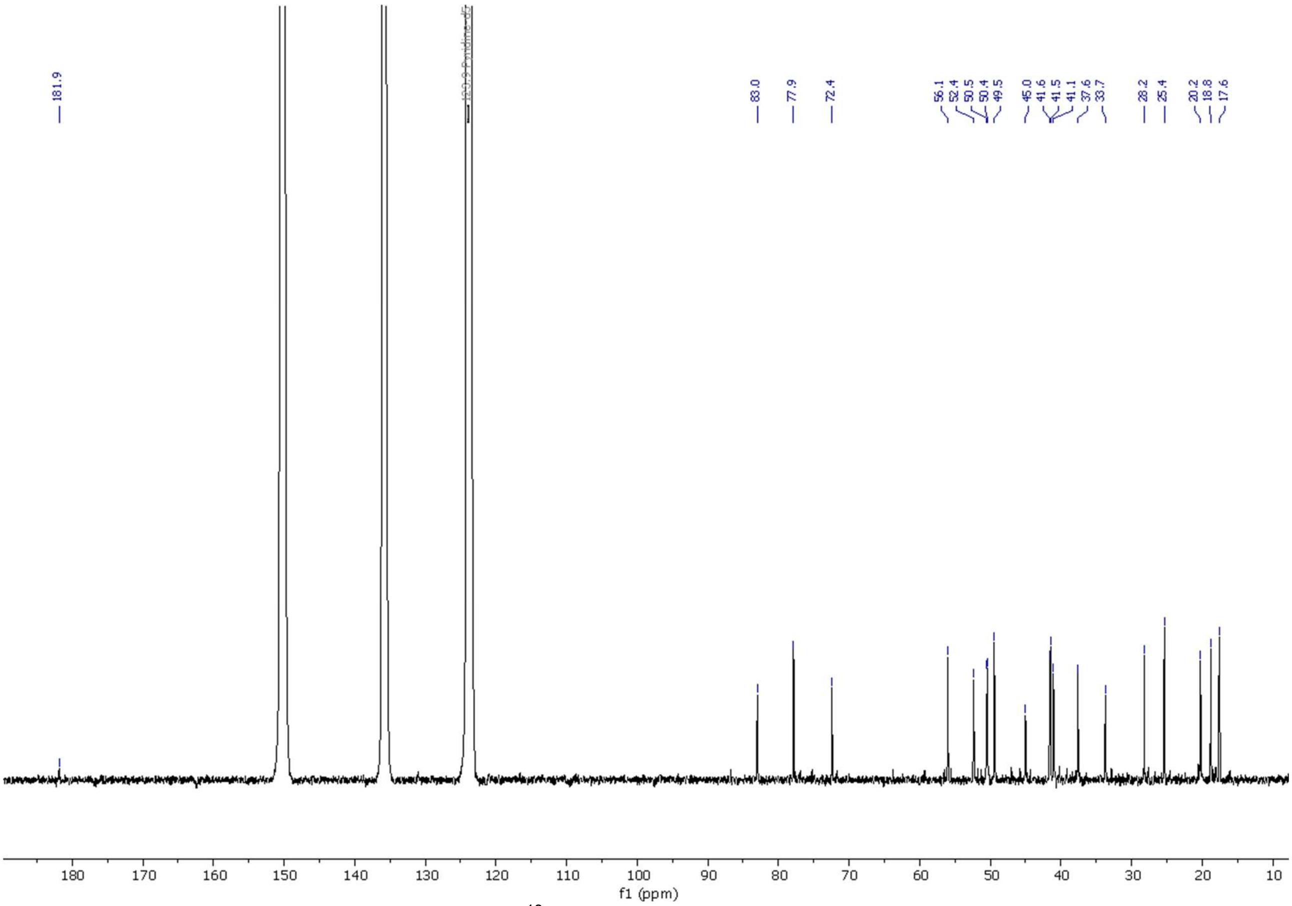

Figure N27.I ^{13}C NMR of **27** in pyridine-d$_5$ at 151 MHz.

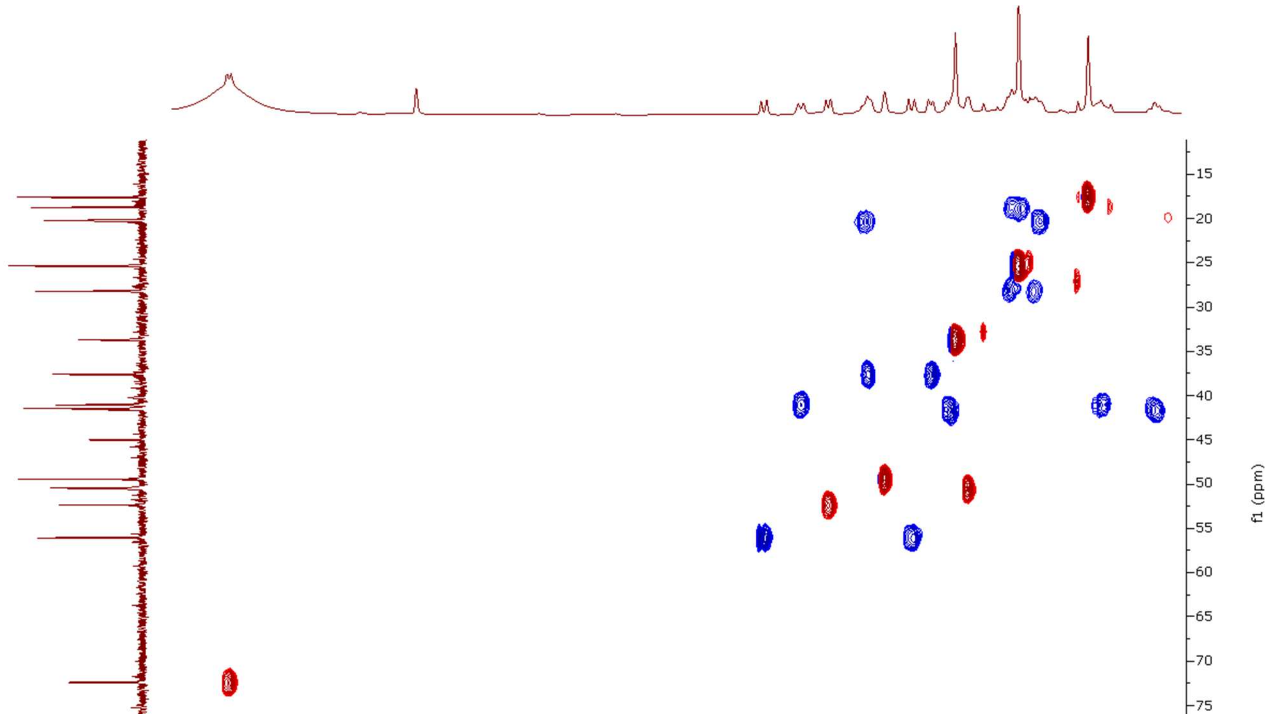

Figure N27.J HSQC NMR of **27** in pyridine-d$_5$.

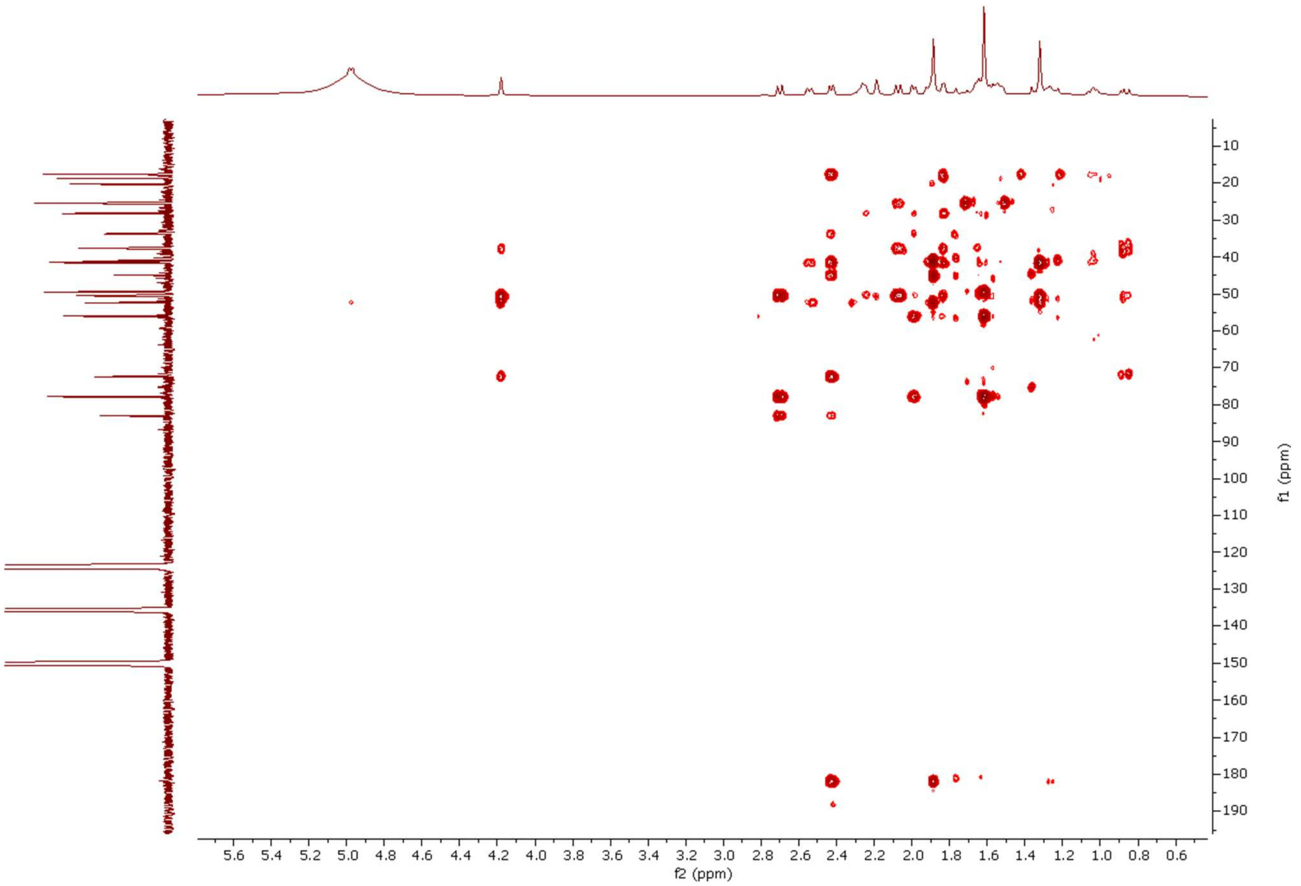

Figure N27.K HMBC NMR of **27** in pyridine-d$_5$.

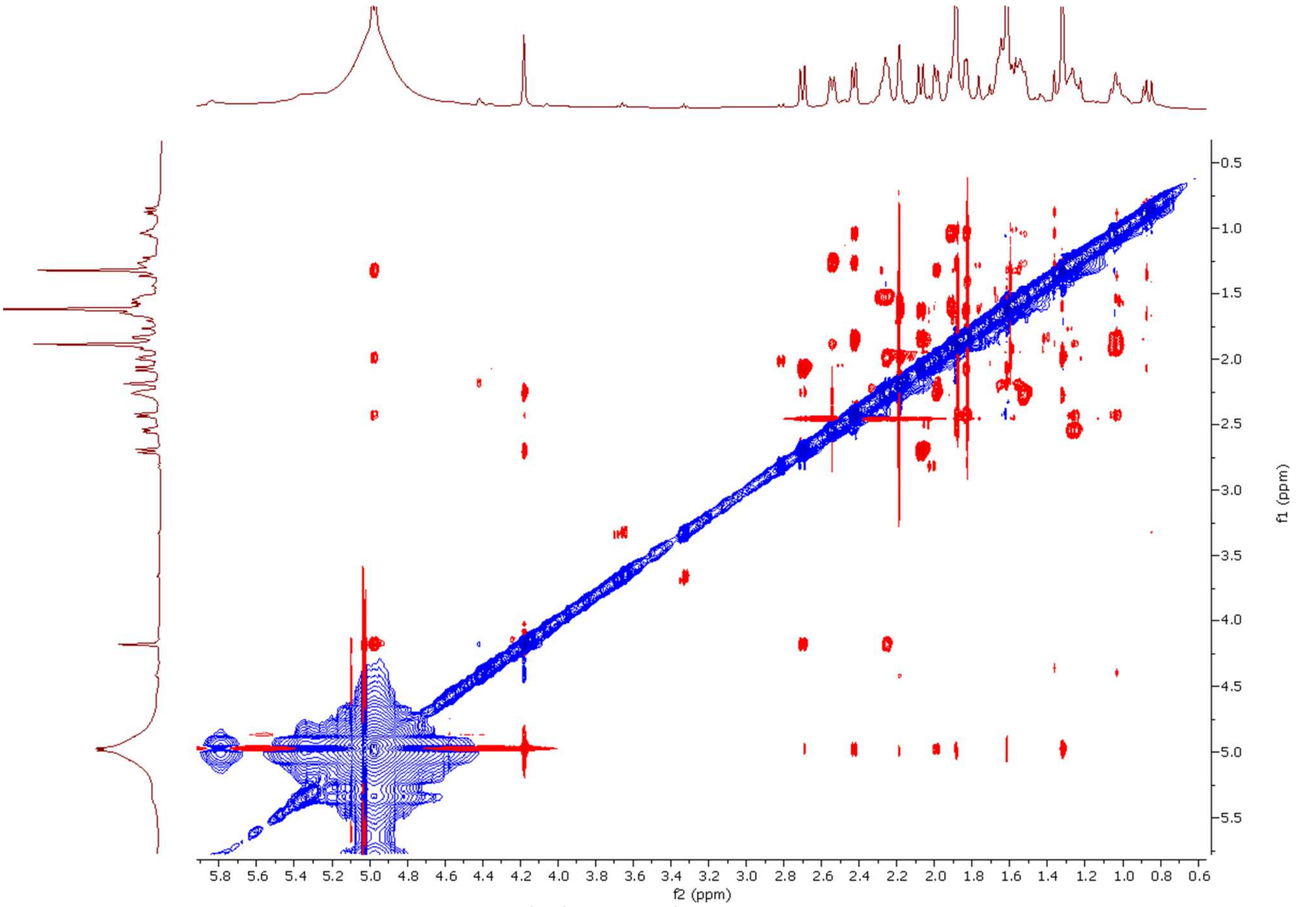

Figure N27.L NOESY NMR of **27** in pyridine-d$_5$ at 600 MHz.

1,10-Didehydro-20-nor-GA_{14} dimethyl ester (28a)

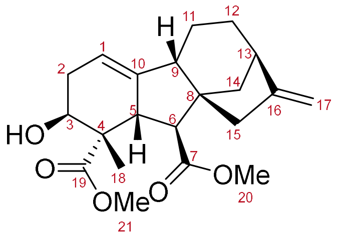

1,10-Didehydro-20-nor-GA_{14} dimethyl ester (**28a**)

Chemical Formula: $C_{21}H_{28}O_5$

Exact Mass: 360.1937

	**Reference in $CDCl_3$ [49] (only δ_H reported)**	**Measured in $CDCl_3$, 298K**	
**Pos.**	δ_H (J/Hz) 400 MHz	δ_C 151 MHz	δ_H (J/Hz) 600 MHz
**1**	5.25, q (2)	110.5	5.25, br s
**2**	n.r.	31.3	a 2.19, m<br>b 2.61, m
**3**	4.04, s	70.7	eq 4.03, ddd (6.0, 4.8, 4.8)
**4**	-	48.7	-
**5**	n.r.	47.2	3.05, dd (6.8, 3.0)
**6**	n.r.	50.5	2.80, d (6.8)
**7**	-	176.4	-
**8**	-	52.1	-
**9**	n.r.	47.3	2.55, m
**10**	-	142.1	-
**11**	n.r.	17.3	a 1.71, m<br>b 1.72, m
**12**	n.r.	31.2	a 1.45, m<br>b 1.58, m
**13**	n.r.	42.0	2.58, m
**14**	n.r.	42.1	a 1.31, m<br>b 1.54, m
**15**	n.r.	40.7	a 2.20, br d (16.0)<br>b 2.36, ddd (16.0, 2.7, 2.7)
**16**	-	154.0	-
**17**	4.89, br s<br>4.89, br s	106.3	a 4.89, br s<br>b 4.89, br s
**18**	1.31, s	20.6	1.31, s
**19**	-	175.6	-
**20**	3.70, s	51.6	3.70, s
**21**	3.64, s	51.7	3.63, s
**3-OH**	n.r.	-	1.98, d (6.0)

n.r. = not reported.

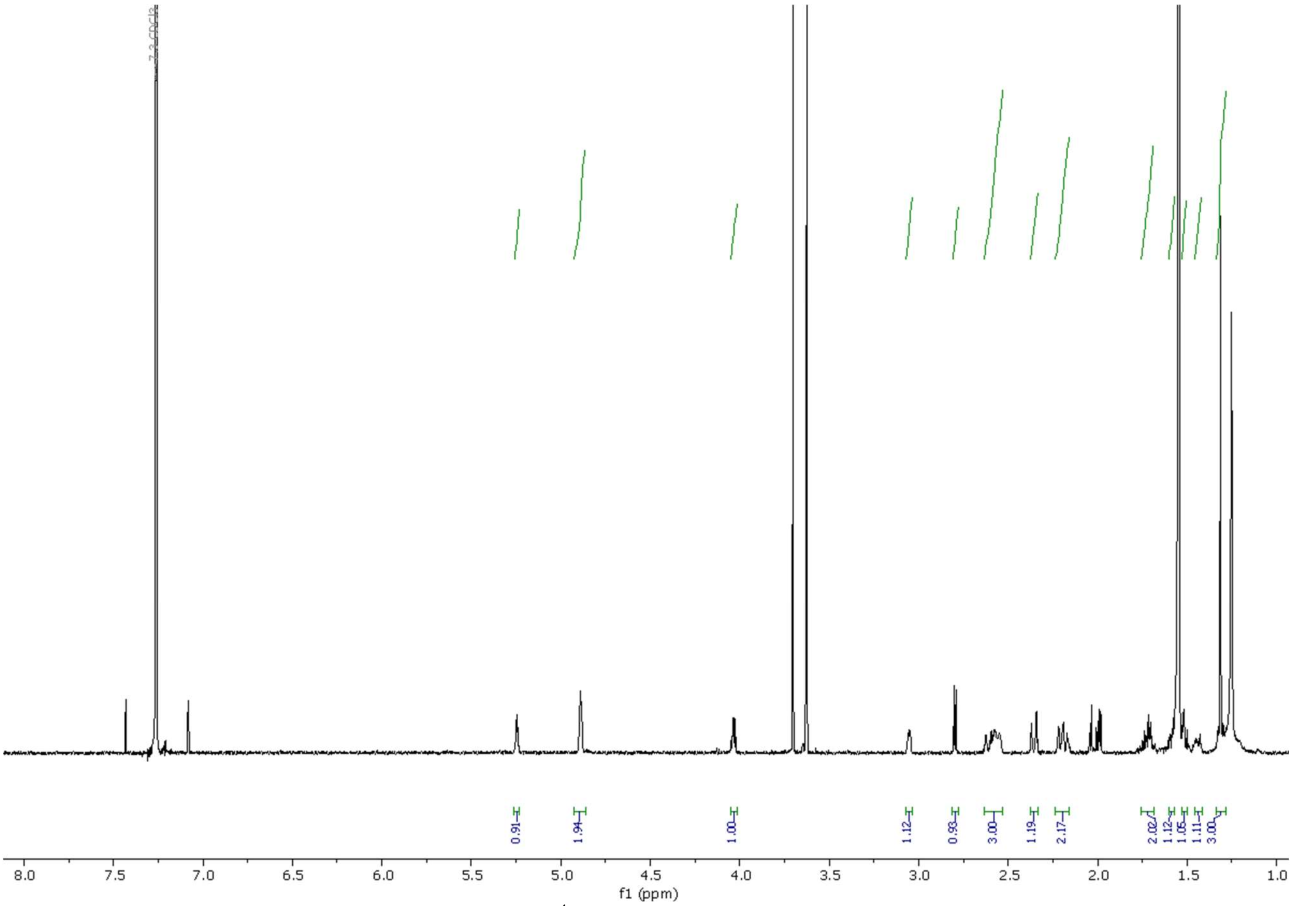

Figure N28a.A ^{1}H NMR of **28a** in $CDCl_3$ at 600 MHz.

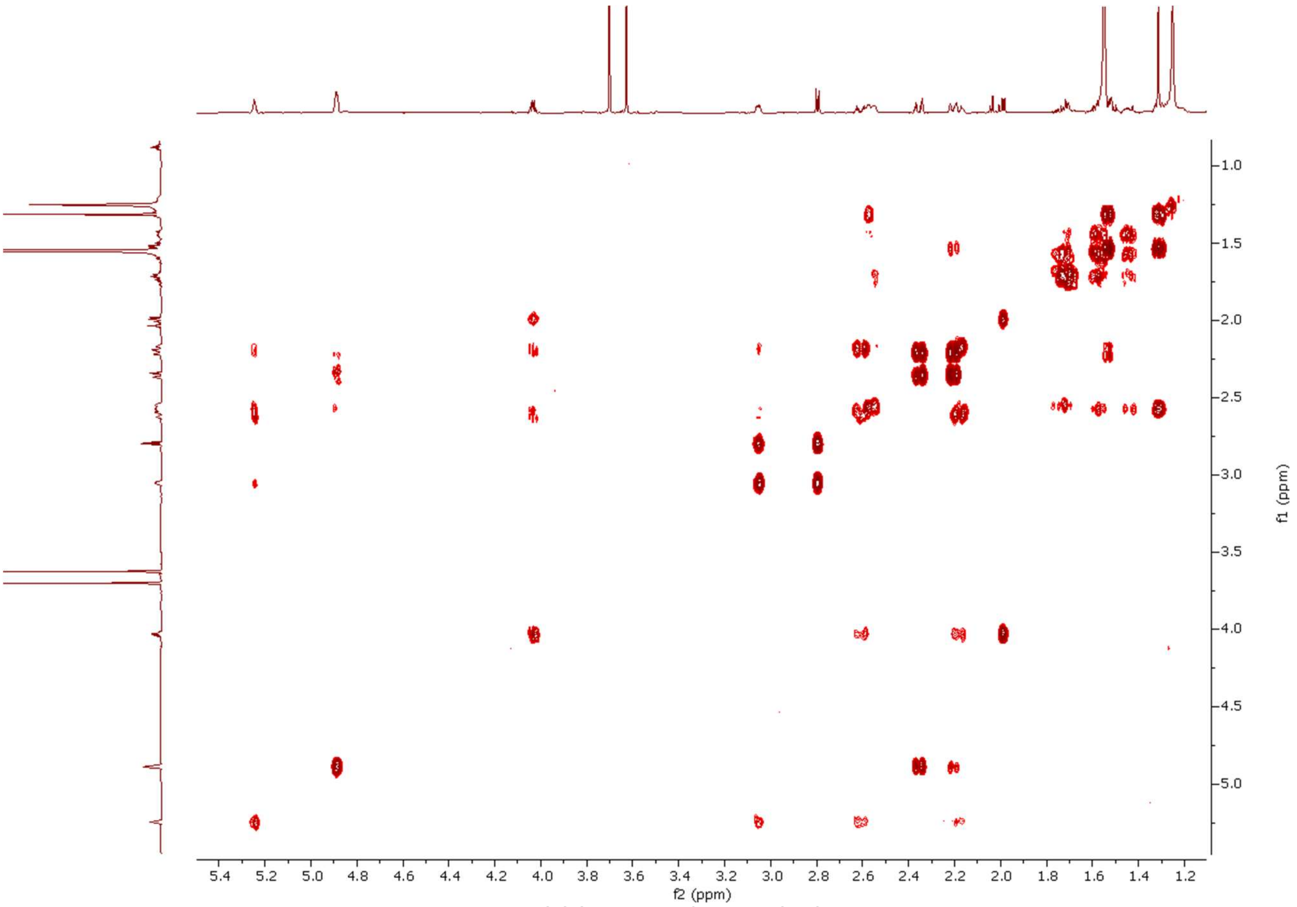

Figure N28a.B COSY NMR of **28a** in $CDCl_3$ at 600 MHz.

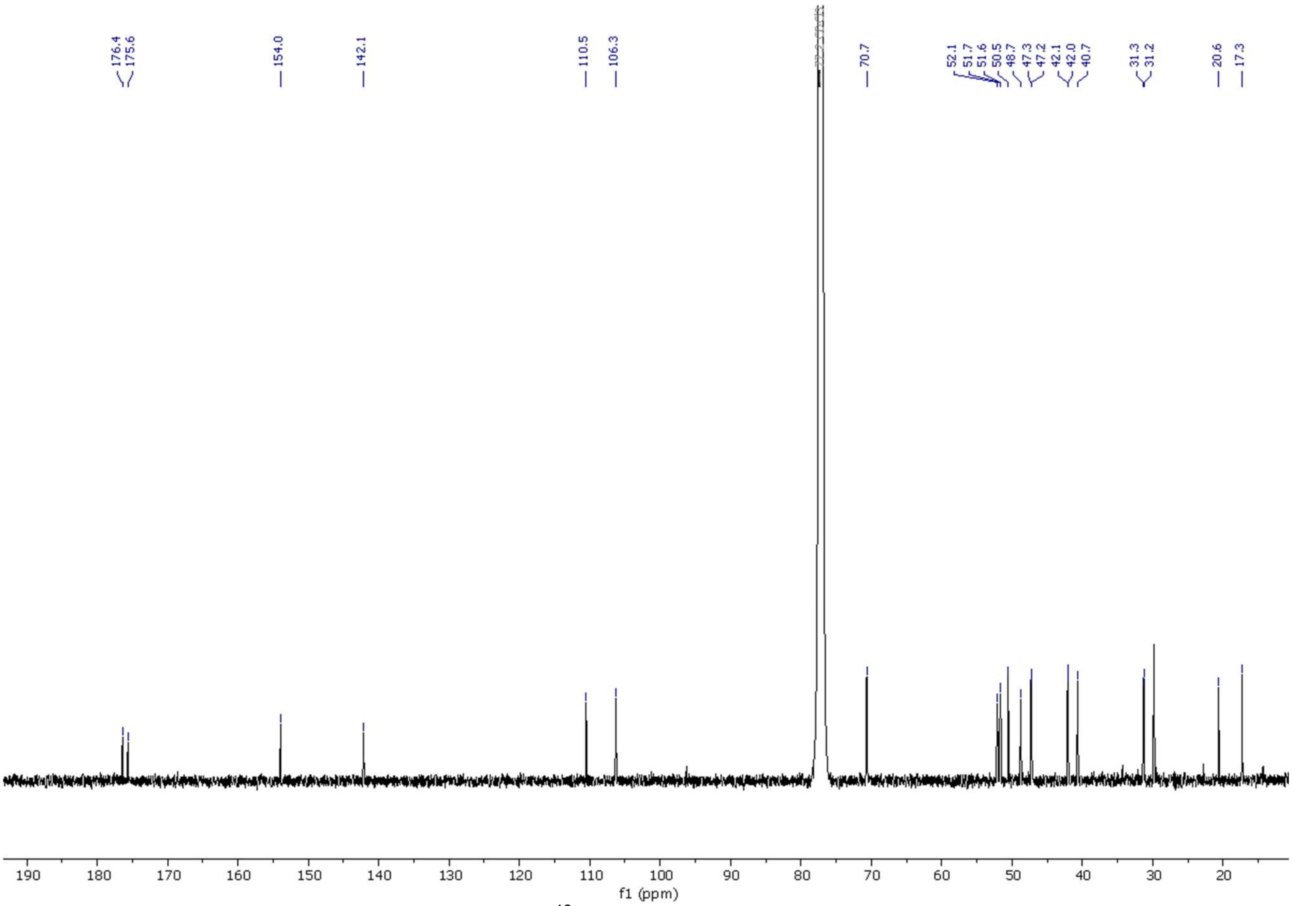

Figure N28a.C ^{13}C NMR of **28a** in $CDCl_3$ at 151 MHz.

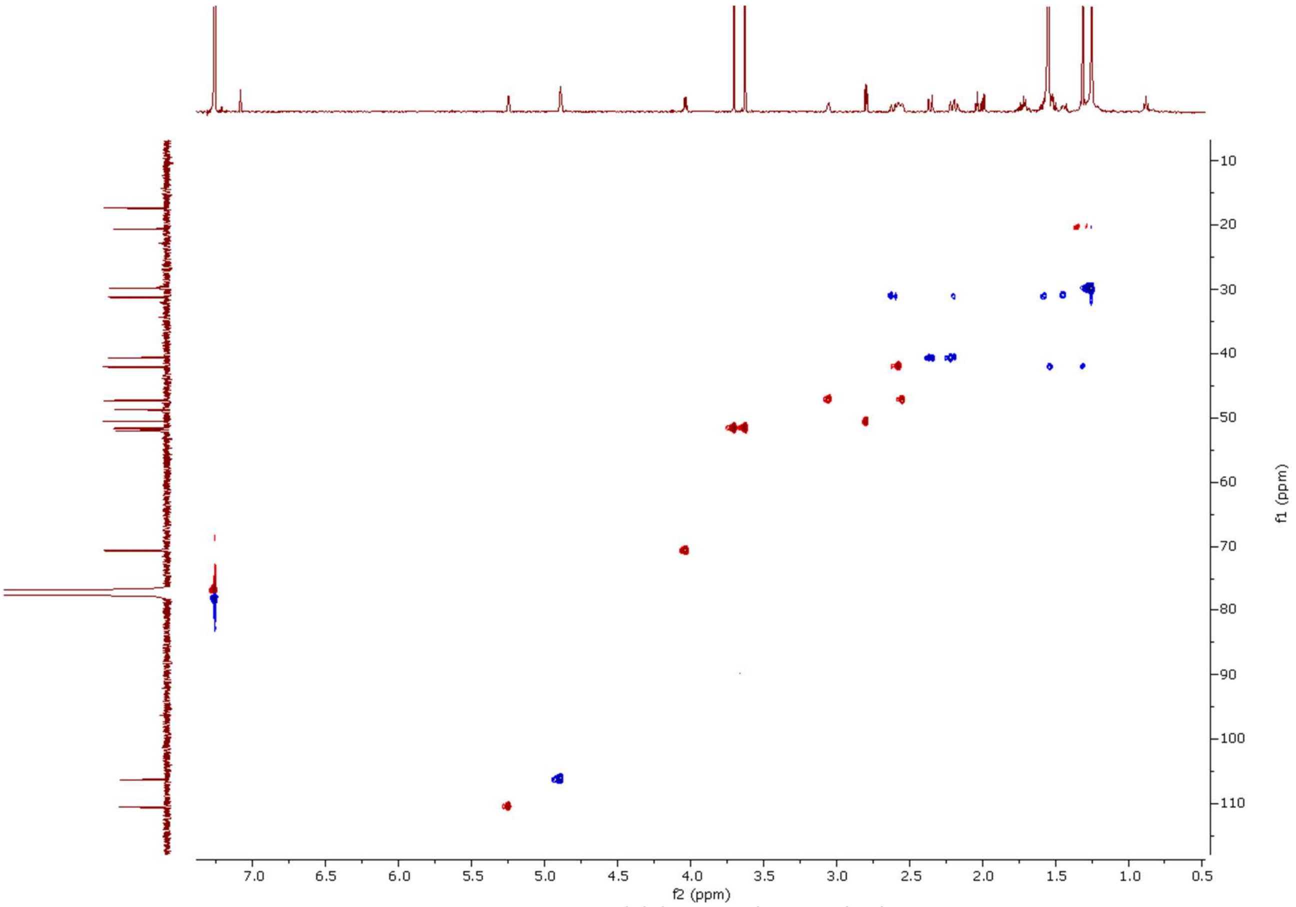

Figure N28a.D HSQC NMR of **28a** in $CDCl_3$.

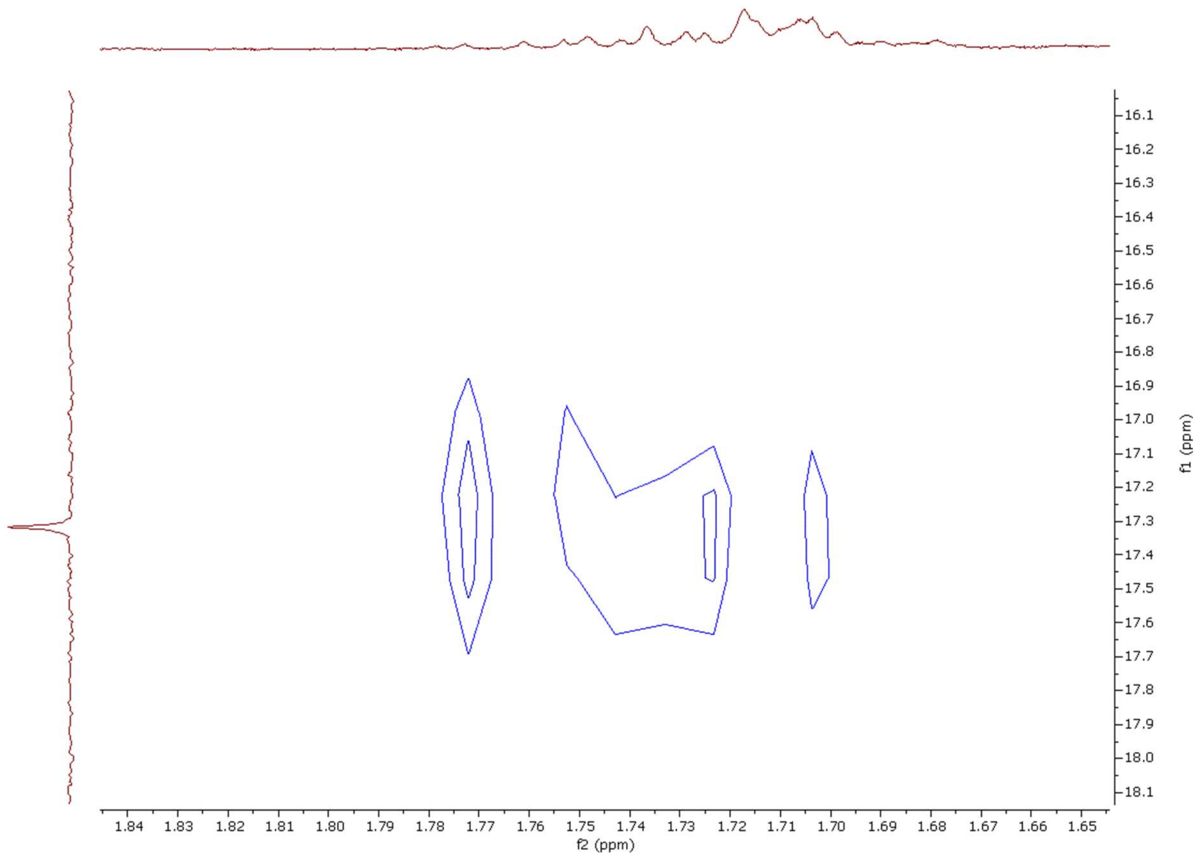

Figure N28a.E HSQC (zoomed in) NMR of **28a** in $CDCl_3$.

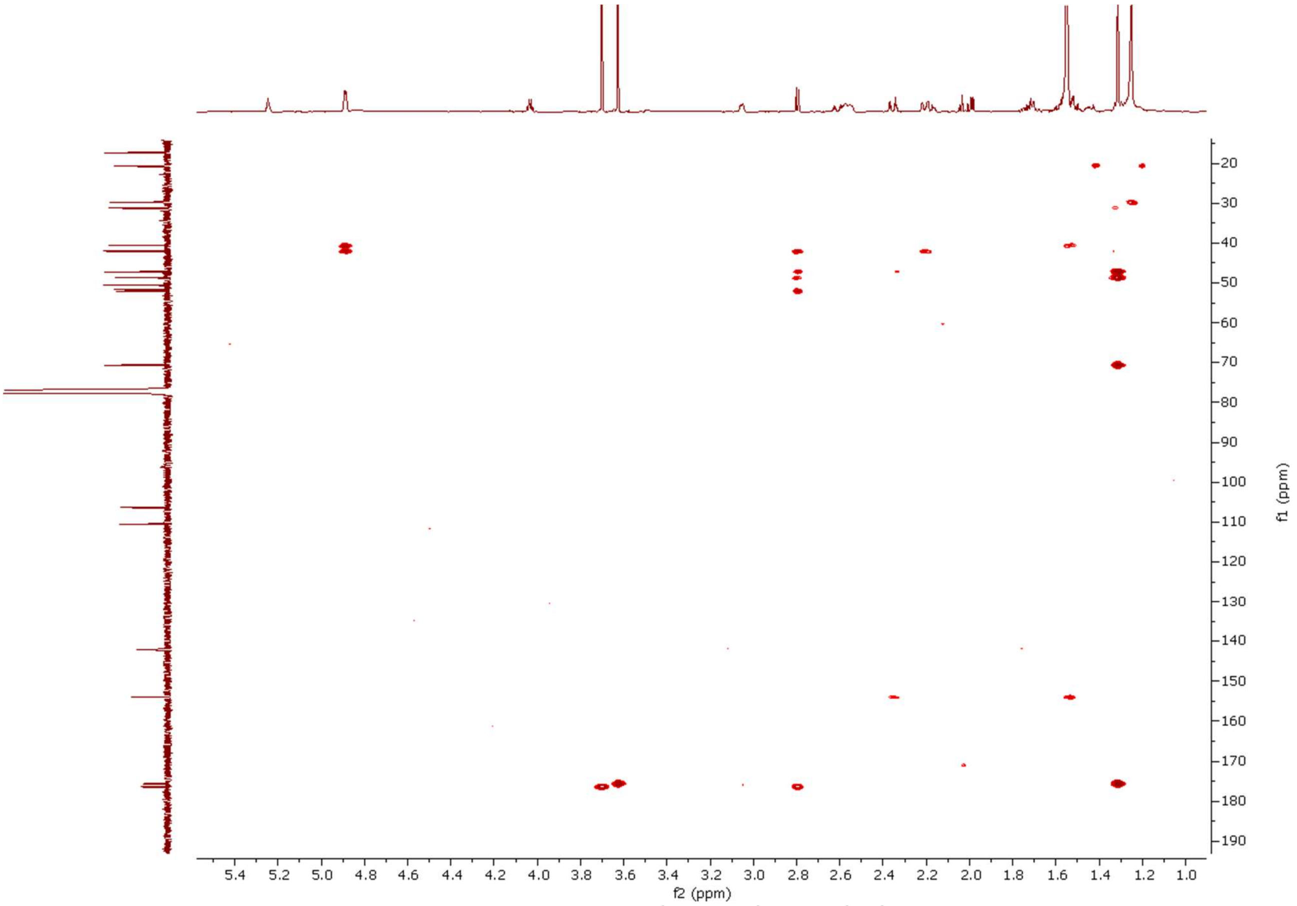

Figure N28a.F HMBC NMR of **28a** in $CDCl_3$.

Steviol (29)

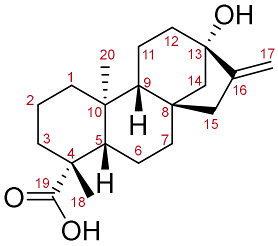

Steviol (**29**)
Chemical Formula: $C_{20}H_{30}O_3$
Exact Mass: 318.2195

Pos.	Reference in $CDCl_3$ [10] δ_C 75 MHz	Reference in $CDCl_3$ [10] δ_H (*J*/Hz) 300 MHz	Reference in $CDCl_3$ [47] (only δ_C reported) δ_C 50.1 MHz	Measured in $CDCl_3$, 298K δ_C 151 MHz	Measured in $CDCl_3$, 298K δ_H (*J*/Hz) 600 MHz
**1**	41.2	n.r.	40.5	40.6	ax 0.83, ddd (13.5, 13.5, 3.7)<br>eq 1.89, overlapped
**2**	20.3	n.r.	19.0	19.1	a 1.45, overlapped<br>b 1.93, overlapped
**3**	40.4	n.r.	37.8	37.9	ax 1.01, ddd (13.5, 13.5, 4.0)<br>eq 2.15, overlapped
**4**	41.7	-	43.6	43.7	-
**5**	56.8	n.r.	56.9	57.0	1.08, dd (12.0, 2.0)
**6**	18.9	n.r.	21.8	21.9	a 1.79, overlapped<br>b 1.88, overlapped
**7**	39.4	n.r.	41.2	41.4	a 1.44, overlapped<br>b 1.56, overlapped
**8**	43.5	-	41.8	41.9	-
**9**	53.8	n.r.	53.8	53.9	0.96, overlapped
**10**	39.2	-	39.5	39.6	-
**11**	21.7	n.r.	20.5	20.6	a 1.59, overlapped<br>b 1.79, overlapped
**12**	37.6	n.r.	39.5	39.5	a 1.55, overlapped<br>b 1.79, overlapped
**13**	80.4	-	80.4	80.5	-
**14**	47.3	n.r.	47.4	47.1	a 1.30, br d (10.3)<br>b 2.10, overlapped
**15**	46.8	n.r.	47.0	47.5	a 2.07, overlapped<br>b 2.20, overlapped
**16**	155.5	-	155.7	155.9	-
**17**	103.1	4.8, d (2.1) [tent.]<br>5.0, t (2.6) [tent.]	103.0	103.2	a 4.82, br s<br>b 4.98, br s
**18**	28.1	1.2, s [tent.]	28.8	28.9	1.24, s
**19**	183.8	-	183.5	183.3	-
**20**	15.4	1.0, s [tent.]	15.4	15.5	0.96, s

n.r. = not reported.

[tent.] = tentative assignments. The published reference data in $CDCl_3$ reported only few ^1H chemical shifts and those were not assigned. ^1H NMR ($CDCl_3$, 300 MHz) δ (ppm): 1.0 (3H, s), 1.2 (3H, s), 1.0 (3H, s), 2.1 (2H, dt, *J*=6.1, 2.5 Hz), 2.2 (1H, q, *J*=3.3 Hz), 4.8 (1H, d, *J*=2.1 Hz), 5.0 (1H, t, *J*=2.6 Hz). . Assignments of ^1H reference data in our table were made based on close similarity to our data, which were fully supported by 2D NMR measurements.

Yellow highlighted data are possibly swapped assignments in the referenced data. Our assignments are supported by HMBC correlations.

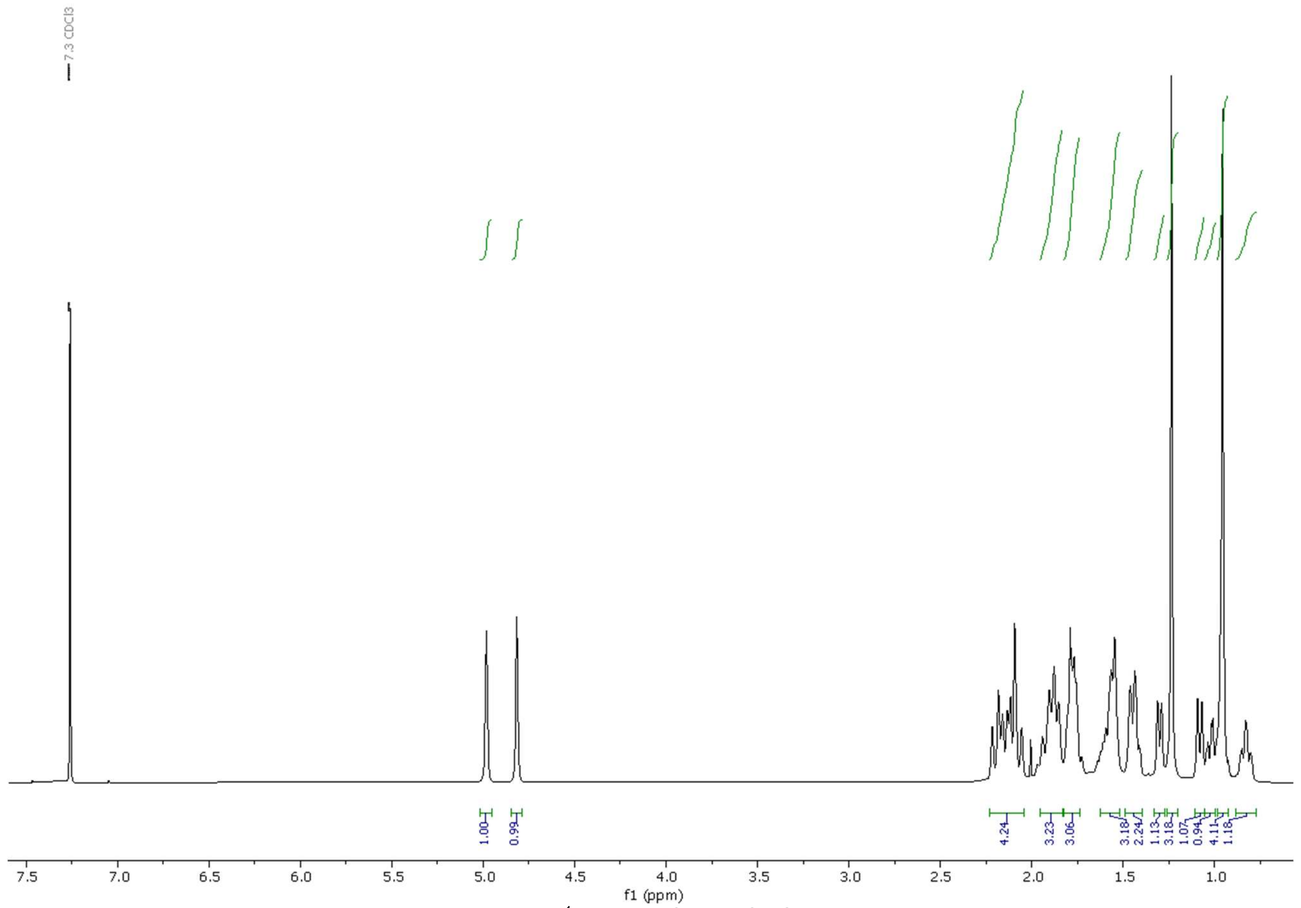

Figure N29.A ^{1}H NMR of **29** in $CDCl_3$ at 600 MHz.

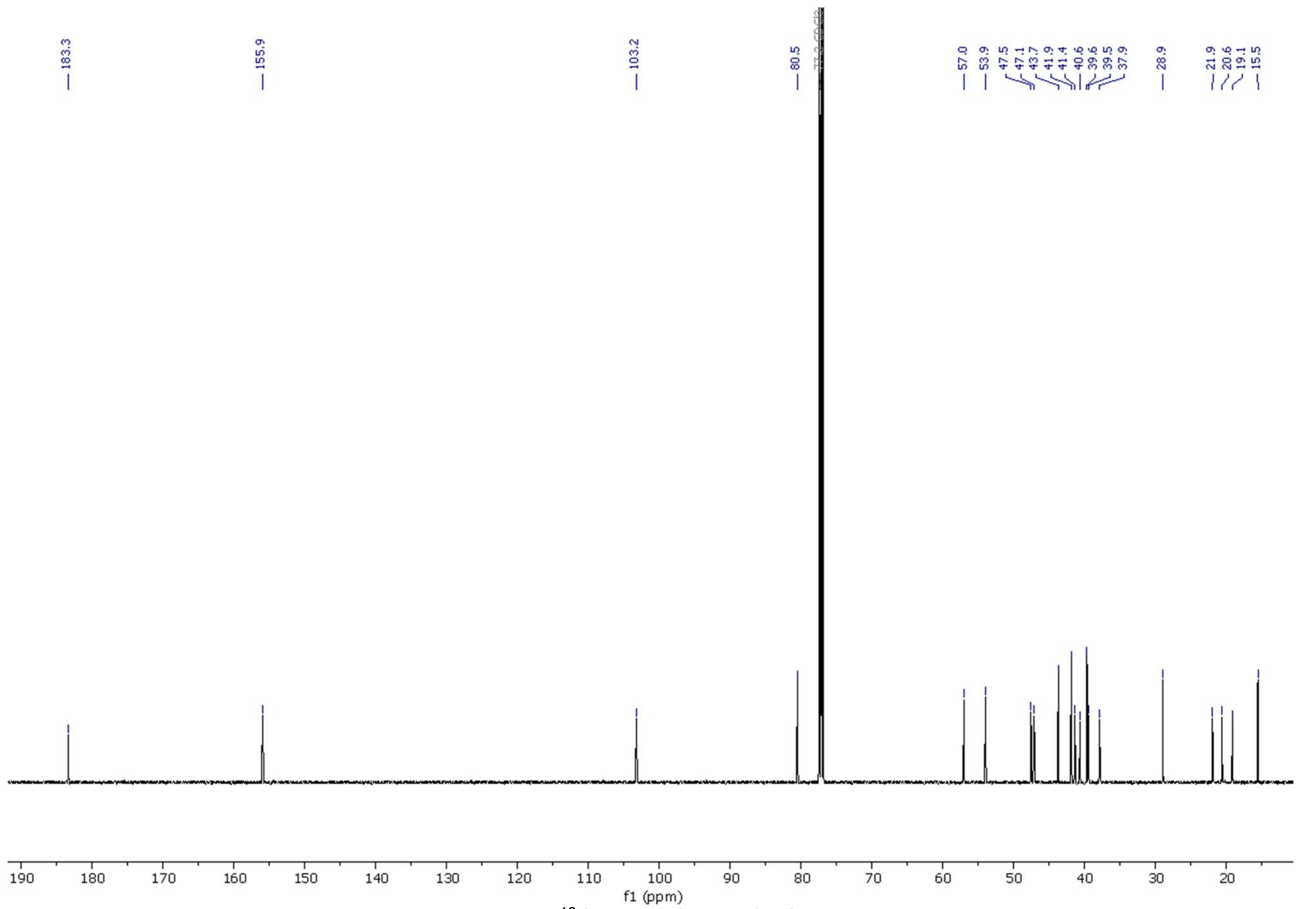

Figure N29.B ^{13}C NMR of **29** in $CDCl_3$ at 151 MHz.

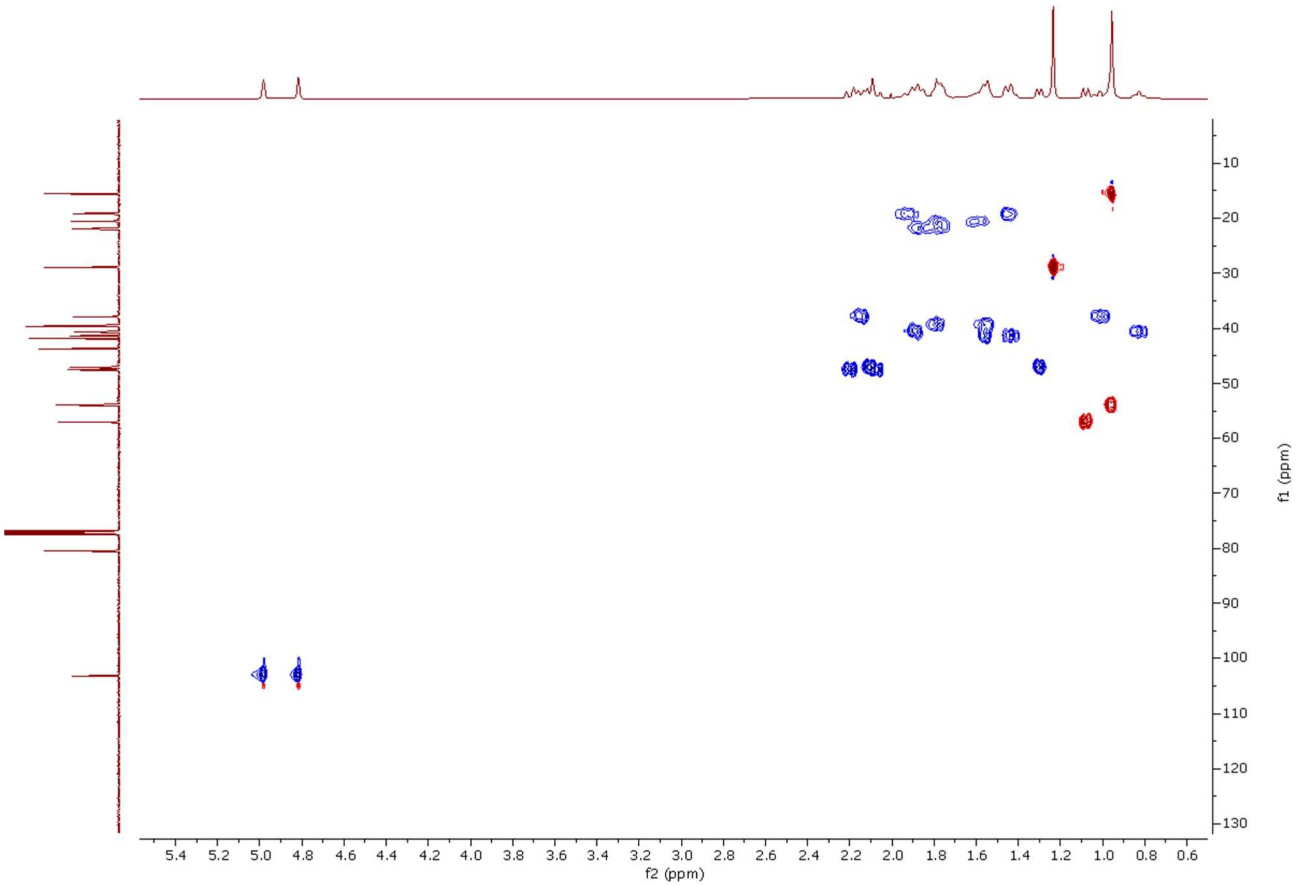

Figure N29.C HSQC NMR of **29** in $CDCl_3$.

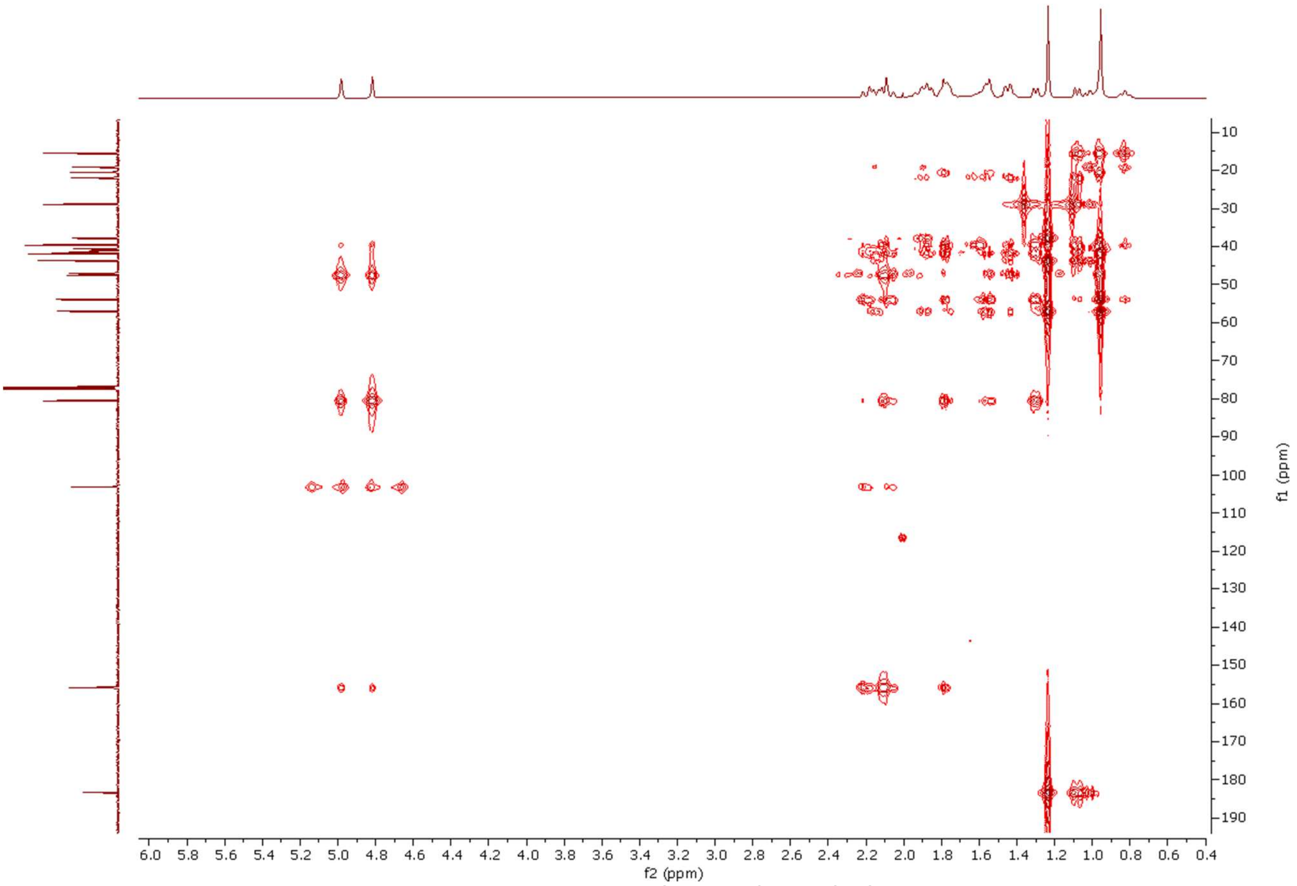

Figure N29.D HMBC NMR of **29** in $CDCl_3$.

Isosteviol (30)

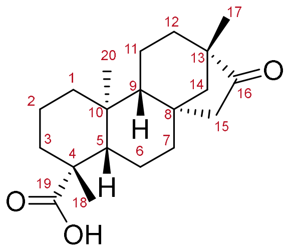

Isosteviol (**30**)
Chemical Formula: $C_{20}H_{30}O_3$
Exact Mass: 318.2195

Pos.	Reference in $CDCl_3$ [50]		Measured in $CDCl_3$, 298K	
	δ_C 101 MHz	δ_H *(J/Hz)* 400 MHz	δ_C 151 MHz	δ_H *(J/Hz)* 600 MHz
**1**	39.9 [tent.]	0.95–0.85, m [tent.]<br>n.a.	39.9	ax 0.91, ddd (13.5, 13.3, 3.7)<br>eq 1.73, overlapped
**2**	19.0 [tent.]	n.a.<br>n.a.	19.0	a 1.44, overlapped<br>b 1.84, overlapped
**3**	37.8 [tent.]	1.07–1.00, m [tent.]<br>2.15, d (13.3) [tent.]	37.8	ax 1.03, ddd (13.4, 13.4, 4.0)<br>eq 2.17, br d (13.4)
**4**	43.8 [tent.]	-	43.8	-
**5**	57.1 [tent.]	n.a.	57.1	1.15, br d (12.4)
**6**	21.7 [tent.]	n.a.<br>n.a.	21.8	a 1.75, overlapped<br>b 1.88, overlapped
**7**	41.6 [tent.]	1.49, dd (13.6, 2.9) [tent.]<br>n.a.	41.6	a 1.49, dd (13.6, 2.9)<br>b 1.66, overlapped
**8**	39.6 [tent.]	-	39.6	-
**9**	54.8 [tent.]	n.a.	54.9	1.20, overlapped
**10**	38.3 [tent.]	-	38.3	-
**11**	20.5 [tent.]	n.a.<br>n.a.	20.5	a 1.20, overlapped<br>b 1.68, overlapped
**12**	37.4 [tent.]	n.a.<br>n.a.	37.4	a 1.37, overlapped<br>b 1.61, overlapped
**13**	48.9 [tent.]	-	48.9	-
**14**	54.4 [tent.]	n.a.<br>n.a.	54.4	a 1.41, d (11.6)<br>b 1.54, d (11.6)
**15**	48.6 [tent.]	2.63, dd (18.6, 2.4) [tent.]	48.6	a 1.81, d (18.6)<br>b 2.64, br d (18.6)
**16**	n.r	-	223.0	-
**17**	20.0 [tent.]	0.97, s [tent.]	20.0	0.98, s
**18**	29.1 [tent.]	1.24, s [tent.]	29.1	1.25, s
**19**	183.8 [tent.]	-	183.3	-
**20**	13.4 [tent.]	0.77, s [tent.]	13.5	0.79, s

n.r. = not reported.

n.a. = not assigned.

[tent.] = tentative assignments.

The published reference data in $CDCl_3$ reported only unassigned ^{1}H and ^{13}C NMR chemical shifts, including broad multiplet regions (e.g., 1.90–1.53 ppm, m, 9H) that cannot be correlated to individual protons. Assignments of reference data in our table were made based on close similarity to our data, which were fully supported by 2D NMR measurements. Other reference signals remain unassigned.

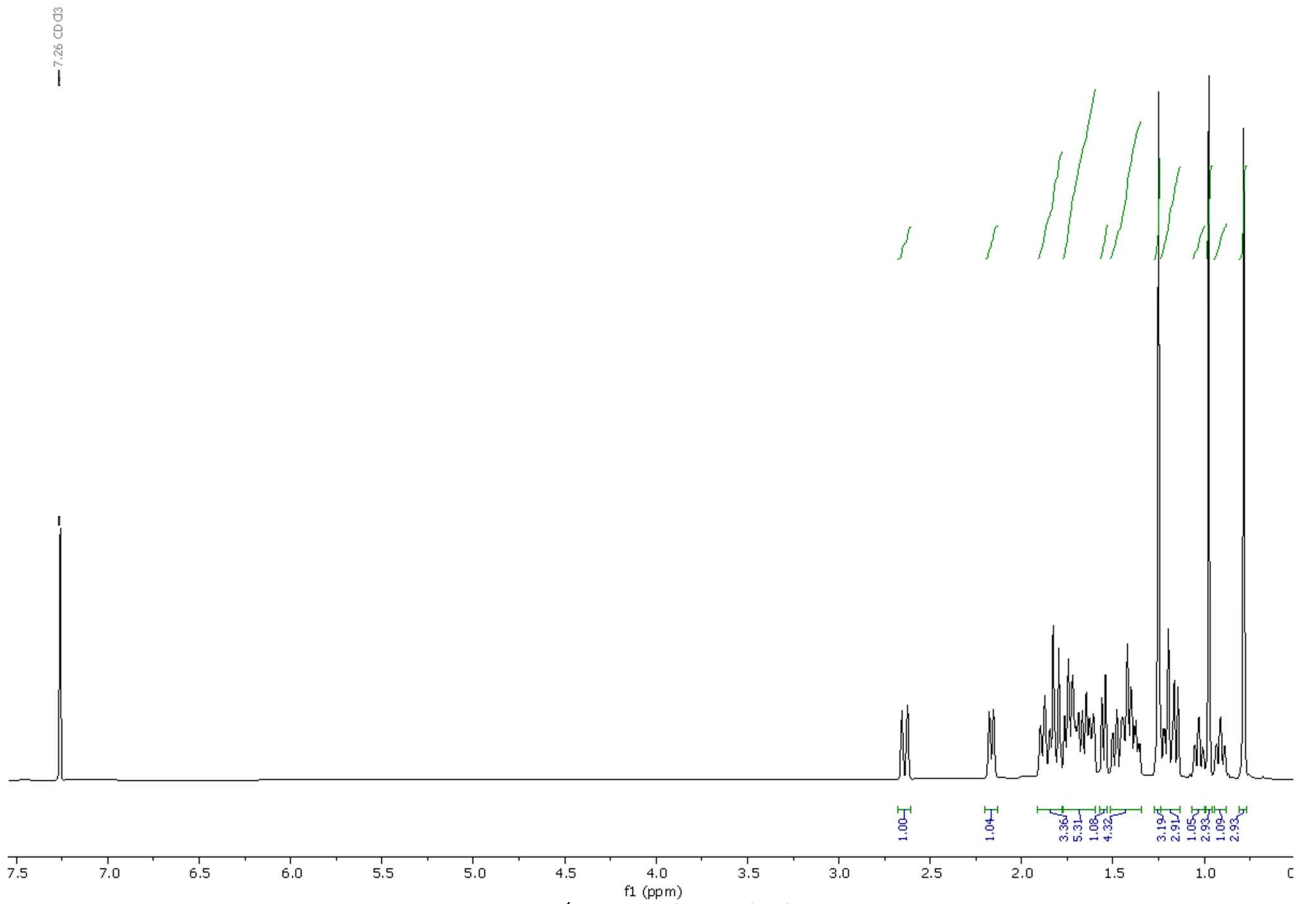

Figure N30.A ^{1}H NMR of **30** in $CDCl_3$ at 600 MHz.

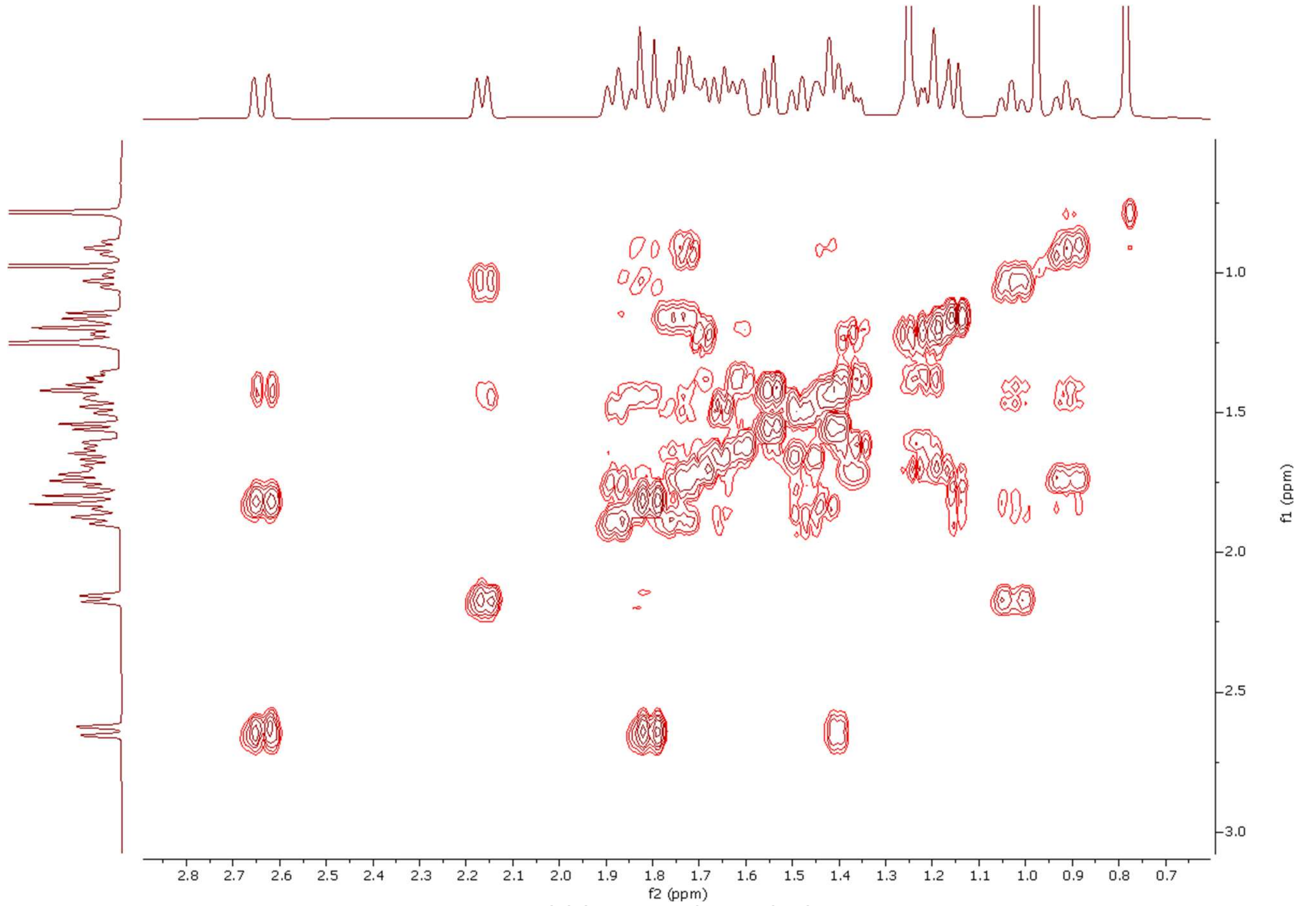

Figure N30.B COSY NMR of **30** in $CDCl_3$ at 600 MHz.

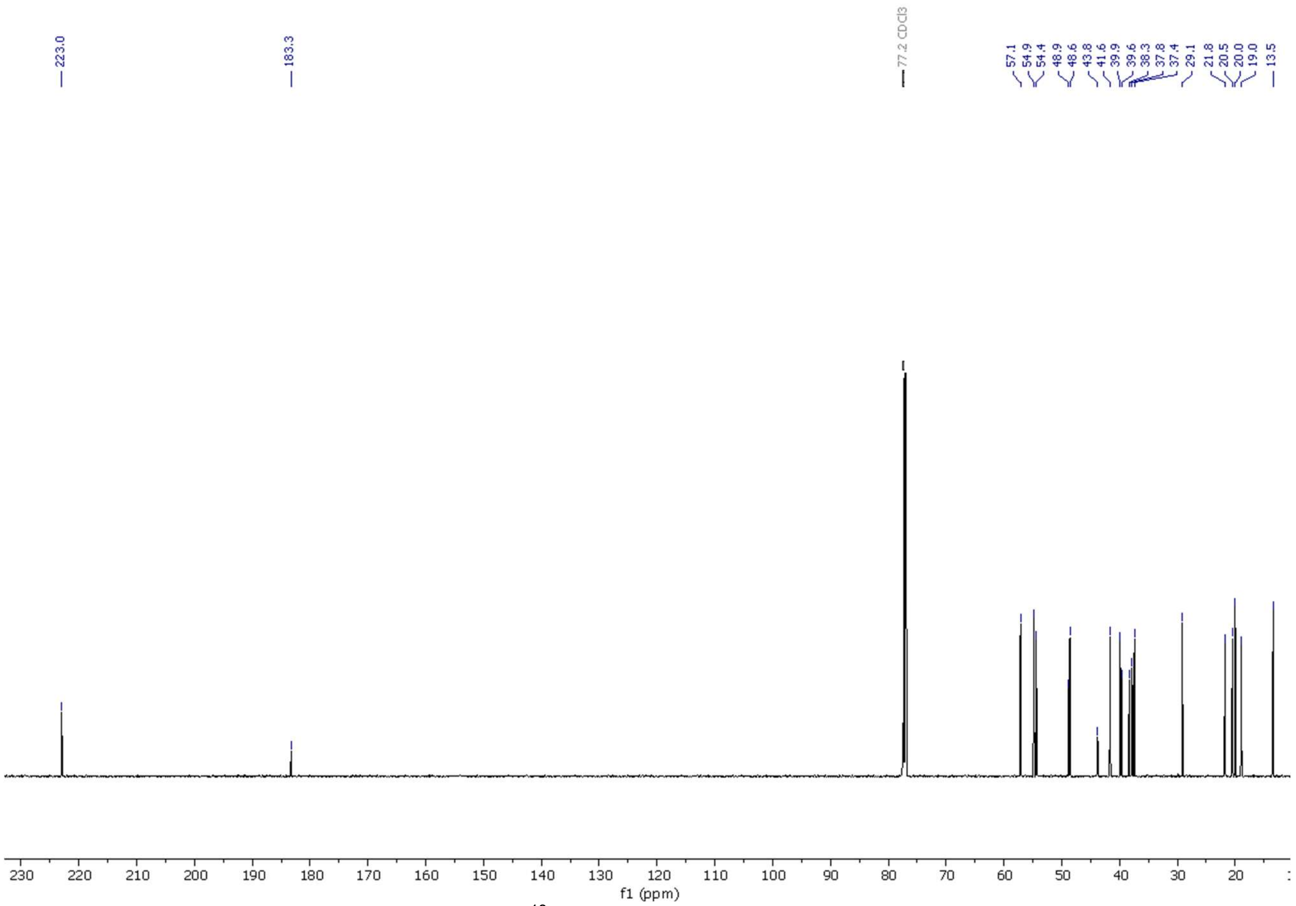

Figure N30.C ^{13}C NMR of **30** in $CDCl_3$ at 151 MHz.

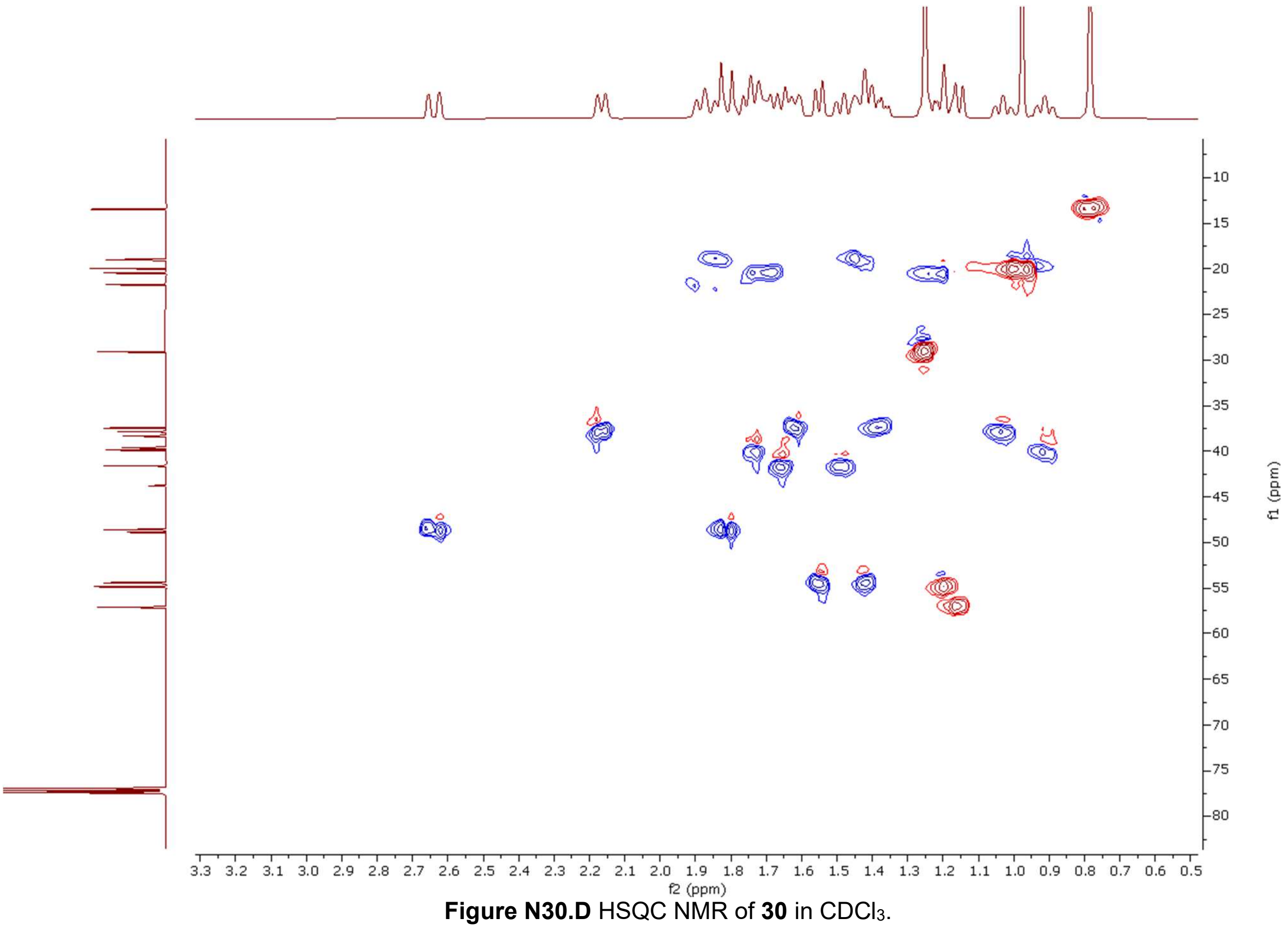

Figure N30.D HSQC NMR of **30** in $CDCl_3$.

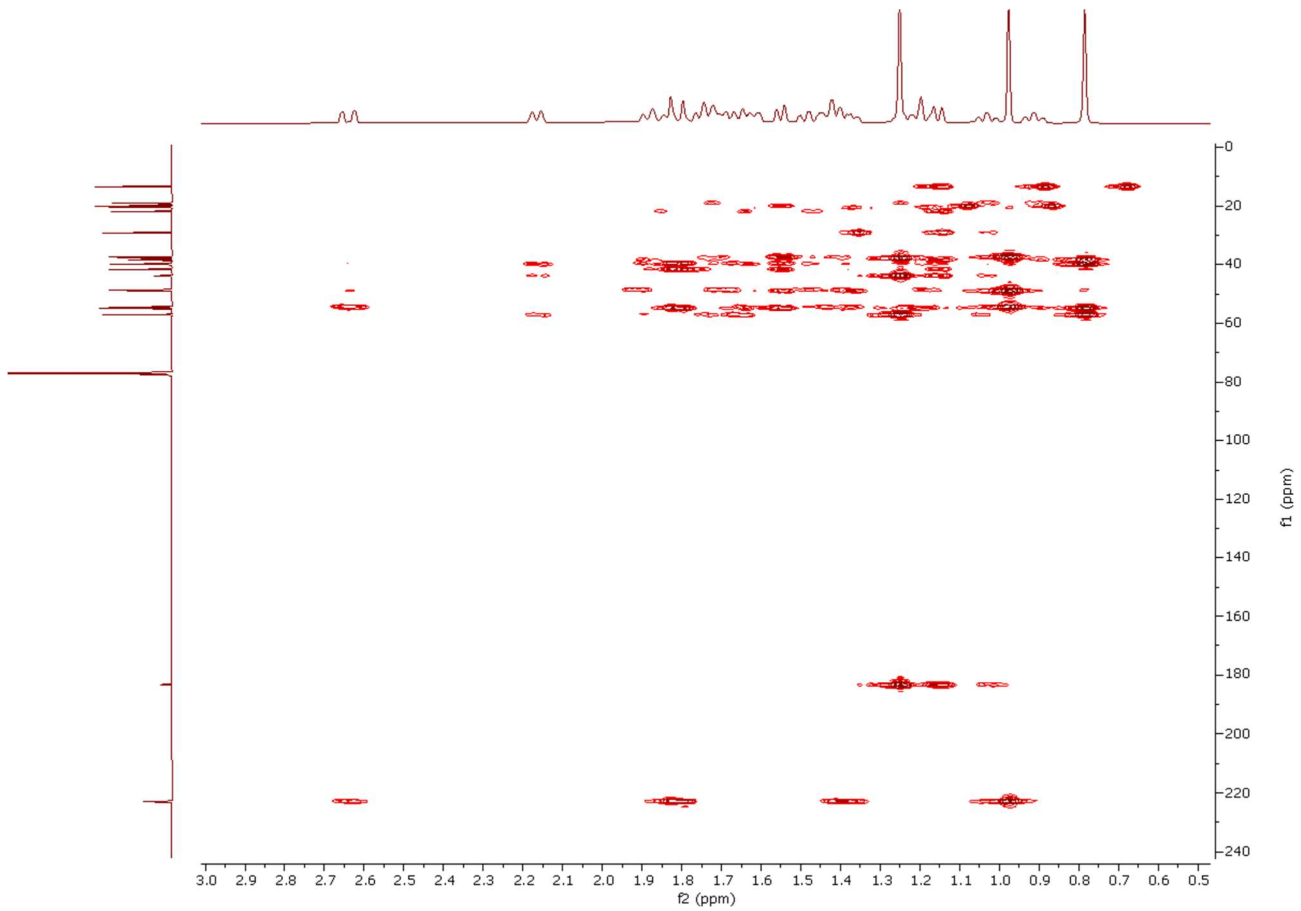

Figure N30.E HMBC NMR of **30** in $CDCl_3$.

7β,17-Dihydroxy-16-*epi*-*ent*-kauranoic acid (31)

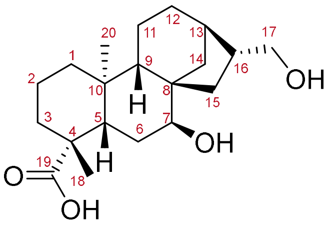

7β,17-Dihydroxy-16-*epi*-*ent*-kauranoic acid (**31**)

Chemical Formula: $C_{20}H_{32}O_4$
Exact Mass: 336.2301

	Reference in C_5D_5N [51] (only δ_H reported)	Measured in DMSO-d$_6$, 298K		
**Pos.**	δ_H (*J/Hz*)<br>90 MHz	δ_C<br>151 MHz	δ_H (*J/Hz*)<br>600 MHz	**Selected NOESY correlations to H [a]**
**1**	n.r.	40.4	ax 0.77, ddd (13.0, 13.0, 3.1)<br>eq 1.76, overlapped	H-1eq<br>H_3-20, H-1ax
**2**	n.r.	19.1	a 1.32, overlapped<br>b 1.81, overlapped	
**3**	n.r.	38.0	ax 0.91, overlapped<br>eq 2.03, m	 H_3-18
**4**	-	42.4	-	-
**5**	n.r.	46.2	1.65, d (12.5)	H_3-18
**6**	n.r.	30.0	a 1.78, overlapped<br>b 1.91, dd (12.5, 12.5)	H_3-18.<br>H_3-20
**7**	3.87, br s	75.5	3.28, br s	
**8**	-	48.2	-	-
**9**	n.r.	48.9	1.31, overlapped	
**10**	-	38.7	-	-
**11**	n.r.	18.1	a 1.42, m<br>b 1.52, overlapped	
**12**	n.r.	31.7	a 1.31, overlapped<br>b 1.49, overlapped	
**13**	n.r.	37.5	2.03, overlapped	H_2-17
**14**	n.r.	35.8	a 0.95, dd (11.3, 4.0)<br>b 1.55, br d (11.3)	
**15**	n.r.	41.5	a 1.00, dd (13.7, 5.2)<br>b 1.48, overlapped	H_2-17
**16**	n.r.	43.1	1.75, overlapped	H-12b, H-15b, H_2-17
**17**	3.70, d (7)	65.7	3.13, m	H-16, H-15a, H-13
**18**	1.41, s	28.7	1.04, s	H-3eq, H-6a, H-5
**19**	-	179.0 [HMBC]	-	-
**20**	1.23, s	15.5	0.87, s	H-1eq, H-6b

[a] Key NOESY correlations are shown in blue text.

n.r. = not reported.

[HMBC] = detected based on HMBC.

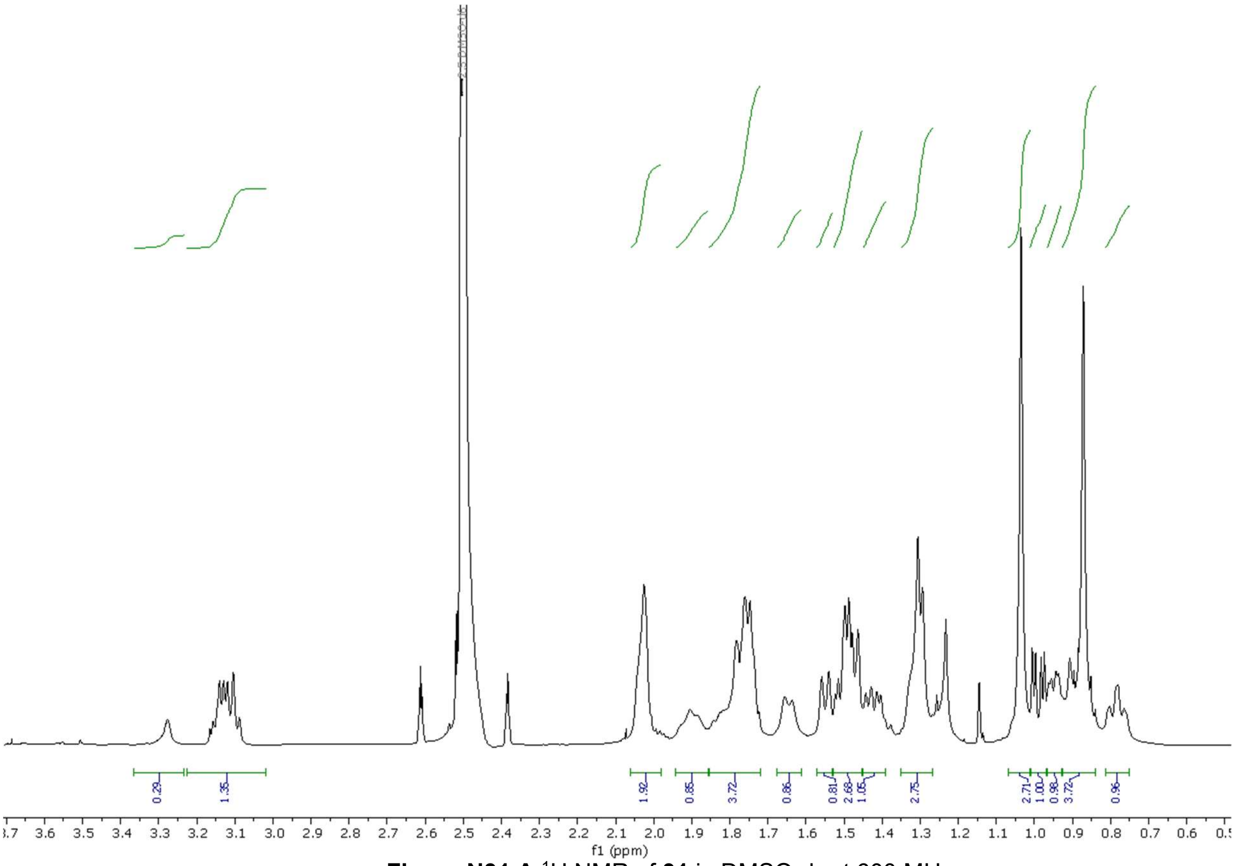

Figure N31.A ^{1}H NMR of **31** in DMSO-d_6 at 600 MHz.

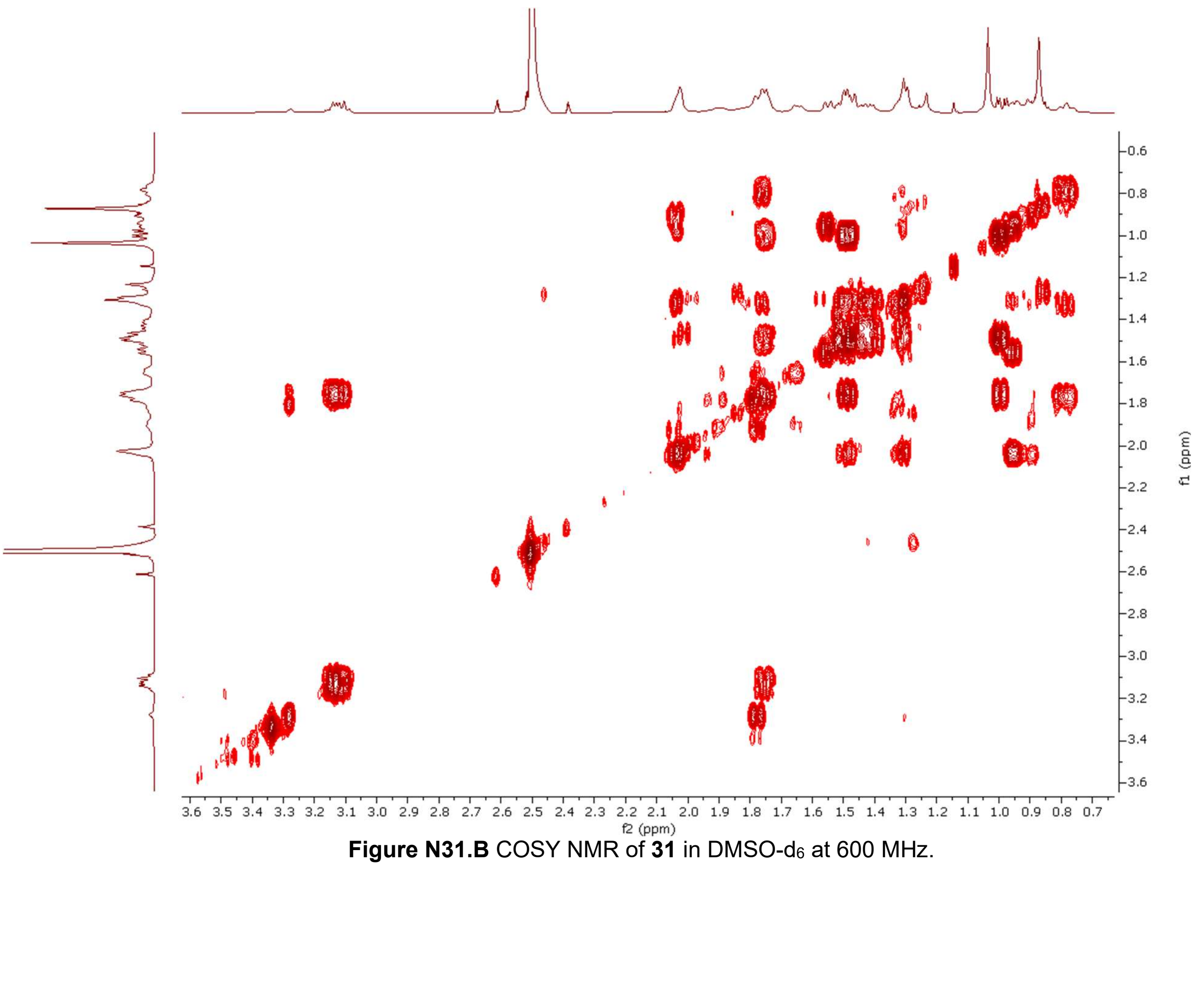

Figure N31.B COSY NMR of **31** in DMSO-d_6 at 600 MHz.

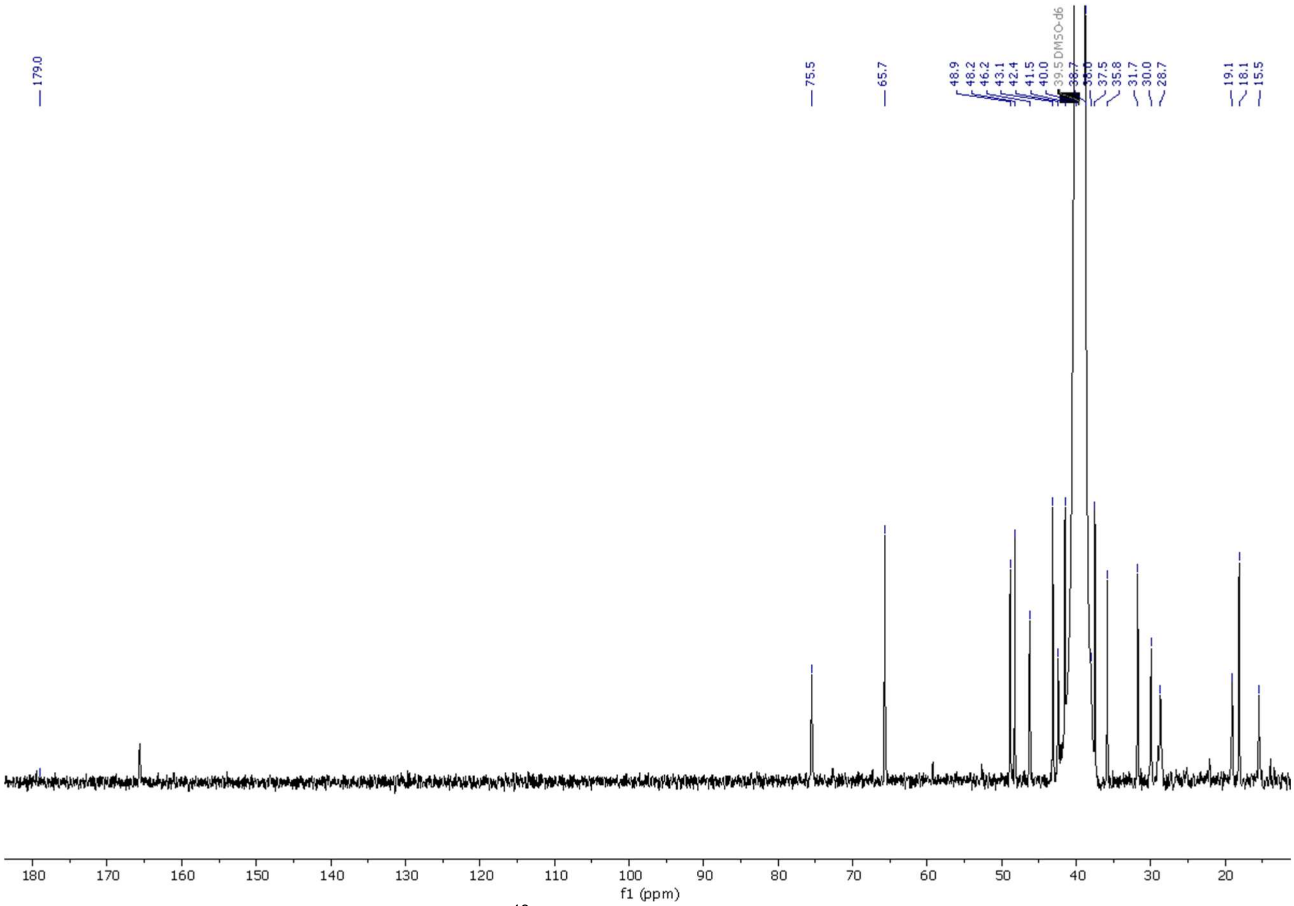

Figure N31.C ^{13}C NMR of **31** in DMSO-d_6 at 151 MHz.

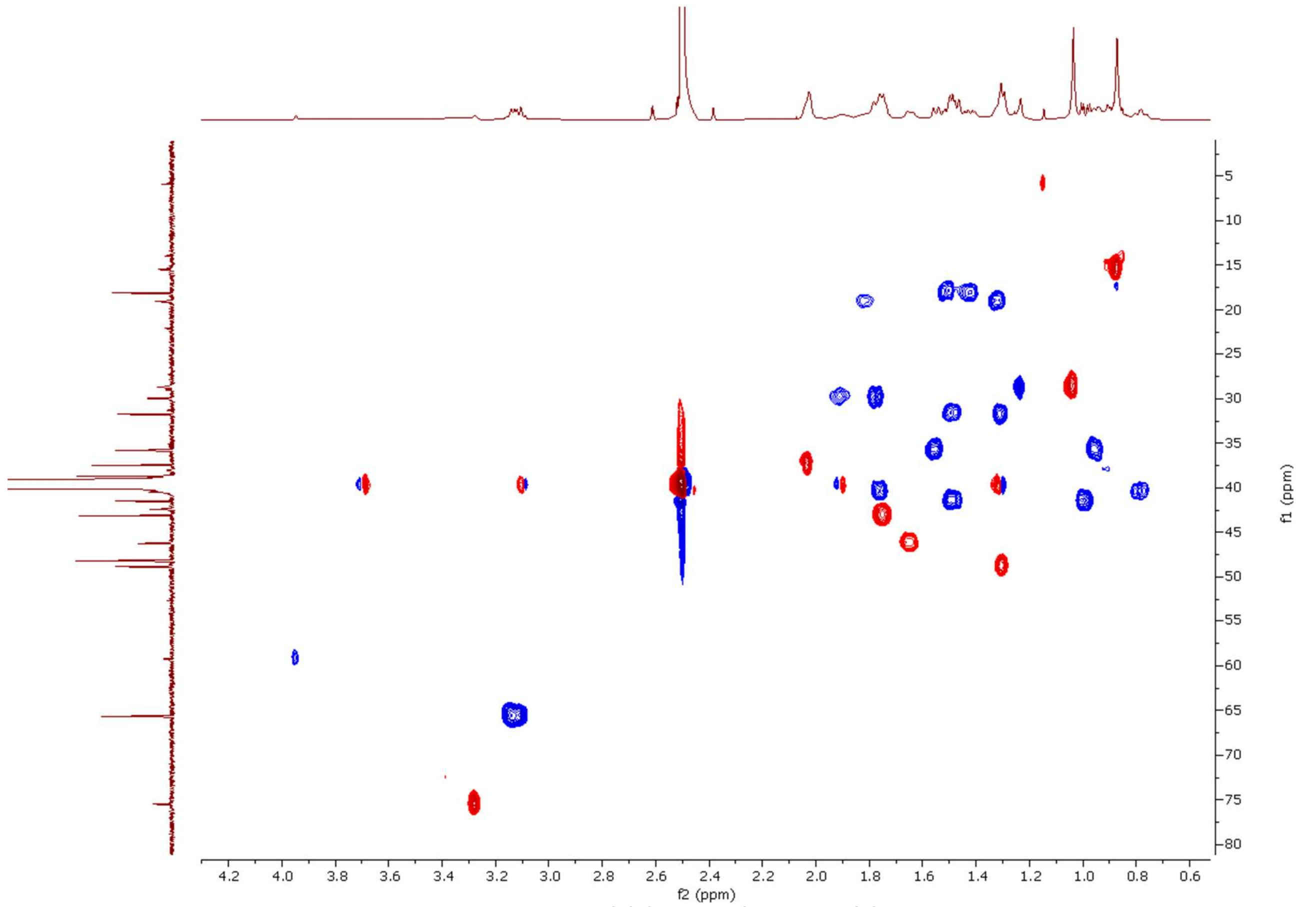

Figure N31.D HSQC NMR of **31** in DMSO-d_6.

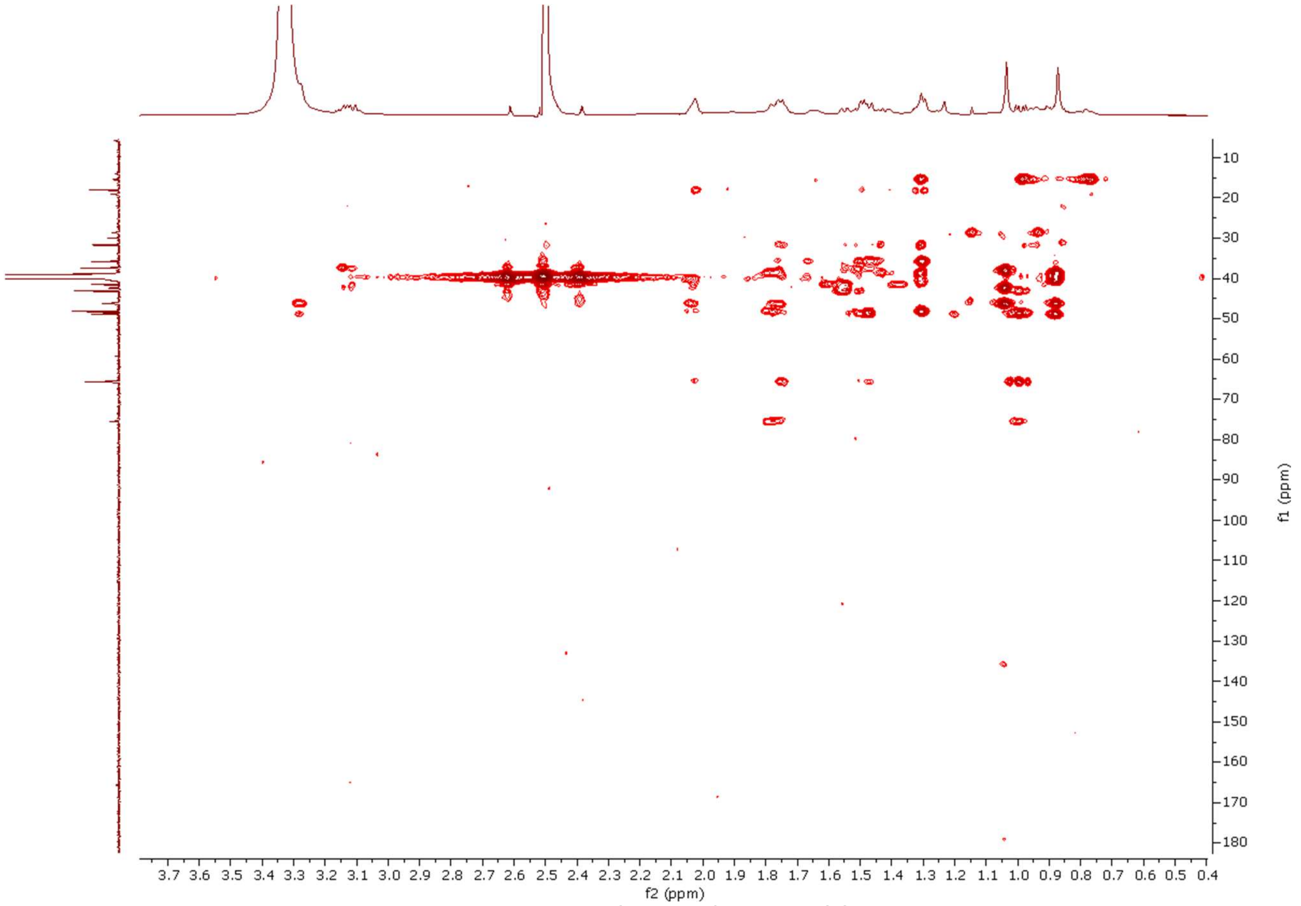

Figure N31.E HMBC NMR of **31** in DMSO-d_6.

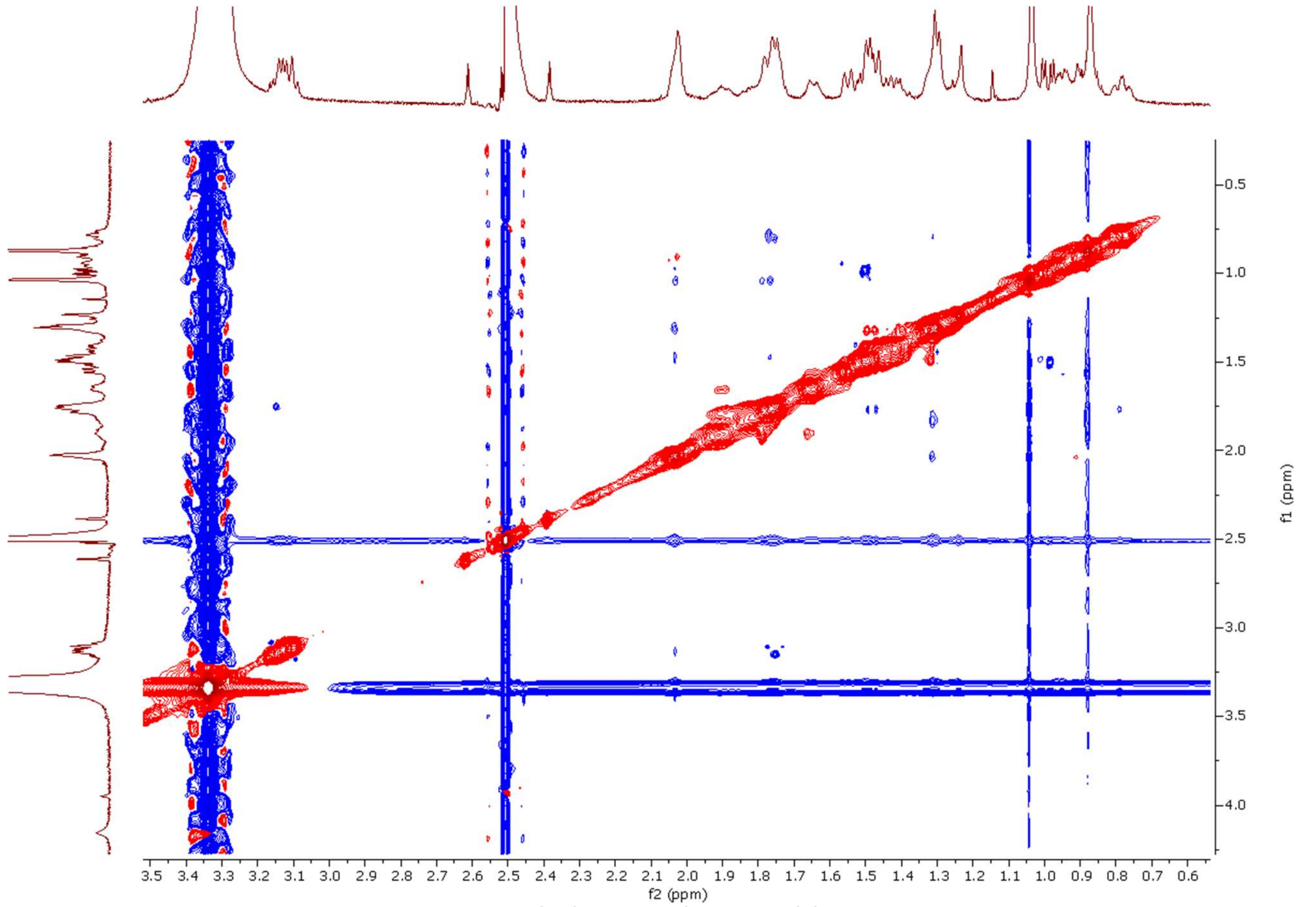

Figure N31.F NOESY NMR of **31** in DMSO-d_6 at 600 MHz.

7β,17-Dihydroxy-16-*epi*-*ent*-kauranoic acid methyl ester (31a)

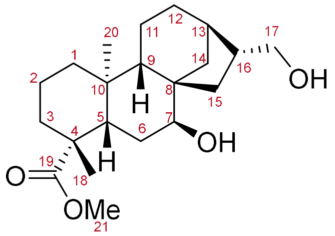

7β,17-Dihydroxy-16-*epi*-*ent*-kauranoic acid methyl ester (**31a**)
Chemical Formula: $C_{21}H_{34}O_4$
Exact Mass: 350.2457

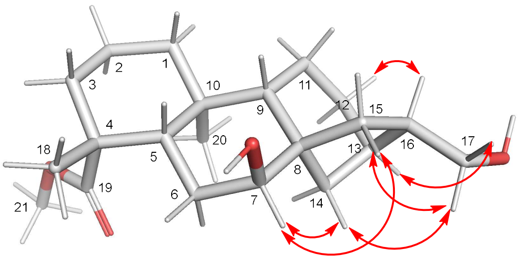

Key nOe

	Measured in $CDCl_3$, 298K		
**Pos.**	*δ*$_C$ 151 MHz	*δ*$_H$ *(J/Hz)* 600 MHz	**Selected NOESY correlations to H** [a]
**1**	40.7	ax 0.90, ddd (13.9, 13.9, 3.5)<br>eq 1.85, m	H-1eq<br>H-1ax
**2**	19.3	1.45, overlapped	
**3**	38.1	ax 1.07, overlapped<br>eq 2.19, ddd (13.2, 3.8, 3.8)	H-3eq<br>H-3ax
**4**	43.5	-	-
**5**	47.4	1.71, overlapped	H_3-18
**6**	29.6	a 2.00, overlapped<br>b 2.03, overlapped	H-7, H_3-18<br>H_3-20
**7**	77.8	3.56, br s	H-14a, H-15a, H-6a
**8**	48.8	-	-
**9**	49.4	1.41, br d (7.7)	
**10**	39.2	-	-
**11**	18.5	a 1.52, m<br>b 1.62, m	
**12**	32.0	a 1.45, overlapped<br>b 1.59, overlapped	H-16<br>H_3-20
**13**	38.3	2.11, br s	H_2-17
**14**	36.6	a 1.06, overlapped<br>b 1.72, overlapped	H-7<br>H_3-20
**15**	41.5	a 1.10, dd (13.5, 5.2)<br>b 1.72, overlapped	H-7, H_2-17
**16**	43.7	1.99, overlapped	H-12a, H_2-17
**17**	67.6	3.43, m	H-15a, H-14a, H-16, H-13
**18**	28.7	1.16, s	H-5, H-6a
**19**	178.4	-	-
**20**	15.4	0.83, s	H-14b, H-12b
**21**	51.4	3.65, s	

[a] Key NOESY correlations are shown in blue text.

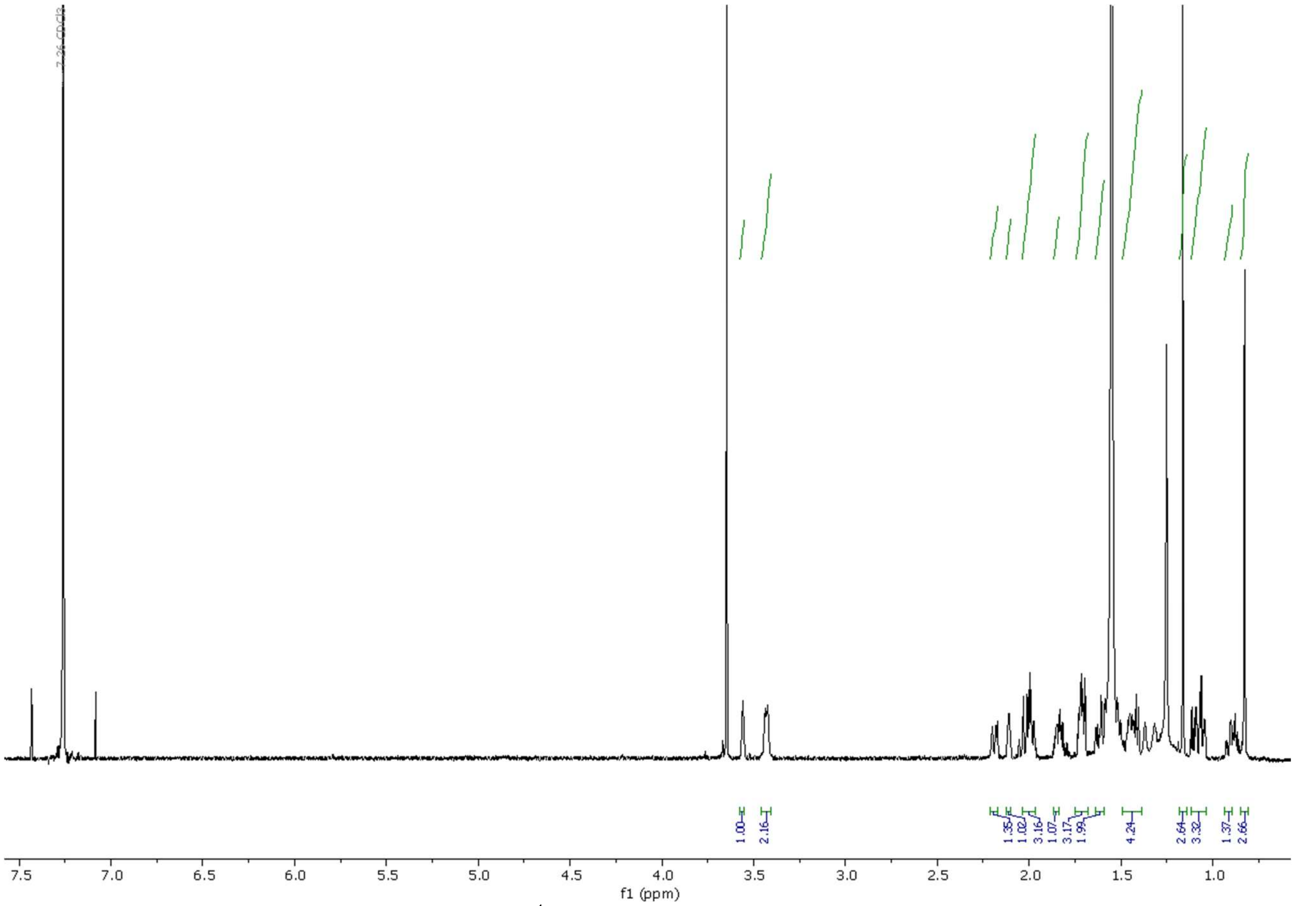

Figure N31a.A ^{1}H NMR of **31a** in $CDCl_3$ at 600 MHz.

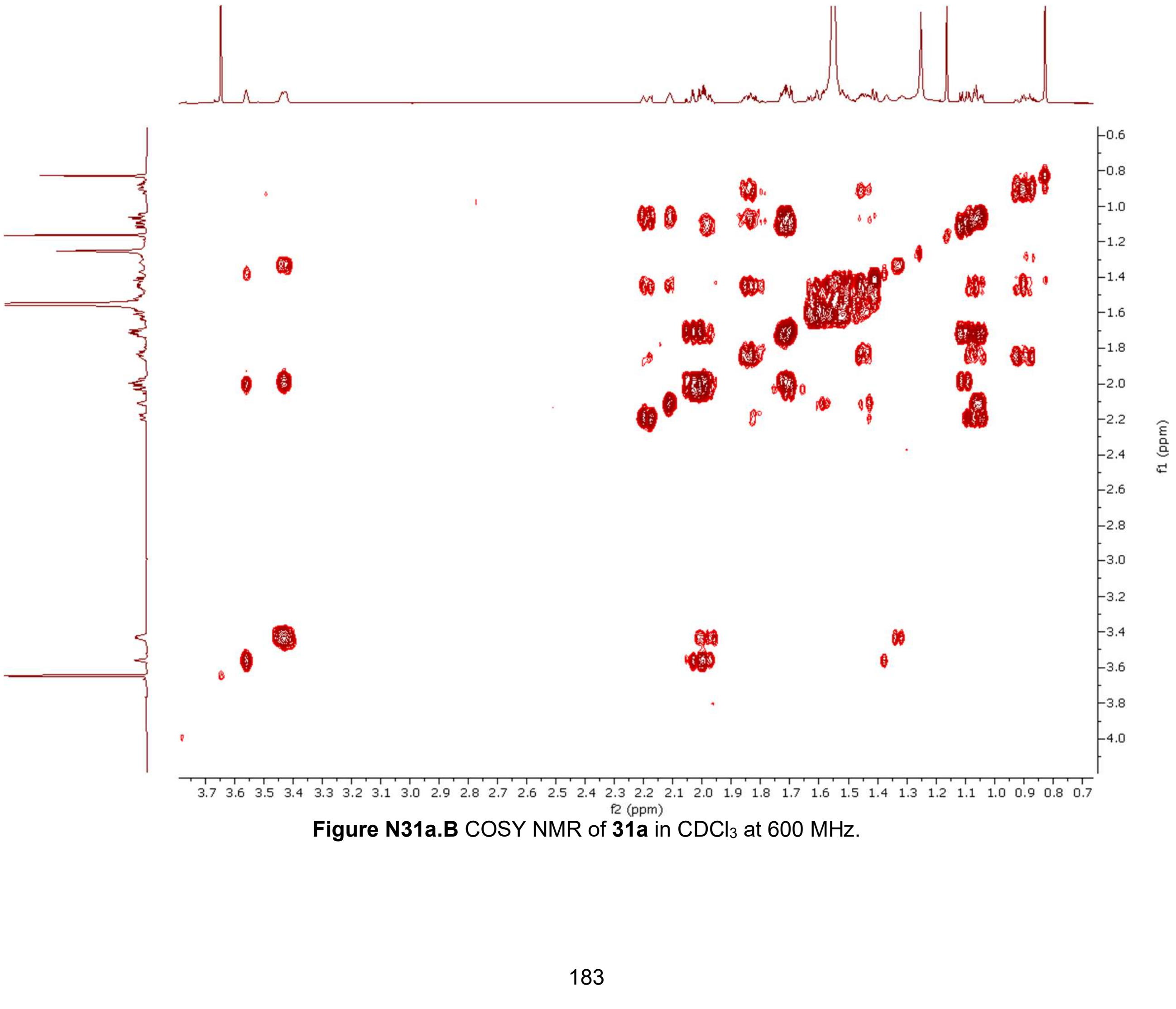

Figure N31a.B COSY NMR of **31a** in $CDCl_3$ at 600 MHz.

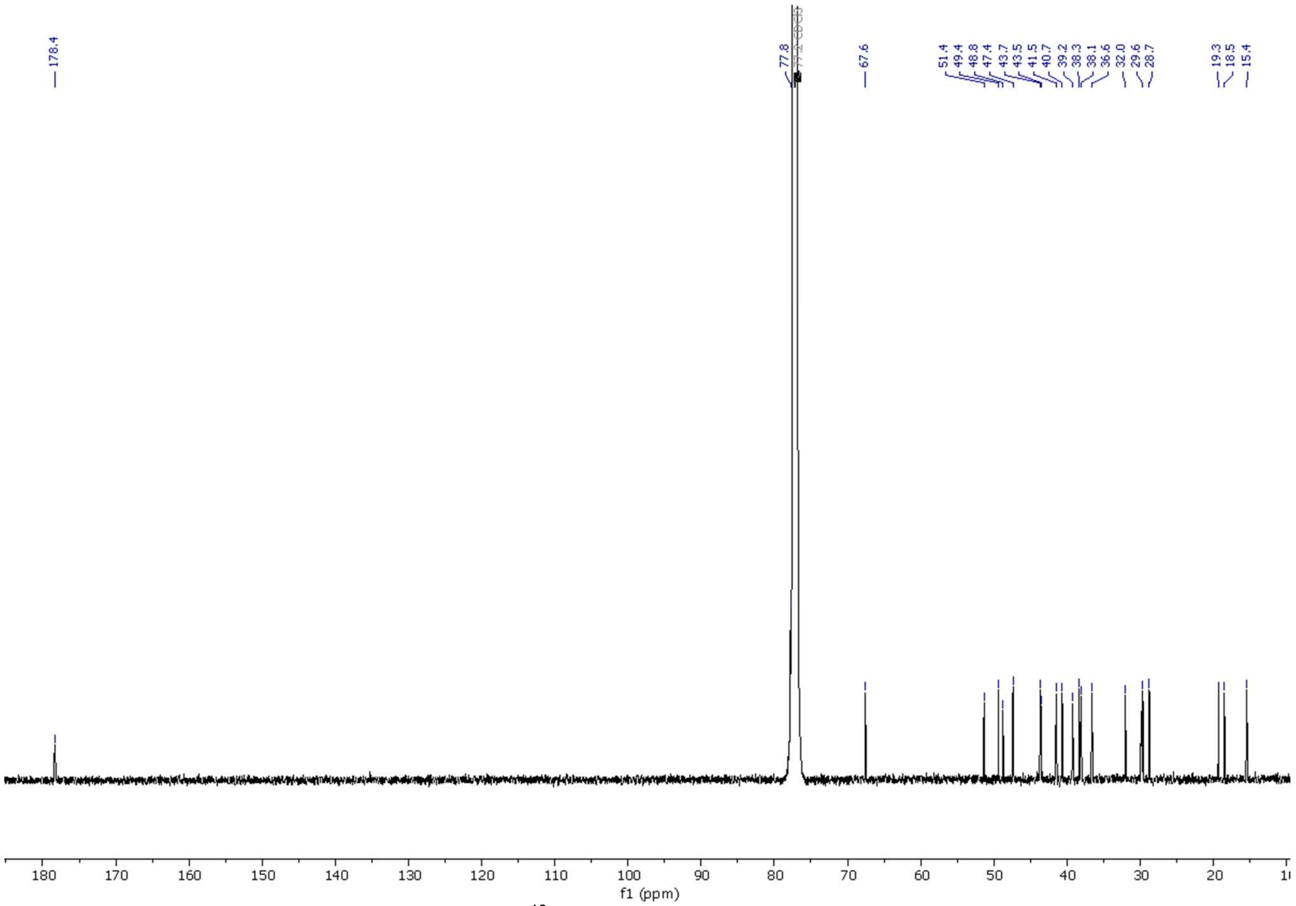

Figure N31a.C ^{13}C NMR of **31a** in $CDCl_3$ at 151 MHz.

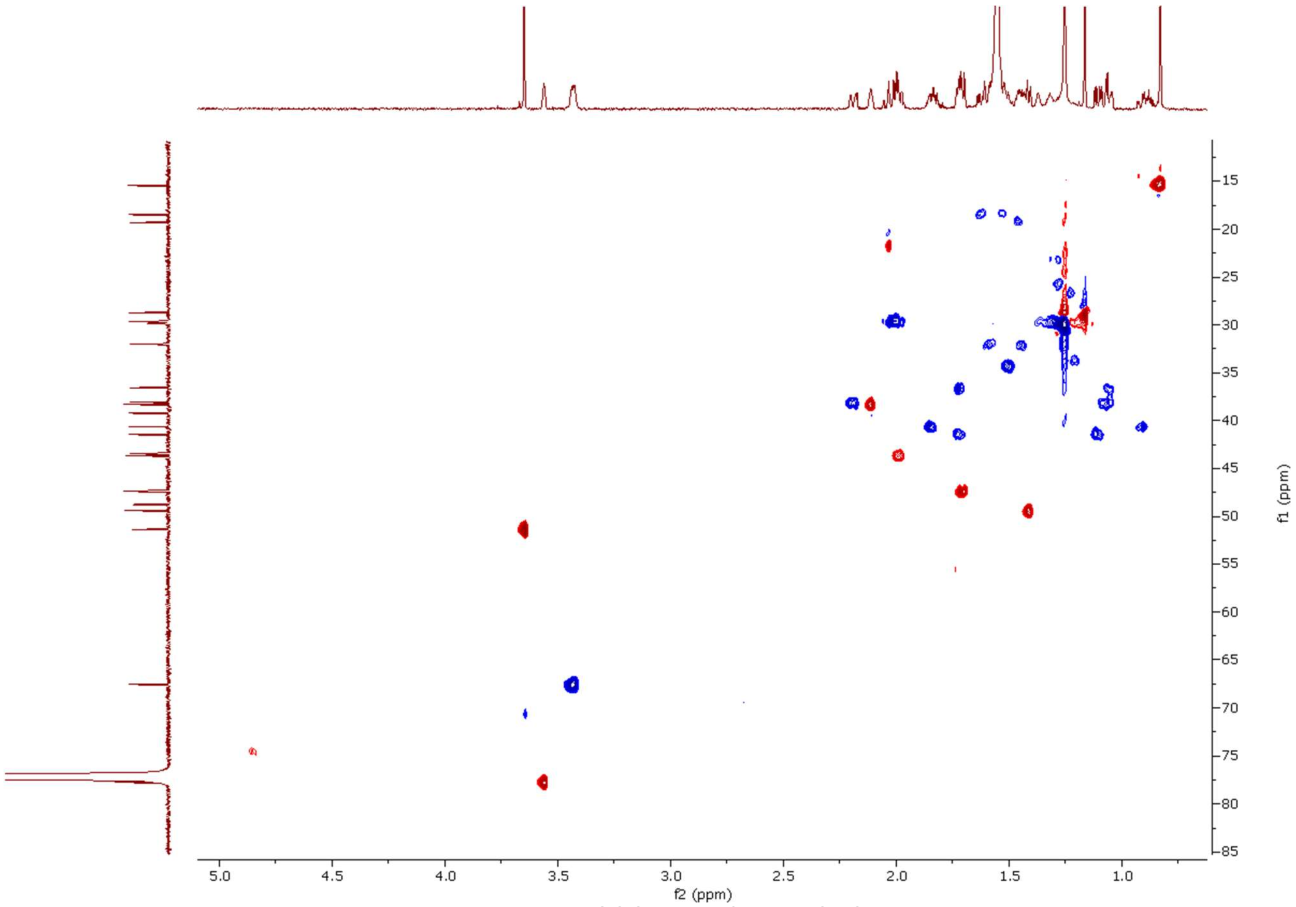

Figure N31a.D HSQC NMR of **31a** in $CDCl_3$.

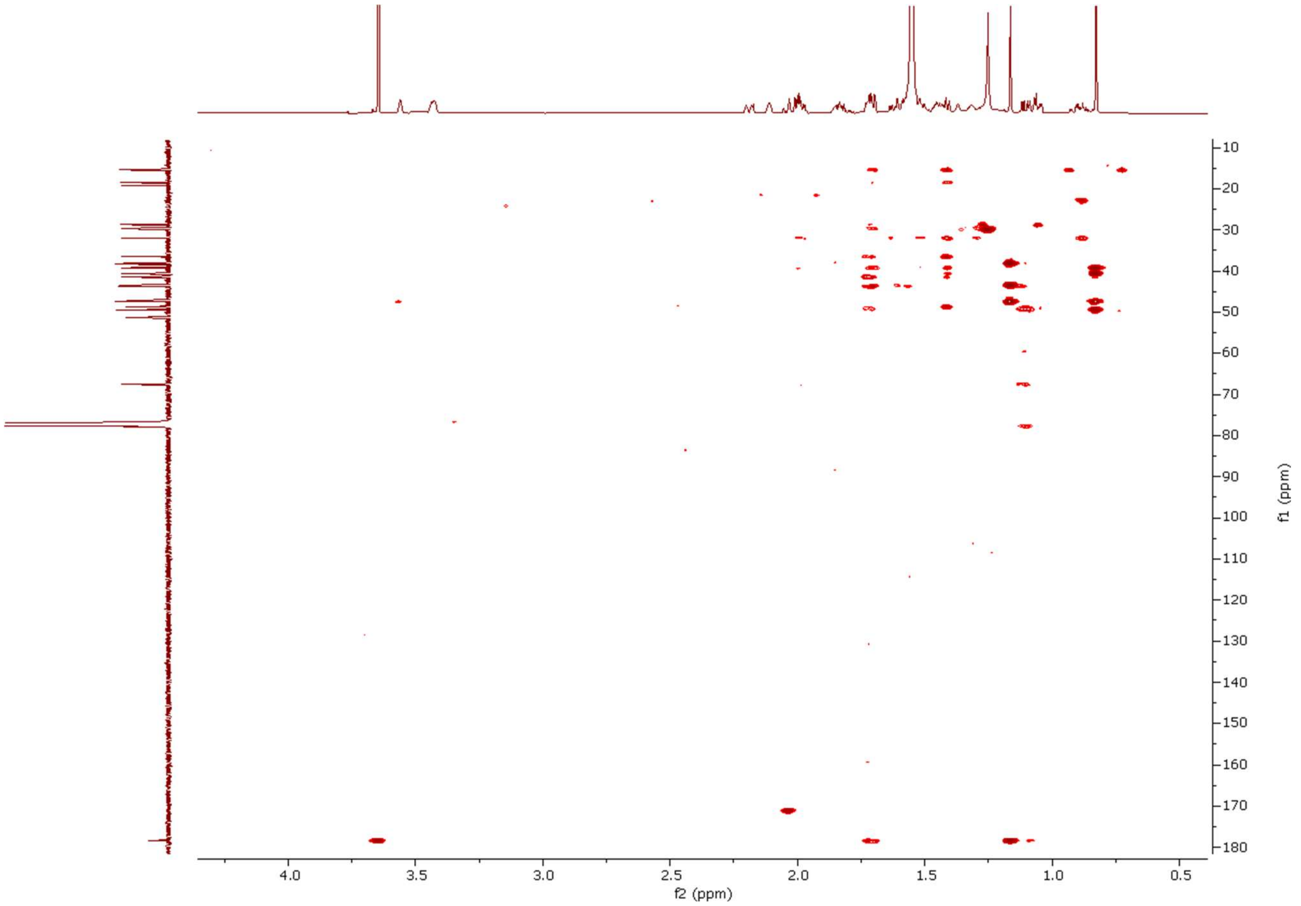

Figure N31a.E HMBC NMR of **31a** in $CDCl_3$.

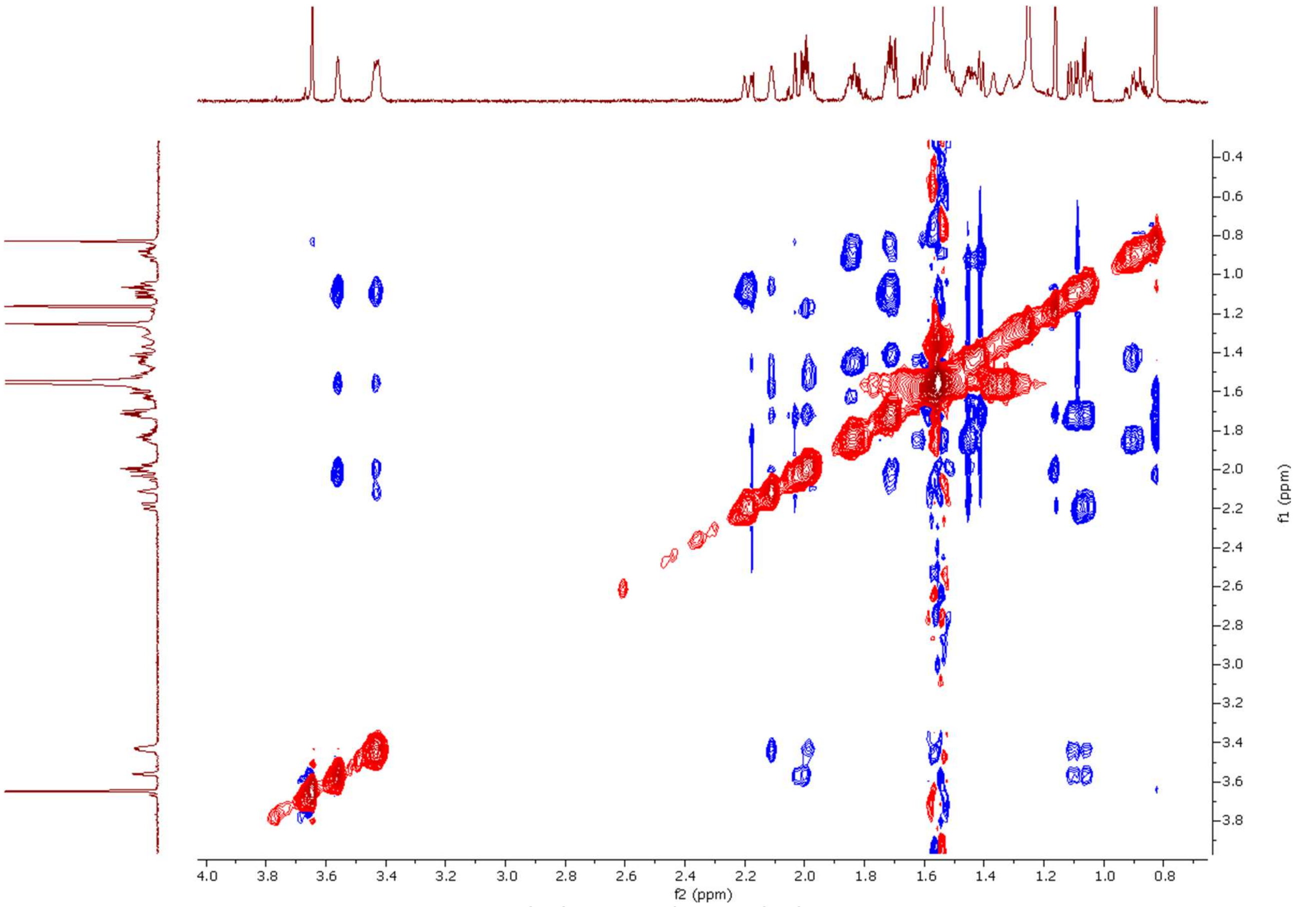

Figure N31a.F NOESY NMR of **31a** in $CDCl_3$ at 600 MHz.

7β-Hydroxy-16-*epi*-*ent*-kauran-17,19-dioic acid (32)

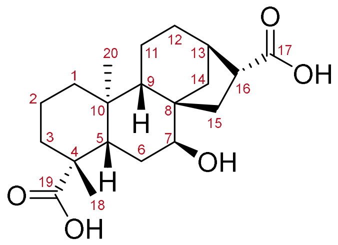

7β-Hydroxy-16-*epi*-*ent*-kauran-17,19-dioic acid (**32**)

Chemical Formula: $C_{20}H_{30}O_5$

Exact Mass: 350.2093

Measured in DMSO-D$_6$, 298K

Pos.	δ_C 151 MHz	δ_H (*J/Hz*) 600 MHz	Selected NOESY correlations to H [a]
**1**	40.3	ax 0.79, ddd (13.9, 13.9, 3.0)<br>eq 1.76, overlapped	H-2a<br>H_3-20
**2**	19.0	a 1.33, overlapped<br>b 1.79, overlapped	H-2b, H-1ax<br>H-2a
**3**	37.9	ax 0.92, ddd (13.3, 12.3, 3.2)<br>eq 2.02, ddd (12.3, 3.2, 3.2)	H-3eq<br>H_3-18, H-3ax
**4**	42.4	-	-
**5**	46.2	1.67, br d (12.7)	H_3-18
**6**	30.0	a 1.79, overlapped<br>b 1.89, br t (12.7)	H_3-18
**7**	75.1	3.35, overlapped	
**8**	48.6	-	-
**9**	48.7	1.32, overlapped	
**10**	38.7	-	-
**11**	17.9	a 1.40, overlapped<br>b 1.52, m	
**12**	31.4	a 1.40, overlapped<br>b 1.48, m	H-16<br>H-13
**13**	40.2	2.36, m	H-12b, H-14b
**14**	36.9	a 1.10, dd (11.3, 4.3)<br>b 1.59, overlapped	H-14b<br>H-14a
**15**	41.3	a 1.59, overlapped<br>b 1.82, overlapped	
**16**	45.4	2.43, br t (7.0)	H-12a
**17**	n.d	-	-
**18**	28.6	1.04, s	H-3eq, H-6a, H-5
**19**	179.3	-	-
**20**	15.3	0.84, s	H-1eq

[a] Key NOESY correlations are shown in blue text.

n.d. = not detected - see NMR data of dimethyl ester (**32a**) for complete data.

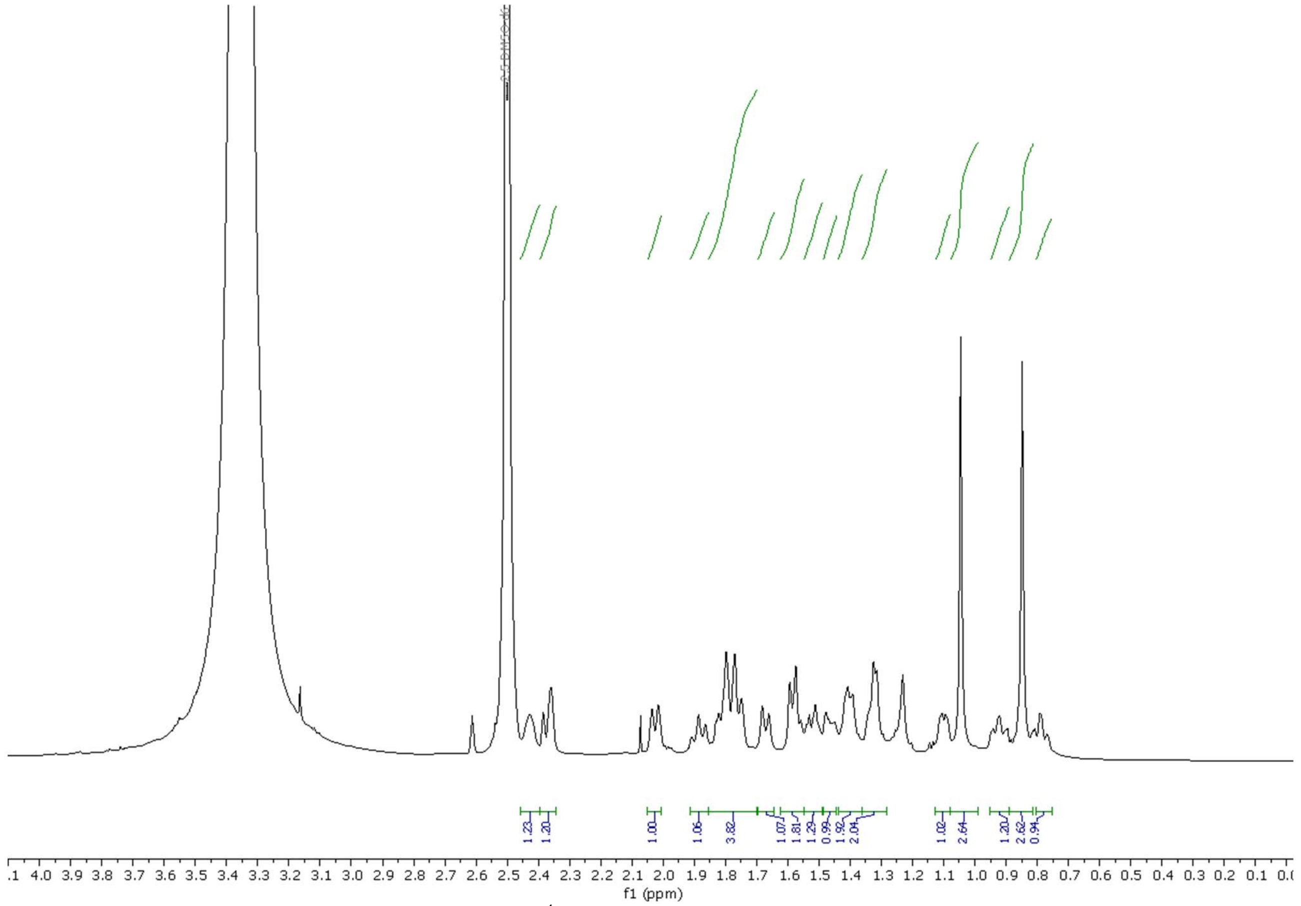

Figure N32.A ^1H NMR of **32** in DMSO-d_6 at 600 MHz.

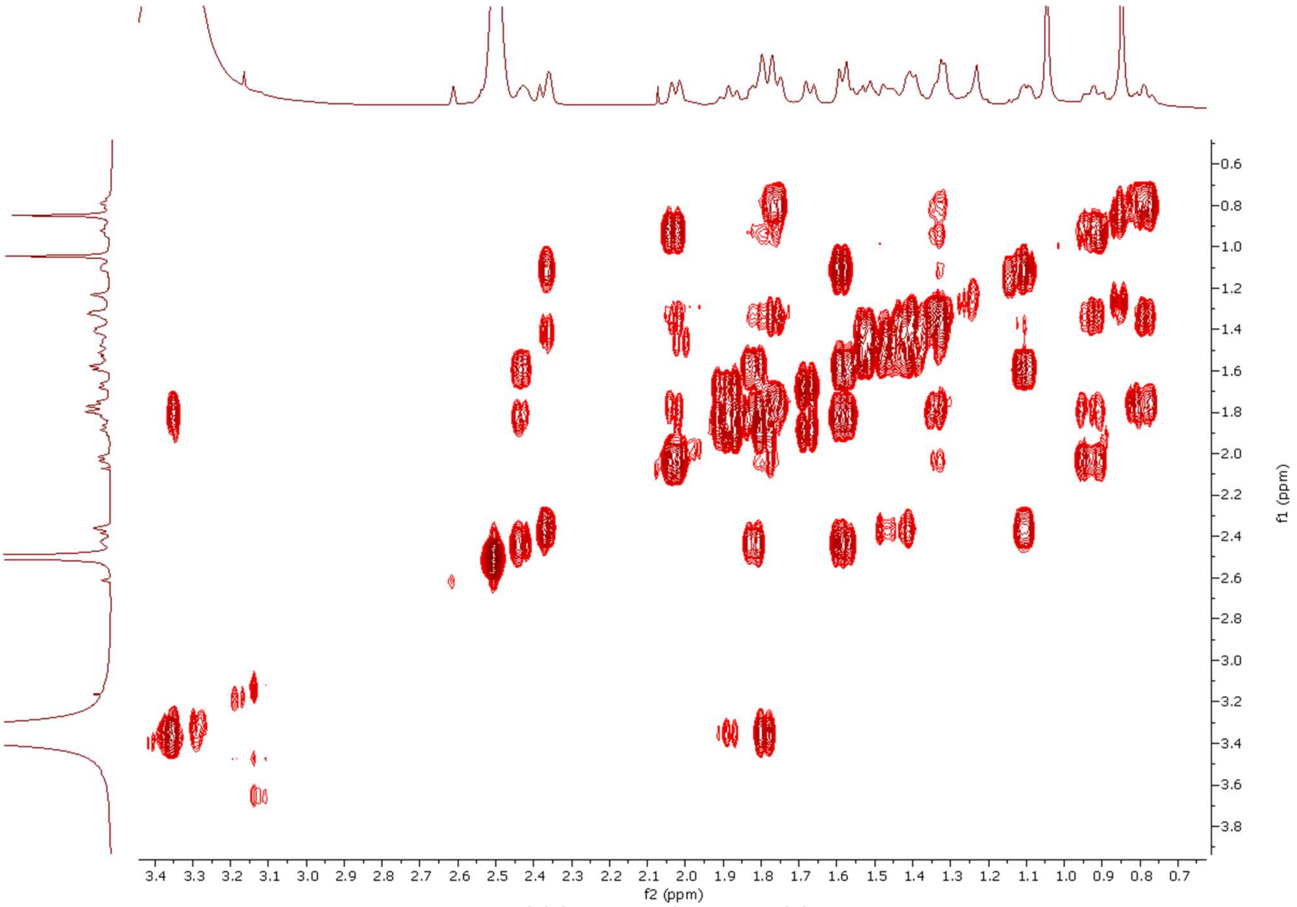

Figure N32.B COSY NMR of **32** in DMSO-d_6 at 600 MHz.

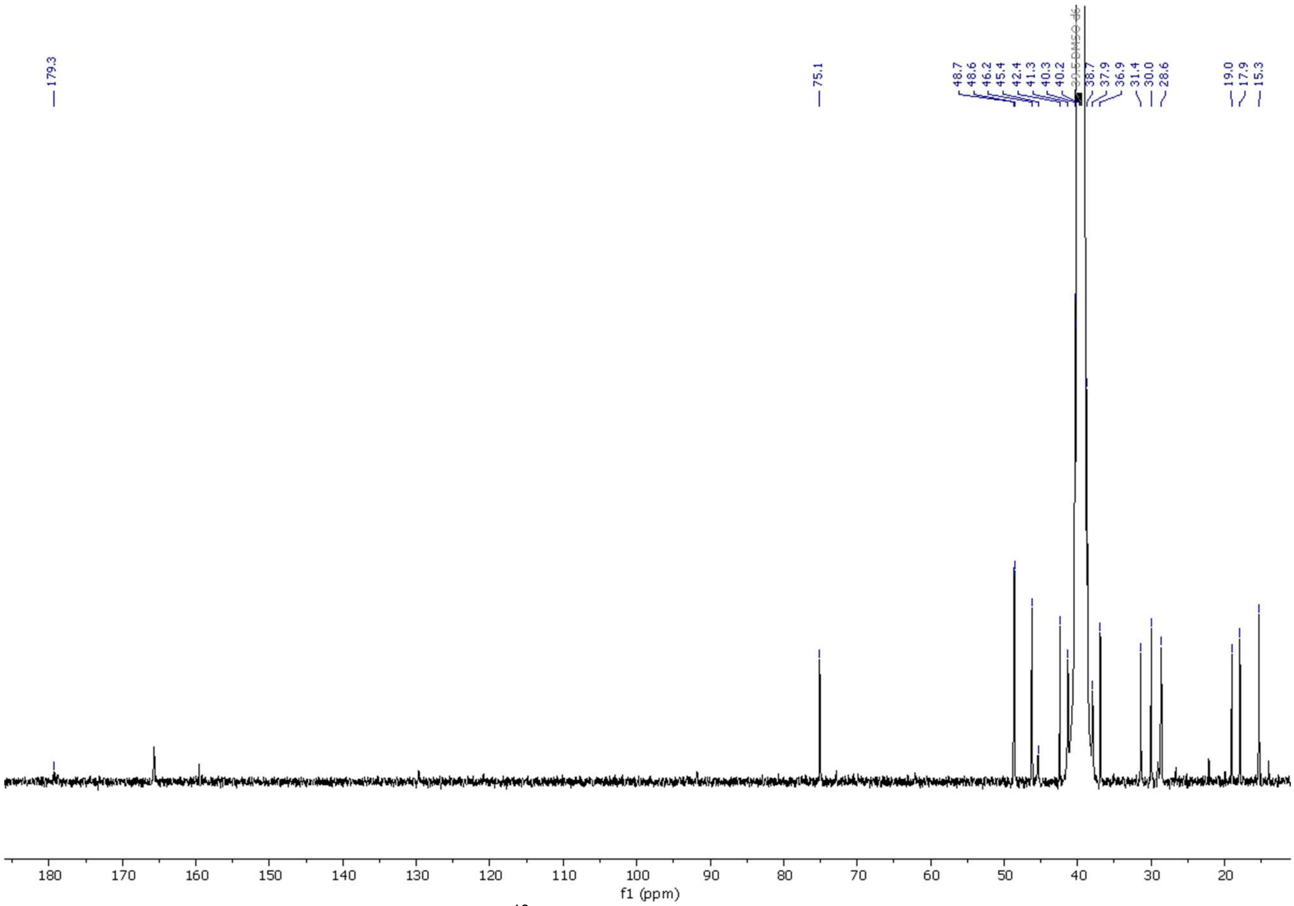

Figure N32.C ^{13}C NMR of **32** in DMSO-d$_6$ at 151 MHz.

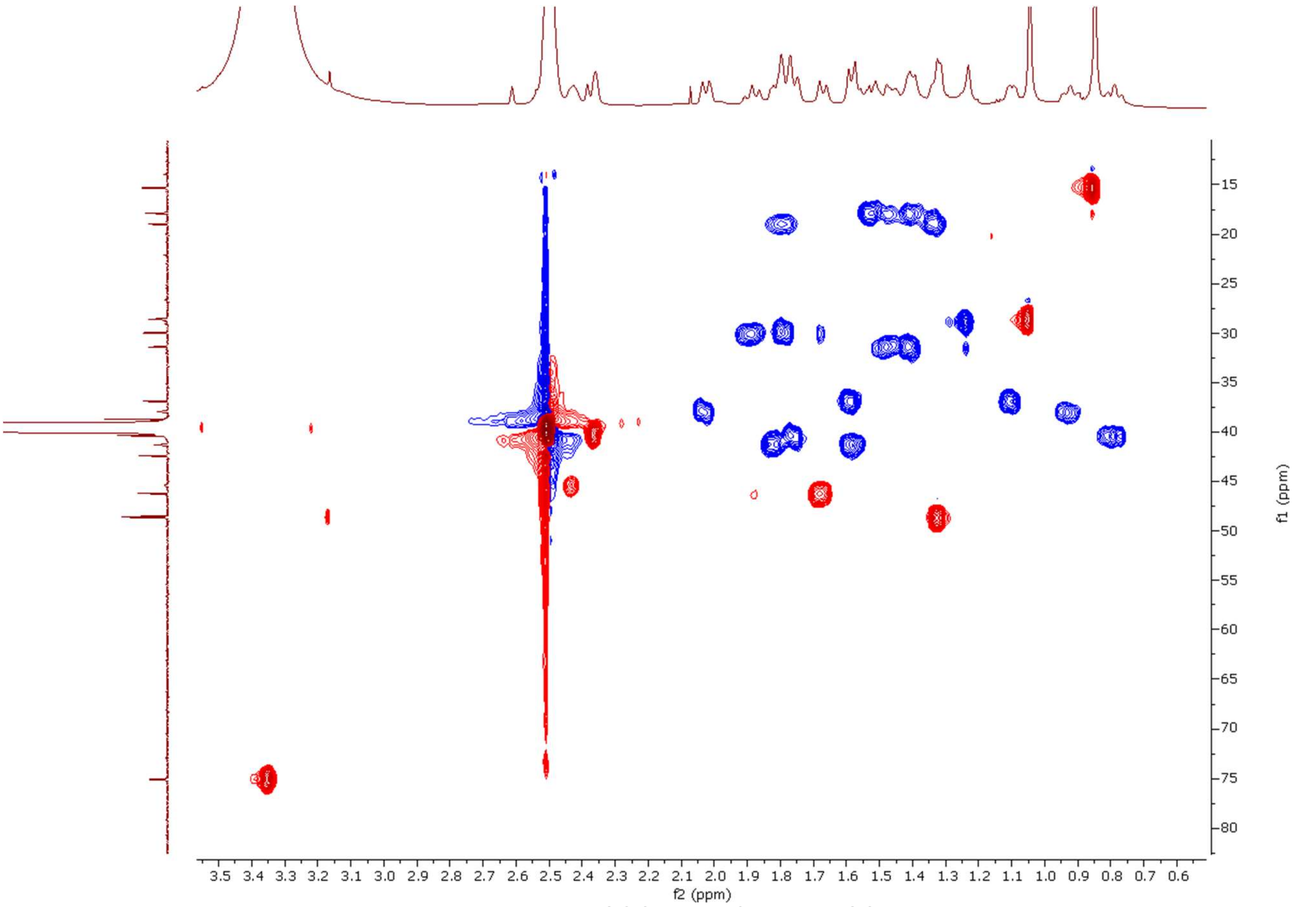

Figure N32.D HSQC NMR of **32** in DMSO-d$_6$.

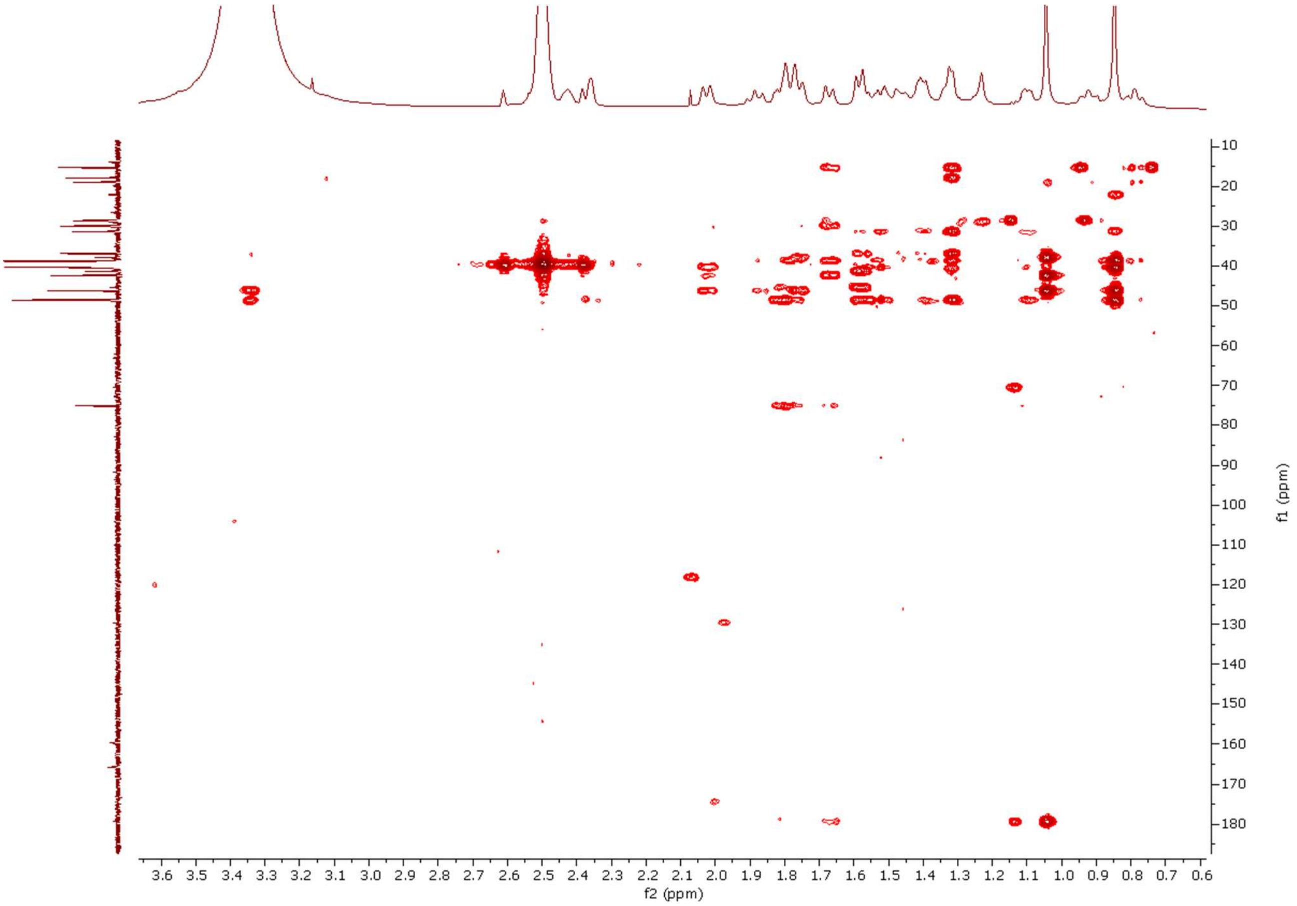

Figure N32.E HMBC NMR of **32** in DMSO-d$_6$.

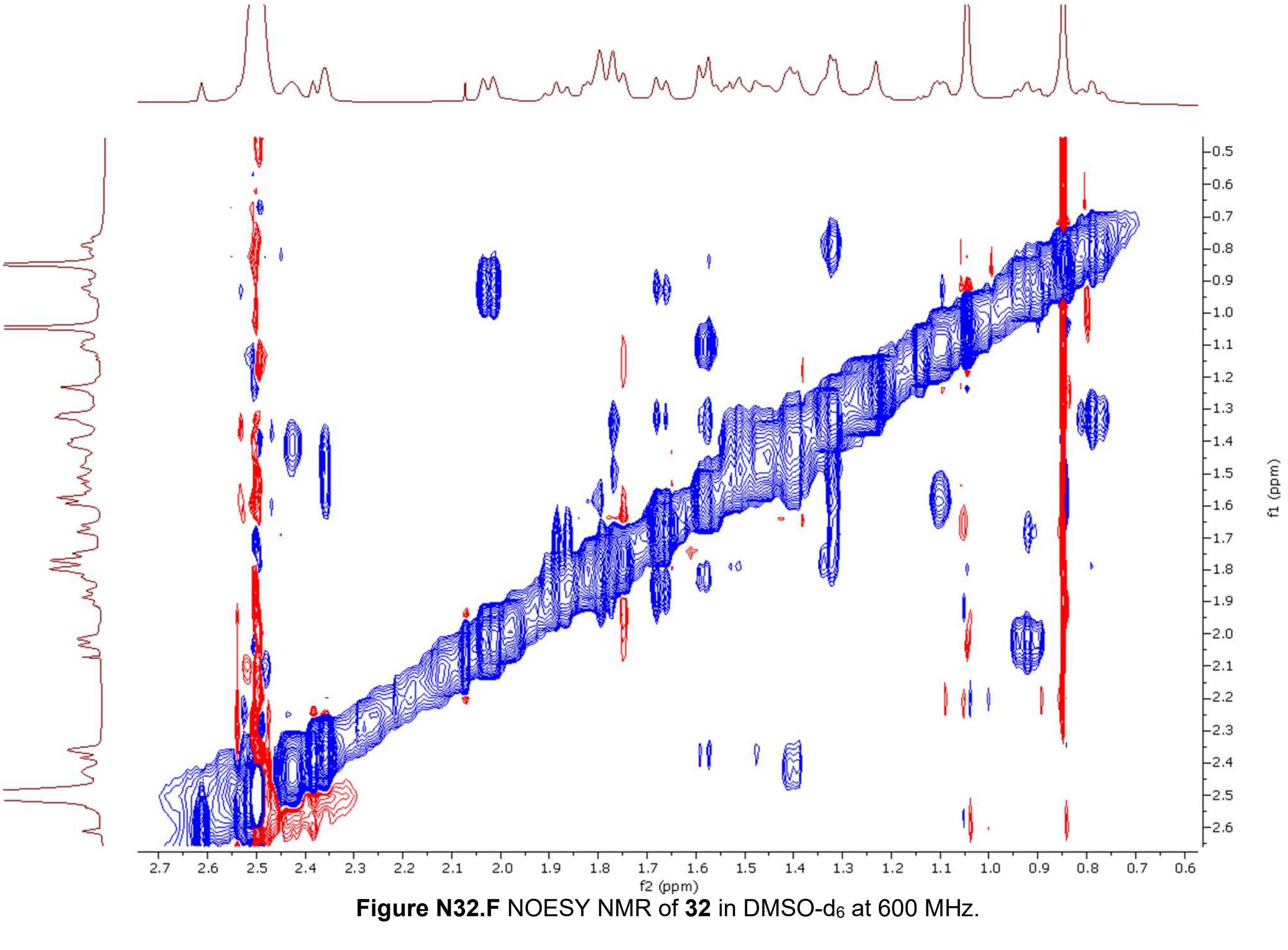

Figure N32.F NOESY NMR of **32** in DMSO-d$_6$ at 600 MHz.

7β-Hydroxy-16-*epi*-*ent*-kauran-17,19-dioic acid dimethyl ester (32a)

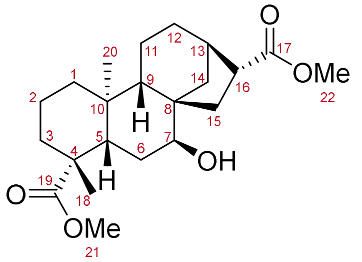

7β-Hydroxy-16-*epi*-*ent*-kauran-17,19-dioic acid dimethyl ester (**32a**)

Chemical Formula: $C_{22}H_{34}O_5$
Exact Mass: 378.2406

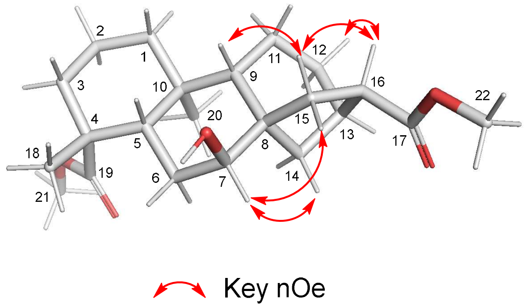

	Measured in $CDCl_3$, 298K		
**Pos.**	δ_C 151 MHz	δ_H (*J/Hz*) 600 MHz	**Selected NOESY correlations to H** [a]
**1**	40.5	ax 0.91, ddd (13.5, 13.5, 4.1)<br>eq 1.83, overlapped	H-2a, H-1eq, H-5<br>H-1eq
**2**	19.3	a 1.45, overlapped<br>b 1.83, overlapped	H-1ax
**3**	38.1	ax 1.07, ddd (13.6, 4.5, 4.5)<br>eq 2.19, ddd (13.6, 3.3, 3.3)	H-3eq, H-5<br>H-3ax, H_3-18
**4**	43.5	-	-
**5**	47.3	1.72, overlapped	H-3ax, H_3-18, H-1ax, H-9
**6**	29.8	a 2.00, m<br>b 2.03, m	H_3-18, H-7<br>H_3-20, H-7
**7**	77.2	3.68, dd (2.8, 2.8)	H-15b, H-6a, H-6b, H-14a
**8**	49.0	-	-
**9**	49.2	1.44, overlapped	H-5, H-15a
**10**	39.2	-	-
**11**	18.3	1.66, m	H-9
**12**	31.7	a 1.52, overlapped<br>b 1.57, overlapped	H-13<br>H-13
**13**	41.2	2.49, dd (7.2, 3.6)	H-14a, H-12a, H-12b, H-14b, H-16
**14**	37.3	a 1.28, overlapped<br>b 1.73, overlapped	H-13, H-14b<br>H-13, H-14a
**15**	41.2	a 1.79, overlapped<br>b 1.91, dd (13.5, 5.5)	H-16, H-15b, H-9<br>H-15a, H-7
**16**	45.6	2.65, dd (9.3, 5.5)	H-13, H-15a, H-9, H-12a
**17**	177.8	-	-
**18**	28.7	1.16, s	H-3eq, H-5
**19**	178.3	-	-
**20**	15.3	0.81, s	H-6b, H-1eq, H_3-21
**21**	51.4	3.65, s	H_3-20
**22**	51.9	3.67, s	

[a] Key NOESY correlations are shown in blue text.

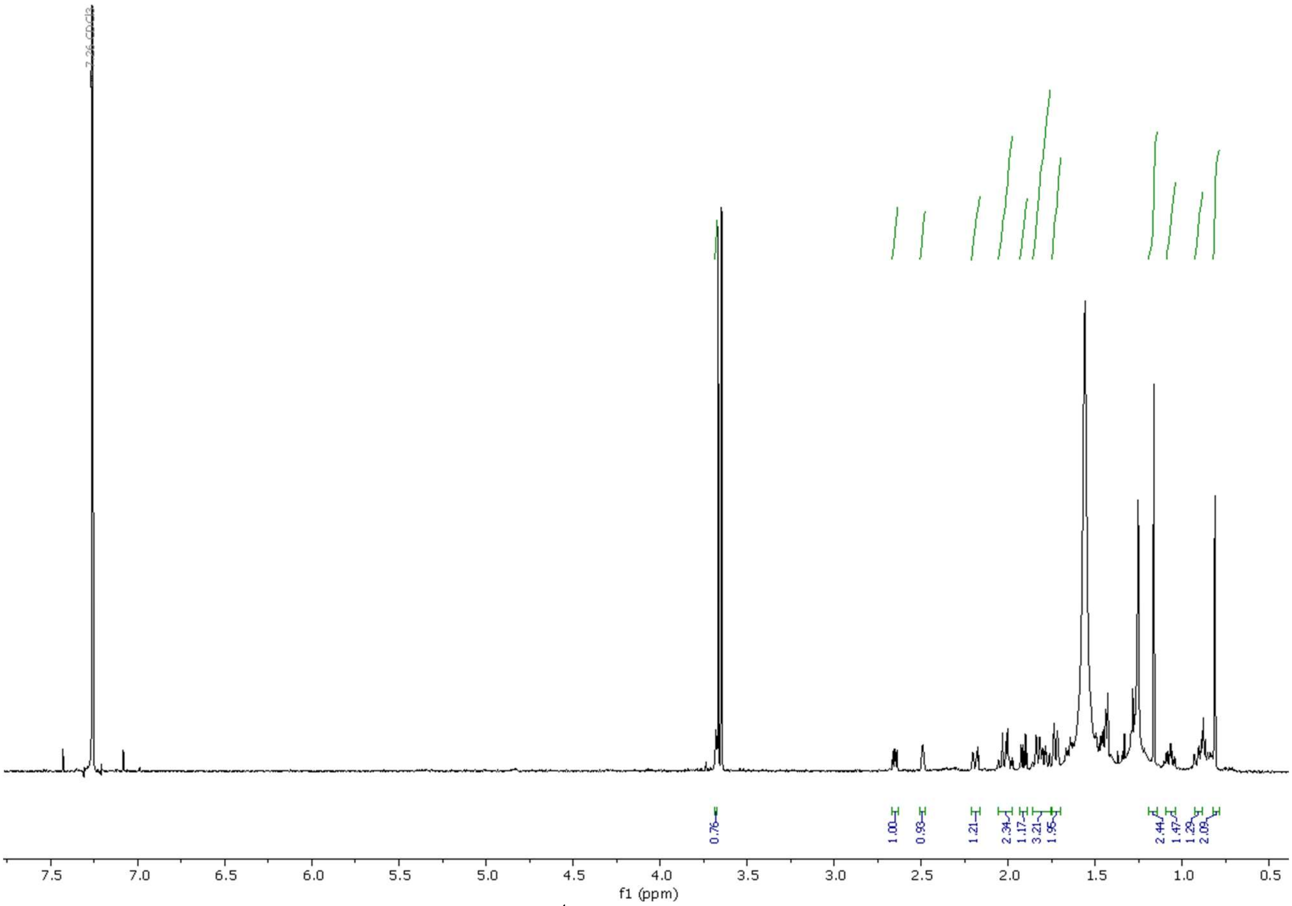

Figure N32a.A ^{1}H NMR of **32a** in $CDCl_3$ at 600 MHz.

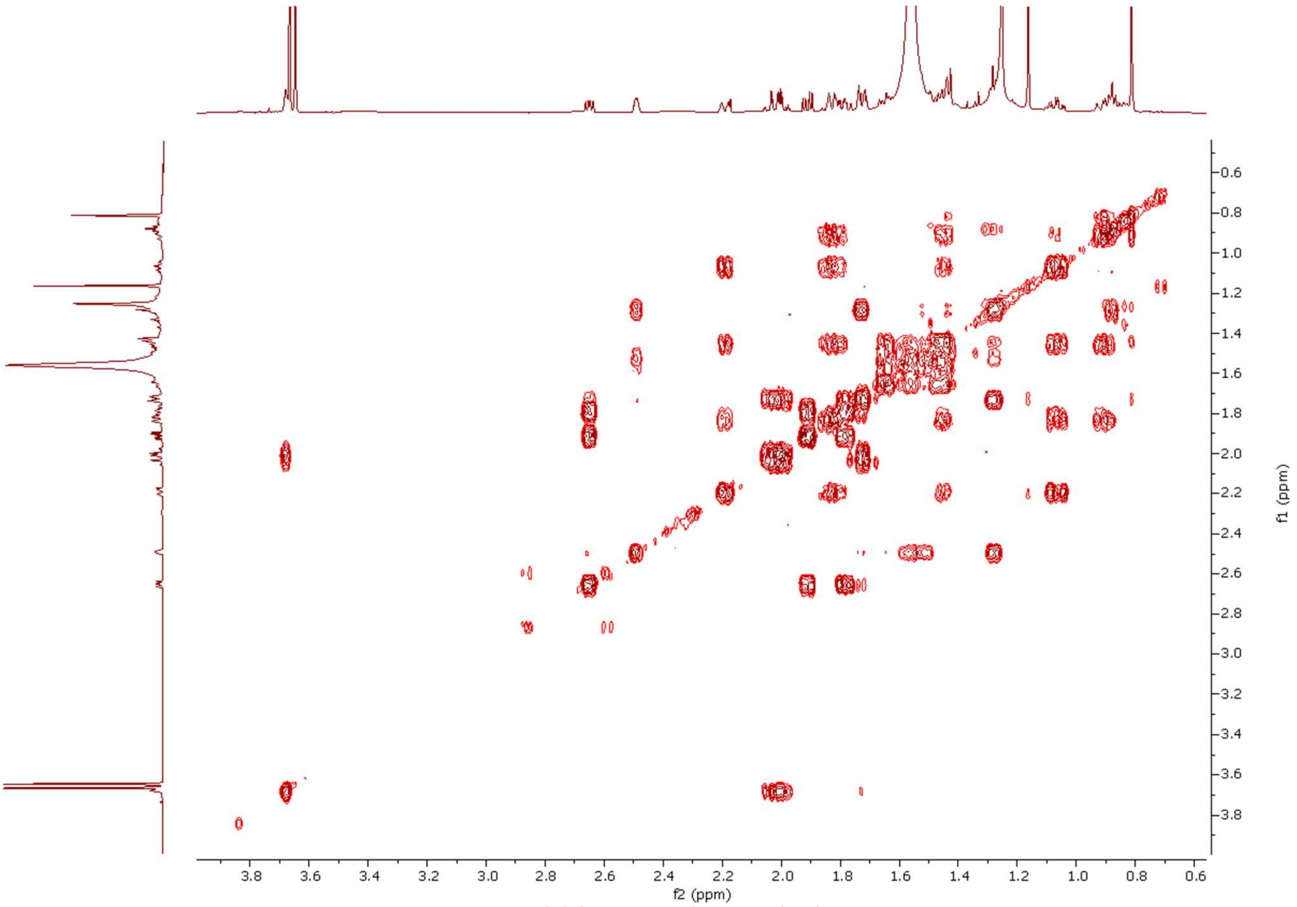

Figure N32a.B COSY NMR of **32a** in $CDCl_3$ at 600 MHz.

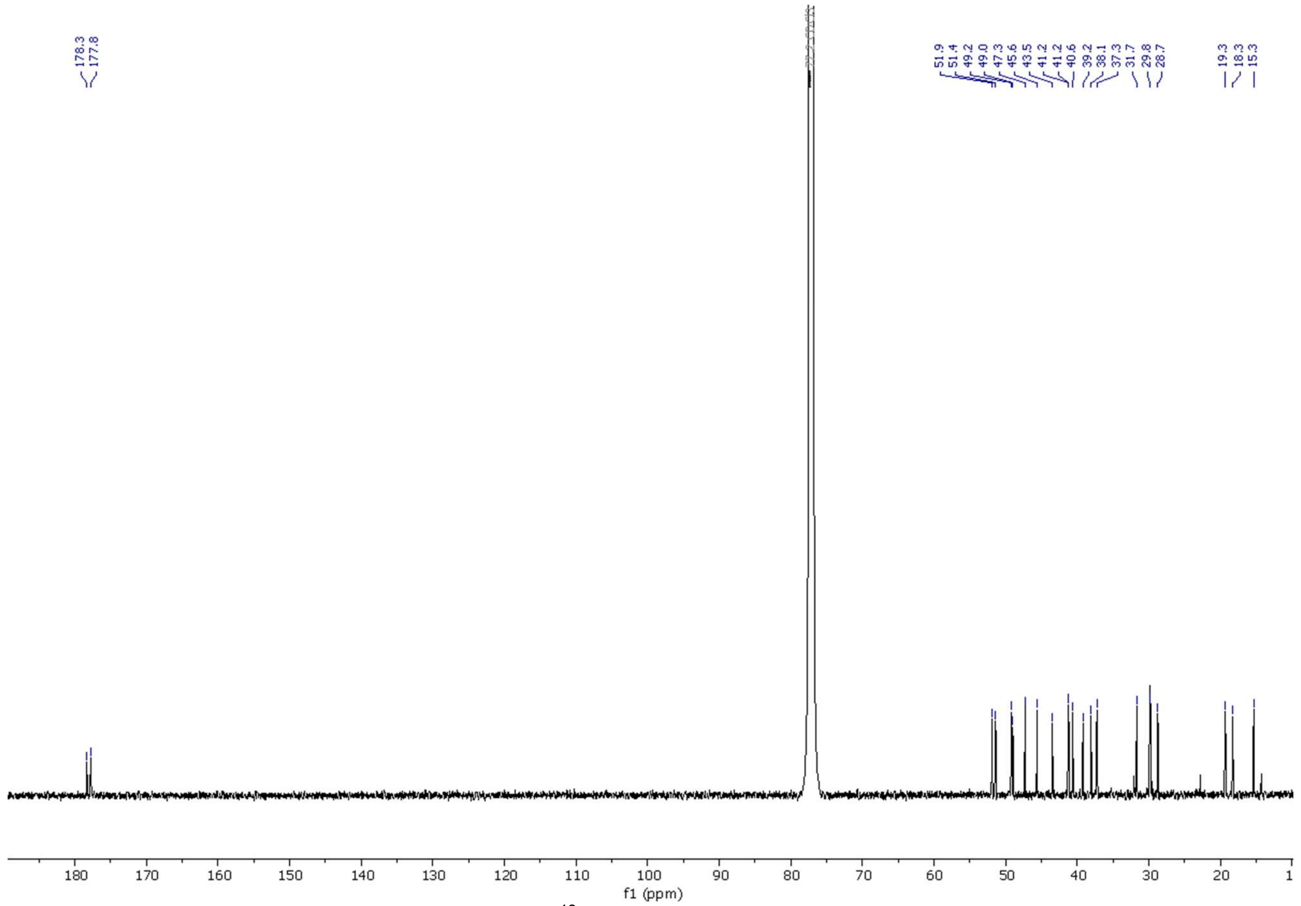

Figure N32a.C ^{13}C NMR of **32a** in $CDCl_3$ at 151 MHz.

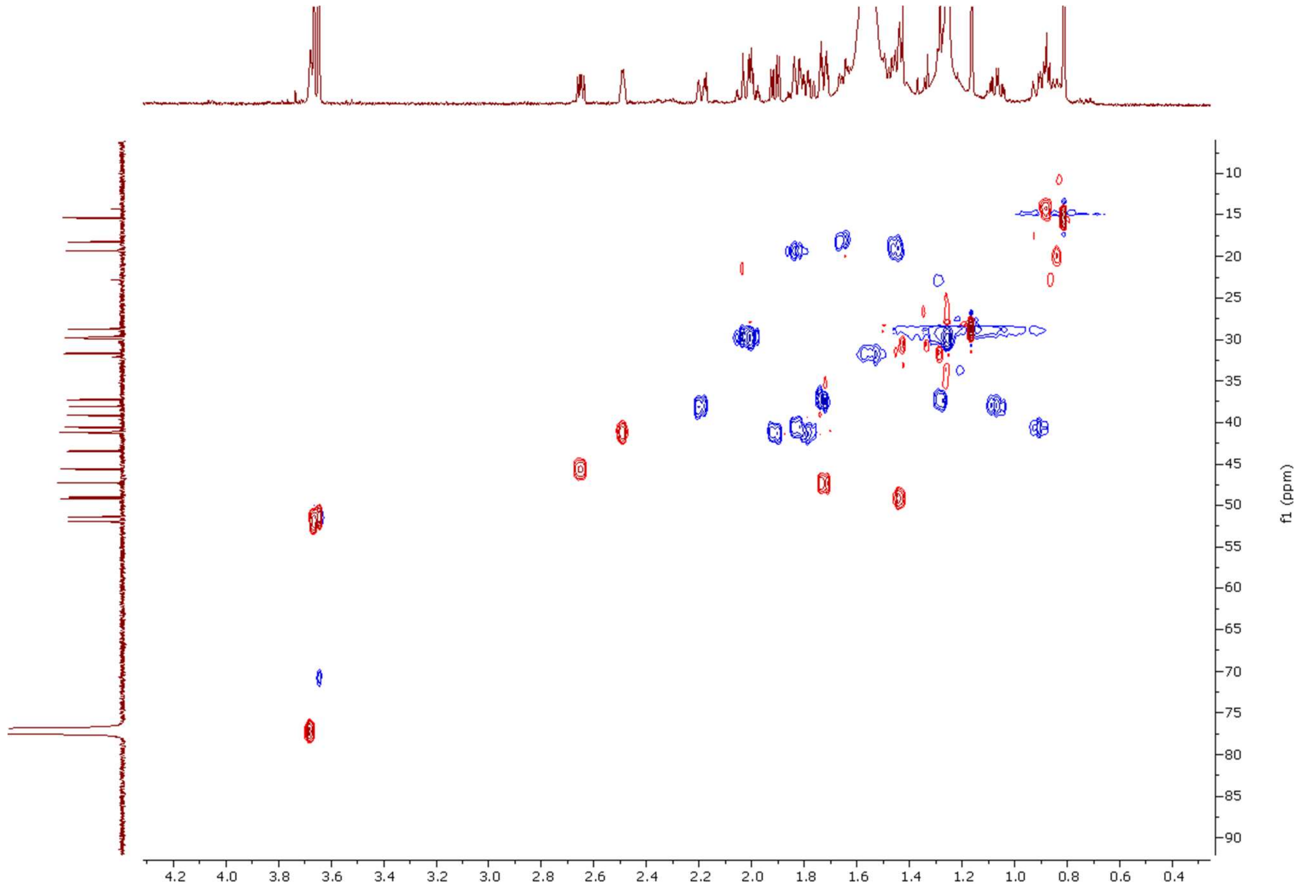

Figure N32a.D HSQC NMR of **32a** in $CDCl_3$.

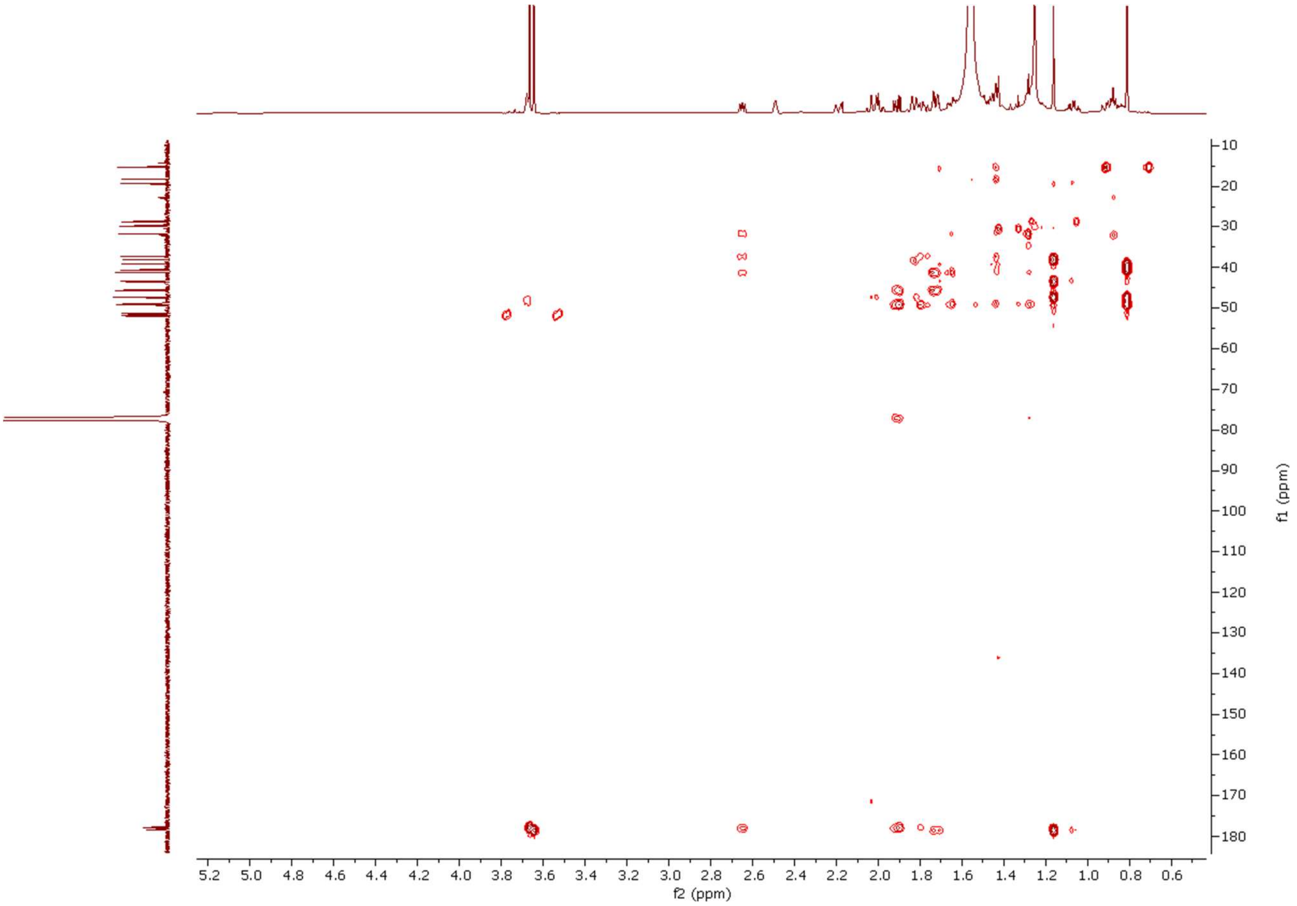

Figure N32a.E HMBC NMR of **32a** in $CDCl_3$.

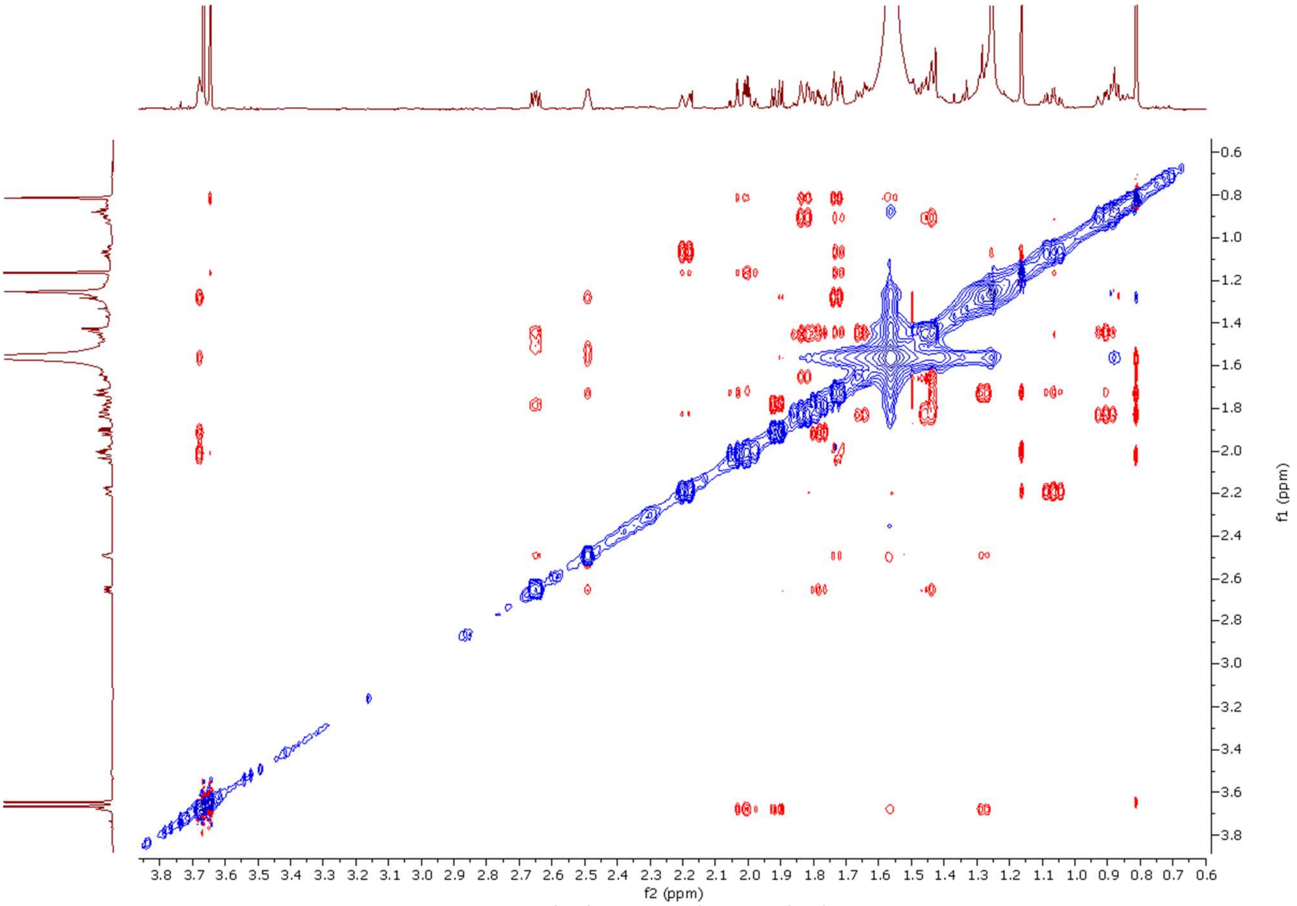

Figure N32a.F NOESY NMR of **32a** in $CDCl_3$ at 600 MHz.

6β,7β,17-Trihydroxy-16-*epi*-*ent*-kauranoic acid (33)

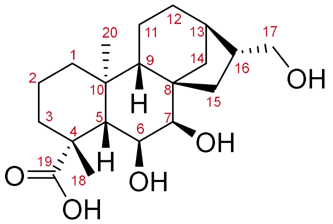

6β,7β,17-Trihydroxy-16-*epi*-*ent*-kauranoic acid (**33**)

Chemical Formula: $C_{20}H_{32}O_5$

Exact Mass: 352.2250

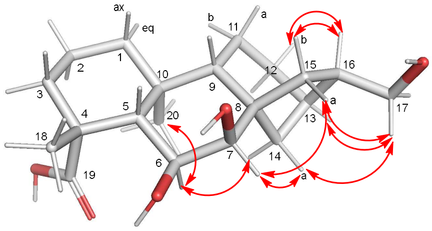

Key nOe

	Measured in d_4-methanol, 298K		
**Pos.**	δ_C 151 MHz	δ_H (*J/Hz*) 600 MHz	**Selected NOESY correlations to H** [a]
**1**	42.1	ax 0.88, ddd (14.6, 13.9, 4.0)<br>eq 1.85, overlapped	H-5
**2**	20.3	a 1.36, overlapped<br>b 1.87, overlapped	
**3**	41.1	ax 1.07, ddd (14.6,13.3, 4.0)<br>eq 2.12, m overlapped	H-5
**4**	45.3	-	-
**5**	52.6	1.80, d (11.2)	H-3ax, H-1ax
**6**	73.3	4.28, dd (11.2, 2.2)	H-14b, H$_3$-20, H-7
**7**	83.1	3.42, d (2.2)	H-14a, H-15a, H-6
**8**	50.2	-	-
**9**	50.3	1.36, overlapped	H-5, H-11a
**10**	41.9	-	-
**11**	19.5	a 1.56, overlapped<br>b 1.63, m	 H-1eq
**12**	32.9	a 1.42, overlapped<br>b 1.55, overlapped	H-16, H-13<br>H-13
**13**	39.4	2.12, overlapped	H-14a, H-14b, H-16, H-12a, H-12b, H$_2$-17
**14**	37.4	a 1.15, overlapped<br>b 1.75, dd (11.2, 1.0)	H-13<br>H-13
**15**	42.6	a 1.17, dd (13.9, 5.3)<br>b 1.71, ddd (13.9, 8.7, 2.1)	H-7, H$_2$-17<br>H-16
**16**	44.2	1.93, m	H-12a, H-11a, H-15b, H-13, H$_2$-17
**17**	67.6	3.32 overlapped	H-13, H-16, H-15a, H-14a
**18**	33.6	1.42, s	
**19**	183.8	-	-
**20**	17.3	1.00, s	H-6

[a] Key NOESY correlations are shown in blue text.

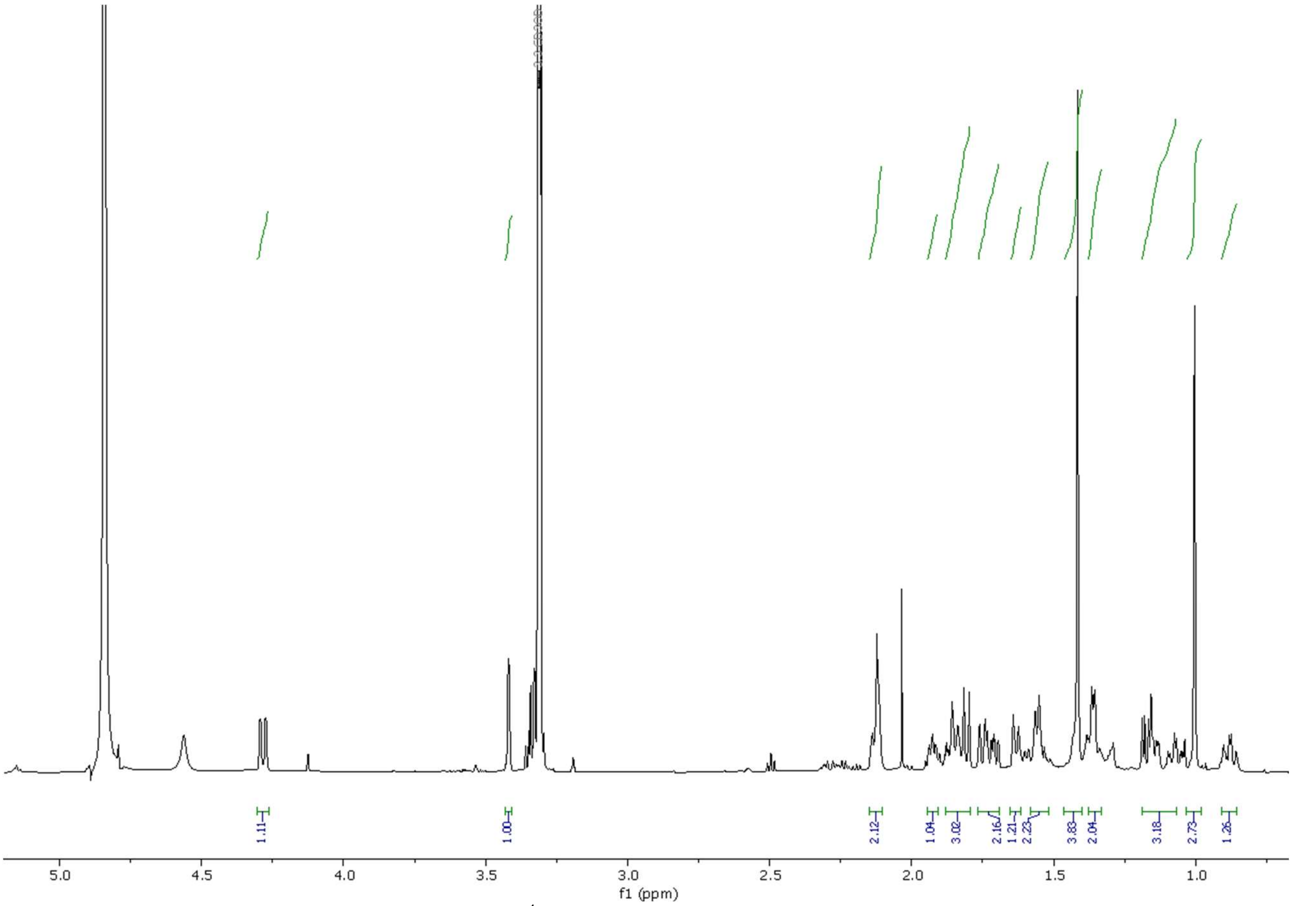

Figure N33.A ^{1}H NMR of **33** in d$_4$-methanol at 600 MHz.

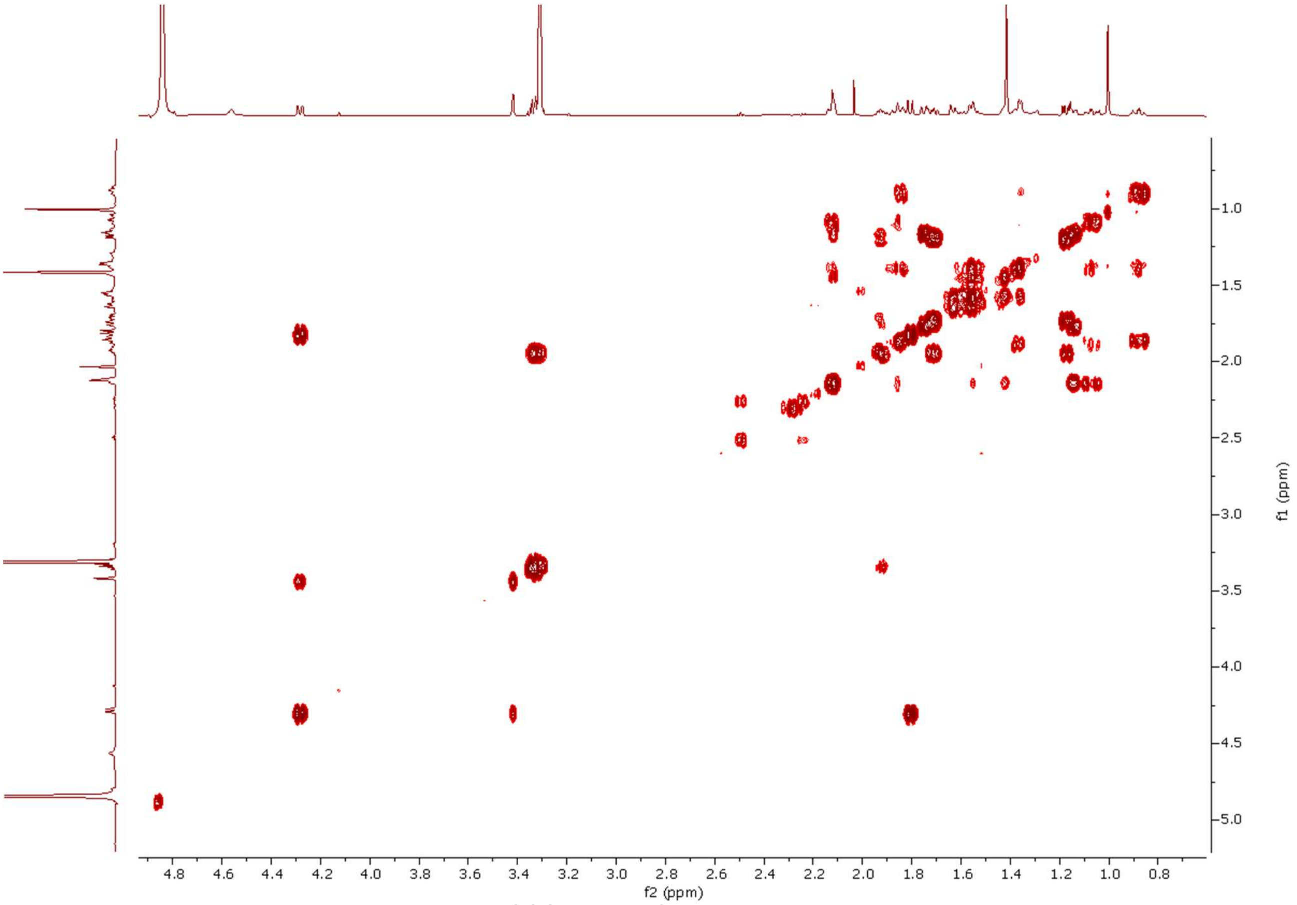

Figure N33.B COSY NMR of **33** in d$_4$-methanol at 600 MHz.

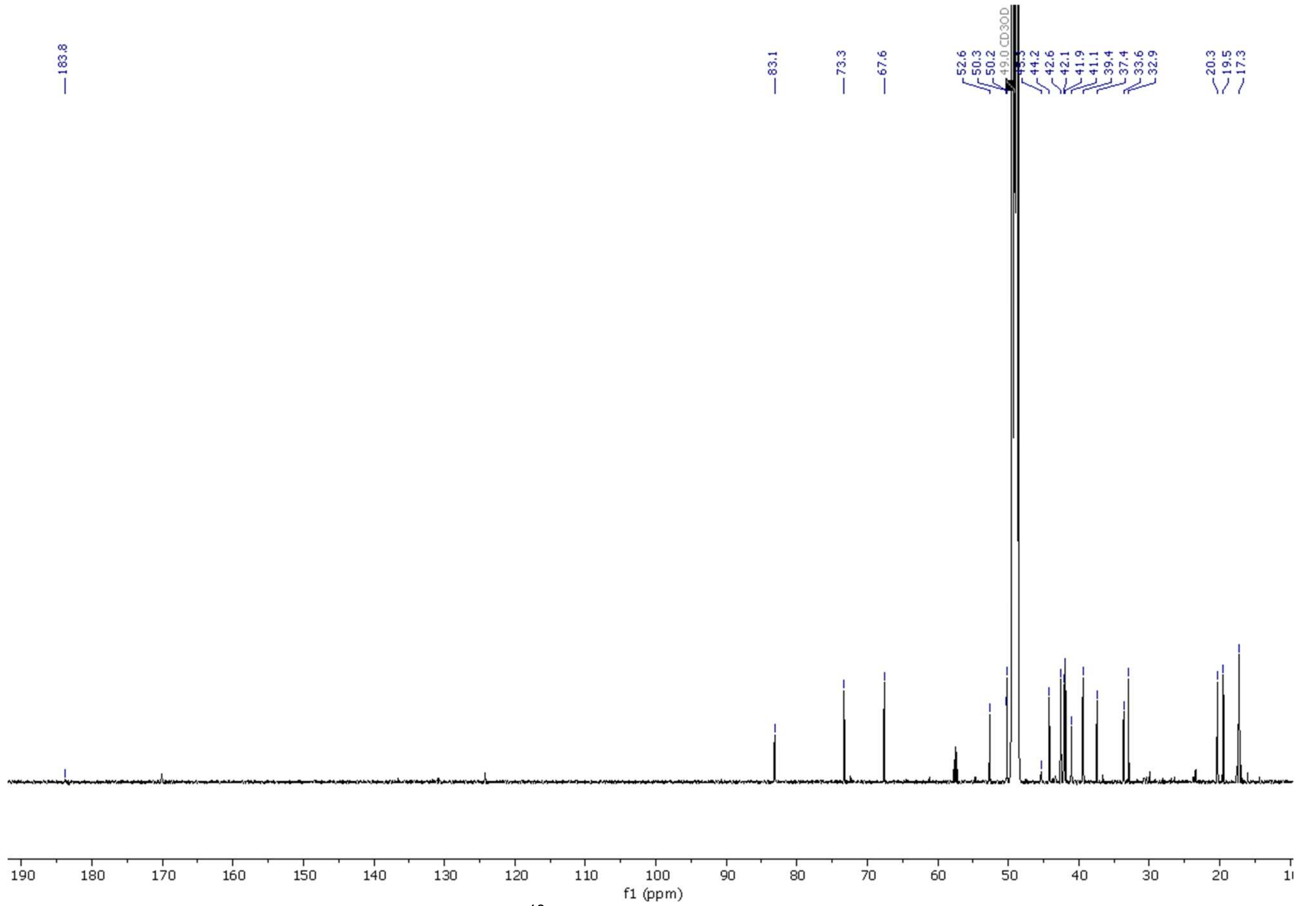

Figure N33.C ^{13}C NMR of **33** in d_4-methanol at 151 MHz.

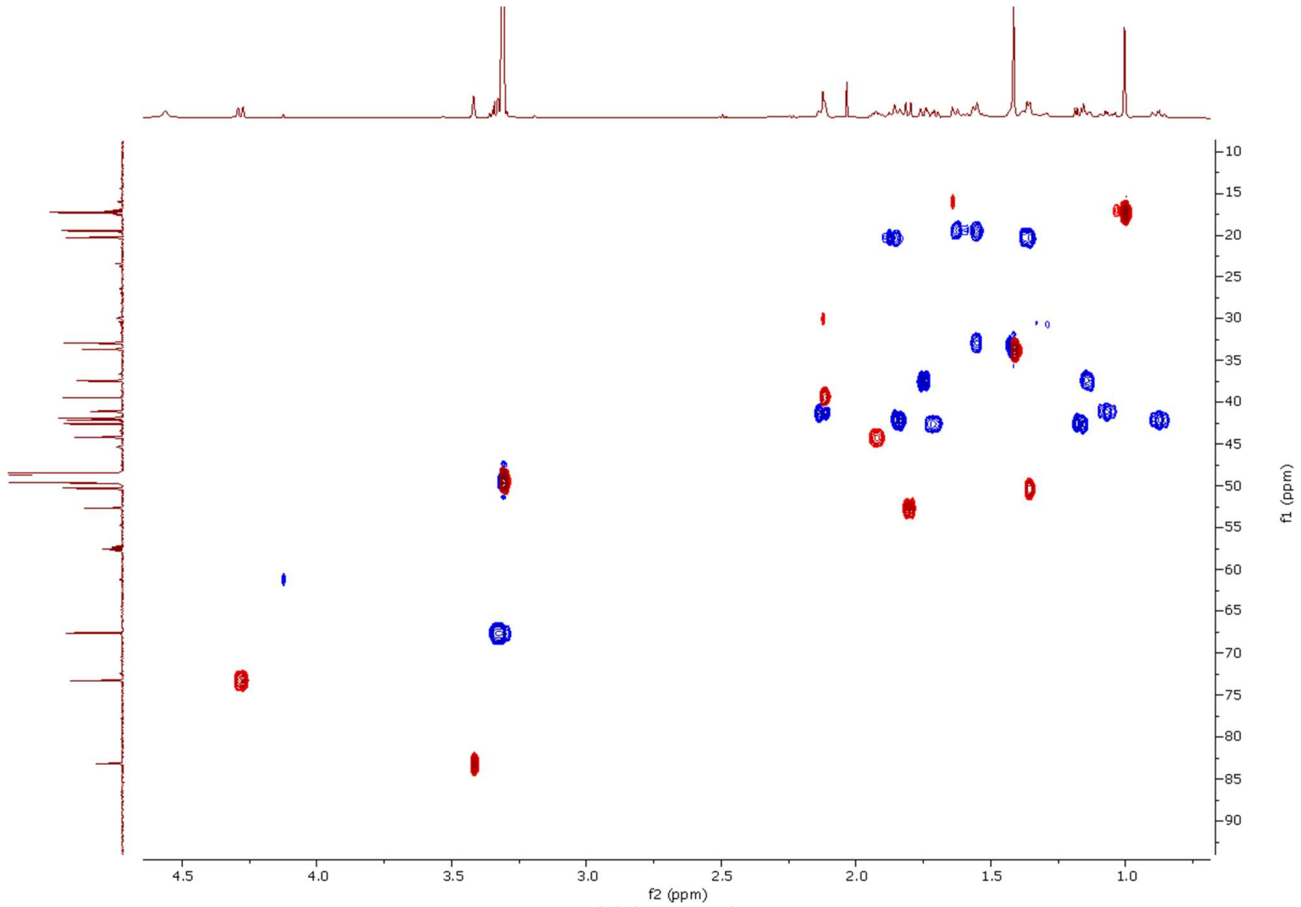

Figure N33.D HSQC NMR of **33** in d_4-methanol.

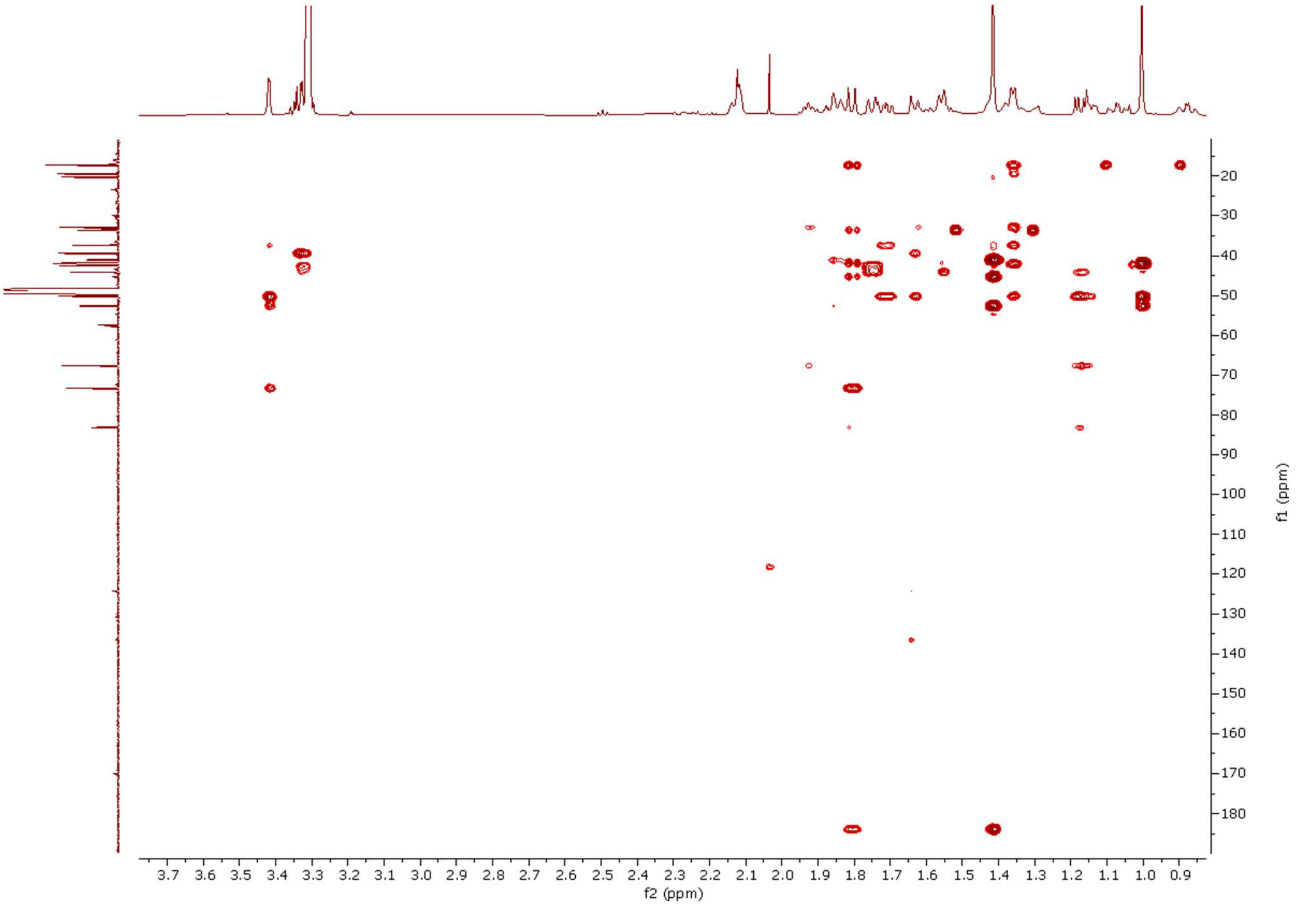

Figure N33.E HMBC NMR of **33** in d$_4$-methanol.

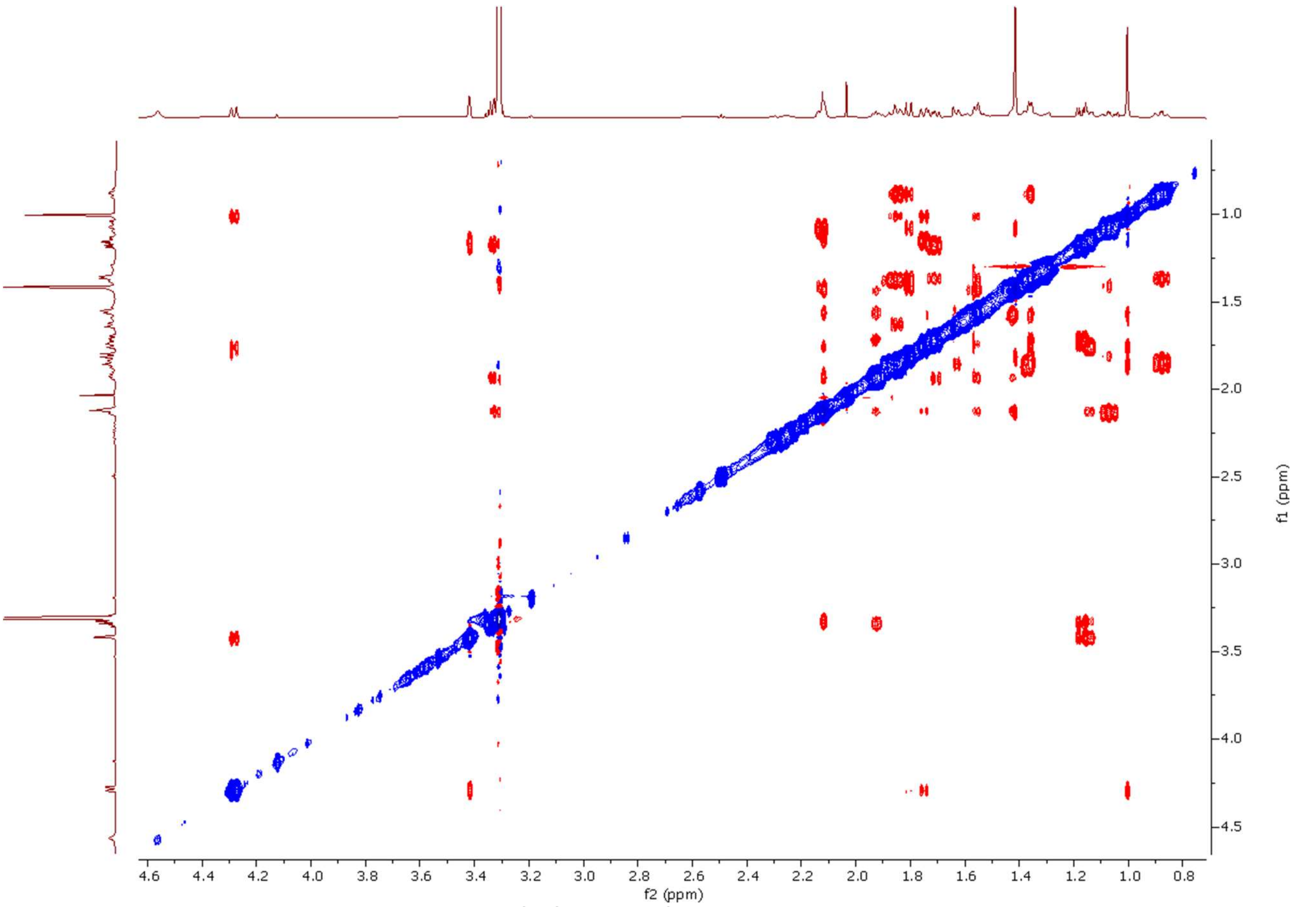

Figure N33.F NOESY NMR of **33** in d$_4$-methanol at 600 MHz.

6β,7β,13-Trihydroxy-*ent*-kaurenoic acid (34)

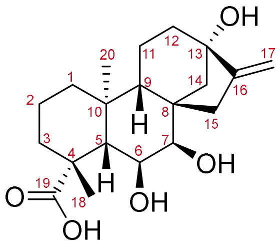

6β,7β,13-Trihydroxy-*ent*-kaurenoic acid (**34**)
Chemical Formula: $C_{20}H_{30}O_5$
Exact Mass: 350.2093

	Measured in C_5D_5N, 298K		
**Pos.**	δ_C 151 MHz	δ_H (*J/Hz*) 600 MHz	**Selected NOESY correlations to H** [a]
**1**	41.5	ax 1.01, ddd (14.4, 13.7, 2.5)<br>eq 1.89, overlapped	H-1eq, H-9<br>H-1ax
**2**	20.2	a 1.50, br d (13.7)<br>b 2.21, m	H-2b<br>H-2a
**3**	40.9	ax 1.24, overlapped<br>eq 2.52, br d (13.4)	H-3eq<br>H-3ax
**4**	44.9	-	-
**5**	52.4	2.43, d (10.9)	H-1ax, H-3ax, H-9
**6**	72.3	4.94, d (10.9)	H-7, H-20, H-14b
**7**	82.2	3.99, br s	H-15a, H-14a
**8**	46.9	-	-
**9**	48.6	1.80, overlapped	H-1ax, H-5, H-15b
**10**	41.5	-	-
**11**	21.1	1.82, overlapped	
**12**	41.7	1.89, overlapped<br>2.10, ddd (13.5, 13.0, 6.0)	
**13**	80.1	-	-
**14**	46.7	a 1.77, d (10.8)<br>b 2.32, d (10.8)	H-14b<br>H-14a
**15**	45.7	b 2.75, d (17.3)<br>a 2.88, d (17.3)	H-15a<br>H-15b
**16**	157.8	-	
**17**	103.4	5.08, br s<br>5.50, br s	H-15a, H-15b<br>H-12a
**18**	33.6	1.87, s	
**19**	181.8	-	
**20**	17.5	1.30, s	

[a] Key NOESY correlations are shown in blue text.

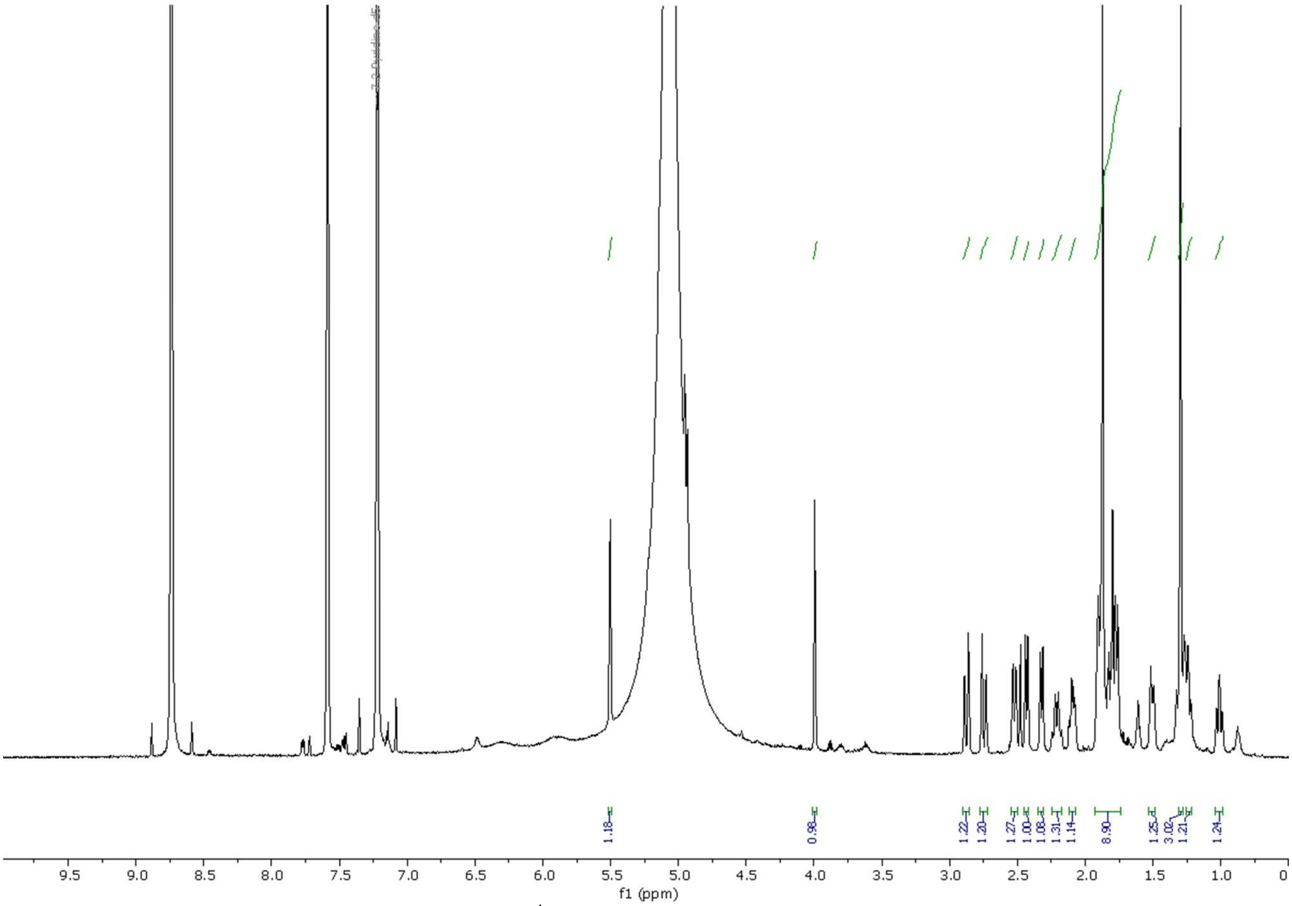

Figure N34.A ^{1}H NMR of **34** in pyridine-d_5 at 600 MHz.

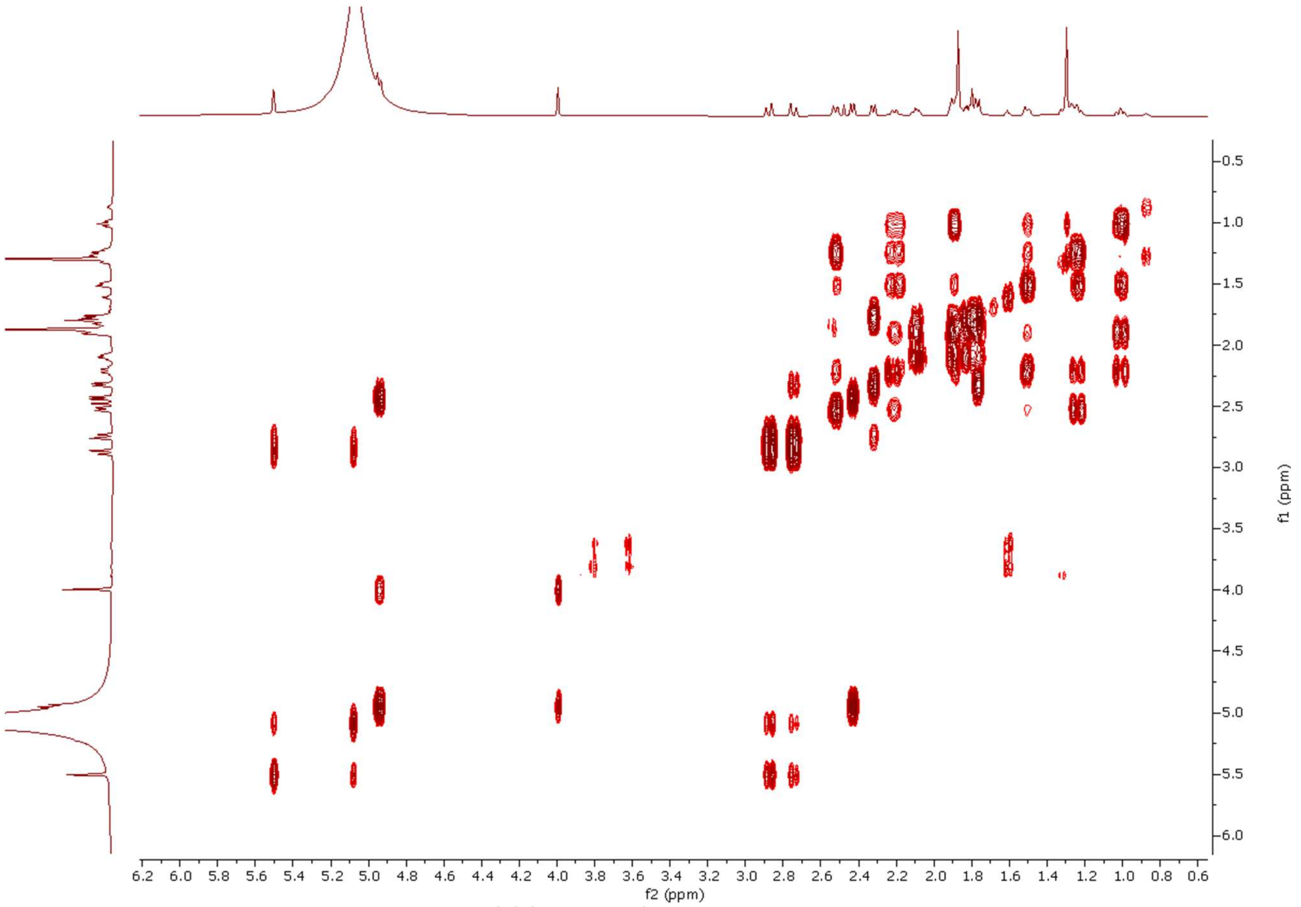

Figure N34.B COSY NMR of **34** in pyridine-d_5 at 600 MHz.

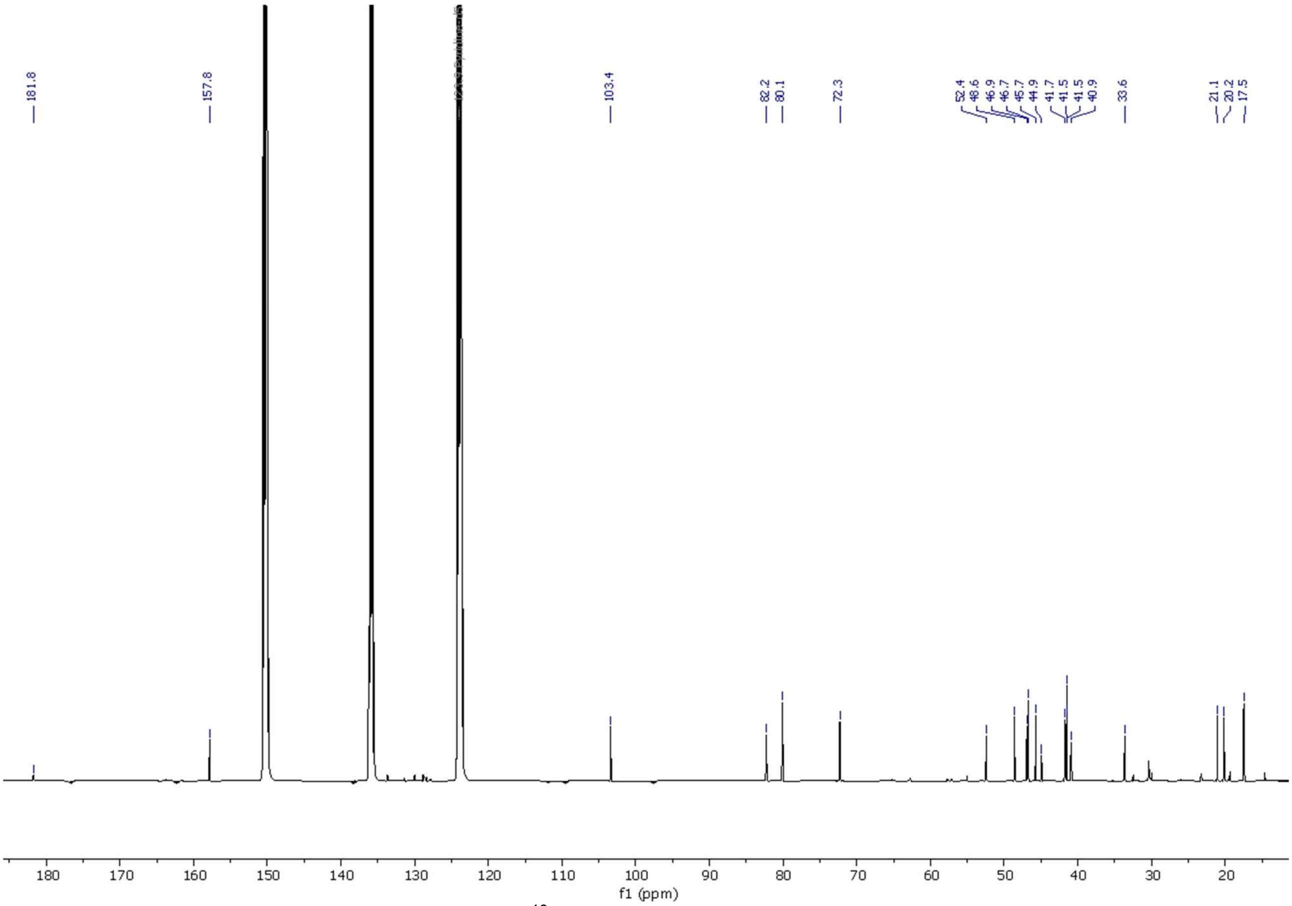

Figure N34.C ^{13}C NMR of **34** in pyridine-d_5 at 151 MHz.

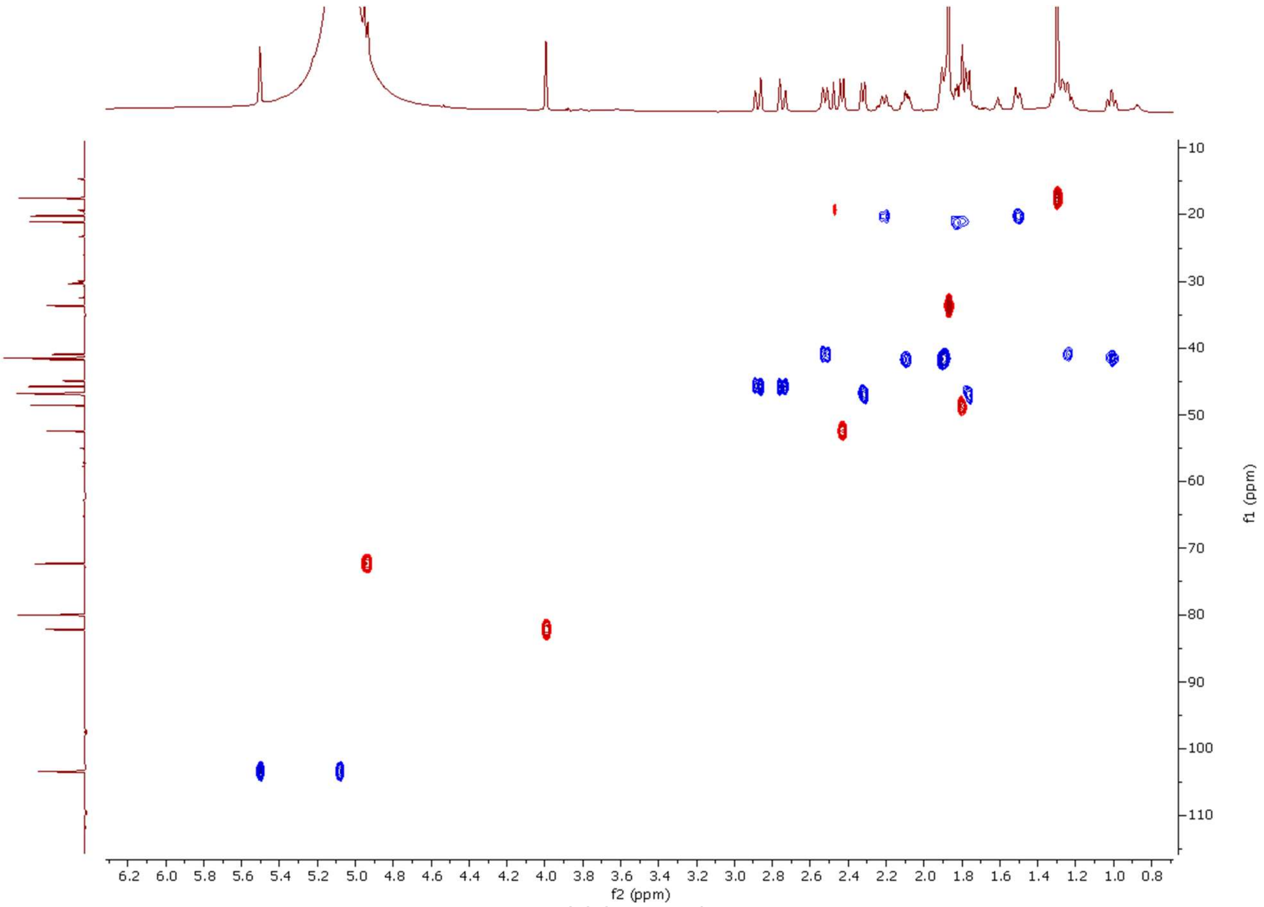

Figure N34.D HSQC NMR of **34** in pyridine-d_5.

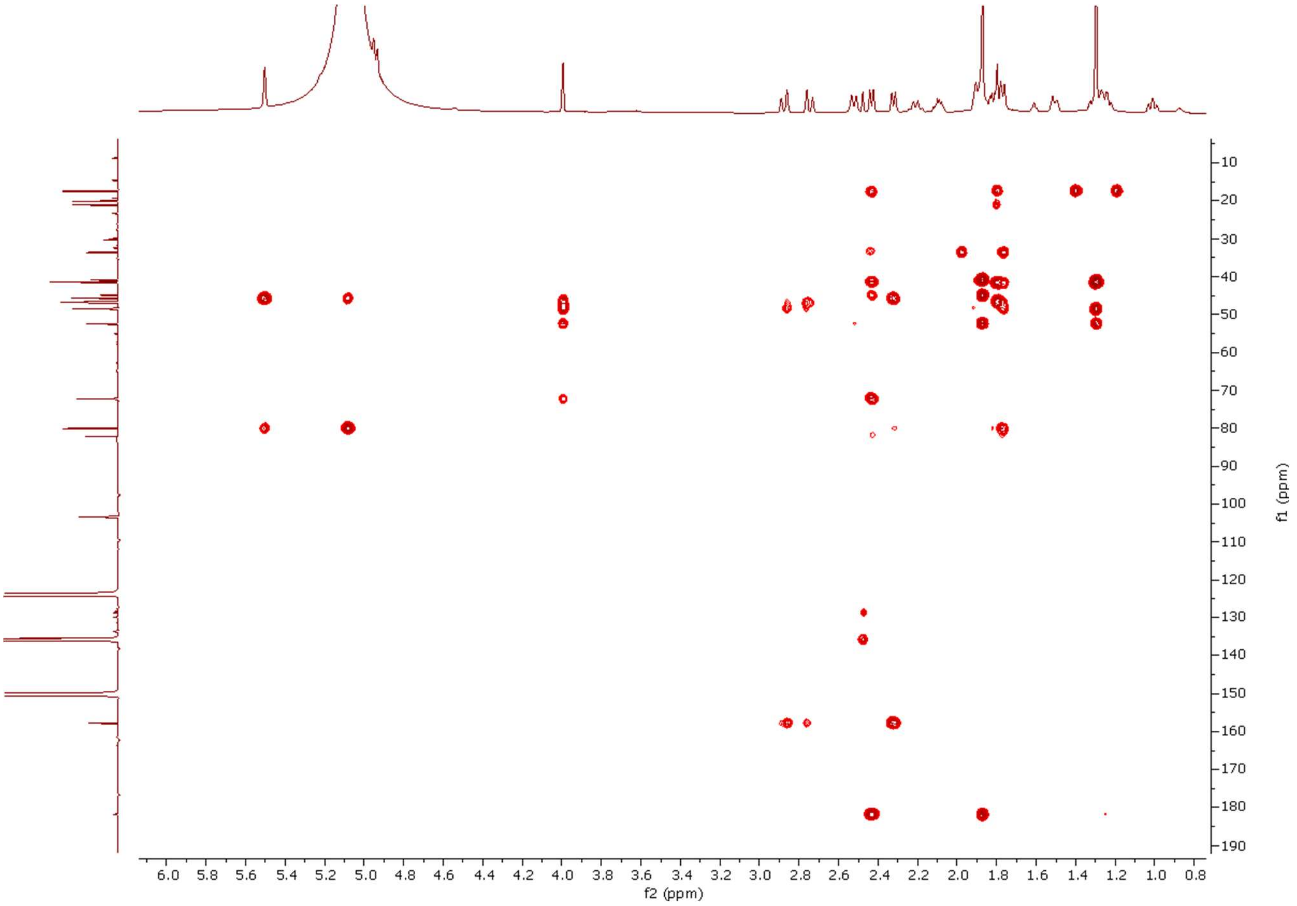

Figure N34.E HMBC NMR of **34** in pyridine-d5.

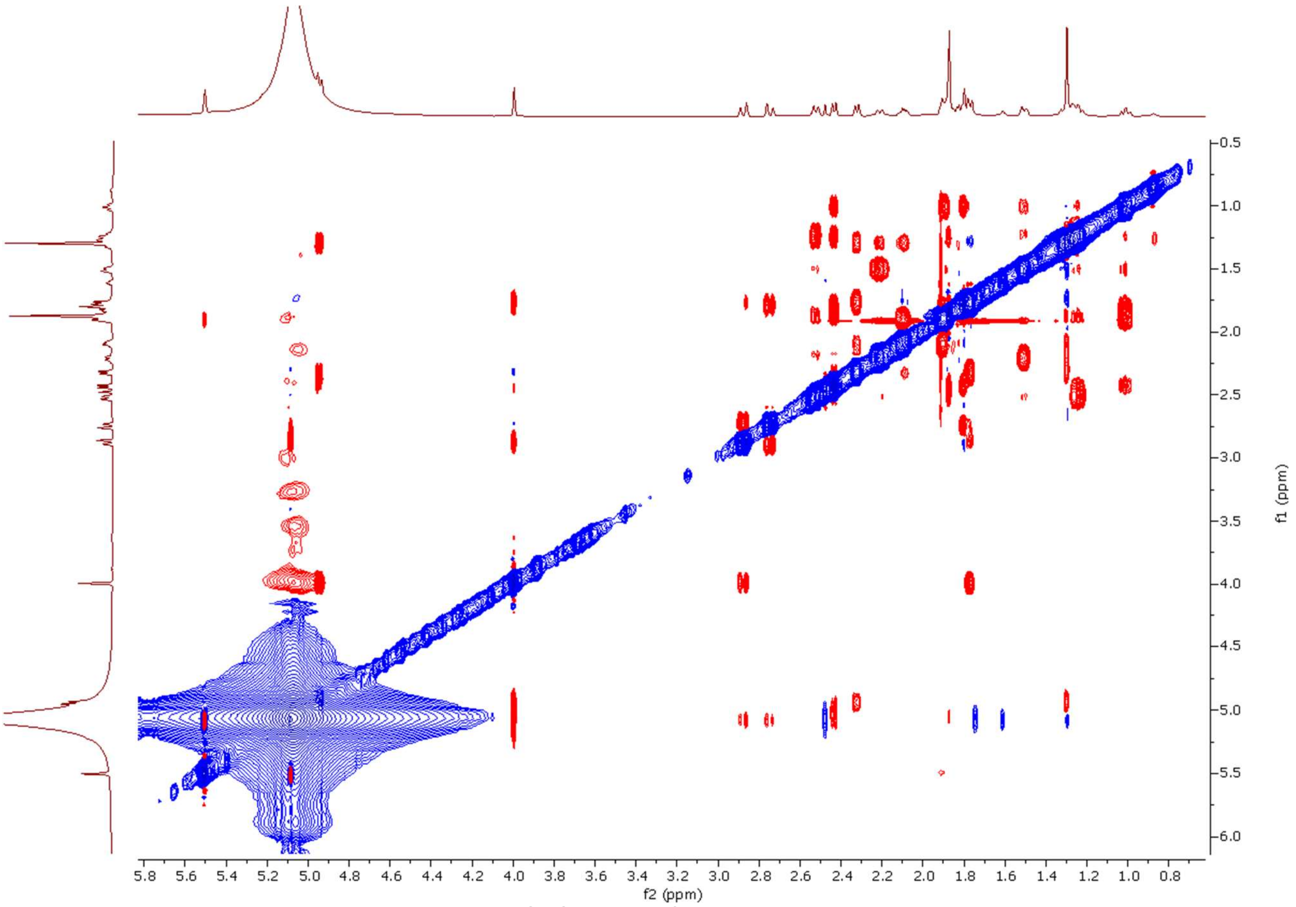

Figure N34.F NOESY NMR of **34** in pyridine-d5 at 600 MHz.

15β-Hydroxy-*ent*-kaurenoic acid (35)

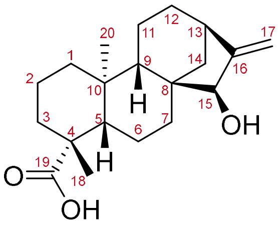

15β-Hydroxy-*ent*-kaurenoic acid (**35**)

Chemical Formula: $C_{20}H_{30}O_3$

Exact Mass: 318.2195

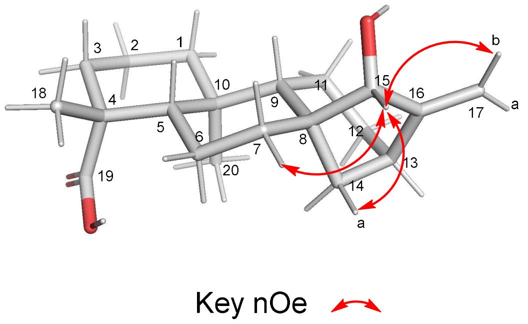

Pos.	Reference in $CDCl_3$ [52]		Reference in $CDCl_3$ [47] (only δ_C reported)	Measured in $CDCl_3$, 298K		
	δ_C 100 MHz	δ_H (*J*/Hz) 400 MHz	δ_C 50.1MHz	δ_C 151 MHz	δ_H (*J*/Hz) 600 MHz	Selected NOESY correlations to H [a]
**1**	39.6	1.88, m<br>1.12, m	40.6	40.7	ax 0.90, ddd (13.2, 13.2, 4.0)<br>eq 1.92, ddd (13.2, 3.2, 3.1)	H-1eq, H-5<br>H-1ax
**2**	19.8	1.82, m<br>1.44, m	19.2	19.3	a 1.43, overlapped<br>b 1.87, overlapped	 H_3-20
**3**	36.6	2.10, m<br>0.96, m	38.0	37.9	ax 1.00, overlapped<br>eq 2.16, ddd (13.4, 3.8, 3.1)	H-3eq<br>H-3ax
**4**	42.6	-	43.7	43.9	-	
**5**	52.2	1.02, m	56.4	56.5	1.09, dd (12.5, 2.5)	H-1ax, H_3-18, H-9
**6**	17.9	1.81, m<br>1.64, m	21.6	21.6	a 1.81, m<br>b 1.86, overlapped	
**7**	35.1	2.35, m<br>1.86, m	38.9	39.1	a 1.35, overlapped<br>b 1.60, overlapped	H-15<br>H-15
**8**	46.6	-	45.7	45.9	-	
**9**	55.9	1.08, m	45.5	45.7	1.34, overlapped	H-5
**10**	38.7	-	39.3	39.5	-	
**11**	17.2	2.42, m<br>1.89, m	18.3	18.5	a 1.47, overlapped<br>b 1.57, overlapped	
**12**	31.5	1.55, m<br>1.40, m	33.1	33.3	a 1.47, overlapped<br>b 1.61, overlapped	H-17a
**13**	41.2	2.69, m	40.0	40.2	2.66, dd (4.7, 4.7)	H-17a
**14**	34.1	1.75, m<br>1.28, m	36.3	36.5	a 1.05, overlapped<br>b 1.99, d (12.0)	H-15<br>H_3-20
**15**	81.6	3.74, m	82.5	82.7	3.76, dd (2.7, 2.7)	H-14a, H-7a, H-7b, H-17b
**16**	159.1	-	158.3	158.5	-	
**17**	107.7	5.01, br s<br>5.13, br s	104.8	105.0	a 4.97, d (2.7)<br>b 5.09, br s	H-17b, H-13, H-12a<br>H-17a, H-15
**18**	27.8	1.19, s	29.0	29.1	1.24, s	H-5, H-3eq
**19**	182.9	-	183.1	183.9	-	
**20**	14.7	0.88, s	15.7	15.8	0.97, s	H-2b, H-14b

[a] Key NOESY correlations are shown in blue text.

The first reference shows noticeable deviations in both ^{1}H and ^{13}C chemical shifts, whereas the second reference closely matches our measured values. Our assignments are fully supported by 2D NMR experiments.

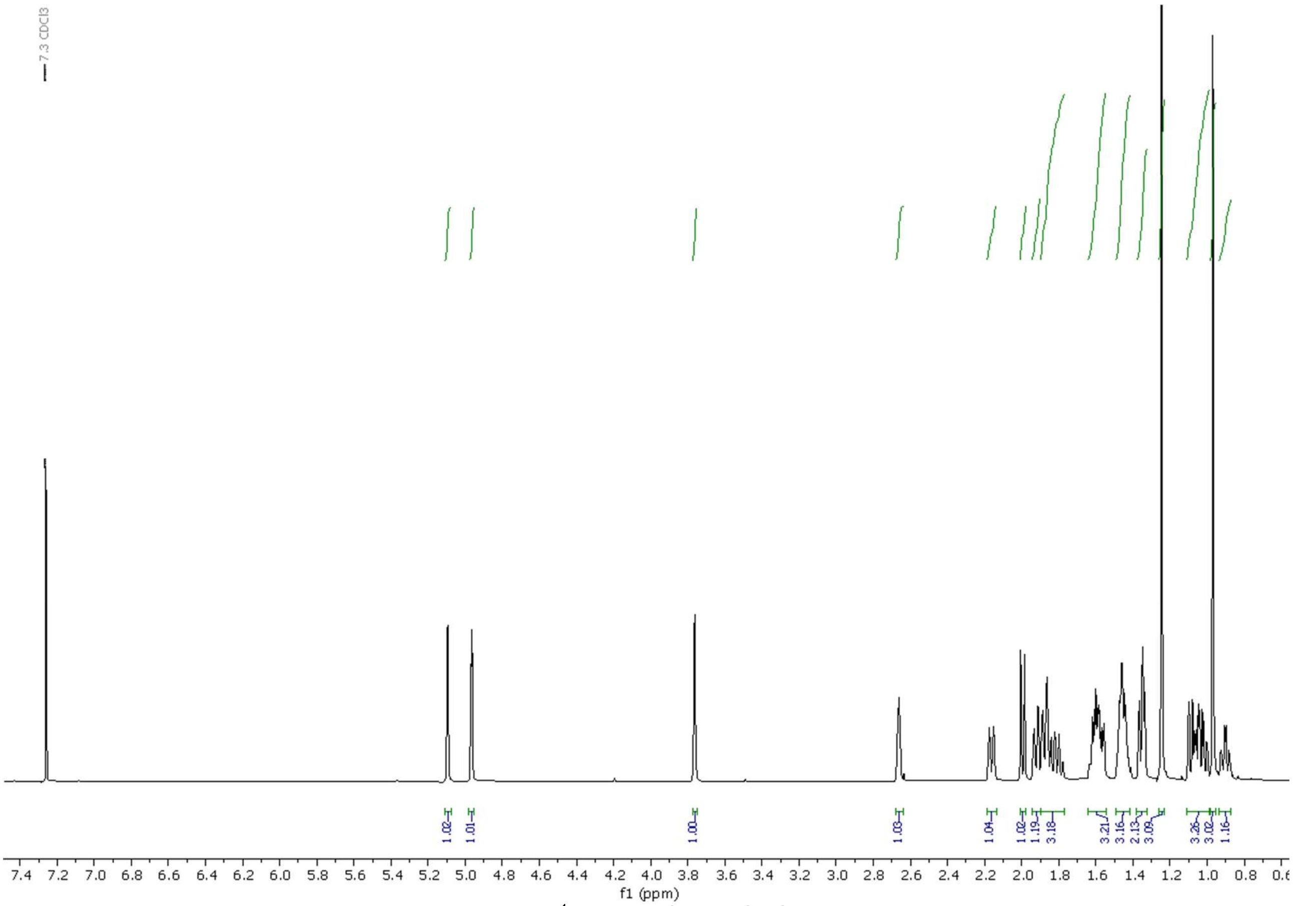

Figure N35.A ^{1}H NMR of **35** in $CDCl_3$ at 600 MHz.

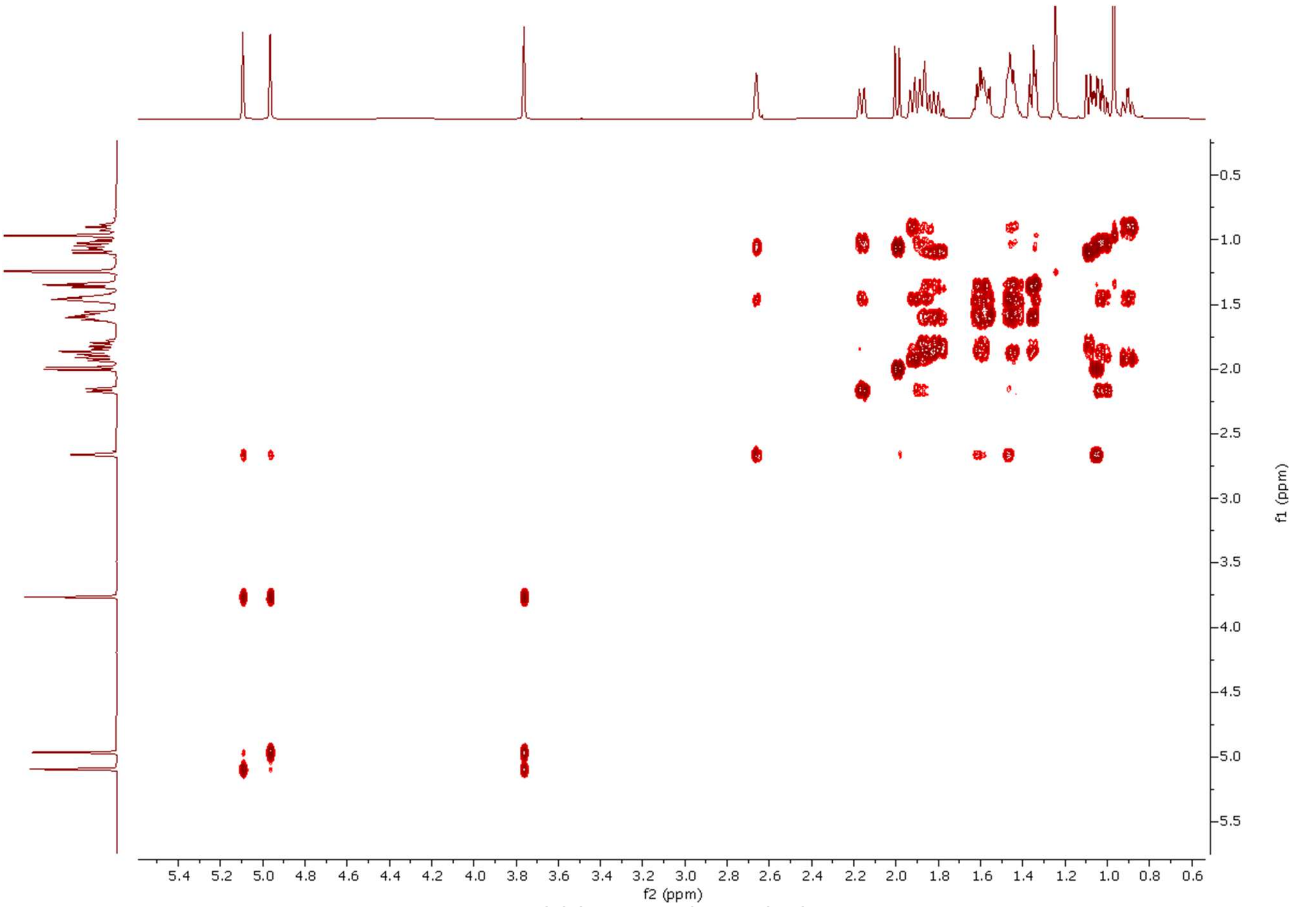

Figure N35.B COSY NMR of **35** in $CDCl_3$ at 600 MHz.

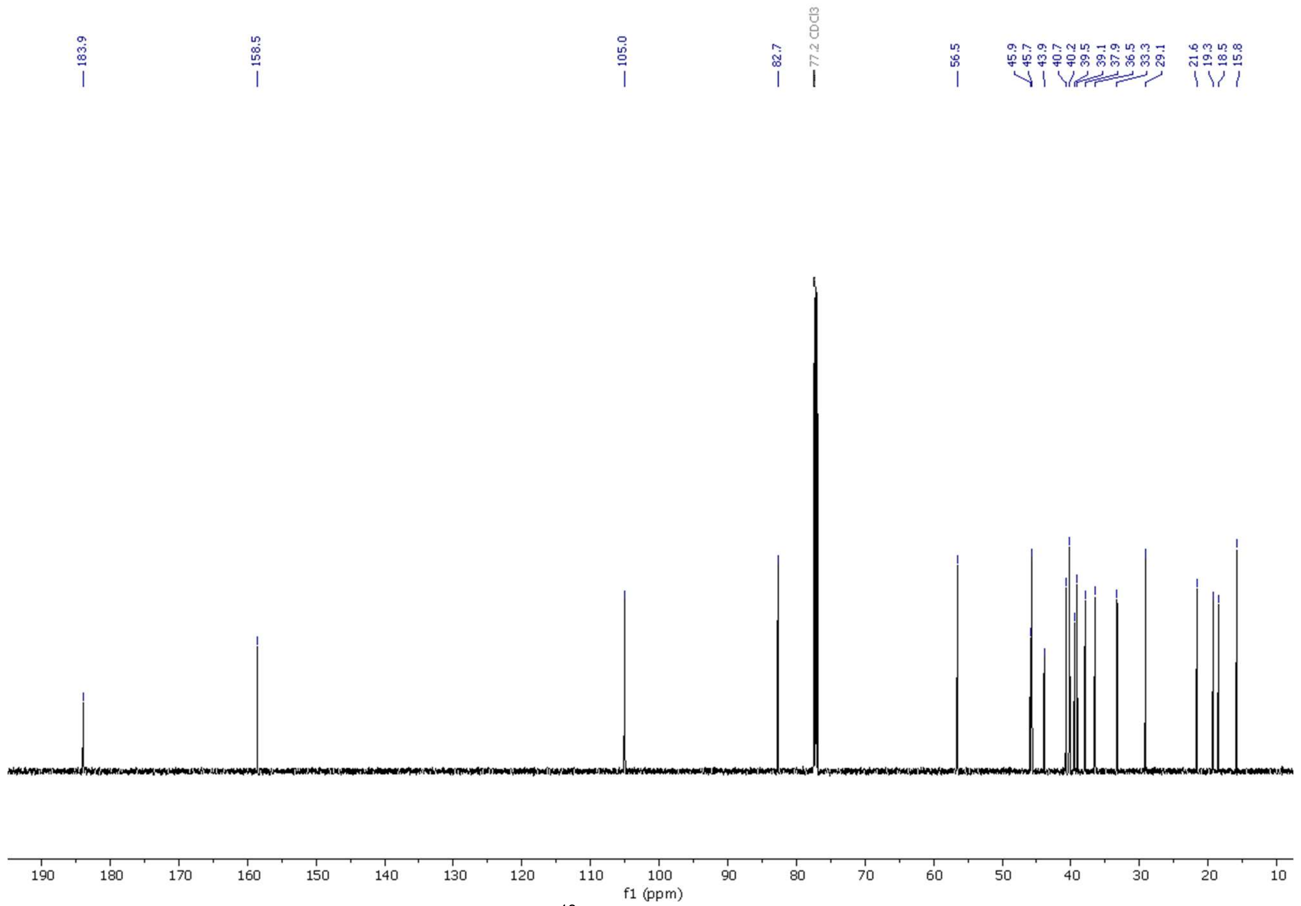

Figure N35.C ^{13}C NMR of **35** in $CDCl_3$ at 151 MHz.

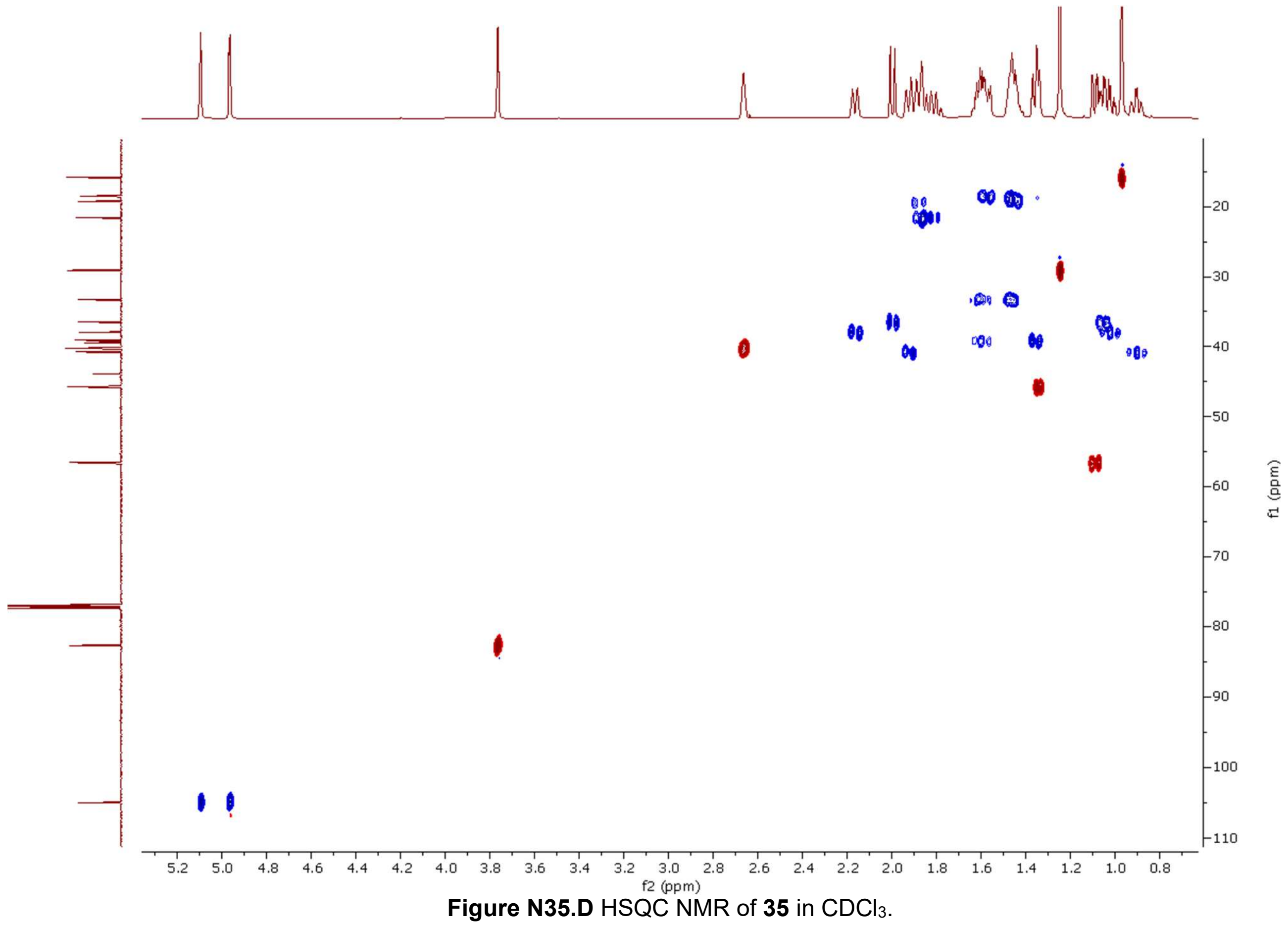

Figure N35.D HSQC NMR of **35** in $CDCl_3$.

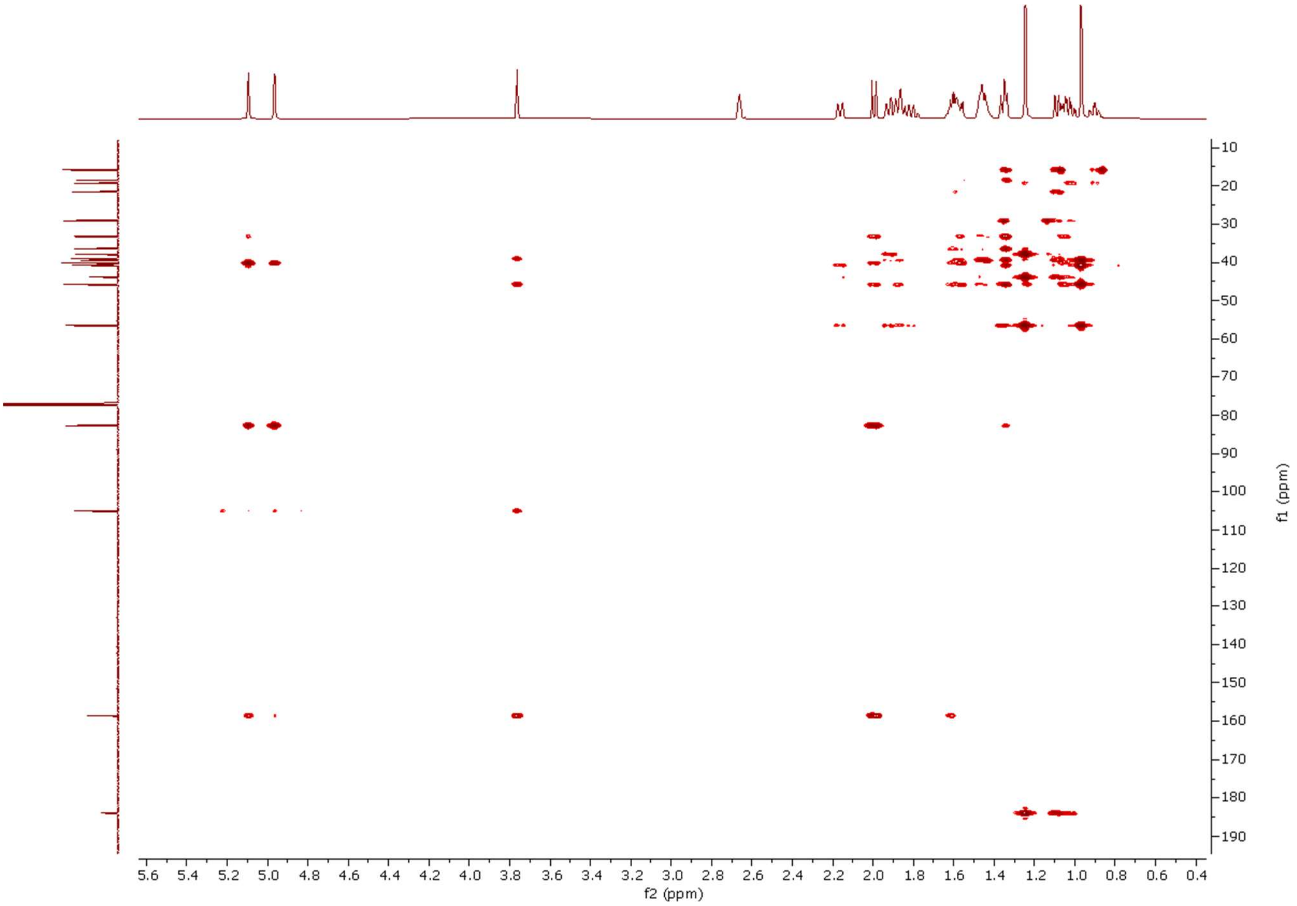

Figure N35.E HMBC NMR of **35** in $CDCl_3$.

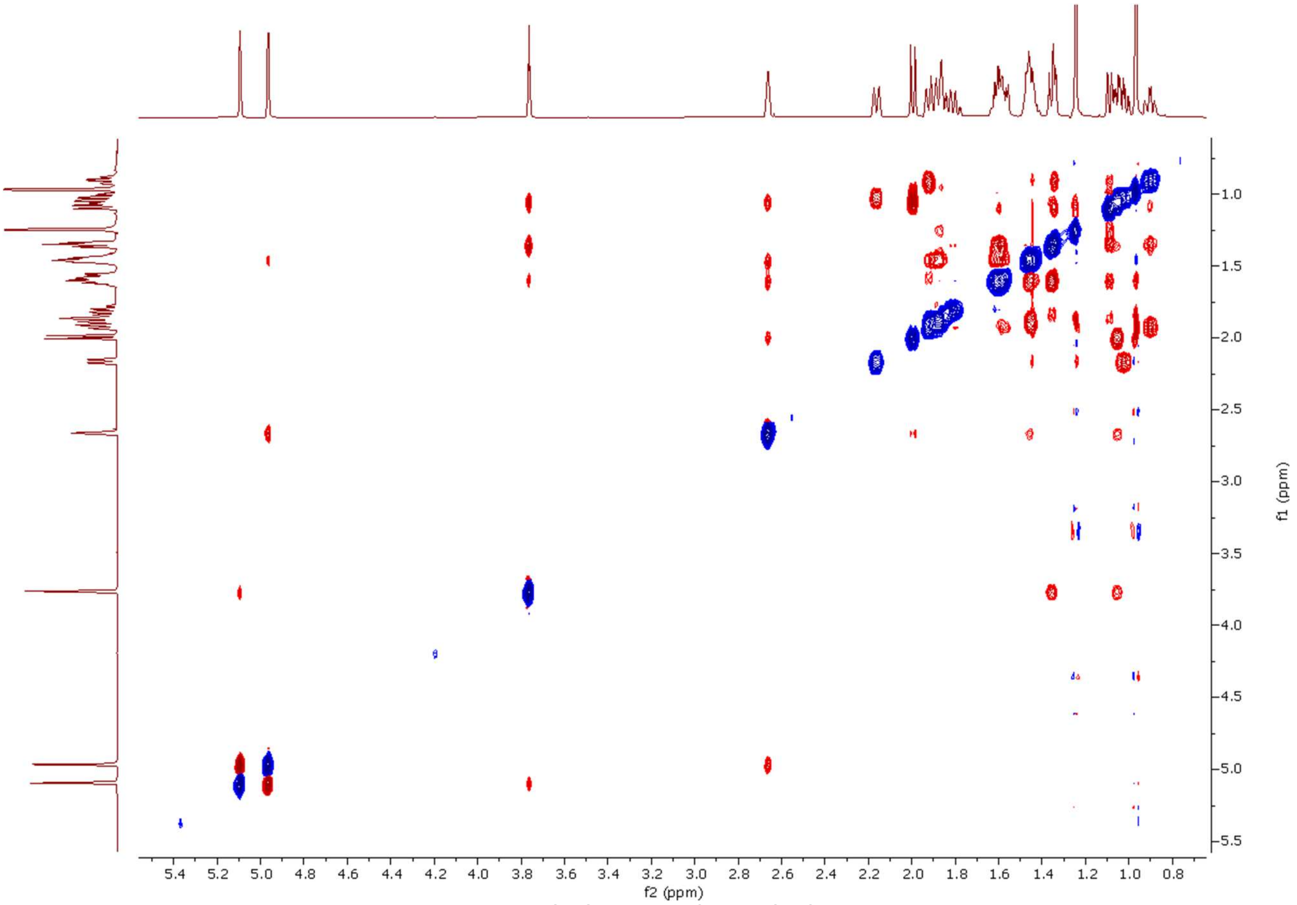

Figure N35.F NOESY NMR of **35** in $CDCl_3$ at 600 MHz.

7β,15β-Dihydroxy-*ent*-kaurenoic acid (36)

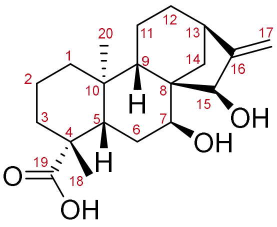

7β,15β-Dihydroxy-*ent*-kaurenoic acid (**36**)
Chemical Formula: $C_{20}H_{30}O_4$
Exact Mass: 334.2144

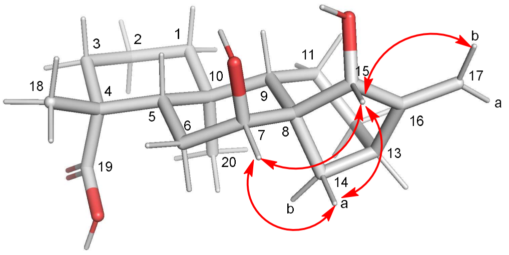

	Reference in C_5D_5N [47] (only δ_C reported)	Reference in C_5D_5N [24] (only δ_H reported)	Measured in C_5D_5N, 298K		
**Pos.**	δ_C 50.1 MHz	δ_H (*J*/Hz) 200 MHz	δ_C 151 MHz	δ_H (*J*/Hz) 400 MHz	**Selected NOESY correlations to H** [a]
**1**	41.0	n.r.	41.5	ax 1.07, overlapped<br>eq 1.99, ddd (13.1, 3.5, 3.5)	H-1eq<br>H-1ax
**2**	19.9	n.r.	20.3	a 1.55, overlapped<br>b 2.33, overlapped	
**3**	38.7	n.r.	39.2	ax 1.17, ddd (13.2, 13.2, 4.3)<br>eq 2.54, overlapped	H-3eq<br>H-3ax
**4**	43.5	-	43.9	-	
**5**	47.1	n.r.	47.5	2.24, overlapped	H_3-18
**6**	30.6	n.r.	31.1	a 2.39, overlapped<br>b 2.49, overlapped	H_3-18
**7**	78.0	4.54, s	78.4	eq 3.95, dd (2.9, 1.8)	H-14a, H-15, H-6a, H-6b
**8**	48.3	-	48.7	-	
**9**	42.1	n.r.	42.5	2.26, overlapped	
**10**	39.4	-	39.8	-	
**11**	18.0	n.r.	18.5	a 1.63, overlapped<br>b 2.06, m	H-11b<br>H-11a
**12**	34.4	n.r.	34.8	a 1.56, overlapped<br>b 1.66, overlapped	
**13**	41.0	n.r.	41.4	2.65, br s	H-15, H-17a, H-14b
**14**	35.4	n.r.	35.8	a 1.11, overlapped<br>b 1.88, d (11.8)	H-14b, H-7, H-15<br>H-14a, H_3-20, H-13
**15**	82.7	3.93, s	83.1	4.57, dd (2.7, 2.3)	H-14a, H-7, H-13, H-17b
**16**	157.8	-	158.3	-	
**17**	104.5	5.09, br s<br>5.45, br s	104.8	a 5.11, ddd (2.7, 1.2, 1.2)<br>b 5.48, ddd (2.3, 1.2,1.2)	H-13, H-17b<br>H-17a, H-15
**18**	29.2	1.37, s	29.6	1.39, s	H-5, H-6a
**19**	180.4	-	180.9	-	
**20**	16.1	1.23, s	16.5	1.26, s	H-14b

[a] Key NOESY correlations are shown in blue text.

n.r. = not reported.

Yellow highlighted data are possibly swapped assignments in the referenced data. Our assignments are supported by HMBC correlations.

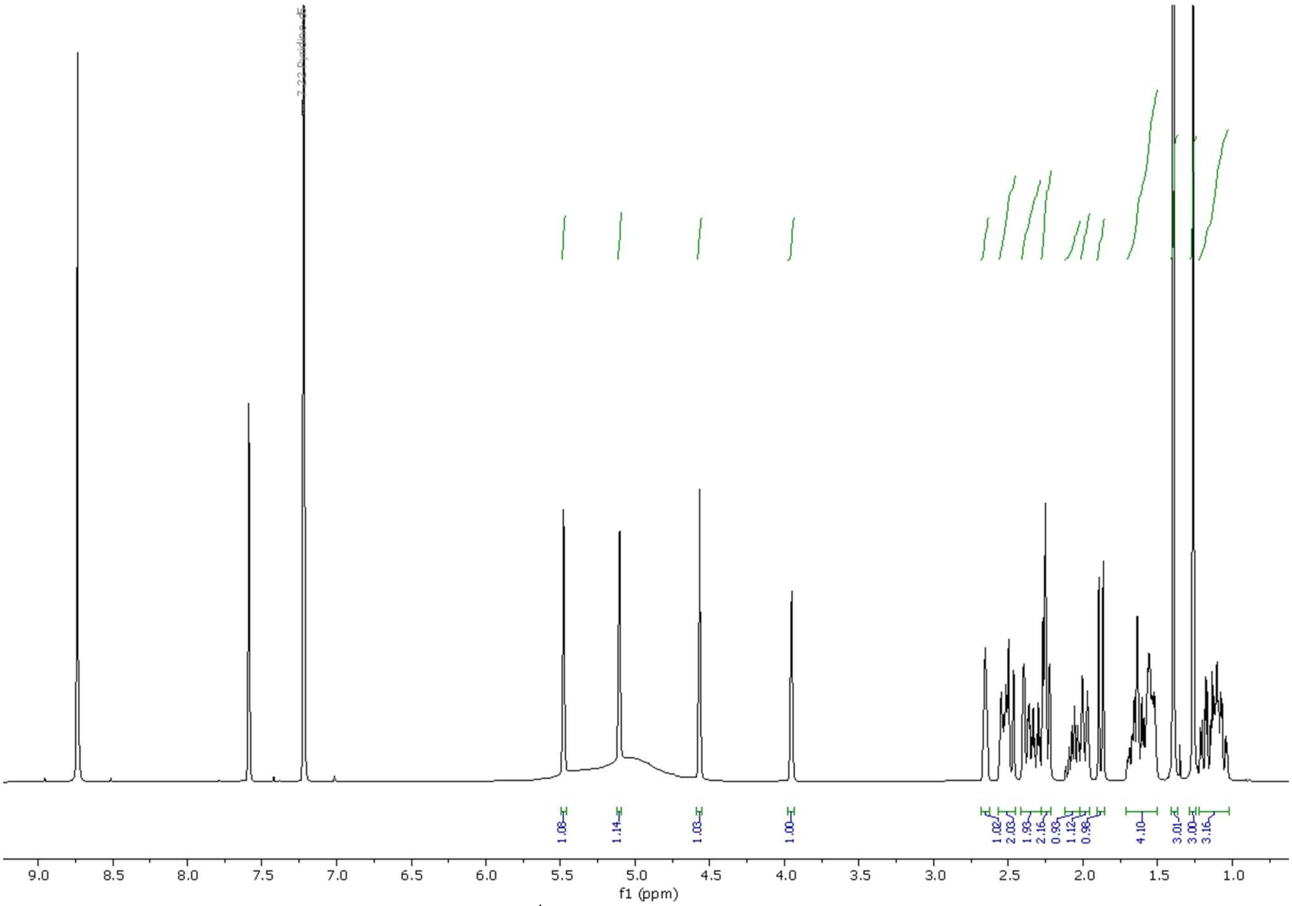

Figure N36.A ^{1}H NMR of **36** in pyridine-d_5 at 400 MHz.

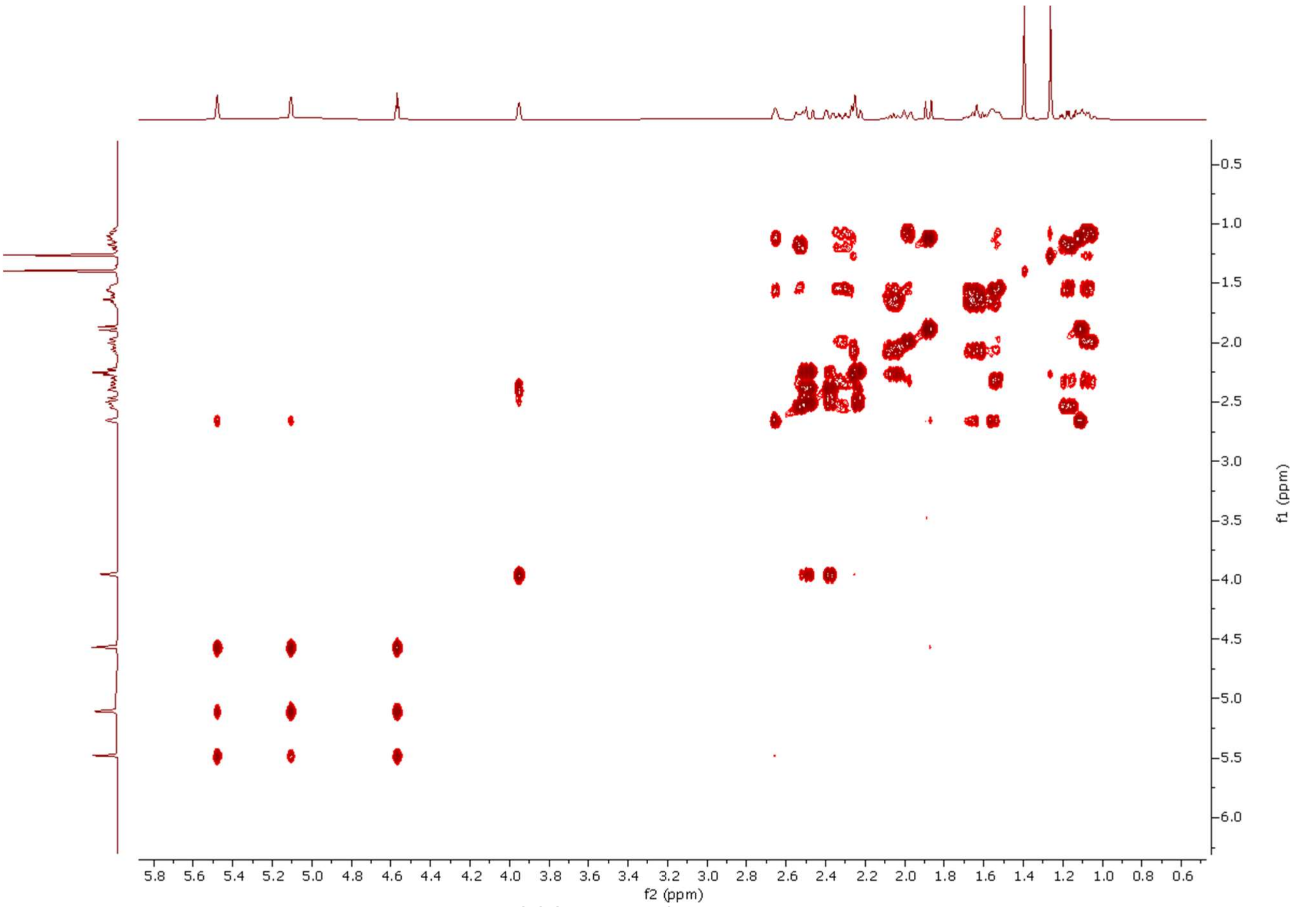

Figure N36.B COSY NMR of **36** in pyridine-d_5 at 600 MHz.

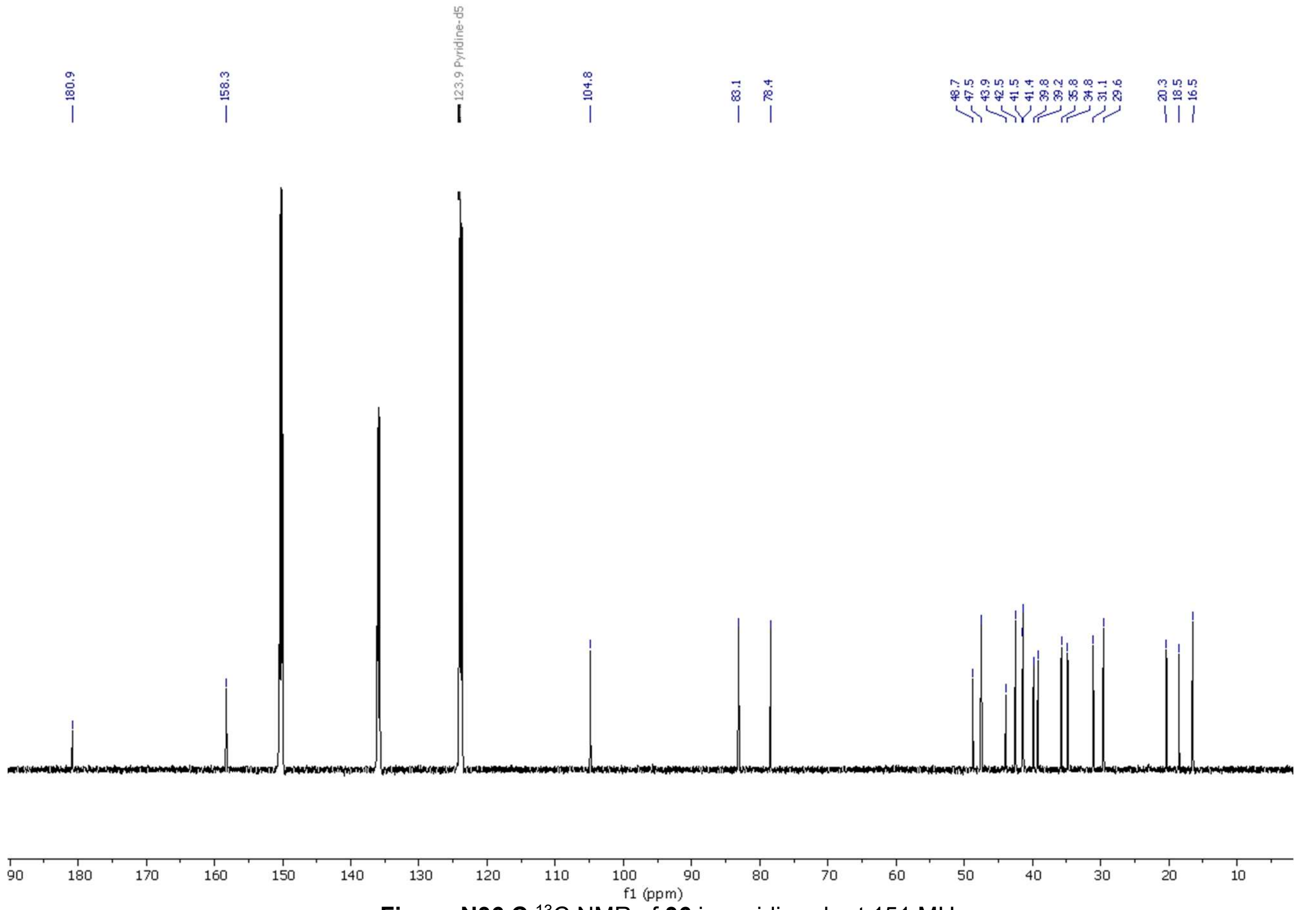

Figure N36.C ^{13}C NMR of **36** in pyridine-d_5 at 151 MHz.

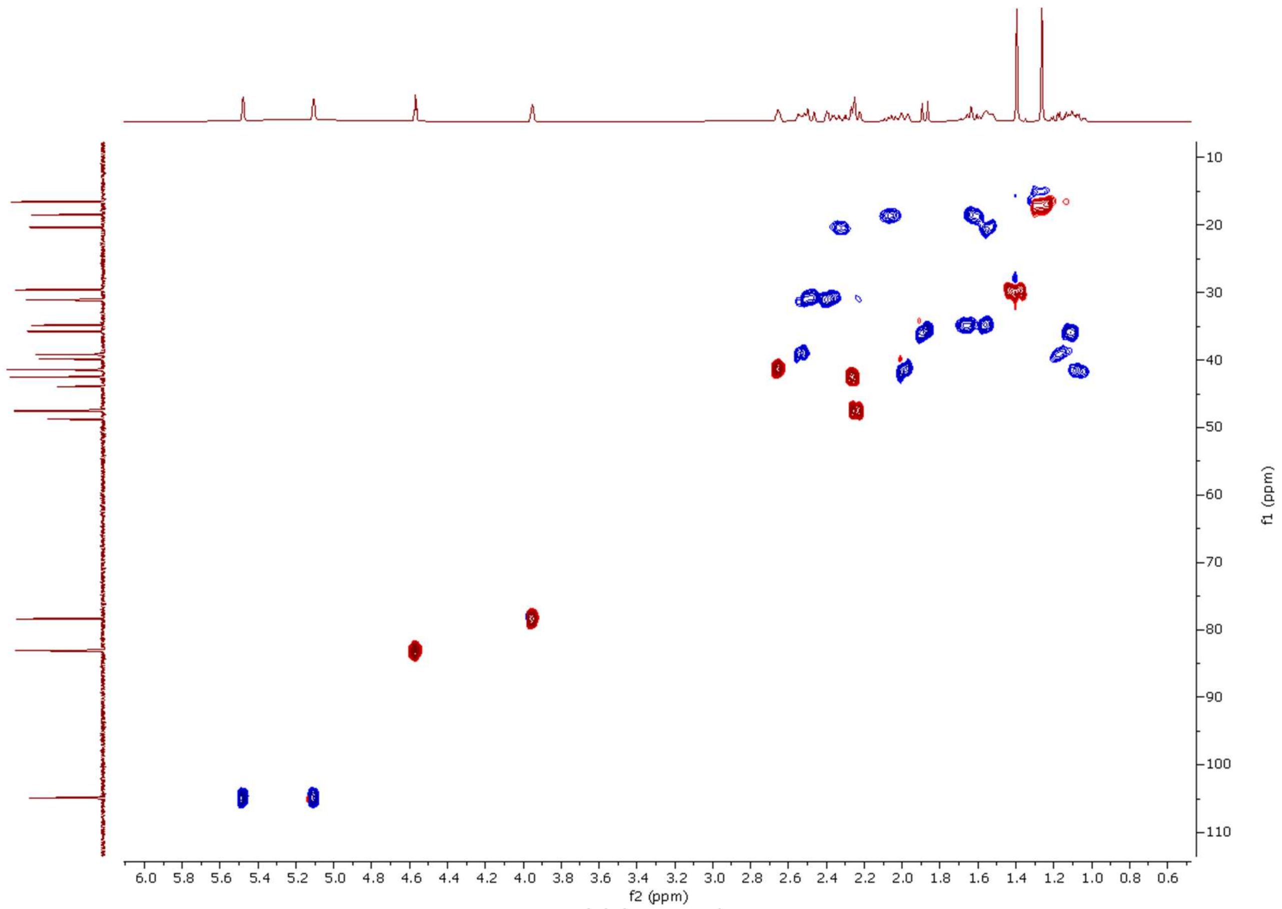

Figure N36.D HSQC NMR of **36** in pyridine-d_5.

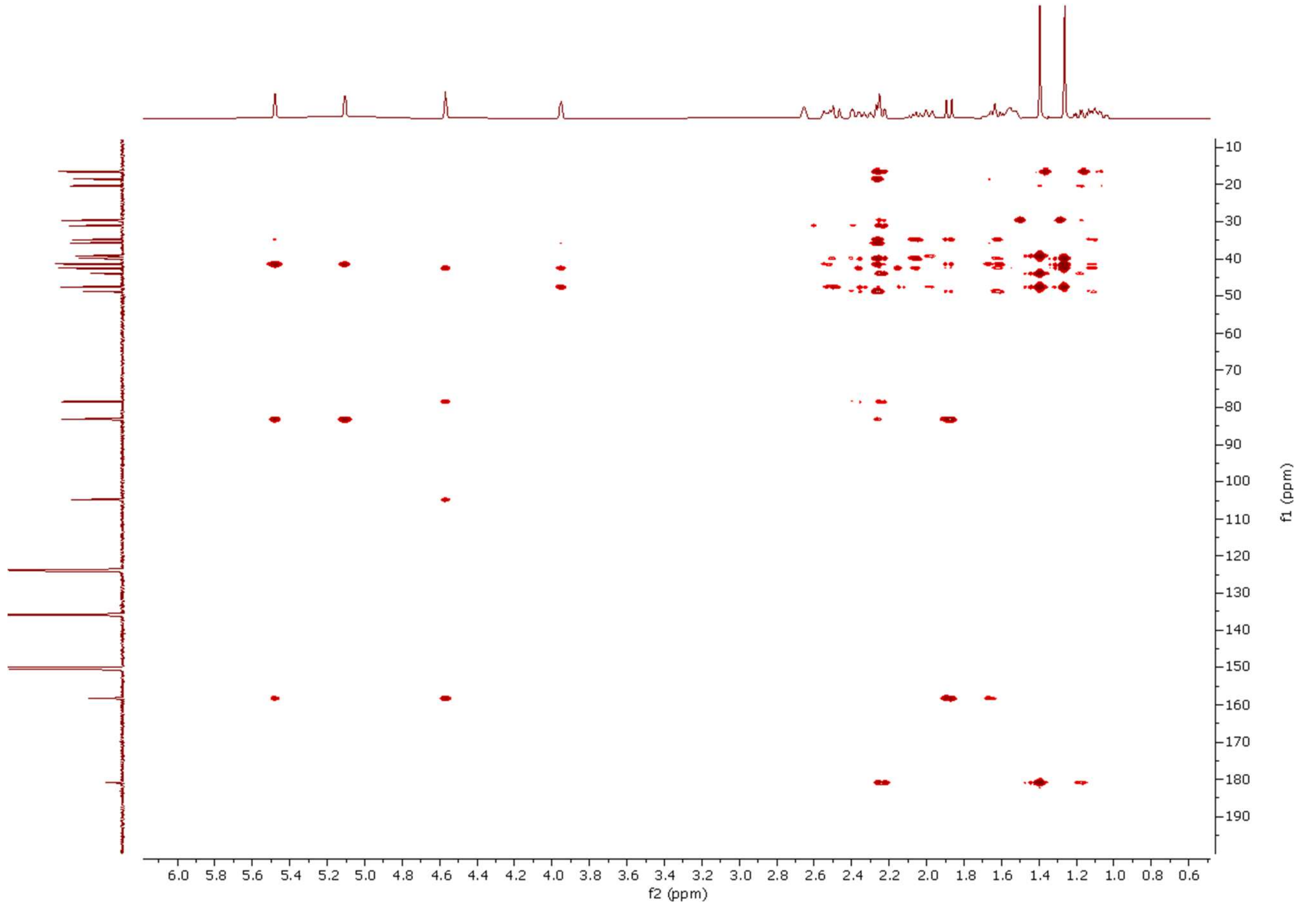

Figure N36.E HMBC NMR of **36** in pyridine-d$_5$.

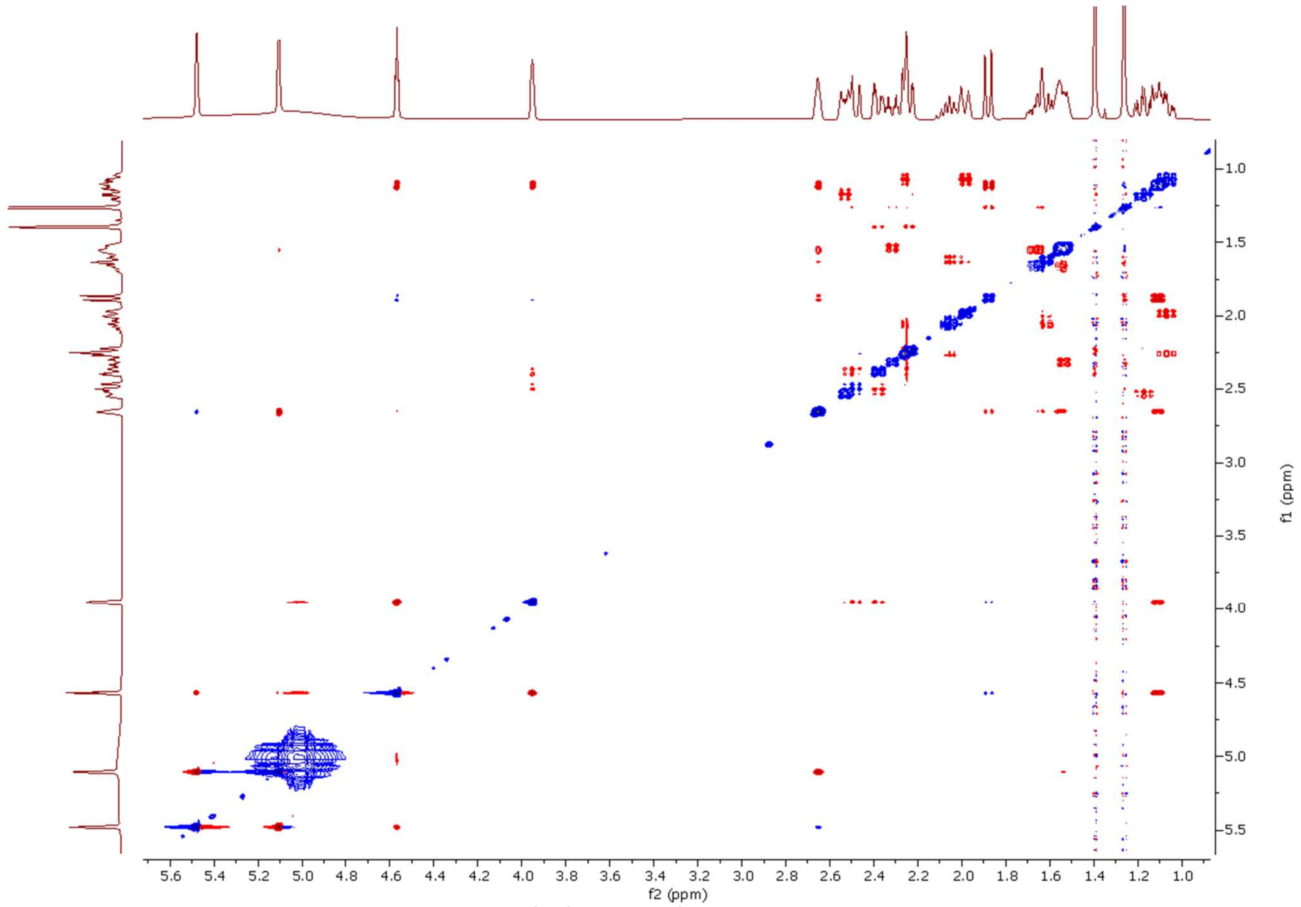

Figure N36.F NOESY NMR of **36** in pyridine-d$_5$ at 400 MHz.

7β,15β-Dihydroxy-*ent*-kaurenolide (42)

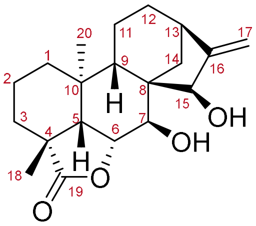

7β,15β-Dihydroxy-*ent*-kaurenolide (**42**)
Chemical Formula: $C_{20}H_{28}O_4$
Exact Mass: 332.1988

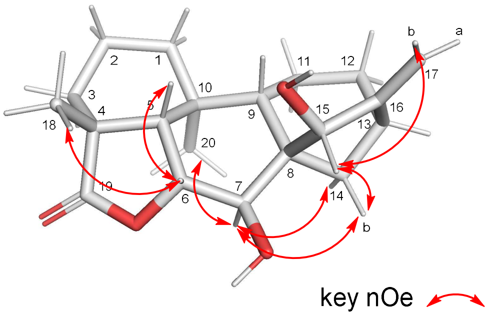

	**Reference in CD_3COCD_3 [24] (only δ_H reported)**	**Measured in d_4-methanol, 298K**		
**Pos.**	δ_H (*J*/Hz) 200 MHz	δ_C 151 MHz	δ_H (*J*/Hz) 600 MHz	**Selected NOESY correlations to H [a]**
**1**	n.r.	38.3	a 1.15, m<br>b 1.58, overlapped	H-1b<br>H-1a
**2**	n.r.	18.5	a 1.53, overlapped<br>b 1.56, overlapped	 H_3-20
**3**	n.r.	29.2	a 1.41, overlapped<br>b 2.03, m	H-3b<br>H_3-18, H-3a
**4**	-	43.1	-	-
**5**	2.20, d (6.0)	52.2	2.16, d (6.4)	H-6, H_3-18, H-9
**6**	4.75, t (6.0)	86.0	4.79, dd (6.4, 6.4)	H-5, H_3-18
**7**	4.31, d (6.0)	72.1	4.30, d (6.4)	H_3-20, H-14b, H-14a
**8**	-	49.3	-	-
**9**	n.r.	46.6	1.59, overlapped	H-1a, H-5
**10**	-	35.0	-	-
**11**	n.r.	17.7	a 1.38, overlapped<br>b 1.47, overlapped	
**12**	n.r.	34.6	a 1.43, overlapped<br>b 2.15, m	H-17a<br>H-13
**13**	2.56, m	37.4	2.57, m	H-17a, H-12b, H-14b
**14**	n.r.	30.2	a 1.46, overlapped<br>b 1.60, overlapped	H-7<br>H-7, H-15, H-13
**15**	3.67, br t	74.3	4.58, dd (2.9, 2.7)	H-14b, H-17b
**16**	-	163.0	-	-
**17**	5.0, br s	108.1	b 5.04, ddd (2.7, 1.3, 0.8)<br>a 5.05, ddd (2.9, 1.3, 0.8)	H-15<br>H-12a, H-13
**18**	1.20, s	25.8	1.26, s	H-6, H-5, H-3b
**19**	-	185.6	-	-
**20**	0.86, s	21.5	0.89, s	H-7, H-2b

[a] Key NOESY correlations are shown in blue text.

n.r. = not reported.

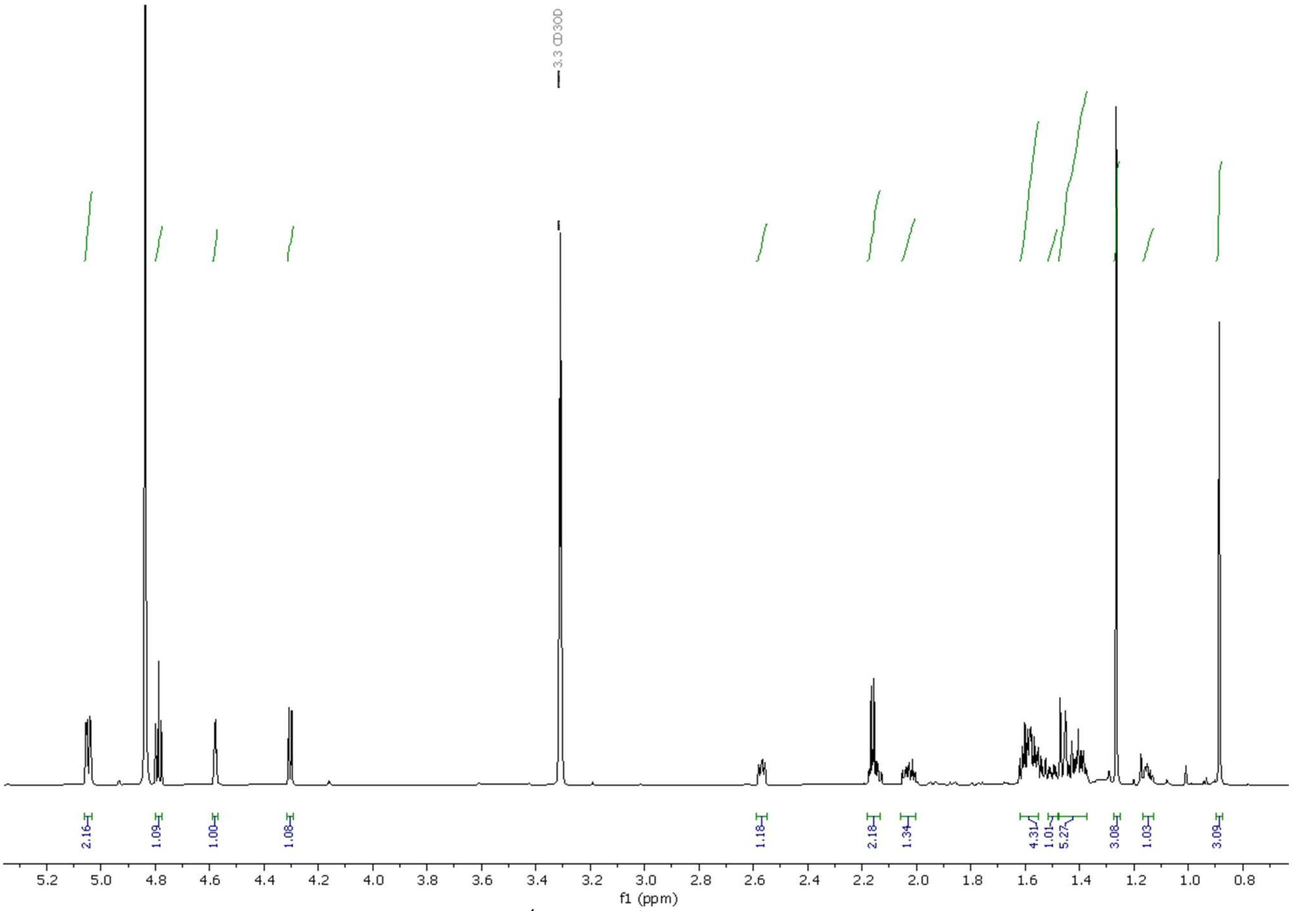

Figure N42.A ^{1}H NMR of **42** in d_4-methanol at 600 MHz.

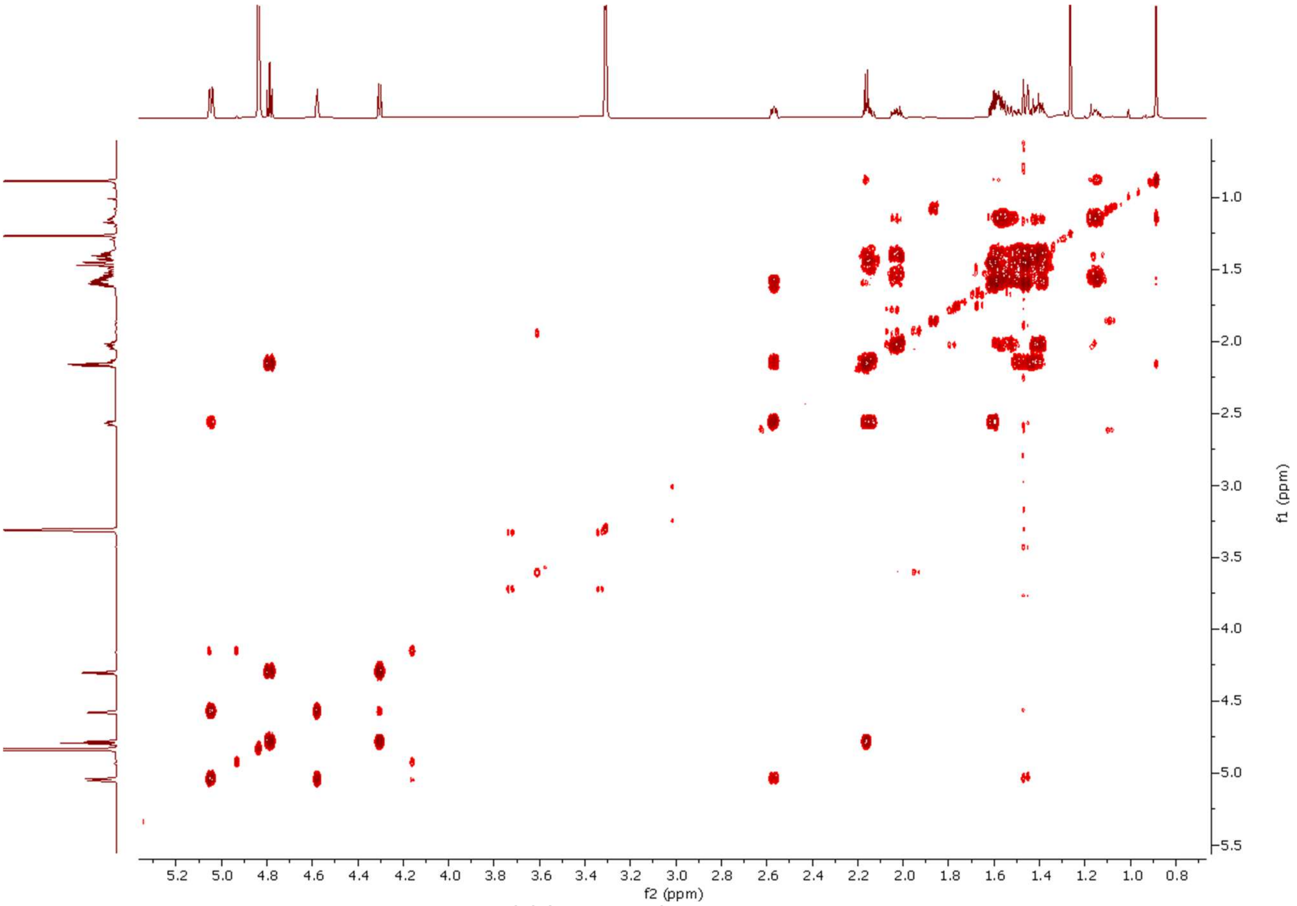

Figure N42.B COSY NMR of **42** in d_4-methanol at 600 MHz.

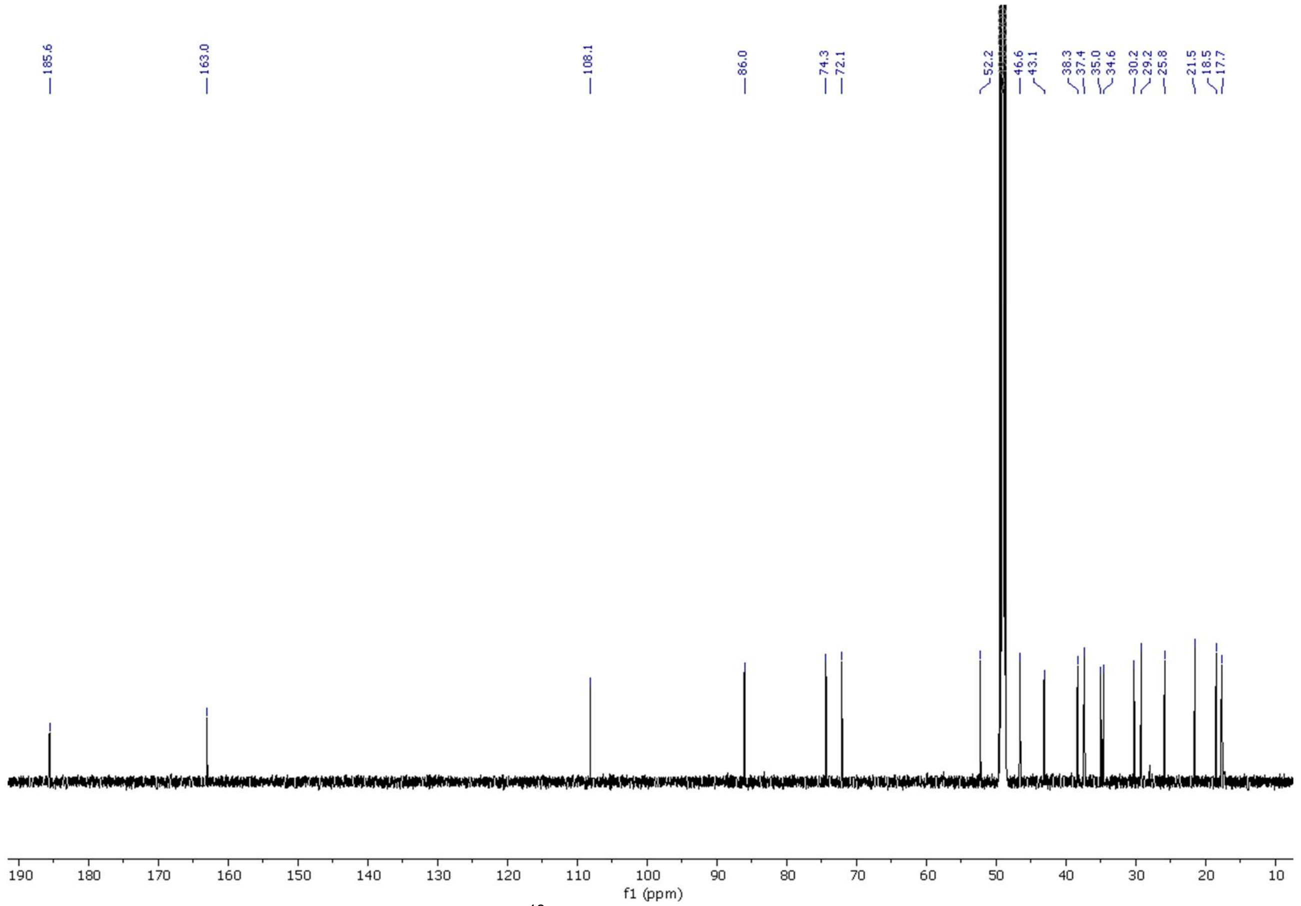

Figure N42.C ^{13}C NMR of **42** in d_4-methanol at 151 MHz.

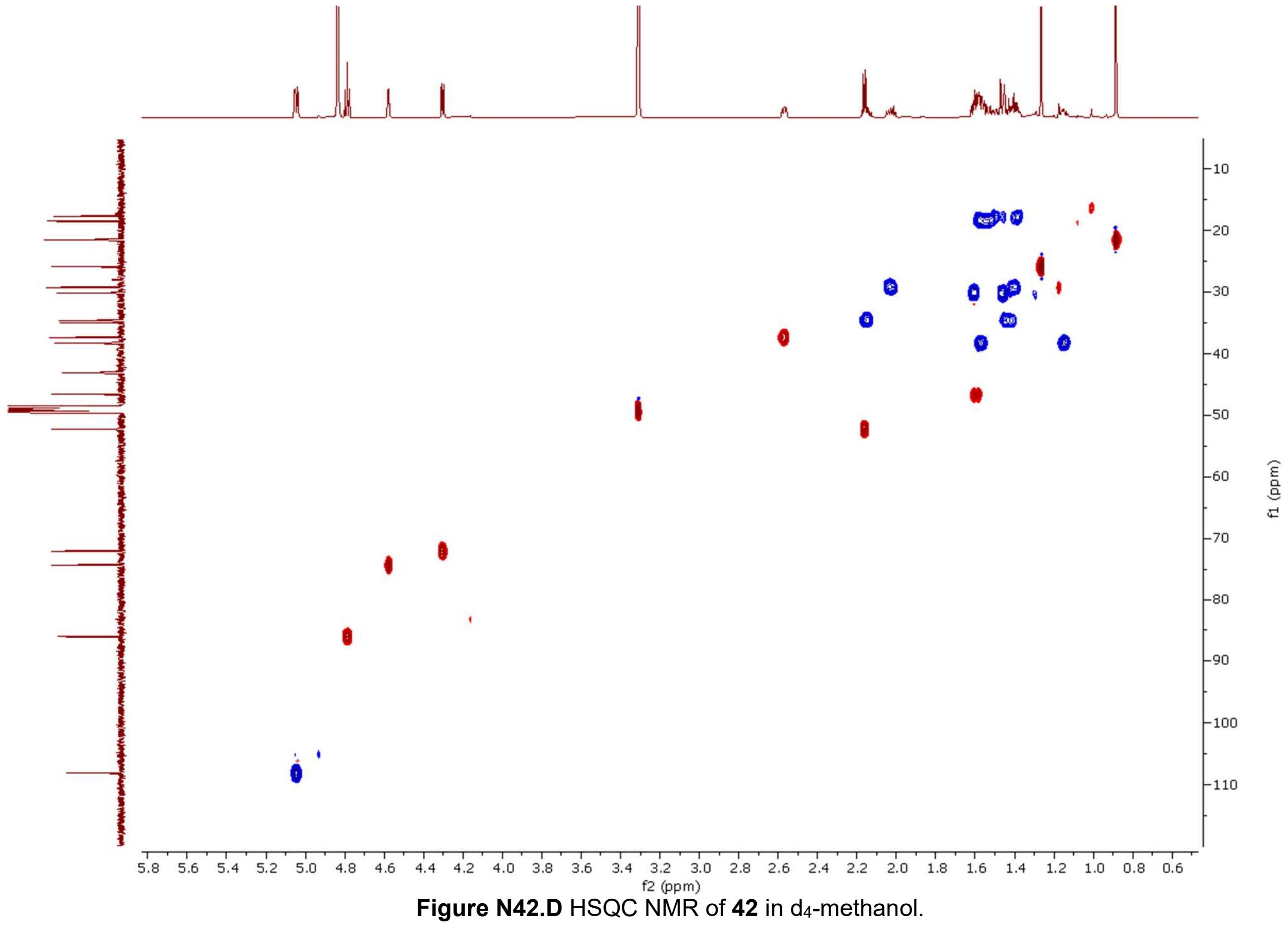

Figure N42.D HSQC NMR of **42** in d_4-methanol.

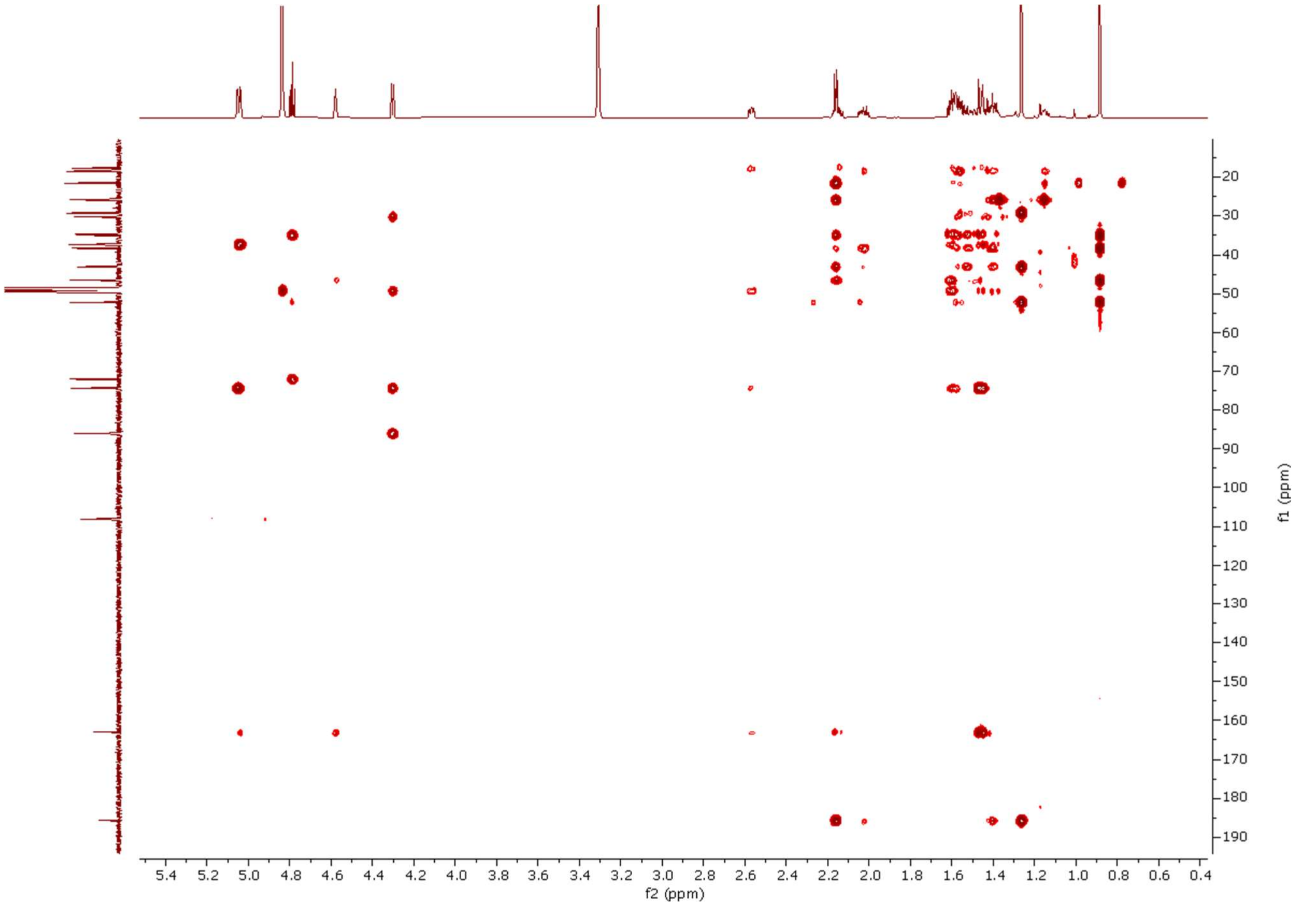

Figure N42.E HMBC NMR of **42** in d4-methanol.

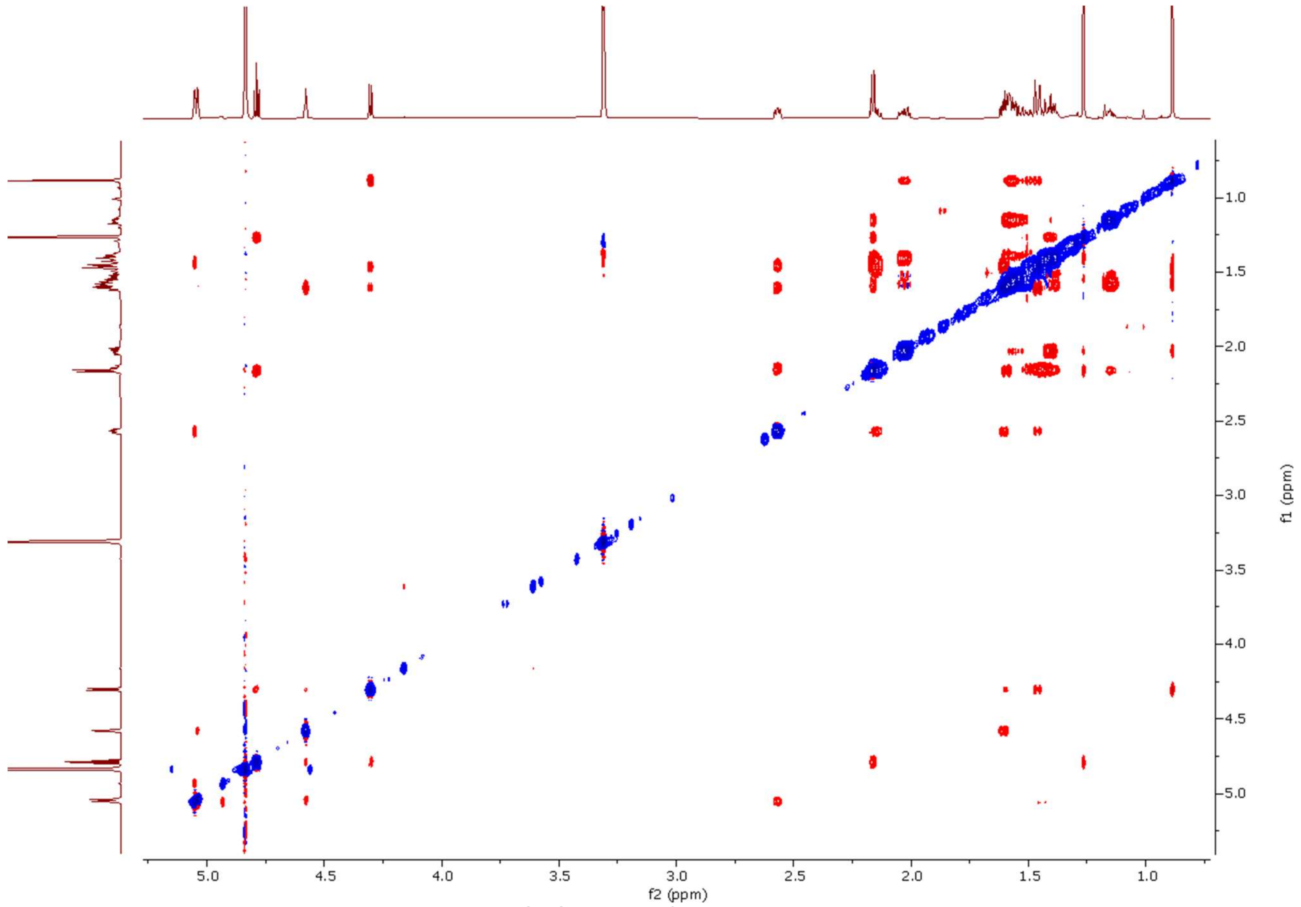

Figure N42.F NOESY NMR of **42** in d4-methanol at 600 MHz.

1β,7β,15β-Trihydroxy-*ent*-kaurenoic acid (43)

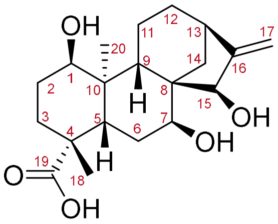

1β,7β,15β-Trihydroxy-*ent*-kaurenoic acid (**43**)
Chemical Formula: $C_{20}H_{30}O_5$
Exact Mass: 350.2093

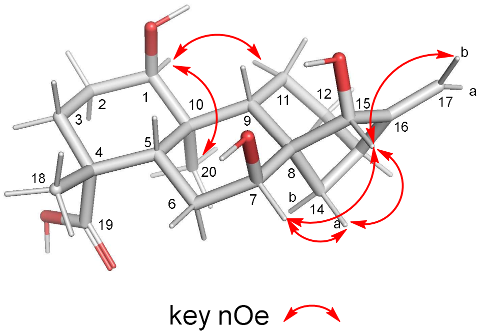

Measured in d_4-methanol, 298K			
**Pos.**	δ_C 151 MHz	δ_H (*J/Hz*) 600 MHz	**Selected NOESY correlations to H** [a]
**1**	71.6	eq 3.74, br s	H-11a, H-2a, H_3-20, H-2b, H-9
**2**	27.8	a 1.52, overlapped<br>b 2.29, m	H-1eq, H-2b<br>H-1eq, H-2a
**3**	32.2	a 1.51, overlapped<br>b 1.90, overlapped	H-5
**4**	44.4	-	-
**5**	40.7	2.20, br d (12.5)	H_3-18, H-3a, H-6a, H-9
**6**	30.4	a 1.96, ddd (14.2, 3.0, 3.0)<br>b 2.09, dd (14.2, 12.5)	H-7, H-5, H_3-18<br>H-7
**7**	78.9	3.58, br s	H-15, H-14a, H-6b, H-6a
**8**	48.9	-	-
**9**	32.7	2.60, br d (7.7)	H-5, H-11b
**10**	44.1	-	-
**11**	17.8	a 1.50, overlapped<br>b 1.65, m	H-1eq<br>H-9
**12**	34.9	a 1.50, overlapped<br>b 1.69, m	H-13<br>H-13
**13**	42.1	2.63, br s	H-15, H-14a, H-12a, H-12b, H-14b, H-17a
**14**	36.0	a 1.09, dd (11.8, 5.0)<br>b 1.88, d (11.8)	H-7, H-15, H-13<br>H-13, H_3-20
**15**	83.6	4.17, dd (2.7, 2.5)	H-14a, H-13, H-7, H-17b
**16**	157.8	-	-
**17**	105.0	a 4.93, br d (2.7)<br>b 5.06, br s	H-13<br>H-15
**18**	29.1	1.18, s	H-5, H-6a
**19**	182.8 [HMBC]	-	-
**20**	17.1	1.01, s	H-1eq, H-14b

[a] Key NOESY correlations are shown in blue text.

[HMBC] detected based on HMBC.

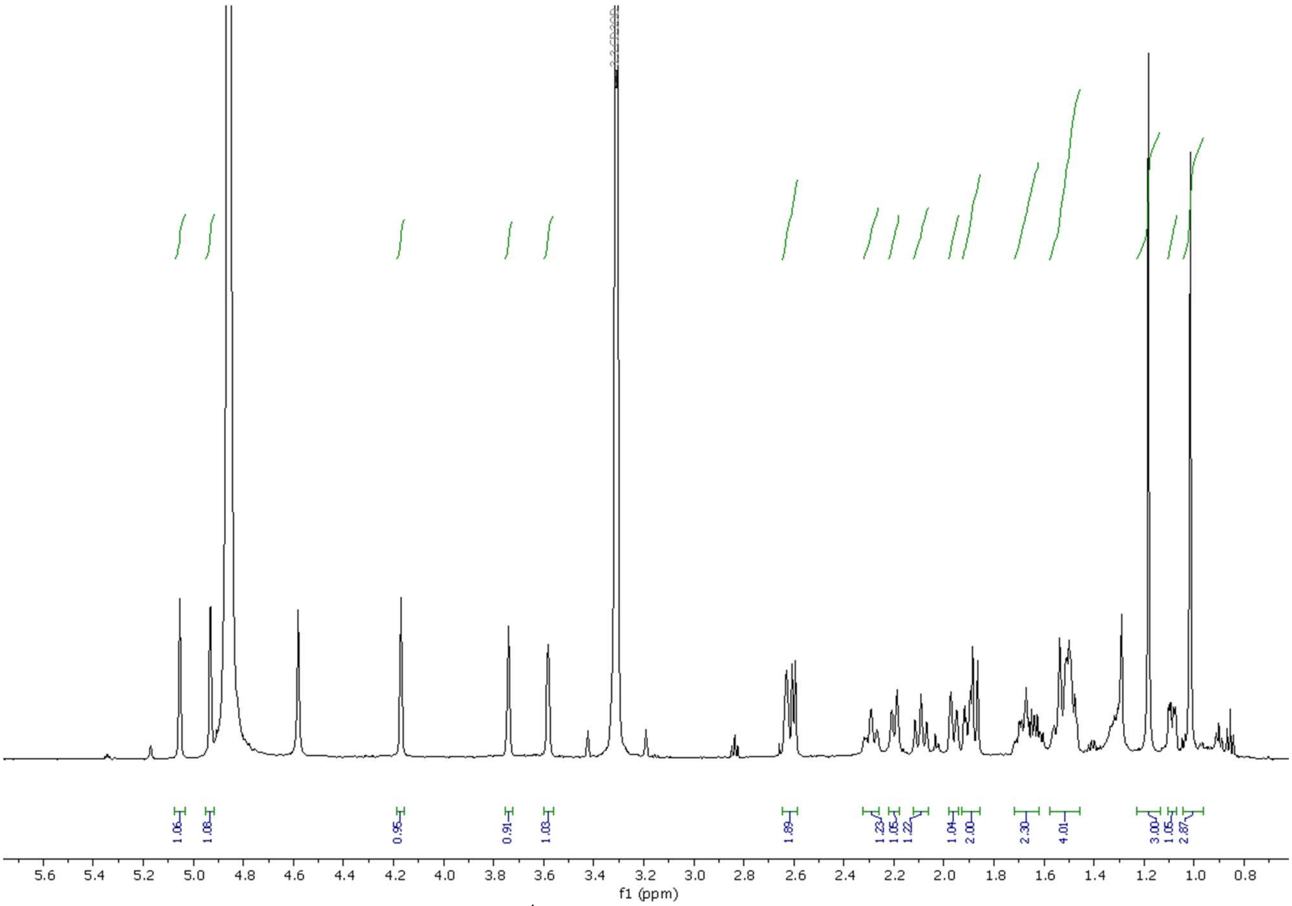

Figure N43.A ^{1}H NMR of **43** in d$_4$-methanol at 600 MHz.

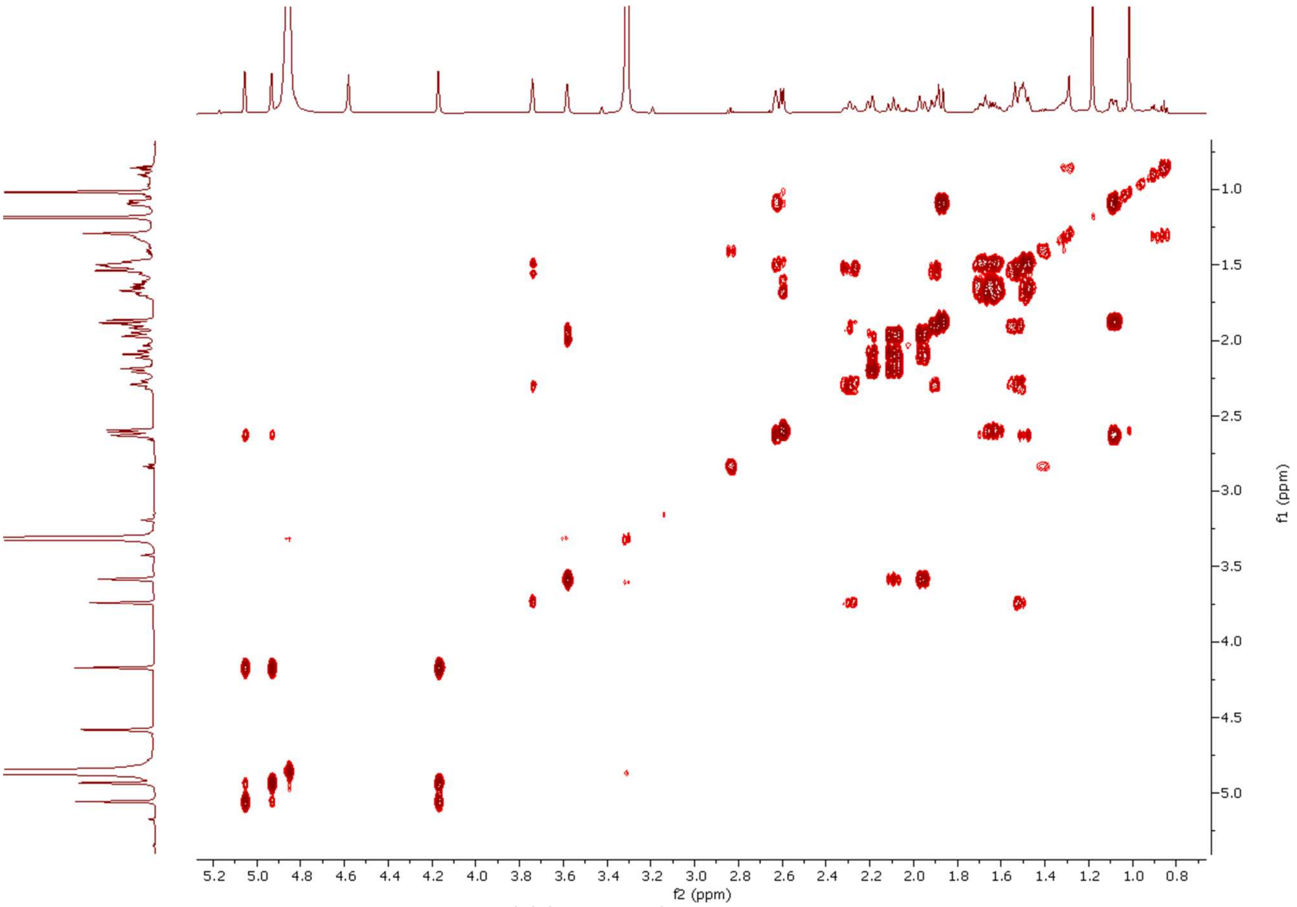

Figure N43.B COSY NMR of **43** in d$_4$-methanol at 600 MHz.

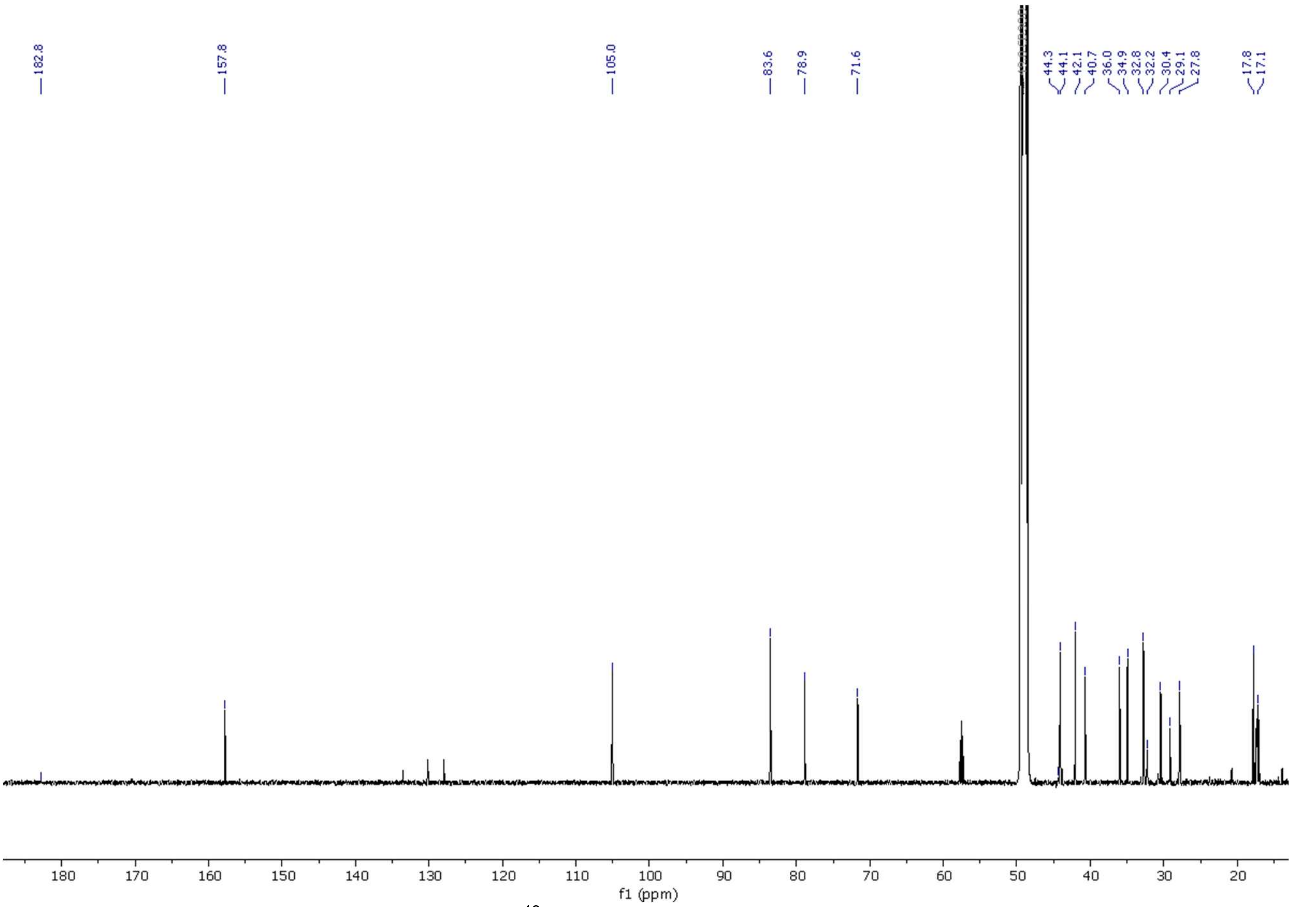

Figure N43.C ^{13}C NMR of **43** in d_4-methanol at 151 MHz.

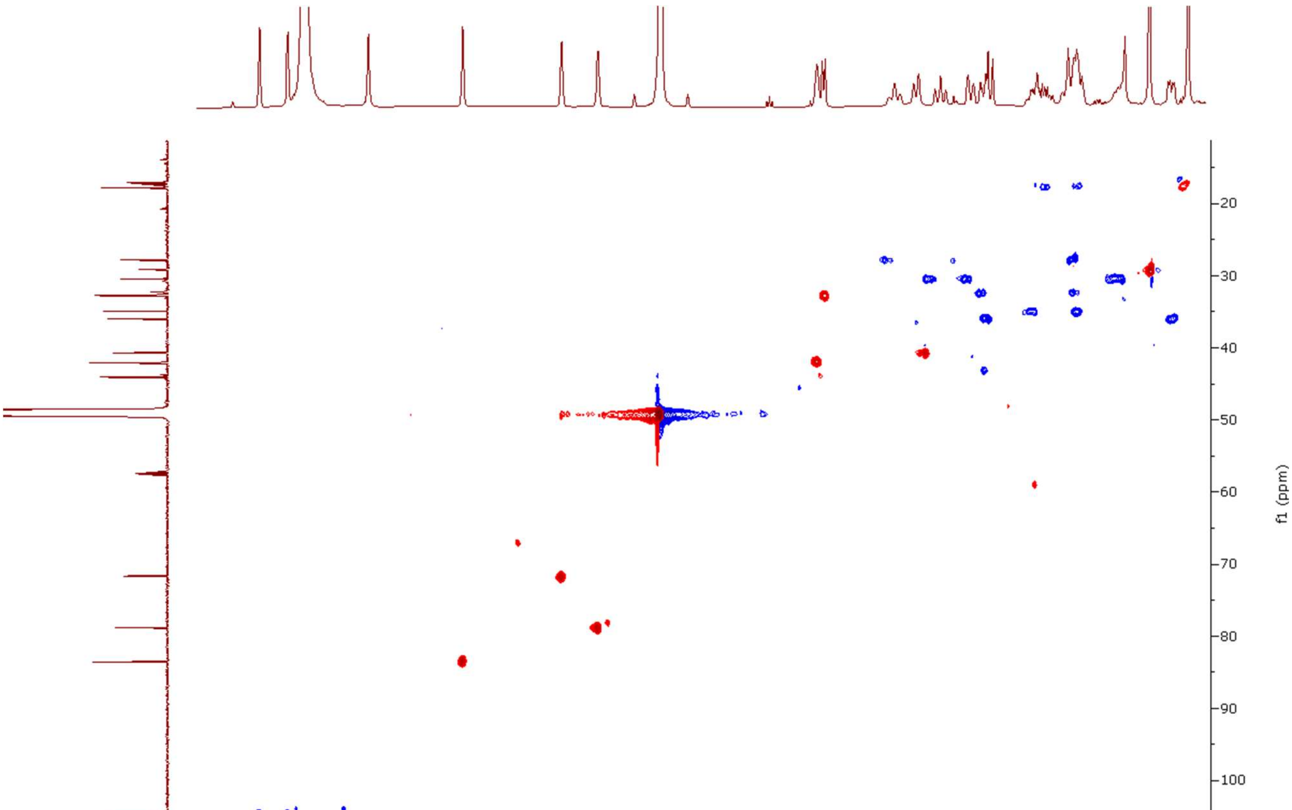

Figure N43.D HSQC NMR of **43** in d_4-methanol.

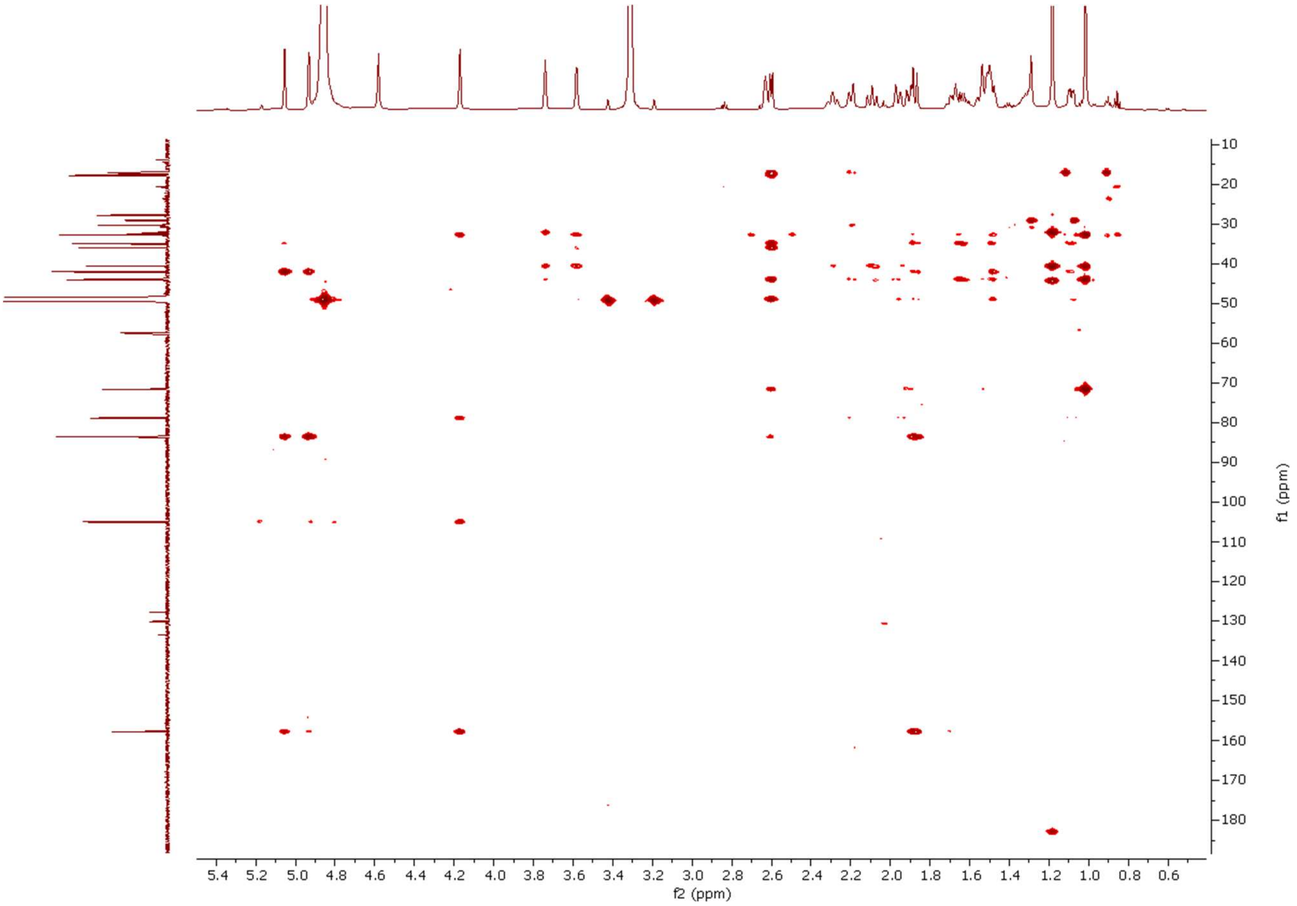

Figure N43.E HMBC NMR of **43** in d$_4$-methanol.

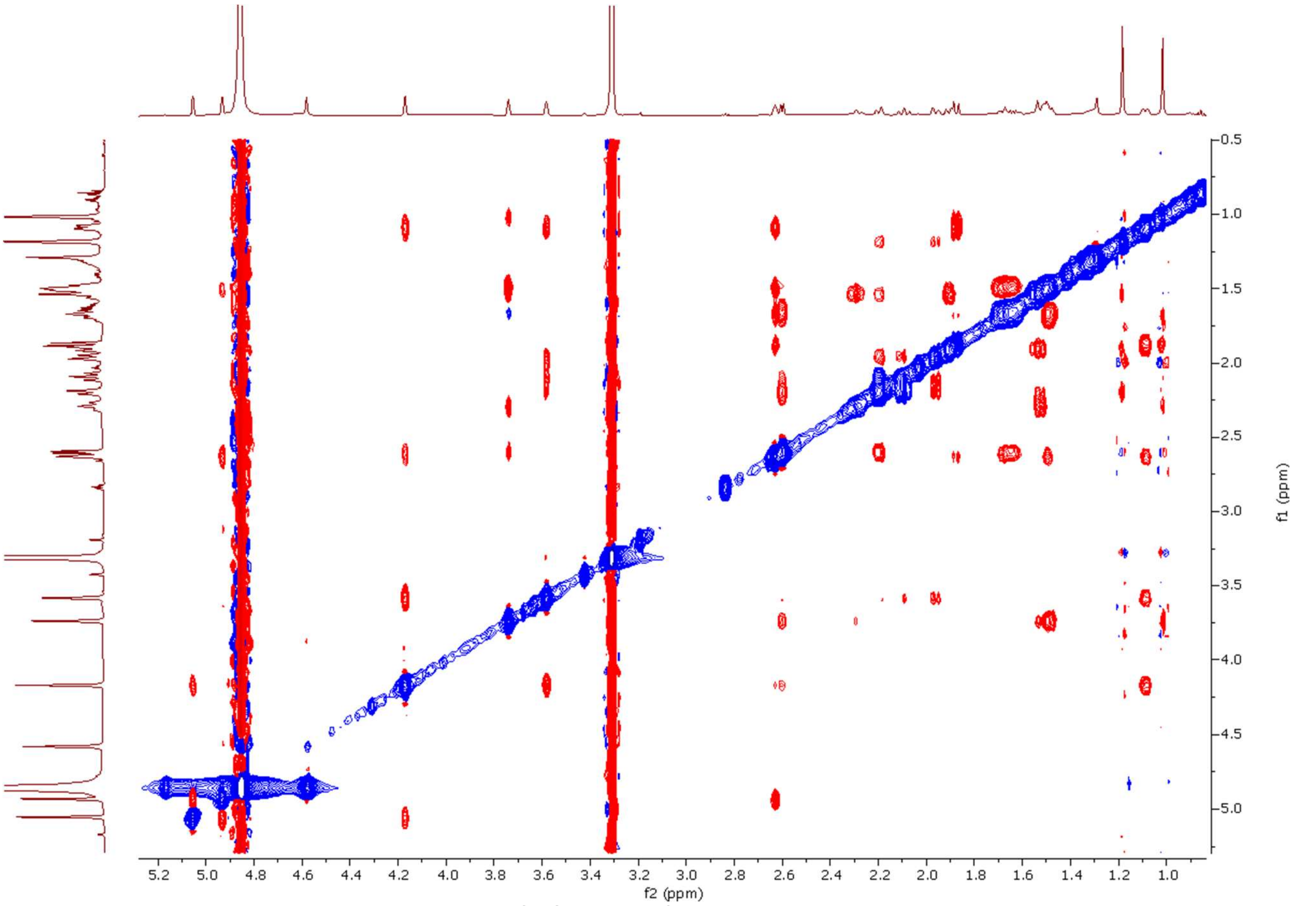

Figure N43.F NOESY NMR of **43** in d$_4$-methanol at 600 MHz.

3β,7β,15β-Trihydroxy-*ent*-kaurenoic acid (44)

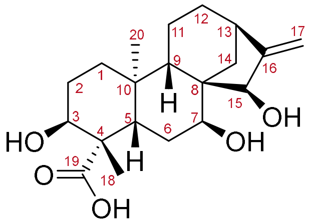

3β,7β,15β-Trihydroxy-*ent*-kaurenoic acid (**44**)
Chemical Formula: $C_{20}H_{30}O_5$
Exact Mass: 350.2093

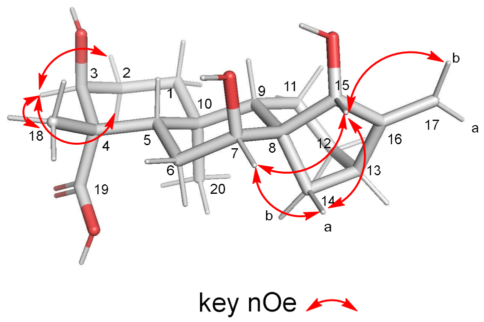

Measured in d_4-methanol, 298K			
**Pos.**	δ_C 151 MHz	δ_H (*J/Hz*) 600 MHz	**Selected NOESY correlations to H** [a]
**1**	35.2	ax 1.40, ddd (13.5, 13.5, 4.0)<br>eq 1.64, ddd (13.2, 4.3, 4.3)	H-1eq, H-9, H-5<br>H-1ax, H_3-20
**2**	27.3	a 1.58, ddd (14.5, 6.5, 2.9)<br>b 2.20, m	H-3<br>H-3, H_3-20
**3**	71.8	eq 4.02, dd (2.9, 2.9)	H_3-18, H-2a, H-2b
**4**	48.3	-	-
**5**	40.3	2.25, dd (13.2, 2.2)	H-1ax, H_3-18, H-9
**6**	30.3	eq 1.82, ddd (14.5, 3.8, 2.2)<br>ax 2.07, ddd (14.5, 13.2, 2.2)	H-7, H_3-18<br>H-7, H_3-20
**7**	79.1	3.61, dd (3.8, 2.2)	H-14a, H-6ax, H-6eq, H-15
**8**	49.1	-	-
**9**	42.7	1.83, overlapped	H-1ax, H-5
**10**	40.0	-	-
**11**	18.6	a 1.54, m<br>b 1.68, overlapped	
**12**	35.0	a 1.48, m<br>b 1.68, overlapped	H-13<br>H-13
**13**	42.0	2.62, m	H-17a, H-14a, H-12a, H-12b, H-14b
**14**	36.0	a 1.09, dd (11.8, 4.9)<br>b 1.87, d (11.8)	H-7, H-15, H-13<br>H_3-20, H-13
**15**	83.3	4.16, dd (2.9, 2.4)	H-14a, H-17b, H-7
**16**	157.7	-	-
**17**	105.1	a 4.93, ddd (2.4, 1.1, 1.1)<br>b 5.05, ddd (2.9, 1.1, 1.1)	H-13, H-17b<br>H-15, H-17a
**18**	24.9	1.23, s	H-3, H-6eq, H-5
**19**	181.8	-	-
**20**	16.1	1.01, s	H-1eq, H-14b, H-6ax, H-2b

[a] Key NOESY correlations are shown in blue text.

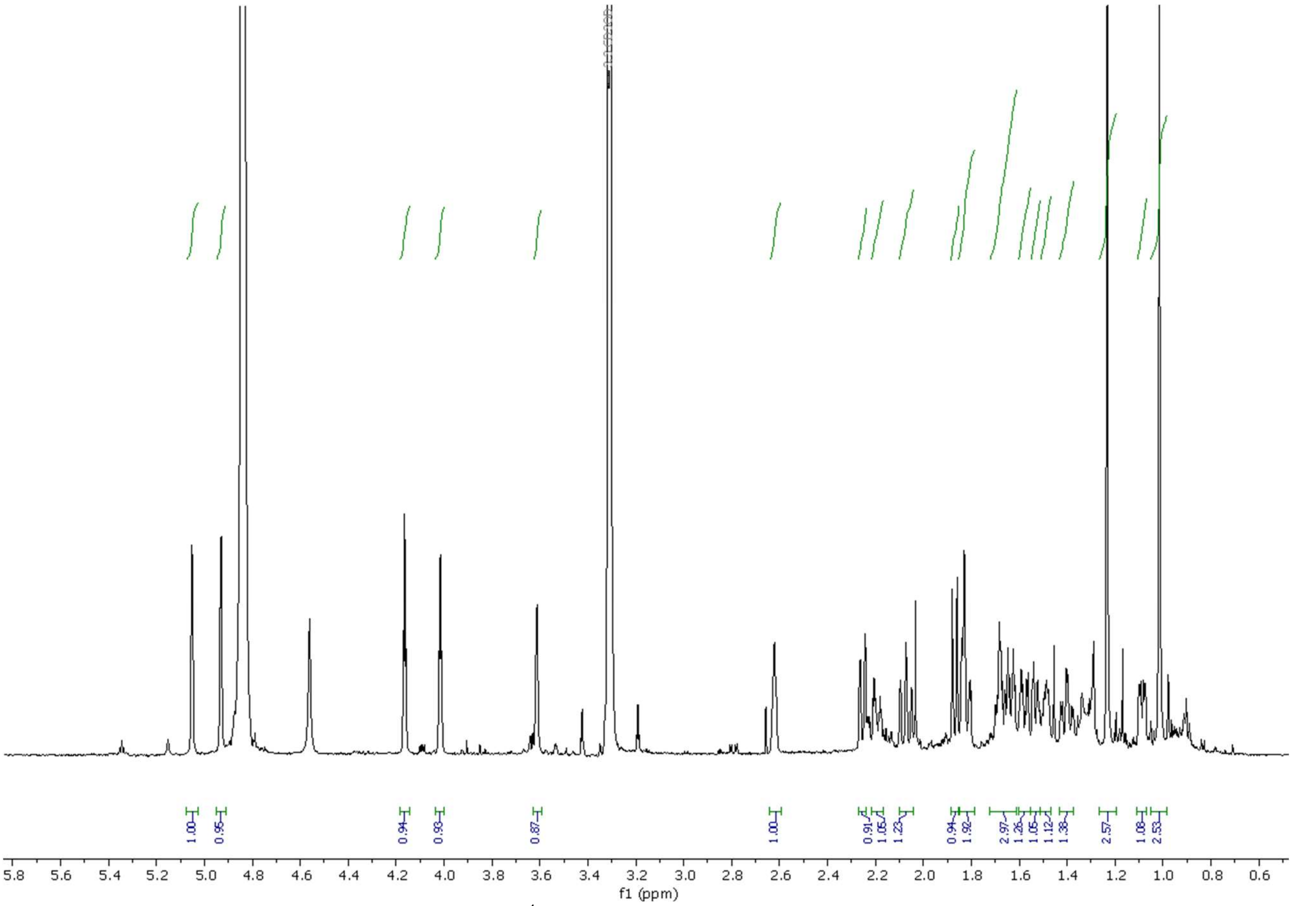

Figure N44.A ^{1}H NMR of **44** in d$_4$-methanol at 600 MHz.

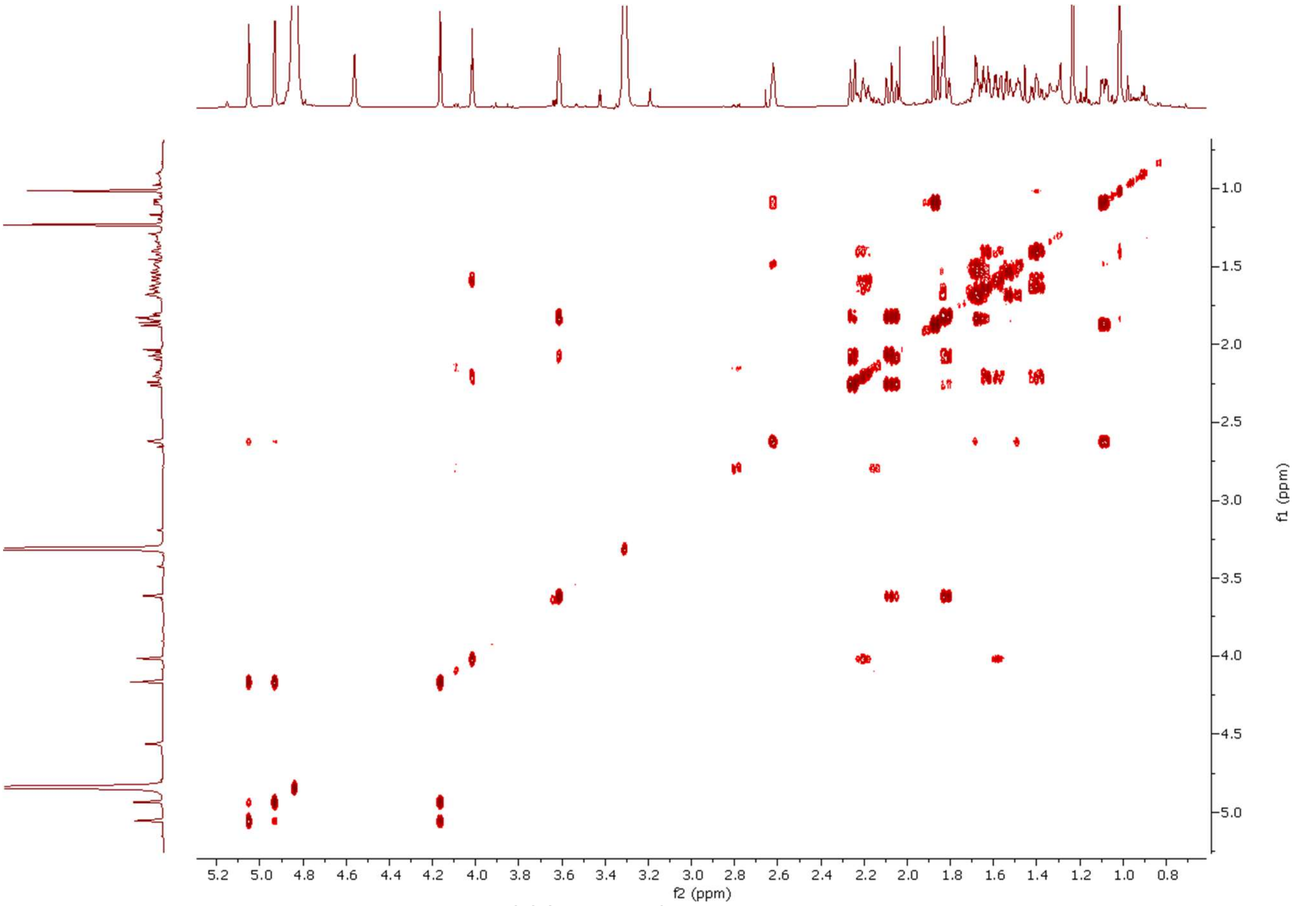

Figure N44.B COSY NMR of **44** in d$_4$-methanol at 600 MHz.

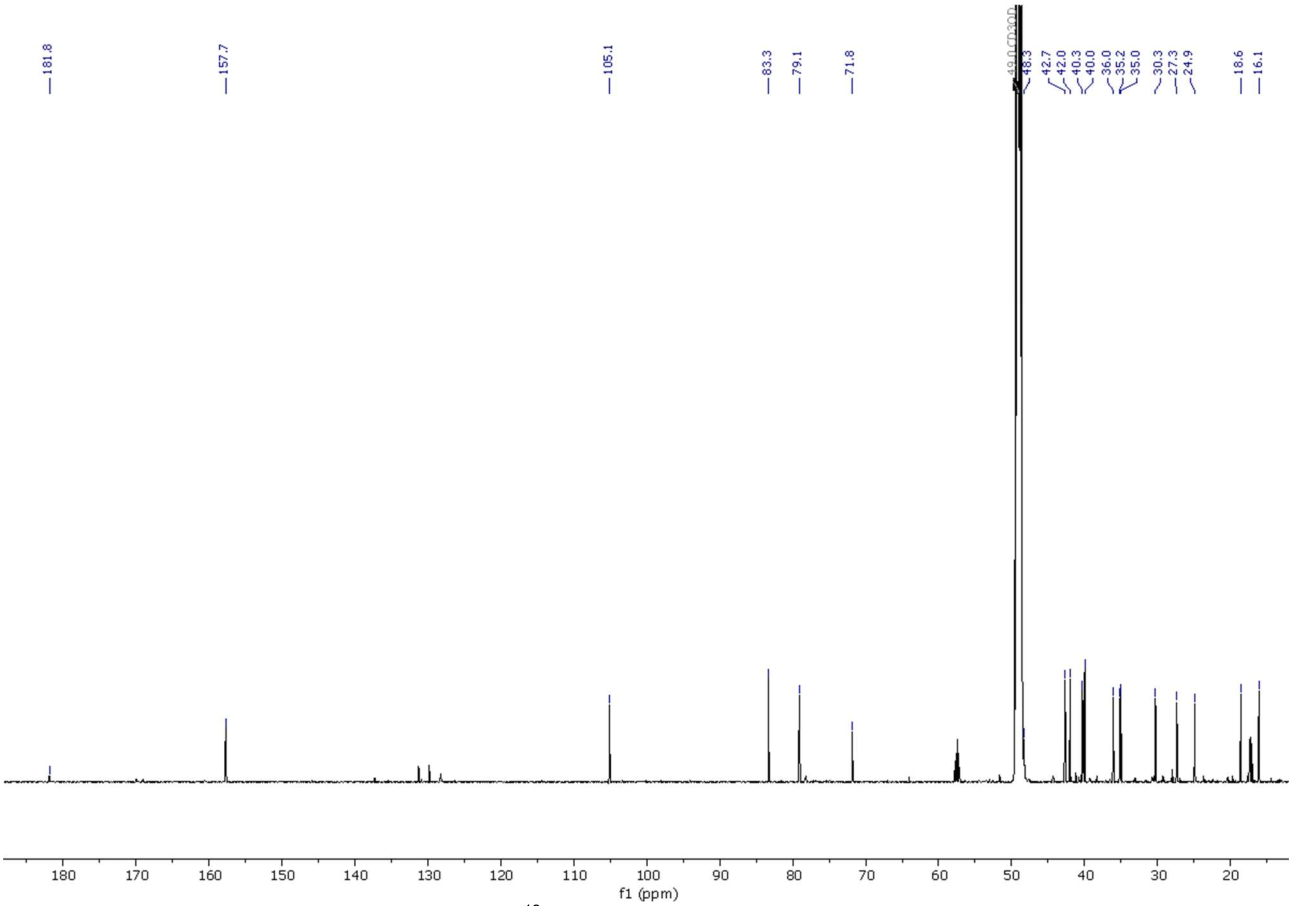

Figure N44.C ^{13}C NMR of **44** in d_4-methanol at 151 MHz.

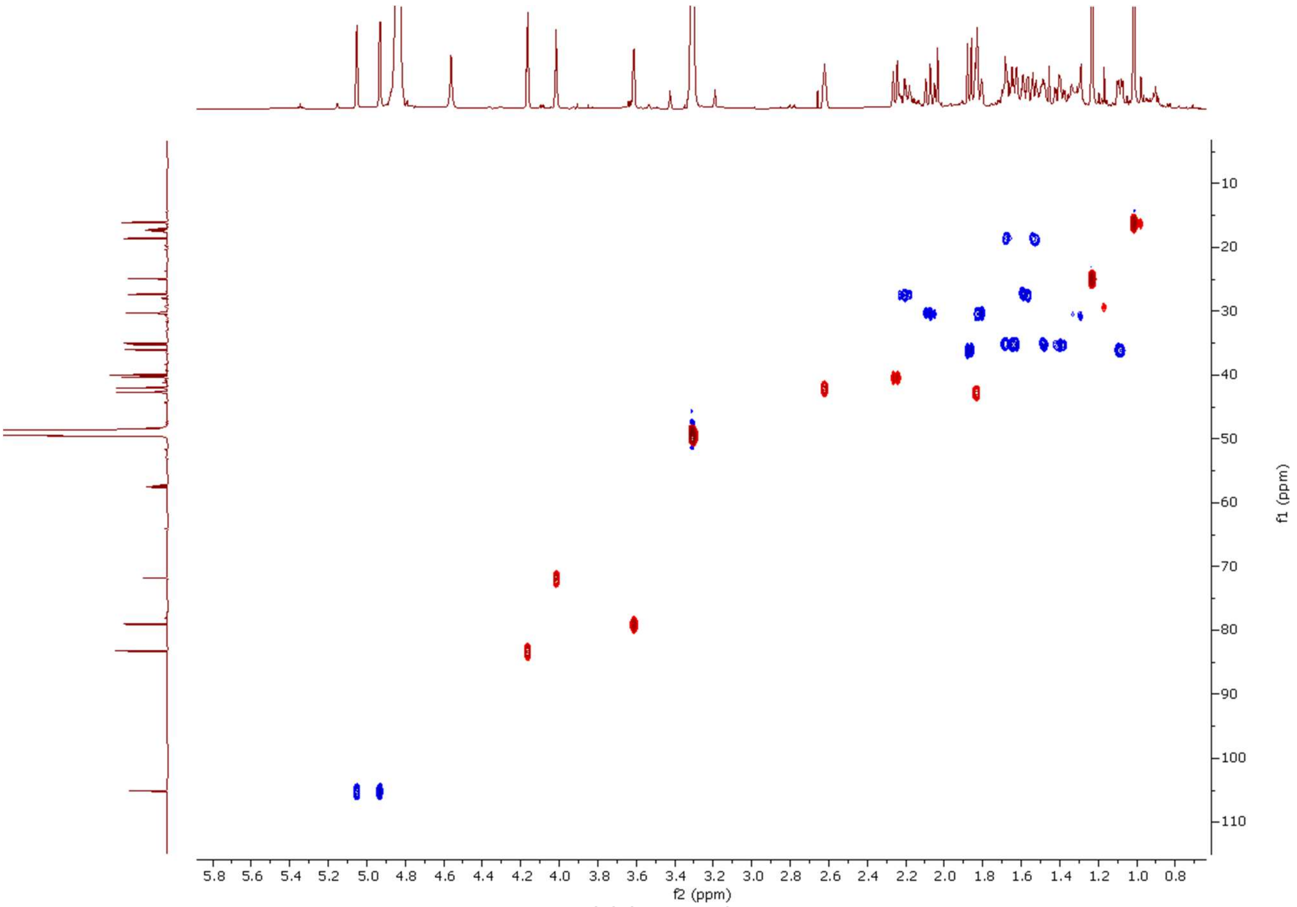

Figure N44.D HSQC NMR of **44** in d_4-methanol.

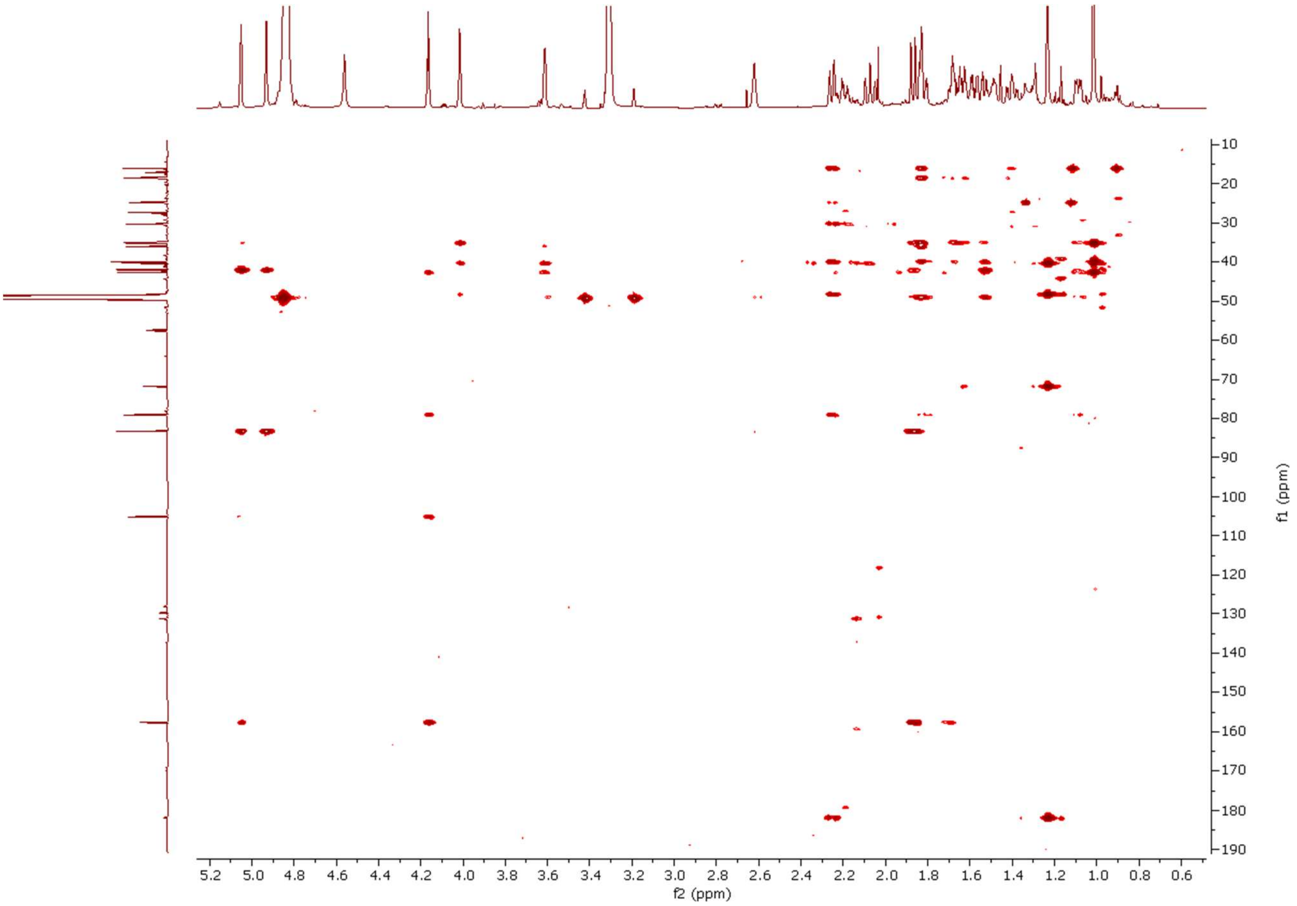

Figure N44.E HMBC NMR of **44** in d_4-methanol.

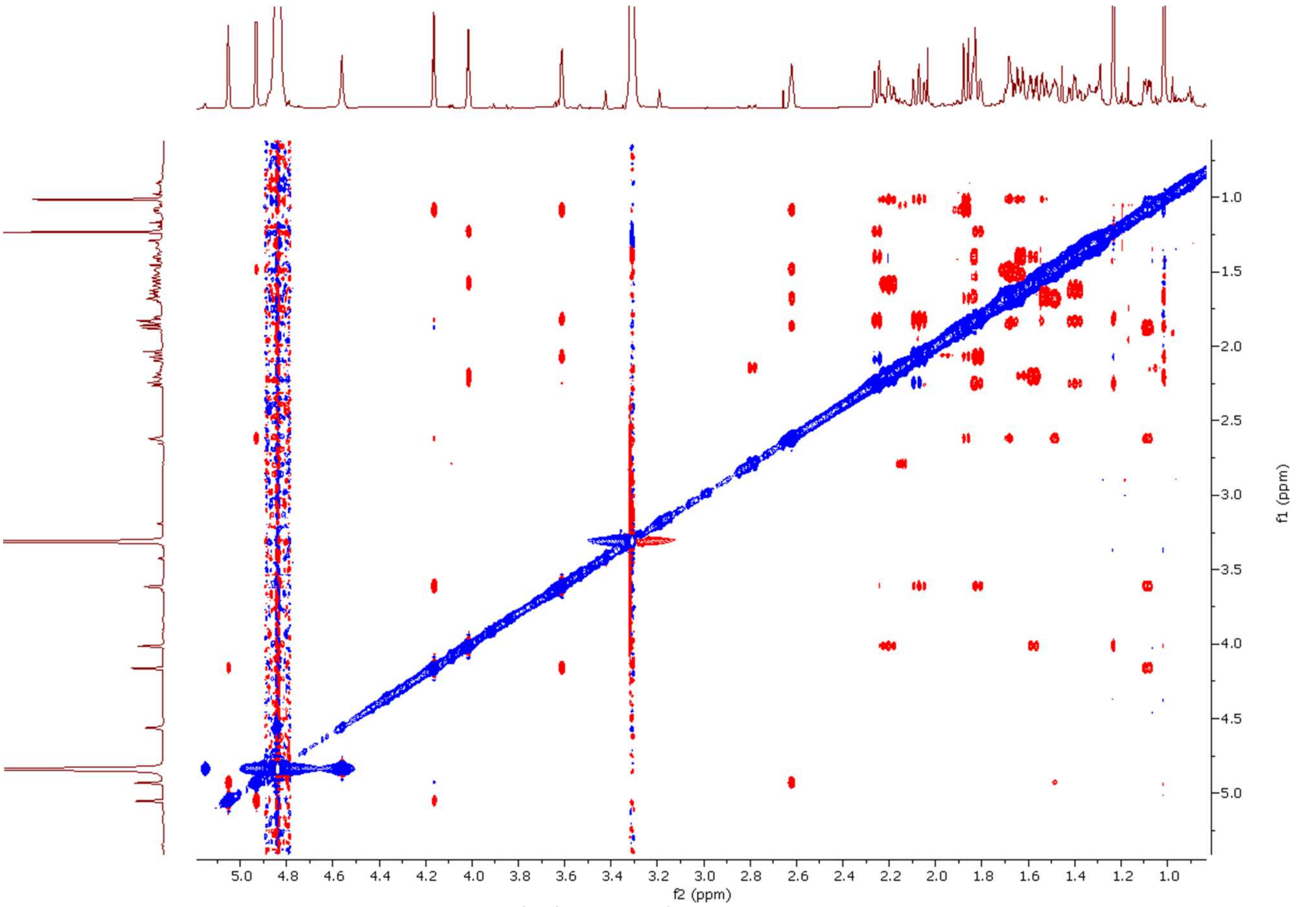

Figure N44.F NOESY NMR of **44** in d_4-methanol at 600 MHz.

6β,7β,15β-Trihydroxy-*ent*-kaurenoic acid methyl ester (45a)

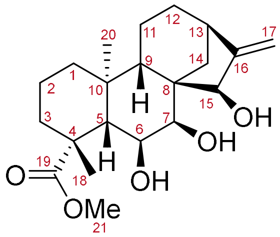

6β,7β,15β-Trihydroxy-*ent*-kaurenoic acid methyl ester (**45a**)
Chemical Formula: $C_{21}H_{32}O_5$
Exact Mass: 364.2250

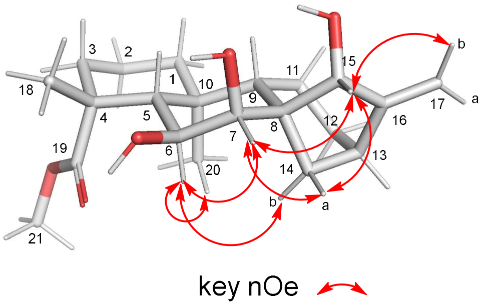

	Measured in $CDCl_3$, 298K		
**Pos.**	δ_C 151 MHz	δ_H *(J/Hz)* 600 MHz	**Selected NOESY correlations to H** [a]
**1**	40.4	ax 0.96, ddd (13.5, 13.5, 4.0)<br>eq 1.89, overlapped	H-1eq, H-5, H-9<br>H-1ax, H_3-20
**2**	19.1	a 1.44, m<br>b 1.76, overlapped	H-2b<br>H-2a
**3**	39.1	ax 1.15, ddd (13.8, 13.8, 4.5)<br>eq 2.17, ddd (13.8, 3.5, 3.5)	 H_3-18
**4**	43.9	-	-
**5**	52.1	1.87, d (10.9)	H_3-18, H-1ax, H-9
**6**	71.5	4.22, dd (10.9, 2.2)	H_3-20, H-7, H-14b
**7**	82.2	3.69, d (2.2)	H-14a, H-6, H-15
**8**	48.1	-	-
**9**	41.3	1.77, overlapped	H-5, H-1ax
**10**	40.2	-	-
**11**	17.6	a 1.51, overlapped<br>b 1.64, m	
**12**	33.8	a 1.51, m<br>b 1.56, m	
**13**	40.4	2.66, br s	H-14a, H-17a
**14**	35.2	a 1.21, dd (11.9, 4.8)<br>b 1.78, overlapped	H-15, H-13, H-7<br>H_3-20
**15**	81.3	4.32, dd (2.9, 2.5)	H-7, H-14a, H-17b
**16**	155.5	-	-
**17**	105.5	a 4.98, ddd (2.9, 1.2, 1.0)<br>b 5.15, ddd (2.5, 1.2, 1.2)	H-13<br>H-15
**18**	33.1	1.41, s	H-3eq, H-5
**19**	179.3	-	-
**20**	16.5	0.87, s	H-6, H-1eq, H_3-21, H-14b
**21**	52.1	3.74, s	H_3-20

[a] Key NOESY correlations are shown in blue text.

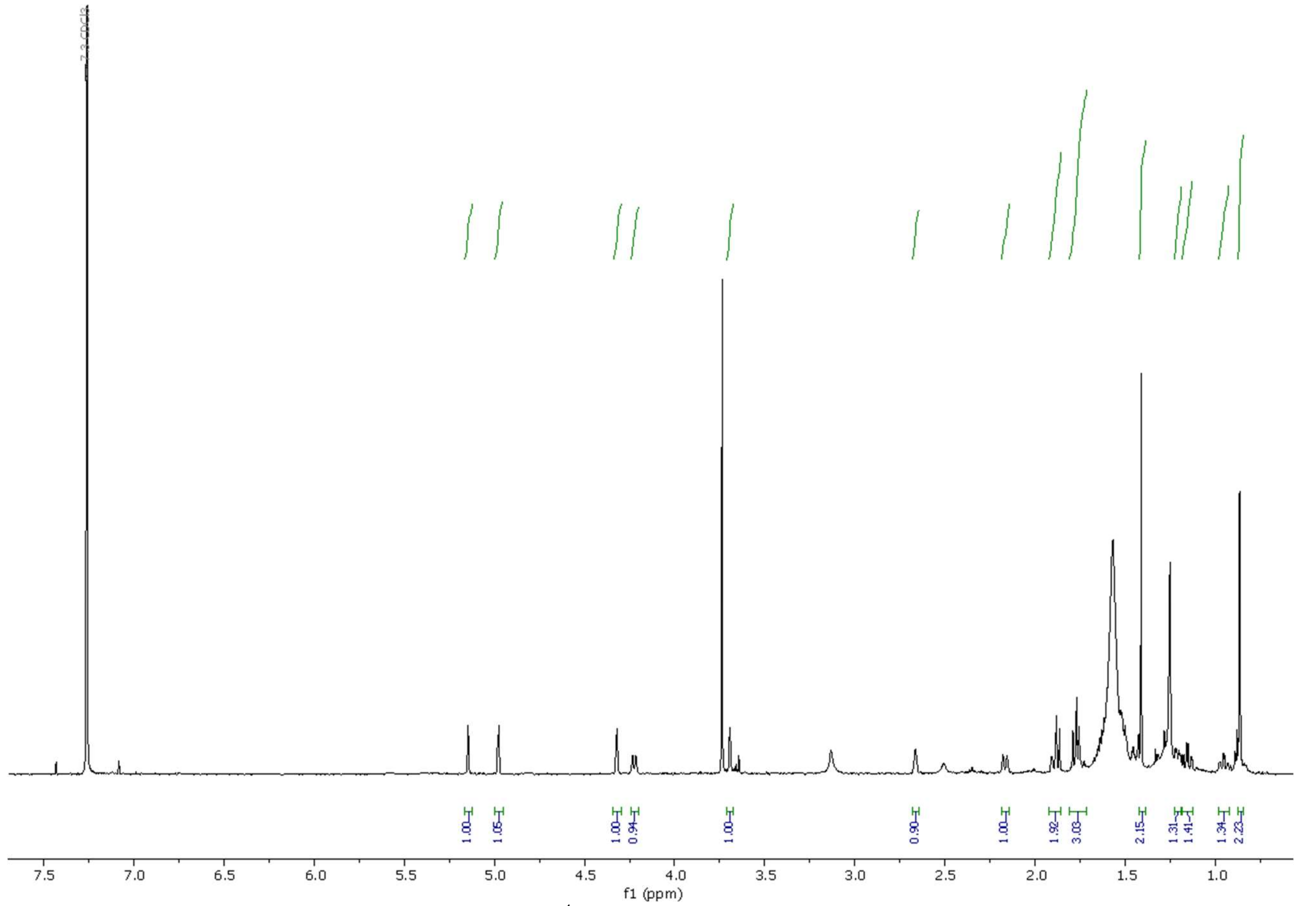

Figure N45a.A ^{1}H NMR of **45a** in $CDCl_3$ at 600 MHz.

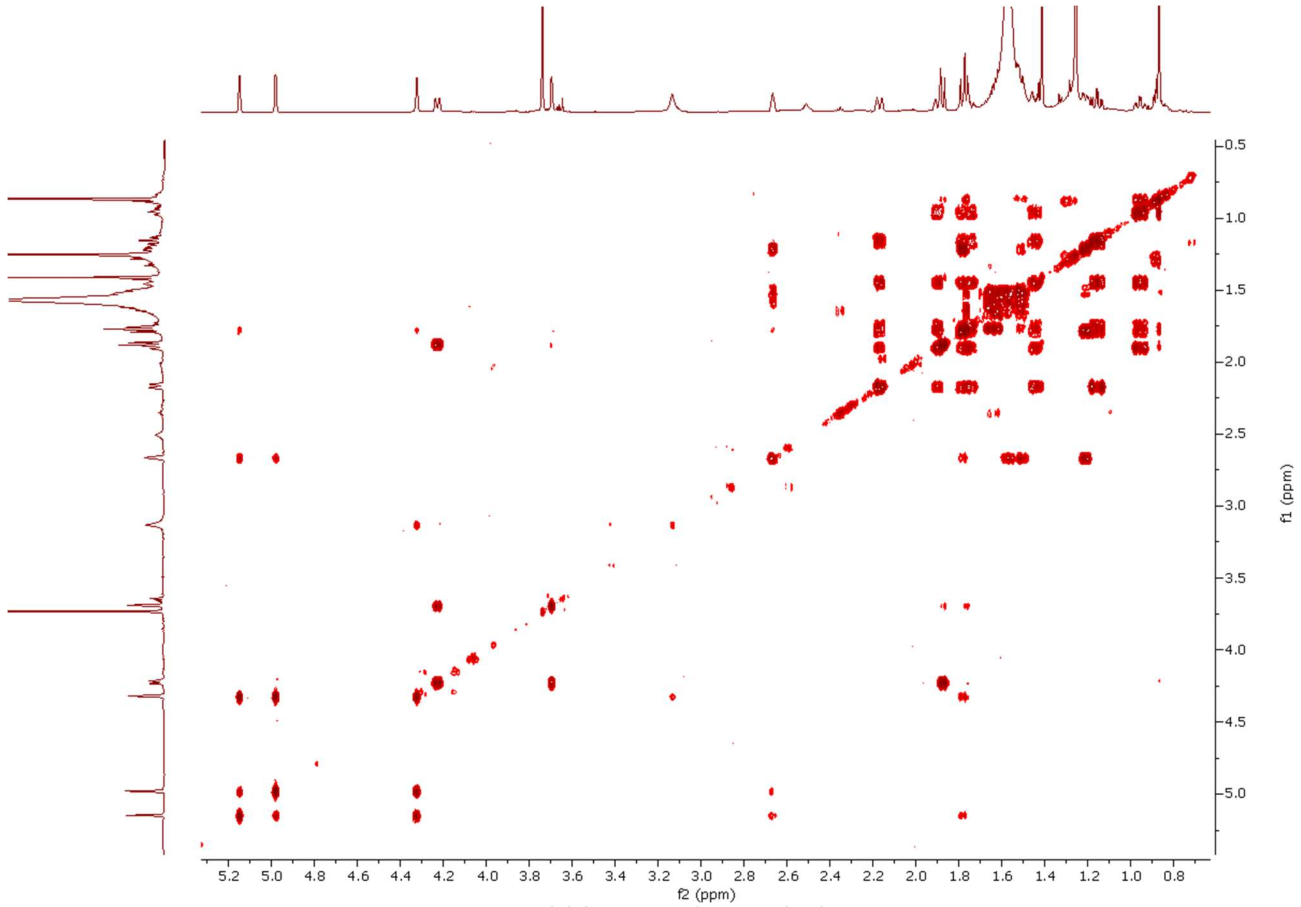

Figure N45a.B COSY NMR of **45a** in $CDCl_3$ at 600 MHz.

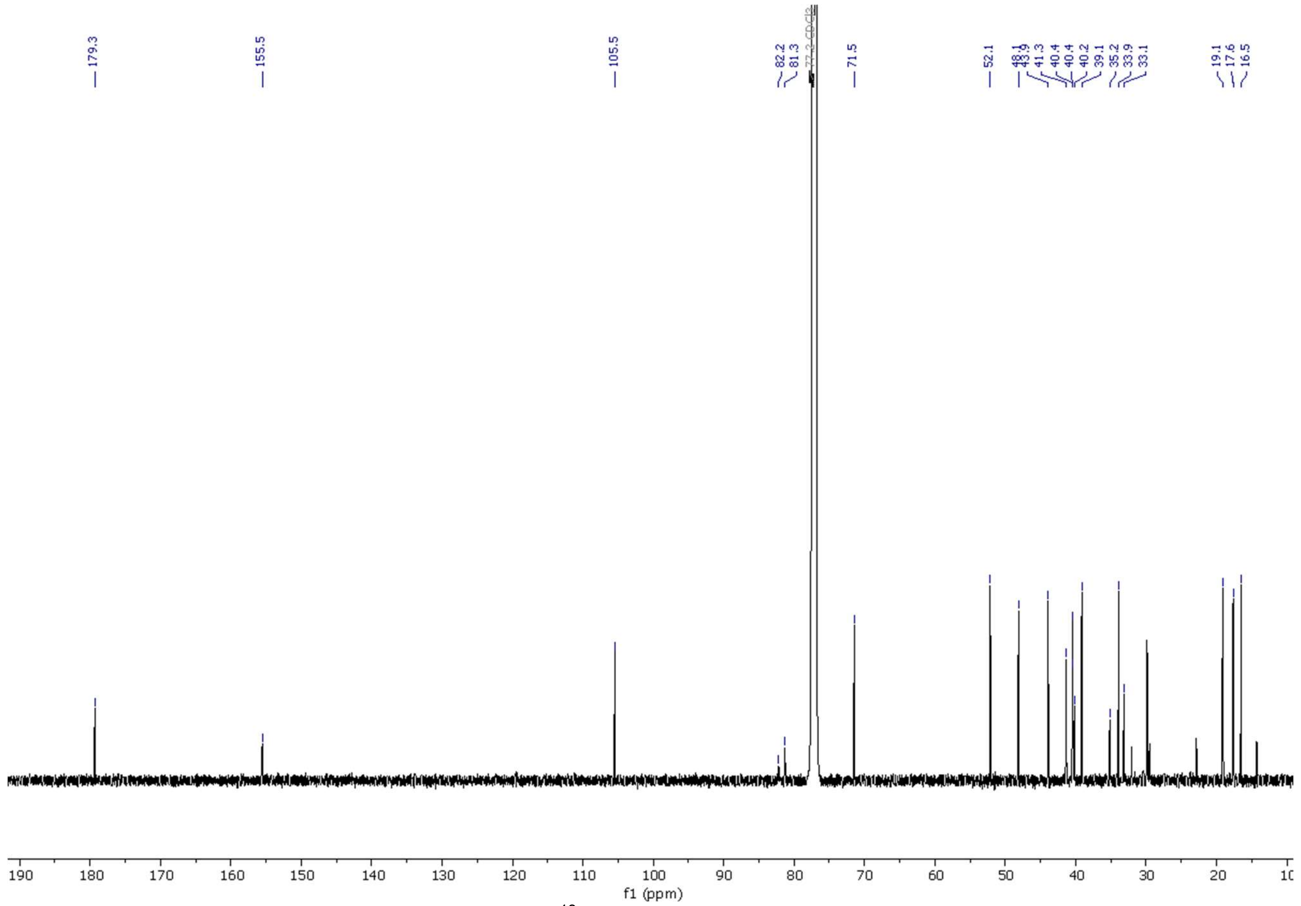

Figure N45a.C ^{13}C NMR of **45a** in $CDCl_3$ at 151 MHz.

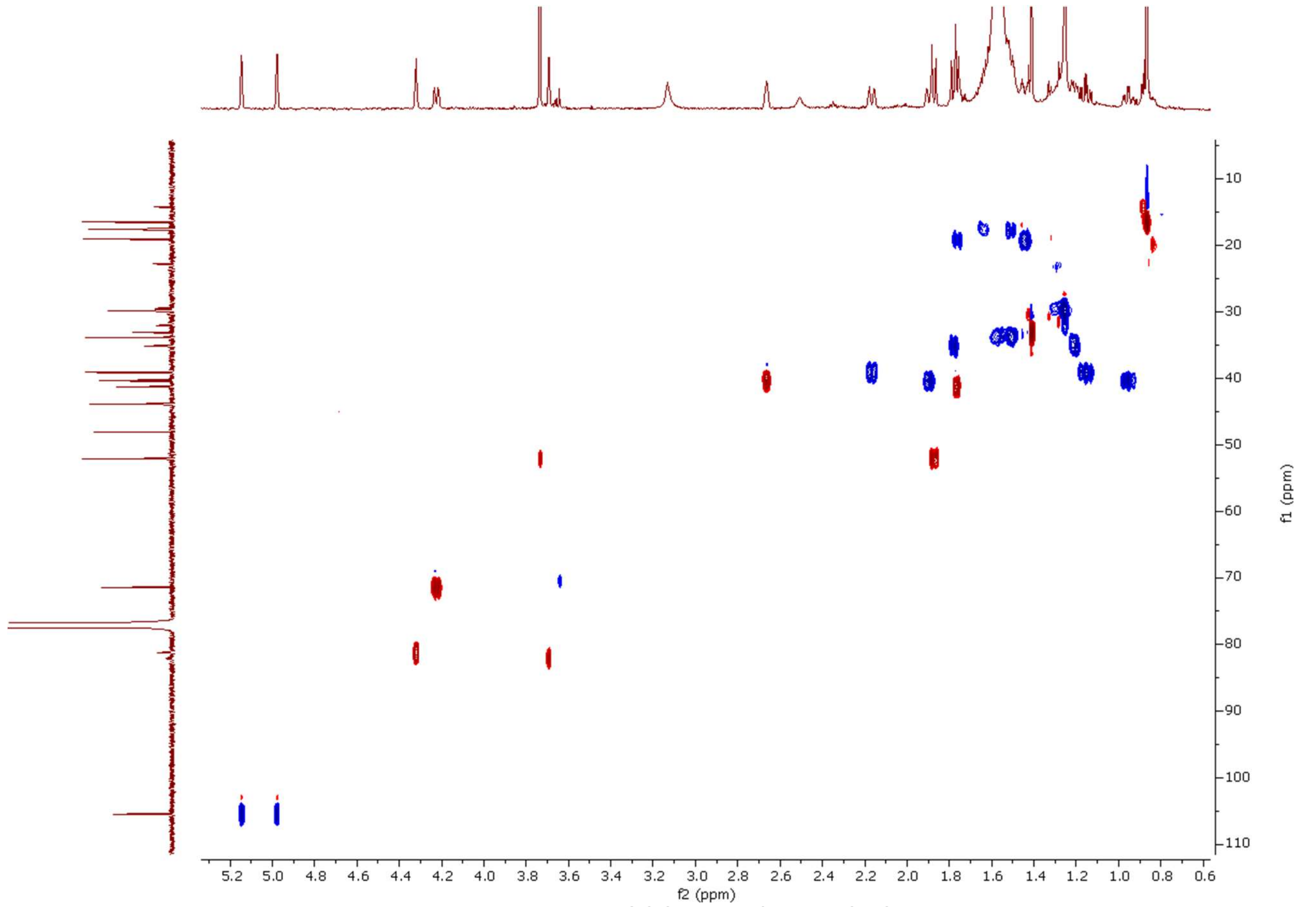

Figure N45a.D HSQC NMR of **45a** in $CDCl_3$.

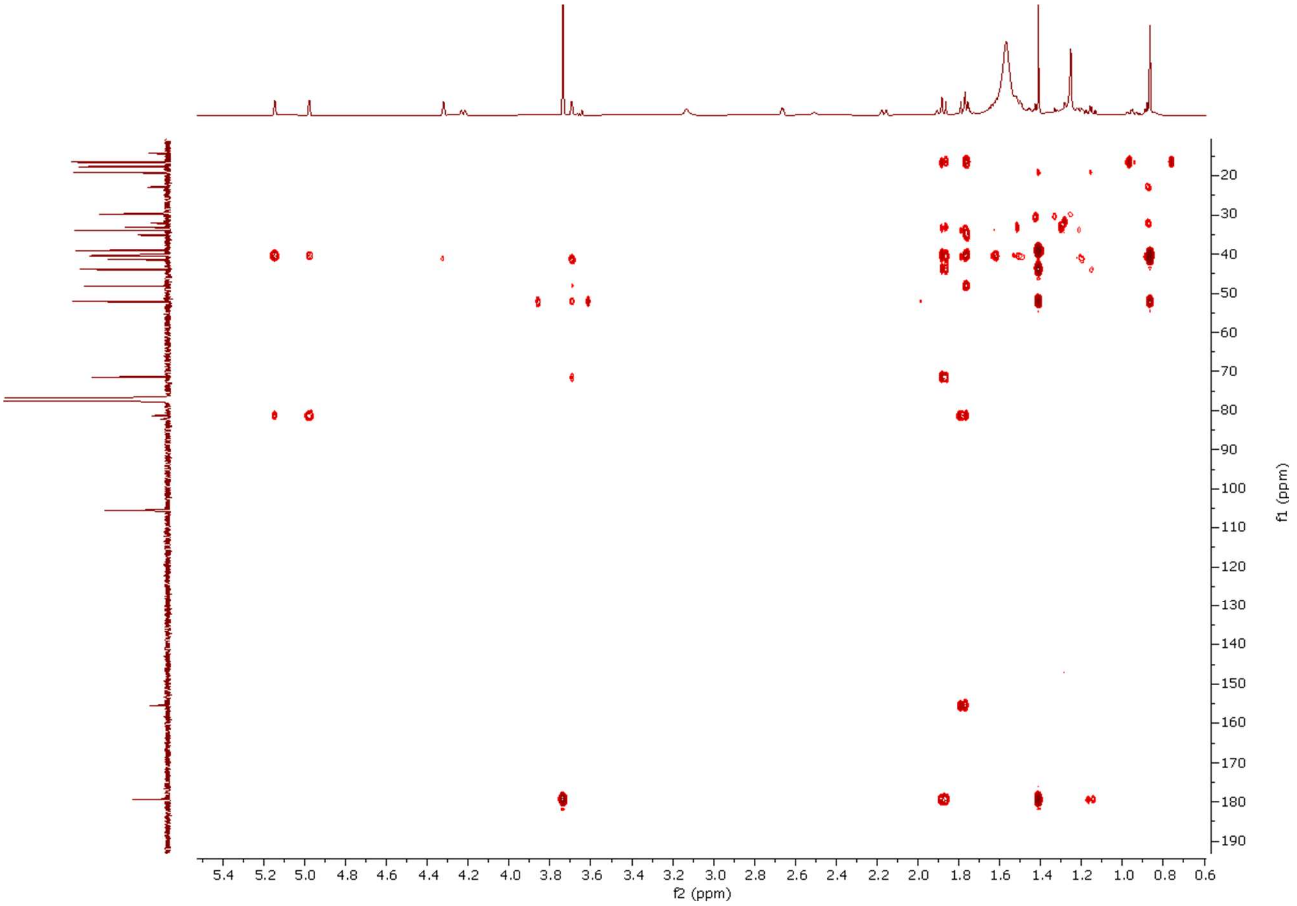

Figure N45a.E HMBC NMR of **45a** in $CDCl_3$.

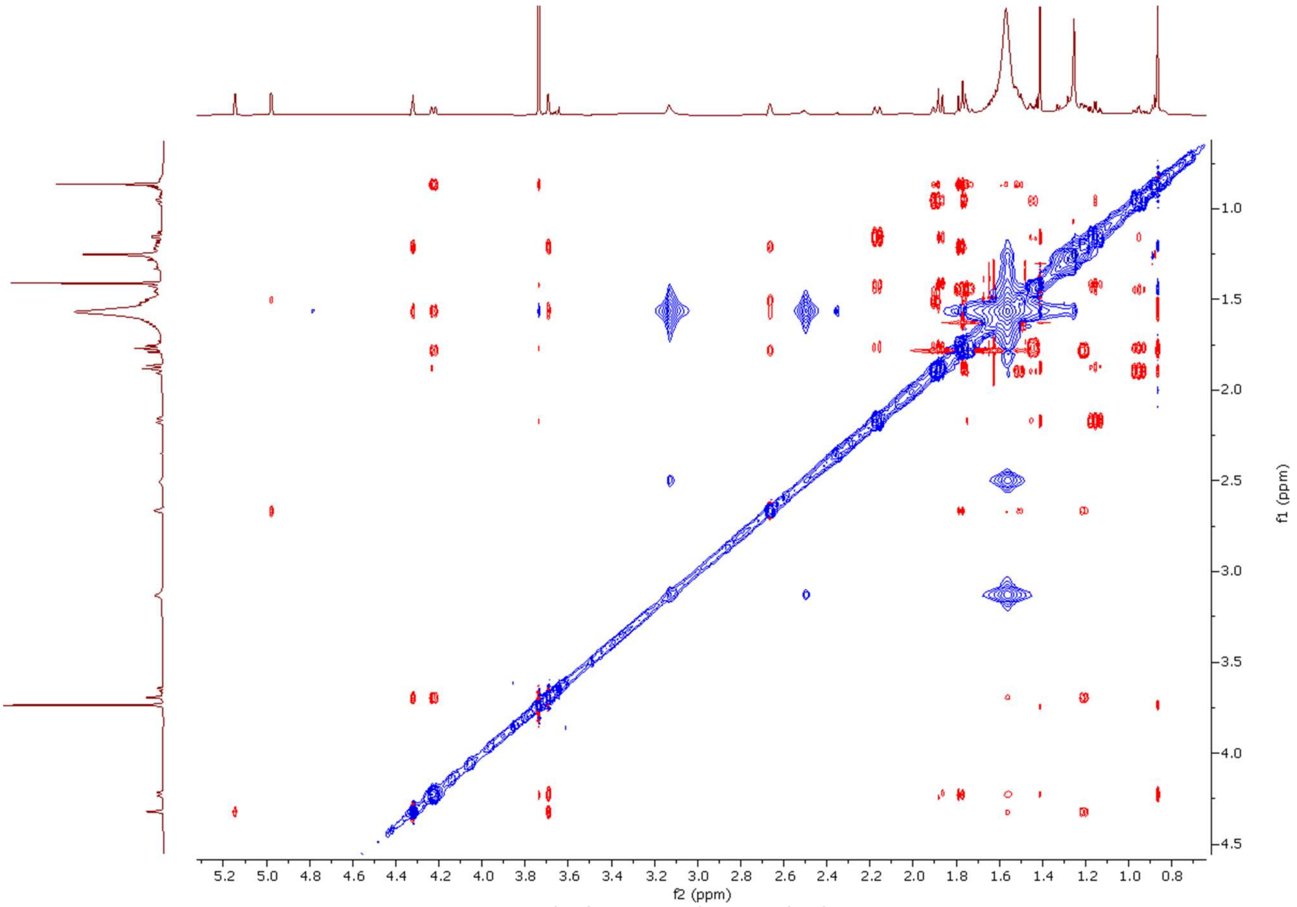

Figure N45a.F NOESY NMR of **45a** in $CDCl_3$ at 600 MHz.

Supporting References

(1) Chuang, L.; Franke, J. Rapid Combinatorial Coexpression of Biosynthetic Genes by Transient Expression in the Plant Host *Nicotiana benthamiana*. In *Engineering Natural Product Biosynthesis: Methods and Protocols*; Skellam, E., Ed.; Methods in Molecular Biology; Springer US: New York, NY, 2022; pp 395–420. https://doi.org/10.1007/978-1-0716-2273-5_20.

(2) Sun, Y.; Tian, D.; Kuhnert, E.; Goff, G. L.; Arcile, G.; Ouazzani, J.; Cox, R. J. Total Biosynthesis of Fungal Tetraketide Pyrones. *Chem. Commun.* **2023**, *59* (91), 13587–13590. https://doi.org/10.1039/D3CC04758J.

(3) Jin, F. J.; Maruyama, J.; Juvvadi, P. R.; Arioka, M.; Kitamoto, K. Development of a Novel Quadruple Auxotrophic Host Transformation System by *argB* Gene Disruption Using *adeA* Gene and Exploiting Adenine Auxotrophy in *Aspergillus oryzae*. *FEMS Microbiol. Lett.* **2004**, *239* (1), 79–85. https://doi.org/10.1016/j.femsle.2004.08.025.

(4) Alberti, F.; Khairudin, K.; Davies, J. A.; Sangmalee, S.; Willis, C. L.; Foster, G. D.; Bailey, A. M. Biosynthesis of Pleuromutilin Congeners Using an *Aspergillus oryzae* Expression Platform. *Chem. Sci.* **2023**, *14* (14), 3826–3833. https://doi.org/10.1039/D2SC06638F.

(5) Halo, L. M.; Heneghan, M. N.; Yakasai, A. A.; Song, Z.; Williams, K.; Bailey, A. M.; Cox, R. J.; Lazarus, C. M.; Simpson, T. J. Late Stage Oxidations during the Biosynthesis of the 2-Pyridone Tenellin in the Entomopathogenic Fungus *Beauveria bassiana*. *J. Am. Chem. Soc.* **2008**, *130* (52), 17988–17996. https://doi.org/10.1021/ja807052c.

(6) Sun, Y.; Gerke, J.; Becker, K.; Kuhnert, E.; Verwaaijen, B.; Wibberg, D.; Kalinowski, J.; Stadler, M.; Cox, R. J. Rapid Discovery of Terpene Tailoring Enzymes for Total Biosynthesis. *Chem. Sci.* **2023**, *14* (46), 13463–13467. https://doi.org/10.1039/D3SC04172G.

(7) Peyret, H.; Brown, J. K. M.; Lomonossoff, G. P. Improving Plant Transient Expression through the Rational Design of Synthetic 5′ and 3′ Untranslated Regions. *Plant Methods* **2019**, *15* (1), 108. https://doi.org/10.1186/s13007-019-0494-9.

(8) Sainsbury, F.; Thuenemann, E. C.; Lomonossoff, G. P. pEAQ: Versatile Expression Vectors for Easy and Quick Transient Expression of Heterologous Proteins in Plants. *Plant Biotechnol. J.* **2009**, *7* (7), 682–693. https://doi.org/10.1111/j.1467-7652.2009.00434.x.

(9) Kamileen, M. O.; Nakamura, Y.; Luck, K.; Heinicke, S.; Hong, B.; Colinas, M.; Lichman, B. R.; O'Connor, S. E. Streamlined Screening Platforms Lead to the Discovery of Pachysiphine Synthase from *Tabernanthe iboga*. *New Phytol.* **2024**, *244* (4), 1437–1449. https://doi.org/10.1111/nph.20133.

(10) Murillo, Jilmar. A.; Echeverri, F.; Quinones, W.; Torres, F.; Isaza, L.; Robledo, S. M.; Pineda, T.; Olivo, H. F.; Escobar, G. A. Synthesis, Cytotoxicity, and Leishmanicidal Evaluation of *ent*-Beyerene and *ent*-Kaurene Derivatives. *Eur. J. Org. Chem.* **2021**, *2021* (23), 3386–3397. https://doi.org/10.1002/ejoc.202001424.

(11) Hagberg, A.; Swart, P. J.; Schult, D. A. *Exploring Network Structure, Dynamics, and Function Using NetworkX*; LA-UR-08-05495; LA-UR-08-5495; Los Alamos National Laboratory (LANL), 2007. https://www.osti.gov/biblio/960616 (accessed 2025-11-05).

(12) Li, L.; Liang, W.; Rivera, M. E.; Wang, Y.-C.; Dai, M. Concise Synthesis of (−)-GA18 Methyl Ester. *J. Am. Chem. Soc.* **2023**, *145* (1), 53–57. https://doi.org/10.1021/jacs.2c12470.

(13) Sun, Y.; Shao, J.; Liu, H.; Wang, H.; Wang, G.; Li, J.; Mao, Y.; Chen, Z.; Ma, K.; Xu, L.; Wang, Y. A Chromosome-Level Genome Assembly Reveals That Tandem-Duplicated CYP706V Oxidase Genes Control Oridonin Biosynthesis in the Shoot Apex of *Isodon rubescens*. *Mol. Plant* **2023**, *16* (3), 517–532. https://doi.org/10.1016/j.molp.2022.12.007.

(14) Mitchum, M. G.; Yamaguchi, S.; Hanada, A.; Kuwahara, A.; Yoshioka, Y.; Kato, T.; Tabata, S.; Kamiya, Y.; Sun, T. Distinct and Overlapping Roles of Two Gibberellin 3-Oxidases in Arabidopsis Development. *Plant J.* **2006**, *45* (5), 804–818. https://doi.org/10.1111/j.1365-313X.2005.02642.x.

(15) Phillips, A. L.; Ward, D. A.; Uknes, S.; Appleford, NEJ.; Lange, T.; Huttly, A. K.; Gaskin, P.; Graebe, J. E.; Hedden, P. Isolation and Expression of Three Gibberellin 20-Oxidase cDNA Clones from Arabidopsis. *Plant Physiol.* **1995**, *108* (3), 1049–1057. https://doi.org/10.1104/pp.108.3.1049.

(16) Scheler, U.; Brandt, W.; Porzel, A.; Rothe, K.; Manzano, D.; Božić, D.; Papaefthimiou, D.; Balcke, G. U.; Henning, A.; Lohse, S.; Marillonnet, S.; Kanellis, A. K.; Ferrer, A.; Tissier, A. Elucidation of the Biosynthesis of Carnosic Acid and Its Reconstitution in Yeast. *Nat. Commun.* **2016**, *7* (1), 12942. https://doi.org/10.1038/ncomms12942.

(17) Frey, M.; Bathe, U.; Meink, L.; Balcke, G. U.; Schmidt, J.; Frolov, A.; Soboleva, A.; Hassanin, A.; Davari, M. D.; Frank, O.; Schlagbauer, V.; Dawid, C.; Tissier, A. Combinatorial Biosynthesis in Yeast Leads to over 200 Diterpenoids. *Metab. Eng.* **2024**, *82*, 193–200. https://doi.org/10.1016/j.ymben.2024.02.006.

(18) Watanabe, D.; Takahashi, I.; Jaroensanti-Tanaka, N.; Miyazaki, S.; Jiang, K.; Nakayasu, M.; Wada, M.; Asami, T.; Mizutani, M.; Okada, K.; Nakajima, M. The Apple Gene Responsible for Columnar Tree Shape Reduces the Abundance of Biologically Active Gibberellin. *Plant J.* **2021**, *105* (4), 1026–1034. https://doi.org/10.1111/tpj.15084.

(19) He, J.; Chen, Q.; Xin, P.; Yuan, J.; Ma, Y.; Wang, X.; Xu, M.; Chu, J.; Peters, R. J.; Wang, G. CYP72A Enzymes Catalyse 13-Hydrolyzation of Gibberellins. *Nat. Plants* **2019**, *5* (10), 1057–1065. https://doi.org/10.1038/s41477-019-0511-z.

(20) Nomura, T.; Magome, H.; Hanada, A.; Takeda-Kamiya, N.; Mander, L. N.; Kamiya, Y.; Yamaguchi, S. Functional Analysis of *Arabidopsis* CYP714A1 and CYP714A2 Reveals That They Are Distinct Gibberellin Modification Enzymes. *Plant Cell Physiol.* **2013**, *54* (11), 1837–1851. https://doi.org/10.1093/pcp/pct125.

(21) Liu, H.; Guo, S.; Lu, M.; Zhang, Y.; Li, J.; Wang, W.; Wang, P.; Zhang, J.; Hu, Z.; Li, L.; Si, L.; Zhang, J.; Qi, Q.; Jiang, X.; Botella, J. R.; Wang, H.; Song, C.-P. Biosynthesis of DHGA12 and Its Roles in *Arabidopsis* Seedling Establishment. *Nat. Commun.* **2019**, *10* (1), 1768. https://doi.org/10.1038/s41467-019-09467-5.

(22) Xiong, W.; Ye, T.; Yao, X.; Liu, X.; Ma, S.; Chen, X.; Chen, M.-L.; Feng, Y.-Q.; Wu, Y. The Dioxygenase GIM2 Functions in Seed Germination by Altering Gibberellin Production in *Arabidopsis*. *J. Integr. Plant Biol.* **2018**, *60* (4), 276–291. https://doi.org/10.1111/jipb.12619.

(23) *Gibberellins in Plants*. https://agrikaido.com/plant-hormones/gibberellins/nomenclature/ (accessed 2025-11-10).

(24) Hutchison, M.; Gaskin, P.; MacMillan, J.; Phinney, B. O. Gibberellins in Seeds of *Helianthus annuus*. *Phytochemistry* **1988**, *27* (8), 2695–2701. https://doi.org/10.1016/0031-9422(88)87046-8.

(25) Santes, C. M.; Hedden, P.; Gaskin, P.; Garcia-Martinez, JoséL. Gibberellins and Related Compounds in Young Fruits of Pea and Their Relationship to Fruit-Set. *Phytochemistry* **1995**, *40* (5), 1347–1355. https://doi.org/10.1016/0031-9422(95)00489-T.

(26) Beeley, L. J.; Gaskin, P.; Macmillan, J. Gibberellin A_{43} and Other Terpenes in Endosperm of *Echinocystis macrocarpa*. *Phytochemistry* **1975**, *14* (3), 779–783. https://doi.org/10.1016/0031-9422(75)83034-2.

(27) Toyota, M.; Odashima, T.; Wada, T.; Ihara, M. Application of Palladium-Catalyzed Cycloalkenylation Reaction to C20 Gibberellin Synthesis: Formal Syntheses of GA_{12}, GA_{111}, and GA_{112}. *J. Am. Chem. Soc.* **2000**, *122* (37), 9036–9037. https://doi.org/10.1021/ja0017413.

(28) Sugai, Y.; Miyazaki, S.; Mukai, S.; Yumoto, I.; Natsume, M.; Kawaide, H. Enzymatic Total Synthesis of Gibberellin A_4 from Acetate. *Biosci. Biotechnol. Biochem.* **2011**, *75* (1), 128–135. https://doi.org/10.1271/bbb.100733.

(29) Escamilla S, E. M.; Dendooven, L.; Magaña, I. P.; Parra S, R.; De la Torre, M. Optimization of Gibberellic Acid Production by Immobilized *Gibberella fujikuroi* Mycelium in Fluidized Bioreactors. *J. Biotechnol.* **2000**, *76* (2), 147–155. https://doi.org/10.1016/S0168-1656(99)00182-0.

(30) Lale, G.; Gadre, R. Enhanced Production of Gibberellin A_4 (GA_4) by a Mutant of *Gibberella fujikuroi* in Wheat Gluten Medium. *J. Ind. Microbiol. Biotechnol.* **2010**, *37* (3), 297–306. https://doi.org/10.1007/s10295-009-0673-1.

(31) Kang, S.-M.; Khan, A. L.; Waqas, M.; You, Y.-H.; Hamayun, M.; Joo, G.-J.; Shahzad, R.; Choi, K.-S.; Lee, I.-J. Gibberellin-Producing *Serratia nematodiphila* PEJ1011 Ameliorates Low Temperature Stress in *Capsicum annuum* L. *Eur. J. Soil Biol.* **2015**, *68*, 85–93. https://doi.org/10.1016/j.ejsobi.2015.02.005.

(32) Kildegaard, K. R.; Arnesen, J. A.; Adiego-Pérez, B.; Rago, D.; Kristensen, M.; Klitgaard, A. K.; Hansen, E. H.; Hansen, J.; Borodina, I. Tailored Biosynthesis of Gibberellin Plant Hormones in Yeast. *Metab. Eng.* **2021**, *66*, 1–11. https://doi.org/10.1016/j.ymben.2021.03.010.

(33) Lin, Y.; Liang, M.; Pang, H.; Wang, Z.; Bi, H.; Wei, Y.; Du, L. Production of Gibberellins via a Non-Natural Pathway Using Steviol as a Substrate. *J. Agric. Food Chem.* **2024**, *72* (1), 540–548. https://doi.org/10.1021/acs.jafc.3c06932.

(34) MacMillan, J.; Suter, P. J. The Occurrence of Gibberellin A_1 in Higher Plants: Isolation from the Seed of Runner Bean (*Phaseolus multiflorus*). *Naturwissenschaften* **1958**, *45* (2), 46–46. https://doi.org/10.1007/BF00635028.

(35) Tamura, S.; Takahashi, N.; Murofushi, N.; Iriuchijima, S.; Kato, J.; Wada, Y.; Watanabe, E.; Aoyama, T. Isolation and Structure of a Novel Gibberellin in Bamboo Shoots (*Phyllostachs edulis*). *Tetrahedron Lett.* **1966**, *7* (22), 2465–2472. https://doi.org/10.1016/S0040-4039(00)75677-2.

(36) Koshimizu, K.; Fukui, H.; Kusaki, T.; Mitsui, T.; Ogawa, Y. A New C20 Gibberellin in Immature Seeds of *Lupinus luteus*. *Tetrahedron Lett.* **1966**, *7* (22), 2459–2463. https://doi.org/10.1016/S0040-4039(00)75676-0.

(37) Yamaguchi, I.; Yokota, T.; Murofushi, N.; Ogawa, Y.; Takahashi, N. Isolation and Structure of a New Gibberellin from Immature Seeds of *Prunus persica*. *Agric. Biol. Chem.* **1970**, *34* (9), 1439–1441. https://doi.org/10.1080/00021369.1970.10859793.

(38) Cossey, A. L.; Lombardo, L.; Mander, L. N. Total Synthesis of Gibberellin A_4. *Tetrahedron Lett.* **1980**, *21* (45), 4383–4386. https://doi.org/10.1016/S0040-4039(00)77864-6.

(39) King, R. W.; Evans, L. T.; Mander, L. N.; Moritz, T.; Pharis, R. P.; Twitchin, B. Synthesis of Gibberellin GA_6 and Its Role in Flowering of *Lolium temulentum*. *Phytochemistry* **2003**, *62* (1), 77–82. https://doi.org/10.1016/S0031-9422(02)00447-8.

(40) Preiss, A.; Adam, G.; Šaman, D.; Buděšinsky, M. Two-Dimensional NMR Analysis of the Phytohormone Gibberellin A_3. *Magn. Reson. Chem.* **1987**, *25* (3), 239–243. https://doi.org/10.1002/mrc.1260250312.

(41) Yamaguchi, I.; Takahashi, N.; Fujita, K. Application of ^{13}C Nuclear Magnetic Resonance to the Study of Gibberellins. *J. Chem. Soc. Perkin 1* **1975**, No. 11, 992–996. https://doi.org/10.1039/P19750000992.

(42) Lewer, P.; MacMillan, J. An NMR Study of the Loss of Carbon-20 in the Biosynthesis of Gibberellin A_3 by *Gibberella fujikuroi*. *Phytochemistry* **1984**, *23* (12), 2803–2811. https://doi.org/10.1016/0031-9422(84)83019-8.

(43) Sousa, I. P.; De Sousa Teixeira, M. V.; Freitas, J. A.; Ferreira, A. G.; Pires, L. M.; Santos, R. A.; Heleno, V. C. G.; Furtado, N. A. J. C. Production of More Potent Anti-Candida Labdane Diterpenes by Biotransformation Using *Cunninghamella elegans*. *Chem. Biodivers.* **2022**, *19* (3), e202100757. https://doi.org/10.1002/cbdv.202100757.

(44) Joy, B.; Remani, P. Antitumor Constituents from *Annona squamosa* Fruit Pericarp. *Med. Chem. Res.* **2008**, *17* (2), 345–355. https://doi.org/10.1007/s00044-007-9070-3.

(45) Song, J.-L.; Yuan, Y.; Nie, L.-H.; Li, B.-L.; Qin, X.-B.; Li, Y.; Wu, J.-W.; Qiu, S.-X. A New *ent*-Kaurane Diterpene Derivative from the Stems of *Eurya chinensis* R.Br. *Nat. Prod. Res.* **2018**, *32* (2), 182–188. https://doi.org/10.1080/14786419.2017.1343327.

(46) Silva, E. A.; Takahashi, J. A.; Boaventura, M. A. D.; Oliveira, A. B. The Biotransformation of *ent*-Kaur-16-en-19-oic Acid by *Rhizopus stolonifer*. *Phytochemistry* **1999**, *52* (3), 397–400. https://doi.org/10.1016/S0031-9422(99)00219-8.

(47) Hutchison, M.; Lewer, P.; MacMillan, J. Carbon-13 Nuclear Magnetic Resonance Spectra of Eighteen Derivatives of *ent*-Kaur-16-en-19-oic Acid. *J. Chem. Soc. Perkin 1* **1984**, 2363–2366. https://doi.org/10.1039/P19840002363.

(48) Song, C.; Liu, J.; Wang, H.; Li, X.; Liu, B.; Zhang, M.; Shan, X.; Li, H.; Gao, J.; Qin, J. New Derivatives from Microbial Transformation of *ent*-Kaur-16-en-19-oic Acid by *Cunninghamella echinulata*. *Chem. Biodivers.* **2020**, *17* (6), e2000178. https://doi.org/10.1002/cbdv.202000178.

(49) Ward, J. L.; Gaskin, P.; Brown, R. G. S.; Jackson, G. S.; Hedden, P.; Phillips, A. L.; Willis, C. L.; Beale, M. H. Probing the Mechanism of Loss of Carbon-20 in Gibberellin Biosynthesis. Synthesis of Gibberellin 3α,20-Hemiacetal and 19,20-Lactol Analogues and Their Metabolism by a Recombinant GA 20-Oxidase. *J. Chem. Soc. Perkin 1* **2002**, No. 2, 232–241. https://doi.org/10.1039/B106990J.

(50) Liu, J.; Li, L.; Li, X.; Wang, X.; Zhao, X.; Qiao, Y.; Xu, Y.; Sun, Y.; Qian, L.; Liu, Z.; Ji, A.; Lou, H. Discovery of Lysosome-Targeted Covalent Anticancer Agents Based on Isosteviol Skeleton. *Eur. J. Med. Chem.* **2021**, *209*, 112896. https://doi.org/10.1016/j.ejmech.2020.112896.

(51) Croft, K. D.; Ghisalberti, E. L.; Jefferies, P. R.; Knox, J. R.; Mahoney, T. J.; Sheppard, P. N. Chemical and Microbiological Syntheses of Intermediates in Gibberellin Biosynthesis. *Tetrahedron* **1974**, *30* (19), 3663–3667. https://doi.org/10.1016/S0040-4020(01)97052-8.

(52) Rocha, A. D.; Vieira, H. da S.; Takahashi, J. A.; Boaventura, M. A. D. Synthesis of a New Allelopathic Agent from the Biotransformation of *ent*-15α-Hydroxy-16-Kauren-19-oic Acid with *Fusarium proliferatum*. *Nat. Prod. Res.* **2017**, *31* (22), 2647–2653. https://doi.org/10.1080/14786419.2017.1290614.
